# Supplementary material for: Comparative safety and efficacy of cognitive enhancers for Alzheimer’s dementia: a systematic review with individual patient data network meta-analysis
Source: BMJ Open. 2022 Apr 25;12(4):e053012. doi: 10.1136/bmjopen-2021-053012 (PMC9045061; doi:10.1136/bmjopen-2021-053012)
Supplement: Supplementary data [file bmjopen-2021-053012supp001.pdf]

Additional File 1: ***Comparative safety and efficacy of cognitive enhancers for Alzheimer's dementia: A systematic review with individual patient data network meta-analysis***

Appendix 1: Additional information on the methods used in the review ... 2

Appendix 2: Studies included in the systematic review ... 7

Appendix 3: Studies with available IPD but insufficient data to be included in the analysis ... 13

Appendix 4: List of studies requested and sponsor response ... 14

Appendix 5: Study characteristics of the included RCTs ... 17

Appendix 6. Characteristics of studies with shared IPD ... 24

Appendix 7: Comparison of studies with shared IPD with (a) all remaining studies and (b) studies for which sponsors claimed unavailable IPD. AD: aggregate data; IPD: individual patient data ... 27

Appendix 8: Cochrane Risk-of-bias appraisal results (n = 80) ... 29

Appendix 9: Overall risk of bias for studies with shared IPD against (a) all remaining studies and (b) studies for which sponsors claimed unavailable IPD. AD: aggregate data; IPD: individual patient data ... 31

Appendix 10: Study-specific effect sizes calculated from shared IPD and published data. IPD: individual patient data ... 32

Appendix 11: Correlation between participant age and dropout in studies with IPD. IPD: individual patient data ... 37

Appendix 12: Comparison Adjusted Funnel plot (all treatments vs placebo) ... 38

Appendix 13: Distribution of potential effect modifiers per treatment comparison and outcome ... 39

Appendix 14: Consistency Assessment – Loop-specific approach (using adjusted treatment effects) ... 42

Appendix 15: Network and standard meta-analysis results ... 43

Appendix 16: Network subgroup and meta-regression analysis results ... 46

Appendix 17: CINeMA results ... 53

Appendix 18: Study definitions for adverse events ... 56

Appendix 19: Time taken to achieve at least an adverse event using individual patient data ... 59

Appendix 20: Rank-heat plot for adverse events ... 60

Appendix 21: Challenges encountered during the individual patient data request from sponsors ... 61

Appendix 1: Additional information on the methods used in the review

Eligibility criteria, search strategy and study selection

We considered an adverse event (AE) as defined in the individual trials. Definitions were captured for each study separately. We included donepezil, rivastigmine, galantamine, and memantine alone or in combination with other treatment and compared with each other, supportive care or placebo. We excluded studies examining other cognitive enhancers or including individuals with mixed causes of dementia. We included published studies written in any language and of any duration.

Using terms from our previous review,4 the MEDLINE literature search was drafted by an experienced librarian (Dr. Laure Perrier) and revised after another librarian (Ms. Becky Skidmore) peer-reviewed the search terms.10 Subsequently, we searched the following databases: MEDLINE, EMBASE, Cochrane Methodology Register, CINAHL, Ageline and Cochrane Central Register of Controlled Trials. We also scanned reference lists of included studies and relevant reviews to supplement the electronic literature searches.

After pilot-testing, the results from the literature search were screened by pairs of reviewers working independently. Pairs of reviewers independently abstracted data (e.g., study characteristics, patient characteristics, outcome results) after a pilot-test. We resolved conflicts through discussion. The overall agreement among the reviewers for screening was over 70%.

IPD collection process and data abstraction

During the author contact process, two authors (a senior scientist ACT and a research assistant SL) sent a data request following several strategies as outlined in the RCT protocol:[1] a) an email requesting their IPD, b) email reminders (4 in total) at 2, 6, 10, and 14-week intervals after the initial email, c) reminders by post in week 7, and d) reminders via telephone in week 15. We also invited eligible authors to be a co-author on our updated systematic review provided that they share their anonymized IPD, and meet the International Committee of Medical Journal Editors (ICMJE) criteria for authorship.[2] Our team (AAV, SL) also contacted sponsors of the eligible trials, as reported in the publications. If a sponsor was not reported in a publication, we contacted the author (whom we emailed during the RCT) to determine who sponsored the study. To contact industry sponsors, we navigated the data sharing process from their websites or via an email, online portal, or phone inquiry. When no response was received, two follow-up reminders were sent to the sponsors.

We requested IPD on 1) patients: age, sex, severity of Alzheimer's disease (e.g. baseline MMSE [Mini-Mental State Examination] level), presence of behavioral disturbance, comorbid conditions (e.g., stroke, cardiovascular conditions, Parkinson's disease), other medications used for each patient, number of drop-outs, reasons for drop-out, and number of participants, 2) medication: treatment each patient was allocated to, dosage, 3) outcomes: event, date of event, time taken to achieve the event for AEs, MMSE values and measurement dates, and 4) date and method of randomization. We checked IPD provided for consistency with results from published RCTs., and contacted IPD providers when data inconsistencies were found.

Data extraction items included a) study characteristics: year of publication, country and continent according to the first author, journal in which the study was published, funding information; b) aggregate patient characteristics: study size and percentage of males, c) outcome data: study data (e.g., events or mean and standard deviations, and sample size per arm), and d) treatments compared. We also abstracted the corresponding authors' contact details. We categorized each study according to funding source (industry-sponsored, publicly-sponsored, mixed, and non-sponsored).

Certainty of the evidence

We used CINeMA (Confidence in Network Meta-Analysis) to assess confidence in the NMA estimates.[3] Six domains were evaluated with scores 'no concerns', 'some concerns' and 'major concerns': 1) within-study bias, 2) reporting bias, 3) indirectness, 4) imprecision, 5) heterogeneity, and 6) incoherence. We used the overall risk of bias per study, and for each treatment comparison we applied the average risk of bias. Similarly, for all treatment comparisons we used the average for indirectness. We assessed reporting bias based on the comparison-adjusted funnel plot since there are no established statistical methods to explore reporting bias. We used a comparison-adjusted funnel to account for the fact that each set of studies estimates a different summary effect in NMA. This is a scatterplot of the difference between the study-specific effect sizes from the

corresponding comparison-specific effect (obtained from standard meta-analysis) against the corresponding study-specific standard error. We used the fixed effect model for the standard meta-analysis performed for each treatment comparison, ordered treatments chronologically according to year of availability in Canada, and used only treatment comparisons versus placebo. We used the *netfunnel* command in Stata to produce the comparison-adjusted funnel plot.[4]

For imprecision, we considered a MD=1.4 and a OR=1 as a clinically important size of effect for MMSE and AE, respectively, and followed the CINeMA guidelines for exploring whether statistical significance and clinical importance coincide. Similarly, heterogeneity and incoherence (i.e. inconsistency) were assessed by following the standard CINeMA approach.

CINeMA assesses the credibility of the NMA results and heterogeneity examining the range of both confidence intervals (CIs; which do not capture heterogeneity) and prediction intervals (PIs; which capture heterogeneity) in relation to their equivalence. If a PI includes values that lead to a different conclusion than an assessment based on the corresponding CI, then this suggests that there is considerable heterogeneity. PIs are expected to include the true intervention effects in future studies with characteristics similar to the existing studies, and they incorporate the extent of between-study heterogeneity.[5 6] In the presence of considerable heterogeneity, they are wide to include intervention effects with different implications for practice. However, caution is needed in the interpretation of results in the presence of funnel plot asymmetry, since PIs are based on the assumption of a normal distribution for the study-specific effects and as such they may be problematic if the data do not follow a normal distribution.

Statistical Analysis

We performed a descriptive analysis using frequencies and percentages of the discrete characteristics of the included patients and treatments of the eligible studies. We explored the distributions of the continuous patient characteristics per outcome and treatment group using means and standard deviations. For studies not providing outcome results for a certain outcome, we presented distributions of the available and requested patient characteristics, whenever available. Outliers for each patient characteristic were also explored in each study dataset using boxplots. We also recorded the number of missing participants per treatment group and overall. We compared the characteristics of the unavailable and the available by the sponsors' studies. In particular, we explored whether these were well-conducted according to overall risk of bias, and compared distributions of mean participant age, publication year, study duration, study size, percent male, and magnitude of treatment effect, to assess for potential bias in IPD sharing. We conducted a two-stage analysis for both standard meta-analysis and NMA. The network geometry was explored through the presentation of network plots.

First stage

All IPD from included studies were first aggregated to study-level summary statistics using each sponsor's portal. The use of different platforms and failure to obtain IPD from all studies restricted us from combining IPD in a one-stage analysis. For each separate study with IPD available, we fitted a logistic regression model for the binary outcome and a linear regression model for the continuous outcome. For MMSE, we considered the longest duration of follow-up per study (most frequently at week 24). In the shared IPD, when we were unable to make a judgement on first and last date of visit per patient, we used the older coded date and the newest coded date as baseline and final value for each patient respectively.

Initially, we did not adjust for any of the patient characteristics provided, but in a subsequent analysis we included patient-level covariates with as many interaction terms in the model as the patient characteristics were provided (considering only the ones we have asked for). For each study, we obtained the adjusted odds ratio (OR) for binary data and adjusted mean difference (MD) for continuous data, along their corresponding 95% CI. We adjusted for any of the following variables that were available in each study: age, sex, severity of Alzheimer's disease (e.g., baseline Mini-Mental State Examination [MMSE] level), presence of behavioural disturbance, comorbidity, and other medications. The first stage of the IPD analyses were conducted in RStudio,[7] which was available in data providers. Additional medications and comorbid conditions were grouped into broader categories according to their clinical relevance to increase power in our analysis (e.g., grouped medications as anti-psychotics, anti-depressants, and cognitive enhancers, as well as comorbid conditions as psychiatric, neurological, and cardiac disorders). Eligible studies with insufficient data to derive a pairwise estimate for NMA were summarized descriptively without performing a statistical analysis.

We applied an available case analysis for each study, since we were unable to install R packages in most sponsor-specific platforms, and hence we applied a consistent approach across all IPD datasets. We explored the impact of missing data during the second stage of analysis. Reasons for missing participants and time taken to have a adverse event were captured (when available).

We synthesized IPD at the first stage in four different proprietary sponsor-specific platforms. Analyses were conducted in the RStudio using different R versions[7] according to what was provided in each sponsor's platform: R version 3.4.1 for AbbVie, R version 3.4.3 for CSDR, R version 3.5.1 for YODA, R version 3.6.0 for Lundbeck.

Second stage

Since we were not successful in obtaining IPD for all eligible studies, we combined both IPD and aggregate data in a single meta-analysis or NMA model. Both IPD and aggregate data studies shared the same amount of heterogeneity. In both meta-analysis and NMA models, we combined the adjusted IPD estimates with the aggregate data (main analysis). As a secondary analysis, we combined the unadjusted estimates from retrieved IPD with the evidence provided by the aggregated data studies in a joint NMA model. A common-within network between-study variance was assumed across comparisons for all NMA models.[8] We estimated the between-study variance using the DerSimonian and Laird[9] method and compared it with the relevant distributions provided by Turner et al[10] and Rhodes et al[11] to assess heterogeneity. We also calculated I^2 on the NMA level to quantify overall heterogeneity and inconsistency in each outcome.

To assess the validity of the transitivity assumption for each outcome, we assessed the distribution of potential effect modifiers (e.g., age, sex) across treatment comparisons in each network.[12-14] We visually inspected similarity and assessed whether these characteristics were likely to modify the treatment effect. We evaluated the consistency assumption using the design-by-treatment interaction model[15 16] and the loop-specific method.[17 18] In the presence of statistically significant inconsistency, we checked the data for discrepancies and if none were identified, we planned to conduct subgroup NMA or network meta-regression analysis adjusting for potential variables influencing the results.

We conducted additional NMA analyses for all potential effect modifiers requested from data providers. If relevant data were not available in the IPD, we used aggregate data of the relevant publications. Additional NMA analyses included: 1) subgroup analysis for industry vs. publicly sponsored studies, for studies with available IPD vs. studies with aggregate data (unadjusted estimates), and for AD severity, classified according to MMSE scores using the National Institute for Health and Care Excellence categories: mild (21–24), moderate (10–20), severe (<10),[19] 2) network meta-regression accounting for study duration, year of publication, mean age, and sex (% of male participants) effect modifiers separately and assuming a common regression coefficient across comparisons (studies with aggregate data were used only; studies with available IPD were pooled in a NMA separately adjusted for available covariates at first stage), 3) sensitivity analysis including studies with low risk of bias for allocation concealment and incomplete outcome data items, as these items may have an important impact on the meta-analysis results according to our previous NMA,[20] and 4) the 'informative missingness difference of means' (IMDoM) imputation method[21] for MMSE for the aggregate data studies to assess the impact of missing data in our NMA. In all additional NMA analyses, we used the adjusted effect estimates derived from the IPD within-study analysis and the aggregate data extracted from the eligible publications. Network meta-regression was performed in a Bayesian setting using OpenBUGS version 3.2.3, non-informative priors for all parameters in the model and a half-normal prior for the between standard deviation. We compared the results of the additional models by evaluating the treatment effect estimates and ranking statistics, as well as monitoring the reduction in the between-study variance.

We present the results using summary effect sizes, and in particular the MD for MMSE and the OR for AE, along with their corresponding CIs and PIs.[6] We ranked the interventions for each outcome according to their efficacy and safety using P-scores in frequentist analyses and SUCRAs (surface under the cumulative ranking curve) in Bayesian analyses (e.g., meta-regression analysis).[22 23] SUCRA is the numeric presentation of the intervention ranking and is based on the surface under the cumulative ranking probability function for each treatment. An equivalent frequentist statistic is the P-score measure that is based on the observed treatment effect estimates and their uncertainty. Both measures summarize the estimated probabilities for all possible ranks, account for uncertainty in relative ranking, and range between 0-100%, with 100% reflecting the best intervention with no uncertainty and 0% reflecting the worst intervention with no uncertainty. Ranking strategies are commonly encountered in NMAs,[24-26] and we present the hierarchy of cognitive enhancers in a rank-heat plot.[27]

Meta-analysis and NMA at the 2nd stage were conducted in the RStudio using R version 3.6.2 and the *meta*[28] and *netmeta*[29] packages, respectively.

Appendix 1 References:

1. Veroniki AA, Straus SE, Ashoor H, et al. Contacting authors to retrieve individual patient data: study protocol for a randomized controlled trial. *Trials* 2016;17(1):138. doi: 10.1186/s13063-016-1238-z [published Online First: 2016/03/16]
2. Hager K, Baseman AS, Nye JS, et al. Effects of galantamine in a 2-year, randomized, placebo-controlled study in Alzheimer's disease. *Neuropsychiatric disease and treatment* 2014;10:391-401. doi: 10.2147/ndt.s57909 [published Online First: 2014/03/05]
3. CINeMA: Confidence in Network Meta-Analysis. Institute of Social and Preventive Medicine, University of Bern. *Available from cinemaispmch* 2017
4. Chaimani A, Higgins JP, Mavridis D, et al. Graphical tools for network meta-analysis in STATA. *PLoS One* 2013;8(10):e76654. doi: 10.1371/journal.pone.0076654 [published Online First: 2013/10/08]
5. Higgins JP, Thompson SG, Spiegelhalter DJ. A re-evaluation of random-effects meta-analysis. *J R Stat Soc Ser A Stat Soc* 2009;172(1):137-59. doi: 10.1111/j.1467-985X.2008.00552.x [published Online First: 2009/04/22]
6. Riley RD, Higgins JP, Deeks JJ. Interpretation of random effects meta-analyses. *Bmj* 2011;342:d549. doi: 10.1136/bmj.d549 [published Online First: 2011/02/12]
7. R Core Team. R: A Language and Environment for Statistical Computing. Vienna, Austria: R Foundation for Statistical Computing, 2019.
8. Lu G, Ades AE. Combination of direct and indirect evidence in mixed treatment comparisons. *Statistics in medicine* 2004;23(20):3105-24. doi: 10.1002/sim.1875 [published Online First: 2004/09/28]
9. DerSimonian R, Laird N. Meta-analysis in clinical trials. *Controlled clinical trials* 1986;7(3):177-88. doi: 10.1016/0197-2456(86)90046-2
10. Turner RM, Davey J, Clarke MJ, et al. Predicting the extent of heterogeneity in meta-analysis, using empirical data from the Cochrane Database of Systematic Reviews. *International journal of epidemiology* 2012;41(3):818-27. doi: 10.1093/ije/dys041 [published Online First: 2012/03/31]
11. Rhodes KM, Turner RM, Higgins JP. Predictive distributions were developed for the extent of heterogeneity in meta-analyses of continuous outcome data. *Journal of clinical epidemiology* 2015;68(1):52-60. doi: 10.1016/j.jclinepi.2014.08.012 [published Online First: 2014/10/12]
12. Salanti G. Indirect and mixed-treatment comparison, network, or multiple-treatments meta-analysis: many names, many benefits, many concerns for the next generation evidence synthesis tool. *Res Synth Methods* 2012;3(2):80-97. doi: 10.1002/jrsm.1037 [published Online First: 2012/06/11]
13. Jansen JP, Naci H. Is network meta-analysis as valid as standard pairwise meta-analysis? It all depends on the distribution of effect modifiers. *BMC Med* 2013;11:159. doi: 10.1186/1741-7015-11-159 [published Online First: 2013/07/04]
14. Cipriani A, Higgins JP, Geddes JR, et al. Conceptual and technical challenges in network meta-analysis. *Annals of internal medicine* 2013;159(2):130-7. doi: 10.7326/0003-4819-159-2-201307160-00008
15. Higgins JP, Jackson D, Barrett JK, et al. Consistency and inconsistency in network meta-analysis: concepts and models for multi-arm studies. *Res Synth Methods* 2012;3(2):98-110. doi: 10.1002/jrsm.1044 [published Online First: 2012/06/01]
16. White IR BJ, Jackson D, Higgins JPT. Consistency and inconsistency in network meta-analysis: model estimation using multivariate meta-regression. *Res Synth Methods* 2012;3(2):15.
17. Song F, Altman DG, Glenny AM, et al. Validity of indirect comparison for estimating efficacy of competing interventions: empirical evidence from published meta-analyses. *Bmj* 2003;326(7387):472. doi: 10.1136/bmj.326.7387.472 [published Online First: 2003/03/01]
18. Veroniki AA, Vasiliadis HS, Higgins JP, et al. Evaluation of inconsistency in networks of interventions. *International journal of epidemiology* 2013;42(1):332-45. doi: 10.1093/ije/dys222
19. National Institute for Health and Clinical Excellence. Donepezil, galantamine, rivastigmine and memantine for the treatment of Alzheimer's disease. Review of NICE technology appraisal guidance 111 NICE technology appraisal guidance 217. London, UK, 2011.
20. Veroniki AA, Straus SE, Ashoor HM, et al. Comparative safety and effectiveness of cognitive enhancers for Alzheimer's dementia: protocol for a systematic review and individual patient data network meta-analysis. *BMJ open* 2016;6(1):e010251. doi: 10.1136/bmjopen-2015-010251

21. Mavridis D, White IR, Higgins JP, et al. Allowing for uncertainty due to missing continuous outcome data in pairwise and network meta-analysis. *Statistics in medicine* 2015;34(5):721-41. doi: 10.1002/sim.6365 [published Online First: 2014/11/14]
22. Rucker G, Schwarzer G. Ranking treatments in frequentist network meta-analysis works without resampling methods. *BMC Med Res Methodol* 2015;15:58. doi: 10.1186/s12874-015-0060-8 [published Online First: 2015/08/01]
23. Salanti G, Ades AE, Ioannidis JP. Graphical methods and numerical summaries for presenting results from multiple-treatment meta-analysis: an overview and tutorial. *J Clin Epidemiol* 2011;64(2):163-71. doi: 10.1016/j.jclinepi.2010.03.016 [published Online First: 2010/08/07]
24. Mbuagbaw L, Rochwerg B, Jaeschke R, et al. Approaches to interpreting and choosing the best treatments in network meta-analyses. *Syst Rev* 2017;6(1):79. doi: 10.1186/s13643-017-0473-z [published Online First: 2017/04/14]
25. Petropoulou M, Nikolakopoulou A, Veroniki AA, et al. Bibliographic study showed improving statistical methodology of network meta-analyses published between 1999 and 2015. *J Clin Epidemiol* 2017;82:20-28. doi: 10.1016/j.jclinepi.2016.11.002 [published Online First: 2016/11/20]
26. Veroniki AA, Straus SE, Rucker G, et al. Is providing uncertainty intervals in treatment ranking helpful in a network meta-analysis? *J Clin Epidemiol* 2018;100:122-29. doi: 10.1016/j.jclinepi.2018.02.009 [published Online First: 2018/02/13]
27. Veroniki AA, Straus SE, Fyraridis A, et al. The rank-heat plot is a novel way to present the results from a network meta-analysis including multiple outcomes. *Journal of clinical epidemiology* 2016;76:193-9. doi: 10.1016/j.jclinepi.2016.02.016 [published Online First: 2016/03/05]
28. Balduzzi S, Rücker G, Schwarzer G. How to perform a meta-analysis with R: a practical tutorial. *Evidence-based mental health* 2019;22(4):153-60. doi: 10.1136/ebmental-2019-300117 [published Online First: 2019/09/30]
29. Rücker G, Schwarzer G, Krahn U, et al. netmeta: Network Meta-Analysis using Frequentist Methods. R package version 0.9-8. 2018 [Available from: https://CRAN.R-project.org/package=netmeta.

Appendix 2: Studies included in the systematic review

80 Main Studies:

30. Agid Y, Dubois B, Anand R, Gharabawi G, International Rivastigmine Investigators. Efficacy and tolerability of rivastigmine in patients with dementia of the Alzheimer type. *Current Therapeutic Research* 1998; **59**(12): 837-45.
31. Ancoli-Israel S, Amatniek J, Ascher S, Sadik K, Ramaswamy K. Effects of galantamine versus donepezil on sleep in patients with mild to moderate Alzheimer disease and their caregivers: a double-blind, head-to-head, randomized pilot study. *Alzheimer disease and associated disorders* 2005; **19**(4): 240-5.
32. Andersen F, Viitanen M, Halvorsen DS, Straume B, Wilsgaard T, Engstad TA. The effect of stimulation therapy and donepezil on cognitive function in Alzheimer's disease. A community based RCT with a two-by-two factorial design. *BMC neurology* 2012; **12**: 59.
33. Araki T, Wake R, Miyaoka T, et al. The effects of combine treatment of memantine and donepezil on Alzheimer's disease patients and its relationship with cerebral blood flow in the prefrontal area. *International journal of geriatric psychiatry* 2014; **29**(9): 881-9.
34. Bakchine S, Loft H. Memantine treatment in patients with mild to moderate Alzheimer's disease: results of a randomised, double-blind, placebo-controlled 6-month study. *Journal of Alzheimer's disease : JAD* 2008; **13**(1): 97-107.
35. Black SE, Doody R, Li H, et al. Donepezil preserves cognition and global function in patients with severe Alzheimer disease. *Neurology* 2007; **69**(5): 459-69.
36. Blesa González R, Boada Rovira M, Martínez Parra C, Gil-Saladié D, Almagro CA, Gobartt Vázquez AL. Evaluation of the convenience of changing the rivastigmine administration route in patients with Alzheimer disease. *Neurologia (Barcelona, Spain)* 2011; **26**(5): 262-71.
37. Burns A, Bernabei R, Bullock R, et al. Safety and efficacy of galantamine (Reminyl) in severe Alzheimer's disease (the SERAD study): a randomised, placebo-controlled, double-blind trial. *The Lancet Neurology* 2009; **8**(1): 39-47.
38. Burns A, Perry E, Holmes C, et al. A double-blind placebo-controlled randomized trial of Melissa officinalis oil and donepezil for the treatment of agitation in Alzheimer's disease. *Dementia and geriatric cognitive disorders* 2011; **31**(2): 158-64.
39. Burns A, Rossor M, Hecker J, et al. The effects of donepezil in Alzheimer's disease - results from a multinational trial. *Dementia and geriatric cognitive disorders* 1999; **10**(3): 237-44.
40. Choi SH, Park KW, Na DL, et al. Tolerability and efficacy of memantine add-on therapy to rivastigmine transdermal patches in mild to moderate Alzheimer's disease: a multicenter, randomized, open-label, parallel-group study. *Current medical research and opinion* 2011; **27**(7): 1375-83.
41. Corey-Bloom J. A randomized trial evaluating the efficacy and safety of ENA 713 (rivastigmine tartrate), a new acetylcholinesterase inhibitor, in patients with mild to moderately severe Alzheimer's disease. *Int J Geriatr Psyopharmacol* 1998; **1**: 55-65.
42. Creţu O, Szalontay AS, Chiriţă R, Chiriţă V. Effect of memantine treatment on patients with moderate-to-severe Alzheimer's disease treated with donepezil. *Revista medico-chirurgicala a Societatii de Medici si Naturalisti din Iasi* 2008; **112**(3): 641-5.
43. Dysken MW, Sano M, Asthana S, et al. Effect of vitamin E and memantine on functional decline in Alzheimer disease: the TEAM-AD VA cooperative randomized trial. *Jama* 2014; **311**(1): 33-44.
44. Farlow MR, Grossberg GT, Sadowsky CH, Meng X, Somogyi M. A 24-week, randomized, controlled trial of rivastigmine patch 13.3 mg/24 h versus 4.6 mg/24 h in severe Alzheimer's dementia. *CNS neuroscience & therapeutics* 2013; **19**(10): 745-52.
45. Feldman H, Gauthier S, Hecker J, Vellas B, Subbiah P, Whalen E. A 24-week, randomized, double-blind study of donepezil in moderate to severe Alzheimer's disease. *Neurology* 2001; **57**(4): 613-20.
46. Feldman HH, Lane R. Rivastigmine: a placebo controlled trial of twice daily and three times daily regimens in patients with Alzheimer's disease. *Journal of neurology, neurosurgery, and psychiatry* 2007; **78**(10): 1056-63.

47. Fox C, Crugel M, Maidment I, et al. Efficacy of memantine for agitation in Alzheimer's dementia: a randomised double-blind placebo controlled trial. *PloS one* 2012; **7**(5): e35185.

48. Frölich L, Ashwood T, Nilsson J, Eckerwall G. Effects of AZD3480 on cognition in patients with mild-to-moderate Alzheimer's disease: a phase IIb dose-finding study. *Journal of Alzheimer's disease : JAD* 2011; **24**(2): 363-74.

49. Fuschillo C, Ascoli E, Franzese G, et al. Alzheimer's disease and acetylcholinesterase inhibitor agents: a two-year longitudinal study. *Archives of gerontology and geriatrics Supplement* 2004; (9): 187-94.

50. Gault LM, Ritchie CW, Robieson WZ, Pritchett Y, Othman AA, Lenz RA. A phase 2 randomized, controlled trial of the α7 agonist ABT-126 in mild-to-moderate Alzheimer's dementia. *Alzheimer's & dementia (New York, N Y)* 2015; **1**(1): 81-90.

51. Gold M, Alderton C, Zvartau-Hind M, et al. Rosiglitazone monotherapy in mild-to-moderate Alzheimer's disease: results from a randomized, double-blind, placebo-controlled phase III study. *Dementia and geriatric cognitive disorders* 2010; **30**(2): 131-46.

52. Greenberg SM, Tennis MK, Brown LB, et al. Donepezil therapy in clinical practice: a randomized crossover study. *Archives of neurology* 2000; **57**(1): 94-9.

53. Grossberg GT, Manes F, Allegri RF, et al. The safety, tolerability, and efficacy of once-daily memantine (28 mg): a multinational, randomized, double-blind, placebo-controlled trial in patients with moderate-to-severe Alzheimer's disease taking cholinesterase inhibitors. *CNS drugs* 2013; **27**(6): 469-78.

54. Hager K, Baseman AS, Nye JS, et al. Effects of galantamine in a 2-year, randomized, placebo-controlled study in Alzheimer's disease. *Neuropsychiatric disease and treatment* 2014; **10**: 391-401.

55. Haig GM, Pritchett Y, Meier A, et al. A randomized study of H3 antagonist ABT-288 in mild-to-moderate Alzheimer's dementia. *Journal of Alzheimer's disease : JAD* 2014; **42**(3): 959-71.

56. Hernandez C, Unturbe F, Martinez-Lage P, Lucas A, Gregorio P, Alonso T. Effects of combined pharmacologic and cognitive treatment in the progression of moderate dementia: a two-year follow-up. *REVISTA ESPANOLA DE GERIATRIA Y GERONTOLOGIA* 2007; **42**(1): 3.

57. Herrmann N, Gauthier S, Boneva N, Lemming OM. A randomized, double-blind, placebo-controlled trial of memantine in a behaviorally enriched sample of patients with moderate-to-severe Alzheimer's disease. *International psychogeriatrics* 2013; **25**(6): 919-27.

58. Holmes C, Wilkinson D, Dean C, et al. The efficacy of donepezil in the treatment of neuropsychiatric symptoms in Alzheimer disease. *Neurology* 2004; **63**(2): 214-9.

59. Homma A, Imah Y, Hariguchi S. Late phase II clinical study of acetylcholinesterase inhibitor E 2020 in patients with alzheimer-type dementia-12-weeks double-blind, placebo-controlled study 3 mg/day, 5mg/day. *Clinical Evaluation* 1998; **26**: 251-84.

60. Homma A, Imai Y, Tago H, et al. Donepezil treatment of patients with severe Alzheimer's disease in a Japanese population: results from a 24-week, double-blind, placebo-controlled, randomized trial. *Dementia and geriatric cognitive disorders* 2008; **25**(5): 399-407.

61. Hong Z, Zhang Z, Wang L, et al. A randomized study comparing the effect and safety of galantamine and donepezil in patients with mild to moderate Alzheimer's disease. *Chin J Neurol* 2006; **39**(6): 379-82.

62. Howard R, McShane R, Lindesay J, et al. Donepezil and memantine for moderate-to-severe Alzheimer's disease. *New England Journal of Medicine* 2012; **366**(10): 893-903.

63. Howard RJ, Juszczak E, Ballard CG, et al. Donepezil for the treatment of agitation in Alzheimer's disease. *The New England journal of medicine* 2007; **357**(14): 1382-92.

64. Hu HT, Zhang ZX, Yao JL, et al. [Clinical efficacy and safety of akatinol memantine in treatment of mild to moderate Alzheimer disease: a donepezil-controlled, randomized trial]. *Zhonghua nei ke za zhi* 2006; **45**(4): 277-80.

65. Johannsen P, Salmon E, Hampel H, et al. Assessing therapeutic efficacy in a progressive disease: a study of donepezil in Alzheimer's disease. *CNS drugs* 2006; **20**(4): 311-25.

66. Jones RW, Soininen H, Hager K, et al. A multinational, randomised, 12-week study comparing the effects of donepezil and galantamine in patients with mild to moderate Alzheimer's disease. *International journal of geriatric psychiatry* 2004; **19**(1): 58-67.

67. Kadir A, Darreh-Shori T, Almkvist O, et al. PET imaging of the in vivo brain acetylcholinesterase activity and nicotine binding in galantamine-treated patients with AD. *Neurobiology of aging* 2008; **29**(8): 1204-17.
68. Kano O, Ito H, Takazawa T, et al. Clinically meaningful treatment responses after switching to galantamine and with addition of memantine in patients with Alzheimer's disease receiving donepezil. *Neuropsychiatric disease and treatment* 2013; **9**: 259-65.
69. Karaman Y, Erdoğan F, Köseoğlu E, Turan T, Ersoy AO. A 12-month study of the efficacy of rivastigmine in patients with advanced moderate Alzheimer's disease. *Dementia and geriatric cognitive disorders* 2005; **19**(1): 51-6.
70. Likitjaroen Y, Meindl T, Friese U, et al. Longitudinal changes of fractional anisotropy in Alzheimer's disease patients treated with galantamine: a 12-month randomized, placebo-controlled, double-blinded study. *European archives of psychiatry and clinical neuroscience* 2012; **262**(4): 341-50.
71. Lorenzi M, Beltramello A, Mercuri NB, et al. Effect of memantine on resting state default mode network activity in Alzheimer's disease. *Drugs & aging* 2011; **28**(3): 205-17.
72. Maher-Edwards G, Dixon R, Hunter J, et al. SB-742457 and donepezil in Alzheimer disease: a randomized, placebo-controlled study. *International journal of geriatric psychiatry* 2011; **26**(5): 536-44.
73. Marek GJ, Katz DA, Meier A, et al. Efficacy and safety evaluation of HSD-1 inhibitor ABT-384 in Alzheimer's disease. *Alzheimer's & dementia : the journal of the Alzheimer's Association* 2014; **10**(5 Suppl): S364-73.
74. Mazza M, Capuano A, Bria P, Mazza S. Ginkgo biloba and donepezil: a comparison in the treatment of Alzheimer's dementia in a randomized placebo-controlled double-blind study. *European journal of neurology* 2006; **13**(9): 981-5.
75. Mohs RC, Doody RS, Morris JC, et al. A 1-year, placebo-controlled preservation of function survival study of donepezil in AD patients. *Neurology* 2001; **57**(3): 481-8.
76. Moretti DV. Alpha rhythm oscillations and MMSE scores are differently modified by transdermal or oral rivastigmine in patients with Alzheimer's disease. *American journal of neurodegenerative disease* 2014; **3**(2): 72-83.
77. Mowla A, Mosavinasab M, Haghshenas H, Borhani Haghighi A. Does serotonin augmentation have any effect on cognition and activities of daily living in Alzheimer's dementia? A double-blind, placebo-controlled clinical trial. *Journal of clinical psychopharmacology* 2007; **27**(5): 484-7.
78. Nakamura Y, Imai Y, Shigeta M, et al. A 24-week, randomized, double-blind, placebo-controlled study to evaluate the efficacy, safety and tolerability of the rivastigmine patch in Japanese patients with Alzheimer's disease. *Dementia and geriatric cognitive disorders extra* 2011; **1**(1): 163-79.
79. Nakano S, Asada T, Matsuda H, Uno M, Takasaki M. Donepezil hydrochloride preserves regional cerebral blood flow in patients with Alzheimer's disease. *Journal of nuclear medicine : official publication, Society of Nuclear Medicine* 2001; **42**(10): 1441-5.
80. Nordberg A, Darreh-Shori T, Peskind E, et al. Different cholinesterase inhibitor effects on CSF cholinesterases in Alzheimer patients. *Current Alzheimer research* 2009; **6**(1): 4-14.
81. Pakdaman H, Harandi AA, Hatamian H, et al. Effectiveness and Safety of MLC601 in the Treatment of Mild to Moderate Alzheimer's Disease: A Multicenter, Randomized Controlled Trial. *Dementia and geriatric cognitive disorders extra* 2015; **5**(1): 96-106.
82. Peng D, Xianhao X, Wang L. Efficiency and safety assessment of donepezil for treating mild and moderate Alzheimer disease. *Chinese Journal of Tissue Engineering Research* 2005; **9**(13): 170-2.
83. Peskind ER, Potkin SG, Pomara N, et al. Memantine treatment in mild to moderate Alzheimer disease: a 24-week randomized, controlled trial. *The American journal of geriatric psychiatry : official journal of the American Association for Geriatric Psychiatry* 2006; **14**(8): 704-15.
84. Peters O, Fuentes M, Joachim LK, et al. Combined treatment with memantine and galantamine-CR compared with galantamine-CR only in antidementia drug naïve patients with mild-to-moderate Alzheimer's disease. *Alzheimer's & dementia (New York, N Y)* 2015; **1**(3): 198-204.
85. Reisberg B, Doody R, Stöffler A, Schmitt F, Ferris S, Möbius HJ. Memantine in moderate-to-severe Alzheimer's disease. *The New England journal of medicine* 2003; **348**(14): 1333-41.

86. Rockwood K, Fay S, Song X, MacKnight C, Gorman M. Attainment of treatment goals by people with Alzheimer's disease receiving galantamine: a randomized controlled trial. *CMAJ : Canadian Medical Association journal = journal de l'Association medicale canadienne* 2006; **174**(8): 1099-105.
87. Rockwood K, Mintzer J, Truyen L, Wessel T, Wilkinson D. Effects of a flexible galantamine dose in Alzheimer's disease: a randomised, controlled trial. *Journal of neurology, neurosurgery, and psychiatry* 2001; **71**(5): 589-95.
88. Rogers SL, Doody RS, Mohs RC, Friedhoff LT. Donepezil improves cognition and global function in Alzheimer disease: a 15-week, double-blind, placebo-controlled study. Donepezil Study Group. *Archives of internal medicine* 1998; **158**(9): 1021-31.
89. Rogers SL, Farlow MR, Doody RS, Mohs R, Friedhoff LT. A 24-week, double-blind, placebo-controlled trial of donepezil in patients with Alzheimer's disease. Donepezil Study Group. *Neurology* 1998; **50**(1): 136-45.
90. Rogers SL, Friedhoff LT. The efficacy and safety of donepezil in patients with Alzheimer's disease: results of a US Multicentre, Randomized, Double-Blind, Placebo-Controlled Trial. The Donepezil Study Group. *Dementia (Basel, Switzerland)* 1996; **7**(6): 293-303.
91. Saxton J, Hofbauer RK, Woodward M, et al. Memantine and functional communication in Alzheimer's disease: results of a 12-week, international, randomized clinical trial. *Journal of Alzheimer's disease : JAD* 2012; **28**(1): 109-18.
92. Scarpini E, Bruno G, Zappalà G, et al. Cessation versus continuation of galantamine treatment after 12 months of therapy in patients with Alzheimer's disease: a randomized, double blind, placebo controlled withdrawal trial. *Journal of Alzheimer's disease : JAD* 2011; **26**(2): 211-20.
93. Schmidt R, Ropele S, Pendl B, et al. Longitudinal multimodal imaging in mild to moderate Alzheimer disease: a pilot study with memantine. *Journal of neurology, neurosurgery, and psychiatry* 2008; **79**(12): 1312-7.
94. Seltzer B, Zolnouni P, Nunez M, et al. Efficacy of donepezil in early-stage Alzheimer disease: a randomized placebo-controlled trial. *Archives of neurology* 2004; **61**(12): 1852-6.
95. Shao ZQ. Comparison of the efficacy of four cholinesterase inhibitors in combination with memantine for the treatment of Alzheimer's disease. *International journal of clinical and experimental medicine* 2015; **8**(2): 2944-8.
96. Shimizu S, Kanetaka H, Hirose D, Sakurai H, Hanyu H. Differential effects of acetylcholinesterase inhibitors on clinical responses and cerebral blood flow changes in patients with Alzheimer's disease: a 12-month, randomized, and open-label trial. *Dementia and geriatric cognitive disorders extra* 2015; **5**(1): 135-46.
97. Solé-Padullés C, Bartrés-Faz D, Lladó A, et al. Donepezil treatment stabilizes functional connectivity during resting state and brain activity during memory encoding in Alzheimer's disease. *Journal of clinical psychopharmacology* 2013; **33**(2): 199-205.
98. Tariot PN, Cummings JL, Katz IR, et al. A randomized, double-blind, placebo-controlled study of the efficacy and safety of donepezil in patients with Alzheimer's disease in the nursing home setting. *Journal of the American Geriatrics Society* 2001; **49**(12): 1590-9.
99. Tariot PN, Solomon PR, Morris JC, Kershaw P, Lilienfeld S, Ding C. A 5-month, randomized, placebo-controlled trial of galantamine in AD. The Galantamine USA-10 Study Group. *Neurology* 2000; **54**(12): 2269-76.
100. Thomas A, Iacono D, Bonanni L, D'Andreamatteo G, Onofrj M. Donepezil, rivastigmine, and vitamin E in Alzheimer disease: a combined P300 event-related potentials/neuropsychologic evaluation over 6 months. *Clinical neuropharmacology* 2001; **24**(1): 31-42.
101. Wilcock G, Howe I, Coles H, et al. A long-term comparison of galantamine and donepezil in the treatment of Alzheimer's disease. *Drugs & aging* 2003; **20**(10): 777-89.
102. Wilkinson D. A Clinical Study Evaluating the Effects of Memantine on Brain Atrophy in Patients With Alzheimer's Disease; 2012.
103. Wilkinson D, Murray J. Galantamine: a randomized, double-blind, dose comparison in patients with Alzheimer's disease. *International journal of geriatric psychiatry* 2001; **16**(9): 852-7.

104. Wilkinson DG, Passmore AP, Bullock R, et al. A multinational, randomised, 12-week, comparative study of donepezil and rivastigmine in patients with mild to moderate Alzheimer's disease. *International journal of clinical practice* 2002; **56**(6): 441-6.
105. Winblad B, Cummings J, Andreasen N, et al. A six-month double-blind, randomized, placebo-controlled study of a transdermal patch in Alzheimer's disease--rivastigmine patch versus capsule. *International journal of geriatric psychiatry* 2007; **22**(5): 456-67.
106. Winblad B, Engedal K, Soininen H, et al. A 1-year, randomized, placebo-controlled study of donepezil in patients with mild to moderate AD. *Neurology* 2001; **57**(3): 489-95.
107. Winblad B, Kilander L, Eriksson S, et al. Donepezil in patients with severe Alzheimer's disease: double-blind, parallel-group, placebo-controlled study. *Lancet (London, England)* 2006; **367**(9516): 1057-65.
108. Zhang Y, Rong-jia Y, Ju-ying G, H. G, Chao-mei W. Effects of aricept on the treatment of Alzheimer disease evaluated by skull multi-slice helical CT. *Chin J Clin Rehab* 2005; **9**(25): 20-1.
109. Zhang Z, Yu L, Gaudig M, Schäuble B, Richarz U. Galantamine versus donepezil in Chinese patients with Alzheimer's disease: results from a randomized, double-blind study. *Neuropsychiatric disease and treatment* 2012; **8**: 571-7.

16 Companion Reports

110. Aronson S, Van Baelen B, Kavanagh S, Schwalen S. Optimal dosing of galantamine in patients with mild or moderate Alzheimer's disease: post Hoc analysis of a randomized, double-blind, placebo-controlled trial. *Drugs & aging* 2009; 26(3): 231-9.
111. Cummings JL, Schneider L, Tariot PN, Kershaw PR, Yuan W. Reduction of behavioral disturbances and caregiver distress by galantamine in patients with Alzheimer's disease. *The American journal of psychiatry* 2004; 161(3): 532-8.
112. Feldman H, Gauthier S, Hecker J, et al. Economic evaluation of donepezil in moderate to severe Alzheimer disease. *Neurology* 2004; 63(4): 644-50.
113. Feldman H, Gauthier S, Hecker J, et al. Efficacy and safety of donepezil in patients with more severe Alzheimer's disease: a subgroup analysis from a randomized, placebo-controlled trial. *International journal of geriatric psychiatry* 2005; 20(6): 559-69.
114. Gaudig M, Richarz U, Han J, Van Baelen B, Schäuble B. Effects of galantamine in Alzheimer's disease: double-blind withdrawal studies evaluating sustained versus interrupted treatment. *Current Alzheimer research* 2011; 8(7): 771-80.
115. Gauthier S, Feldman H, Hecker J, Vellas B, Emir B, Subbiah P. Functional, cognitive and behavioral effects of donepezil in patients with moderate Alzheimer's disease. *Current medical research and opinion* 2002; 18(6): 347-54.
116. Grossberg GT, Farlow MR, Meng X, Velting DM. Evaluating high-dose rivastigmine patch in severe Alzheimer's disease: analyses with concomitant memantine usage as a factor. *Current Alzheimer research* 2015; 12(1): 53-60.
117. Han HJ, Kim BC, Lee JY, et al. Response to rivastigmine transdermal patch or memantine plus rivastigmine patch is affected by apolipoprotein E genotype in Alzheimer patients. *Dementia and geriatric cognitive disorders* 2012; 34(3-4): 167-73.
118. Jelic V, Haglund A, Kowalski J, Langworth S, Winblad B. Donepezil treatment of severe Alzheimer's disease in nursing home settings. A responder analysis. *Dementia and geriatric cognitive disorders* 2008; 26(5): 458-66.
119. Kumar V, Anand R, Messina J, Hartman R, Veach J. An efficacy and safety analysis of Exelon in Alzheimer's disease patients with concurrent vascular risk factors. *European journal of neurology* 2000; 7(2): 159-69.
120. Ott BR, Blake LM, Kagan E, Resnick M. Open label, multicenter, 28-week extension study of the safety and tolerability of memantine in patients with mild to moderate Alzheimer's disease. *Journal of neurology* 2007; 254(3): 351-8.

121. Reisberg B, Doody R, Stöffler A, Schmitt F, Ferris S, Möbius HJ. A 24-week open-label extension study of memantine in moderate to severe Alzheimer disease. *Archives of neurology* 2006; 63(1): 49-54.

122. Schwam E, Xu Y. Cognition and function in Alzheimer's disease: identifying the transitions from moderate to severe disease. *Dementia and geriatric cognitive disorders* 2010; 29(4): 309-16.

123. van Dyck CH, Tariot PN, Meyers B, Malca Resnick E. A 24-week randomized, controlled trial of memantine in patients with moderate-to-severe Alzheimer disease. *Alzheimer disease and associated disorders* 2007; 21(2): 136-43.

124. Wimo A, Winblad B, Engedal K, et al. An economic evaluation of donepezil in mild to moderate Alzheimer's disease: results of a 1-year, double-blind, randomized trial. *Dementia and geriatric cognitive disorders* 2003; 15(1): 44-54.

125. Winblad B, Grossberg G, Frölich L, et al. IDEAL: a 6-month, double-blind, placebo-controlled study of the first skin patch for Alzheimer disease. *Neurology* 2007; 69(4 Suppl 1): S14-22.

Appendix 3: Studies with available IPD but insufficient data to be included in the analysis

A study[1] of 859 participants comparing transdermal rivastigmine vs. placebo included only IPD for the placebo arm. Another study[2] of 285 participants comparing 22·5 mg of galantamine vs. 30 mg of galantamine vs. 45 mg of galantamine vs. placebo did not provide information about the AE or MMSE outcomes in the shared IPD.

CSDR: Novartis (study: NVT_SA_ENA713D1301) – Nakamura 2011

The study compares rivastigmine patch vs. placebo, but includes data only on placebo. Hence, we cannot conduct an analysis to convert data on their aggregated form so that to be included in our network meta-analysis. The IPD of this study included 288 participants in total.
According to the publication, 284 were allocated to the rivastigmine patch 5 cm2 group, 287 to the rivastigmine patch 10 cm2 group, and 288 to the placebo group.

Baseline characteristics of included patients

Characteristics	PLAC	Total	Missing Data	P-value	Outliers
Males	92 (32 %)	92 (32 %)	No	-	No
Age, mean (SD)	74.6 (7.4)	74.6 (7.4)	No	-	Yes - 1 value
AE, events/sample size	19/288	19/288	No	-	-
Baseline MMSE, mean (SD)	16.6 (2.9)	16.6 (2.9)	Yes - 1 value	-	No
MMSE, mean (SD)	17.5 (3.4)	17.5 (3.4)	No	-	No
Change score, mean (SD)	0.9 (1.6)	0.9 (1.6)	Yes - 2 values	-	Yes - 41 values
Total number of patients	288 (100 %)	288			

YODA: JNJ-Study-GAL-93-01 –Wilkinson 2001

The study compares galantamine 22.5mg, 30mg and 45mg vs placebo. In our analysis we combined galantamine 22.5mg, 30mg and 45mg in a single group. However, we only descriptively can include this study in our paper - not in the network meta-analysis – as it does not provide any info about the AE or MMSE outcomes (only total score for baseline). The IPD of this study included 285 participants in total.
According to the publication, 285 patients were randomized to: galantamine 18mg, 24mg, 36mg/day and placebo. Of the outcomes of interest, publication reported the AE outcome. According to the sponsor there are no differences in the reporting of doses:

- galantamine hydrobromide 7.5 mg =6 mg galantamine base was administered tid i.e galantamine hydrobromide 22.5 mg/d = galantamine base **18mg/day**
- galantamine hydrobromide 10 mg =8 mg galantamine base was administered tid i.e galantamine hydrobromide 30mg/d= galantamine base **24mg/day** and
- galantamine hydrobromide 15 mg =12 mg galantamine base was administered tid i.e galantamine hydrobromide 45mg/d= galantamine base **36mg/day**

Baseline characteristics of included patients

Characteristics	GALA	PLAC	Total	Missing Data	P-value	Outliers
Males	85 (30%)	36 (12%)	121 (42%)	No	<0.001	No
Age, mean (SD)	73.5 (8.2)	74.2 (9.0)	73.8 (8.5)	No	0.242	Yes - 1 value
AE, events/sample size*	-	-	-	-	-	-
Baseline MMSE, mean (SD)	18.6 (3.2)	18.8 (3.1)	18.7 (3.2)	No	0.616	No
MMSE, mean (SD)	-	-	-	-	-	-
Change score, mean (SD)	-	-	-	-	-	-
Total number of patients	198 (69%)	87 (31%)	285 (100%)			

*AE in publication is as follows, PLAC: 3/87, GALA 18mg: 6/88, GALA 24mg: 0/56, GALA 36mg: 5/54

[1] Nakamura Y, Imai Y, Shigeta M, et al. A 24-week, randomized, double-blind, placebo-controlled study to evaluate the efficacy, safety and tolerability of the rivastigmine patch in Japanese patients with Alzheimer's disease. Dement Geriatr Cogn Dis Extra 2011; 1(1): 163-79.
[2] Wilkinson D, Murray J. Galantamine: a randomized, double-blind, dose comparison in patients with Alzheimer's disease. Int J Geriatr Psychiatry 2001; 16(9): 852-7.

Appendix 4: List of studies requested and sponsor response

Sponsor	Author, year	Interventions compared (dosage mg)*	Sponsor Response	IPD Received
Abbvie	Gault, 2015	Placebo/No treatment, Donepezil (10 mg)	Available	Yes
	Haig, 2014	Placebo/No treatment, Donepezil (5 – 10 mg)	Available	Yes
	Marek, 2014	Placebo/No treatment, Donepezil (5 – 10 mg)	Unavailable (Cannot share data (Potential business considerations under review))	No
AstraZeneca	Frolich, 2011	Placebo/No treatment, Donepezil (5 – 10 mg)	Available	No
Daiichi-Sankyo	Shimizu, 2015	Donepezil (5 mg), Galantamine (24 mg), Rivastigmine (18 mg)	Unavailable (Do not own data)	No
Eisai	Black, 2007	Placebo/No treatment, Donepezil (5 – 10 mg)	Available	Yes
	Burns, 1999	Placebo/No treatment, Donepezil (5 – 10 mg)	Unavailable (Cannot share data (Old study))	No
	Feldman, 2001	Placebo/No treatment, Donepezil (5 – 10 mg)	Unavailable (Do not own data)	No
	Feldman, 2004	Placebo/No treatment, Donepezil (5 – 10 mg)	Unavailable (Do not own data)	No
	Feldman, 2005	Placebo/No treatment, Donepezil (5 – 10 mg)	Unavailable (Do not own data)	No
	Gauthier, 2002	Placebo/No treatment, Donepezil (5 – 10 mg)	Unavailable (Do not own data)	No
	Holmes, 2004	Placebo/No treatment, Donepezil (10 mg)	Unavailable (Do not own data)	No
	Homma, 2008	Placebo/No treatment, Donepezil (10 mg)	Unavailable (Cannot share data (Old study))	No
	Johannsen, 2006	Placebo/No treatment, Donepezil (10 mg)	Unavailable (Do not own data)	No
	Jones, 2004	Donepezil (5 – 10 mg), Galantamine (8 – 24 mg)	Unavailable (Cannot share data (Old study))	No
	Mohs, 2001	Placebo/No treatment, Donepezil (5 – 10 mg)	Unavailable (Cannot share data (Old study))	No
	Rogers, 1996	Placebo/No treatment, Donepezil (5 mg)	Unavailable (Cannot share data (Old study))	No
	Rogers, 1998	Placebo/No treatment, Donepezil (10 mg)	Unavailable (Cannot share data (Old study))	No
	Rogers, 1998	Placebo/No treatment, Donepezil (10 mg)	Unavailable (Cannot share data (Old study))	No
	Schwam, 2010	Placebo/No treatment, Donepezil (5 – 10 mg)	Unavailable (Do not own data)	No
	Seltzer, 2004	Donepezil (5 – 10 mg), Placebo/No treatment	Unavailable (Cannot share data (Old study))	No
	Shimizu, 2015	Donepezil (5 mg), Galantamine (24 mg), Rivastigmine (18 mg)	Unavailable (Do not own data)	No
	Sole-Padulles, 2013	Placebo/No treatment, Donepezil (5 – 10 mg)	Unavailable (Do not own data)	No
	Tariot, 2001	Placebo/No treatment, Donepezil (5 – 10 mg)	Unavailable (Cannot share data (Old study))	No
	Wilkinson, 2002	Donepezil (5 – 10 mg), Rivastigmine (6 – 12 mg)	Unavailable (Do not own data)	No
Forest Laboratories/Allergen	Grossberg, 2013	Donepezil (NR) + Rivastigmine (13.3 mg) + Galantamine + Placebo, Donepezil (NR) + Rivastigmine (4.6 mg) + Galantamine (NR)+ Memantine (NR)	Unavailable (Cannot share data (No details provided))	No
	Ott, 2007	Placebo/No treatment, Memantine (5 -20 mg)	Unavailable (Cannot share data (No details provided))	No
	Peskind, 2006	Placebo/No treatment, Memantine (5 -20 mg)	Unavailable (Cannot share data (No details provided))	No
	Saxton, 2012	Placebo/No treatment, Memantine (20 mg)	Unavailable (Cannot share data (No details provided))	No
	van Dyck, 2007	Placebo/No treatment, Memantine (20 mg)	Unavailable (Cannot share data (No details provided))	No
GlaxoSmithKline	Gold, 2010	Placebo/No treatment, Donepezil (10 mg)	Available	Yes
	Maher-Edwards, 2011	Placebo/No treatment, Donepezil (5 – 10 mg)	Unavailable (Do not own data)	No
Janssen	Ancoli-Israel, 2005	Donepezil (10 mg), Galantamine (8 mg)	Unavailable (Cannot identify study)	No
	Aronson, 2009	Placebo/No treatment, Galantamine (16 – 24 mg)	Unavailable (Cannot identify study)	No
	Burns, 2009	Placebo/No treatment, Galantamine (8-24 mg)	Available	Yes
	Cummings, 2004	Placebo/No treatment, Galantamine (4, 8, 12 mg)	Available	Yes
	Gaudig, 2011	Placebo/No treatment, Galantamine (8 mg)	Unavailable (Cannot identify study)	No
	Hager K, 2014	Placebo/No treatment, Galantamine (8 – 24 mg)	Available	Yes
	Kadir, 2008	Placebo/No treatment, Galantamine (16 – 24 mg)	Unavailable (Cannot identify study)	No
	Likitjaroen, 2012	Placebo/No treatment, Galantamine (8 – 24 mg)	Unavailable(Do not own data)	No
	Rockwood, 2001	Placebo/No treatment, Galantamine (24, 32 mg)	Available	Yes
	Rockwood, 2006	Placebo/No treatment, Galantamine (16 – 24 mg)	Unavailable (IPD not available)	No
	Scarpini, 2011	Placebo/No treatment, Galantamine (16 mg)	Unavailable (IPD not available)	No

Sponsor	Author, year	Interventions compared (dosage mg)*	Sponsor Response	IPD Received
	Shimizu, 2015	Donepezil (5 mg), Galantamine (24 mg), Rivastigmine (18 mg)	Unavailable (Cannot identify study)	No
	Tariot, 2000	Placebo/No treatment, Galantamine (8 mg)	Unavailable (Cannot identify study)	No
	Wilcock, 2003	Donepezil (5 – 10 mg), Galantamine (16 – 24 mg)	Unavailable (Cannot identify study)	No
	Zhang, 2012	Donepezil (5 – 10 mg), Galantamine (6 – 16 mg or 6 – 24 mg)	Unavailable (IPD not available)	No
	Wilkinson, 2001	Placebo/No treatment, Galantamine (18 - 36 mg)	Available	Yes
Lundbeck	Bakchine, 2008	Placebo/No treatment, Memantine (20 mg)	Available	Yes
	Fox, 2012	Placebo/No treatment, Memantine (5 – 20 mg)	Unavailable (Do not own data)	No
	Herrmann, 2013	Placebo/No treatment, Memantine (5 – 20 mg)	Available	Yes
	Lorenzi, 2011	Placebo/No treatment, Memantine (5 – 20 mg)	Unavailable (Do not own data)	No
	Wilkinson, 2012	Placebo/No treatment, Memantine (5 – 20 mg)	Available	Yes
Merz	Reisberg, 2003	Placebo/No treatment, Memantine (20 mg)	No response from sponsor	No
	Reisberg, 2006	Placebo/No treatment, Memantine (20 mg)	No response from sponsor	No
	Schmidt, 2008	Placebo/No treatment, Memantine (5 – 20 mg)	No response from sponsor	No
	Winblad, 2007	Placebo/No treatment, Rivastigmine (3 – 12 mg)	No response from sponsor	No
Novartis	Agid, 1998	Placebo/No treatment, Rivastigmine (6 mg)	Unavailable (Cannot identify study)	No
	Blesa González, 2011	Placebo/No treatment, Rivastigmine (6 – 12 mg)	Unavailable (Cannot share data)	No
	Choi, 2011	Placebo/No treatment, Memantine (5 – 20 mg)	Unavailable (Do not own data)	No
	Corey-Bloom, 1998	Placebo/No treatment, Rivastigmine (6 – 12 mg)	Unavailable (Cannot identify study)	No
	Farlow, 2013	Rivastigmine (4.6 - 13.3 mg), Rivastigmine (4.6 mg) + Memantine (20 mg)	Unavailable (Cannot share data (Phase 4 study))	No
	Feldman, 2007	Placebo/No treatment, Rivastigmine (2 – 12 mg)	Unavailable (Cannot identify study)	No
	Grossberg, 2015	Rivastigmine (4.6 - 13.3 mg), Rivastigmine (4.6 mg) + Memantine (20 mg)	Unavailable (Cannot share data (Phase 4 study))	No
	Han, 2012	Placebo/No treatment, Memantine (5 – 20 mg)	Unavailable (Cannot identify study)	No
	Kumar, 2000	Placebo/No treatment, Rivastigmine (1 – 12 mg)	Unavailable (Cannot identify study)	No
	Nakamura, 2011	Placebo/No treatment, Rivastigmine (4.5 – 9.5 mg)	Available	Yes
	Nordberg, 2009	Donepezil (5 – 10 mg), Galantamine (8 – 24 mg), Rivastigmine (3 – 12 mg)	Unavailable (Cannot share data (Phase 4 study))	No
	Shimizu, 2015	Donepezil (5 mg), Galantamine (24 mg), Rivastigmine (18 mg)	Unavailable (Cannot identify study)	No
	Winblad, 2007	Placebo/No treatment, Rivastigmine (3 – 12 mg)	Available	Yes
ONO	Nakamura, 2011	Placebo/No treatment, Rivastigmine (4.5 – 9.5 mg)	No response from sponsor	No
Pfizer	Black, 2007	Placebo/No treatment, Donepezil (10 mg)	Unavailable (Do not own data)	No
	Feldman, 2001	Placebo/No treatment, Donepezil (5 – 10 mg)	Available	No
	Feldman, 2004	Placebo/No treatment, Donepezil (5 – 10 mg)	Unavailable (Cannot identify study)	No
	Feldman, 2005	Placebo/No treatment, Donepezil (5 – 10 mg)	Unavailable (Cannot identify study)	No
	Gauthier, 2002	Placebo/No treatment, Donepezil (5 – 10 mg)	Unavailable (Cannot identify study)	No
	Holmes, 2004	Placebo/No treatment, Donepezil (10 mg)	Unavailable (Cannot identify study)	No
	Jelic, 2008	Placebo/No treatment, Donepezil (5 – 10 mg)	Unavailable (Cannot identify study)	No
	Johannsen, 2006	Placebo/No treatment, Donepezil (10 mg)	Unavailable (Cannot identify study)	No
	Jones, 2004	Donepezil, Galantamine (8 – 24 mg)	Unavailable (Cannot identify study)	No
	Mohs, 2001	Placebo/No treatment, Donepezil (5 – 10 mg)	Unavailable (Cannot identify study)	No
	Schwam, 2010	Placebo/No treatment, Donepezil (5 – 10 mg)	Unavailable (Cannot identify study)	No
	Seltzer, 2004	Donepezil (5 – 10 mg), Placebo/No treatment	Unavailable (Cannot identify study)	No
	Sole-Padulles, 2013	Placebo/No treatment, Donepezil (5 – 10 mg)	Unavailable (Cannot identify study)	No
	Tariot, 2001	Placebo/No treatment, Donepezil (5 – 10 mg)	Unavailable (Cannot identify study)	No

Veroniki AA, *et al. BMJ Open* 2022; 12:e053012. doi: 10.1136/bmjopen-2021-053012

Sponsor	Author, year	Interventions compared (dosage mg)*	Sponsor Response	IPD Received
	Wilkinson, 2002	Donepezil (5 – 10 mg), Rivastigmine (6 – 12 mg)	Unavailable (Cannot identify study)	No
	Wimo, 2003	Placebo/No treatment, Donepezil (5 – 10 mg)	Unavailable (Cannot identify study)	No
	Winblad, 2001	Placebo/No treatment, Donepezil (5 – 10 mg)	Unavailable (Cannot identify study)	No
	Winblad, 2006	Placebo/No treatment, Donepezil (5 – 10 mg)	Unavailable (Cannot identify study)	No
Roivant	Maher-Edwards, 2011	Placebo/No treatment, Donepezil (5 – 10 mg)	No response from sponsor	No
Shire Pharmaceuticals	Wilcock, 2003	Donepezil (5 – 10 mg), Galantamine (16 – 24 mg)	Unavailable (Do not own data)	No
	Wilkinson, 2001	Placebo/No treatment, Galantamine (24 mg)	Unavailable (Do not own data)	No
Takeda	Shimizu, 2015	Donepezil (5 mg), Galantamine (24 mg), Rivastigmine (18 mg)	Unavailable (Do not own data)	No
Non-Pharmaceutical	Andersen, 2012	Placebo/No treatment, Donepezil (5 – 10 mg)	NA	No
	Araki, 2014	Placebo/No treatment, Donepezil (NR) + Memantine (5 – 20 mg)	NA	No
	Burns, 2011	Placebo/No treatment, Donepezil (5 – 10 mg)	NA	No
	Dysken, 2014	Placebo/No treatment, Memantine (20 mg)	Available	No
	Greenberg, 2000	Placebo/No treatment, Donepezil (5 mg)	Unavailable (Need to contact PI)	No
	Howard, 2007	Placebo/No treatment, Donepezil (5 – 10 mg)	Unavailable (Do not own data)	No
	Howard, 2012	Donepezil (10 mg) + Memantine (5 – 20 mg), Donepezil (10 mg) + Placebo	Unavailable (Do not own data)	No
	Mowla, 2007	Placebo/No treatment, Rivastigmine (3 – 12 mg)	NA	No
	Peters, 2015	Galantamine (24 mg) + Placebo, Galantamine (24 mg) + Memantine (20 mg)	NA	No
Not reported	Cretu, 2008	Placebo/No treatment, Memantine (5 – 20 mg)	NA	No
	Fuschillo, 2001	Donepezil (5 mg), Rivastigmine (6 – 9 mg)	NA	No
	Hernández, 2007	Placebo/No treatment, Donepezil (10 mg)	NA	No
	Homma, 1998	Donepezil (3 – 5 mg), Placebo/no treatment	NA	No
	Hong, 2006	Placebo/No treatment, Galantamine (8 – 24 mg)	NA	No
	Hu, 2006	Donepezil (5 mg), Memantine (5 – 10 mg)	NA	No
	Kano, 2013	Donepezil(10 mg), Donepezil (10 mg) + Memantine (20 mg)	NA	No
	Karaman, 2005	Placebo/No treatment, Rivastigmine (3 – 12 mg)	NA	No
	Mazza, 2006	Placebo/No treatment, Donepezil (5 mg)	NA	No
	Moretti, 2014	Placebo/No treatment, Rivastigmine (3 – 12 mg)	NA	No
	Nakano, 2001	Placebo/No treatment, Donepezil (5 mg)	NA	No
	Pakdaman H, 2015	Donepezil (NR), Galantamine (NR), Rivastigmine (NR)	NA	No
	Peng, 2005	Placebo/No treatment, Donepezil (5 mg)	NA	No
	Shao, 2015	Memantine (5 – 10 mg)+ Placebo, Rivastigmine (1.5 – 3 mg) + Memantine (5 – 10 mg), Donepezil (5 – 10 mg) + Memantine (5 – 10 mg), Galantamine (2 – 6 mg) + Memantine (5 – 10 mg)	NA	No
	Thomas, 2001	Donepezil (5 – 10 mg), Rivastigmine (6 – 12 mg)	NA	No
	Zhang-Yi, 2005	Placebo/No treatment, Donepezil (5 mg)	NA	No

Abbreviations: NA, not applicable; NPH, neutral protamine Hagedorn; NR, not reported; PI, principal investigator

* In studies that examined different dosages of the same intervention, we selected the dosages that were consistent with those approved for use in Canada.

Appendix 5: Study characteristics of the included RCTs

Study	Country of conduct	Sample size; Longest duration of follow-up (weeks)	Treatments compared; Outcomes	Funding information	Date of randomization; Date trial opened; Randomization ratio	IPD available; Reasons for not providing IPD by the data providers
Agid, 1998	12 countries - Austria, Belgium, Czechoslovakia, Denmark, Finland, France, Germany, Ireland, Norway, Sweden, Switzerland, and the UK	402; 13	Rivastigmine, Placebo/No treatment; MMSE, Nausea, Vomiting, Diarrhea, AEs, Headaches	Industry-sponsored	Not reported; Not reported; Not reported	No; Cannot identify study
Ancoli-Israel, 2005	USA	63; 8	Galantamine, Donepezil; CIBIC-plus, Mortality, Nausea, Diarrhea, AEs, Headaches	Industry-sponsored	Not reported; Not reported; Not reported	No; Cannot identify study
Andersen, 2012	Norway	180; 52	Donepezil, Placebo; MMSE, ADAS-cog	Publicly-sponsored	Not reported; June 2003; Not reported	No; NA
Araki, 2014	Japan	37; 24	Donepezil + Memantine, Placebo; MMSE, NPI	Publicly-sponsored	Not reported; Not reported; Not reported	No; NA
Bakchine, 2008	12 countries -Austria, Belgium, Denmark, Finland, France, Greece, Lithuania, the Netherlands, Poland, Spain, Sweden and UK	470; 24	Memantine, Placebo/no treatment; ADAS-cog, ADCS-ADL, NPI, CIBIC-plus, Mortality, AEs, Headaches, Falls	Industry-sponsored	Not reported; Not reported; Not reported	Yes; NA
Black, 2007	5 countries - USA, Canada, France, UK, Australia	343; 24	Donepezil, Placebo/No treatment; MMSE, ADCS-ADL, NPI, CIBIC-plus, Nausea, Vomiting, Diarrhea, AEs	Industry-sponsored	Not reported; January 2001; Not reported	Yes; Do not own data
Blesa González, 2011	Spain	139; 12	Rivastigmine Patch, Rivastigmine Oral; MMSE, Nausea, Vomiting, Diarrhea	Industry-sponsored	Not reported; Not reported; Not reported	No; Cannot share data (Phase 4 study)
Burns, 1999	Australia, Belgium, Canada, France, Germany,Ireland, New Zealand, South Africa and the UK	818; 30	Donepezil, Placebo/no treatment; ADAS-cog, CIBIC-plus, Mortality, Diarrhea, Nausea, AEs, Vomiting	Industry-sponsored	Not reported; Not reported; Not reported	No; Cannot share data (Old study)
Burns, 2009	Belgium, Finland, France, Italy, Norway, Netherlands, Spain, Sweden, Switzerland, UK	407; 26	Galantamine, Placebo/no treatment; Mortality, Nausea, Vomiting, Diarrhea, AEs, Headaches, Falls	Industry-sponsored	Not reported; December 2003; Not reported	Yes; NA
Burns, 2011	UK	62; 12	Donepezil, Placebo/no treatment; NPI, AEs	Publicly-sponsored	Not reported; January 2006; Not reported	No; NA
Choi, 2011	South Korea	171; 16	Memantine, Placebo/No treatment; MMSE, ADAS-cog, ADCS-ADL, NPI, AEs, Nausea, Diarrhea, Vomiting, Headaches	Publicly-sponsored + Industry-sponsored	Not reported; December 2008; Not reported	No; Do not own data
Corey-Bloom, 1998	USA	699; 26	Rivastigmine, Placebo/No treatment; MMSE, ADAS-cog, CIBIC-plus, Mortality, Nausea, Vomiting	Industry-sponsored	Not reported; Not reported; Not reported	No; Cannot identify study

Cretu, 2008	Romania	43;<br>24	Memantine, Placebo/No treatment;<br>MMSE, ADAS-cog, NPI	NA	Not reported;<br>Not reported;<br>Not reported	No;<br>NR
Dysken, 2014	USA	307;<br>26-208	Memantine, Placebo;<br>MMSE, ADAS-cog, ADCS-ADL, NPI, Mortality, AEs	Publicly-sponsored	Not reported;<br>August 2007;<br>1:1:1:1	No;<br>NA
Farlow, 2013	USA	716;<br>24	Rivastigmine + Memantine, Rivastigmine;<br>NPI, Mortality, Falls, Vomiting, Diarrhea, Nausea	Industry-sponsored	Not reported;<br>July 2009;<br>1:1	No;<br>Cannot share data (Phase 4 study)
Feldman, 2001	Canada, Australia, France	290;<br>24	Donepezil, Placebo/No treatment;<br>MMSE, NPI, CIBIC-plus, Mortality, Vomiting, Nausea, Diarrhea, AEs, Headaches	Industry-sponsored	Not reported;<br>Not reported;<br>"50/50 split"	No;<br>NA
Feldman, 2007	Australia, Canada, Ireland, Italy, South Africa, UK	450;<br>26	Rivastigmine, Placebo/No treatment;<br>MMSE, ADAS-cog, CIBIC-plus, AEs, Bradycardia, Headaches, Vomiting, Diarrhea, Nausea	Industry-sponsored	Not reported;<br>Not reported;<br>1:1:1	No;<br>Cannot identify study
Fox, 2012	UK	149;<br>12	Memantine, Placebo;<br>MMSE, NPI, Mortality	Industry-sponsored	Not reported;<br>September 2007;<br>"assigned with equal probability"	No;<br>Unavailable (Do not own data)
Frolich, 2011	Austria, Belgium, Bulgaria, Czech Republic, Germany, Romania, Russia, Spain, UK, Canada	324;<br>12	Donepezil, Placebo/No treatment;<br>MMSE, ADAS-cog, Nausea, Vomiting, Diarrhea, Headaches	Industry-sponsored	Not reported;<br>July 2007;<br>Not reported	No;<br>Available
Fuschillo, 2001	Italy	27;<br>30	Donepezil, Rivastigmine;<br>MMSE, ADAS-cog, Headaches, Vomiting, Diarrhea, Nausea	NA	Not reported;<br>Not reported;<br>Not reported	No;<br>NR
Gault, 2015	USA, Bulgaria, Czech Republic, Slovakia, UK, South Africa	136;<br>14	Donepezil, Placebo;<br>MMSE, ADAS-cog, ADCS-ADL, NPI, CIBIC-plus, Mortality, AEs, Bradycardia, Falls, Headaches, Vomiting, Diarrhea, Nausea	Industry-sponsored	Not reported;<br>October 2009;<br>Not reported	Yes;<br>Available
Gold, 2010	Austria, Bulgaria, Chile, China, Croatia, Estonia, Germany, Greece, Hungary, Mexico, New Zealand, Pakistan, Peru, Republic of the Philippines, Puerto Rico, Republic of Korea, Russian Federation, UK and USA	248;<br>24	Donepezil, Placebo/no treatment;<br>ADAS-cog, CIBIC-plus, Mortality, Headaches, Nausea, Diarrhea, AEs	Industry-sponsored	Not reported;<br>February 2007;<br>2:2:2:1	Yes;<br>Available
Greenberg, 2000	USA	103;<br>24	Donepezil, Placebo/no treatment;<br>ADAS-cog, AEs, Diarrhea, Nausea	Publicly-sponsored	Not reported;<br>Not reported;<br>Not reported	No;<br>Contact PI
Grossberg, 2013	Argentina, USA, Mexico, Chile	676;<br>24	Donepezil + Rivastigmine + Galantamine + Memantine, Donepezil + Rivastigmine + Galantamine + Placebo;<br>NPI, CIBIC-plus, Mortality, Falls,	Industry-sponsored	Not reported;<br>June 2005;<br>1:1	No;<br>Cannot share dat

			Headaches, Vomiting, Diarrhea, Nausea, AEs			
Hager K, 2014	Czech Republic, Estonia, France, Germany, Greece, Italy, Latvia, Lithuania, Romania, Russia, Slovakia, Slovenia, Ukraine	2045; 104	Galantamine, Placebo; MMSE, Mortality, Headaches, Vomiting, Diarrhea, Nausea, AEs	Industry-sponsored	Not reported; May 2008; 1:1	Yes; NA
Haig, 2014	Russia, Ukraine	123; 12	Donepezil, Placebo; MMSE, ADAS-cog, ADCS-ADL, NPI, Headaches, Nausea, AEs	Industry-sponsored	Not reported; Not reported; 1:1:1	Yes; NA
Hernández, 2007	Spain	20; 48	Donepezil, Placebo/No treatment; MMSE, ADAS-cog	NA	Not reported; Not reported; Not reported	No; NR
Herrmann, 2013	Canada	369; 24	Memantine, Placebo; NPI, Mortality, Falls, Nausea, AEs	Industry-sponsored	Not reported; December 2003; "equally allocated"	Yes; NA
Holmes, 2004	UK	96; 24	Donepezil, Placebo/No treatment; MMSE, NPI	Industry-sponsored	Not reported; Not reported; 3:2	No; Cannot identify study
Homma, 1998	Japan	187; 12	Donepezil, Placebo/no treatment; ADAS-cog, Mortality, AEs, Headaches	NA	Not reported; Not reported; Not reported	No; NR
Homma, 2008	Japan	267; 24	Donepezil, Placebo/no treatment; ADCS-ADL, CIBIC-plus, Mortality, AEs, Falls, Vomiting, Diarrhea	Industry-sponsored	Not reported; Not reported; 1:1:1	No; Cannot share data (Old study)
Hong, 2006	China	218; 16	Galantamine, Placebo/no treatment; ADAS-cog, ADCS-ADL, NPI, AEs	NA	Not reported; Not reported; Not reported	No; NR
Howard, 2007	England	259; 12	Donepezil, Placebo/No treatment; MMSE, NPI, Mortality, Falls, Diarrhea	Publicly-sponsored	Not reported; November 2003; "probability ratios of 0.75 and 0.25 to assign treatment"	No; NA
Howard, 2012	Europe	295; 52	Donepezil + Placebo, Donepezil + Memantine; MMSE, Mortality, AEs, Falls	Publicly-sponsored	Not reported; February 2008; Not reported	No; Do not own data
Hu, 2006	China	97; 16	Memantine, Donepezil; MMSE	NA	Not reported; Not reported; Not reported	No; NA
Johannsen, 2006	Belgium, Denmark, Germany, Greece, Hungary, Iceland, The Netherlands, Poland, USA	202; 48	Donepezil, Placebo/No treatment; MMSE, ADAS-cog, NPI, Headaches, Diarrhea, Nausea	Industry-sponsored	Not reported; February 1999; Not reported	No; Do not own data
Jones, 2004	UK, Finland, Germany and Norway	120; 12	Donepezil, Galantamine; MMSE, ADAS-cog, Headaches, Vomiting, Diarrhea, Nausea, AEs	Industry-sponsored	Not reported; Not reported; 1:1	No; Cannot share data (Old study)
Kadir, 2008	Sweden	18; 48	Galantamine, Placebo/No treatment; MMSE, ADAS-cog	Industry-sponsored + Other	Not reported; Not reported; Not reported	No; Cannot identify study

Kano, 2013;	Japan	30;<br>28	Donepezil, Donepezil + Memantine ;<br>MMSE	NA	Not reported;<br>August 2011;<br>Not reported	No;<br>NR
Karaman, 2005	Turkey	44;<br>52	Rivastigmine, Placebo/No treatment;<br>MMSE, ADAS-cog, ADAS-ADL, CIBIC-plus, Headaches, Vomiting, Nausea	NA	Not reported;<br>Not reported;<br>Not reported	No;<br>NR
Likitjaroen, 2012	Germany	25;<br>26	Galantamine, Placebo;<br>MMSE	Publicly-sponsored + Industry-sponsored	Not reported;<br>September 2006;<br>Not reported	No;<br>Do not own data
Lorenzi, 2011	Italy	15;<br>24	Memantine, Placebo/No treatment;<br>MMSE	Publicly-sponsored + Industry-sponsored	Not reported;<br>Not reported;<br>Not reported	No;<br>Do not own data
Maher-Edwards, 2011	Austria, Bulgaria, Chile, Estonia, Germany, Russia, Slovakia, and UK	129;<br>24	Donepezil, Placebo/no treatment;<br>ADAS-cog, CIBIC-plus, Mortality, AEs, Headaches, Nausea	Industry-sponsored	Not reported;<br>May 2006;<br>1:1:1	No;<br>No response from sponsor
Marek, 2014	UK, Ukraine, South Africa, Russia	132;<br>16	Donepezil, Placebo;<br>MMSE, ADAS-cog, NPI, CIBIC-plus, Mortality, Headaches, Vomiting, Diarrhea, AEs	Industry-sponsored	Not reported;<br>May 2010;<br>"equal proportions"	No;<br>Cannot share data
Mazza, 2006	Italy	51;<br>24	Donepezil, Placebo/No treatment;<br>MMSE	NA	Not reported;<br>March 2003;<br>1:1:1	No;<br>NR
Mohs, 2001	USA	431;<br>54	Donepezil, Placebo/No treatment;<br>MMSE, Mortality, AEs, Headaches, Diarrhea, Nausea	Industry-sponsored	Not reported;<br>Not reported;<br>Not reported	No;<br>Cannot share data
Moretti, 2014	Italy	20;<br>78	Rivastigmine Patch, Rivastigmine Oral;<br>MMSE	NA	Not reported;<br>Not reported;<br>Not reported	No;<br>NA
Mowla, 2007	Iran	81;<br>12	Rivastigmine, Placebo/No treatment;<br>MMSE	Publicly-sponsored	Not reported;<br>Not reported;<br>Not reported	No;<br>NA
Nakamura, 2011	Japan	855;<br>24	Rivastigmine, Placebo/No treatment;<br>MMSE, AEs, Vomiting, Nausea, Diarrhea	Industry-sponsored	Not reported;<br>January 2007;<br>Not reported	Yes;<br>NA
Nakano, 2001	Japan	35;<br>48	Donepezil, Placebo/No treatment;<br>MMSE	NA	Not reported;<br>Not reported;<br>Not reported	No;<br>NR
Nordberg, 2009	USA	63;<br>13	Rivastigmine, Donepezil, Galantamine;<br>AEs, Headaches, Vomiting, Diarrhea, Nausea	Industry-sponsored	Not reported;<br>Not reported;<br>1:1:1	No;<br>Cannot share data
Pakdaman H, 2015	Iran	198;<br>68.8	Donepezil, Galantamine, Rivastigmine;<br>MMSE, ADAS-cog, Mortality,	Industry-sponsored	Not reported;<br>Not reported;<br>Not reported	No;<br>NR

			Headaches, Vomiting, Diarrhea, Nausea			
Peng, 2005	China	89;<br>12	Donepezil, Placebo/No treatment;<br>MMSE	NA	Not reported;<br>1998;<br>Not reported	No;<br>NR
Peskind, 2006	USA	403;<br>24	Memantine, Placebo/no treatment;<br>ADAS-cog, ADCS-ADL, NPI, CIBIC-plus, Nausea, Vomiting, Diarrhea	Industry-sponsored	Not reported;<br>Not reported;<br>Not reported	No;<br>Cannot share data
Peters, 2015	Europe	226;<br>52	Galantamine + Memantine, Galantamine + Placebo;<br>ADAS-cog, ADCS-ADL, NPI, Mortality, AEs, Falls	Publicly-sponsored	Not reported;<br>Not reported;<br>Not reported	No;<br>NA
Reisberg, 2003	USA	252;<br>28	Memantine, Placebo/No treatment;<br>MMSE, ADCS-ADL, NPI, CIBIC-plus, Mortality, AEs, Diarrhea	Publicly-sponsored + Industry-sponsored	Not reported;<br>August 1998;<br>Not reported	No;<br>No response from sponsor
Rockwood, 2001	Australia, Canada, Great Britian, New Zealand, South Africa, USA	386;<br>12	Galantamine, Placebo/no treatment;<br>ADAS-cog, NPI, CIBIC-plus, Mortality, AEs, Vomiting, Nausea	Industry-sponsored	Not reported;<br>Not reported;<br>Not reported	Yes;<br>NA
Rockwood, 2006	Canada	130;<br>16	Galantamine, Placebo/no treatment;<br>ADAS-cog, CIBIC-plus, AEs, Vomiting, Nausea	Publicly-sponsored + Industry-sponsored	Not reported;<br>November 2001;<br>Not reported	No;<br>IPD not available
Rogers, 1996	USA	161;<br>12	Donepezil, Placebo/No treatment;<br>MMSE, ADAS-cog, Headaches, Diarrhea	Industry-sponsored	Not reported;<br>Not reported;<br>Not reported	No;<br>Cannot share data
Rogers, 1998	USA	468;<br>12	Donepezil, Placebo/No treatment;<br>MMSE, ADAS-cog, CIBIC-plus, AEs, Headaches, Vomiting, Diarrhea, Nausea	Industry-sponsored	Not reported;<br>Not reported;<br>Not reported	No;<br>Cannot share data
Rogers, 1998	USA	473;<br>24	Donepezil, Placebo/No treatment;<br>MMSE, ADAS-cog, CIBIC-plus, Mortality, AEs, Vomiting, Diarrhea, Nausea	Industry-sponsored	Not reported;<br>Not reported;<br>Not reported	No;<br>Cannot share data
Saxton, 2012	Australia, South Africa, New Zealand	264;<br>12	Memantine, Placebo;<br>Mortality, Falls, Headaches, Diarrhea, Nausea, AEs	Industry-sponsored	Not reported;<br>April 2007;<br>Not reported	No;<br>Cannot share data
Scarpini, 2011	Italy	139;<br>96	Galantamine, Placebo/no treatment;<br>Mortality, AEs	Industry-sponsored	Not reported;<br>July 2001;<br>Not reported	No;<br>IPD not available
Schmidt, 2008	Europe	36;<br>52	Memantine, Placebo/No treatment;<br>MMSE, ADAS-cog, ADCS-ADL	Industry-sponsored	Not reported;<br>Not reported;<br>Not reported	No;<br>No response from sponsor
Seltzer, 2004	USA	153;<br>24	Donepezil, Placebo/No treatment;<br>MMSE, ADAS-cog, Diarrhea, Nausea	Industry-sponsored	Not reported;<br>Not reported;<br>Not reported	No;<br>Cannot identify study

Shao, 2015	China	110;<br>24	Donepezil + Memantine, Galantamine + Memantine, Memantine + Placebo, Rivastigmine + Memantine;<br>MMSE, ADCS-ADL	NA	Not reported;<br>October 2009;<br>Not reported	No;<br>NR
Shimizu, 2015	Japan	75;<br>52	Donepezil, Galantamine, Rivastigmine;<br>MMSE, ADAS-cog, NPI, Headaches, Vomiting, Nausea	Industry-sponsored	Not reported;<br>Not reported;<br>Not reported	No;<br>Do not own data
Sole-Padulles, 2013	Spain	14;<br>13	No treatment, Donepezil;<br>MMSE, NPI	Industry-sponsored	Not reported;<br>Not reported;<br>Not reported	No;<br>Do not own data
Tariot, 2000	USA	978;<br>20	Galantamine, Placebo/no treatment;<br>ADAS-cog, ADCS-ADL, NPI, Mortality, AEs, Vomiting, Diarrhea, Nausea	Industry-sponsored	Not reported;<br>Not reported;<br>Not reported	No;<br>Cannot identify study
Tariot, 2001	USA	208;<br>24	Donepezil, Placebo/No treatment;<br>MMSE, Mortality, AEs, Bradycardia, Headaches, Vomiting, Diarrhea, Nausea	Industry-sponsored	Not reported;<br>Not reported;<br>Not reported	No;<br>Cannot identify study
Thomas, 2001	Italy	40;<br>24	Donepezil, Rivastigmine;<br>MMSE, ADAS-cog	NA	Not reported;<br>Not reported;<br>Not reported	No;<br>NR
Wilcock, 2003	UK	188;<br>52	Galantamine, Donepezil;<br>MMSE, ADAS-cog, Mortality, AEs, Falls, Headaches, Vomiting, Nausea	Industry-sponsored	Not reported;<br>June 2000;<br>Not reported	No;<br>Cannot identify study
Wilkinson, 2001	UK	180;<br>12	Galantamine, Placebo/no treatment;<br>ADAS-cog, AEs, Headaches, Vomiting, Diarrhea, Nausea	Industry-sponsored	Not reported;<br>May 1994;<br>Not reported	Yes;<br>NA
Wilkinson, 2002	UK, South Africa, and Switzerland	111;<br>12	Donepezil, Rivastigmine;<br>MMSE, ADAS-cog, Mortality, AEs, Bradycardia, Headaches, Vomiting, Nausea	Industry-sponsored	Not reported;<br>Not reported;<br>1:1	No;<br>Cannot identify study
Wilkinson, 2012	France, Germany, Switzerland, UK	277;<br>52	Memantine, Placebo/No treatment;<br>MMSE, NPI, Mortality, AEs, Falls	Industry-sponsored	Not reported;<br>September 2005;<br>1:1	Yes;<br>NA
Winblad, 2001	Denmark, Finland, Norway, Sweden, the Netherlands	286;<br>52	Donepezil, Placebo/No treatment;<br>MMSE, AEs, Bradycardia, Headaches, Diarrhea, Nausea	Industry-sponsored	Not reported;<br>Not reported;<br>Not reported	No;<br>Cannot identify study
Winblad, 2006	Sweden	248;<br>24	Donepezil, Placebo/No treatment;<br>MMSE, NPI, Mortality, AEs, Falls, Diarrhea, Nausea	Industry-sponsored	Not reported;<br>October 2002;<br>Not reported	No;<br>Cannot identify study
Winblad, 2007	Chile, Czech Republic, Denmark, Finland, Germany, Guatemala, Israel, Italy, Korea, Mexico, Norway, Peru, Poland, Portugal, Russia, Slovak Republic, Sweden, Taiwan, USA, Uruguay, Venezuela	1190;<br>24	Rivastigmine, Placebo/No treatment;<br>MMSE, ADAS-cog, ADCS-ADL, NPI, Mortality, AEs, Headaches, Vomiting, Diarrhea, Nausea	Industry-sponsored	Not reported;<br>November 2003;<br>Not reported	No;<br>No response from sponsor

Zhang-Yi, 2005	China	120;<br>8	Donepezil, Placebo/No treatment;<br>MMSE	NA	Not reported;<br>Not reported;<br>Not reported	No;<br>NR
Zhang, 2012	China	218;<br>16	Galantamine, Donepezil;<br>MMSE, ADAS-cog, ADCS-ADL, NPI, Mortality, Vomiting, Diarrhea, Nausea, AEs	Industry-sponsored	Not reported;<br>Not reported;<br>Not reported	No;<br>IPD not available

Appendix 6. Characteristics of studies with shared IPD

Study	Provided by	Severity of AD*	Previous response to treatment for AD	Presence of behavioural disturbance	Comorbid conditions	Other medications used	Treatment Group	Males (%)	Age, mean (SD)
Black 2007	CSDR - EISAI	Severe	NR	NR	All patients included the same exact comorbidities	NR	Donepezil	48 (27%)	78 (7.9)
							Placebo	54 (32%)	78 (8.1)
Gold 2010	CSDR - GSK	Mild-Moderate	NR	NR	Multiple reported	Multiple reported	Donepezil	16 (29%)	76.6 (8.2)
							Placebo	49 (46%)	75.5 (8.2)
Winblad 2007	CSDR - Novartis	Mild-Moderate	NR	NR	Multiple reported	Multiple reported	Rivastigmine patch	198 (33 %)	73.9 (8.0)
							Rivastigmine oral	102 (34 %)	72.9 (8.2)
							Placebo	101 (33%)	73.8 (7.5)
Hager 2014	YODA - Janssen	Mild-Moderate	NR	NR	NR	Multiple reported	Galantamine	354 (34%)	73 (8.9)
							Placebo	367 (36%)	73 (8.7)
Rockwood 2001	YODA - Janssen	Mild-Moderate	NR	NR	NR	Multiple reported	Galantamine	113 (43%)	75 (7.3)
							Placebo	58 (46%)	75 (7.6)
Cummings 2004	YODA - Janssen	NR	NR	NR	Multiple reported	Multiple reported	Galantamine	245 (35%)	76.9 (7.8)
							Placebo	108 (38%)	77.2 (7.9)
Burns 2009	YODA - Janssen	Severe	NR	NR	Multiple reported	Multiple reported	Galantamine	42 (20%)	84.0 (6.5)
							Placebo	39 (19%)	83.8 (6.7)
Gault 2015	AbbVie	Mild-Moderate	NR	NR	NR	Multiple reported	Donepezil	37 (54%)	72.4 (8.4)
							Placebo	26 (38%)	73.6 (8.2)
Haig 2014	AbbVie	Mild-Moderate	NR	NR	Multiple reported	Multiple reported	Donepezil	24 (40%)	70 (8.3)
							Placebo	24 (38%)	70 (7.8)
Bakchine 2008	Lundbeck	Mild-Moderate	NR	NR	NR	Multiple reported	Memantine	112 (35%)	74 (7.4)
							Placebo	61 (40%)	73 (6.9)
Herrman 2013	Lundbeck	69 (48%)	NR	NR	NR	Multiple reported	Memantine	77 (42%)	75 (7.9)
							Placebo	77 (41%)	75 (6.9)
Wilkinson 2012	Lundbeck	NR	NR	NR	NR	Multiple reported	Memantine	50 (38%)	74 (8.8)
							Placebo	69 (48%)	74 (7.8)

Additional characteristics of studies with shared IPD

Study	Patients experiencing at least one AE	Missing data in AE outcome	Baseline MMSE, mean (SD)	Final MMSE, mean (SD)	Change score, mean (SD)	Missing data in MMSE outcome	Total number of patients	Reasons for dropouts as indicated in the provided IPD	Time taken for the 1st AE
Black 2007	21	0 (0%)	7.5 (3.3)	8.2 (5.2)	0.63 (3.1)	27 (15%)	176 (51%)	• intercurrent illness (1 [2%] – donepezil = 1; placebo = 0),<br>• request of patient or investigator (4 [7%] –	617 days (range [110, 1292])

	25	0 (0%)	7.4 (3.6)	7.6 (4.8)	-0.15 (3.5)	27 (16%)	167 (49%)	donepezil = 3; placebo = 1),<br>• patient entered nursing home/facility (5 [9%] – donepezil = 1; placebo =) 4,<br>• due to adverse experience (30 [56%] – donepezil = 15; placebo = 15), and<br>• other (14 [26%] – donepezil = 7; placebo = 7)	691 days (range [78, 1475]).
Gold 2010	6	0 (0%)	20 (3.7)	21 (4.6)	1.11 (2.3)	18 (32%)	56 (34%)	• Adverse Event (16 [39%] – donepezil = 9; placebo = 7),<br>• Lost to Follow-Up (4 [10%] – donepezil = 3; placebo = 1),<br>• Non-compliance (6 [15%] – donepezil = 2; placebo = 4),<br>• Subject decided to withdraw (11 [26%] – donepezil = 4; placebo = 7)	349 days (range [48, 656])
	10	0 (0%)	20.1 (4.2)	20.4 (5.4)	0.08 (2.7)	23 (22%)	107 (66%)		492 days (range [95, 780])
Winblad 2007	83	0 (0%)	16.6 (3.0)	17.7 (4.7)	1 (3.4)	74 (10%)	598 (50 %)	NR	NR
	37	0 (0%)	16.4 (3.1)	17.2 (4.6)	0.8 (3.2)	31 (12%)	297 (25 %)	NR	NR
	45	0 (0%)	16.4 (3.0)	16.4 (5.3)	-0.1 (3.6)	21 (7%)	302 (25 %)	NR	NR
Hager 2014	73	0 (0%)	19.0 (4.1)	17.81 (6.2)	-1.38 (4.3)	228 (22%)	1027 (50%)	NR	NR
	92	0 (0%)	19.0 (4.0)	16.99 (6.3)	-2.15 (4.4)	236 (23%)	1022 (50%)	NR	NR
Rockwood 2001	27	0 (0%)	23.2 (5.2)	NR	NR	NR	261 (68%)	NR	NR
	5	0 (0%)	22.9 (5.0)	NR	NR	NR	125 (32%)	NR	NR
Cummings 2004	23	0 (0%)	20.7 (4.9)	NR	NR	NR	692 (71%)	NR	NR
	81	0 (0%)	20.6 (4.9)	NR	NR	NR	286 (29%)	NR	NR
Burns 2009	62	0 (0%)	NR	9.2 (4.5)†	NR	NR	211 (51%)	NR	NR
	75	0 (0%)	NR	9.6 (4.9)†	NR	NR	204 (49%)	NR	NR
Gault 2015	5	0 (0%)	19.2 (4.1)	20.7 (5.1)	1.5 (2.6)	48 (71%)	68 (50%)	NR	305 days (range [224, 377])
	3	0 (0%)	18.8 (4)	18.9 (4.8)	0.1 (2.4)	45 (66%)	68 (50%)	NR	239 days (range [206, 295])
Haig 2014	2	0 (0%)	17.9 (4.2)	19.7 (3.9)	1.2 (2.8)	41 (68%)	60 (49%)	NR	286 days (range N/A – a single date was provided)
	1	0 (0%)	17.8 (3.8)	19.9 (4.2)	1.8 (1.8)	47 (75%)	63 (51%)	NR	270 days (range [161, 379]).
Bakchine 2008	33	0 (0%)	18.7 (3.3)	NR	NR	NR	318 (68%)	NR	NR
	9	0 (0%)	18.9 (3.2)	NR	NR	NR	152 (32%)	NR	NR
Herrman 2013	18	0 (0%)	11.9 (3.1)	11.3 (4.9)	-0.76 (3.4)	31 (8%)	182 (49%)	NR	NR
	11	0 (0%)	11.8 (2.9)	11.1 (4.7)	-0.68 (3.2)	32 (9%)	187 (51%)	NR	NR

Wilkinson 2012	17	0 (0%)	16.7 (2.5)	16.4 (5.2)	-0.46 (3.9)	30 (11%)	133 (48%)	NR	NR
	20	0 (0%)	17.1 (2.4)	16.4 (5.6)	-0.69 (4.0)	30 (11%)	144 (52%)	NR	NR

* According to publication
† The MMSE final value comes from visit 8 (last available visit in IPD). MMSE was not reported in study publication
Abbreviations: AD, Alzheimer's Dementia; IPD, individual patient data; MMSE, Mini-Mental State Examination; NR, not reported; N/A, not applicable; AE, adverse event

Appendix 7: Comparison of studies with shared IPD with (a) all remaining studies and (b) studies for which sponsors claimed unavailable IPD. AD: aggregate data; IPD: individual patient data

a. Comparison of studies with shared IPD with all remaining studies (irrespective type of sponsor)

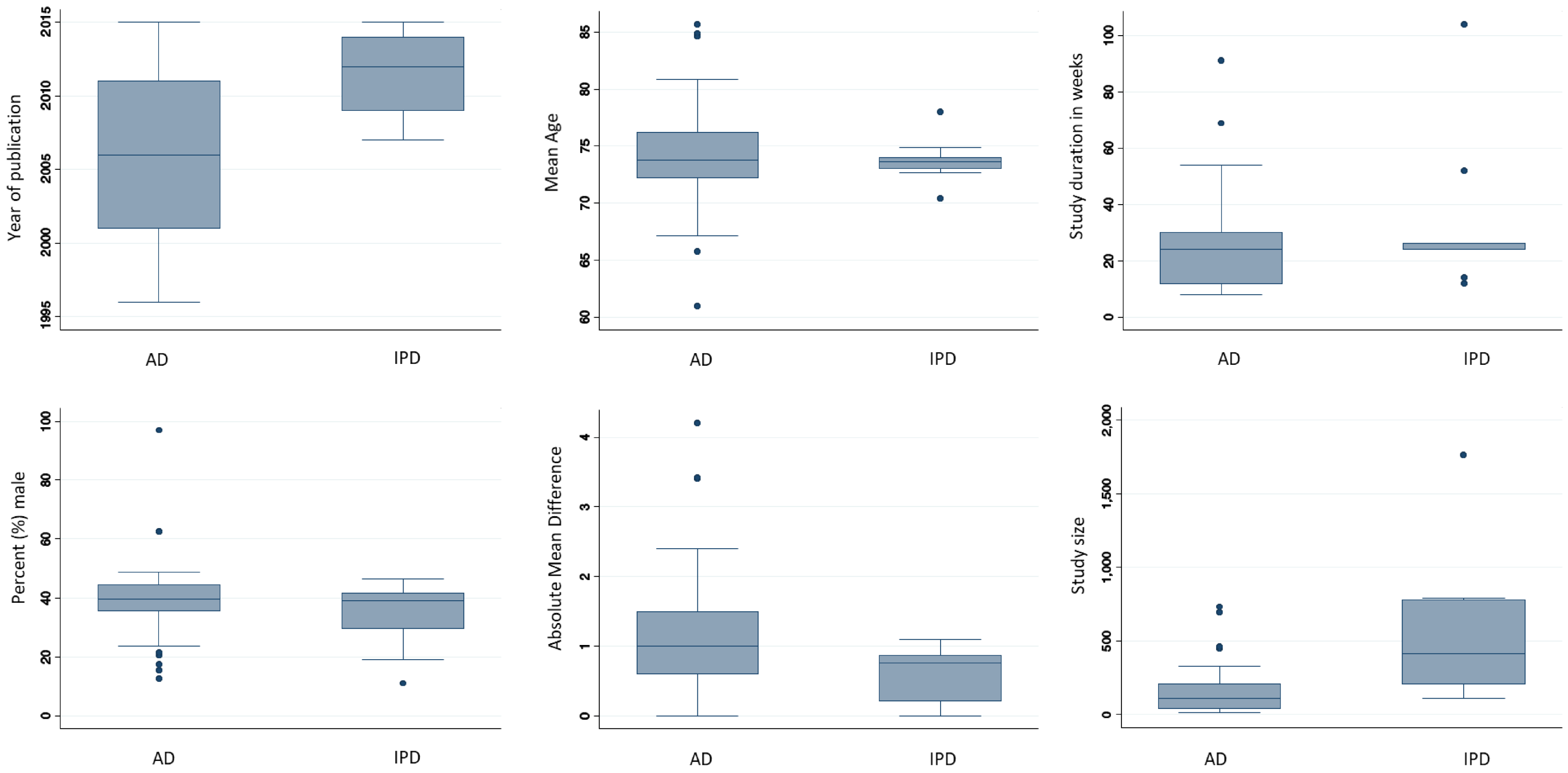

27

b. Comparison of studies with available and unavailable IPD (industry-sponsored studies only)

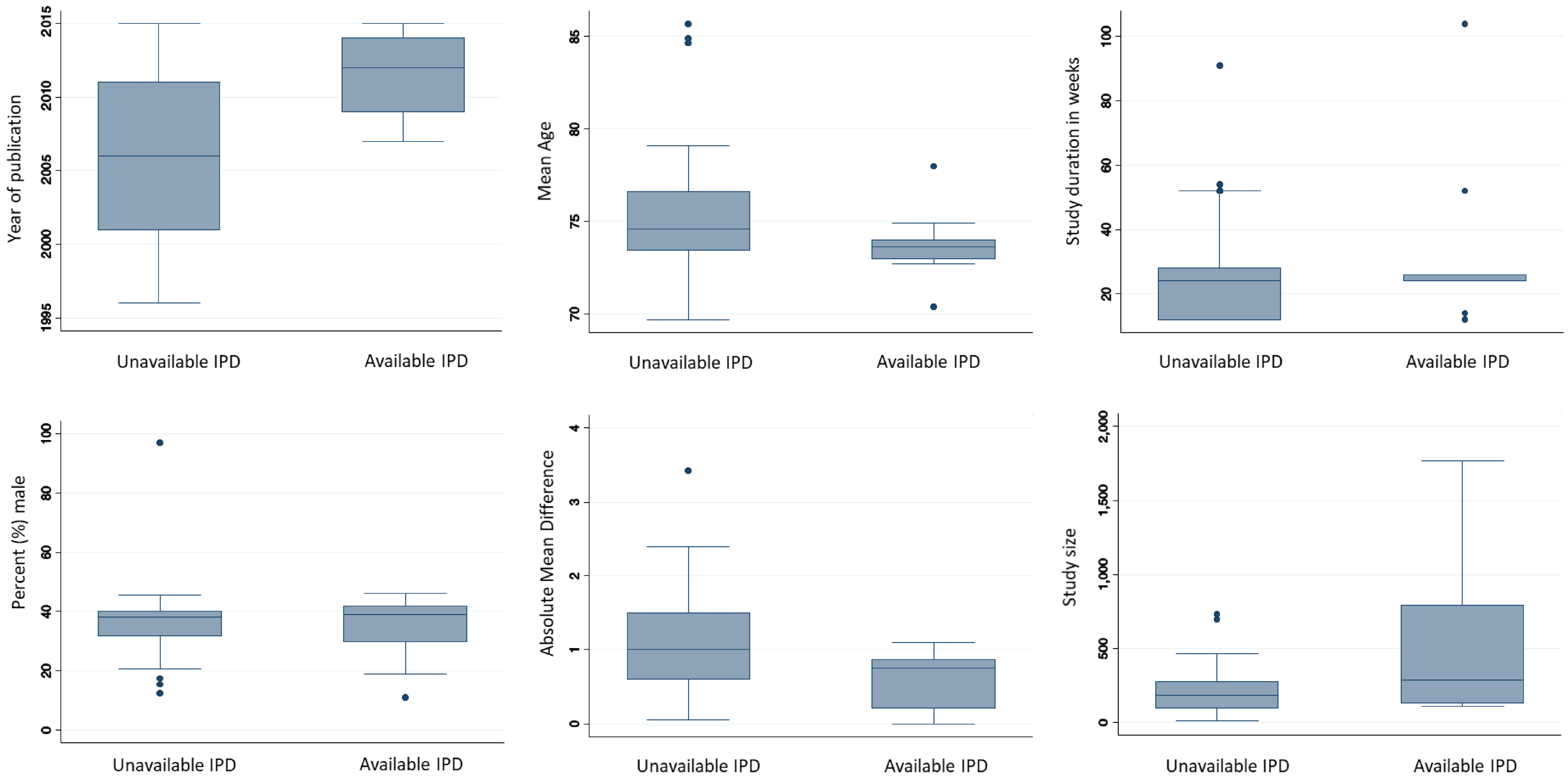

28

Appendix 8: Cochrane Risk-of-bias appraisal results (n = 80)

Study	1. Random sequence generation	2. Allocation concealment	3. Blinding of participants and personnel	4. Blinding of outcome assessment	5. Incomplete outcome data	6. Selective reporting	7. Other bias*
Agid, 1998	Low	High	Low	Unclear	High	Unclear	High
Ancoli-Israel, 2005	Unclear	Unclear	Unclear	Unclear	High	Unclear	High
Andersen, 2012	Unclear	Low	Low	Low	High	Low	Low
Araki, 2014	Low	Unclear	Unclear	Unclear	High	Unclear	Unclear
Bakchine, 2008	Low	Low	Low	Low	Low	High	High
Black, 2007	Low	Low	Low	Low	Low	Unclear	High
Blesa Gonzalez, 2011	Unclear	Unclear	High	Unclear	High	Low	High
Burns, 1999	Unclear	Unclear	Unclear	Unclear	High	Unclear	High
Burns, 2009	Low	Low	Low	Low	Low	Unclear	High
Burns, 2011	Low	Unclear	Low	Low	High	Unclear	Unclear
Choi, 2011	Unclear	Unclear	High	High	High	Low	Low
Corey-Bloom, 1998	Low	Low	Low	Low	High	Unclear	High
Cretu, 2008	Unclear	Unclear	Unclear	Low	Unclear	Unclear	Unclear
Dysken, 2014	Low	Low	Low	Unclear	Low	Low	Low
Farlow, 2013	Low	Unclear	Low	Low	High	Unclear	High
Feldman, 2001	Low	Unclear	Low	Low	High	Unclear	High
Feldman, 2007	Low	Low	Low	Low	High	Unclear	High
Fox, 2012	Low	Low	High	Low	High	High	Unclear
Frolich, 2011	Unclear	Unclear	Low	Low	High	Low	High
Fuschillo, 2001	Unclear	Unclear	Unclear	Unclear	Low	Low	Unclear
Gault, 2015	Low	Low	Low	Unclear	Low	Low	High
Gold, 2010	Low	Unclear	Low	Low	High	Low	High
Greenberg, 2000	Low	Low	Low	Unclear	High	Low	Low
Grossberg, 2013	Low	Low	Low	Low	High	Low	High
Hager K, 2014	Low	Low	Low	Low	High	High	High
Haig, 2014	Low	Low	Low	Low	High	Low	High
Hernández, 2007	Low	Low	Low	Low	Unclear	Low	Low
Herrmann, 2013	Low	Low	Low	Low	High	Low	High
Holmes, 2004	Low	Unclear	Low	Low	High	Low	High
Homma, 1998	Low	Low	Low	Low	Low	Unclear	High
Homma, 2008	Low	Low	Low	Low	High	Unclear	Unclear
Hong, 2006	Unclear	Unclear	Unclear	Unclear	Low	Unclear	Unclear
Howard, 2007	Low	Low	Low	Low	Low	Unclear	Low
Howard, 2012	Low	Low	Low	Low	High	Low	Low
Hu, 2006	Unclear	Unclear	Unclear	Unclear	Low	Unclear	Unclear
Johannsen, 2006	Unclear	Unclear	Low	Low	Low	Unclear	High
Jones, 2004	Low	Unclear	Unclear	Low	Low	Unclear	High
Kadir, 2008	Unclear	Unclear	Unclear	Unclear	High	Unclear	High
Kano, 2013	Unclear	Unclear	Unclear	Unclear	Low	Unclear	Unclear
Karaman, 2005	Low	Unclear	Low	Low	Unclear	Unclear	Unclear
Likitjaroen, 2012	Low	Low	Low	Unclear	High	High	Unclear
Lorenzi, 2011	Unclear	Unclear	Unclear	Unclear	Unclear	Unclear	High
Maher-Edwards, 2011	Low	Unclear	Unclear	Unclear	High	Unclear	High
Marek, 2014	Low	Low	Low	Low	High	Low	High
Mazza, 2006	Low	Unclear	Low	Low	High	Unclear	Unclear
Mohs, 2001	Low	Low	Low	Low	High	Unclear	High
Moretti, 2014	Unclear	Unclear	Unclear	Unclear	Low	Unclear	Low
Mowla, 2007	Low	Unclear	Low	Unclear	High	Unclear	Unclear
Nakamura, 2011	Unclear	Low	Low	Low	Low	Low	High
Nakano, 2001	Unclear	Unclear	Unclear	Unclear	Unclear	Unclear	Unclear
Nordberg, 2009	Unclear	Unclear	High	High	Unclear	Unclear	High
Pakdaman H, 2015	Low	Unclear	High	High	High	Unclear	Unclear
Peng, 2005	Unclear	Unclear	Unclear	Unclear	Low	Unclear	Unclear
Peskind, 2006	Low	Low	Low	Unclear	Low	Unclear	High
Peters, 2015	Unclear	Unclear	Low	Low	High	Low	Low
Reisberg, 2003	Low	Unclear	Low	Unclear	High	Low	Unclear
Rockwood, 2001	Low	Low	Low	Low	Unclear	Low	High
Rockwood, 2006	Low	Low	Low	Low	Low	Unclear	Unclear
Rogers, 1996	Unclear	Unclear	Low	Unclear	Low	Unclear	Unclear
Rogers, 1998	Unclear	Unclear	Low	Low	Low	Unclear	High
Rogers, 1998	Low	Unclear	Low	Unclear	High	Unclear	High
Saxton, 2012	Low	Low	Low	Low	Low	Low	High
Scarpini, 2011	Low	Low	Low	Unclear	High	Unclear	High
Schmidt, 2008	Low	Low	Low	Low	High	Unclear	High
Seltzer, 2004	Low	Unclear	Unclear	Unclear	Unclear	Unclear	High

Shao, 2015	Unclear	Unclear	Unclear	Unclear	Low	Unclear	Unclear
Shimizu, 2015	Low	Unclear	High	Low	High	Unclear	Unclear
Sole-Padulles, 2013	Unclear	Unclear	Unclear	Unclear	Low	Unclear	Unclear
Tariot, 2000	Low	Unclear	Low	Low	High	Low	High
Tariot, 2001	Low	Low	Low	Low	Unclear	Unclear	High
Thomas, 2001	Low	Unclear	Unclear	Unclear	Unclear	Unclear	Unclear
Wilcock, 2003	Unclear	Unclear	Unclear	Unclear	Unclear	Unclear	Unclear
Wilkinson, 2001	Low	Low	Low	Low	High	Unclear	High
Wilkinson, 2002	Low	Low	Low	Low	High	Unclear	High
Wilkinson, 2012	Low	High	Low	Low	High	Low	High
Winblad, 2001	Low	Unclear	Unclear	Low	High	Unclear	High
Winblad, 2006	Low	Low	Low	Low	High	Low	High
Winblad, 2007	Low	Low	Low	Low	High	Unclear	High
Yi, 2005	Unclear	Unclear	Unclear	Unclear	Low	Unclear	Unclear
Zhang, 2012	Unclear	Unclear	Unclear	Unclear	High	Unclear	High

* Other bias was categorized as:
a) *low risk of bias* when the study appeared to be free of other sources of bias,
b) *high risk of bias* when there was at least one important risk of bias. For example, when the study had:

- A potential source of bias related to the specific study design used; or
- A conflict of interest related to funding source; or
- An author was an employee of the drug company that sponsored the study; or
- Been claimed to have been fraudulent; or
- Other potential biases.

c) *unclear risk of bias* when there was a potential for bias, but there was either:

- Insufficient information to assess whether an important risk of bias exists; or
- Insufficient rationale/evidence that an identified problem would introduce bias; or
- Funding by drug company, but conflicts were not described

Appendix 9: Overall risk of bias for studies with shared IPD against (a) all remaining studies and (b) studies for which sponsors claimed unavailable IPD. AD: aggregate data; IPD: individual patient data

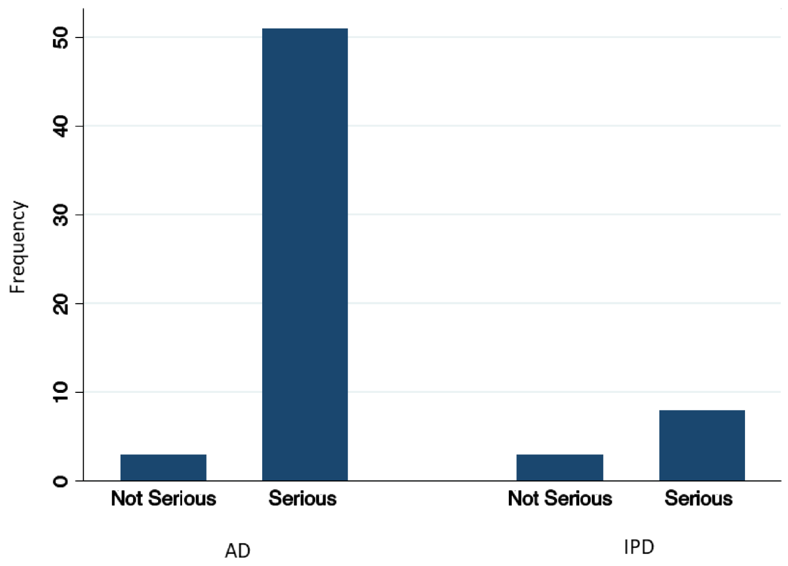

a. Comparison of studies with shared IPD with all remaining studies (irrespective type of sponsor)

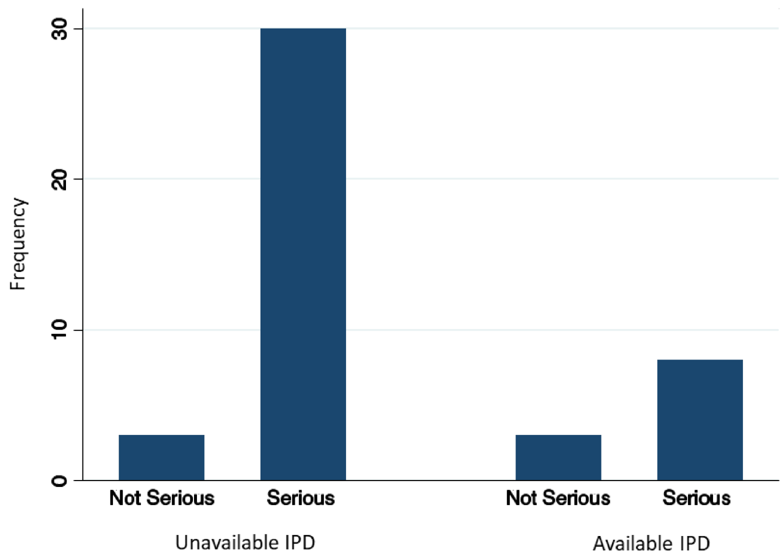

b. Comparison of studies with available and unavailable IPD (industry-sponsored studies only)

Veroniki AA, *et al. BMJ Open* 2022; 12:e053012. doi: 10.1136/bmjopen-2021-053012

Appendix 10: Study-specific effect sizes calculated from shared IPD and published data. IPD: individual patient data

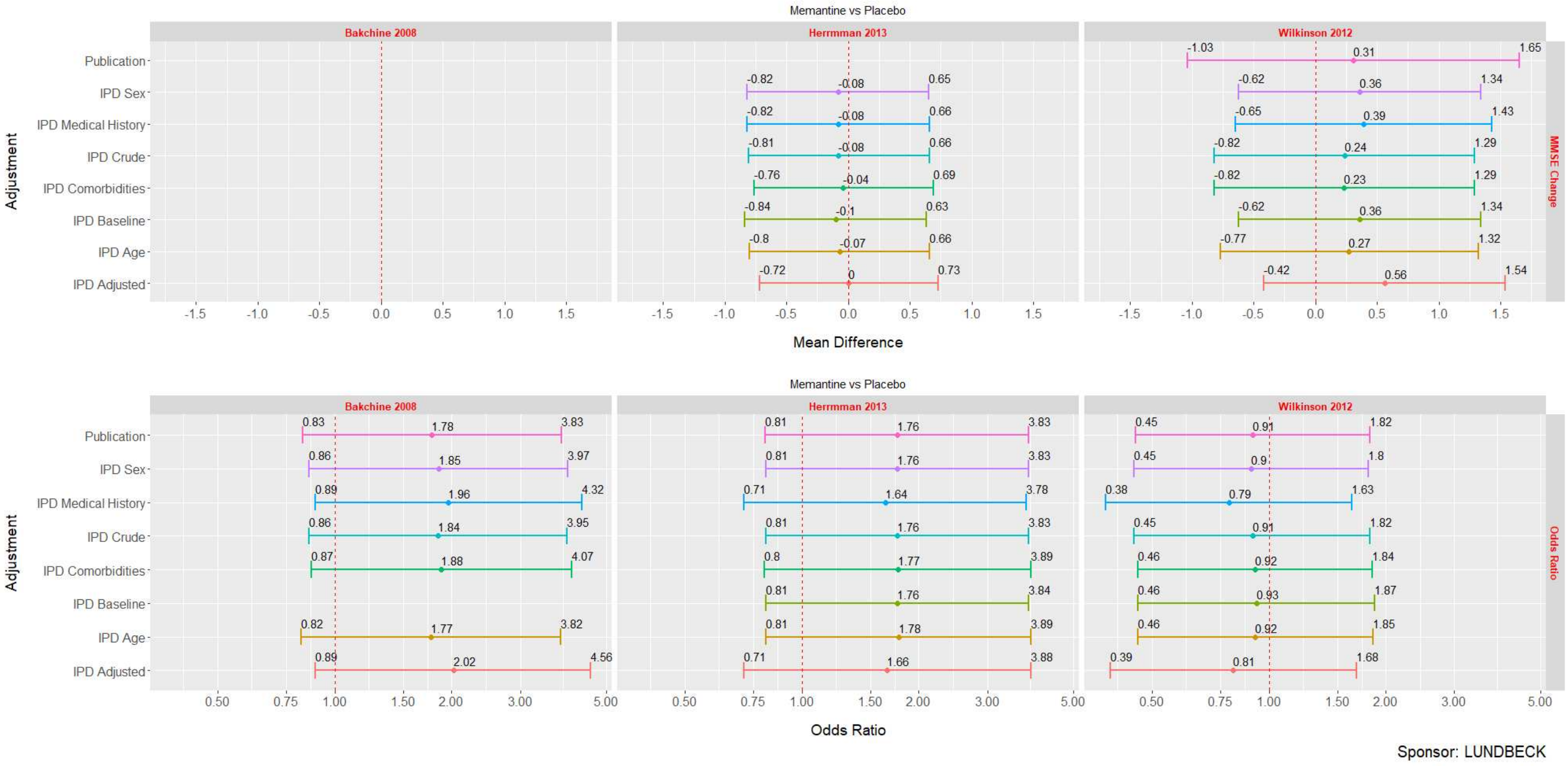

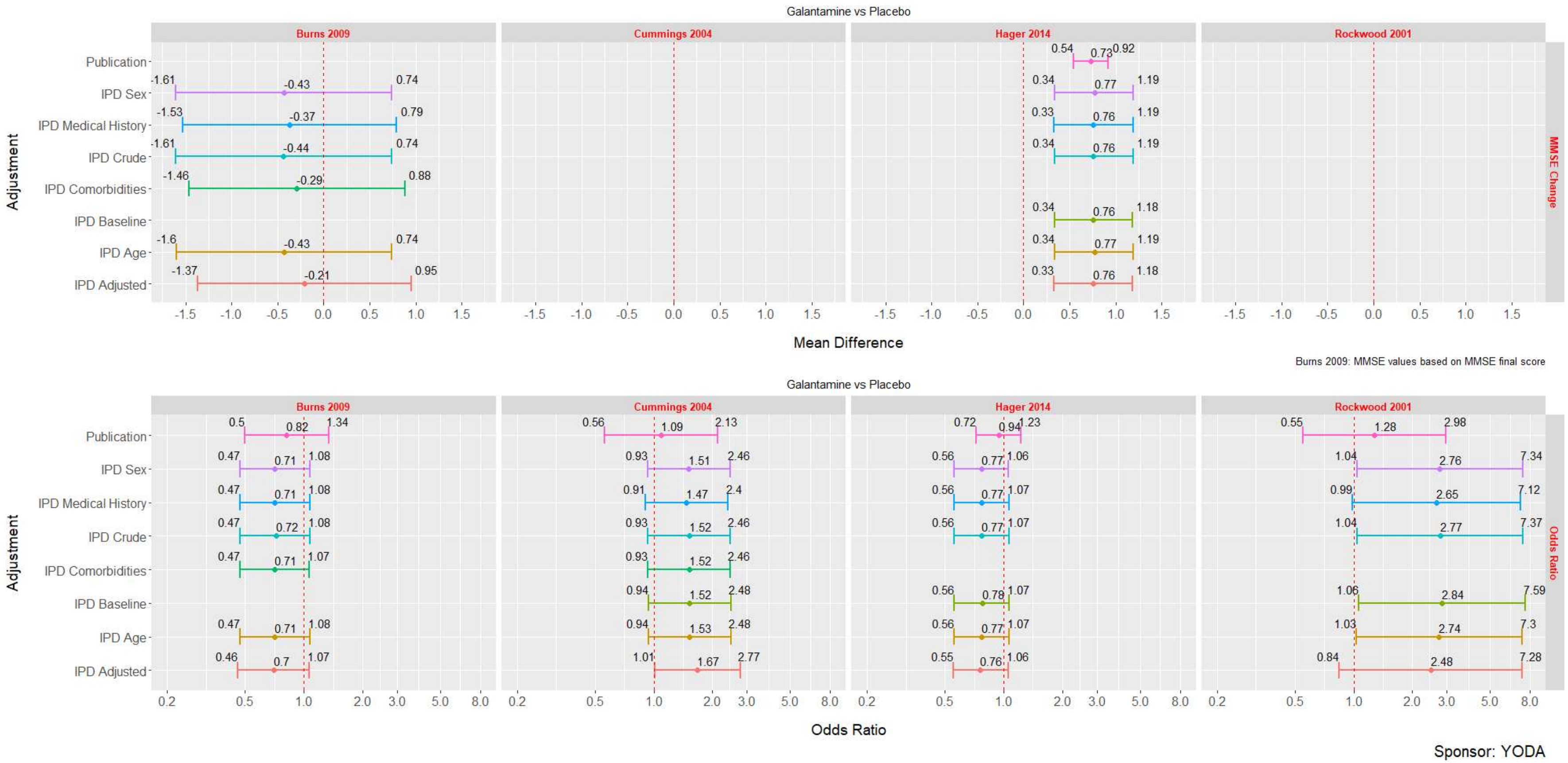
Galantamine vs Placebo
Burns 2009
Cummings 2004
Hager 2014
Rockwood 2001
Adjustment
Publication
IPD Sex
IPD Medical History
IPD Crude
IPD Comorbidities
IPD Baseline
IPD Age
IPD Adjusted
0.54 0.73 0.92
-1.61 -0.43 0.74
0.34 0.77 1.19
-1.53 -0.37 0.79
0.33 0.76 1.19
-1.61 -0.44 0.74
0.34 0.76 1.19
-1.46 -0.29 0.88
0.34 0.76 1.18
-1.6 -0.43 0.74
0.34 0.77 1.19
-1.37 -0.21 0.95
0.33 0.76 1.18
MMSE Change
Mean Difference
Burns 2009: MMSE values based on MMSE final score
Galantamine vs Placebo
Burns 2009
Cummings 2004
Hager 2014
Rockwood 2001
0.5 0.82 1.34
0.56 1.09 2.13
0.72 0.94 1.23
0.55 1.28 2.98
0.47 0.71 1.08
0.93 1.51 2.46
0.56 0.77 1.06
1.04 2.76 7.34
0.47 0.71 1.08
0.91 1.47 2.4
0.56 0.77 1.07
0.99 2.65 7.12
0.47 0.72 1.08
0.93 1.52 2.46
0.56 0.77 1.07
1.04 2.77 7.37
0.47 0.71 1.07
0.93 1.52 2.46
0.94 1.52 2.48
0.56 0.78 1.07
1.06 2.84 7.59
0.47 0.71 1.08
0.94 1.53 2.48
0.56 0.77 1.07
1.03 2.74 7.3
0.46 0.7 1.07
1.01 1.67 2.77
0.55 0.76 1.06
0.84 2.48 7.28
Odds Ratio
Sponsor: YODA

33

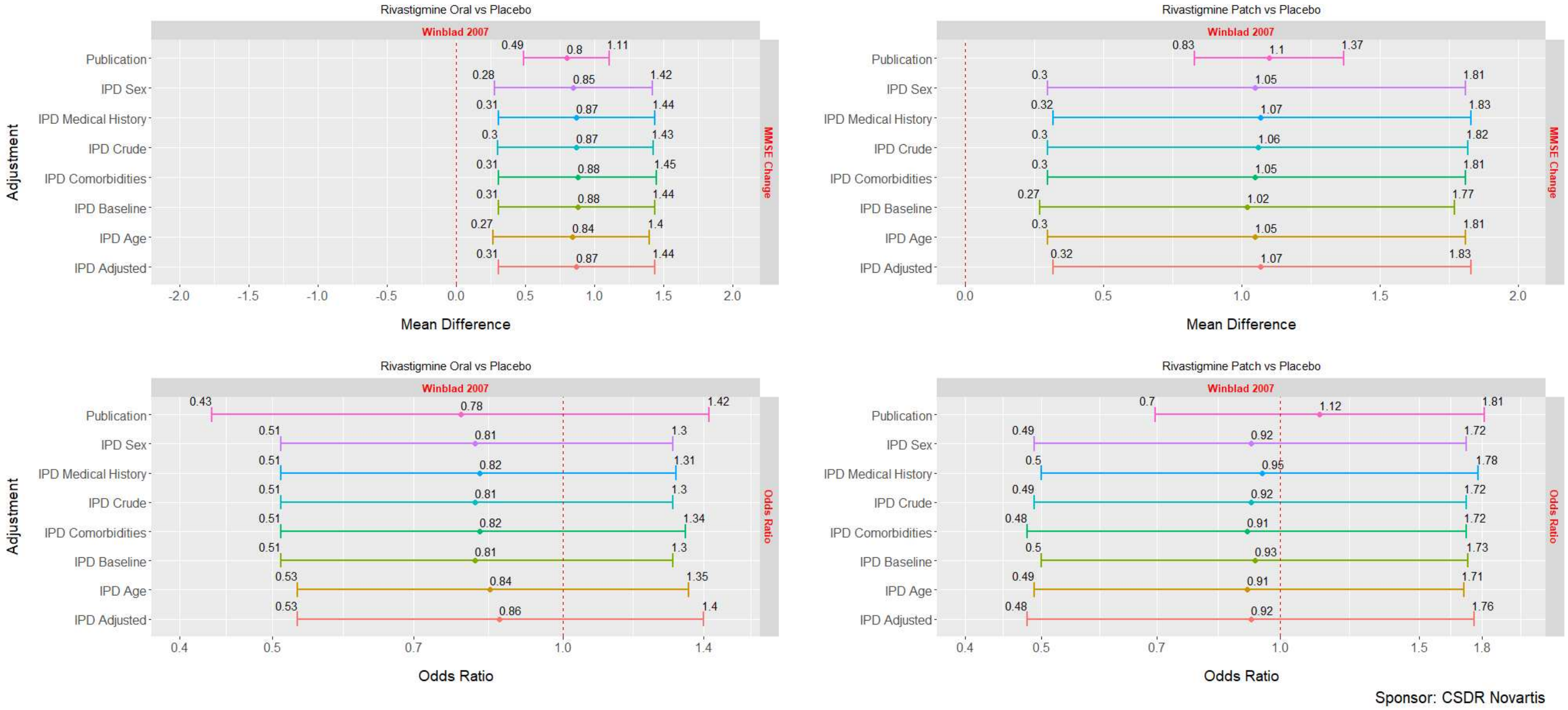
Rivastigmine Oral vs Placebo
Winblad 2007
MMSE Change
Adjustment
Publication 0.49 0.8 1.11
IPD Sex 0.28 0.85 1.42
IPD Medical History 0.31 0.87 1.44
IPD Crude 0.3 0.87 1.43
IPD Comorbidities 0.31 0.88 1.45
IPD Baseline 0.31 0.88 1.44
IPD Age 0.27 0.84 1.4
IPD Adjusted 0.31 0.87 1.44
-2.0 -1.5 -1.0 -0.5 0.0 0.5 1.0 1.5 2.0
Mean Difference
Rivastigmine Patch vs Placebo
Winblad 2007
MMSE Change
Publication 0.83 1.1 1.37
IPD Sex 0.3 1.05 1.81
IPD Medical History 0.32 1.07 1.83
IPD Crude 0.3 1.06 1.82
IPD Comorbidities 0.3 1.05 1.81
IPD Baseline 0.27 1.02 1.77
IPD Age 0.3 1.05 1.81
IPD Adjusted 0.32 1.07 1.83
0.0 0.5 1.0 1.5 2.0
Mean Difference
Rivastigmine Oral vs Placebo
Winblad 2007
Odds Ratio
Adjustment
Publication 0.43 0.78 1.42
IPD Sex 0.51 0.81 1.3
IPD Medical History 0.51 0.82 1.31
IPD Crude 0.51 0.81 1.3
IPD Comorbidities 0.51 0.82 1.34
IPD Baseline 0.51 0.81 1.3
IPD Age 0.53 0.84 1.35
IPD Adjusted 0.53 0.86 1.4
0.4 0.5 0.7 1.0 1.4
Odds Ratio
Rivastigmine Patch vs Placebo
Winblad 2007
Odds Ratio
Publication 0.7 1.12 1.81
IPD Sex 0.49 0.92 1.72
IPD Medical History 0.5 0.95 1.78
IPD Crude 0.49 0.92 1.72
IPD Comorbidities 0.48 0.91 1.72
IPD Baseline 0.5 0.93 1.73
IPD Age 0.49 0.91 1.71
IPD Adjusted 0.48 0.92 1.76
0.4 0.5 0.7 1.0 1.5 1.8
Odds Ratio
Sponsor: CSDR Novartis

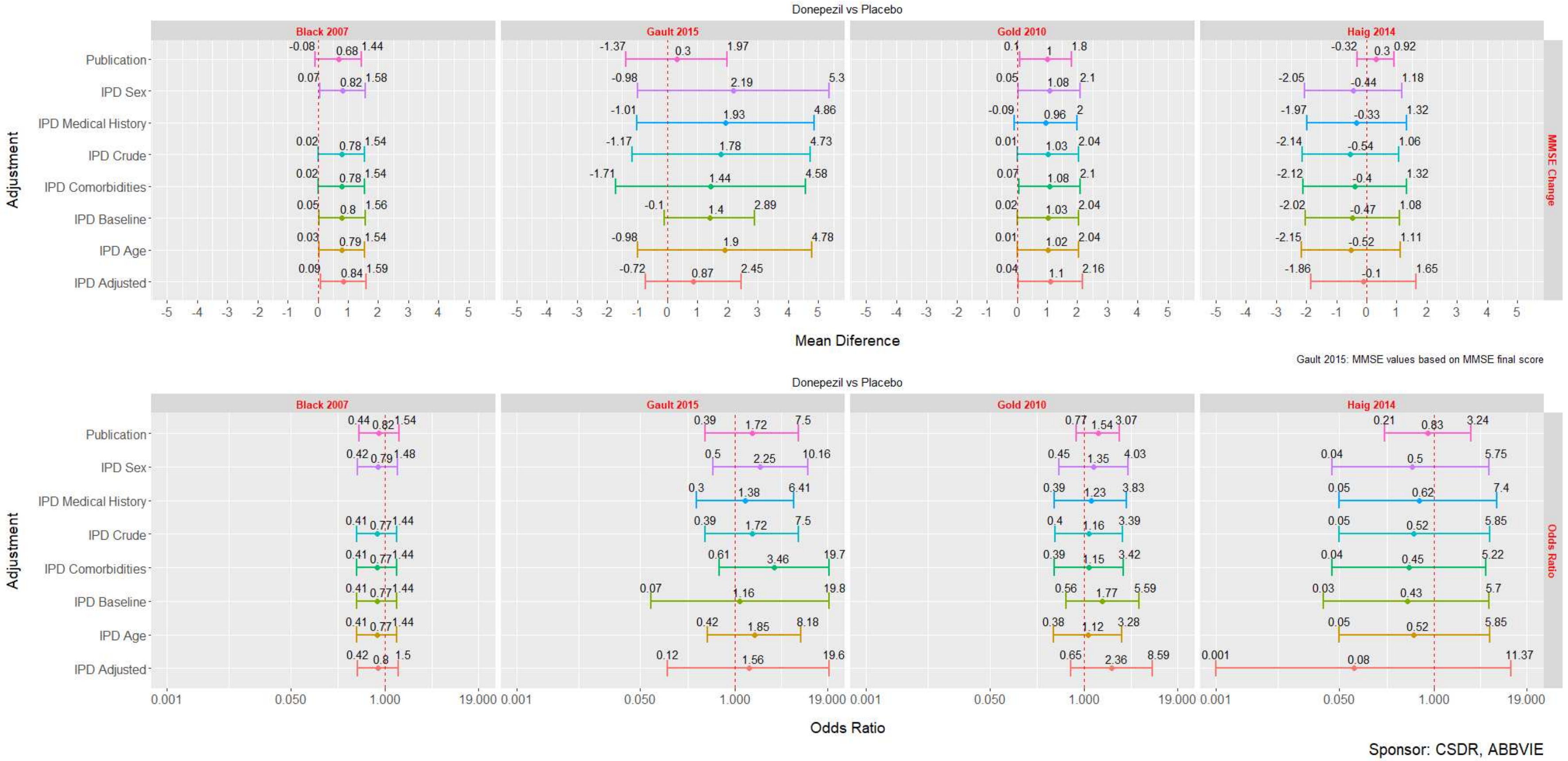

CSDR includes studies sponsored by GlaxoSmithKline, Eisai, Novartis, whereas YODA includes studies sponsored by Janssen

We also calculated the odds ratio for patients experiencing at least one AE excluding missing participants as shown in the MMSE outcome: Gold 2010: OR 2.78, 95% CI: 0.63-12.25; Black 2007: OR 1.19, 95% CI: 0.08-17.96; Winbland 2007: rivastigmine oral, OR 1.28, 95% CI: 0.09-18.16, rivastigmine patch, OR 0.81, 95% CI: 0.02-33.59; Wilkinson 2012: OR 0.84, 95% CI: 0.38-1.86; Herrmman 2013: OR 1.70, 95% CI: 0.71-4.08; Bachine 2008: OR 1.83, 95% CI: 0.77-4.32.

We were unable to assess this for studies obtained through YODA and AbbVie, since at the time of this assessement we did not have access to these data.

Abbreviations: IPD sex, regression analysis adjusting for sex; IPD medical history, regression analysis adjusting for medical history; IPD crude, analysis with no adjustments; IPD comorbidities, regression analysis adjusting for comorbidities; IPD baseline, regression analysis adjusting for MMSE baseline; IPD age, regression analysis adjusting for age; IPD adjusted, regression analysis adjusting for all available variables (we only considered those that we initially requested from sponsor)

Veroniki AA, *et al. BMJ Open* 2022; 12:e053012. doi: 10.1136/bmjopen-2021-053012

Appendix 11: Correlation between participant age and dropout in studies with IPD. IPD: individual patient data

	Study*	Correlation	P-Value
**CSDR**	Black 2007 (EISAI)	0.079	0.147
	Gold 2010 (GSK)	0.141	0.072
	Winblad 2007 (Novartis)	0.016	0.584
**Lundbeck**	Wilkinson 2012	0.066	0.273
	Herrmman 2013	0.124	0.017

* We were unable to assess this correlation for studies obtained through YODA and AbbVie, since at the time of this assessment we did not have access to these data

Appendix 12: Comparison Adjusted Funnel plot (all treatments vs placebo)

(a) MMSE

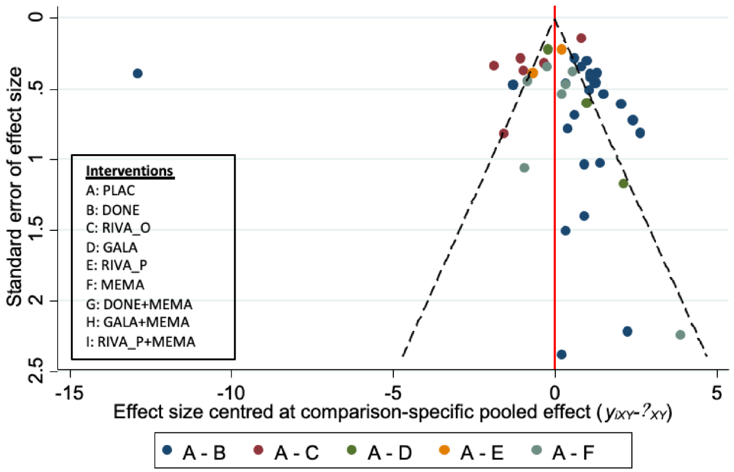

Note: Comparisons including only one study (when present) have been excluded

(b) Adverse Events

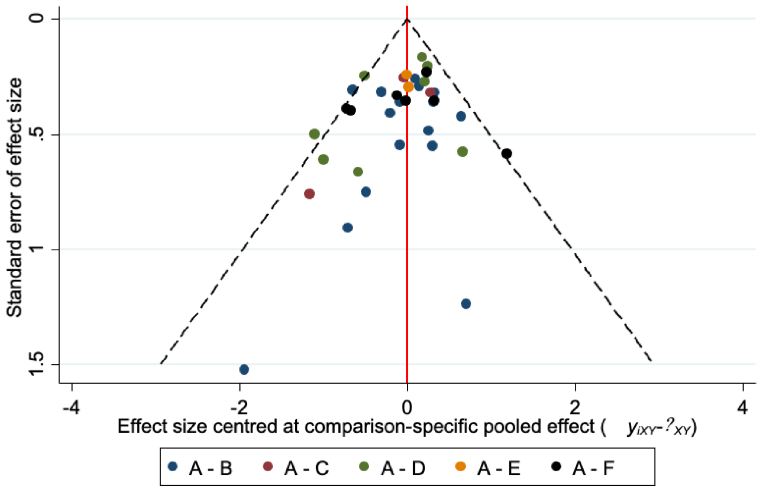

Note: Comparisons including only one study (when present) have been excluded

Appendix 13: Distribution of potential effect modifiers per treatment comparison and outcome

(a) MMSE

(b) Adverse Events

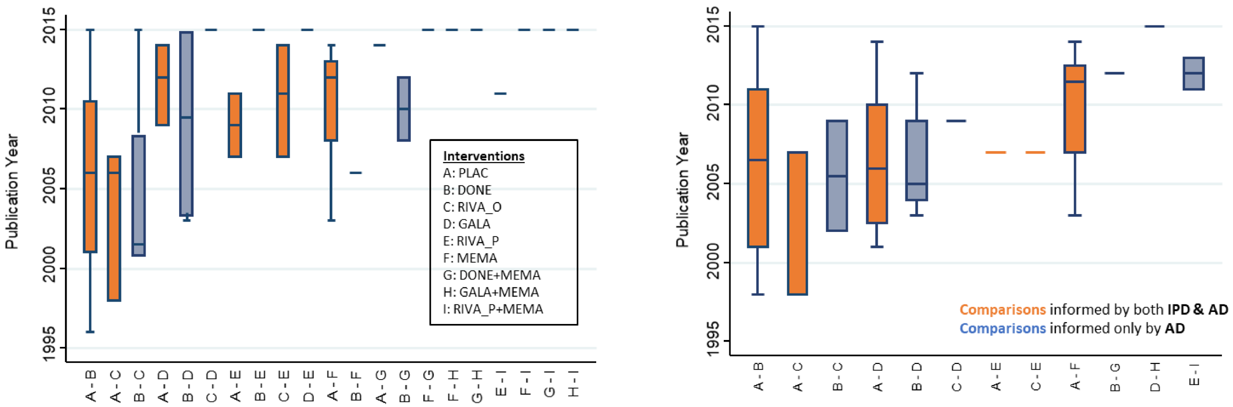

a. Publication year

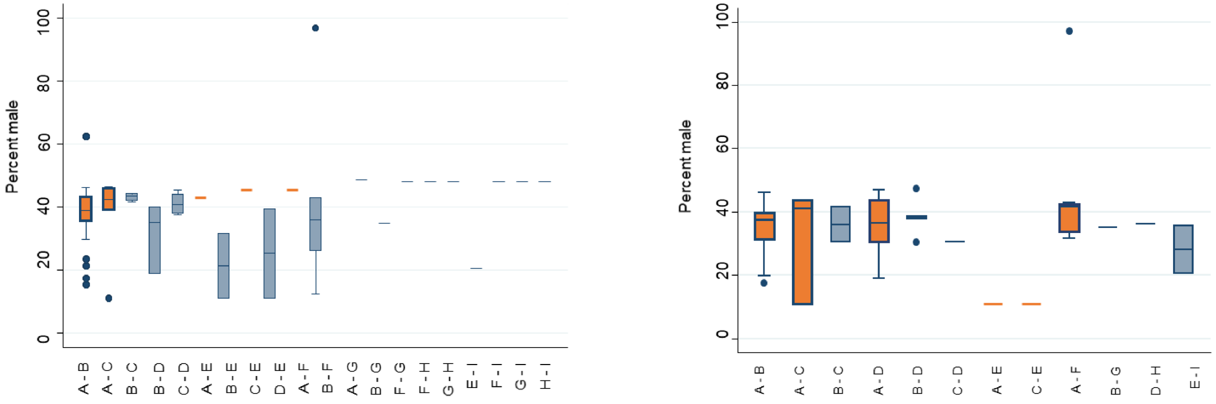

b. Percentage male

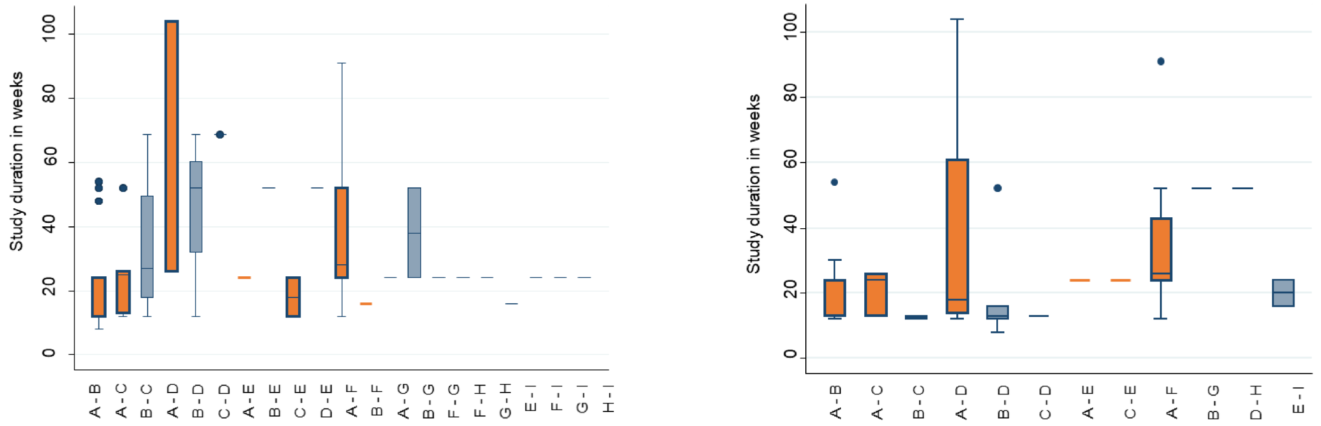

c. Study duration

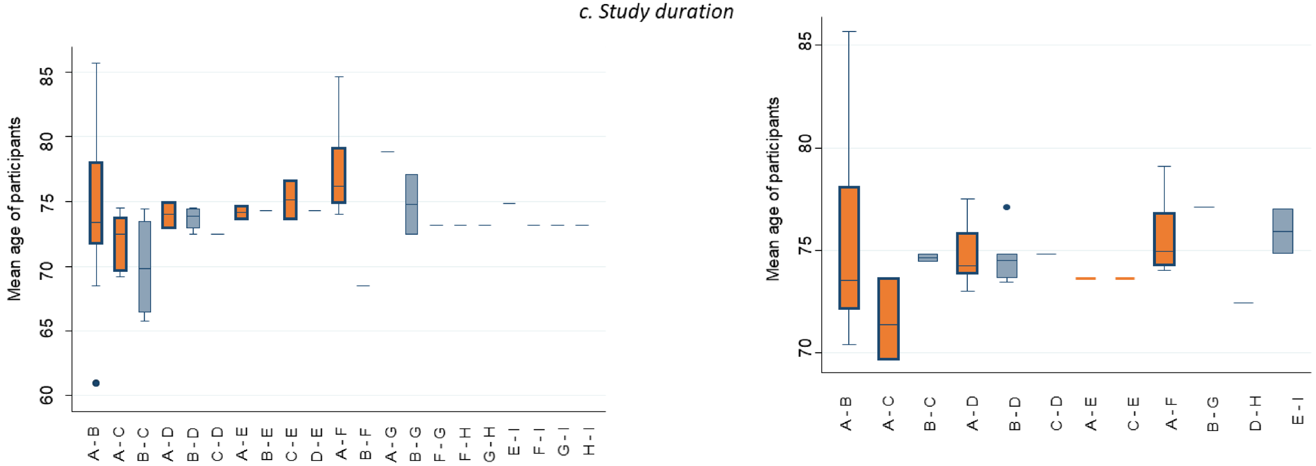

d. Mean participant age

Veroniki AA, *et al. BMJ Open* 2022; 12:e053012. doi: 10.1136/bmjopen-2021-053012

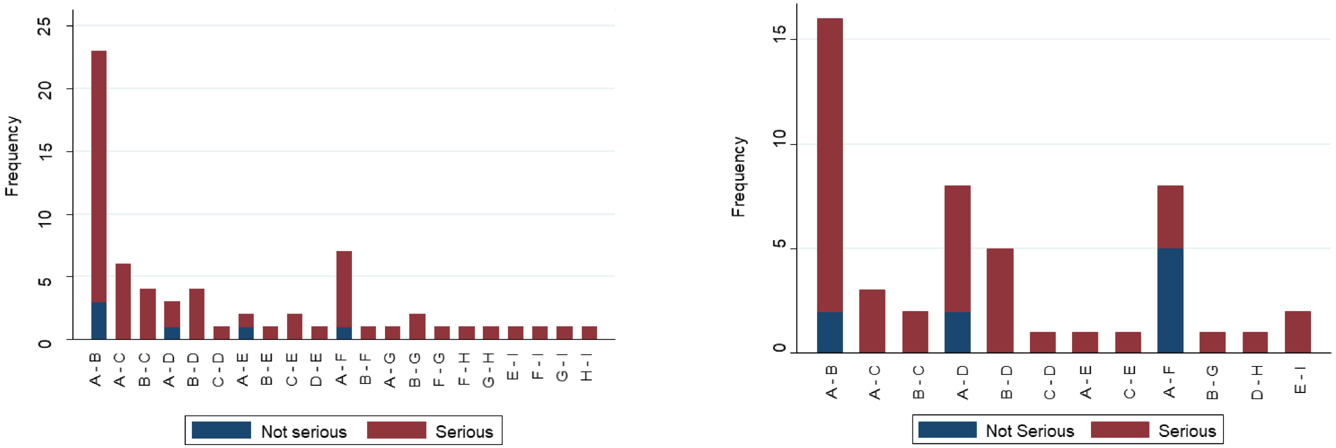

e. Overall Risk of Bias

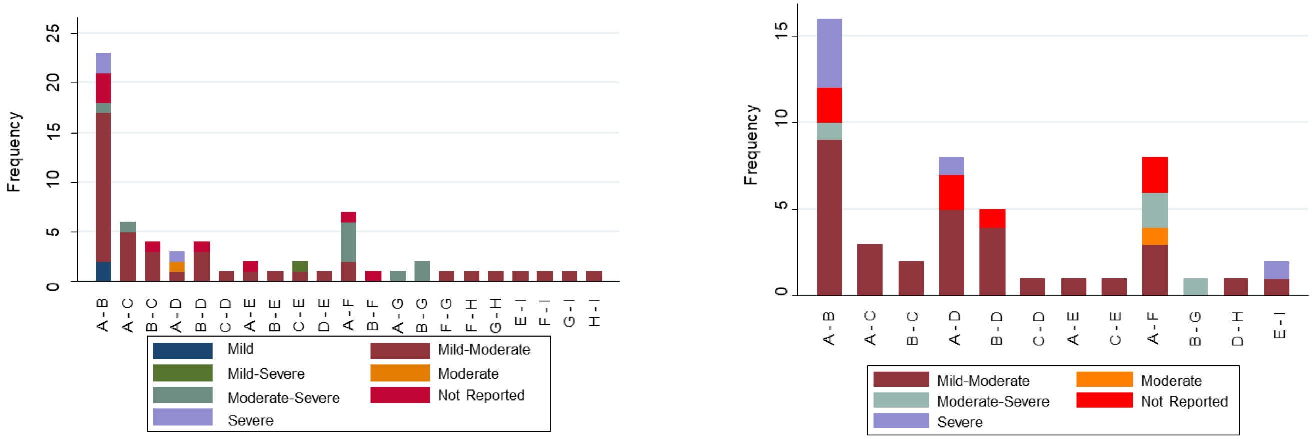

f. Alzheimer's Dementia Severity

Veroniki AA, *et al. BMJ Open* 2022; 12:e053012. doi: 10.1136/bmjopen-2021-053012

Appendix 14: Consistency Assessment – Loop-specific approach (using adjusted treatment effects)

(a) MMSE

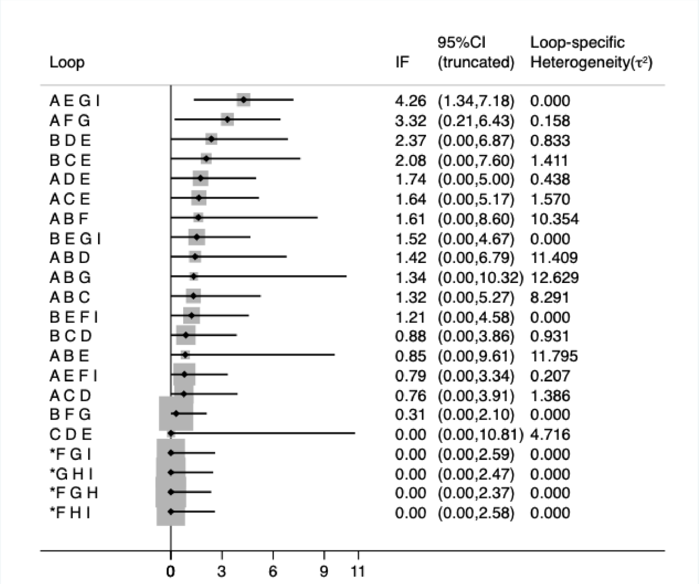

Note: * These loops are formed only by multi-arm trial(s)

Design-by-treatment interaction model:

χ^2 statistic: 4.36, 13 degrees of freedom, P value: 0.987, between-study variance: 7.34. I^2 statistic=96%

(b) Adverse Events

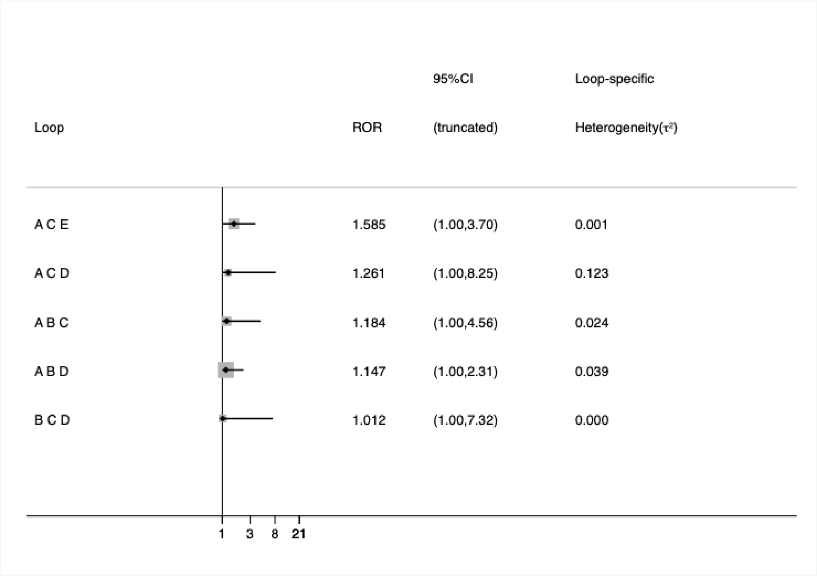

Design-by-treatment interaction model:

χ^2 statistic: 3.57, 6 degrees of freedom, P value: 0.735, between-study variance: 0.06. I^2 statistic=22%

Appendix 15: Network and standard meta-analysis results

Treatment Comparison	NMA estimate	95% CI	95% PI	P-score	MA estimate	95% CI	95% PI	#studies
**Mini-Mental State Examination (MMSE)*†**								
Donepezil vs Placebo	1.41	0.51 to 2.32	-3.48 to 6.31	0.59	1.65	0.16 to 3.14	-6.02 to 9.32	24
Rivastigmine oral vs Placebo	0.69	-0.79 to 2.18	-4.35 to 5.74	0.36	0.60	-0.43 to 1.62	-3.07 to 4.26	6
Galantamine vs Placebo	0.41	-1.44 to 2.26	-4.76 to 5.58	0.28	0.04	-1.09 to 1.17	-12.39 to 12.47	3
Rivastigmine transdermal vs Placebo	2.11	-0.04 to 4.26	-3.18 to 7.40	0.72	0.56	-0.33 to 1.45	--	2
Memantine vs Placebo	0.67	-0.99 to 2.34	-4.43 to 5.78	0.35	0.52	0.03 to 1.01	-0.69 to 1.73	7
Donepezil + Memantine vs Placebo	2.57	0.07 to 5.07	-2.88 to 8.02	0.80	4.21	1.94 to 6.48	--	1
Galantamine + Memantine vs Placebo	2.24	-2.13 to 6.61	-4.33 to 8.81	0.66				
Rivastigmine transdermal + Memantine vs Placebo	1.79	-1.70 to 5.27	-4.20 to 7.78	0.60				
Placebo (reference)				0.14				
Rivastigmine transdermal vs Rivastigmine oral	1.41	-0.80 to 3.62	-3.90 to 6.73		2.26	-0.48 to 4.99	-30.56 to 35.07	3
Rivastigmine oral vs Donepezil	-0.72	-2.28 to 0.84	-5.79 to 4.35		0.16	-0.57 to 0.90	-1.45 to 1.77	4
Galantamine vs Rivastigmine oral	-0.29	-2.48 to 1.91	-5.60 to 5.02		0.06	-1.05 to 1.17		1
Rivastigmine transdermal vs Donepezil	0.69	-1.52 to 2.91	-4.62 to 6.01		-0.20	-2.78 to 2.38		1
Rivastigmine transdermal vs Galantamine	1.70	-0.93 to 4.33	-3.81 to 7.21		2.20	-0.19 to 4.59		1
Rivastigmine transdermal + Memantine vs Rivastigmine transdermal	-0.32	-3.82 to 3.18	-6.32 to 5.68		-0.40	-1.40 to 0.60		1
Memantine vs Donepezil	-0.74	-2.56 to 1.08	-5.90 to 4.42		0.20	0.88 to 1.28		1
Donepezil + Memantine vs Donepezil	1.15	-1.33 to 3.64	-4.29 to 6.59		0.88	0.64 to 1.11		2
Galantamine vs Donepezil	-1.01	-2.86 to 0.84	-6.18 to 4.16		-0.35	-1.52 to 0.83	-5.31 to 4.62	4
Donepezil + Memantine vs Memantine	1.89	-0.88 to 4.67	-3.69 to 7.48		0.37	-1.04 to 1.78		1
Galantamine + Memantine vs Memantine	1.57	-2.78 to 5.92	-4.98 to 8.12		0.82	-0.58 to 2.22		1

Veroniki AA, *et al. BMJ Open* 2022; 12:e053012. doi: 10.1136/bmjopen-2021-053012

Rivastigmine transdermal + Memantine vs Memantine	1.12	-2.47 to 4.70	-4.93 to 7.16		0.41	-1.17 to 1.99		1
Galantamine + Memantine vs Donepezil + Memantine	-0.33	-4.72 to 4.06	-6.91 to 6.23		0.45	-0.85 to 1.75		1
Rivastigmine transdermal + Memantine vs Donepezil + Memantine	-0.78	-4.53 to 2.97	-6.93 to 5.38		0.04	-1.45 to 1.53		1
Rivastigmine transdermal + Memantine vs Galantamine + Memantine	-0.45	-5.05 to 4.14	-7.18 to 6.28		-0.41	-1.89 to 1.07		1
Common within-network between-study variance τ^2 = 5.75, I^2 = 96% (96%, 97%)								
Design-by-treatment interaction model for inconsistency χ^2 *(d.f., P-value,* τ^2*)*: 4.36 (13, 0.987, 7.35)								
**Adverse Events (AEs)***‡								
Donepezil vs Placebo	1.08	0.87 to 1.35	0.67 to 1.75	0.30	1.07	0.88 to 1.31	0.84 to 1.37	16
Rivastigmine oral vs Placebo	1.26	0.82 to 1.94	0.69 to 2.33	0.16	1.26	0.75 to 2.12	0.01 to 161.35	3
Galantamine vs Placebo	0.95	0.74 to 1.22	0.58 to 1.55	0.53	1.02	0.71 to 1.46	0.38 to 2.77	8
Rivastigmine transdermal vs Placebo	0.90	0.58 to 1.42	0.48 to 1.69	0.57	0.86	0.53 to 1.40		1
Memantine vs Placebo	0.88	0.64 to 1.20	0.52 to 1.49	0.63	0.87	0.63 to 1.20	0.38 to 1.99	8
Donepezil + Memantine vs Placebo	0.77	0.34 to 1.73	0.30 to 1.96	0.69				
Galantamine + Memantine vs Placebo	1.03	0.45 to 2.39	0.39 to 2.70	0.43				
Rivastigmine transdermal + Memantine vs Placebo	0.72	0.32 to 1.59	0.28 to 1.81	0.75				
Placebo (reference)				0.44				
Rivastigmine oral Donepezil vs	1.17	0.73 to 1.87	0.61 to 2.22		2.08	0.21 to 20.73		2
Galantamine vs Donepezil	0.88	0.64 to 1.19	0.52 to 1.49		0.79	0.46 to 1.39	0.32 to 1.96	5
Donepezil + Memantine vs Donepezil	0.71	0.33 to 1.55	0.29 to 1.76		0.71	0.37 to 1.38		1
Rivastigmine transdermal vs Rivastigmine oral	0.72	0.42 to 1.23	0.36 to 1.44		0.94	0.52 to 1.68		1
Rivastigmine transdermal + Memantine vs Rivastigmine transdermal	0.79	0.41 to 1.54	0.36 to 1.77		0.79	0.45 to 1.39		2
Galantamine vs Rivastigmine oral	0.75	0.46 to 1.22	0.39 to 1.45		0.63	0.15 to 2.64		1

Galantamine + Memantine vs Galantamine	1.09	0.49 to 2.42	0.43 to 2.75		1.09	0.55 to 2.17		1
Common within-network between-study variance τ2 = 0.04, I^2 = 22% (0%, 48%)								
Design-by-treatment interaction model for inconsistency χ^2 *(d.f., P-value,* τ^2*)*: 3.57 (6, 0.735, 0.06)								

* Aggregate data and fully adjusted results from studies with available individual patient data were used in both meta-analysis and NMA. The mean difference effect size is presented for MMSE and the odds ratio for AE.
† MMSE: Studies with available IPD included only available participants –to assess the missing data impact on the second stage (IMDoM) a separate analysis was applied
‡ AE: Studies with available IPD included all randomized participants

Veroniki AA, *et al. BMJ Open* 2022; 12:e053012. doi: 10.1136/bmjopen-2021-053012

Appendix 16: Network subgroup and meta-regression analysis results

Treatment Comparison	NMA estimate	95% CI	95%PI	P-score
**Mini-Mental State Examination (MMSE)†**				
**Mean Difference: Aggregate data and crude results from studies with available individual patient data**				
Donepezil vs Placebo	1.41	0.50 to 2.33	-3.51 to 6.34	0.59
Rivastigmine oral vs Placebo	0.69	-0.80 to 2.19	-4.38 to 5.76	0.36
Galantamine vs Placebo	0.37	-1.49 to 2.23	-4.82 to 5.57	0.28
Rivastigmine transdermal vs Placebo	2.10	-0.06 to 4.26	-3.22 to 7.42	0.72
Memantine vs Placebo	0.63	-1.05 to 2.30	-4.51 to 5.76	0.34
Donepezil + Memantine vs Placebo	2.56	0.04 to 5.07	-2.92 to 8.04	0.79
Galantamine + Memantine vs Placebo	2.22	-2.18 to 6.61	-4.39 to 8.82	0.66
Rivastigmine transdermal + Memantine vs Placebo	1.77	-1.73 to 5.27	-4.25 to 7.79	0.60
Placebo (reference)				0.14
Common within-network between-study variance τ^2 = 5.81, I^2 = 96% (96%, 97%)				
Design-by-treatment interaction model for inconsistency χ^2 *(d.f., P-value,* τ^2*)*: 4.42 (13, 0.986, 7.44)				
**Mean Difference: Aggregate data results****				
Donepezil vs Placebo	1.55	0.41 to 2.68	-4.16 to 7.25	0.57
Rivastigmine oral vs Placebo	0.71	-1.10 to 2.52	-5.18 to 6.60	0.34
Galantamine vs Placebo	0.57	-1.98 to 3.12	-5.61 to 6.74	0.32
Rivastigmine transdermal vs Placebo	2.60	-0.20 to 5.40	-3.69 to 8.89	0.75
Memantine vs Placebo	0.82	-1.37 to 3.01	-5.21 to 6.84	0.37
Donepezil + Memantine vs Placebo	2.71	-0.17 to 5.60	-3.62 to 9.04	0.76
Galantamine + Memantine vs Placebo	2.44	-2.61 to 7.48	-5.19 to 10.07	0.65
Rivastigmine transdermal + Memantine vs Placebo	2.09	-1.98 to 6.15	-4.89 to 9.07	0.61
Placebo (reference)				0.15
Common within-network between-study variance τ^2 = 7.66, I2 = 97% (96%, 97%)				
Design-by-treatment interaction model for inconsistency χ^2 *(d.f., P-value,* τ^2*)*: 3.92 (11, 0.972, 8.76)				
**Mean Difference: Crude results from studies with available individual patient data**				
Donepezil vs Placebo	0.70	0.01 to 1.40	-0.67 to 2.07	0.65
Rivastigmine oral vs Placebo	0.87	-0.01 to 1.75	-0.70 to 2.44	0.73
Galantamine vs Placebo	0.45	-0.24 to 1.14	-0.91 to 1.82	0.48
Rivastigmine transdermal vs Placebo	1.06	0.04 to 2.08	-0.67 to 2.79	0.82
Memantine vs Placebo	0.05	-0.74 to 0.83	-1.42 to 1.51	0.20
Placebo (reference)				0.13
Common within-network between-study variance τ^2 = 0.12, I^2 = 29% (0%, 71%)				
Design-by-treatment interaction model for inconsistency χ^2 *(d.f., P-value,* τ^2*)*: N/A (no closed loops)				
**Mean Difference: Low Risk of Bias for Allocation Concealment***				
Donepezil vs Placebo	2.02	-0.24 to 4.28	-6.19 to 10.23	0.70
Rivastigmine oral vs Placebo	1.38	-2.27 to 5.02	-7.39 to 10.14	0.57
Galantamine vs Placebo	-0.31	-4.61 to 3.98	-9.42 to 8.79	0.31
Rivastigmine transdermal vs Placebo	0.82	-4.08 to 5.72	-8.63 to 10.27	0.48
Memantine vs Placebo	0.69	-3.01 to 4.39	-8.10 to 9.49	0.46
Donepezil + Memantine vs Placebo	2.88	-4.75 to 10.51	-8.48 to 14.23	0.69
Placebo (reference)				0.30
Common within-network between-study variance: τ^2 = 13.82, I^2 = 98% (98%, 99%)				
Design-by-treatment interaction model for inconsistency χ^2 *(d.f., P-value,* τ^2*)*: 0.13 (3, 0.99, 19.10)				
**Mean Difference: Low risk of bias for Incomplete Data***				
Donepezil vs Placebo	0.87	0.07 to 1.66	-1.67 to 3.40	0.61
Rivastigmine oral vs Placebo	-1.52	-4.41 to 1.37	-5.54 to 2.50	0.10
Galantamine vs Placebo	0.52	-0.94 to 1.99	-2.36 to 3.41	0.48
Rivastigmine transdermal vs Placebo	1.37	-0.64 to 3.38	-1.91 to 4.65	0.71
Memantine vs Placebo	0.57	-1.12 to 2.27	-2.47 to 3.62	0.48
Donepezil + Memantine vs Placebo	0.94	-2.11 to 4.00	-3.23 to 5.11	0.57
Galantamine + Memantine vs Placebo	1.39	-1.66 to 4.44	-2.77 to 5.56	0.70
Rivastigmine transdermal + Memantine vs Placebo	0.98	-2.15 to 4.12	-3.26 to 5.23	0.58
Placebo (reference)				0.27
Common within-network between-study variance: τ^2 = 1.16, I^2 = 79% (65%, 88%)				
Design-by-treatment interaction model for inconsistency χ^2 *(d.f., P-value,* τ^2*)*: 12.15 (3, 0.007, 0.863)				
**Mean Difference: Publicly-Sponsored Studies***				
Donepezil vs Placebo	6.57	-4.68 to 17.81	-129.61 to 142.74	0.71
Rivastigmine oral vs Placebo	1.40	-16.41 to 19.21	-161.58 to 164.38	0.44
Memantine vs Placebo	0.11	-17.65 to 17.87	-162.64 to 162.86	0.39
Rivastigmine transdermal + Memantine vs Placebo	5.83	-7.98 to 19.64	-139.93 to 151.59	0.65
Placebo (reference)				0.32

Common within-network between-study variance: τ^2 = 81.93, I^2 = 99% (99%, 100%)				
Design-by-treatment interaction model for inconsistency χ^2 (d.f., P-value, τ^2): 0.05 (1, 0.815, 116.71)				
**Mean Difference: Industry-Sponsored Studies***				
Donepezil vs Placebo	0.98	0.69 to 1.27	0.10 to 1.86	0.85
Rivastigmine oral vs Placebo	0.82	0.35 to 1.29	-0.14 to 1.78	0.69
Galantamine vs Placebo	0.41	-0.15 to 0.96	-0.60 to 1.41	0.34
Rivastigmine transdermal vs Placebo	0.80	0.18 to 1.41	-0.25 to 1.84	0.67
Memantine vs Placebo	0.60	0.06 to 1.15	-0.39 to 1.60	0.50
Rivastigmine transdermal + Memantine vs Placebo	0.40	-1.02 to 1.81	-1.29 to 2.08	0.39
Placebo (reference)				0.06
Common within-network between-study variance: τ^2 = 0.16, I^2 = 43% (15%, 62%)				
Design-by-treatment interaction model for inconsistency χ^2 (d.f., P-value, τ^2): 8.06 (7, 0.327, 0.16)				
**Mean Difference: Studies with Mild to Moderate cognitive impairment, assessed with MMSE at baseline ***				
Donepezil vs Placebo	1.68	0.31 to 3.06	-4.81 to 8.18	0.69
Rivastigmine oral vs Placebo	0.88	-1.29 to 3.05	-5.85 to 7.61	0.51
Galantamine vs Placebo	0.31	-2.47 to 3.09	-6.66 to 7.28	0.40
Rivastigmine transdermal vs Placebo	2.74	-0.68 to 6.16	-4.53 to 10.01	0.81
Memantine vs Placebo	-0.58	-4.84 to 3.69	-8.31 to 7.16	0.28
Donepezil + Memantine vs Placebo	0.43	-6.36 to 7.21	-9.06 to 9.91	0.45
Galantamine + Memantine vs Placebo	0.88	-5.90 to 7.66	-8.61 to 10.37	0.51
Rivastigmine transdermal + Memantine vs Placebo	1.11	-4.20 to 6.42	-7.30 to 9.52	0.55
Placebo (reference)				0.31
Common within-network between-study variance: τ^2 = 9.67, I^2 = 97% (97%, 98%)				
Design-by-treatment interaction model for inconsistency χ^2 (d.f., P-value, τ^2): 3.22 (9, 0.96, 13.28)				
**Mean Difference: Studies with Moderate to Severe cognitive impairment, assessed with MMSE at baseline ***				
Donepezil vs Placebo	1.31	0.66 to 1.96	-0.01 to 2.63	0.78
Rivastigmine oral vs Placebo	-1.00	-1.87 to -0.12	-2.51 to 0.51	0.04
Galantamine vs Placebo	-0.21	-1.64 to 1.21	-2.28 to 1.86	0.28
Memantine vs Placebo	0.69	0.07 to 1.31	-0.61 to 2.00	0.59
Donepezil + Memantine vs Placebo	2.49	1.55 to 3.44	0.92 to 4.07	1.00
Placebo (reference)				0.32
Common within-network between-study variance: τ^2 = 0.18, I^2 = 44% (0%, 75%)				
Design-by-treatment interaction model for inconsistency χ^2 (d.f., P-value, τ^2): 2.60 (1, 0.11, 0.11)				
**Mean Difference: Excluding outlier studies*[§]**				
Donepezil vs Placebo	0.95	0.59 to 1.32	-0.64 to 2.54	0.57
Rivastigmine oral vs Placebo	0.65	0.09 to 1.22	-1.00 to 2.30	0.37
Galantamine vs Placebo	0.36	-0.38 to 1.09	-1.36 to 2.07	0.22
Rivastigmine transdermal vs Placebo	1.03	0.15 to 1.91	-0.76 to 2.82	0.59
Memantine vs Placebo	0.67	0.02 to 1.32	-1.01 to 2.35	0.39
Donepezil + Memantine vs Placebo	2.04	1.03 to 3.05	0.18 to 3.90	0.92
Galantamine + Memantine vs Placebo	1.87	0.08 to 3.66	-0.53 to 4.26	0.82
Rivastigmine transdermal + Memantine vs Placebo	1.10	-0.33 to 2.53	-1.03 to 3.23	0.58
Placebo (reference)				0.04
Common within-network between-study variance: τ^2 = 0.59, I^2 = 73% (64%, 79%)				
Design-by-treatment interaction model for inconsistency χ^2 (d.f., P-value, τ^2): 10.60 (13, 0.64, 0.61)				
**Accounting for missing outcome data - Informative Missingness Difference of Means[¶]**				
Donepezil vs Placebo	1.42	0.51 to 2.33	0.51 to 2.33	0.59[‖]
Rivastigmine oral vs Placebo	0.45	-1.09 to 1.99	-1.09 to 1.99	0.30[‖]
Galantamine vs Placebo	0.19	-1.78 to 2.17	-1.78 to 2.17	0.25[‖]
Rivastigmine transdermal vs Placebo	2.37	-0.03 to 4.79	-0.03 to 4.79	0.76[‖]
Memantine vs Placebo	0.60	-1.09 to 2.42	-1.09 to 2.42	0.36[‖]
Donepezil + Memantine vs Placebo	2.55	0.09 to 5.01	0.09 to 5.01	0.80[‖]
Galantamine + Memantine vs Placebo	2.26	-2.03 to 6.56	-2.03 to 6.56	0.68[‖]
Rivastigmine transdermal + Memantine vs Placebo	1.81	-1.66 to 5.28	-1.66 to 5.28	0.61[‖]
Placebo (reference)				0.16[‖]
Common within-network between-study variance: τ^2 = 5.47[‖]				
Design-by-treatment interaction model for inconsistency χ^2 (d.f., P-value, τ^2): 4.45 (11, 0.955, 6.45)				
**Mean Difference: Meta-regression, Trial Mean Age[**]**				
Donepezil vs Placebo	1.53	0.52 to 2.53	-3.17 to 6.27	0.50 [††]
Rivastigmine oral vs Placebo	0.80	-0.84 to 2.44	-4.15 to 5.79	0.37 [††]
Galantamine vs Placebo	0.60	-1.63 to 2.83	-4.57 to 5.72	0.25 [††]
Rivastigmine transdermal vs Placebo	2.53	0.06 to 4.98	-2.72 to 7.80	0.75 [††]
Memantine vs Placebo	0.79	-1.18 to 2.74	-4.33 to 5.85	0.37 [††]
Donepezil + Memantine vs Placebo	2.66	0.09 to 5.19	-2.70 to 7.97	0.87 [††]
Galantamine + Memantine vs Placebo	2.39	-2.02 to 6.84	-4.14 to 8.83	0.75 [††]
Rivastigmine transdermal + Memantine vs Placebo	2.05	-1.53 to 5.59	-3.83 to 7.94	0.75 [††]
Placebo (reference)				0.12 [††]
Regression coefficient	0.03	-0.14 to 0.20		
Common within-network between-study variance: τ^2 = 5.50	3.72 to 8.51			

Design-by-treatment interaction model for inconsistency χ² (d.f., P-value, τ²): 3.92 (11, 0.972, 8.76)				
**Mean Difference: NMA of studies with IPD adjusted for Age**				
Donepezil vs Placebo	0.72	0.03 to 1.42	-0.66 to 2.10	0.66
Rivastigmine oral vs Placebo	0.84	-0.05 to 1.73	-0.75 to 2.43	0.70
Galantamine vs Placebo	0.46	-0.24 to 1.15	-0.92 to 1.83	0.48
Rivastigmine transdermal vs Placebo	1.05	0.04 to 2.06	-0.68 to 2.78	0.83
Memantine vs Placebo	0.06	-0.72 to 0.84	-1.40 to 1.53	0.21
Placebo (reference)				0.12
Common within-network between-study variance: τ^2 = 0.12, I^2 = 29% (0%, 71%)				
Design-by-treatment interaction model for inconsistency χ² (d.f., P-value, τ²): N/A (one closed loop with a single multi-arm trial)				
**Mean Difference: Meta-regression, Percent of Male Participants**[**]				
Donepezil vs Placebo	1.62	0.58 to 2.65	-3.40 to 6.61	0.62 [††]
Rivastigmine oral vs Placebo	0.73	-0.90 to 2.35	-4.30 to 5.81	0.37 [††]
Galantamine vs Placebo	0.62	-1.65 to 2.89	-4.75 to 5.93	0.25 [††]
Rivastigmine Transdermal vs Placebo	2.51	0.01 to 5.04	-2.78 to 7.94	0.75 [††]
Memantine vs Placebo	0.66	-1.47 to 2.77	-4.54 to 5.88	0.25 [††]
Donepezil + Memantine vs Placebo	2.52	-0.40 to 5.45	-3.09 to 8.17	0.75 [††]
Galantamine + Memantine vs Placebo	2.27	-2.28 to 6.83	-4.37 to 8.90	0.75 [††]
Rivastigmine transdermal + Memantine vs Placebo	1.98	-1.67 to 5.65	-4.02 to 7.99	0.75 [††]
Placebo (reference)				0.12 [††]
Regression coefficient	0.01	-0.05 to 0.06		
Common within-network between-study variance: τ^2 = 5.73	3.83 to 8.84			
Design-by-treatment interaction model for inconsistency χ² (d.f., P-value, τ²): 3.72 (10, 0.959, 8.97)				
**Mean difference: NMA of studies with IPD adjusted for Percent of Male Participants**				
Donepezil vs Placebo	0.76	0.05 to 1.47	-0.67 to 2.19	0.67
Rivastigmine oral vs Placebo	0.85	-0.07 to 1.77	-0.80 to 2.50	0.69
Galantamine vs Placebo	0.45	-0.27 to 1.16	-0.99 to 1.88	0.46
Rivastigmine transdermal vs Placebo	1.05	0.01 to 2.09	-0.74 to 2.84	0.81
Memantine vs Placebo	0.10	-0.68 to 0.89	-1.40 to 1.61	0.23
Placebo (reference)				0.11
Common within-network between-study variance: τ^2 = 0.13, I^2 = 32% (0%, 72%)				
Design-by-treatment interaction model for inconsistency χ² (d.f., P-value, τ²): N/A (one closed loop with a single multi-arm trial)				
**Mean Difference: NMA of studies with IPD adjusted for cognitive impairment, assessed with MMSE at baseline**				
Donepezil vs Placebo	0.79	0.26 to 1.32	-0.06 to 1.64	0.64
Rivastigmine oral vs Placebo	0.88	0.31 to 1.45	-0.05 to 1.81	0.69
Galantamine vs Placebo	0.76	0.34 to 1.18	0.08 to 1.44	0.62
Rivastigmine transdermal vs Placebo	1.02	0.27 to 1.77	-0.20 to 2.24	0.82
Memantine vs Placebo	0.07	-0.52 to 0.66	-0.89 to 1.03	0.14
Placebo (reference)				0.08
Common within-network between-study variance: τ^2 = 0.00, I^2 = 0% (0%, 79%)				
Design-by-treatment interaction model for inconsistency χ² (d.f., P-value, τ²): N/A (one closed loop with a single multi-arm trial)				
**Mean Difference: NMA of studies with IPD adjusted for comorbidities**				
Donepezil vs Placebo	0.77	0.21 to 1.33	-0.15 to 1.68	0.71
Rivastigmine oral vs Placebo	0.88	0.31 to 1.45	-0.05 to 1.81	0.75
Galantamine vs Placebo	-0.29	-1.46 to 0.88	-2.19 to 1.61	0.15
Rivastigmine transdermal vs Placebo	1.05	0.30 to 1.80	-0.17 to 2.27	0.88
Memantine vs Placebo	0.05	-0.55 to 0.64	-0.92 to 1.01	0.27
Placebo (reference)				0.15
Common within-network between-study variance: τ^2 = 0.00, I^2 = 0% (0%, 67%)				
Design-by-treatment interaction model for inconsistency χ² (d.f., P-value, τ²): N/A (one closed loop with a single multi-arm trial)				
**Mean Difference: NMA of studies with IPD adjusted for other medications**				
Donepezil vs Placebo	0.67	-0.34 to 1.69	-1.44 to 2.79	0.61
Rivastigmine oral vs Placebo	0.87	-0.12 to 1.86	-1.21 to 2.95	0.71
Galantamine vs Placebo	0.42	-0.35 to 1.19	-1.40 to 2.25	0.47
Rivastigmine transdermal vs Placebo	1.07	-0.04 to 2.18	-1.16 to 3.30	0.81
Memantine vs Placebo	0.11	-0.74 to 0.96	-1.80 to 2.02	0.26
Placebo (reference)				0.14
Common within-network between-study variance: τ^2 = 0.17, I^2 = 35% (0%, 76%)				
Design-by-treatment interaction model for inconsistency χ² (d.f., P-value, τ²): N/A (one closed loop with a single multi-arm trial)				
**Mean Difference: Meta-regression, Study Duration**[**]				
Donepezil vs Placebo	1.66	0.67 to 2.66	-3.12 to 6.32	0.62 [††]
Rivastigmine oral vs Placebo	0.80	-0.77 to 2.37	-4.14 to 5.69	0.37 [††]
Galantamine vs Placebo	0.47	-1.75 to 2.68	-4.64 to 5.66	0.25 [††]
Rivastigmine transdermal vs Placebo	2.38	-0.04 to 4.83	-2.87 to 7.56	0.75 [††]
Memantine vs Placebo	0.67	-1.27 to 2.58	-4.35 to 5.79	0.25 [††]
Donepezil + Memantine vs Placebo	2.67	0.18 to 5.16	-2.60 to 7.97	0.88 [††]
Galantamine + Memantine vs Placebo	2.43	-1.94 to 6.79	-3.94 to 8.81	0.75 [††]
Rivastigmine transdermal + Memantine vs Placebo	2.13	-1.40 to 5.63	-3.62 to 7.87	0.75 [††]
Placebo (reference)				0.12 [††]

Regression coefficient	0.02	-0.01 to 0.06		
Common within-network between-study variance: τ^2 = 5.40	3.63 to 8.29			
Design-by-treatment interaction model for inconsistency χ^2 *(d.f., P-value,* τ^2*)*: 4.36 (13, 0.987, 7.35)				
**Mean Difference: Meta-regression, Year of Publication**[**]				
Donepezil vs Placebo	1.53	0.51 to 2.54	-3.27 to 6.31	0.50[††]
Rivastigmine oral vs Placebo	0.66	-1.01 to 2.32	-4.31 to 5.65	0.25[††]
Galantamine vs Placebo	0.60	-1.65 to 2.85	-4.65 to 5.83	0.25[††]
Rivastigmine transdermal vs Placebo	2.59	0.09 to 5.12	-2.73 to 7.95	0.75[††]
Memantine vs Placebo	0.89	-1.05 to 2.80	-4.17 to 5.90	0.38[††]
Donepezil + Memantine vs Placebo	2.82	0.19 to 5.44	-2.57 to 8.21	0.88[††]
Galantamine + Memantine vs Placebo	2.59	-1.93 to 7.16	-3.98 to 9.12	0.75[††]
Rivastigmine transdermal + Memantine vs Placebo	2.21	-1.49 to 5.95	-3.81 to 8.24	0.75[††]
Placebo (reference)				0.12[††]
Regression coefficient	-0.02	-0.17 to 0.14		
Common within-network between-study variance: τ^2 = 5.53	3.71 to 8.48			
Design-by-treatment interaction model for inconsistency χ^2 *(d.f., P-value,* τ^2*)*: 4.36 (13, 0.987, 7.35)				
**Adverse Events (AEs)‡**				
**Odds Ratio: Aggregate data and crude results from studies with available individual patient data**				
Donepezil vs Placebo	1.07	0.86 to 1.32	0.68 to 1.67	0.31
Rivastigmine oral vs Placebo	1.26	0.83 to 1.90	0.70 to 2.24	0.16
Galantamine vs Placebo	0.95	0.75 to 1.21	0.60 to 1.51	0.52
Rivastigmine transdermal vs Placebo	0.87	0.57 to 1.35	0.48 to 1.58	0.61
Memantine vs Placebo	0.91	0.67 to 1.22	0.55 to 1.49	0.59
Donepezil + Memantine vs Placebo	0.76	0.34 to 1.68	0.31 to 1.88	0.69
Galantamine + Memantine vs Placebo	1.03	0.45 to 2.36	0.41 to 2.64	0.42
Rivastigmine transdermal + Memantine vs Placebo	0.69	0.32 to 1.51	0.28 to 1.70	0.77
Placebo (reference)				0.43
Common within-network between-study variance τ^2 = 0.04, I^2 = 20% (0%, 47%)				
Design-by-treatment interaction model for inconsistency χ^2 *(d.f., P-value,* τ^2*)*: 3.58 (6, 0.733, 0.05)				
**Odds Ratio: Aggregate data results**[**]				
Donepezil vs Placebo	1.09	0.89 to 1.33	0.88 to 1.35	0.25
Rivastigmine oral vs Placebo	1.43	0.92 to 2.21	0.90 to 2.26	0.07
Galantamine vs Placebo	0.88	0.63 to 1.25	0.62 to 1.27	0.54
Rivastigmine transdermal vs Placebo	0.79	0.44 to 1.41	0.43 to 1.45	0.61
Memantine vs Placebo	0.70	0.51 to 0.97	0.50 to 0.98	0.77
Donepezil + Memantine vs Placebo	0.77	0.39 to 1.54	0.37 to 1.60	0.64
Galantamine + Memantine vs Placebo	0.96	0.45 to 2.08	0.43 to 2.16	0.44
Rivastigmine transdermal + Memantine vs Placebo	0.62	0.28 to 1.40	0.27 to 1.46	0.80
Placebo (reference)				0.38
Common within-network between-study variance τ^2 = 0.00, I^2 = 0% (0%, 42%)				
Design-by-treatment interaction model for inconsistency χ^2 *(d.f., P-value,* τ^2*)*: 2.29 (4, 0.682, 0.01)				
**Odds Ratio: Crude results from studies with available individual patient data**				
Donepezil vs Placebo	0.95	0.50 to 1.78	0.33 to 2.70	0.57
Rivastigmine oral vs Placebo	0.81	0.37 to 1.75	0.25 to 2.61	0.71
Galantamine vs Placebo	1.05	0.71 to 1.56	0.44 to 2.50	0.46
Rivastigmine transdermal vs Placebo	0.92	0.38 to 2.20	0.26 to 3.31	0.57
Memantine vs Placebo	1.41	0.81 to 2.45	0.53 to 3.79	0.16
				0.53
Common within-network between-study variance τ^2 = 0.10, I^2 = 48% (0%, 76%)				
Design-by-treatment interaction model for inconsistency χ^2 *(d.f., P-value,* τ^2*)*: N/A (no closed loops)				
**Odds Ratio: Low Risk of Bias for Allocation Concealment***				
Donepezil vs Placebo	0.88	0.60 to 1.29	0.42 to 1.83	0.52
Rivastigmine oral vs Placebo	1.15	0.67 to 1.98	0.50 to 2.68	0.21
Galantamine vs Placebo	0.94	0.64 to 1.38	0.45 to 1.95	0.44
Rivastigmine transdermal vs Placebo	0.88	0.52 to 1.49	0.39 to 2.02	0.51
Memantine vs Placebo	0.86	0.55 to 1.36	0.40 to 1.88	0.54
Donepezil + Memantine vs Placebo	0.63	0.24 to 1.62	0.19 to 2.05	0.75
Rivastigmine transdermal + Memantine vs Placebo	0.67	0.25 to 1.80	0.20 to 2.28	0.71
Placebo (reference)				0.33
Common within-network between-study variance: τ^2 = 0.08, I^2 = 37% (0%, 64%)				
Design-by-treatment interaction model for inconsistency χ^2 *(d.f., P-value,* τ^2*)*: 2.19 (3, 0.53, 0.1)				
**Odds Ratio: Low Risk of Bias for Incomplete Data***				
Donepezil vs Placebo	0.83	0.53 to 1.29	0.45 to 1.51	0.51
Galantamine vs Placebo	0.69	0.50 to 0.97	0.42 to 1.13	0.80
Rivastigmine transdermal vs Placebo	0.79	0.42 to 1.49	0.36 to 1.76	0.56
Memantine vs Placebo	0.86	0.60 to 1.22	0.51 to 1.43	0.47
Placebo (reference)				0.16

Common within-network between-study variance: τ^2 = 0.02, I^2 = 10% (0%, 50%)				
Design-by-treatment interaction model for inconsistency χ^2 (d.f., P-value, τ^2): 0.00 (1, 0.95, 0.04)				
**Odds Ratio: Publicly-Sponsored Studies***				
Donepezil vs Placebo	2.15	0.36 to 12.69	--	0.16
Memantine vs Placebo	0.71	0.45 to 1.12	--	0.86
Donepezil + Memantine vs Placebo	1.53	0.23 to 10.18	--	0.46
Placebo (reference)				0.51
Common within-network between-study variance: τ^2 = N/A (each comparison includes a single study)				
Design-by-treatment interaction model for inconsistency χ^2 (d.f., P-value, τ^2): N/A (no closed loops)				
**Odds Ratio: Industry-Sponsored Studies***				
Donepezil vs Placebo	1.08	0.86 to 1.35	0.64 to 1.82	0.34
Rivastigmine oral vs Placebo	1.27	0.82 to 1.98	0.66 to 2.44	0.16
Galantamine vs Placebo	0.99	0.75 to 1.31	0.57 to 1.71	0.52
Rivastigmine transdermal vs Placebo	0.91	0.57 to 1.44	0.46 to 1.77	0.62
Memantine vs Placebo	0.95	0.65 to 1.37	0.52 to 1.73	0.58
Rivastigmine transdermal + Memantine vs Placebo	0.72	0.31 to 1.64	0.27 to 1.90	0.79
Placebo (reference)				0.50
Common within-network between-study variance: τ^2 = 0.05, I^2 = 25% (0%, 50%)				
Design-by-treatment interaction model for inconsistency χ^2 (d.f., P-value, τ^2): 3.68 (6, 0.72, 0.07)				
**Odds Ratio: Studies with Mild to Moderate cognitive impairment, assessed with MMSE at baseline ***				
Donepezil vs Placebo	1.27	0.88 to 1.83	0.61 to 2.65	0.29
Rivastigmine oral vs Placebo	1.36	0.83 to 2.24	0.60 to 3.09	0.25
Galantamine vs Placebo	1.01	0.67 to 1.55	0.47 to 2.19	0.56
Rivastigmine transdermal vs Placebo	1.02	0.50 to 2.05	0.39 to 2.69	0.55
Memantine vs Placebo	0.86	0.54 to 1.37	0.39 to 1.91	0.73
Galantamine + Memantine vs Placebo	1.10	0.40 to 3.00	0.32 to 3.78	0.48
Rivastigmine transdermal + Memantine vs Placebo	0.96	0.18 to 5.19	0.14 to 6.37	0.55
Placebo (reference)				0.59
Common within-network between-study variance: τ^2 = 0.09, I^2 = 29% (0%, 57%)				
Design-by-treatment interaction model for inconsistency χ^2 (d.f., P-value, τ^2): 3.29 (5, 0.66, 0.13)				
**Odds Ratio: Studies with Moderate to Severe cognitive impairment, assessed with MMSE at baseline ***				
Donepezil vs Placebo	0.92	0.67 to 1.27	0.59 to 1.45	0.38
Galantamine vs Placebo	0.70	0.46 to 1.07	0.38 to 1.28	0.76
Memantine vs Placebo	0.95	0.55 to 1.62	0.44 to 2.02	0.36
Donepezil + Memantine vs Placebo	0.66	0.32 to 1.37	0.23 to 1.86	0.76
Placebo (reference)				0.23
Common within-network between-study variance: τ^2 = 0.00, I^2 = 0% (0%, 72%)				
Design-by-treatment interaction model for inconsistency χ^2 (d.f., P-value, τ^2): 2.90 (1, 0.09, 0.00)				
**Odds Ratio: NMA of studies with IPD – available case analysis**				
Donepezil vs Placebo	1.63	0.49 to 5.41	0.30 to 8.73	0.33
Rivastigmine oral vs Placebo	1.28	0.08 to 19.94	0.04 to 39.11	0.46
Galantamine vs Placebo	1.05	0.67 to 1.63	0.38 to 2.85	0.58
Rivastigmine transdermal vs Placebo	0.81	0.02 to 35.04	0.01 to 82.49	0.59
Memantine vs Placebo	1.35	0.72 to 2.55	0.43 to 4.24	0.38
Placebo (reference)				0.64
Common within-network between-study variance: τ^2 = 0.13, I^2 = 50% (0%, 77%)				
Design-by-treatment interaction model for inconsistency χ^2 (d.f., P-value, heterogeneity): N/A (no closed loops)				
**Odds Ratio: Meta-regression, Trial Mean Age****				
Donepezil vs Placebo	1.13	0.88 to 1.43	0.68 to 1.86	0.25 ††
Rivastigmine oral vs Placebo	1.52	0.89 to 2.53	0.77 to 3.04	0.00 ††
Galantamine vs Placebo	0.91	0.60 to 1.30	0.52 to 1.59	0.50 ††
Rivastigmine transdermal vs Placebo	0.84	0.39 to 1.58	0.34 to 1.80	0.75 ††
Memantine vs Placebo	0.74	0.48 to 1.07	0.39 to 1.26	0.75 ††
Donepezil + Memantine vs Placebo	0.92	0.38 to 1.89	0.33 to 2.15	0.62 ††
Galantamine + Memantine vs Placebo	0.99	0.37 to 2.27	0.33 to 2.55	0.50 ††
Rivastigmine transdermal + Memantine vs Placebo	0.73	0.24 to 1.70	0.22 to 1.87	0.87 ††
Placebo (reference)				0.37 ††
Regression coefficient (log-scale)	-0.03	-0.08 to 0.02		
Common within-network between-study variance: τ^2 = 0.02	0.00 to 0.19			
Design-by-treatment interaction model for inconsistency χ^2 (d.f., P-value, τ^2): 3.57 (6, 0.735, 0.06)				
**Odds Ratio: NMA of studies with IPD adjusted for Age**				
Donepezil vs Placebo	0.95	0.50 to 1.78	0.33 to 2.73	0.57
Rivastigmine oral vs Placebo	0.84	0.39 to 1.81	0.26 to 2.74	0.68
Galantamine vs Placebo	1.04	0.70 to 1.55	0.43 to 2.52	0.46
Rivastigmine transdermal vs Placebo	0.91	0.38 to 2.17	0.25 to 3.28	0.58
Memantine vs Placebo	1.39	0.80 to 2.44	0.52 to 3.79	0.17
Placebo (reference)				0.53
Common within-network between-study variance: τ^2 = 0.10, I^2 = 48% (0%, 76%)				
Design-by-treatment interaction model for inconsistency χ^2 (d.f., P-value, τ^2): N/A (no closed loops)				

**Odds Ratio: Meta-regression, Percent of Male Participants**[**]				
Donepezil vs Placebo	1.12	0.87 to 1.44	0.64 to 2.01	0.25 [††]
Rivastigmine oral vs Placebo	1.71	0.97 to 2.92	0.83 to 3.67	0.00 [††]
Galantamine vs Placebo	0.93	0.62 to 1.36	0.49 to 1.77	0.50 [††]
Rivastigmine transdermal vs Placebo	0.89	0.39 to 1.79	0.34 to 2.05	0.63 [††]
Memantine vs Placebo	0.64	0.37 to 1.00	0.29 to 1.21	0.88 [††]
Donepezil + Memantine vs Placebo	0.88	0.35 to 1.88	0.30 to 2.13	0.63 [††]
Galantamine + Memantine vs Placebo	1.13	0.39 to 2.58	0.36 to 2.95	0.38 [††]
Rivastigmine transdermal + Memantine vs Placebo	0.77	0.24 to 1.93	0.21 to 2.13	0.88 [††]
Placebo (reference)				0.38 [††]
Regression coefficient (log-scale)	0.00	0.00 to 0.02		
Common within-network between-study variance: τ^2 = 0.03	0.00 to 0.23			
Design-by-treatment interaction model for inconsistency χ^2 (d.f., P-value, τ^2): 3.57 (6, 0.735, 0.06)				
**Odds Ratio: NMA of studies with IPD adjusted for Percent of Male Participants**				
Donepezil vs Placebo	1.04	0.54 to 1.99	0.34 to 3.16	0.49
Rivastigmine oral vs Placebo	0.81	0.37 to 1.80	0.24 to 2.79	0.72
Galantamine vs Placebo	1.05	0.70 to 1.59	0.42 to 2.65	0.48
Rivastigmine transdermal vs Placebo	0.92	0.37 to 2.27	0.24 to 3.52	0.58
Memantine vs Placebo	1.40	0.80 to 2.48	0.50 to 3.98	0.19
Placebo (reference)				0.55
Common within-network between-study variance: τ^2 = 0.11, I^2 = 51% (0%, 77%)				
Design-by-treatment interaction model for inconsistency χ^2 (d.f., P-value, τ^2): N/A (no closed loops)				
**Odds Ratio: NMA of studies with IPD adjusted for cognitive impairment, assessed with MMSE at baseline**				
Donepezil vs Placebo	0.97	0.46 to 2.06	0.23 to 4.03	0.56
Rivastigmine oral vs Placebo	0.81	0.33 to 2.01	0.17 to 3.91	0.70
Galantamine vs Placebo	1.29	0.74 to 2.25	0.37 to 4.55	0.28
Rivastigmine transdermal vs Placebo	0.93	0.34 to 2.53	0.18 to 4.91	0.57
Memantine vs Placebo	1.26	0.59 to 2.70	0.30 to 5.28	0.33
Placebo (reference)				0.56
Common within-network between-study variance: τ^2 = 0.16, I^2 = 52% (0%, 80%)				
Design-by-treatment interaction model for inconsistency χ^2 (d.f., P-value, τ^2): N/A (no closed loops)				
**Odds Ratio: NMA of studies with IPD adjusted for comorbidities**				
Donepezil vs Placebo	1.01	0.52 to 1.96	0.29 to 3.50	0.51
Rivastigmine oral vs Placebo	0.82	0.36 to 1.87	0.20 to 3.32	0.69
Galantamine vs Placebo	1.02	0.57 to 1.80	0.32 to 3.26	0.50
Rivastigmine transdermal vs Placebo	0.91	0.36 to 2.31	0.20 to 4.11	0.58
Memantine vs Placebo	1.42	0.79 to 2.55	0.44 to 4.59	0.18
Placebo (reference)				0.53
Common within-network between-study variance: τ^2 = 0.12, I^2 = 44% (0%, 77%)				
Design-by-treatment interaction model for inconsistency χ^2 (d.f., P-value, τ^2): N/A (no closed loops)				
**Odds Ratio: NMA of studies with IPD adjusted for other medications**				
Donepezil vs Placebo	1.17	0.49 to 3.03	0.28 to 4.88	0.41
Rivastigmine oral vs Placebo	0.82	0.37 to 1.81	0.23 to 2.91	0.72
Galantamine vs Placebo	1.03	0.69 to 1.55	0.40 to 2.65	0.51
Rivastigmine transdermal vs Placebo	0.95	0.39 to 2.34	0.24 to 2.91	0.56
Memantine vs Placebo	1.34	0.75 to 2.39	0.46 to 3.92	0.25
Placebo (reference)				0.56
Common within-network between-study variance: τ^2 = 0.11, I^2 = 51% (0%, 78%)				
Design-by-treatment interaction model for inconsistency χ^2 (d.f., P-value, τ^2): N/A (no closed loops)				
**Odds Ratio: Meta-regression, Study Duration**[**]				
Donepezil vs Placebo	1.12	0.87 to 1.43	0.63 to 1.95	0.25 [††]
Rivastigmine oral vs Placebo	1.76	1.00 to 2.99	0.88 to 3.68	0.00 [††]
Galantamine vs Placebo	0.92	0.62 to 1.36	0.50 to 1.69	0.50 [††]
Rivastigmine transdermal vs Placebo	0.87	0.39 to 1.70	0.34 to 1.96	0.63 [††]
Memantine vs Placebo	0.61	0.37 to 0.93	0.31 to 1.13	0.88 [††]
Donepezil + Memantine vs Placebo	0.76	0.29 to 1.69	0.26 to 1.90	0.75 [††]
Galantamine + Memantine vs Placebo	0.98	0.34 to 2.26	0.30 to 2.53	0.50 [††]
Rivastigmine transdermal + Memantine vs Placebo	0.75	0.25 to 1.81	0.23 to 1.97	0.75 [††]
Placebo (reference)				0.38 [††]
Regression coefficient (log-scale)	0.00	0.00 to 0.01		
Common within-network between-study variance: τ^2 = 0.03	0.00 to 0.22			
Design-by-treatment interaction model for inconsistency χ^2 (d.f., P-value, τ^2): 3.57 (6, 0.735, 0.06)				
**Odds Ratio: Meta-regression, Year of Publication**[**]				
Donepezil vs Placebo	1.05	0.79 to 1.38	0.61 to 1.77	0.38[††]
Rivastigmine oral vs Placebo	1.68	0.98 to 2.77	0.85 to 3.37	0.00 [††]
Galantamine vs Placebo	0.91	0.61 to 1.32	0.50 to 1.64	0.63 [††]
Rivastigmine transdermal vs Placebo	0.92	0.40 to 1.84	0.36 to 2.04	0.63 [††]
Memantine vs Placebo	0.73	0.46 to 1.05	0.38 to 1.28	0.88 [††]
Donepezil + Memantine vs Placebo	0.88	0.35 to 1.83	0.31 to 2.15	0.75 [††]

Galantamine + Memantine vs Placebo	1.24	0.43 to 2.85	0.39 to 3.25	0.25 [††]
Rivastigmine transdermal + Memantine vs Placebo	0.88	0.24 to 2.24	0.24 to 2.42	0.75 [††]
Placebo (reference)				0.38 [††]
Regression coefficient (log-scale)	-0.02	-0.06 to 0.03		
Common within-network between-study variance: τ^2 = 0.02	0.00 to 0.21			
Design-by-treatment interaction model for inconsistency χ^2 (d.f., P-value, τ^2): 3.57 (6, 0.735, 0.06)				

* Aggregate data and fully adjusted results from studies with available individual patient data
† MMSE: Studies with available IPD included only available participants – to assess the missing data impact on the second stage a separate analysis was applied (IMDoM)
‡ AE: Studies with available IPD included all randomized participants
§ Outlier studies:

- Hernandez C, Unturbe F, Martinez-Lage P, Lucas A, Gregorio P, Alonso T. Effects of combined pharmacologic and cognitive treatment in the progression of moderate dementia: a two-year follow-up. REVISTA ESPANOLA DE GERIATRIA Y GERONTOLOGIA. 2007;42(1):3
- Moretti DV. Alpha rhythm oscillations and MMSE scores are differently modified by transdermal or oral rivastigmine in patients with Alzheimer's disease. American journal of neurodegenerative disease. 2014;3(2):72-83.

¶ Included studies with available raw data only, irrespective having access to individual patient data
‖ Analyses were conducted in Stata using the *metamiss2* and *network* commands; I2 is not available; SUCRA values are presented instead of P-scores
** Studies with aggregate data were used (studies with available individual patient data were not included in this analysis)
†† Analyses were conducted in OpenBUGS, and SUCRA values were calculated instead of P-scores

Veroniki AA, *et al. BMJ Open* 2022; 12:e053012. doi: 10.1136/bmjopen-2021-053012

Appendix 17: CINeMA results

Risk of bias contributions: The bar chart shows the contributions of each piece of study to the network estimate

Interventions
A: PLAC
B: DONE
C: RIVA_O
D: GALA
E: RIVA_P
F: MEMA
G: DONE+MEMA
H: GALA+MEMA
I: RIVA_P+MEMA

MMSE outcome

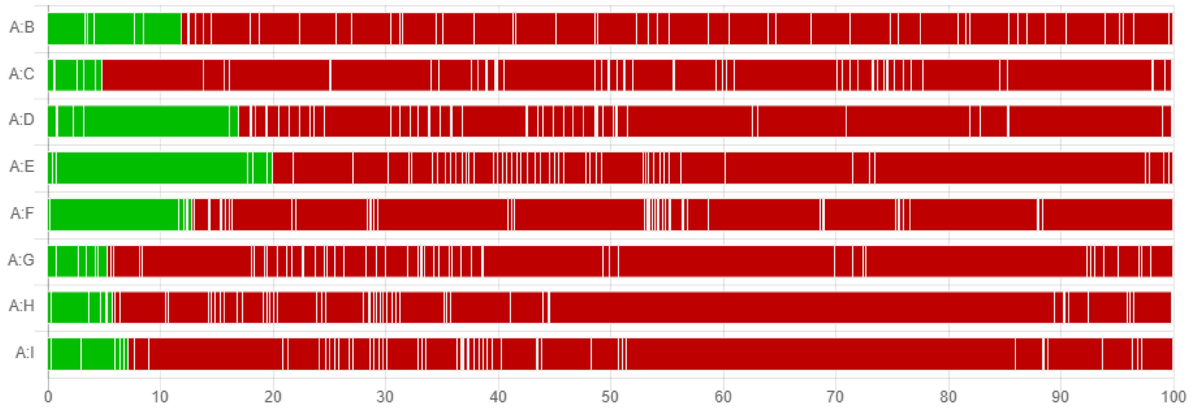

AE outcome

Veroniki AA, *et al. BMJ Open* 2022; 12:e053012. doi: 10.1136/bmjopen-2021-053012

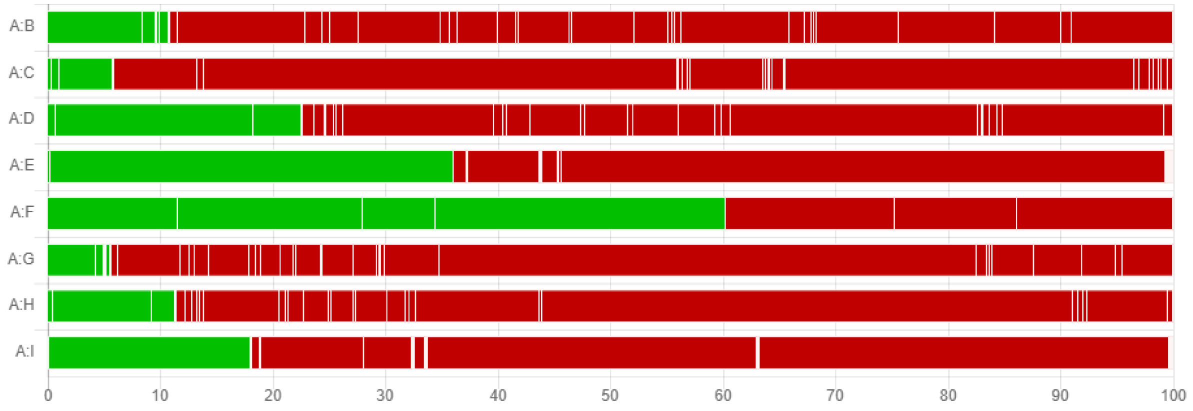

CINeMA report

MMSE outcome

Comparison	# of studies	Nature of evidence	Type of data	Within-study bias (D1)	Reporting bias (D2)	Indirectness (D3)	Imprecision (D4)	Heterogeneity (D5)	Incoherence (D6)	Confidence rating	Downgrading due to
DONE vs PLAC	24	Mixed	IPD+AD	Major concerns	Suspected	No concerns	No concerns	Major concerns	No concerns	Moderate	D5
RIVA_O vs PLAC	6	Mixed	IPD+AD	Major concerns	Suspected	No concerns	Some concerns	Some concerns	No concerns	Moderate	D4;D5
GALA vs PLAC	3	Mixed	IPD+AD	Major concerns	Suspected	No concerns	Major concerns	No concerns	No concerns	Moderate	D4
RIVA_P vs PLAC	2	Mixed	IPD+AD	Major concerns	Suspected	No concerns	Some concerns	Some concerns	No concerns	Moderate	D4;D5
MEMA vs PLAC	7	Mixed	IPD+AD	Major concerns	Suspected	No concerns	Some concerns	Some concerns	No concerns	Moderate	D4;D5
DONE+MEMA vs PLAC	1	Mixed	AD	Major concerns	Suspected	No concerns	No concerns	Major concerns	No concerns	Moderate	D5
GALA+MEMA vs PLAC	0	Indirect	-	Major concerns	Suspected	No concerns	Major concerns	No concerns	No concerns	Moderate	D4
RIVA_P+MEMA vs PLAC	0	Indirect	-	Major concerns	Suspected	No concerns	Major concerns	No concerns	No concerns	Moderate	D4

AE outcome

Comparison	# of studies	Nature of evidence	Type of data	Within-study bias (D1)	Reporting bias (D2)	Indirectness (D3)	Imprecision (D4)	Heterogeneity (D5)	Incoherence (D6)	Confidence rating	Downgrading due to
DONE vs PLAC	16	Mixed	IPD+AD	Major concerns	Undetected	No concerns	Major concerns	No concerns	No concerns	Moderate	D1
RIVA_O vs PLAC	3	Mixed	IPD+AD	Major concerns	Undetected	No concerns	Major concerns	No concerns	No concerns	Moderate	D1
GALA vs PLAC	8	Mixed	IPD+AD	Major concerns	Undetected	No concerns	Major concerns	No concerns	No concerns	Moderate	D1
RIVA_P vs PLAC	2	Mixed	IPD+AD	Some concerns	Undetected	No concerns	Major concerns	No concerns	No concerns	High	
MEMA vs PLAC	7	Mixed	IPD+AD	Some concerns	Undetected	No concerns	Major concerns	No concerns	No concerns	High	
DONE+MEMA vs PLAC	2	Mixed	AD	Major concerns	Undetected	No concerns	Major concerns	No concerns	No concerns	Moderate	D1
GALA+MEMA vs PLAC	0	Indirect	-	Major concerns	Undetected	No concerns	Major concerns	No concerns	No concerns	Moderate	D1
RIVA_P+MEMA vs PLAC	0	Indirect	-	Major concerns	Undetected	No concerns	Major concerns	No concerns	No concerns	Moderate	D1

Abbreviations: DONE, donepezil; GALA, galantamine; MEMA, memantine; PLAC, placebo; RIVA_O, rivastigmine oral; RIVA_P, rivastigmine patch

Veroniki AA, *et al. BMJ Open* 2022; 12:e053012. doi: 10.1136/bmjopen-2021-053012

Appendix 18: Study definitions for adverse events

Author, Year	Source of Definition	Definition
Agid, 1998	Determined by Investigator	"Patients and caregivers were questioned systematically regarding the occurrence of adverse events at each clinical visit"
Ancoli-Israel, 2005	Determined by Investigator	"Only one serious AE leading to discontinuation, hepatic failure, in the donepezil-treated group was considered to be possibly due to study treatment by the investigator."
Andersen, 2012	NA	NA
Araki, 2014	NA	NA
Bakchine, 2008	Determined by Investigator	" A patient could also be withdrawn from the study if: they had a serious adverse event (SAE: death, life-threatening condition, hospitalisation) [..] Three patients had an SAE that was considered by the investigator to be possibly or probably related to treatment."
Black, 2007	Determined by Investigator	"AEs were considered serious (SAEs) when death occurred, life was threatened, hospitalization or prolonged hospitalization was required, or a significant disability occurred."
Blesa González, 2011	NA	NA
Burns,1999	COSTART	"Serious adverse events (SAE) included fatal or life-threatening situations, permanently disabling conditions or incidents that required or prolonged hospitalisation […] Events were coded using a modified COSTART dictionary, and the assessment of relationship to treatment for all adverse events was conducted blind to treatment assignment."
Burns, 2009	NR	NR
Burns, 2011	NR	NR
Choi, 2011	Determined by Investigator	"Investigators were asked to evaluate severity (mild, moderate, or severe), relationship to study drug (not related, probable relationship with rivastigmine patch, probable relationship with memantine, or probable relationship with an interaction of the two drugs), and seriousness of the AEs."
Corey-Bloom, 1998	NA	NA
Cretu, 2008	NA	NA
Dysken, 2014	Medical Dictionary for Regulatory Activities	"Serious AEs were coded according to the Medical Dictionary for Regulatory Activities."
Farlow, 2013	NA	NA
Feldman, 2001	Determined by Investigator	"Serious AE was defined as any AE that was life threatening or resulted in death, hospitalization, prolongation of hospitalization, or significant disability."
Feldman, 2007	World Health Organisation preferred terms	" A similar proportion of patients in each treatment group experienced at least one serious adverse event (any event that was fatal, considered life threatening or required hospitalisation) […] All adverse events were recorded using the Novartis Medical Terminology Thesaurus (a modified version of the WHO adverse reaction terminology dictionary)."
Fox, 2012	NA	NA
Frolich, 2011	NA	NA
Fuschillo, 2001	NA	NA
Gault L, 2015	Medical Dictionary for Regulatory Activities	"AEs were coded using the Medical Dictionary for Regulatory Activities"
Gold, 2010	NR	"SAE (fatal or nonfatal) "
Greenberg, 2000	Determined by Investigator	"Of 9 withdrawals from the study after randomization, 2 were due to serious adverse events judged to be possibly related to donepezil therapy: syncope and generalized seizure (1 patient each). "
Grossberg, 2013	Medical Dictionary for Regulatory Activities	"Adverse events were coded according to the Medical Dictionary for Regulatory Activities (version 7.0 or newer), and an assessment of the severity, chronicity, causal relationship to study medication, and seriousness of the event was provided by an investigator"
Hager, 2014	Determined by Investigator	"Safety data were monitored during the study by a company-commissioned, external, independent, blinded Data Safety Monitoring Board (DSMB). Secondary safety outcomes were the number of treatment emergent adverse events (TEAEs), including serious TEAEs."
Haig, 2014	Determined by Investigator	"The incidence of adverse events considered possibly or probably related to study drug as assessed by the investigator was generally similar across treatment groups (range 20.6% to 26.8%)." "Treatment emergent adverse events were tabulated by primary Medical Dictionary for Regulatory Activities (MedDRA) [23] version 13.1 System Organ Class and Preferred Term"
Hernández, 2007	NA	NA
Herrmann, 2013	Determined by Investigator	"The incidence of adverse events considered related to the study drug by the investigator was 30% in the placebo group and 36% in the memantine group"
Holmes, 2004	Determined by Investigator	"During these (clinic) visits, psychometric evaluations, medication compliance checks, and adverse event (AE) monitoring took place"

Homma, 1998	NR	NR
Homma, 2008	Medical Dictionary for Regulatory Activities – Japanese Version	"AE terms were standardized according to the Medical Dictionary for Regulatory Activities – Japanese Version . AEs were graded on a 3-point scale (mild: discomfort noticed, but no disruption of normal daily activity; moderate: discomfort sufficient to reduce or affect normal daily activity; severe: incapacitating, with inability to work or to perform normal daily activity). "
Hong, 2006	NR	NR
Howard, 2007	NA	NA
Howard, 2012	NR	NR
Hu, 2006	NA	NA
Johannsen, 2006	NA	NA
Jones, 2004	Determined by Investigator	"A serious adverse event (SAE) was defined as any AE that was life threatening or resulted in death, hospitalisation, prolongation of hospitalisation, or significant disability"
Kadir, 2008	NA	NA
Kano, 2013	NA	NA
Karaman, 2005	NA	NA
Likitjaroen, 2012	NA	NA
Lorenzi, 2011	NA	NA
Maher-Edwards, 2011	Determined by Investigator	"Eight subjects experienced nonfatal serious AEs; all were considered unrelated to the study drug"
Marek, 2014	Medical Dictionary for Regulatory Activities	"Aes were coded using the Medical Dictionary for Regulatory Activities (MedDRA, version 14.0) by system organ class and preferred term"
Mazza, 2006	NA	NA
Mohs, 2001	Determined by Investigator	"In all cases, judgment of the relationship of study treatment to an adverse event and of the severity of the event was made by the investigator under double-blind conditions. "
Moretti, 2014	NA	NA
Mowla, 2007	NA	NA
Nakamura, 2011	Determined by Investigator	"Safety evaluations included recording all adverse events on Adverse Event Case Report Forms. Every serious adverse event occurring after the patient provided informed consent and until 28 days after the patient stopped the study was reported. "
Nakano, 2001	NA	NA
Nordberg, 2009	Determined by Investigator	"Safety and tolerability were monitored throughout the study by recording all adverse events (AEs). "
Pakdaman H, 2015	NA	NA
Peng, 2005	NA	NA
Peskind, 2006	Determined by Investigator	"Overall, the type and incidence of SAEs were similar between the memantine and placebo groups. One participant death occurred in each group during the trial; neither was rated by the investigator as being treatment-related"
Peters O, 2015	NR	NR
Reisberg, 2003	NR	NR
Rockwood, 2001	World Health Organisation preferred terms	"adverse events (classified according to World Health Organisation preferred terms)."
Rockwood, 2006	NR	NR
Rogers, 1996		
Rogers, 1998	COSTART	"Events, recorded using investigator terminology, were grouped and coded into common terms using a modified COSTART dictionary"
Rogers, 1998	COSTART	"Events, recorded using investigator terminology, were grouped and coded into common terms using a modified COSTART dictionary. "
Saxton, 2012	Determined by Investigator	"Treatment-emergent adverse events (TEAEs) and serious adverse events (SAEs) were recorded at all post-Screening study visits"
Scarpini, 2011	Determined by Investigator	"Subjects with a treatment 51 (20.1) 2 (2.6) 4 (6.3) related AE, as judged by the investigator"
Schmidt, 2008	NA	NA
Seltzer, 2004	NA	NA
Shao, 2015	NA	NA
Shimizu, 2015	NA	NA
Sole-Padulles, 2013	NA	NA
Tariot, 2000	World Health Organisation preferred terms	"adverse events (classified according to World Health Organization Preferred Term). "
Tariot, 2001	COSTART	"Investigator terms describing AEs were coded to standard preferred terms using a modified Coding Symbols for Thesaurus of Adverse Reaction Terms dictionary. "
Thomas, 2001		

Wilcock, 2003	World Health Organisation preferred terms	"monitoring for adverse events (classified according to WHO preferred terms) "
Wilkinson, 2001	Determined by Investigator	"All adverse events were recorded, regardless of the considered relationship to treatment. All details of adverse events and their outcomes were recorded including severity and relationship to treatment. Serious adverse events were documented separately. "
Wilkinson, 2002	NR	NR
Wilkinson, 2012	Determined by Investigator	"Tolerability and safety were based on the incidence of adverse events, either reported spontaneously by the patients or in response to a non-leading question by the investigator throughout the study"
Winblad, 2001	NR	NR
Winblad, 2006	COSTART	"We recorded all treatment emergent adverse events, coding them according to a modified COSTART dictionary. "
Winblad, 2007	Determined by Investigator	"Safety evaluations included recording all adverse events, which were coded using a standard glossary."
Zhang-Yi, 2005	NA	NA
Zhang, 2012	Determined by Investigator	"Serious adverse events considered to be possibly related to treatment occurred in one patient in each treatment arm"

Notes: [a]Unpublished data, [b]Non-English studies
Abbreviations: CR, companion report; NA, not applicable; NR, not reported.

Veroniki AA, *et al. BMJ Open* 2022; 12:e053012. doi: 10.1136/bmjopen-2021-053012

Appendix 19: Time taken to achieve at least an adverse event using individual patient data

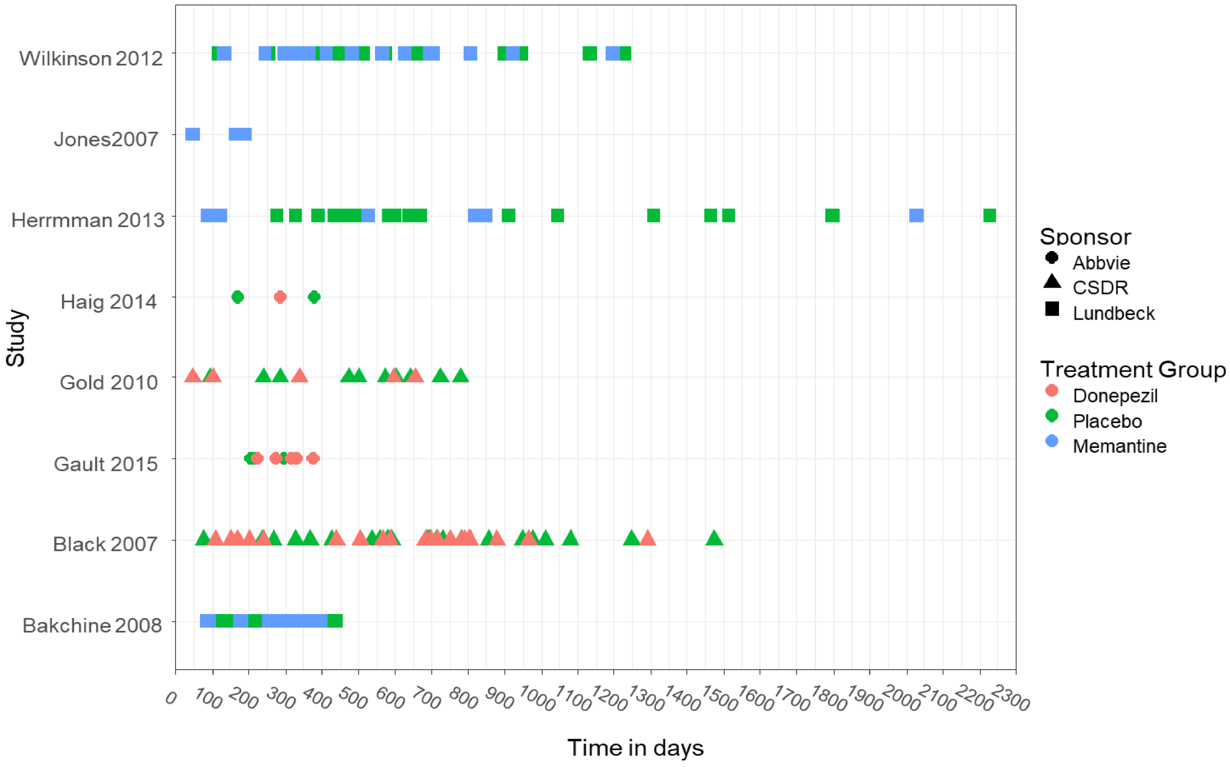

Veroniki AA, *et al. BMJ Open* 2022; 12:e053012. doi: 10.1136/bmjopen-2021-053012

Appendix 20: Rank-heat plot for adverse events

Circles from inside out present results for different network meta-analyses including: i) aggregate data (AD) only (studies with available IPD are not included in the analysis), ii) crude results from individual studies with individual patient data (IPD), iii) AD and crude results from studies with available IPD, and iv) AD and fully adjusted results from studies with available IPD. Numbers within each sector correspond to the P-score values as calculated in each model.

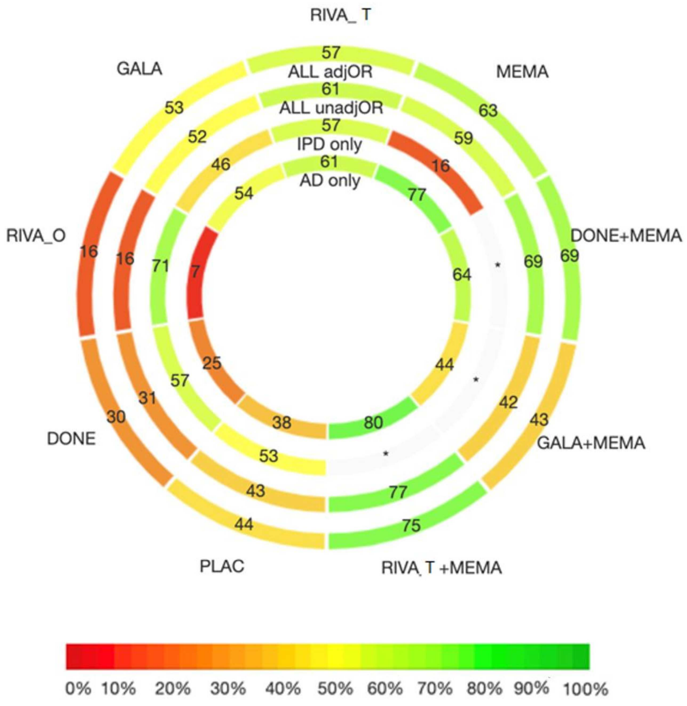

Veroniki AA, *et al. BMJ Open* 2022; 12:e053012. doi: 10.1136/bmjopen-2021-053012

Appendix 21: Challenges encountered during the individual patient data request from sponsors

- The identification of the trial data set when certain details were not available (e.g. NCT number; particularly for studies published before 2005 that this was established).
- Data ownership.
- Sponsors switched platforms, while we were navigating the data.
- IPD available through proprietary sponsor-specific platforms did not allow for combination of IPD from different sponsor platforms; hence a one-stage analysis as planned in our protocol, was impossible.
- Software availability: Required R packages (e.g., mice) were not available/provided, and we were not allowed to install any new R packages; some R packages were older versions (e.g. lme4).
- Time that the platform permitted access to the IPD was often limited. This is a significant constraint given that IPD from different studies became available at different time points.
- Cost associated with obtaining access to the data for a certain amount of time. Additionally, cost associated with the WHO Drug Dictionary license to obtain access to the additional medications used for each patient; this license's approximate cost was $8,958·25 USD per sponsor.
- Available IPD did not include the full information as shown in the publication: For example, only data for placebo were available, or did not give information about a reported outcome (e.g. only baseline MMSE values were available). Also, date of follow-up was coded in some studies and it was impossible to make a judgement on first and last date.

Veroniki AA, *et al. BMJ Open* 2022; 12:e053012. doi: 10.1136/bmjopen-2021-053012
