# Supplementary material for: Comparative safety and efficacy of cognitive enhancers for Alzheimer’s dementia: a systematic review with individual patient data network meta-analysis
Source: BMJ Open. 2022 Apr 25;12(4):e053012. doi: 10.1136/bmjopen-2021-053012 (PMC9045061; doi:10.1136/bmjopen-2021-053012)
Supplement: Supplementary data [file bmjopen-2021-053012supp002.pdf]

Additional File 2: MEDLINE Search Strategy

MEDLINE Search

Database: Ovid MEDLINE(R) In-Process & Other Non-Indexed Citations and Ovid MEDLINE(R) <1946 to Present>, Embase<1980 to 2014 Week 50> Search Strategy:
----
1 alzheimer$.mp.
2 "benign senescent forgetfulness".mp.
3 (cognit$ adj2 (impair$ or declin$ or deficit$ or degenerat$ or deteriorat$ or los$ or disorder$ or complain$ or disturb$)).mp.
4 (cerebr$ adj2 (impair$ or declin$ or deficit$ or degenerat$ or deteriorat$ or los$ or disorder$ or complain$ or disturb$)).mp.
5 (mental adj2 (impair$ or declin$ or deficit$ or degenerat$ or deteriorat$ or los$ or disorder$ or complain$ or disturb$)).mp.
6 (ne?rocognit$ adj2 (impair$ or declin$ or deficit$ or degenerat$ or deteriorat$ or los$ or disorder$ or complain$ or disturb$)).mp.)
7 (ne?ro-cognit$ adj2 (impair$ or declin$ or deficit$ or degenerat$ or deteriorat$ or los$ or disorder$ or complain$ or disturb$)).mp.
8 ((cognit$ or memory or cerebral or brain) adj2 (improv$ or enhanc$ or perform$ or process$ or function$ or rehabilitation or aid$ or stimulat$)).mp.
9 cognition.ti.
10 (confusion$ or confused).tw.
11 dement$.mp.
12 ("normal pressure hydrocephalus" and shunt$).mp.
13 "organic brain disease$".mp.
14 "organic brain syndrome".mp.
15 (presenil$ or pre-senil$ or senil$).tw.
16 Alzheimer Disease/
17 Cognition/de
18 Confusion/
19 Dementia/
20 or/1-19
21 abixa.tw.
22 aricept.tw.
23 (acetylcholinesteraseadj inhibitor$).tw.
24 axura.tw.
25 akatinol.tw.
26 (anticholinesterase? or anti-cholinesterase?).tw.
27 (cognitive adjenhanc$).mp.
28 (cholinesterase adj inhibitor$).mp.
29 ChEI.tw.
30 donepezil.mp.
31 ebixa.tw.
32 eranz.tw.
33 exelon.tw.
34 galant?amin$.tw.
35 lycoremine.tw.

Veroniki AA, *et al. BMJ Open* 2022; 12:e053012. doi: 10.1136/bmjopen-2021-053012

36 memantin$.tw.
37 memox.tw.
38 namenda.tw.
39 nimvastid.tw.
40 nivalin$.tw.
41 "N-Methyl-D-aspartic acid receptor antagonist$".tw.
42 prometax.tw.
43 razadyne.tw.
44 reminyl.tw.
45 rivastigmine.mp.
46 exp Cholinesterase Inhibitors/
47 Galantamine/
48 Memantine/
49 Galantamin.rn.
50 Memantine.rn.
51 Donepezil.rn.
52 Donepezil Hydrochloride.rn.
53 Rivastigmine.rn.
54 or/21-53
55 20 and 54
56 exp Animals/ not (exp Animals/ and Humans/)
57 55 and 56
58 (comment or editorial or interview or news).pt.
59 (letter not (letter and randomized controlled trial)).pt.
60 57 not (58 or 59)
61 (201111* or 201112* or 2012* or 2013* or 2014*).ed.
62 60 and 61
63 alzheimer$.mp.
64 "benign senescent forgetfulness".mp.
65 (cognit$ adj2 (impair$ or declin$ or deficit$ or degenerat$ or deteriorat$ or los$ or disorder$ or complain$ or disturb$)).mp.
66 (cerebr$ adj2 (impair$ or declin$ or deficit$ or degenerat$ or deteriorat$ or los$ or disorder$ or complain$ or disturb$)).mp.
67 (mental adj2 (impair$ or declin$ or deficit$ or degenerat$ or deteriorat$ or los$ or disorder$ or complain$ or disturb$)).mp.
68 (ne?rocognit$ adj2 (impair$ or declin$ or deficit$ or degenerat$ or deteriorat$ or los$ or disorder$ or complain$ or disturb$)).mp.
69 (ne?ro-cognit$ adj2 (impair$ or declin$ or deficit$ or degenerat$ or deteriorat$ or los$ or disorder$ or complain$ or disturb$)).mp.
70 ((cognit$ or memory or cerebral or brain) adj2 (improv$ or enhanc$ or perform$ or process$ or function$ or rehabilitation or aid$ or stimulat$)).mp.
71 cognition.ti.
72 (confusion$ or confused).tw.
73 dement$.mp.
74 ("normal pressure hydrocephalus" and shunt$).mp.
75 "organic brain disease$".mp.
76 "organic brain syndrome".mp.

77 (presenil$ or pre-senil$ or senil$).tw
78 Alzheimer disease/
79 cognitive defect/
80 confusion/
81 dementia/
82 organic brain syndrome/
83 or/63-82
84 abixa.tw.
85 aricept.tw.
86 (acetylcholinesteraseadj inhibitor$).tw.
87 axura.tw.
88 akatinol.tw.
89 (anticholinesterase? or anti-cholinesterase?).tw.
90 (cognitive adjenhanc$).mp.
91 (cholinesterase adj inhibitor$).mp.
92 ChEI.tw.
93 donepezil.mp.
94 ebixa.tw.
95 eranz.tw.
96 exelon.tw.
97 galant?amin$.tw.
98 lycoremine.tw.
99 memantin$.tw.
100 memox.tw.
101 namenda.tw.
102 nimvastid.tw.
103 nivalin$.tw.
104 "N-Methyl-D-aspartic acid receptor antagonist$".tw.
105 prometax.tw.
106 razadyne.tw.
107 reminyl.tw.
108 rivastigmine.mp.
109 exp cholinesterase inhibitor/
110 donepezil/ or donepezil plus memantine/
111 galantamine/
112 memantine/
113 rivastigmine/
114 357-70-0.rn.
115 19982-08-2.rn.
116 120011-70-3.rn.
117 120014-06-4.rn.
118 rivastigmine.rn.
119 or/84-118
120 83 and 119
121 randomized controlled trial/ or controlled clinical trial/
122 exp "clinical trial (topic)"/
123 (randomi#ed or randomly or RCT$1 or placebo*).tw.

124 ((singl* or doubl* or trebl* or tripl*) adj (mask* or blind* or dumm*)).tw.
125 trial.ti.
126 or/121-125
127 120 and 126
128 exp controlled clinical trial/
129 exp "controlled clinical trial (topic)"/
130 (control* adj2 trial*).tw.
131 (nonrandom* or non-random* or quasi-random* or quasi-experiment*).tw.
132 (nRCT or nRCTs or non-RCT$1).tw.
133 (control* adj3 ("before and after" or "before after")).tw.
134 time series analysis/
135 (time series adj3 interrupt*).tw.
136 pretest posttest control group design/
137 (pre- adj3 post-).tw.
138 (pretest adj3 posttest).tw.
139 controlled study/
140 (control* adj2 stud$3).tw.
141 control group/
142 (control$ adj2 group$1).tw.
143 or/128-142
144 120 and 143
145 cohort analysis/
146 cohort.tw.
147 retrospective study/
148 longitudinal study/
149 prospective study/
150 (longitudinal or prospective or retrospective).tw.
151 follow up/
152 ((followup or follow-up) adj (study or studies)).tw.
153 observational study/
154 (observation$2 adj (study or studies)).tw.
155 population research/
156 ((population or population-based) adj (study or studies or analys#s)).tw.
157 ((multidimensional or multi-dimensional) adj (study or studies)).tw.
158 exp comparative study/
159 ((comparative or comparison) adj (study or studies)).tw.
160 exp case control study/
161 ((case-control* or case-based or case-comparison) adj (study or studies)).tw.
162 or/145-161
163 120 and 162
164 127 or 144 or 163
165 exp animal experimentation/ or exp models animal/ or exp animal experiment/ or nonhuman/ or exp vertebrate/
166 exp humans/ or exp human experimentation/ or exp human experiment/
167 165 not 166
168 164 not 167
169 editorial.pt.

Veroniki AA, *et al. BMJ Open* 2022; 12:e053012. doi: 10.1136/bmjopen-2021-053012

170 letter.pt.not (letter.pt. and randomized controlled trial/)
171 168 not (169 or 170)
172 (2011112* or 2011113* or 201112* or 2012* or 2013* or 2014*).dd.
173 171 and 172
174 62 use prmz
175 173 use emez
176 174 or 175
177 remove duplicates from 176
178 177 use prmz [MEDLINE UNIQUE HITS]
179 177 use emez [EMBASE UNIQUE HITS]
****
