# Supplementary material for: Comparative safety and efficacy of cognitive enhancers for Alzheimer’s dementia: a systematic review with individual patient data network meta-analysis
Source: BMJ Open. 2022 Apr 25;12(4):e053012. doi: 10.1136/bmjopen-2021-053012 (PMC9045061; doi:10.1136/bmjopen-2021-053012)
Supplement: Author's manuscript [file bmjopen-2021-053012.draft_revisions.pdf]

BMJ Open

BMJ Open is committed to open peer review. As part of this commitment we make the peer review history of every article we publish publicly available.

When an article is published we post the peer reviewers' comments and the authors' responses online. We also post the versions of the paper that were used during peer review. These are the versions that the peer review comments apply to.

The versions of the paper that follow are the versions that were submitted during the peer review process. They are not the versions of record or the final published versions. They should not be cited or distributed as the published version of this manuscript.

BMJ Open is an open access journal and the full, final, typeset and author-corrected version of record of the manuscript is available on our site with no access controls, subscription charges or pay-per-view fees (http://bmjopen.bmj.com).

If you have any questions on BMJ Open's open peer review process please email info.bmjopen@bmj.com

BMJ Open

Comparative safety and efficacy of cognitive enhancers for Alzheimer's dementia: An individual patient data network meta-analysis

Journal:	*BMJ Open*
Manuscript ID	bmjopen-2021-053012
Article Type:	Original research
Date Submitted by the Author:	03-May-2021
Complete List of Authors:	Veroniki, Areti; University of Ioannina, Department of Primary Education; St. Michael's Hospital, Knowledge Translation Program<br>Ashoor, Huda; St Michael's Hospital, Knowledge Translation Program<br>Rios, Patricia; St Michael's Hospital, Knowledge Translation Program<br>Seitidis, Georgios; University of Ioannina, Department of Primary Education<br>Stewart, Lesley; University of York, Centre for Reviews and Dissemination<br>Clarke, Mike; Queen's University Belfast, Northern Ireland Hub for Trials Methodology Research<br>Tudur-Smith, Catrin; University of Liverpool, Department of Biostatistics<br>Mavridis, Dimitris ; University of Ioannina, Department of Primary Education<br>Hemmelgarn, Brenda; University of Alberta, Department of Medicine<br>Holroyd-Leduc, Jayna; University of Alberta, Department of Medicine<br>Straus, Sharon; St Michael's Hospital, Knowledge Translation Program; University of Toronto, Department of Geriatric Medicine<br>Tricco, Andrea; St Michael's Hospital, Knowledge Translation Program; University of Toronto, Dalla Lana School of Public Health
Keywords:	STATISTICS & RESEARCH METHODS, EPIDEMIOLOGY, Dementia < NEUROLOGY

SCHOLARONE™
Manuscripts

I, the Submitting Author has the right to grant and does grant on behalf of all authors of the Work (as defined in the below author licence), an exclusive licence and/or a non-exclusive licence for contributions from authors who are: i) UK Crown employees; ii) where BMJ has agreed a CC-BY licence shall apply, and/or iii) in accordance with the terms applicable for US Federal Government officers or employees acting as part of their official duties; on a worldwide, perpetual, irrevocable, royalty-free basis to BMJ Publishing Group Ltd ("BMJ") its licensees and where the relevant Journal is co-owned by BMJ to the co-owners of the Journal, to publish the Work in this journal and any other BMJ products and to exploit all rights, as set out in our licence.

The Submitting Author accepts and understands that any supply made under these terms is made by BMJ to the Submitting Author unless you are acting as an employee on behalf of your employer or a postgraduate student of an affiliated institution which is paying any applicable article publishing charge ("APC") for Open Access articles. Where the Submitting Author wishes to make the Work available on an Open Access basis (and intends to pay the relevant APC), the terms of reuse of such Open Access shall be governed by a Creative Commons licence – details of these licences and which Creative Commons licence will apply to this Work are set out in our licence referred to above.

Other than as permitted in any relevant BMJ Author's Self Archiving Policies, I confirm this Work has not been accepted for publication elsewhere, is not being considered for publication elsewhere and does not duplicate material already published. I confirm all authors consent to publication of this Work and authorise the granting of this licence.

For peer review only

Comparative safety and efficacy of cognitive enhancers for Alzheimer's dementia: An individual patient data network meta-analysis

Areti Angeliki Veroniki[1,2,3*] PhD e-mail: averoniki@uoi.gr
Huda M. Ashoor[2] BSc e-mail: huda.ashoor@unityhealth.to
Patricia Rios[2] MSc e-mail: patricia.rios@unityhealth.to
Georgios Seitidis[1] MSc e-mail: g.seitidis@uoi.gr
Lesley A. Stewart[4] PhD e-mail: lesley.stewart@york.ac.uk
Mike Clarke[5] PhD e-mail: m.clarke@qub.ac.uk
Catrin Tudur Smith[6] PhD e-mail: cat1@liverpool.ac.uk
Dimitris Mavridis[1] PhD e-mail: dmavridi@uoi.gr
Brenda R. Hemmelgarn[7] PhD e-mail: brenda.hemmelgarn@albertahealthservices.ca
Jayna Holroyd-Leduc[7] MD e-mail: jayna.holroyd-leduc@albertahealthservices.ca
Sharon E. Straus[2,8] MD e-mail: sharon.straus@utoronto.ca
Andrea C. Tricco[2,9] PhD e-mail: andrea.tricco@unityhealth.to

[1] Department of Primary Education, School of Education, University of Ioannina, Ioannina, Greece
[2] Knowledge Translation Program, Li Ka Shing Knowledge Institute, St. Michael's Hospital, Toronto, Ontario, Canada
[3] Institute of Reproductive and Developmental Biology, Department of Surgery & Cancer, Faculty of Medicine, Imperial College, London, United Kingdom
[4] Centre for Reviews and Dissemination, University of York, York, United Kingdom

For peer review only - http://bmjopen.bmj.com/site/about/guidelines.xhtml

[5] Northern Ireland Hub for Trials Methodology Research, Queen's University Belfast, Belfast, United Kingdom
[6] Department of Biostatistics, University of Liverpool, Block F, Waterhouse Building, 1-5 Brownlow Hill, Liverpool, L69 3GL, UK
[7] Departments of Medicine, University of Alberta, Edmonton, Alberta, Canada;
[8] Department of Geriatric Medicine, University of Toronto, Toronto, Ontario, Canada
[9] Epidemiology Division & Institute of Health Policy, Management, and Evaluation, Dalla Lana School of Public Health, University of Toronto, Toronto, Ontario, Canada

***Corresponding Author:**
Dr. Areti Angeliki Veroniki, MSc, PhD
Research Fellow at the Department of Primary Education, School of Education, University of Ioannina, Ioannina, Greece
Phone: +30 26510 05694; fax: +30 26510 05854; e-mail: averoniki@uoi.gr

Word count: 2,989 (max 4000); 1 table; 4 figures; 22 appendices; 27 references

For peer review only

Abstract

Words: 291 (Max 300 words)

Objective: To examine the comparative efficacy and safety of cognitive enhancers by patient characteristics for managing Alzheimer's Dementia (AD).

Design: Systematic review and individual patient data (IPD) network meta-analysis (NMA)

Participants: 80 randomized controlled trials (RCTs) including 21,138 adults with AD, and 12 RCTs with IPD including 6,906 patients.

Interventions: Cognitive enhancers (donepezil, rivastigmine, galantamine and memantine) alone or in any combination against other cognitive enhancers or placebo.

Data extraction and Synthesis: We requested IPD from authors, sponsors and data sharing platforms. When IPD were not available, we used aggregate data. We conducted a two-stage random-effects IPD-NMA, and assessed their findings using CINeMA (Confidence in Network meta-analysis).

Primary and Secondary Outcomes: We included trials assessing cognition with the Mini-Mental State Examination (MMSE), and serious adverse events (SAEs).

Results: Our IPD-NMA compared 9 treatments (including placebo). Donepezil (mean difference [MD] = 1.41, 95% confidence interval [CI]: 0.51 to 2.32) and donepezil+memantine (MD = 2.57, 95% CI: 0.07 to 5.07) improved MMSE score (56 RCTs, 11,619 participants; CINeMA score: moderate) compared to placebo. Oral rivastigmine (odds ratio [OR] = 1.26, 95% CI: 0.82 to 1.94) and donepezil (OR = 1.08, 95% CI: 0.87 to 1.35) were associated with higher odds of a SAE than placebo (45 RCTs, 15,649 patients; CINeMA score: moderate to high). For moderate to severe impairment,

For peer review only - http://bmjopen.bmj.com/site/about/guidelines.xhtml

donepezil, memantine and their combination performed best, but for mild to moderate impairment donepezil and transdermal rivastigmine ranked best. Adjusting for MMSE baseline differences, oral rivastigmine and galantamine improved MMSE score, whereas when adjusting for comorbidities only oral rivastigmine was effective.

Conclusions: The choice among the different cognitive enhancers may depend on patient's characteristics. All cognitive enhancers except for oral rivastigmine, galantamine, and memantine, were clinically important for cognition (MMSE score greater than 1.4).

Protocol registration number: PROSPERO # CRD42015023507

Keywords: network meta-analysis; multiple treatments meta-analysis; individual participant data; Nootropic Agents; Alzheimer Disease

Strengths and limitations of this study

- This is one of the most comprehensive systematic reviews and network meta-analysis of cognitive enhancers including individual patient data for Alzheimer's Dementia to produce treatment recommendations by patient characteristics.
- We followed the methodologically rigorous guidelines in the Cochrane Handbook for systematic reviews, and the CINeMA quality assessment guidelines.
- Access to individual patient data allowed us to 1) observe minor differences between the original published results and our re-analysis, potentially due to differences in imputation methods for missing data or because original studies have excluded some patients, and hence have used a smaller sample size, 2) overcome potential reporting bias, and 3) assess for potential

For peer review only

For peer review only - http://bmjopen.bmj.com/site/about/guidelines.xhtml

effect modifiers that were not reported in the original publications (e.g., comorbidities, additional medications) and explore for treatment-by-covariate interactions on the patient-level.

- Two thirds of the included RCTs, were associated with high risk of bias for incomplete outcome data due to attrition.
- We were unable to include individual patient data for all RCTs (only 15% of the studies shared their individual patient data), highlighting potential availability bias.

For peer review only

For peer review only - http://bmjopen.bmj.com/site/about/guidelines.xhtml

Introduction

Alzheimer's dementia (AD) is the most common type of dementia.[1] Patients living with AD have a lower quality of life due to deterioration in function, cognition, behavior, and mental health over time, as well as increased mortality.[2] Pharmacological treatment for AD predominantly consists of cholinesterase inhibitors (donepezil, galantamine, rivastigmine) and the N-methyl-d-aspartate (NMDA) receptor antagonist, memantine. It is unclear whether galantamine, rivastigmine, or donepezil should be used by patients with severe AD, or whether memantine is the optimal treatment for severe AD.[3]

In AD, disease severity and sex are potential effect modifiers. However, aggregate data and covariates of interest (e.g., sex, disease severity) are not consistently reported across randomized clinical trials (RCTs).[4] The aim of this study was to examine the comparative efficacy and safety of cognitive enhancers for patients with different characteristics, such as severities of AD and for females versus males through a systematic review and individual patient data (IPD) NMA.

Methods

We reported our results according to the Preferred Items for Systematic Reviews and Meta-analysis (PRISMA) Statement for NMA and PRISMA-IPD.[5,6]

Protocol

For peer review only - http://bmjopen.bmj.com/site/about/guidelines.xhtml

The research question and protocol were based on our previous systematic review and NMA.[4] We registered our systematic review protocol with the prospective register of systematic reviews (PROSPERO: CRD42015023507), and published our protocol.[7] Additional information is also provided in Appendix 1. Herein, we briefly summarize our methods.

Eligibility criteria

We updated our previous systematic review,[4] using similar population, interventions, comparators, study designs and time period (PICOST) criteria. The literature search was updated from January 2015 to March 2016. We included RCTs that assessed cognition via the Mini-Mental State Examination (MMSE; efficacy and primary outcome) and/or serious adverse events (SAE; safety outcome) in adults with Alzheimer's dementia.

IPD collection process

We contacted the corresponding author followed by the next-in-order author, as presented in each eligible RCT, to obtain IPD. The author contact process was part of a RCT that our team conducted to assess methods that may optimize response rates for IPD retrieval.[8] We also contacted sponsors of eligible trials, as reported in the publications. We contacted industry sponsors only, as we were not able to locate contact information for the majority of non-industry sponsors (e.g., grants and university funding). If a study had multiple sponsors, we contacted all of them. To further facilitate IPD access, we contacted the Clinical Study Data Request (CSDR)[9] and Yale University Open Data

For peer review only - http://bmjopen.bmj.com/site/about/guidelines.xhtml

Access (YODA) data sharing platforms.[10] If a data provider was unable to provide IPD we noted the reason.

Risk of bias and quality appraisal

We appraised study quality using the Cochrane risk of bias tool.[11] To ensure data consistency[6] we compared IPD with aggregate data reported in the publication. We assessed whether randomization of patients was adequate (i.e., intervention and comparison groups were balanced for important patient characteristics), by comparing numbers and types of patients in each arm.

When at least 10 studies were available for each treatment against placebo, publication bias and small-study effects were examined visually using funnel plots under the fixed-effect model.[12] Confidence in NMA findings was assessed for each outcome using CINeMA (Confidence in Network meta-analysis, see Appendix 1 for more details).[13]

Synthesis

We performed a descriptive analysis using frequencies and distributions of the characteristics of the included patients and treatments. For each outcome, we present the network geometry according to IPD availability. We conducted a two-stage IPD analysis, whereby data were analysed separately in each trial in the first stage and the trial parameter estimates were synthesised in a random-effects meta-analysis or NMA in the second stage.

For peer review only - http://bmjopen.bmj.com/site/about/guidelines.xhtml

The summary treatment effects are presented using the odds ratio (OR) or mean difference (MD) along with their corresponding CIs and predictive intervals (PrIs).[14] We ranked the interventions for each outcome using the P-scores (and SUCRAs [surface under the cumulative ranking curve] in meta-regression analysis), and present them in a rank-heat plot.[15,16]

Results

Literature search, study selection and IPD obtained

After screening 20,410 titles and abstracts and 1,968 full-text articles, 96 studies fulfilled the eligibility criteria; 80 unique studies and 16 companion reports (Figure 1a, Appendix 2).

(Figure 1 here)

Of the 80 RCTs, 55 reported at least one industry-sponsored funder (i.e. 40 studies reported a single industry-sponsor and 15 multiple industry-sponsors). In the remaining studies, 9 were publicly-sponsored and 16 did not report any information about funding. We requested IPD by contacting the corresponding authors for 80 RCTs that included 21,138 participants. None of the original authors shared their IPD. Fifteen commercial sponsors were then contacted and 6 (40%) sponsors shared their data through proprietary sponsor-specific platforms. The 6 sponsors were contacted for 46 RCTs (14,580 participants), and we obtained IPD for 30% (14 RCTs, 8,007 participants) of these RCTs (1,058 total waiting days up to March 9, 2020). The study flow for obtaining IPD is depicted in Figure 1b.

For peer review only - http://bmjopen.bmj.com/site/about/guidelines.xhtml

We were able to include 12 (6,906 patients) of 14 RCTs in our NMA due to incompleteness of provided IPD (Appendix 3). The number of studies with available/non-available IPD from each data provider along with reasons for non-availability of IPD are presented in Appendix 4.

Study and patient characteristics

Most included studies (33%) were multi-national. The mean age of patients ranged from 61 to 86 years. The majority of the RCTs included patients with mild-moderate AD (56%), although the diagnostic criteria used for AD varied widely (Table 1). The most frequent longest duration of follow-up was 24 weeks (24 RCTs, 30%; Appendix 5). The intervention and comparison groups were not balanced across all RCTs with provided IPD for important patient characteristics, such as percent of male and dropout rates (Appendix 6). Comparing study and patient characteristics of available and non-available IPD when a study was industry-sponsored, we found differences in the year of study publication, study size, and absolute mean difference (Appendix 7).

(Table 1 here)

Risk of bias and IPD integrity

Using the Cochrane risk-of-bias tool, allocation concealment was at low risk of bias for 43% and blinding of participants and personnel was low for 64% of the RCTs (Appendix 8). One third of the RCTs had low risk of incomplete outcome data bias due to attrition and almost two thirds had high potential risk of "other" bias, specifically, funding bias. The other risk of bias

For peer review only - http://bmjopen.bmj.com/site/about/guidelines.xhtml

item was scored as unclear for 32%. Overall risk of bias was comparable in studies with available and unavailable IPD (Appendix 9).

All IPD provided were checked for consistency and results from published RCTs were reproduced and provided in Appendix 10. High dropout rates were observed in the IPD; experiencing an adverse event was the most common reason for dropout. Despite the high dropout rates observed in the individual studies, there was no indication of correlation between age and dropout (Appendix 11). Comparison-adjusted funnel plots suggested there is indication for small-study effects (see Appendix 12).

Network meta-analysis

In both MMSE and SAE outcomes, on average there were no important concerns regarding the transitivity and consistency assumptions (Appendices 13 and 14; design-by-treatment interaction model MMSE: χ^2= 4·36, 13 degrees of freedom (df), P= 0·987; SAE: χ^2= 3·57, 6 df, P= 0·735). Below we present the main analysis results compared to placebo. Additional analyses are presented in Appendices 15-16). The network geometry is presented in Figure 2.

(Figure 2 here)

Cognition

The NMA for MMSE included 56 RCTs, 9 treatments (including placebo), and 11,619 participants. Nine RCTs (3,625 patients) contributed IPD and 47 RCTs (7,994 patients) contributed aggregated data to the NMA. Two studies[17,18] did not report MMSE

For peer review only - http://bmjopen.bmj.com/site/about/guidelines.xhtml

in the final publication, but in the retrieved IPD we were able to use data for this outcome.

NMA of studies with IPD and aggregate data

Studies in this NMA compared all available treatments. Donepezil (MD= 1·41, 95% CI: 0·51 to 2·32) and donepezil+memantine (MD= 2·57, 95% CI: 0·07 to 5·07) were superior to placebo (Appendices 16-17). PrIs suggested results are not conclusive. Transdermal rivastigmine, and the combinations donepezil+memantine, galantamine+memantine, and transdermal rivastigmine+memantine were associated with a minimal clinically important difference (MCID; above 1·40)[19] (Figure 3a). However, donepezil+memantine had the highest likelihood of being the most effective in improving MMSE score (P-score range 79-80%, Figure 4). Confidence in NMA results was moderate (Appendix 18).

(Figure 3 here)

(Figure 4 here)

NMA of studies with aggregate data

Studies in this NMA compared also donepezil+memantine, galantanmine+memantine, and transdermal rivastigmine +memantine. Donepezil improved MMSE score significantly (MD= 1·55 95% CI: 0·41 to 2·68). The MCID results were in agreement with the NMA of IPD and aggregate data, and donepezil+memantine was likely the most effective in improving MMSE score (P-score= 76%).

NMA of studies with IPD

Studies in this NMA compared placebo, donepezil, oral rivastigmine, transdermal rivastigmine, galantamine, and memantine. Donepezil (MD= 0·70, 95% CI: 0·01 to 1·40) and transdermal rivastigmine (MD= 1·06, 95% CI: 0·04 to 2·08) were

For peer review only - http://bmjopen.bmj.com/site/about/guidelines.xhtml

superior to placebo, but none was at a MCID. The most effective treatment was likely transdermal rivastigmine (P-score= 82%).

Serious adverse events

A NMA was conducted on serious adverse events (study definitions are provided in Appendix 19) with 45 RCTs, 9 treatments (including placebo), and 15,649 patients (Figure 2b). In particular, 12 RCTs (6420 patients) contributed to the NMA using their IPD and 33 RCTs (9229 patients) using their data on their aggregated form. The time taken to achieve at least one SAE was available in 8 studies with available IPD and ranged between 45 and 2228 days (Appendix 20). Only one study included a patient with a SAE occurring earlier than the trial opening and was excluded from the study.[20]

NMA of studies with IPD and aggregate data

Studies in this NMA compared all available treatments. Oral rivastigmine had the least favourable safety profile regarding SAE (OR= 1·26, 95% CI: 0·82 to 1·94, P-score= 16%). Donepezil (OR= 1·08, 95% CI: 0·87 to 1·35, P-score= 30%) and galantamine+memantine (OR= 1·03, 95% CI: 0·45 to 2·39, P-score= 43%) were associated with higher odds of a SAE than placebo, yet none of these comparisons were statistically significant (Figure 3b; Appendices 17, 21). All other treatments were considered to have a favourable safety profile compared with placebo. Confidence in NMA results ranged between moderate and high (Appendix 18).

NMA of studies with aggregate data

Studies in this NMA compared all available treatments. Results were mainly consistent with NMA of IPD and aggregate data, but

For peer review only

For peer review only - http://bmjopen.bmj.com/site/about/guidelines.xhtml

for memantine which was statistically significantly associated with lower odds of a SAE than placebo when using aggregate data only (OR 0·70, 95% CI: 0·51 to 0·97, P-score= 77%, Appendix 16).

NMA of studies with IPD

Studies in this NMA compared placebo, donepezil, oral rivastigmine, transdermal rivastigmine, galantamine, and memantine. Results were on average consistent with NMA of IPD and aggregate data.

Discussion

We compared the efficacy and safety of cognitive enhancers regarding MMSE and SAE outcomes to update our previous systematic review[4] and included studies with both aggregate data and IPD. Our results are in agreement with our previous systematic review,[4] and show that donepezil+memantine, donepezil alone and transdermal rivastigmine were the most effective treatments for improving MMSE score. However, heterogeneity was a major concern, and this was also captured by PrIs. Both donepezil+memantine and transdermal rivastigmine had a favourable safety profile regarding SAE. Among all cognitive enhancers, the therapy with the least favourable profile was oral rivastigmine followed by donepezil. According to CINeMA within-study bias and reporting bias were the highest concerns for the MMSE outcome, whereas within-study bias and imprecision of effect estimates were the highest concerns for the SAE outcome.

Overall, the choice among the different cognitive enhancers may depend on the patient's characteristics. In participants with moderate to severe cognitive impairment (defined by MMSE), a

For peer review only - http://bmjopen.bmj.com/site/about/guidelines.xhtml

larger improvement in cognitive performance was observed for donepezil and memantine, and their combination (donepezil+memantine), and these efficacy-related results are expected to also be reflected when a future study becomes available. The least effective cognitive enhancer in participants with moderate to severe cognitive impairment was oral rivastigmine. For patients with mild to moderate impairments based on MMSE scores, donepezil and transdermal rivastigmine were most likely the best performing cognitive enhancers. For patients with moderate to severe cognitive impairment, cognitive enhancers were well tolerated. For patients with mild to moderate cognitive impairment, all except for memantine and its combination with transdermal rivastigmine, were associated with increased odds of a SAE, yet none of these results reached statistical significance. Of note, the accuracy of SAE reporting may be impacted by the degree of cognitive impairment. Using IPD only and adjusting for MMSE baseline differences, oral rivastigmine and galantamine improved MMSE score, whereas when adjusting for comorbidities only oral rivastigmine was effective, but results can change in a future study. Considering a MCID equal to 1·4,[19] all cognitive enhancers except for oral rivastigmine, galantamine, and memantine, were clinically important for cognition. Our results did not differ by participant characteristics sex, age, and other medications, or by study characteristics, study duration and year of publication. However, these findings might be due to low power since meta-regression analyses depend on the number and size of studies, magnitude of the relationship between the covariate and effect size, along with its precision and heterogeneity.[21]

For peer review only - http://bmjopen.bmj.com/site/about/guidelines.xhtml

To the best of our knowledge, our study was the first to add IPD in a NMA of cognitive enhancers for patients with Alzheimer's Dementia to produce treatment recommendations by patient characteristics. We followed the methods guidelines in the Cochrane Handbook for systematic reviews,[22] the reporting guidelines in the PRISMA-NMA and PRISMA-IPD statements,[5,6] and the CINeMA quality assessment guidelines.[13] Compared to previous systematic reviews, we included a larger number of studies and/or studies with shared IPD, compared in a wider range of cognitive enhancers.[4,23] Our results are in agreement with previous studies overall. Access to IPD allowed us to observe minor differences between the original published results and our re-analysis. An explanation in these differences may be that many studies used the last-observation-carried-forward imputation method, whereas we used the available case analysis when assessing MMSE. Another potential explanation might be that original studies excluded some patients, and hence used a smaller sample size.

Comparing NMA, results between aggregate data and IPD were in agreement. The only difference was observed in transdermal rivastigmine that was associated with a MCID of MMSE in the aggregate data NMA compared to the IPD NMA, yet a statistically significant improvement was achieved in the IPD NMA. The inclusion of IPD in our NMA, allowed us to overcome potential reporting bias and to include IPD for 1) a study that we previously were unable to include since arm-level data were not reported in the RCT publication,[20] and 2) two studies that did not report MMSE results in their publications.[17,18] The use of IPD also allowed us to assess for potential effect modifiers that were not reported in the original publications (e.g., comorbidities, additional medications) and explore for treatment-by-covariate interactions on the patient-level. Several challenges were encountered during the IPD request from sponsors, showing that repositories are not a panacea (Appendix 22).

For peer review only - http://bmjopen.bmj.com/site/about/guidelines.xhtml

An important finding of our review is that the two thirds of the published RCTs, were associated with high risk of bias for incomplete outcome data due to attrition, and the majority of these RCTs used the last-observation-carried-forward technique for missing data. This approach may bias results favouring cognitive enhancers, since the dropout rates were greater in the treatment group compared to the placebo group in 63% of the included studies and because dementia is a progressive disease. Of the 27 studies comparing treatment against placebo and reporting the number of dropouts, 17 studies had a greater dropout rate in the treatment group (treatment group: median dropout rate= 28% IQR [17% to 39%]; placebo group: median dropout rate= 21% IQR [15% to 31%]). Last-observation-carried-forward is an inappropriate imputation method for Alzheimer's Dementia studies, since it ignores expected deterioration of the patient's condition and stabilizes the outcome at the value observed at the time of dropout (i.e., the last observation).[24] Restricting to low risk of attrition bias studies, we found that galantamine was significantly associated with decreased odds of experiencing a SAE.

Our study has limitations worth mentioning. First, we were unable to include IPD for all eligible studies (only 15% of the included RCTs shared their IPD), highlighting potential availability bias for IPD. However, recent simulations have shown that combining IPD and aggregate data in a NMA can significantly improve precision, reduce bias, and increase information compared to NMA relying on aggregated data alone.[25] Second, missing data is a big concern in the published RCTs for Alzheimer's Dementia. To assess the impact of missing data in our NMA, we applied the informative missingness of difference in means.[26] Third, the lack of studies in certain treatment comparisons may have affected the P-score calculation and treatment ranking. In particular, polytherapies were informed by maximum two studies, and ranking may have been in favour of the complex intervention group with the smaller number of studies.[27] For example,

For peer review only - http://bmjopen.bmj.com/site/about/guidelines.xhtml

in MMSE the polytherapies including memantine in conjunction with one of the three treatments donepezil, galantamine, transdermal rivastigmine had a P-score ≥60%, but these all had wide 95% CIs for MD. As such, ranking should be interpreted with caution and along with the estimated effect sizes and their uncertainty measures. Fourth, the comparison-adjusted funnel plot for MMSE suggested there is an indication for small-study effects pointing to the treatment being better, and results should be interpreted with caution. Overall, MMSE score is only a surrogate maker for determining the impact of treatments on dementia. A full assessment that considers the potential impact of treatments on cognition, function and behavioural symptoms needs to be considered within the clinical context.

We expect that our findings will increase scientific knowledge, because people with Alzheimer's Dementia require personalized medicine to optimize their healthcare. Well-conducted meta-analyses of IPD are considered the 'gold-standard' and influence patient care since patient-level data can be provided to facilitate tailored decision making. However, results from meta-analyses of IPD are likely subject to retrieval bias and awareness of these limitations and their potential impact on findings is required.

For peer review only - http://bmjopen.bmj.com/site/about/guidelines.xhtml

Contributors

AAV, SES and ACT conceived and designed the study. AAV conducted the analyses, abstracted data, contacted sponsors, analysed data, interpreted results, appraised quality of results, and wrote a draft manuscript. GS conducted the analyses, appraised quality of results, and edited the manuscript. HMA coordinated the review, screened citations and full-text articles, abstracted data, appraised quality, cleaned the data, contacted sponsors, and edited the manuscript. PR helped coordinate the study, screened citations and full-text articles, extracted and categorized data, appraised quality, and edited the manuscript. SES and ACT interpreted results and edited the manuscript. ACT and HMA contacted authors. LAS, MC, CTS, DM, BRH, JHL provided input into the design, interpreted results, and edited the manuscript. All authors read and approved the final manuscript.

Declaration of interests

The authors declare that they have no competing interests.

Data sharing statement

All data relevant to the study are included in the article or uploaded as supplementary information.

Funding

This research was funded by the CIHR Drug Safety and Effectiveness Network (grant number 137713). The funder contributed to defining the scope of the systematic review but otherwise had no role in study design, data collection, analysis, and

For peer review only

interpretation of data. Data sharing sponsors were provided the ability to review at the time of submission of the manuscript for publication.

AAV was previously funded by the Canadian Institutes of Health Research (CIHR) Banting Postdoctoral Fellowship Program [No. 139157]. AAV, GS and DM are funded from the European Union's Horizon 2020 [No. 754936]. SES is funded by a Tier 1 Canada Research Chair in Knowledge Translation [No. N/A]. ACT is funded by a Tier 2 Canada Research Chair in Knowledge Synthesis [No. N/A].

Open Access

This is an open access article distributed in accordance with the Creative Commons Attribution Non Commercial (CC BY-NC 4.0) license, which permits others to distribute, remix, adapt, build upon this work non-commercially, and license their derivative works on different terms, provided the original work is properly cited, appropriate credit is given, any changes made indicated, and the use is non-commercial. See: http:// creativecommons. org/ licenses/ by- nc/ 4. 0/.

Patient and public involvement

None.

Acknowledgements

We thank Laure Perrier for conducting the initial literature search, Alissa Epworth for updating it, and Becky Skidmore for peer reviewing the literature search. We thank Robert Peterson for his support on this study as a knowledge user. We would also like to thank Paul A. Khan, Fatemeh Yazdi, Marco Ghassemi, John D. Ivory, Charlene Soobiah, Erik Blondal, Joanne M. Ho, Shirra Berliner, and Carmen H. Ng for screening some of the citations, data abstracting included studies, or both. We thank Susan Le for helping with contacting the authors and sponsors of the included studies in this review. Finally, we thank Susan Le for formatting the manuscript and assisting with the submission.

For peer review only

For peer review only - http://bmjopen.bmj.com/site/about/guidelines.xhtml

We would also like to thank the following sponsors for sharing the data with us:

- "This publication is based on research using data from data contributors, AbbVie, Inc, that has been made available through Vivli, Inc. Vivli has not contributed to or approved, and is not in any way responsible for, the contents of this publication."
- "This study, carried out under YODA Project #2017-1671, used data obtained from the Yale University Open Data Access Project, which has an agreement with JANSSEN RESEARCH & DEVELOPMENT, L.L.C.. The interpretation and reporting of research using this data are solely the responsibility of the authors and does not necessarily represent the official views of the Yale University Open Data Access Project or JANSSEN RESEARCH & DEVELOPMENT, L.L.C.."
- This publication used data obtained from Eisai, GlaxoSmithKline, and Novartis carried under www.ClinicalStudyDataRequest.com
- This publication used data obtained from Lundbeck

For peer review only - http://bmjopen.bmj.com/site/about/guidelines.xhtml

References

1. Health NCCfM. Dementia: A NICE-SCIE guideline on supporting people with dementia and their carers in health and social care. 2007: British Psychological Society; 2007.
2. Dudgeon S. Rising Tide: The Impact of Dementia on Canadian Society: a Study. 2010: Alzheimer Society of Canada; 2010.
3. National Institute for Health and Clinical Excellence. Donepezil, galantamine, rivastigmine and memantine for the treatment of Alzheimer's disease. London, UK, 2011.
4. Tricco AC, Ashoor HM, Soobiah C, et al. Comparative Effectiveness and Safety of Cognitive Enhancers for Treating Alzheimer's Disease: Systematic Review and Network Metaanalysis. *J Am Geriatr Soc* 2018; **66**(1): 170-8.
5. Hutton B, Salanti G, Caldwell DM, et al. The PRISMA extension statement for reporting of systematic reviews incorporating network meta-analyses of health care interventions: checklist and explanations. *Ann Intern Med* 2015; **162**(11): 777-84.
6. Stewart LA, Clarke M, Rovers M, et al. Preferred Reporting Items for Systematic Review and Meta-Analyses of individual participant data: the PRISMA-IPD Statement. *JAMA* 2015; **313**(16): 1657-65.
7. Veroniki AA, Straus SE, Ashoor HM, et al. Comparative safety and effectiveness of cognitive enhancers for Alzheimer's dementia: protocol for a systematic review and individual patient data network meta-analysis. *BMJ Open* 2016; **6**(1): e010251.
8. Veroniki AA, Ashoor HM, Le SPC, et al. Retrieval of individual patient data depended on study characteristics: a randomized controlled trial. *J Clin Epidemiol* 2019; **113**: 176-88.
9. So D, Knoppers BM. Ethics approval in applications for open-access clinical trial data: An analysis of researcher statements to clinicalstudydatarequest.com. *PLoS One* 2017; **12**(9): e0184491.
10. Krumholz HM, Waldstreicher J. The Yale Open Data Access (YODA) Project--A Mechanism for Data Sharing. *N Engl J Med* 2016; **375**(5): 403-5.
11. Higgins JP, Altman DG, Gotzsche PC, et al. The Cochrane Collaboration's tool for assessing risk of bias in randomised trials. *BMJ* 2011; **343**: d5928.
12. Sterne JA, Egger M. Funnel plots for detecting bias in meta-analysis: guidelines on choice of axis. *J Clin Epidemiol* 2001; **54**(10): 1046-55.
13. CINeMA: Confidence in Network Meta-Analysis. Institute of Social and Preventive Medicine, University of Bern. *Available from cinemaispmch* 2017.
14. Riley RD, Higgins JP, Deeks JJ. Interpretation of random effects meta-analyses. *BMJ* 2011; **342**: d549.
15. Veroniki AA, Straus SE, Fyraridis A, Tricco AC. The rank-heat plot is a novel way to present the results from a network meta-analysis including multiple outcomes. *J Clin Epidemiol* 2016; **76**: 193-9.
16. Rucker G, Schwarzer G. Ranking treatments in frequentist network meta-analysis works without resampling methods. *BMC Med Res Methodol* 2015; **15**: 58.
17. Herrmann N, Gauthier S, Boneva N, Lemming OM. A randomized, double-blind, placebo-controlled trial of memantine in a behaviorally enriched sample of patients with moderate-to-severe Alzheimer's disease. *Int Psychogeriatr* 2013; **25**(6): 919-27.
18. Burns A, Bernabei R, Bullock R, et al. Safety and efficacy of galantamine (Reminyl) in severe Alzheimer's disease (the SERAD study): a randomised, placebo-controlled, double-blind trial. *Lancet Neurol* 2009; **8**(1): 39-47.
19. Howard R, Phillips P, Johnson T, et al. Determining the minimum clinically important differences for outcomes in the DOMINO trial. *Int J Geriatr Psychiatry* 2011; **26**(8): 812-7.
20. Gold M, Alderton C, Zvartau-Hind M, et al. Rosiglitazone monotherapy in mild-to-moderate Alzheimer's disease: results from a randomized, double-blind, placebo-controlled phase III study. *Dement Geriatr Cogn Disord* 2010; **30**(2): 131-46.
21. Hedges L, Higgins J, Rothstein H. Introduction to Meta-Analysis. John Wiley & Sons; 2009.

For peer review only

For peer review only - http://bmjopen.bmj.com/site/about/guidelines.xhtml

22. Higgins JP, Thomas J, Chandler J, et al. Cochrane handbook for systematic reviews of interventions: John Wiley & Sons; 2019.
23. Cui CC, Sun Y, Wang XY, Zhang Y, Xing Y. The effect of anti-dementia drugs on Alzheimer disease-induced cognitive impairment: A network meta-analysis. *Medicine (Baltimore)* 2019; **98**(27): e16091.
24. Molnar FJ, Hutton B, Fergusson D. Does analysis using "last observation carried forward" introduce bias in dementia research? *CMAJ* 2008; **179**(8): 751-3.
25. Jansen JP. Network meta-analysis of individual and aggregate level data. *Res Synth Methods* 2012; **3**(2): 14.
26. Mavridis D, White IR. Dealing with missing outcome data in meta-analysis. *Res Synth Methods* 2020; **11**(1): 2-13.
27. Kibret T, Richer D, Beyene J. Bias in identification of the best treatment in a Bayesian network meta-analysis for binary outcome: a simulation study. *Clin Epidemiol* 2014; **6**: 451-60.

For peer review only

Figure Captions

Figure 1. Flow diagram for study inclusion in the review (a) and studies retrieved with individual patient data (b).

Figure 2. Network diagrams for (a) MMSE and (b) SAE outcomes. The size of each node and line indicates the number of studies included in each treatment comparison. The number of studies per treatment comparison is presented on each edge, and the number of studies with individual patient data (IPD) is depicted in a parenthesis. Orange coloured edges are informed by both IPD and aggregate data, whereas black coloured edges are informed by aggregate data only.

Figure 3. Forest plot of network meta-analysis (NMA) results for all cognitive enhancers versus placebo in (a) MMSE outcome, and (b) SAE outcome. NMA results are presented for i) aggregate data (AD) and fully adjusted results from studies with available individual patient data (IPD), ii) AD and crude results from studies with available IPD, iii) AD only (studies with available IPD are not included in the analysis), and iv) crude results from individual studies with individual patient data (IPD).

Figure 4. Rank-heat plot of P-scores for 9 treatments, including placebo, studied in randomized clinical trials with patients with Alzheimer's Dementia assessing MMSE. Circles from inside out present results for different network meta-analyses including: i) aggregate data (AD) only (studies with available IPD are not included in the analysis), ii) crude results from individual studies with individual patient data (IPD), iii) AD and crude results from studies with available IPD, and iv) AD and fully adjusted results from studies with available IPD. Numbers within each sector correspond to the P-score values as calculated in each model.

For peer review only - http://bmjopen.bmj.com/site/about/guidelines.xhtml

Tables

Table 1· Study and patient characteristics

	AD (N=80)	IPD (N=12)
**Total # participants**	21,138	5839
**Longest duration of follow-up in weeks: mean (range)**	28·28 (8 - 208)	29·33 (12 - 104)
**Mean number of patients (range)**	264·23 (14 - 2,045)	486·58 (123 - 2,045)
**Mean age in years (range)**	74·64 (61 - 85·7)	73·94 (70·4 - 78)
**Mean % Female (range)**	61·35 (3 - 89)	62·76 (53·68 - 81)
**Country of conduct: frequency (%)**		
Canada	2 (2·50)	1 (8·33)
China	6 (7·50)	-
Germany	1 (1·25)	-
Iran	2 (2·50)	-
Italy	6 (7·50)	-
Japan	7 (8·75)	1 (8·33)
Norway	1 (1·25)	-
Romania	1 (1·25)	-
South Korea	1 (1·25)	-
Spain	3 (3·75)	-
Sweden	2 (2·50)	-
Turkey	1 (1·25)	-
United Kingdom	6 (7·50)	1 (8·33)
United States	15 (18·75)	-
Multi-national	26 (32·50)	9 (75·00)
**Interventions examined: frequency***		
Placebo/no treatment	61 (76·25)	12 (100·00)
Donepezil	47 (58·75)	4 (33·33)
Galantamine	20 (25·00)	4 (33·33)
Memantine	20 (25·00)	3 (25·00)
Rivastigmine**	18 (22·50)	1 (8·33)
**Effectiveness outcomes reported: frequency***		
Mini-Mental State Examination	57 (71·25)	6 (50·00)
Serious Adverse Events	46 (57·50)	12 (100·00)
**Funding**		
Industry-sponsored	48 (60·00)	12 (100·00)
Publicly-sponsored‡	9 (11·25)	-
Mixed	7 (8·75)	-
Not Reported	16 (20·0)	-
**Severity of Alzheimer's dementia: frequency (%)**		
Mild	3 (3·75)	-
Mild-Moderate	44 (55·00)	7 (58·33)
Mild-Severe	2 (2·50)	-
Moderate	3 (3·75)	-
Moderate-Severe	11 (13·75)	1 (8·33)
Severe	6 (7·50)	2 (16·67)

For peer review only

For peer review only - http://bmjopen.bmj.com/site/about/guidelines.xhtml

Not Reported	11 (13·75)	2 (16·67)
**Diagnostic criteria for Alzheimer's dementia: frequency***		
Mini-Mental State Examination	70 (87·50)	12 (100·00)
National Institute of Neurological Disorders and Stroke-Alzheimer Disease and Related Disorders Association	67 (83·75)	12 (100·00)
Diagnostic and Statistical Manual of Mental Disorders	39 (48·75)	5 (41·67)
Magnetic Resonance Imaging/Computerized Tomography	9 (11·25)	2 (16·67)
Clinical Dementia Rating	6 (7·50)	-
Hachinski Ischemic Score	5 (6·25)	-
Alzheimer's Disease Assessment Scale-Cognitive Subscale	3 (3·75)	1 (8·33)
Other	20 (25·00)	1 (8·33)

Abbreviations: -, not applicable
[*] Multiple interventions and outcomes reported per study;
[**] Rivastigmine refers to either oral or transdermal administration
[ǂ] Including sponsors such as the National Institute of Aging, UK Medical Research Council, and Veteran Affairs

For peer review only

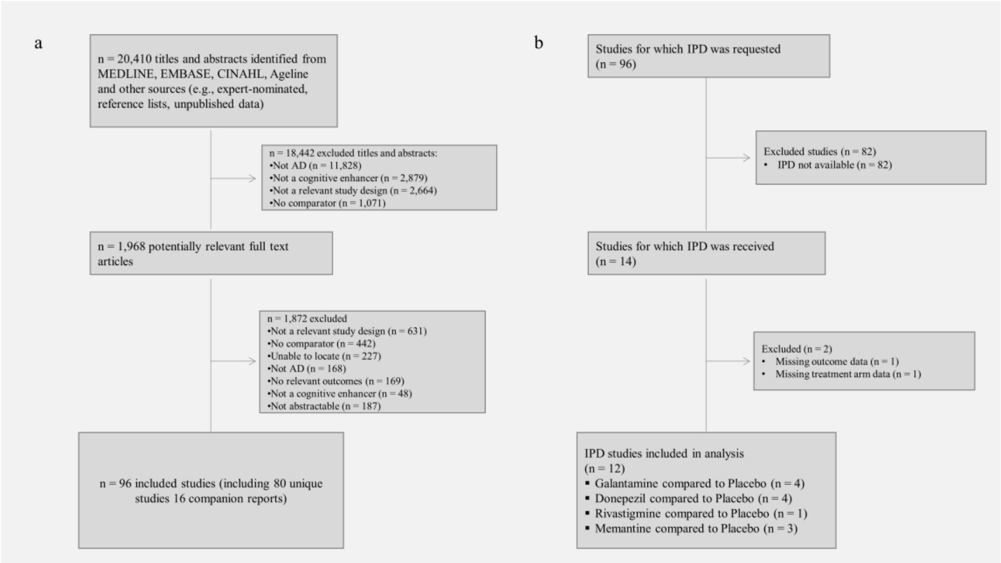

Figure 1. Flow diagram for study inclusion in the review (a) and studies retrieved with individual patient data (b).

338x190mm (300 x 300 DPI)

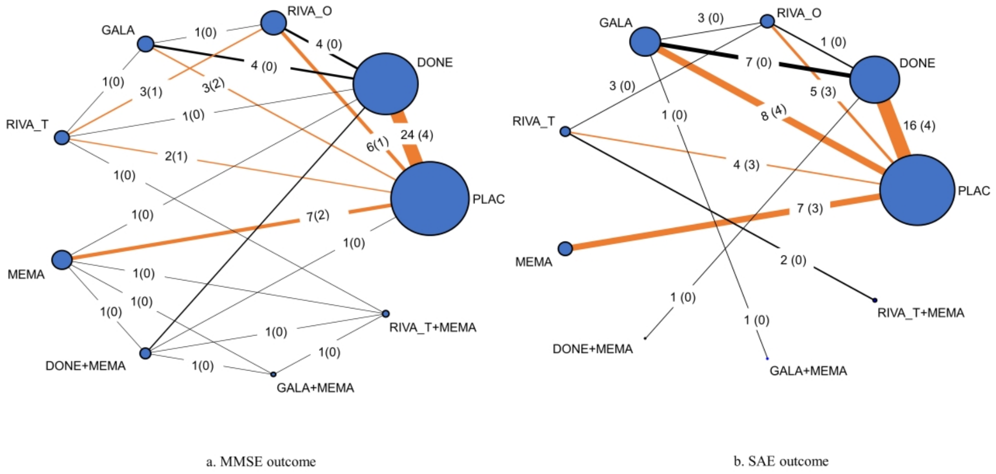

Figure 2. Network diagrams for (a) MMSE and (b) SAE outcomes. The size of each node and line indicates the number of studies included in each treatment comparison. The number of studies per treatment comparison is presented on each edge, and the number of studies with individual patient data (IPD) is depicted in a parenthesis. Orange coloured edges are informed by both IPD and aggregate data, whereas black coloured edges are informed by aggregate data only.

338x190mm (300 x 300 DPI)

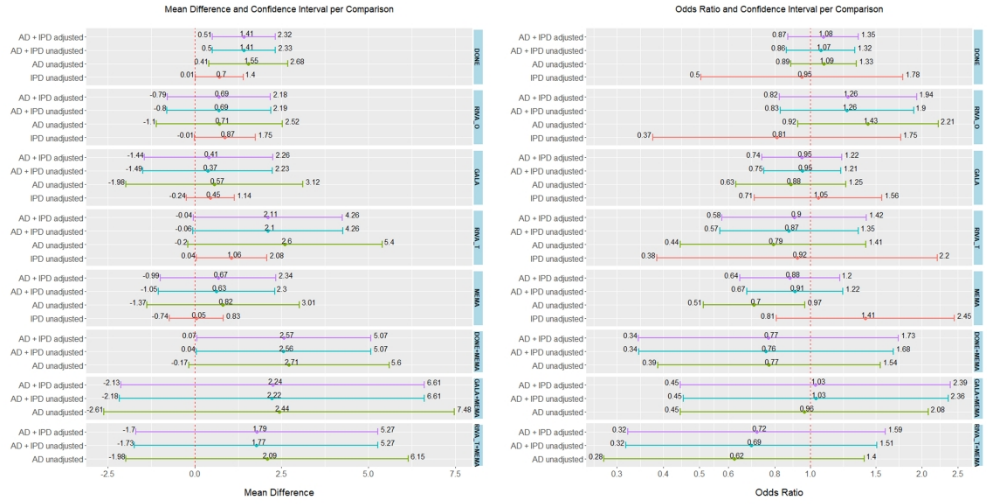

a. MMSE outcome

b. SAE outcome

Figure 3. Forest plot of network meta-analysis (NMA) results for all cognitive enhancers versus placebo in (a) MMSE outcome, and (b) SAE outcome. NMA results are presented for i) aggregate data (AD) and fully adjusted results from studies with available individual patient data (IPD), ii) AD and crude results from studies with available IPD, iii) AD only (studies with available IPD are not included in the analysis), and iv) crude results from individual studies with individual patient data (IPD).

338x190mm (300 x 300 DPI)

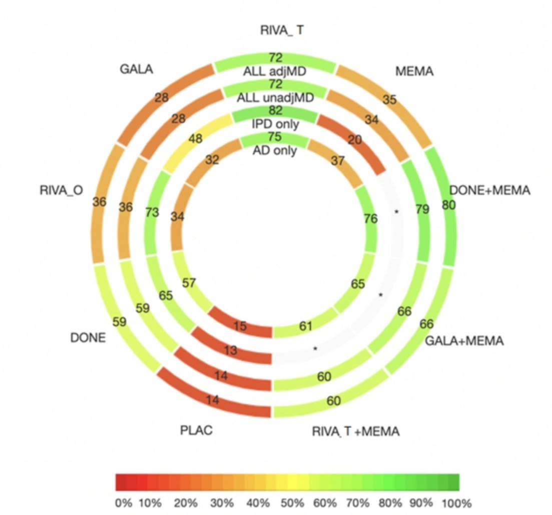

Figure 4. Rank-heat plot of P-scores for 9 treatments, including placebo, studied in randomized clinical trials with patients with Alzheimer's Dementia assessing MMSE. Circles from inside out present results for different network meta-analyses including: i) aggregate data (AD) only (studies with available IPD are not included in the analysis), ii) crude results from individual studies with individual patient data (IPD), iii) AD and crude results from studies with available IPD, and iv) AD and fully adjusted results from studies with available IPD. Numbers within each sector correspond to the P-score values as calculated in each model.

338x190mm (300 x 300 DPI)

Appendix 1: Additional information on the methods used in the review 2
Appendix 2: Studies included in the systematic review 7
Appendix 3: Studies with available IPD but insufficient data to be included in the analysis .13
Appendix 4: List of studies requested and sponsor response 14
Appendix 5: Study characteristics of the included RCTs 17
Appendix 6. Characteristics of studies with shared IPD..24
Appendix 7: Comparison of studies with shared IPD with (a) all remaining studies and (b) studies for which sponsors claimed unavailable IPD. AD: aggregate data; IPD: individual patient data 27
Appendix 8: Cochrane Risk-of-bias appraisal results (n = 80)...29
Appendix 9: Overall risk of bias for studies with shared IPD against (a) all remaining studies and (b) studies for which sponsors claimed unavailable IPD. AD: aggregate data; IPD: individual patient data 31
Appendix 10: Study-specific effect sizes calculated from shared IPD and published data. IPD: individual patient data 32
Appendix 11: Correlation between participant age and dropout in studies with IPD. IPD: individual patient data 37
Appendix 12: Comparison Adjusted Funnel plot (all treatments vs placebo) 38
Appendix 13: Distribution of potential effect modifiers per treatment comparison and outcome 39
Appendix 14: Consistency Assessment – Loop-specific approach (using adjusted treatment effects)..42
Appendix 15: Network and standard meta-analysis results 43
Appendix 16: Additional analyses using IPD and aggregate data 46
Appendix 17: Network subgroup and meta-regression analysis results 47
Appendix 18: CINeMA results 54
Appendix 19: Rank-heat plot for serious adverse events...57
Appendix 20: Study definitions for serious adverse events...58
Appendix 21: Time taken to achieve at least one serious adverse event using individual patient data 61
Appendix 22: Challenges encountered during the individual patient data request from sponsors...62

For peer review only - http://bmjopen.bmj.com/site/about/guidelines.xhtml

Appendix 1: Additional information on the methods used in the review

Eligibility criteria, search strategy and study selection

We considered a SAE as defined in the individual trials, specifically, when an event led to disability or hospitalization or was life-threatening or fatal. Study definitions for a SAE were also abstracted. We included donepezil, rivastigmine, galantamine, and memantine alone or in combination with other treatment and compared with each other, supportive care or placebo. We excluded studies examining other cognitive enhancers or including individuals with mixed causes of dementia. We included published studies written in any language and of any duration.

Using terms from our previous review,4 the MEDLINE literature search was drafted by an experienced librarian (Dr. Laure Perrier) and revised after another librarian (Ms. Becky Skidmore) peer-reviewed the search terms.10 Subsequently, we searched the following databases: MEDLINE, EMBASE, Cochrane Methodology Register, CINAHL, Ageline and Cochrane Central Register of Controlled Trials. We also scanned reference lists of included studies and relevant reviews to supplement the electronic literature searches.

After pilot-testing, the results from the literature search were screened by pairs of reviewers working independently. Pairs of reviewers independently abstracted data (e.g., study characteristics, patient characteristics, outcome results) after a pilot-test. We resolved conflicts through discussion. The overall agreement among the reviewers for screening was over 70%.

IPD collection process and data abstraction

During the author contact process, two authors (a senior scientist ACT and a research assistant SL) sent a data request following several strategies as outlined in the RCT protocol:[1] a) an email requesting their IPD, b) email reminders (4 in total) at 2, 6, 10, and 14-week intervals after the initial email, c) reminders by post in week 7, and d) reminders via telephone in week 15. We also invited eligible authors to be a co-author on our updated systematic review provided that they share their anonymized IPD, and meet the International Committee of Medical Journal Editors (ICMJE) criteria for authorship.[2] Our team (AAV, SL) also contacted sponsors of the eligible trials, as reported in the publications. If a sponsor was not reported in a publication, we contacted the author (whom we emailed during the RCT) to determine who sponsored the study. To contact industry sponsors, we navigated the data sharing process from their websites or via an email, online portal, or phone inquiry. When no response was received, two follow-up reminders were sent to the sponsors.

We requested IPD on 1) patients: age, sex, severity of Alzheimer's disease (e.g. baseline MMSE level), presence of behavioral disturbance, comorbid conditions (e.g., stroke, cardiovascular conditions, Parkinson's disease), other medications used for each patient, number of drop-outs, reasons for drop-out, and number of participants, 2) medication: treatment each patient was allocated to, dosage, 3) outcomes: event, date of event, time taken to achieve the event for SAEs, MMSE values and measurement dates, and 4) date and method of randomization. We checked IPD provided for consistency with results from published RCTs., and contacted IPD providers when data inconsistencies were found.

Data extraction items included a) study characteristics: year of publication, country and continent according to the first author, journal in which the study was published, funding information; b) aggregate patient characteristics: study size and percentage of males, c) outcome data: study data (e.g., events or mean and standard deviations, and sample size per arm), and d) treatments compared. We also abstracted the corresponding authors' contact details. We categorized each study according to funding source (industry-sponsored, publicly-sponsored, mixed, and non-sponsored).

Certainty of the evidence

We used CINeMA (Confidence in Network Meta-Analysis) to assess confidence in the NMA estimates.[3] Six domains were evaluated with scores 'no concerns', 'some concerns' and 'major concerns': 1) within-study bias, 2) reporting bias, 3) indirectness, 4) imprecision, 5) heterogeneity, and 6) incoherence. We used the overall risk of bias per study, and for each treatment comparison we applied the average risk of bias. Similarly, for all treatment comparisons we used the average for indirectness. We assessed reporting bias based on the comparison-adjusted funnel plot since there are no established statistical methods to explore reporting bias. For imprecision, we considered a MD=1.4 and a OR=1 as a clinically important size of effect for MMSE

For peer review only - http://bmjopen.bmj.com/site/about/guidelines.xhtml

and SAE, respectively, and followed the CINeMA guidelines for exploring whether statistical significance and clinical importance coincide. Similarly, heterogeneity and incoherence (i.e. inconsistency) were assessed by following the standard CINEMA approach.

Statistical Analysis

We performed a descriptive analysis using frequencies and percentages of the discrete characteristics of the included patients and treatments of the eligible studies. We explored the distributions of the continuous patient characteristics per outcome and treatment group using means and standard deviations. For studies not providing outcome results for a certain outcome, we presented distributions of the available and requested patient characteristics, whenever available. Outliers for each patient characteristic were also explored in each study dataset using boxplots. We also recorded the number of missing participants per treatment group and overall. We compared the characteristics of the unavailable and the available by the sponsors' studies. In particular, we explored whether these were well-conducted according to overall risk of bias, and compared distributions of mean participant age, publication year, study duration, study size, percent male, and magnitude of treatment effect, to assess for potential bias in IPD sharing. We conducted a two-stage analysis for both standard meta-analysis and NMA. The network geometry was explored through the presentation of network plots.

First stage

All IPD from included studies were first aggregated to study-level summary statistics using each sponsor's portal. The use of different platforms and failure to obtain IPD from all studies restricted us from combining IPD in a one-stage analysis. For each separate study with IPD available, we fitted a logistic regression model for the binary outcome and a linear regression model for the continuous outcome. For MMSE, we considered the longest duration of follow-up per study (most frequently at week 24). In the shared IPD, when we were unable to make a judgement on first and last date of visit per patient, we used the older coded date and the newest coded date as baseline and final value for each patient respectively.

Initially, we did not adjust for any of the patient characteristics provided, but in a subsequent analysis we included patient-level covariates with as many interaction terms in the model as the patient characteristics were provided (considering only the ones we have asked for). For each study, we obtained the adjusted odds ratio (OR) for binary data and adjusted mean difference (MD) for continuous data, along their corresponding 95% confidence interval (CI). The first stage of the IPD analyses were conducted in RStudio,[4] which was available in data providers. Additional medications and comorbid conditions were grouped into broader categories according to their clinical relevance to increase power in our analysis (e.g., grouped medications as anti-psychotics, anti-depressants, and cognitive enhancers, as well as comorbid conditions as psychiatric, neurological, and cardiac disorders). Eligible studies with insufficient data to derive a pairwise estimate for NMA were summarized descriptively without performing a statistical analysis.

We applied an available case analysis for each study, since we were unable to install R packages in most sponsor-specific platforms, and hence we applied a consistent approach across all IPD datasets. We explored the impact of missing data during the second stage of analysis. Reasons for missing participants and time taken to have a serious adverse event were captured (when available).

We synthesized IPD at the first stage in four different proprietary sponsor-specific platforms. Analyses were conducted in the RStudio using different R versions[4] according to what was provided in each sponsor's platform: R version 3.4.1 for AbbVie, R version 3.4.3 for CSDR, R version 3.5.1 for YODA, R version 3.6.0 for Lundbeck.

Second stage

Since we were not successful in obtaining IPD for all eligible studies, we combined both IPD and aggregate data in a single meta-analysis or NMA model. Both IPD and aggregate data studies shared the same amount of heterogeneity. In both meta-analysis and NMA models, we combined the adjusted IPD estimates with the aggregate data (main analysis). As a secondary analysis, we combined the unadjusted estimates from retrieved IPD with the evidence provided by the aggregated data studies in a joint NMA model. A common-within network between-study variance was assumed across comparisons for all NMA models.[5] We estimated the between-study variance using the DerSimonian and Laird[6] method and compared it with the relevant distributions provided by Turner et al[7] and Rhodes et al[8] to assess heterogeneity. We also calculated I^2 on the NMA level to quantify overall heterogeneity and inconsistency in each outcome.

For peer review only - http://bmjopen.bmj.com/site/about/guidelines.xhtml

To assess the validity of the transitivity assumption for each outcome, we assessed the distribution of potential effect modifiers (e.g., age, sex) across treatment comparisons in each network.[9-11] We visually inspected similarity and assessed whether these characteristics were likely to modify the treatment effect. We evaluated the consistency assumption using the design-by-treatment interaction model[12,13] and the loop-specific method.[14,15] In the presence of statistically significant inconsistency, we checked the data for discrepancies and if none were identified, we planned to conduct subgroup NMA or network meta-regression analysis adjusting for potential variables influencing the results.

We conducted additional NMA analyses for all potential effect modifiers requested from data providers. If relevant data were not available in the IPD, we used aggregate data of the relevant publications. Additional NMA analyses included: 1) subgroup analysis for industry vs. publicly sponsored studies, and for studies with available IPD vs. studies with aggregate data (unadjusted estimates), 2) network meta-regression accounting for study duration, year of publication, mean age, and sex (% of male participants) effect modifiers separately and assuming a common regression coefficient across comparisons (studies with aggregate data were used only; studies with available IPD were pooled in a NMA separately adjusted for available covariates at first stage), 3) sensitivity analysis including studies with low risk of bias for allocation concealment and incomplete outcome data items, as these items may have an important impact on the meta-analysis results according to our previous NMA,[16] and 4) the 'informative missingness difference of means' (IMDoM) imputation method[17] for MMSE for the aggregate data studies to assess the impact of missing data in our NMA. In all additional NMA analyses, we used the adjusted effect estimates derived from the IPD within-study analysis and the aggregate data extracted from the eligible publications. Network meta-regression was performed in a Bayesian setting using OpenBUGS version 3.2.3, non-informative priors for all parameters in the model and a half-normal prior for the between standard deviation. We compared the results of the additional models by evaluating the treatment effect estimates and ranking statistics, as well as monitoring the reduction in the between-study variance.

Meta-analysis and NMA at the 2nd stage were conducted in the RStudio using R version 3.6.2 and the *meta*[18] and *netmeta*[19] packages, respectively.

References

1. Veroniki AA, Straus SE, Ashoor H, Stewart LA, Clarke M, Tricco AC. Contacting authors to retrieve individual patient data: study protocol for a randomized controlled trial. *Trials* 2016; **17**(1): 138.
2. Hager K, Baseman AS, Nye JS, et al. Effects of galantamine in a 2-year, randomized, placebo-controlled study in Alzheimer's disease. *Neuropsychiatr Dis Treat* 2014; **10**: 391-401.
3. CINeMA: Confidence in Network Meta-Analysis. Institute of Social and Preventive Medicine, University of Bern. *Available from cinemaispmch* 2017.
4. R Core Team. R: A Language and Environment for Statistical Computing. Vienna, Austria: R Foundation for Statistical Computing; 2019.
5. Lu G, Ades AE. Combination of direct and indirect evidence in mixed treatment comparisons. *Stat Med* 2004; **23**(20): 3105-24.
6. DerSimonian R, Laird N. Meta-analysis in clinical trials. *Control Clin Trials* 1986; **7**(3): 177-88.
7. Turner RM, Davey J, Clarke MJ, Thompson SG, Higgins JP. Predicting the extent of heterogeneity in meta-analysis, using empirical data from the Cochrane Database of Systematic Reviews. *Int J Epidemiol* 2012; **41**(3): 818-27.
8. Rhodes KM, Turner RM, Higgins JP. Predictive distributions were developed for the extent of heterogeneity in meta-analyses of continuous outcome data. *J Clin Epidemiol* 2015; **68**(1): 52-60.
9. Salanti G. Indirect and mixed-treatment comparison, network, or multiple-treatments meta-analysis: many names, many benefits, many concerns for the next generation evidence synthesis tool. *Res Synth Methods* 2012; **3**(2): 80-97.
10. Jansen JP, Naci H. Is network meta-analysis as valid as standard pairwise meta-analysis? It all depends on the distribution of effect modifiers. *BMC Med* 2013; **11**: 159.
11. Cipriani A, Higgins JP, Geddes JR, Salanti G. Conceptual and technical challenges in network meta-analysis. *Ann Intern Med* 2013; **159**(2): 130-7.
12. Higgins JP, Jackson D, Barrett JK, Lu G, Ades AE, White IR. Consistency and inconsistency in network meta-analysis: concepts and models for multi-arm studies. *Res Synth Methods* 2012; **3**(2): 98-110.
13. White IR BJ, Jackson D, Higgins JPT. Consistency and inconsistency in network meta-analysis: model estimation using multivariate meta-regression. *Res Synth Methods* 2012; **3**(2): 15.

For peer review only - http://bmjopen.bmj.com/site/about/guidelines.xhtml

14. Song F, Altman DG, Glenny AM, Deeks JJ. Validity of indirect comparison for estimating efficacy of competing interventions: empirical evidence from published meta-analyses. *BMJ* 2003; **326**(7387): 472.
15. Veroniki AA, Vasiliadis HS, Higgins JP, Salanti G. Evaluation of inconsistency in networks of interventions. *Int J Epidemiol* 2013; **42**(1): 332-45.
16. Veroniki AA, Straus SE, Ashoor HM, et al. Comparative safety and effectiveness of cognitive enhancers for Alzheimer's dementia: protocol for a systematic review and individual patient data network meta-analysis. *BMJ Open* 2016; **6**(1): e010251.
17. Mavridis D, White IR, Higgins JP, Cipriani A, Salanti G. Allowing for uncertainty due to missing continuous outcome data in pairwise and network meta-analysis. *Stat Med* 2015; **34**(5): 721-41.
18. Balduzzi S, Rücker G, Schwarzer G. How to perform a meta-analysis with R: a practical tutorial. *Evid Based Ment Health* 2019; **22**(4): 153-60.
19. Rücker G, Schwarzer G, Krahn U, König J. netmeta: Network Meta-Analysis using Frequentist Methods. R package version 0.9-8. 2018. https://CRAN.R-project.org/package=netmeta.

For peer review only

For peer review only - http://bmjopen.bmj.com/site/about/guidelines.xhtml

For peer review only

For peer review only - http://bmjopen.bmj.com/site/about/guidelines.xhtml

Appendix 2: Studies included in the systematic review

80 Main Studies:

1. Agid Y, Dubois B, Anand R, Gharabawi G, International Rivastigmine Investigators. Efficacy and tolerability of rivastigmine in patients with dementia of the Alzheimer type. *Current Therapeutic Research* 1998; **59**(12): 837-45.
2. Ancoli-Israel S, Amatniek J, Ascher S, Sadik K, Ramaswamy K. Effects of galantamine versus donepezil on sleep in patients with mild to moderate Alzheimer disease and their caregivers: a double-blind, head-to-head, randomized pilot study. *Alzheimer disease and associated disorders* 2005; **19**(4): 240-5.
3. Andersen F, Viitanen M, Halvorsen DS, Straume B, Wilsgaard T, Engstad TA. The effect of stimulation therapy and donepezil on cognitive function in Alzheimer's disease. A community based RCT with a two-by-two factorial design. *BMC neurology* 2012; **12**: 59.
4. Araki T, Wake R, Miyaoka T, et al. The effects of combine treatment of memantine and donepezil on Alzheimer's disease patients and its relationship with cerebral blood flow in the prefrontal area. *International journal of geriatric psychiatry* 2014; **29**(9): 881-9.
5. Bakchine S, Loft H. Memantine treatment in patients with mild to moderate Alzheimer's disease: results of a randomised, double-blind, placebo-controlled 6-month study. *Journal of Alzheimer's disease : JAD* 2008; **13**(1): 97-107.
6. Black SE, Doody R, Li H, et al. Donepezil preserves cognition and global function in patients with severe Alzheimer disease. *Neurology* 2007; **69**(5): 459-69.
7. Blesa González R, Boada Rovira M, Martínez Parra C, Gil-Saladié D, Almagro CA, Gobartt Vázquez AL. Evaluation of the convenience of changing the rivastigmine administration route in patients with Alzheimer disease. *Neurologia (Barcelona, Spain)* 2011; **26**(5): 262-71.
8. Burns A, Bernabei R, Bullock R, et al. Safety and efficacy of galantamine (Reminyl) in severe Alzheimer's disease (the SERAD study): a randomised, placebo-controlled, double-blind trial. *The Lancet Neurology* 2009; **8**(1): 39-47.
9. Burns A, Perry E, Holmes C, et al. A double-blind placebo-controlled randomized trial of Melissa officinalis oil and donepezil for the treatment of agitation in Alzheimer's disease. *Dementia and geriatric cognitive disorders* 2011; **31**(2): 158-64.
10. Burns A, Rossor M, Hecker J, et al. The effects of donepezil in Alzheimer's disease - results from a multinational trial. *Dementia and geriatric cognitive disorders* 1999; **10**(3): 237-44.
11. Choi SH, Park KW, Na DL, et al. Tolerability and efficacy of memantine add-on therapy to rivastigmine transdermal patches in mild to moderate Alzheimer's disease: a multicenter, randomized, open-label, parallel-group study. *Current medical research and opinion* 2011; **27**(7): 1375-83.
12. Corey-Bloom J. A randomized trial evaluating the efficacy and safety of ENA 713 (rivastigmine tartrate), a new acetylcholinesterase inhibitor, in patients with mild to moderately severe Alzheimer's disease. *Int J Geriatr Psyopharmacol* 1998; **1**: 55-65.
13. Creţu O, Szalontay AS, Chiriţă R, Chiriţă V. Effect of memantine treatment on patients with moderate-to-severe Alzheimer's disease treated with donepezil. *Revista medico-chirurgicala a Societatii de Medici si Naturalisti din Iasi* 2008; **112**(3): 641-5.
14. Dysken MW, Sano M, Asthana S, et al. Effect of vitamin E and memantine on functional decline in Alzheimer disease: the TEAM-AD VA cooperative randomized trial. *Jama* 2014; **311**(1): 33-44.
15. Farlow MR, Grossberg GT, Sadowsky CH, Meng X, Somogyi M. A 24-week, randomized, controlled trial of rivastigmine patch 13.3 mg/24 h versus 4.6 mg/24 h in severe Alzheimer's dementia. *CNS neuroscience & therapeutics* 2013; **19**(10): 745-52.
16. Feldman H, Gauthier S, Hecker J, Vellas B, Subbiah P, Whalen E. A 24-week, randomized, double-blind study of donepezil in moderate to severe Alzheimer's disease. *Neurology* 2001; **57**(4): 613-20.
17. Feldman HH, Lane R. Rivastigmine: a placebo controlled trial of twice daily and three times daily regimens in patients with Alzheimer's disease. *Journal of neurology, neurosurgery, and psychiatry* 2007; **78**(10): 1056-63.

For peer review only - http://bmjopen.bmj.com/site/about/guidelines.xhtml

18. Fox C, Crugel M, Maidment I, et al. Efficacy of memantine for agitation in Alzheimer's dementia: a randomised double-blind placebo controlled trial. *PloS one* 2012; **7**(5): e35185.
19. Frölich L, Ashwood T, Nilsson J, Eckerwall G. Effects of AZD3480 on cognition in patients with mild-to-moderate Alzheimer's disease: a phase IIb dose-finding study. *Journal of Alzheimer's disease : JAD* 2011; **24**(2): 363-74.
20. Fuschillo C, Ascoli E, Franzese G, et al. Alzheimer's disease and acetylcholinesterase inhibitor agents: a two-year longitudinal study. *Archives of gerontology and geriatrics Supplement* 2004; (9): 187-94.
21. Gault LM, Ritchie CW, Robieson WZ, Pritchett Y, Othman AA, Lenz RA. A phase 2 randomized, controlled trial of the α7 agonist ABT-126 in mild-to-moderate Alzheimer's dementia. *Alzheimer's & dementia (New York, N Y)* 2015; **1**(1): 81-90.
22. Gold M, Alderton C, Zvartau-Hind M, et al. Rosiglitazone monotherapy in mild-to-moderate Alzheimer's disease: results from a randomized, double-blind, placebo-controlled phase III study. *Dementia and geriatric cognitive disorders* 2010; **30**(2): 131-46.
23. Greenberg SM, Tennis MK, Brown LB, et al. Donepezil therapy in clinical practice: a randomized crossover study. *Archives of neurology* 2000; **57**(1): 94-9.
24. Grossberg GT, Manes F, Allegri RF, et al. The safety, tolerability, and efficacy of once-daily memantine (28 mg): a multinational, randomized, double-blind, placebo-controlled trial in patients with moderate-to-severe Alzheimer's disease taking cholinesterase inhibitors. *CNS drugs* 2013; **27**(6): 469-78.
25. Hager K, Baseman AS, Nye JS, et al. Effects of galantamine in a 2-year, randomized, placebo-controlled study in Alzheimer's disease. *Neuropsychiatric disease and treatment* 2014; **10**: 391-401.
26. Haig GM, Pritchett Y, Meier A, et al. A randomized study of H3 antagonist ABT-288 in mild-to-moderate Alzheimer's dementia. *Journal of Alzheimer's disease : JAD* 2014; **42**(3): 959-71.
27. Hernandez C, Unturbe F, Martinez-Lage P, Lucas A, Gregorio P, Alonso T. Effects of combined pharmacologic and cognitive treatment in the progression of moderate dementia: a two-year follow-up. *REVISTA ESPANOLA DE GERIATRIA Y GERONTOLOGIA* 2007; **42**(1): 3.
28. Herrmann N, Gauthier S, Boneva N, Lemming OM. A randomized, double-blind, placebo-controlled trial of memantine in a behaviorally enriched sample of patients with moderate-to-severe Alzheimer's disease. *International psychogeriatrics* 2013; **25**(6): 919-27.
29. Holmes C, Wilkinson D, Dean C, et al. The efficacy of donepezil in the treatment of neuropsychiatric symptoms in Alzheimer disease. *Neurology* 2004; **63**(2): 214-9.
30. Homma A, Imah Y, Hariguchi S. Late phase II clinical study of acetylcholinesterase inhibitor E 2020 in patients with alzheimer-type dementia-12-weeks double-blind, placebo-controlled study 3 mg/day, 5mg/day. *Clinical Evaluation* 1998; **26**: 251-84.
31. Homma A, Imai Y, Tago H, et al. Donepezil treatment of patients with severe Alzheimer's disease in a Japanese population: results from a 24-week, double-blind, placebo-controlled, randomized trial. *Dementia and geriatric cognitive disorders* 2008; **25**(5): 399-407.
32. Hong Z, Zhang Z, Wang L, et al. A randomzied study comparing the effect and safety of galantamine and donepezil in patients with mild to moderate Alzheimer's disease. *Chin J Neurol* 2006; **39**(6): 379-82.
33. Howard R, McShane R, Lindesay J, et al. Donepezil and memantine for moderate-to-severe Alzheimer's disease. *New England Journal of Medicine* 2012; **366**(10): 893-903.
34. Howard RJ, Juszczak E, Ballard CG, et al. Donepezil for the treatment of agitation in Alzheimer's disease. *The New England journal of medicine* 2007; **357**(14): 1382-92.
35. Hu HT, Zhang ZX, Yao JL, et al. [Clinical efficacy and safety of akatinol memantine in treatment of mild to moderate Alzheimer disease: a donepezil-controlled, randomized trial]. *Zhonghua nei ke za zhi* 2006; **45**(4): 277-80.
36. Johannsen P, Salmon E, Hampel H, et al. Assessing therapeutic efficacy in a progressive disease: a study of donepezil in Alzheimer's disease. *CNS drugs* 2006; **20**(4): 311-25.
37. Jones RW, Soininen H, Hager K, et al. A multinational, randomised, 12-week study comparing the effects of donepezil and galantamine in patients with mild to moderate Alzheimer's disease. *International journal of geriatric psychiatry* 2004; **19**(1): 58-67.

For peer review only - http://bmjopen.bmj.com/site/about/guidelines.xhtml

38. Kadir A, Darreh-Shori T, Almkvist O, et al. PET imaging of the in vivo brain acetylcholinesterase activity and nicotine binding in galantamine-treated patients with AD. *Neurobiology of aging* 2008; **29**(8): 1204-17.
39. Kano O, Ito H, Takazawa T, et al. Clinically meaningful treatment responses after switching to galantamine and with addition of memantine in patients with Alzheimer's disease receiving donepezil. *Neuropsychiatric disease and treatment* 2013; **9**: 259-65.
40. Karaman Y, Erdoğan F, Köseoğlu E, Turan T, Ersoy AO. A 12-month study of the efficacy of rivastigmine in patients with advanced moderate Alzheimer's disease. *Dementia and geriatric cognitive disorders* 2005; **19**(1): 51-6.
41. Likitjaroen Y, Meindl T, Friese U, et al. Longitudinal changes of fractional anisotropy in Alzheimer's disease patients treated with galantamine: a 12-month randomized, placebo-controlled, double-blinded study. *European archives of psychiatry and clinical neuroscience* 2012; **262**(4): 341-50.
42. Lorenzi M, Beltramello A, Mercuri NB, et al. Effect of memantine on resting state default mode network activity in Alzheimer's disease. *Drugs & aging* 2011; **28**(3): 205-17.
43. Maher-Edwards G, Dixon R, Hunter J, et al. SB-742457 and donepezil in Alzheimer disease: a randomized, placebo-controlled study. *International journal of geriatric psychiatry* 2011; **26**(5): 536-44.
44. Marek GJ, Katz DA, Meier A, et al. Efficacy and safety evaluation of HSD-1 inhibitor ABT-384 in Alzheimer's disease. *Alzheimer's & dementia : the journal of the Alzheimer's Association* 2014; **10**(5 Suppl): S364-73.
45. Mazza M, Capuano A, Bria P, Mazza S. Ginkgo biloba and donepezil: a comparison in the treatment of Alzheimer's dementia in a randomized placebo-controlled double-blind study. *European journal of neurology* 2006; **13**(9): 981-5.
46. Mohs RC, Doody RS, Morris JC, et al. A 1-year, placebo-controlled preservation of function survival study of donepezil in AD patients. *Neurology* 2001; **57**(3): 481-8.
47. Moretti DV. Alpha rhythm oscillations and MMSE scores are differently modified by transdermal or oral rivastigmine in patients with Alzheimer's disease. *American journal of neurodegenerative disease* 2014; **3**(2): 72-83.
48. Mowla A, Mosavinasab M, Haghshenas H, Borhani Haghighi A. Does serotonin augmentation have any effect on cognition and activities of daily living in Alzheimer's dementia? A double-blind, placebo-controlled clinical trial. *Journal of clinical psychopharmacology* 2007; **27**(5): 484-7.
49. Nakamura Y, Imai Y, Shigeta M, et al. A 24-week, randomized, double-blind, placebo-controlled study to evaluate the efficacy, safety and tolerability of the rivastigmine patch in Japanese patients with Alzheimer's disease. *Dementia and geriatric cognitive disorders extra* 2011; **1**(1): 163-79.
50. Nakano S, Asada T, Matsuda H, Uno M, Takasaki M. Donepezil hydrochloride preserves regional cerebral blood flow in patients with Alzheimer's disease. *Journal of nuclear medicine : official publication, Society of Nuclear Medicine* 2001; **42**(10): 1441-5.
51. Nordberg A, Darreh-Shori T, Peskind E, et al. Different cholinesterase inhibitor effects on CSF cholinesterases in Alzheimer patients. *Current Alzheimer research* 2009; **6**(1): 4-14.
52. Pakdaman H, Harandi AA, Hatamian H, et al. Effectiveness and Safety of MLC601 in the Treatment of Mild to Moderate Alzheimer's Disease: A Multicenter, Randomized Controlled Trial. *Dementia and geriatric cognitive disorders extra* 2015; **5**(1): 96-106.
53. Peng D, Xianhao X, Wang L. Efficiency and safety assessment of donepezil for treating mild and moderate Alzheimer disease. *Chinese Journal of Tissue Engineering Research* 2005; **9**(13): 170-2.
54. Peskind ER, Potkin SG, Pomara N, et al. Memantine treatment in mild to moderate Alzheimer disease: a 24-week randomized, controlled trial. *The American journal of geriatric psychiatry : official journal of the American Association for Geriatric Psychiatry* 2006; **14**(8): 704-15.
55. Peters O, Fuentes M, Joachim LK, et al. Combined treatment with memantine and galantamine-CR compared with galantamine-CR only in antidementia drug naïve patients with mild-to-moderate Alzheimer's disease. *Alzheimer's & dementia (New York, N Y)* 2015; **1**(3): 198-204.
56. Reisberg B, Doody R, Stöffler A, Schmitt F, Ferris S, Möbius HJ. Memantine in moderate-to-severe Alzheimer's disease. *The New England journal of medicine* 2003; **348**(14): 1333-41.

For peer review only - http://bmjopen.bmj.com/site/about/guidelines.xhtml

57. Rockwood K, Fay S, Song X, MacKnight C, Gorman M. Attainment of treatment goals by people with Alzheimer's disease receiving galantamine: a randomized controlled trial. *CMAJ : Canadian Medical Association journal = journal de l'Association medicale canadienne* 2006; **174**(8): 1099-105.
58. Rockwood K, Mintzer J, Truyen L, Wessel T, Wilkinson D. Effects of a flexible galantamine dose in Alzheimer's disease: a randomised, controlled trial. *Journal of neurology, neurosurgery, and psychiatry* 2001; **71**(5): 589-95.
59. Rogers SL, Doody RS, Mohs RC, Friedhoff LT. Donepezil improves cognition and global function in Alzheimer disease: a 15-week, double-blind, placebo-controlled study. Donepezil Study Group. *Archives of internal medicine* 1998; **158**(9): 1021-31.
60. Rogers SL, Farlow MR, Doody RS, Mohs R, Friedhoff LT. A 24-week, double-blind, placebo-controlled trial of donepezil in patients with Alzheimer's disease. Donepezil Study Group. *Neurology* 1998; **50**(1): 136-45.
61. Rogers SL, Friedhoff LT. The efficacy and safety of donepezil in patients with Alzheimer's disease: results of a US Multicentre, Randomized, Double-Blind, Placebo-Controlled Trial. The Donepezil Study Group. *Dementia (Basel, Switzerland)* 1996; **7**(6): 293-303.
62. Saxton J, Hofbauer RK, Woodward M, et al. Memantine and functional communication in Alzheimer's disease: results of a 12-week, international, randomized clinical trial. *Journal of Alzheimer's disease : JAD* 2012; **28**(1): 109-18.
63. Scarpini E, Bruno G, Zappalà G, et al. Cessation versus continuation of galantamine treatment after 12 months of therapy in patients with Alzheimer's disease: a randomized, double blind, placebo controlled withdrawal trial. *Journal of Alzheimer's disease : JAD* 2011; **26**(2): 211-20.
64. Schmidt R, Ropele S, Pendl B, et al. Longitudinal multimodal imaging in mild to moderate Alzheimer disease: a pilot study with memantine. *Journal of neurology, neurosurgery, and psychiatry* 2008; **79**(12): 1312-7.
65. Seltzer B, Zolnouni P, Nunez M, et al. Efficacy of donepezil in early-stage Alzheimer disease: a randomized placebo-controlled trial. *Archives of neurology* 2004; **61**(12): 1852-6.
66. Shao ZQ. Comparison of the efficacy of four cholinesterase inhibitors in combination with memantine for the treatment of Alzheimer's disease. *International journal of clinical and experimental medicine* 2015; **8**(2): 2944-8.
67. Shimizu S, Kanetaka H, Hirose D, Sakurai H, Hanyu H. Differential effects of acetylcholinesterase inhibitors on clinical responses and cerebral blood flow changes in patients with Alzheimer's disease: a 12-month, randomized, and open-label trial. *Dementia and geriatric cognitive disorders extra* 2015; **5**(1): 135-46.
68. Solé-Padullés C, Bartrés-Faz D, Lladó A, et al. Donepezil treatment stabilizes functional connectivity during resting state and brain activity during memory encoding in Alzheimer's disease. *Journal of clinical psychopharmacology* 2013; **33**(2): 199-205.
69. Tariot PN, Cummings JL, Katz IR, et al. A randomized, double-blind, placebo-controlled study of the efficacy and safety of donepezil in patients with Alzheimer's disease in the nursing home setting. *Journal of the American Geriatrics Society* 2001; **49**(12): 1590-9.
70. Tariot PN, Solomon PR, Morris JC, Kershaw P, Lilienfeld S, Ding C. A 5-month, randomized, placebo-controlled trial of galantamine in AD. The Galantamine USA-10 Study Group. *Neurology* 2000; **54**(12): 2269-76.
71. Thomas A, Iacono D, Bonanni L, D'Andreamatteo G, Onofrj M. Donepezil, rivastigmine, and vitamin E in Alzheimer disease: a combined P300 event-related potentials/neuropsychologic evaluation over 6 months. *Clinical neuropharmacology* 2001; **24**(1): 31-42.
72. Wilcock G, Howe I, Coles H, et al. A long-term comparison of galantamine and donepezil in the treatment of Alzheimer's disease. *Drugs & aging* 2003; **20**(10): 777-89.
73. Wilkinson D. A Clinical Study Evaluating the Effects of Memantine on Brain Atrophy in Patients With Alzheimer's Disease; 2012.
74. Wilkinson D, Murray J. Galantamine: a randomized, double-blind, dose comparison in patients with Alzheimer's disease. *International journal of geriatric psychiatry* 2001; **16**(9): 852-7.

For peer review only - http://bmjopen.bmj.com/site/about/guidelines.xhtml

75. Wilkinson DG, Passmore AP, Bullock R, et al. A multinational, randomised, 12-week, comparative study of donepezil and rivastigmine in patients with mild to moderate Alzheimer's disease. *International journal of clinical practice* 2002; **56**(6): 441-6.
76. Winblad B, Cummings J, Andreasen N, et al. A six-month double-blind, randomized, placebo-controlled study of a transdermal patch in Alzheimer's disease--rivastigmine patch versus capsule. *International journal of geriatric psychiatry* 2007; **22**(5): 456-67.
77. Winblad B, Engedal K, Soininen H, et al. A 1-year, randomized, placebo-controlled study of donepezil in patients with mild to moderate AD. *Neurology* 2001; **57**(3): 489-95.
78. Winblad B, Kilander L, Eriksson S, et al. Donepezil in patients with severe Alzheimer's disease: double-blind, parallel-group, placebo-controlled study. *Lancet (London, England)* 2006; **367**(9516): 1057-65.
79. Zhang Y, Rong-jia Y, Ju-ying G, H. G, Chao-mei W. Effects of aricept on the treatment of Alzheimer disease evaluated by skull multi-slice helical CT. *Chin J Clin Rehab* 2005; **9**(25): 20-1.
80. Zhang Z, Yu L, Gaudig M, Schäuble B, Richarz U. Galantamine versus donepezil in Chinese patients with Alzheimer's disease: results from a randomized, double-blind study. *Neuropsychiatric disease and treatment* 2012; **8**: 571-7.

16 Companion Reports

1. Aronson S, Van Baelen B, Kavanagh S, Schwalen S. Optimal dosing of galantamine in patients with mild or moderate Alzheimer's disease: post Hoc analysis of a randomized, double-blind, placebo-controlled trial. *Drugs & aging* 2009; **26**(3): 231-9.
2. Cummings JL, Schneider L, Tariot PN, Kershaw PR, Yuan W. Reduction of behavioral disturbances and caregiver distress by galantamine in patients with Alzheimer's disease. *The American journal of psychiatry* 2004; **161**(3): 532-8.
3. Feldman H, Gauthier S, Hecker J, et al. Economic evaluation of donepezil in moderate to severe Alzheimer disease. *Neurology* 2004; **63**(4): 644-50.
4. Feldman H, Gauthier S, Hecker J, et al. Efficacy and safety of donepezil in patients with more severe Alzheimer's disease: a subgroup analysis from a randomized, placebo-controlled trial. *International journal of geriatric psychiatry* 2005; **20**(6): 559-69.
5. Gaudig M, Richarz U, Han J, Van Baelen B, Schäuble B. Effects of galantamine in Alzheimer's disease: double-blind withdrawal studies evaluating sustained versus interrupted treatment. *Current Alzheimer research* 2011; **8**(7): 771-80.
6. Gauthier S, Feldman H, Hecker J, Vellas B, Emir B, Subbiah P. Functional, cognitive and behavioral effects of donepezil in patients with moderate Alzheimer's disease. *Current medical research and opinion* 2002; **18**(6): 347-54.
7. Grossberg GT, Farlow MR, Meng X, Velting DM. Evaluating high-dose rivastigmine patch in severe Alzheimer's disease: analyses with concomitant memantine usage as a factor. *Current Alzheimer research* 2015; **12**(1): 53-60.
8. Han HJ, Kim BC, Lee JY, et al. Response to rivastigmine transdermal patch or memantine plus rivastigmine patch is affected by apolipoprotein E genotype in Alzheimer patients. *Dementia and geriatric cognitive disorders* 2012; **34**(3-4): 167-73.
9. Jelic V, Haglund A, Kowalski J, Langworth S, Winblad B. Donepezil treatment of severe Alzheimer's disease in nursing home settings. A responder analysis. *Dementia and geriatric cognitive disorders* 2008; **26**(5): 458-66.
10. Kumar V, Anand R, Messina J, Hartman R, Veach J. An efficacy and safety analysis of Exelon in Alzheimer's disease patients with concurrent vascular risk factors. *European journal of neurology* 2000; **7**(2): 159-69.
11. Ott BR, Blake LM, Kagan E, Resnick M. Open label, multicenter, 28-week extension study of the safety and tolerability of memantine in patients with mild to moderate Alzheimer's disease. *Journal of neurology* 2007; **254**(3): 351-8.

For peer review only - http://bmjopen.bmj.com/site/about/guidelines.xhtml

12. Reisberg B, Doody R, Stöffler A, Schmitt F, Ferris S, Möbius HJ. A 24-week open-label extension study of memantine in moderate to severe Alzheimer disease. *Archives of neurology* 2006; **63**(1): 49-54.
13. Schwam E, Xu Y. Cognition and function in Alzheimer's disease: identifying the transitions from moderate to severe disease. *Dementia and geriatric cognitive disorders* 2010; **29**(4): 309-16.
14. van Dyck CH, Tariot PN, Meyers B, Malca Resnick E. A 24-week randomized, controlled trial of memantine in patients with moderate-to-severe Alzheimer disease. *Alzheimer disease and associated disorders* 2007; **21**(2): 136-43.
15. Wimo A, Winblad B, Engedal K, et al. An economic evaluation of donepezil in mild to moderate Alzheimer's disease: results of a 1-year, double-blind, randomized trial. *Dementia and geriatric cognitive disorders* 2003; **15**(1): 44-54.
16. Winblad B, Grossberg G, Frölich L, et al. IDEAL: a 6-month, double-blind, placebo-controlled study of the first skin patch for Alzheimer disease. *Neurology* 2007; **69**(4 Suppl 1): S14-22.

For peer review only

For peer review only - http://bmjopen.bmj.com/site/about/guidelines.xhtml

Appendix 3: Studies with available IPD but insufficient data to be included in the analysis

A study[1] of 859 participants comparing transdermal rivastigmine vs. placebo included only IPD for the placebo arm. Another study[2] of 285 participants comparing 22·5 mg of galantamine vs. 30 mg of galantamine vs. 45 mg of galantamine vs. placebo did not provide information about the SAE or MMSE outcomes in the shared IPD.

CSDR: Novartis (study: NVT_SA_ENA713D1301) – Nakamura 2011

The study compares rivastigmine patch vs. placebo, but includes data only on placebo. Hence, we cannot conduct an analysis to convert data on their aggregated form so that to be included in our network meta-analysis. The IPD of this study included 288 participants in total.
According to the publication, 284 were allocated to the rivastigmine patch 5 cm2 group, 287 to the rivastigmine patch 10 cm2 group, and 288 to the placebo group.

Baseline characteristics of included patients

Characteristics	PLAC	Total	Missing Data	P-value	Outliers
Males	92 (32 %)	92 (32 %)	No	-	No
Age, mean (SD)	74.6 (7.4)	74.6 (7.4)	No	-	Yes - 1 value
SAE, events/sample size	19/288	19/288	No	-	-
Baseline MMSE, mean (SD)	16.6 (2.9)	16.6 (2.9)	Yes - 1 value	-	No
MMSE, mean (SD)	17.5 (3.4)	17.5 (3.4)	No	-	No
Change score, mean (SD)	0.9 (1.6)	0.9 (1.6)	Yes - 2 values	-	Yes - 41 values
Total number of patients	288 (100 %)	288			

YODA: JNJ-Study-GAL-93-01 –Wilkinson 2001

The study compares galantamine 22.5mg, 30mg and 45mg vs placebo. In our analysis we combined galantamine 22.5mg, 30mg and 45mg in a single group. However, we only descriptively can include this study in our paper - not in the network meta-analysis – as it does not provide any info about the SAE or MMSE outcomes (only total score for baseline). The IPD of this study included 285 participants in total.
According to the publication, 285 patients were randomized to: galantamine 18mg, 24mg, 36mg/day and placebo. Of the outcomes of interest, publication reported the SAE outcome. According to the sponsor there are no differences in the reporting of doses:

- galantamine hydrobromide 7.5 mg =6 mg galantamine base was administered tid i.e galantamine hydrobromide 22.5 mg/d = galantamine base **18mg/day**
- galantamine hydrobromide 10 mg =8 mg galantamine base was administered tid i.e galantamine hydrobromide 30mg/d= galantamine base **24mg/day** and
- galantamine hydrobromide 15 mg =12 mg galantamine base was administered tid i.e galantamine hydrobromide 45mg/d= galantamine base **36mg/day**

Baseline characteristics of included patients

Characteristics	GALA	PLAC	Total	Missing Data	P-value	Outliers
Males	85 (30%)	36 (12%)	121 (42%)	No	<0.001	No
Age, mean (SD)	73.5 (8.2)	74.2 (9.0)	73.8 (8.5)	No	0.242	Yes - 1 value
SAE, events/sample size*	-	-	-	-	-	-
Baseline MMSE, mean (SD)	18.6 (3.2)	18.8 (3.1)	18.7 (3.2)	No	0.616	No
MMSE, mean (SD)	-	-	-	-	-	-
Change score, mean (SD)	-	-	-	-	-	-
Total number of patients	198 (69%)	87 (31%)	285 (100%)			

*SAE in publication is as follows, PLAC: 3/87, GALA 18mg: 6/88, GALA 24mg: 0/56, GALA 36mg: 5/54

1. Nakamura Y, Imai Y, Shigeta M, et al. A 24-week, randomized, double-blind, placebo-controlled study to evaluate the efficacy, safety and tolerability of the rivastigmine patch in Japanese patients with Alzheimer's disease. *Dement Geriatr Cogn Dis Extra* 2011; **1**(1): 163-79.
2. Wilkinson D, Murray J. Galantamine: a randomized, double-blind, dose comparison in patients with Alzheimer's disease. *Int J Geriatr Psychiatry* 2001; **16**(9): 852-7.

For peer review only - http://bmjopen.bmj.com/site/about/guidelines.xhtml

Appendix 4: List of studies requested and sponsor response

Sponsor	Author, year	Interventions compared	Sponsor Response	IPD Received
Abbvie	Gault, 2015	Placebo/No treatment, Donepezil	Available	Yes
	Haig, 2014	Placebo/No treatment, Donepezil	Available	Yes
	Marek, 2014	Placebo/No treatment, Donepezil	Unavailable (Cannot share data (Potential business considerations under review))	No
AstraZeneca	Frolich, 2011	Placebo/No treatment, Donepezil	Available	No
Daiichi-Sankyo	Shimizu, 2015	Donepezil, Galantamine, Rivastigmine	Unavailable (Do not own data)	No
Eisai	Black, 2007	Placebo/No treatment, Donepezil	Available	Yes
	Burns, 1999	Placebo/No treatment, Donepezil	Unavailable (Cannot share data (Old study))	No
	Feldman, 2001	Placebo/No treatment, Donepezil	Unavailable (Do not own data)	No
	Feldman, 2004	Placebo/No treatment, Donepezil	Unavailable (Do not own data)	No
	Feldman, 2005	Placebo/No treatment, Donepezil	Unavailable (Do not own data)	No
	Gauthier, 2002	Placebo/No treatment, Donepezil	Unavailable (Do not own data)	No
	Holmes, 2004	Placebo/No treatment, Donepezil	Unavailable (Do not own data)	No
	Homma, 2008	Placebo/No treatment, Donepezil	Unavailable (Cannot share data (Old study))	No
	Johannsen, 2006	Placebo/No treatment, Donepezil	Unavailable (Do not own data)	No
	Jones, 2004	Donepezil, Galantamine	Unavailable (Cannot share data (Old study))	No
	Mohs, 2001	Placebo/No treatment, Donepezil	Unavailable (Cannot share data (Old study))	No
	Rogers, 1996	Placebo/No treatment, Donepezil	Unavailable (Cannot share data (Old study))	No
	Rogers, 1998	Placebo/No treatment, Donepezil	Unavailable (Cannot share data (Old study))	No
	Rogers, 1998	Placebo/No treatment, Donepezil	Unavailable (Cannot share data (Old study))	No
	Schwam, 2010	Placebo/No treatment, Donepezil	Unavailable (Do not own data)	No
	Seltzer, 2004	Donepezil, Placebo/No treatment	Unavailable (Cannot share data (Old study))	No
	Shimizu, 2015	Donepezil, Galantamine, Rivastigmine	Unavailable (Do not own data)	No
	Sole-Padulles, 2013	Placebo/No treatment, Donepezil	Unavailable (Do not own data)	No
	Tariot, 2001	Placebo/No treatment, Donepezil	Unavailable (Cannot share data (Old study))	No
	Wilkinson, 2002	Donepezil, Rivastigmine	Unavailable (Do not own data)	No
Forest Laboratories/Allergen	Grossberg, 2013	Donepezil + Rivastigmine + Galantamine + Placebo, Donepezil + Rivastigmine + Galantamine + Memantine	Unavailable (Cannot share data (No details provided))	No
	Ott, 2007	Placebo/No treatment, Memantine	Unavailable (Cannot share data (No details provided))	No
	Peskind, 2006	Placebo/No treatment, Memantine	Unavailable (Cannot share data (No details provided))	No
	Saxton, 2012	Placebo/No treatment, Memantine	Unavailable (Cannot share data (No details provided))	No
	van Dyck, 2007	Placebo/No treatment, Memantine	Unavailable (Cannot share data (No details provided))	No
GlaxoSmithKline	Gold, 2010	Placebo/No treatment, Donepezil	Available	Yes
	Maher-Edwards, 2011	Placebo/No treatment, Donepezil	Unavailable (Do not own data)	No
Janssen	Ancoli-Israel, 2005	Donepezil, Galantamine	Unavailable (Cannot identify study)	No
	Aronson, 2009	Placebo/No treatment, Galantamine	Unavailable (Cannot identify study)	No
	Burns, 2009	Placebo/No treatment, Galantamine	Available	Yes
	Cummings, 2004	Placebo/No treatment, Galantamine	Available	Yes

For peer review only - http://bmjopen.bmj.com/site/about/guidelines.xhtml

Sponsor	Author, year	Interventions compared	Sponsor Response	IPD Received
	Gaudig, 2011	Placebo/No treatment, Galantamine	Unavailable (Cannot identify study)	No
	Hager K, 2014	Placebo/No treatment, Galantamine	Available	Yes
	Kadir, 2008	Placebo/No treatment, Galantamine	Unavailable (Cannot identify study)	No
	Likitjaroen, 2012	Placebo/No treatment, Galantamine	Unavailable(Do not own data)	No
	Rockwood, 2001	Placebo/No treatment, Galantamine	Available	Yes
	Rockwood, 2006	Placebo/No treatment, Galantamine	Unavailable (IPD not available)	No
	Scarpini, 2011	Placebo/No treatment, Galantamine	Unavailable (IPD not available)	No
	Shimizu, 2015	Donepezil, Galantamine, Rivastigmine	Unavailable (Cannot identify study)	No
	Tariot, 2000	Placebo/No treatment, Galantamine	Unavailable (Cannot identify study)	No
	Wilcock, 2003	Donepezil, Galantamine	Unavailable (Cannot identify study)	No
	Zhang, 2012	Donepezil, Galantamine	Unavailable (IPD not available)	No
	Wilkinson, 2001	Placebo/No treatment, Galantamine	Available	Yes
Lundbeck	Bakchine, 2008	Placebo/No treatment, Memantine	Available	Yes
	Fox, 2012	Placebo/No treatment, Memantine	Unavailable (Do not own data)	No
	Herrmann, 2013	Placebo/No treatment, Memantine	Available	Yes
	Lorenzi, 2011	Placebo/No treatment, Memantine	Unavailable (Do not own data)	No
	Wilkinson, 2012	Placebo/No treatment, Memantine	Available	Yes
Merz	Reisberg, 2003	Placebo/No treatment, Memantine	No response from sponsor	No
	Reisberg, 2006	Placebo/No treatment, Memantine	No response from sponsor	No
	Schmidt, 2008	Placebo/No treatment, Memantine	No response from sponsor	No
	Winblad, 2007	Placebo/No treatment, Rivastigmine	No response from sponsor	No
Novartis	Agid, 1998	Placebo/No treatment, Rivastigmine	Unavailable (Cannot identify study)	No
	Blesa González, 2011	Placebo/No treatment, Rivastigmine	Unavailable (Cannot share data)	No
	Choi, 2011	Placebo/No treatment, Memantine	Unavailable (Do not own data)	No
	Corey-Bloom, 1998	Placebo/No treatment, Rivastigmine	Unavailable (Cannot identify study)	No
	Farlow, 2013	Rivastigmine, Rivastigmine + Memantine	Unavailable (Cannot share data (Phase 4 study))	No
	Feldman, 2007	Placebo/No treatment, Rivastigmine	Unavailable (Cannot identify study)	No
	Grossberg, 2015	Rivastigmine, Rivastigmine + Memantine	Unavailable (Cannot share data (Phase 4 study))	No
	Han, 2012	Placebo/No treatment, Memantine	Unavailable (Cannot identify study)	No
	Kumar, 2000	Placebo/No treatment, Rivastigmine	Unavailable (Cannot identify study)	No
	Nakamura, 2011	Placebo/No treatment, Rivastigmine	Available	Yes
	Nordberg, 2009	Donepezil, Galantamine, Rivastigmine	Unavailable (Cannot share data (Phase 4 study))	No
	Shimizu, 2015	Donepezil, Galantamine, Rivastigmine	Unavailable (Cannot identify study)	No
	Winblad, 2007	Placebo/No treatment, Rivastigmine	Available	Yes
ONO	Nakamura, 2011	Placebo/No treatment, Rivastigmine	No response from sponsor	No
Pfizer	Black, 2007	Placebo/No treatment, Donepezil	Unavailable (Do not own data)	No
	Feldman, 2001	Placebo/No treatment, Donepezil	Available	No
	Feldman, 2004	Placebo/No treatment, Donepezil	Unavailable (Cannot identify study)	No
	Feldman, 2005	Placebo/No treatment, Donepezil	Unavailable (Cannot identify study)	No
	Gauthier, 2002	Placebo/No treatment, Donepezil	Unavailable (Cannot identify study)	No
	Holmes, 2004	Placebo/No treatment, Donepezil	Unavailable (Cannot identify study)	No
	Jelic, 2008	Placebo/No treatment, Donepezil	Unavailable (Cannot identify study)	No
	Johannsen, 2006	Placebo/No treatment, Donepezil	Unavailable (Cannot identify	No

For peer review only - http://bmjopen.bmj.com/site/about/guidelines.xhtml

Sponsor	Author, year	Interventions compared	Sponsor Response	IPD Received
			study)	
	Jones, 2004	Donepezil, Galantamine	Unavailable (Cannot identify study)	No
	Mohs, 2001	Placebo/No treatment, Donepezil	Unavailable (Cannot identify study)	No
	Schwam, 2010	Placebo/No treatment, Donepezil	Unavailable (Cannot identify study)	No
	Seltzer, 2004	Donepezil, Placebo/No treatment	Unavailable (Cannot identify study)	No
	Sole-Padulles, 2013	Placebo/No treatment, Donepezil	Unavailable (Cannot identify study)	No
	Tariot, 2001	Placebo/No treatment, Donepezil	Unavailable (Cannot identify study)	No
	Wilkinson, 2002	Donepezil, Rivastigmine	Unavailable (Cannot identify study)	No
	Wimo, 2003	Placebo/No treatment, Donepezil	Unavailable (Cannot identify study)	No
	Winblad, 2001	Placebo/No treatment, Donepezil	Unavailable (Cannot identify study)	No
	Winblad, 2006	Placebo/No treatment, Donepezil	Unavailable (Cannot identify study)	No
Roivant	Maher-Edwards, 2011	Placebo/No treatment, Donepezil	No response from sponsor	No
Shire Pharmaceuticals	Wilcock, 2003	Donepezil, Galantamine	Unavailable (Do not own data)	No
	Wilkinson, 2001	Placebo/No treatment, Galantamine	Unavailable (Do not own data)	No
Takeda	Shimizu, 2015	Donepezil, Galantamine, Rivastigmine	Unavailable (Do not own data)	No
Non-Pharmaceutical	Andersen, 2012	Placebo/No treatment, Donepezil	NA	No
	Araki, 2014	Placebo/No treatment, Donepezil + Memantine	NA	No
	Burns, 2011	Placebo/No treatment, Donepezil	NA	No
	Dysken, 2014	Placebo/No treatment, Memantine	Available	No
	Greenberg, 2000	Placebo/No treatment, Donepezil	Unavailable (Need to contact PI)	No
	Howard, 2007	Placebo/No treatment, Donepezil	Unavailable (Do not own data)	No
	Howard, 2012	Donepezil + Memantine , Donepezil + Placebo	Unavailable (Do not own data)	No
	Mowla, 2007	Placebo/No treatment, Rivastigmine	NA	No
	Peters, 2015	Galantamine + Placebo, Galantamine + Memantine	NA	No
Not reported	Cretu, 2008	Placebo/No treatment, Memantine	NA	No
	Fuschillo, 2001	Donepezil, Rivastigmine	NA	No
	Hernández, 2007	Placebo/No treatment, Donepezil	NA	No
	Homma, 1998	Donepezil, Placebo/no treatment	NA	No
	Hong, 2006	Placebo/No treatment, Galantamine	NA	No
	Hu, 2006	Donepezil, Memantine	NA	No
	Kano, 2013	Donepezil, Donepezil + Memantine	NA	No
	Karaman, 2005	Placebo/No treatment, Rivastigmine	NA	No
	Mazza, 2006	Placebo/No treatment, Donepezil	NA	No
	Moretti, 2014	Placebo/No treatment, Rivastigmine	NA	No
	Nakano, 2001	Placebo/No treatment, Donepezil	NA	No
	Pakdaman H, 2015	Donepezil, Galantamine, Rivastigmine	NA	No
	Peng, 2005	Placebo/No treatment, Donepezil	NA	No
	Shao, 2015	Memantine + Placebo, Rivastigmine + Memantine, Donepezil + Memantine, Galantamine + Memantine	NA	No
	Thomas, 2001	Donepezil, Rivastigmine	NA	No
	Zhang-Yi, 2005	Placebo/No treatment, Donepezil	NA	No

Abbreviations: NA, not applicable; NPH, neutral protamine Hagedorn; PI, principle investigator

For peer review only - http://bmjopen.bmj.com/site/about/guidelines.xhtml

Appendix 5: Study characteristics of the included RCTs

Study	Country of conduct	Sample size; Longest duration of follow-up (weeks)	Treatments compared; Outcomes	Funding information	Date of randomization; Date trial opened; Randomization ratio	IPD available; Reasons for not providing IPD by the data providers
Agid, 1998	12 countries - Austria, Belgium, Czechoslovakia, Denmark, Finland, France, Germany, Ireland, Norway, Sweden, Switzerland, and the UK	402; 13	Rivastigmine, Placebo/No treatment; MMSE, Nausea, Vomiting, Diarrhea, SAEs, Headaches	Industry-sponsored	Not reported; Not reported; Not reported	No; Cannot identify study
Ancoli-Israel, 2005	USA	63; 8	Galantamine, Donepezil; CIBIC-plus, Mortality, Nausea, Diarrhea, SAEs, Headaches	Industry-sponsored	Not reported; Not reported; Not reported	No; Cannot identify study
Andersen, 2012	Norway	180; 52	Donepezil, Placebo; MMSE, ADAS-cog	Publicly-sponsored	Not reported; June 2003; Not reported	No; NA
Araki, 2014	Japan	37; 24	Donepezil + Memantine, Placebo; MMSE, NPI	Publicly-sponsored	Not reported; Not reported; Not reported	No; NA
Bakchine, 2008	12 countries -Austria, Belgium, Denmark, Finland, France, Greece, Lithuania, the Netherlands, Poland, Spain, Sweden and UK	470; 24	Memantine, Placebo/no treatment; ADAS-cog, ADCS-ADL, NPI, CIBIC-plus, Mortality, SAEs, Headaches, Falls	Industry-sponsored	Not reported; Not reported; Not reported	Yes; NA
Black, 2007	5 countries - USA, Canada, France, UK, Australia	343; 24	Donepezil, Placebo/No treatment; MMSE, ADCS-ADL, NPI, CIBIC-plus, Nausea, Vomiting, Diarrhea, SAEs	Industry-sponsored	Not reported; January 2001; Not reported	Yes; Do not own data
Blesa González, 2011	Spain	139; 12	Rivastigmine Patch, Rivastigmine Oral; MMSE, Nausea, Vomiting, Diarrhea	Industry-sponsored	Not reported; Not reported; Not reported	No; Cannot share data (Phase 4 study)
Burns, 1999	Australia, Belgium, Canada, France, Germany,Ireland, New Zealand, South Africa and the UK	818; 30	Donepezil, Placebo/no treatment; ADAS-cog, CIBIC-plus, Mortality, Diarrhea, Nausea, SAEs, Vomiting	Industry-sponsored	Not reported; Not reported; Not reported	No; Cannot share data (Old study)
Burns, 2009	Belgium, Finland, France, Italy, Norway, Netherlands, Spain, Sweden, Switzerland, UK	407; 26	Galantamine, Placebo/no treatment; Mortality, Nausea, Vomiting, Diarrhea, SAEs, Headaches, Falls	Industry-sponsored	Not reported; December 2003; Not reported	Yes; NA
Burns, 2011	UK	62; 12	Donepezil, Placebo/no treatment; NPI, SAEs	Publicly-sponsored	Not reported; January 2006; Not reported	No; NA
Choi, 2011	South Korea	171; 16	Memantine, Placebo/No treatment; MMSE, ADAS-cog, ADCS-ADL, NPI, SAEs, Nausea, Diarrhea, Vomiting, Headaches	Publicly-sponsored + Industry-sponsored	Not reported; December 2008; Not reported	No; Do not own data
Corey-Bloom, 1998	USA	699; 26	Rivastigmine, Placebo/No treatment; MMSE, ADAS-cog, CIBIC-plus, Mortality, Nausea, Vomiting	Industry-sponsored	Not reported; Not reported; Not reported	No; Cannot identify study
Cretu, 2008	Romania	43;	Memantine, Placebo/No treatment;	NA	Not reported;	No;

For peer review only - http://bmjopen.bmj.com/site/about/guidelines.xhtml

		24	MMSE, ADAS-cog, NPI		Not reported;<br>Not reported	NR
Dysken, 2014	USA	307;<br>26-208	Memantine, Placebo;<br>MMSE, ADAS-cog, ADCS-ADL, NPI, Mortality, SAEs	Publicly-sponsored	Not reported;<br>August 2007;<br>1:1:1:1	No;<br>NA
Farlow, 2013	USA	716;<br>24	Rivastigmine + Memantine, Rivastigmine;<br>NPI, Mortality, Falls, Vomiting, Diarrhea, Nausea	Industry-sponsored	Not reported;<br>July 2009;<br>1:1	No;<br>Cannot share data (Phase 4 study)
Feldman, 2001	Canada, Australia, France	290;<br>24	Donepezil, Placebo/No treatment;<br>MMSE, NPI, CIBIC-plus, Mortality, Vomiting, Nausea, Diarrhea, SAEs, Headaches	Industry-sponsored	Not reported;<br>Not reported;<br>"50/50 split"	No;<br>NA
Feldman, 2007	Australia, Canada, Ireland, Italy, South Africa, UK	450;<br>26	Rivastigmine, Placebo/No treatment;<br>MMSE, ADAS-cog, CIBIC-plus, SAEs, Bradycardia, Headaches, Vomiting, Diarrhea, Nausea	Industry-sponsored	Not reported;<br>Not reported;<br>1:1:1	No;<br>Cannot identify study
Fox, 2012	UK	149;<br>12	Memantine, Placebo;<br>MMSE, NPI, Mortality	Industry-sponsored	Not reported;<br>September 2007;<br>"assigned with equal probability"	No;<br>Unavailable (Do not own data)
Frolich, 2011	Austria, Belgium, Bulgaria, Czech Republic, Germany, Romania, Russia, Spain, UK, Canada	324;<br>12	Donepezil, Placebo/No treatment;<br>MMSE, ADAS-cog, Nausea, Vomiting, Diarrhea, Headaches	Industry-sponsored	Not reported;<br>July 2007;<br>Not reported	No;<br>Available
Fuschillo, 2001	Italy	27;<br>30	Donepezil, Rivastigmine;<br>MMSE, ADAS-cog, Headaches, Vomiting, Diarrhea, Nausea	NA	Not reported;<br>Not reported;<br>Not reported	No;<br>NR
Gault, 2015	USA, Bulgaria, Czech Republic, Slovakia, UK, South Africa	136;<br>14	Donepezil, Placebo;<br>MMSE, ADAS-cog, ADCS-ADL, NPI, CIBIC-plus, Mortality, SAEs, Bradycardia, Falls, Headaches, Vomiting, Diarrhea, Nausea	Industry-sponsored	Not reported;<br>October 2009;<br>Not reported	Yes;<br>Available
Gold, 2010	Austria, Bulgaria, Chile, China, Croatia, Estonia, Germany, Greece, Hungary, Mexico, New Zealand, Pakistan, Peru, Republic of the Philippines, Puerto Rico, Republic of Korea, Russian Federation, UK and USA	248;<br>24	Donepezil, Placebo/no treatment;<br>ADAS-cog, CIBIC-plus, Mortality, Headaches, Nausea, Diarrhea, SAEs	Industry-sponsored	Not reported;<br>February 2007;<br>2:2:2:1	Yes;<br>Available
Greenberg, 2000	USA	103;<br>24	Donepezil, Placebo/no treatment;<br>ADAS-cog, SAEs, Diarrhea, Nausea	Publicly-sponsored	Not reported;<br>Not reported;<br>Not reported	No;<br>Contact PI
Grossberg, 2013	Argentina, USA, Mexico, Chile	676;<br>24	Donepezil + Rivastigmine + Galantamine + Memantine, Donepezil + Rivastigmine + Galantamine + Placebo;<br>NPI, CIBIC-plus, Mortality, Falls, Headaches, Vomiting, Diarrhea,	Industry-sponsored	Not reported;<br>June 2005;<br>1:1	No;<br>Cannot share dat

			Nausea, SAEs			
Hager K, 2014	Czech Republic, Estonia, France, Germany, Greece, Italy, Latvia, Lithuania, Romania, Russia, Slovakia, Slovenia, Ukraine	2045;<br>104	Galantamine, Placebo;<br>MMSE, Mortality, Headaches, Vomiting, Diarrhea, Nausea, SAEs	Industry-sponsored	Not reported;<br>May 2008;<br>1:1	Yes;<br>NA
Haig, 2014	Russia, Ukraine	123;<br>12	Donepezil, Placebo;<br>MMSE, ADAS-cog, ADCS-ADL, NPI, Headaches, Nausea, SAEs	Industry-sponsored	Not reported;<br>Not reported;<br>1:1:1	Yes;<br>NA
Hernández, 2007	Spain	20;<br>48	Donepezil, Placebo/No treatment;<br>MMSE, ADAS-cog	NA	Not reported;<br>Not reported;<br>Not reported	No;<br>NR
Herrmann, 2013	Canada	369;<br>24	Memantine, Placebo;<br>NPI, Mortality, Falls, Nausea, SAEs	Industry-sponsored	Not reported;<br>December 2003;<br>"equally allocated"	Yes;<br>NA
Holmes, 2004	UK	96;<br>24	Donepezil, Placebo/No treatment;<br>MMSE, NPI	Industry-sponsored	Not reported;<br>Not reported;<br>3:2	No;<br>Cannot identify study
Homma, 1998	Japan	187;<br>12	Donepezil, Placebo/no treatment;<br>ADAS-cog, Mortality, SAEs, Headaches	NA	Not reported;<br>Not reported;<br>Not reported	No;<br>NR
Homma, 2008	Japan	267;<br>24	Donepezil, Placebo/no treatment;<br>ADCS-ADL, CIBIC-plus, Mortality, SAEs, Falls, Vomiting, Diarrhea	Industry-sponsored	Not reported;<br>Not reported;<br>1:1:1	No;<br>Cannot share data (Old study)
Hong, 2006	China	218;<br>16	Galantamine, Placebo/no treatment;<br>ADAS-cog, ADCS-ADL, NPI, SAEs	NA	Not reported;<br>Not reported;<br>Not reported	No;<br>NR
Howard, 2007	England	259;<br>12	Donepezil, Placebo/No treatment;<br>MMSE, NPI, Mortality, Falls, Diarrhea	Publicly-sponsored	Not reported;<br>November 2003;<br>"probability ratios of 0.75 and 0.25 to assign treatment"	No;<br>NA
Howard, 2012	Europe	295;<br>52	Donepezil + Placebo, Donepezil + Memantine;<br>MMSE, Mortality, SAEs, Falls	Publicly-sponsored	Not reported;<br>February 2008;<br>Not reported	No;<br>Do not own data
Hu, 2006	China	97;<br>16	Memantine, Donepezil;<br>MMSE	NA	Not reported;<br>Not reported;<br>Not reported	No;<br>NA
Johannsen, 2006	Belgium, Denmark, Germany, Greece, Hungary, Iceland, The Netherlands, Poland, USA	202;<br>48	Donepezil, Placebo/No treatment;<br>MMSE, ADAS-cog, NPI, Headaches, Diarrhea, Nausea	Industry-sponsored	Not reported;<br>February 1999;<br>Not reported	No;<br>Do not own data
Jones, 2004	UK, Finland, Germany and Norway	120;<br>12	Donepezil, Galantamine;<br>MMSE, ADAS-cog, Headaches, Vomiting, Diarrhea, Nausea, SAEs	Industry-sponsored	Not reported;<br>Not reported;<br>1:1	No;<br>Cannot share data (Old study)
Kadir, 2008	Sweden	18;<br>48	Galantamine, Placebo/No treatment;<br>MMSE, ADAS-cog	Industry-sponsored + Other	Not reported;<br>Not reported;<br>Not reported	No;<br>Cannot identify study

For peer review only

For peer review only - http://bmjopen.bmj.com/site/about/guidelines.xhtml

Kano, 2013;	Japan	30;<br>28	Donepezil, Donepezil + Memantine ;<br>MMSE	NA	Not reported;<br>August 2011;<br>Not reported	No;<br>NR
Karaman, 2005	Turkey	44;<br>52	Rivastigmine, Placebo/No treatment;<br>MMSE, ADAS-cog, ADAS-ADL, CIBIC-plus, Headaches, Vomiting, Nausea	NA	Not reported;<br>Not reported;<br>Not reported	No;<br>NR
Likitjaroen, 2012	Germany	25;<br>26	Galantamine, Placebo;<br>MMSE	Publicly-sponsored + Industry-sponsored	Not reported;<br>September 2006;<br>Not reported	No;<br>Do not own data
Lorenzi, 2011	Italy	15;<br>24	Memantine, Placebo/No treatment;<br>MMSE	Publicly-sponsored + Industry-sponsored	Not reported;<br>Not reported;<br>Not reported	No;<br>Do not own data
Maher-Edwards, 2011	Austria, Bulgaria, Chile, Estonia, Germany, Russia, Slovakia, and UK	129;<br>24	Donepezil, Placebo/no treatment;<br>ADAS-cog, CIBIC-plus, Mortality, SAEs, Headaches, Nausea	Industry-sponsored	Not reported;<br>May 2006;<br>1:1:1	No;<br>No response from sponsor
Marek, 2014	UK, Ukraine, South Africa, Russia	132;<br>16	Donepezil, Placebo;<br>MMSE, ADAS-cog, NPI, CIBIC-plus, Mortality, Headaches, Vomiting, Diarrhea, SAEs	Industry-sponsored	Not reported;<br>May 2010;<br>"equal proportions"	No;<br>Cannot share data
Mazza, 2006	Italy	51;<br>24	Donepezil, Placebo/No treatment;<br>MMSE	NA	Not reported;<br>March 2003;<br>1:1:1	No;<br>NR
Mohs, 2001	USA	431;<br>54	Donepezil, Placebo/No treatment;<br>MMSE, Mortality, SAEs, Headaches, Diarrhea, Nausea	Industry-sponsored	Not reported;<br>Not reported;<br>Not reported	No;<br>Cannot share data
Moretti, 2014	Italy	20;<br>78	Rivastigmine Patch, Rivastigmine Oral;<br>MMSE	NA	Not reported;<br>Not reported;<br>Not reported	No;<br>NA
Mowla, 2007	Iran	81;<br>12	Rivastigmine, Placebo/No treatment;<br>MMSE	Publicly-sponsored	Not reported;<br>Not reported;<br>Not reported	No;<br>NA
Nakamura, 2011	Japan	855;<br>24	Rivastigmine, Placebo/No treatment;<br>MMSE, SAEs, Vomiting, Nausea, Diarrhea	Industry-sponsored	Not reported;<br>January 2007;<br>Not reported	Yes;<br>NA
Nakano, 2001	Japan	35;<br>48	Donepezil, Placebo/No treatment;<br>MMSE	NA	Not reported;<br>Not reported;<br>Not reported	No;<br>NR
Nordberg, 2009	USA	63;<br>13	Rivastigmine, Donepezil, Galantamine;<br>SAEs, Headaches, Vomiting, Diarrhea, Nausea	Industry-sponsored	Not reported;<br>Not reported;<br>1:1:1	No;<br>Cannot share data
Pakdaman H, 2015	Iran	198;<br>68.8	Donepezil, Galantamine, Rivastigmine;<br>MMSE, ADAS-cog, Mortality, Headaches, Vomiting, Diarrhea,	Industry-sponsored	Not reported;<br>Not reported;<br>Not reported	No;<br>NR

For peer review only

			Nausea			
Peng, 2005	China	89;<br>12	Donepezil, Placebo/No treatment;<br>MMSE	NA	Not reported;<br>1998;<br>Not reported	No;<br>NR
Peskind, 2006	USA	403;<br>24	Memantine, Placebo/no treatment;<br>ADAS-cog, ADCS-ADL, NPI, CIBIC-plus, Nausea, Vomiting, Diarrhea	Industry-sponsored	Not reported;<br>Not reported;<br>Not reported	No;<br>Cannot share data
Peters, 2015	Europe	226;<br>52	Galantamine + Memantine, Galantamine + Placebo;<br>ADAS-cog, ADCS-ADL, NPI, Mortality, SAEs, Falls	Publicly-sponsored	Not reported;<br>Not reported;<br>Not reported	No;<br>NA
Reisberg, 2003	USA	252;<br>28	Memantine, Placebo/No treatment;<br>MMSE, ADCS-ADL, NPI, CIBIC-plus, Mortality, SAEs, Diarrhea	Publicly-sponsored + Industry-sponsored	Not reported;<br>August 1998;<br>Not reported	No;<br>No response from sponsor
Rockwood, 2001	Australia, Canada, Great Britian, New Zealand, South Africa, USA	386;<br>12	Galantamine, Placebo/no treatment;<br>ADAS-cog, NPI, CIBIC-plus, Mortality, SAEs, Vomiting, Nausea	Industry-sponsored	Not reported;<br>Not reported;<br>Not reported	Yes;<br>NA
Rockwood, 2006	Canada	130;<br>16	Galantamine, Placebo/no treatment;<br>ADAS-cog, CIBIC-plus, SAEs, Vomiting, Nausea	Publicly-sponsored + Industry-sponsored	Not reported;<br>November 2001;<br>Not reported	No;<br>IPD not available
Rogers, 1996	USA	161;<br>12	Donepezil, Placebo/No treatment;<br>MMSE, ADAS-cog, Headaches, Diarrhea	Industry-sponsored	Not reported;<br>Not reported;<br>Not reported	No;<br>Cannot share data
Rogers, 1998	USA	468;<br>12	Donepezil, Placebo/No treatment;<br>MMSE, ADAS-cog, CIBIC-plus, SAEs, Headaches, Vomiting, Diarrhea, Nausea	Industry-sponsored	Not reported;<br>Not reported;<br>Not reported	No;<br>Cannot share data
Rogers, 1998	USA	473;<br>24	Donepezil, Placebo/No treatment;<br>MMSE, ADAS-cog, CIBIC-plus, Mortality, SAEs, Vomiting, Diarrhea, Nausea	Industry-sponsored	Not reported;<br>Not reported;<br>Not reported	No;<br>Cannot share data
Saxton, 2012	Australia, South Africa, New Zealand	264;<br>12	Memantine, Placebo;<br>Mortality, Falls, Headaches, Diarrhea, Nausea, SAEs	Industry-sponsored	Not reported;<br>April 2007;<br>Not reported	No;<br>Cannot share data
Scarpini, 2011	Italy	139;<br>96	Galantamine, Placebo/no treatment;<br>Mortality, SAEs	Industry-sponsored	Not reported;<br>July 2001;<br>Not reported	No;<br>IPD not available
Schmidt, 2008	Europe	36;<br>52	Memantine, Placebo/No treatment;<br>MMSE, ADAS-cog, ADCS-ADL	Industry-sponsored	Not reported;<br>Not reported;<br>Not reported	No;<br>No response from sponsor
Seltzer, 2004	USA	153;<br>24	Donepezil, Placebo/No treatment;<br>MMSE, ADAS-cog, Diarrhea, Nausea	Industry-sponsored	Not reported;<br>Not reported;<br>Not reported	No;<br>Cannot identify study
Shao, 2015	China	110;	Donepezil + Memantine, Galantamine	NA	Not reported;	No;

For peer review only - http://bmjopen.bmj.com/site/about/guidelines.xhtml

		24	+ Memantine, Memantine + Placebo, Rivastigmine + Memantine; MMSE, ADCS-ADL		October 2009; Not reported	NR
Shimizu, 2015	Japan	75; 52	Donepezil, Galantamine, Rivastigmine; MMSE, ADAS-cog, NPI, Headaches, Vomiting, Nausea	Industry-sponsored	Not reported; Not reported; Not reported	No; Do not own data
Sole-Padulles, 2013	Spain	14; 13	No treatment, Donepezil; MMSE, NPI	Industry-sponsored	Not reported; Not reported; Not reported	No; Do not own data
Tariot, 2000	USA	978; 20	Galantamine, Placebo/no treatment; ADAS-cog, ADCS-ADL, NPI, Mortality, SAEs, Vomiting, Diarrhea, Nausea	Industry-sponsored	Not reported; Not reported; Not reported	No; Cannot identify study
Tariot, 2001	USA	208; 24	Donepezil, Placebo/No treatment; MMSE, Mortality, SAEs, Bradycardia, Headaches, Vomiting, Diarrhea, Nausea	Industry-sponsored	Not reported; Not reported; Not reported	No; Cannot identify study
Thomas, 2001	Italy	40; 24	Donepezil, Rivastigmine; MMSE, ADAS-cog	NA	Not reported; Not reported; Not reported	No; NR
Wilcock, 2003	UK	188; 52	Galantamine, Donepezil; MMSE, ADAS-cog, Mortality, SAEs, Falls, Headaches, Vomiting, Nausea	Industry-sponsored	Not reported; June 2000; Not reported	No; Cannot identify study
Wilkinson, 2001	UK	180; 12	Galantamine, Placebo/no treatment; ADAS-cog, SAEs, Headaches, Vomiting, Diarrhea, Nausea	Industry-sponsored	Not reported; May 1994; Not reported	Yes; NA
Wilkinson, 2002	UK, South Africa, and Switzerland	111; 12	Donepezil, Rivastigmine; MMSE, ADAS-cog, Mortality, SAEs, Bradycardia, Headaches, Vomiting, Nausea	Industry-sponsored	Not reported; Not reported; 1:1	No; Cannot identify study
Wilkinson, 2012	France, Germany, Switzerland, UK	277; 52	Memantine, Placebo/No treatment; MMSE, NPI, Mortality, SAEs, Falls	Industry-sponsored	Not reported; September 2005; 1:1	Yes; NA
Winblad, 2001	Denmark, Finland, Norway, Sweden, the Netherlands	286; 52	Donepezil, Placebo/No treatment; MMSE, SAEs, Bradycardia, Headaches, Diarrhea, Nausea	Industry-sponsored	Not reported; Not reported; Not reported	No; Cannot identify study
Winblad, 2006	Sweden	248; 24	Donepezil, Placebo/No treatment; MMSE, NPI, Mortality, SAEs, Falls, Diarrhea, Nausea	Industry-sponsored	Not reported; October 2002; Not reported	No; Cannot identify study
Winblad, 2007	Chile, Czech Republic, Denmark, Finland, Germany, Guatemala, Israel, Italy, Korea, Mexico, Norway, Peru, Poland, Portugal, Russia, Slovak Republic, Sweden, Taiwan, USA, Uruguay, Venezuela	1190; 24	Rivastigmine, Placebo/No treatment; MMSE, ADAS-cog, ADCS-ADL, NPI, Mortality, SAEs, Headaches, Vomiting, Diarrhea, Nausea	Industry-sponsored	Not reported; November 2003; Not reported	No; No response from sponsor
Zhang-Yi, 2005	China	120; 8	Donepezil, Placebo/No treatment; MMSE	NA	Not reported; Not reported;	No; NR

For peer review only

For peer review only - http://bmjopen.bmj.com/site/about/guidelines.xhtml

					Not reported	
Zhang, 2012	China	218;<br>16	Galantamine, Donepezil;<br>MMSE, ADAS-cog, ADCS-ADL, NPI, Mortality, Vomiting, Diarrhea, Nausea, SAEs	Industry-sponsored	Not reported;<br>Not reported;<br>Not reported	No;<br>IPD not available

For peer review only - http://bmjopen.bmj.com/site/about/guidelines.xhtml

For peer review only

Appendix 6. Characteristics of studies with shared IPD

Study	Provided by	Severity of AD*	Previous response to treatment for AD	Presence of behavioural disturbance	Comorbid conditions	Other medications used	Treatment Group	Males (%)	Age, mean (SD)
Black 2007	CSDR - EISAI	Severe	NR	NR	All patients included the same exact comorbidities	NR	Donepezil	48 (27%)	78 (7.9)
							Placebo	54 (32%)	78 (8.1)
Gold 2010	CSDR - GSK	Mild-Moderate	NR	NR	Multiple reported	Multiple reported	Donepezil	16 (29%)	76.6 (8.2)
							Placebo	49 (46%)	75.5 (8.2)
Winblad 2007	CSDR - Novartis	Mild-Moderate	NR	NR	Multiple reported	Multiple reported	Rivastigmine patch	198 (33 %)	73.9 (8.0)
							Rivastigmine oral	102 (34 %)	72.9 (8.2)
							Placebo	101 (33%)	73.8 (7.5)
Hager 2014	YODA - Janssen	Mild-Moderate	NR	NR	NR	Multiple reported	Galantamine	354 (34%)	73 (8.9)
							Placebo	367 (36%)	73 (8.7)
Rockwood 2001	YODA - Janssen	Mild-Moderate	NR	NR	NR	Multiple reported	Galantamine	113 (43%)	75 (7.3)
							Placebo	58 (46%)	75 (7.6)
Cummings 2004	YODA - Janssen	NR	NR	NR	Multiple reported	Multiple reported	Galantamine	245 (35%)	76.9 (7.8)
							Placebo	108 (38%)	77.2 (7.9)
Burns 2009	YODA - Janssen	Severe	NR	NR	Multiple reported	Multiple reported	Galantamine	42 (20%)	84.0 (6.5)
							Placebo	39 (19%)	83.8 (6.7)
Gault 2015	AbbVie	Mild-Moderate	NR	NR	NR	Multiple reported	Donepezil	37 (54%)	72.4 (8.4)
							Placebo	26 (38%)	73.6 (8.2)
Haig 2014	AbbVie	Mild-Moderate	NR	NR	Multiple reported	Multiple reported	Donepezil	24 (40%)	70 (8.3)
							Placebo	24 (38%)	70 (7.8)
Bakchine 2008	Lundbeck	Mild-Moderate	NR	NR	NR	Multiple reported	Memantine	112 (35%)	74 (7.4)
							Placebo	61 (40%)	73 (6.9)
Herrman 2013	Lundbeck	69 (48%)	NR	NR	NR	Multiple reported	Memantine	77 (42%)	75 (7.9)
							Placebo	77 (41%)	75 (6.9)
Wilkinson 2012	Lundbeck	NR	NR	NR	NR	Multiple reported	Memantine	50 (38%)	74 (8.8)
							Placebo	69 (48%)	74 (7.8)

Additional characteristics of studies with shared IPD

Study	Patients experiencing at least one SAE	Missing data in SAE outcome	Baseline MMSE, mean (SD)	Final MMSE, mean (SD)	Change score, mean (SD)	Missing data in MMSE outcome	Total number of patients	Reasons for dropouts as indicated in the provided IPD	Time taken for the 1st SAE
Black 2007	21	0 (0%)	7.5 (3.3)	8.2 (5.2)	0.63 (3.1)	27 (15%)	176 (51%)	• intercurrent illness (1 [2%] – donepezil = 1; placebo = 0),<br>• request of patient or investigator (4 [7%] –	617 days (range [110, 1292])

For peer review only - http://bmjopen.bmj.com/site/about/guidelines.xhtml

	25	0 (0%)	7.4 (3.6)	7.6 (4.8)	-0.15 (3.5)	27 (16%)	167 (49%)	donepezil = 3; placebo = 1),<br>• patient entered nursing home/facility (5 [9%] – donepezil = 1; placebo =) 4,<br>• due to adverse experience (30 [56%] – donepezil = 15; placebo = 15), and<br>• other (14 [26%] – donepezil = 7; placebo = 7)	691 days (range [78, 1475]).
Gold 2010	6	0 (0%)	20 (3.7)	21 (4.6)	1.11 (2.3)	18 (32%)	56 (34%)	• Adverse Event (16 [39%] – donepezil = 9; placebo = 7),<br>• Lost to Follow-Up (4 [10%] – donepezil = 3; placebo = 1),<br>• Non-compliance (6 [15%] – donepezil = 2; placebo = 4),<br>• Subject decided to withdraw (11 [26%] – donepezil = 4; placebo = 7)	349 days (range [48, 656])
	10	0 (0%)	20.1 (4.2)	20.4 (5.4)	0.08 (2.7)	23 (22%)	107 (66%)		492 days (range [95, 780])
Winblad 2007	83	0 (0%)	16.6 (3.0)	17.7 (4.7)	1 (3.4)	74 (10%)	598 (50 %)	NR	NR
	37	0 (0%)	16.4 (3.1)	17.2 (4.6)	0.8 (3.2)	31 (12%)	297 (25 %)	NR	NR
	45	0 (0%)	16.4 (3.0)	16.4 (5.3)	-0.1 (3.6)	21 (7%)	302 (25 %)	NR	NR
Hager 2014	73	0 (0%)	19.0 (4.1)	17.81 (6.2)	-1.38 (4.3)	228 (22%)	1027 (50%)	NR	NR
	92	0 (0%)	19.0 (4.0)	16.99 (6.3)	-2.15 (4.4)	236 (23%)	1022 (50%)	NR	NR
Rockwood 2001	27	0 (0%)	23.2 (5.2)	NR	NR	NR	261 (68%)	NR	NR
	5	0 (0%)	22.9 (5.0)	NR	NR	NR	125 (32%)	NR	NR
Cummings 2004	23	0 (0%)	20.7 (4.9)	NR	NR	NR	692 (71%)	NR	NR
	81	0 (0%)	20.6 (4.9)	NR	NR	NR	286 (29%)	NR	NR
Burns 2009	62	0 (0%)	NR	9.2 (4.5)†	NR	NR	211 (51%)	NR	NR
	75	0 (0%)	NR	9.6 (4.9)†	NR	NR	204 (49%)	NR	NR
Gault 2015	5	0 (0%)	19.2 (4.1)	20.7 (5.1)	1.5 (2.6)	48 (71%)	68 (50%)	NR	305 days (range [224, 377])
	3	0 (0%)	18.8 (4)	18.9 (4.8)	0.1 (2.4)	45 (66%)	68 (50%)	NR	239 days (range [206, 295])
Haig 2014	2	0 (0%)	17.9 (4.2)	19.7 (3.9)	1.2 (2.8)	41 (68%)	60 (49%)	NR	286 days (range N/A – a single date was provided)
	1	0 (0%)	17.8 (3.8)	19.9 (4.2)	1.8 (1.8)	47 (75%)	63 (51%)	NR	270 days (range [161, 379]).
Bakchine 2008	33	0 (0%)	18.7 (3.3)	NR	NR	NR	318 (68%)	NR	NR
	9	0 (0%)	18.9 (3.2)	NR	NR	NR	152 (32%)	NR	NR
Herrman 2013	18	0 (0%)	11.9 (3.1)	11.3 (4.9)	-0.76 (3.4)	31 (8%)	182 (49%)	NR	NR
	11	0 (0%)	11.8 (2.9)	11.1 (4.7)	-0.68 (3.2)	32 (9%)	187 (51%)	NR	NR

For peer review only - http://bmjopen.bmj.com/site/about/guidelines.xhtml

Wilkinson 2012	17	0 (0%)	16.7 (2.5)	16.4 (5.2)	-0.46 (3.9)	30 (11%)	133 (48%)	NR	NR
	20	0 (0%)	17.1 (2.4)	16.4 (5.6)	-0.69 (4.0)	30 (11%)	144 (52%)	NR	NR

* According to publication
† The MMSE final value comes from visit 8 (last available visit in IPD). MMSE was not reported in study publication
Abbreviations: AD, Alzheimer's Dementia; IPD, individual patient data; MMSE, Mini-Mental State Examination; NR, not reported; N/A, not applicable; SAE, serious adverse event

For peer review only

Appendix 7: Comparison of studies with shared IPD with (a) all remaining studies and (b) studies for which sponsors claimed unavailable IPD. AD: aggregate data; IPD: individual patient data

a. Comparison of studies with shared IPD with all remaining studies (irrespective type of sponsor)

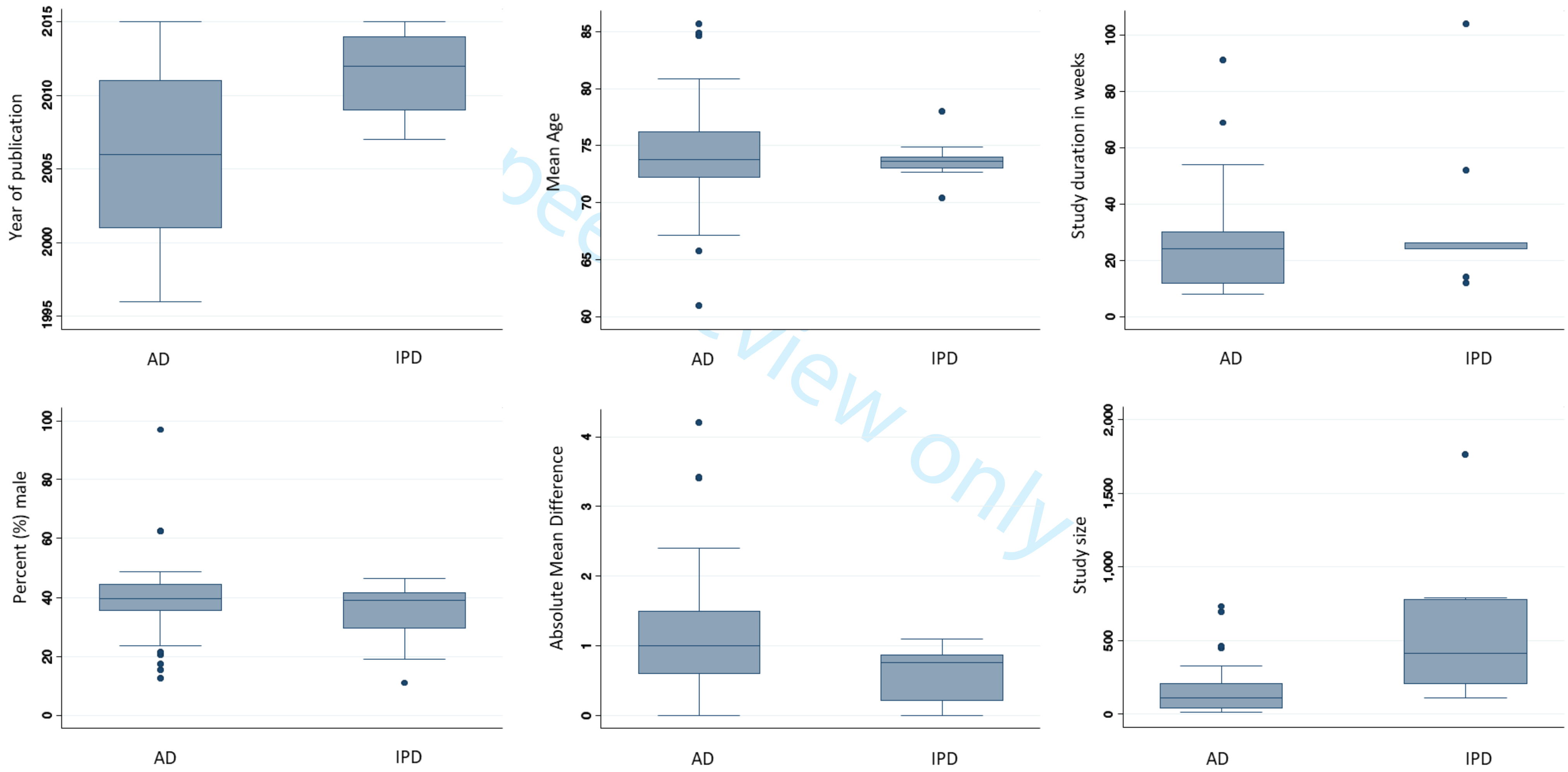

For peer review only - http://bmjopen.bmj.com/site/about/guidelines.xhtml

b. Comparison of studies with available and unavailable IPD (industry-sponsored studies only)

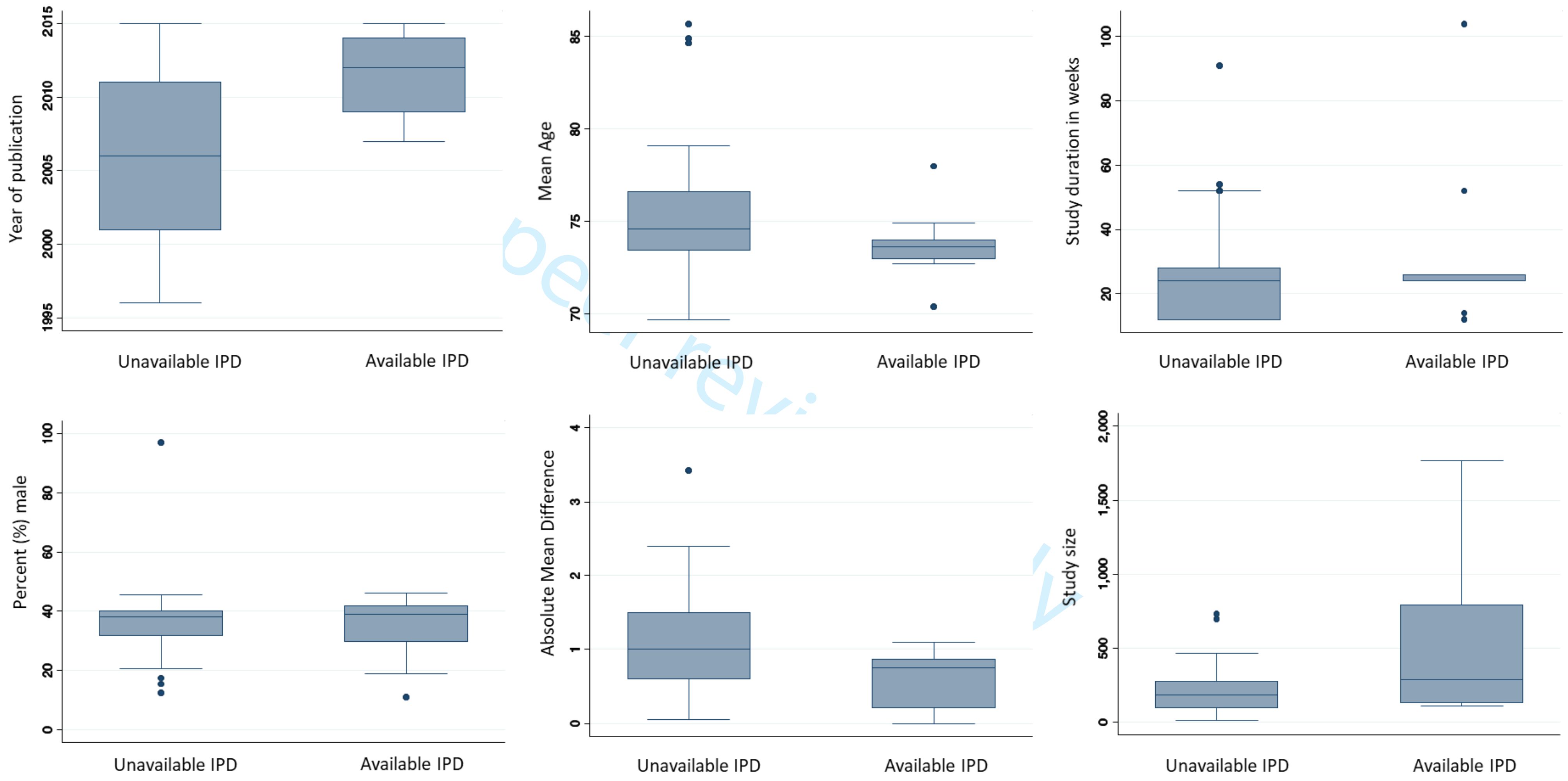

Appendix 8: Cochrane Risk-of-bias appraisal results (n = 80)

Study	1. Random sequence generation	2. Allocation concealment	3. Blinding of participants and personnel	4. Blinding of outcome assessment	5. Incomplete outcome data	6. Selective reporting	7. Other bias
Agid, 1998	Low	High	Low	Unclear	High	Unclear	High
Ancoli-Israel, 2005	Unclear	Unclear	Unclear	Unclear	High	Unclear	High
Andersen, 2012	Unclear	Low	Low	Low	High	Low	Low
Araki, 2014	Low	Unclear	Unclear	Unclear	High	Unclear	Unclear
Bakchine, 2008	Low	Low	Low	Low	Low	High	High
Black, 2007	Low	Low	Low	Low	Low	Unclear	High
Blesa Gonzalez, 2011	Unclear	Unclear	High	Unclear	High	Low	High
Burns, 1999	Unclear	Unclear	Unclear	Unclear	High	Unclear	High
Burns, 2009	Low	Low	Low	Low	Low	Unclear	High
Burns, 2011	Low	Unclear	Low	Low	High	Unclear	Unclear
Choi, 2011	Unclear	Unclear	High	High	High	Low	Low
Corey-Bloom, 1998	Low	Low	Low	Low	High	Unclear	High
Cretu, 2008	Unclear	Unclear	Unclear	Low	Unclear	Unclear	Unclear
Dysken, 2014	Low	Low	Low	Unclear	Low	Low	Low
Farlow, 2013	Low	Unclear	Low	Low	High	Unclear	High
Feldman, 2001	Low	Unclear	Low	Low	High	Unclear	High
Feldman, 2007	Low	Low	Low	Low	High	Unclear	High
Fox, 2012	Low	Low	High	Low	High	High	Unclear
Frolich, 2011	Unclear	Unclear	Low	Low	High	Low	High
Fuschillo, 2001	Unclear	Unclear	Unclear	Unclear	Low	Low	Unclear
Gault, 2015	Low	Low	Low	Unclear	Low	Low	High
Gold, 2010	Low	Unclear	Low	Low	High	Low	High
Greenberg, 2000	Low	Low	Low	Unclear	High	Low	Low
Grossberg, 2013	Low	Low	Low	Low	High	Low	High
Hager K, 2014	Low	Low	Low	Low	High	High	High
Haig, 2014	Low	Low	Low	Low	High	Low	High
Hernández, 2007	Low	Low	Low	Low	Unclear	Low	Low
Herrmann, 2013	Low	Low	Low	Low	High	Low	High
Holmes, 2004	Low	Unclear	Low	Low	High	Low	High
Homma, 1998	Low	Low	Low	Low	Low	Unclear	High
Homma, 2008	Low	Low	Low	Low	High	Unclear	Unclear
Hong, 2006	Unclear	Unclear	Unclear	Unclear	Low	Unclear	Unclear
Howard, 2007	Low	Low	Low	Low	Low	Unclear	Low
Howard, 2012	Low	Low	Low	Low	High	Low	Low
Hu, 2006	Unclear	Unclear	Unclear	Unclear	Low	Unclear	Unclear
Johannsen, 2006	Unclear	Unclear	Low	Low	Low	Unclear	High
Jones, 2004	Low	Unclear	Unclear	Low	Low	Unclear	High
Kadir, 2008	Unclear	Unclear	Unclear	Unclear	High	Unclear	High
Kano, 2013	Unclear	Unclear	Unclear	Unclear	Low	Unclear	Unclear
Karaman, 2005	Low	Unclear	Low	Low	Unclear	Unclear	Unclear
Likitjaroen, 2012	Low	Low	Low	Unclear	High	High	Unclear
Lorenzi, 2011	Unclear	Unclear	Unclear	Unclear	Unclear	Unclear	High
Maher-Edwards, 2011	Low	Unclear	Unclear	Unclear	High	Unclear	High
Marek, 2014	Low	Low	Low	Low	High	Low	High
Mazza, 2006	Low	Unclear	Low	Low	High	Unclear	Unclear
Mohs, 2001	Low	Low	Low	Low	High	Unclear	High
Moretti, 2014	Unclear	Unclear	Unclear	Unclear	Low	Unclear	Low
Mowla, 2007	Low	Unclear	Low	Unclear	High	Unclear	Unclear
Nakamura, 2011	Unclear	Low	Low	Low	Low	Low	High
Nakano, 2001	Unclear	Unclear	Unclear	Unclear	Unclear	Unclear	Unclear
Nordberg, 2009	Unclear	Unclear	High	High	Unclear	Unclear	High
Pakdaman H, 2015	Low	Unclear	High	High	High	Unclear	Unclear
Peng, 2005	Unclear	Unclear	Unclear	Unclear	Low	Unclear	Unclear
Peskind, 2006	Low	Low	Low	Unclear	Low	Unclear	High
Peters, 2015	Unclear	Unclear	Low	Low	High	Low	Low
Reisberg, 2003	Low	Unclear	Low	Unclear	High	Low	Unclear
Rockwood, 2001	Low	Low	Low	Low	Unclear	Low	High
Rockwood, 2006	Low	Low	Low	Low	Low	Unclear	Unclear
Rogers, 1996	Unclear	Unclear	Low	Unclear	Low	Unclear	Unclear
Rogers, 1998	Unclear	Unclear	Low	Low	Low	Unclear	High
Rogers, 1998	Low	Unclear	Low	Unclear	High	Unclear	High
Saxton, 2012	Low	Low	Low	Low	Low	Low	High
Scarpini, 2011	Low	Low	Low	Unclear	High	Unclear	High
Schmidt, 2008	Low	Low	Low	Low	High	Unclear	High
Seltzer, 2004	Low	Unclear	Unclear	Unclear	Unclear	Unclear	High

For peer review only - http://bmjopen.bmj.com/site/about/guidelines.xhtml

Shao, 2015	Unclear	Unclear	Unclear	Unclear	Low	Unclear	Unclear
Shimizu, 2015	Low	Unclear	High	Low	High	Unclear	Unclear
Sole-Padulles, 2013	Unclear	Unclear	Unclear	Unclear	Low	Unclear	Unclear
Tariot, 2000	Low	Unclear	Low	Low	High	Low	High
Tariot, 2001	Low	Low	Low	Low	Unclear	Unclear	High
Thomas, 2001	Low	Unclear	Unclear	Unclear	Unclear	Unclear	Unclear
Wilcock, 2003	Unclear	Unclear	Unclear	Unclear	Unclear	Unclear	Unclear
Wilkinson, 2001	Low	Low	Low	Low	High	Unclear	High
Wilkinson, 2002	Low	Low	Low	Low	High	Unclear	High
Wilkinson, 2012	Low	High	Low	Low	High	Low	High
Winblad, 2001	Low	Unclear	Unclear	Low	High	Unclear	High
Winblad, 2006	Low	Low	Low	Low	High	Low	High
Winblad, 2007	Low	Low	Low	Low	High	Unclear	High
Yi, 2005	Unclear	Unclear	Unclear	Unclear	Low	Unclear	Unclear
Zhang, 2012	Unclear	Unclear	Unclear	Unclear	High	Unclear	High

For peer review only

For peer review only - http://bmjopen.bmj.com/site/about/guidelines.xhtml

Appendix 9: Overall risk of bias for studies with shared IPD against (a) all remaining studies and (b) studies for which sponsors claimed unavailable IPD. AD: aggregate data; IPD: individual patient data

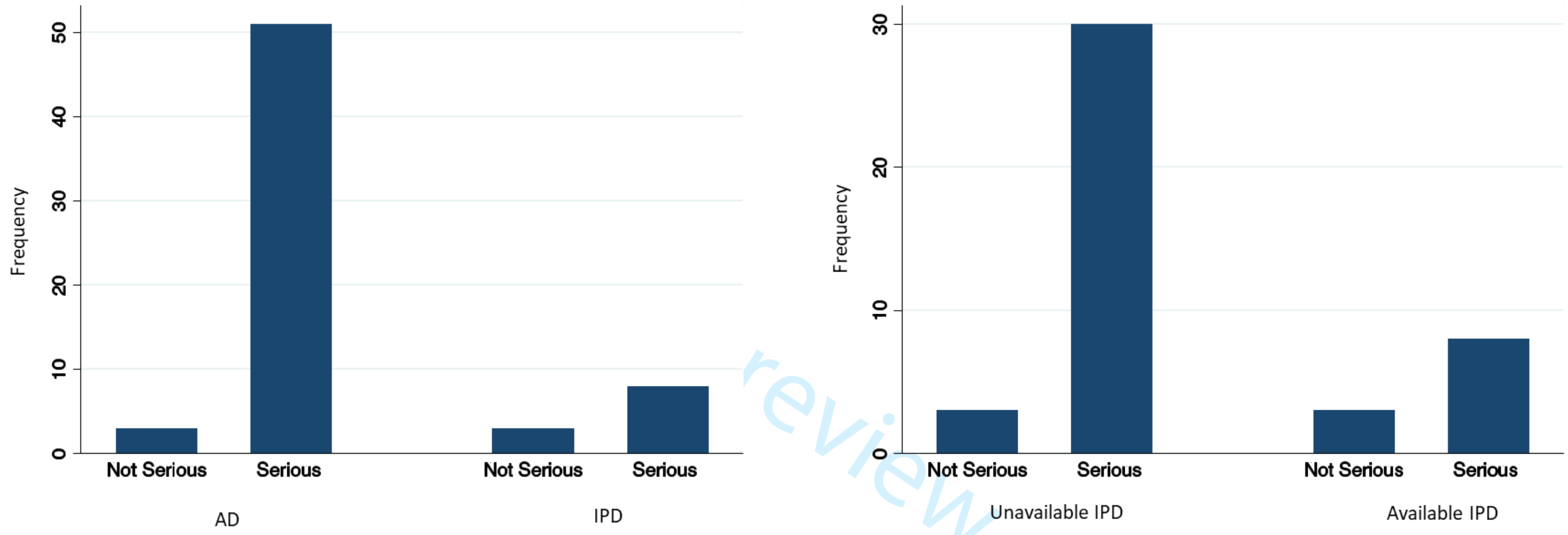

a. Comparison of studies with shared IPD with all remaining studies (irrespective type of sponsor)

b. Comparison of studies with available and unavailable IPD (industry-sponsored studies only)

For peer review only - http://bmjopen.bmj.com/site/about/guidelines.xhtml

Appendix 10: Study-specific effect sizes calculated from shared IPD and published data. IPD: individual patient data

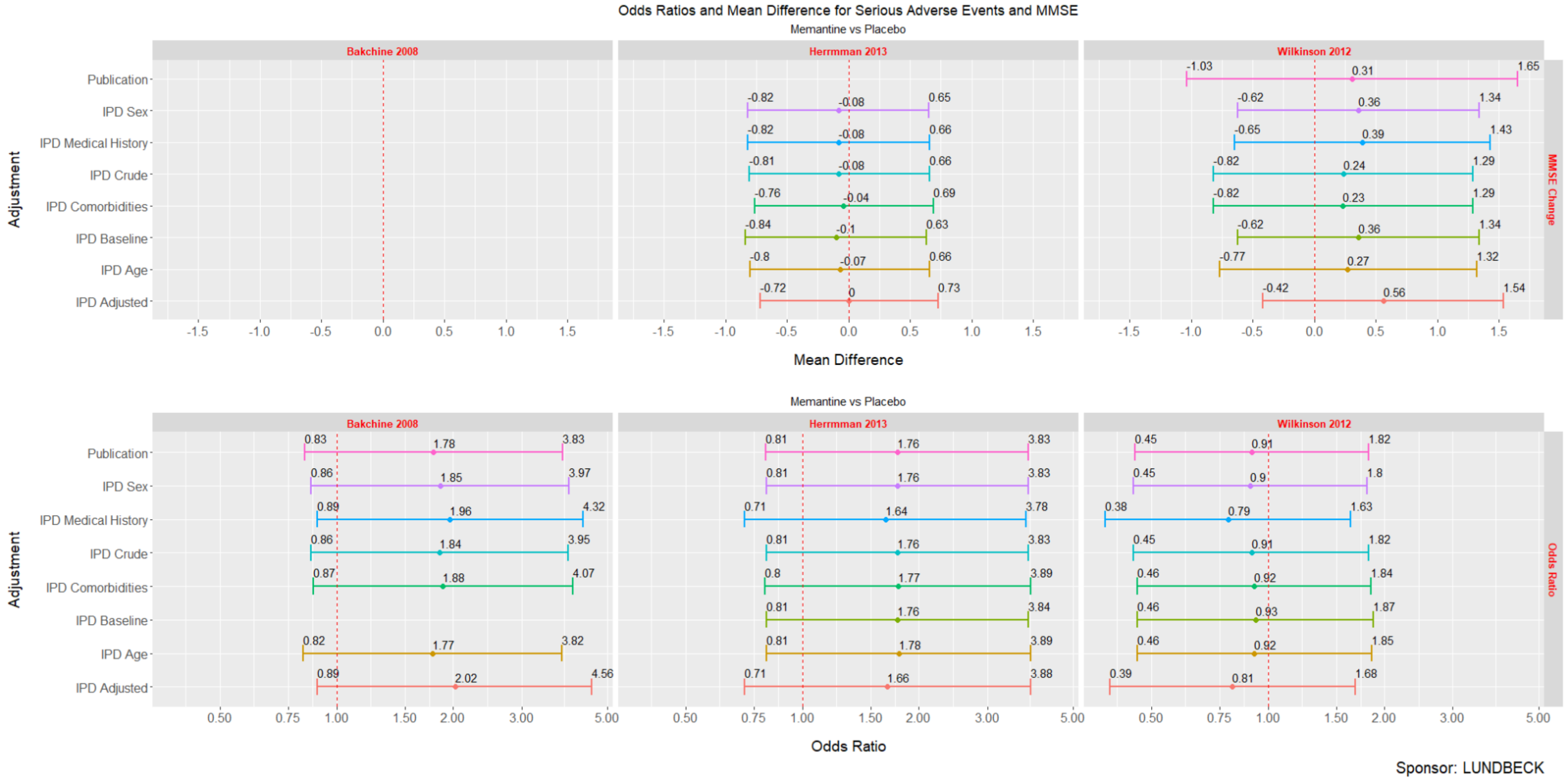

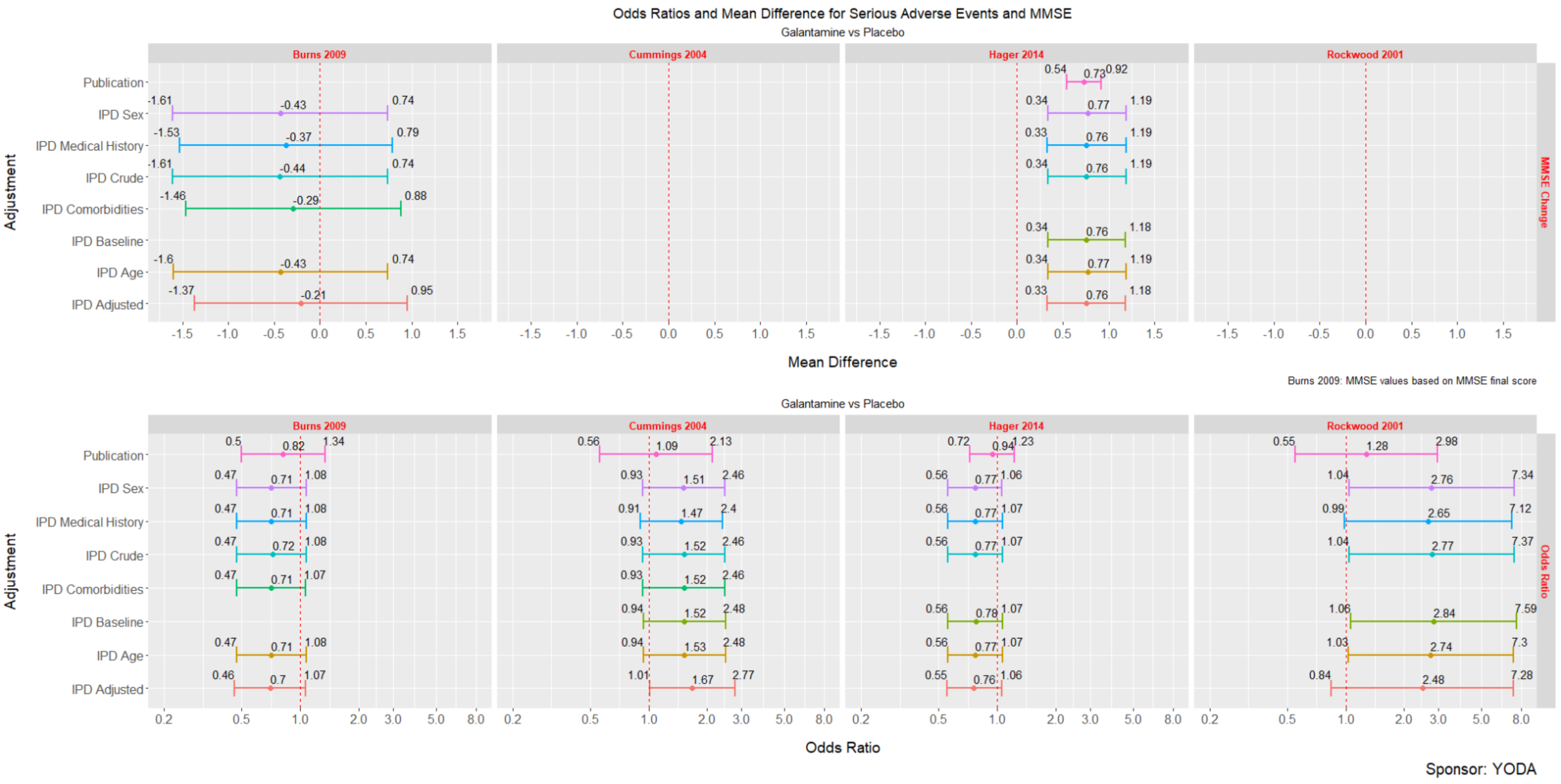
Odds Ratios and Mean Difference for Serious Adverse Events and MMSE
Galantamine vs Placebo
Burns 2009
Cummings 2004
Hager 2014
Rockwood 2001
Publication
IPD Sex
IPD Medical History
IPD Crude
IPD Comorbidities
IPD Baseline
IPD Age
IPD Adjusted
Adjustment
MMSE Change
Mean Difference
Burns 2009: MMSE values based on MMSE final score
Galantamine vs Placebo
Odds Ratio
Sponsor: YODA

33

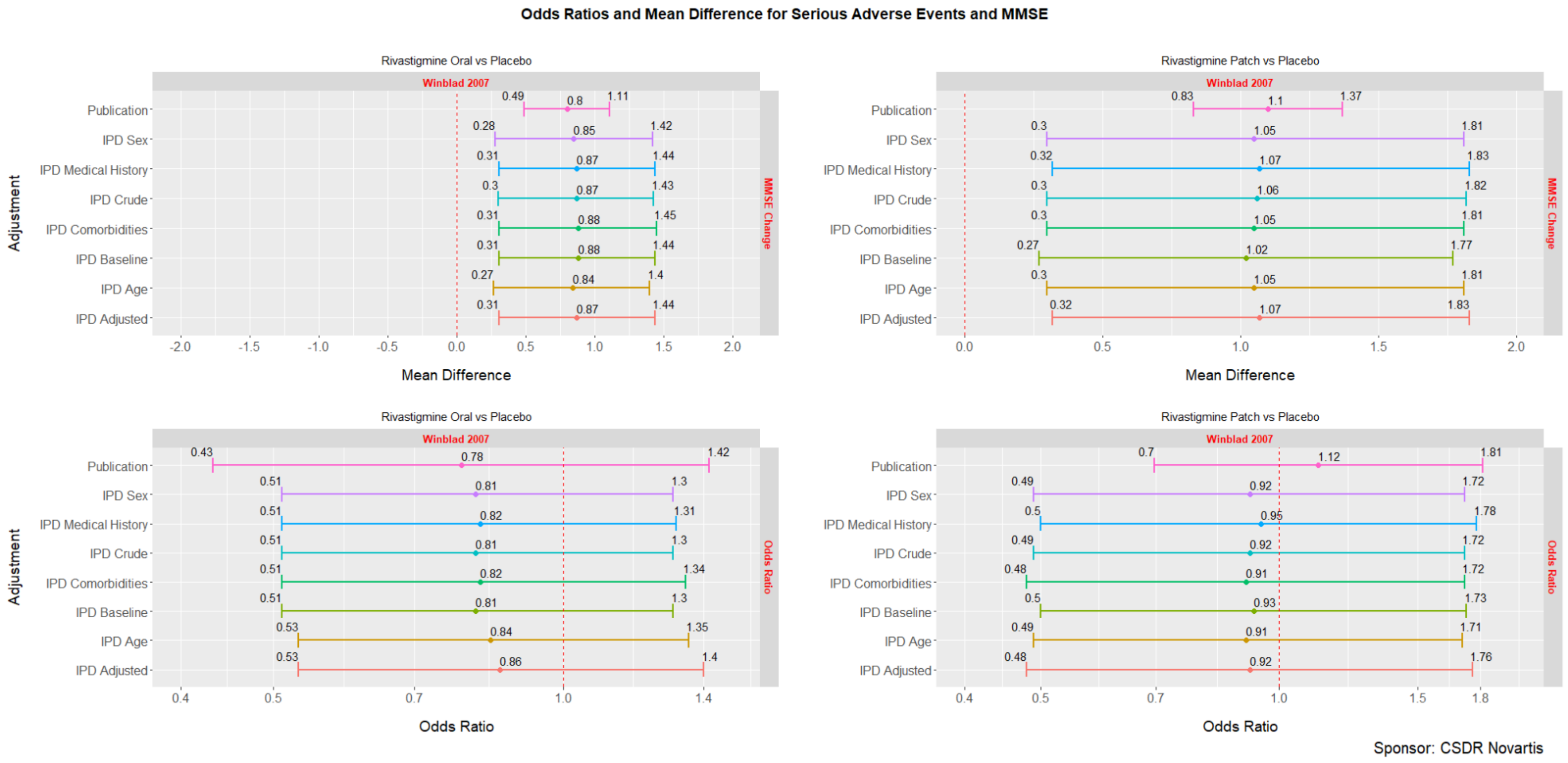
Odds Ratios and Mean Difference for Serious Adverse Events and MMSE
Rivastigmine Oral vs Placebo
Winblad 2007
Rivastigmine Patch vs Placebo
Publication
IPD Sex
IPD Medical History
IPD Crude
IPD Comorbidities
IPD Baseline
IPD Age
IPD Adjusted
Adjustment
MMSE Change
Mean Difference
Odds Ratio
Sponsor: CSDR Novartis

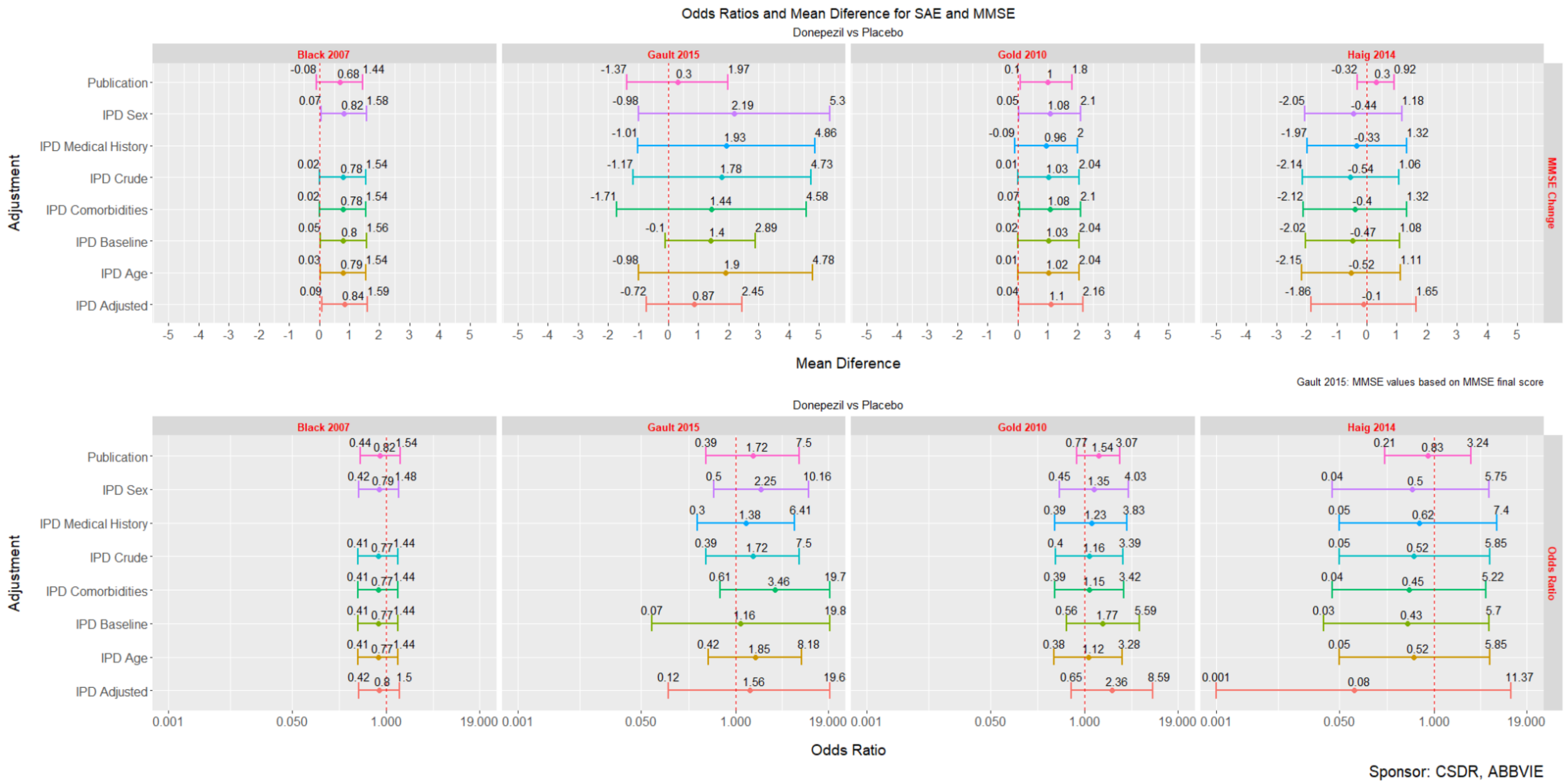

CSDR includes studies sponsored by GlaxoSmithKline, Eisai, Novartis, whereas YODA includes studies sponsored by Janssen

We also calculated the odds ratio for patients experiencing at least one SAE excluding missing participants as shown in the MMSE outcome: Gold 2010: OR 2.78, 95% CI: 0.63-12.25; Black 2007: OR 1.19, 95% CI: 0.08-17.96; Winbland 2007: rivastigmine oral, OR 1.28, 95% CI: 0.09-18.16, rivastigmine patch, OR 0.81, 95% CI: 0.02-33.59; Wilkinson 2012: OR 0.84, 95% CI: 0.38-1.86; Herrmman 2013: OR 1.70, 95% CI: 0.71-4.08; Bachine 2008: OR 1.83, 95% CI: 0.77-4.32.

We were unable to assess this for studies obtained through YODA and AbbVie, since at the time of this assessement we did not have access to these data.

Abbreviations: IPD sex, regression analysis adjusting for sex; IPD medical history, regression analysis adjusting for medical history; IPD crude, analysis with no adjustments; IPD comorbidities, regression analysis adjusting for comorbidities; IPD baseline, regression analysis adjusting for MMSE baseline; IPD age, regression analysis adjusting for age; IPD adjusted, regression analysis adjusting for all available variables (we only considered those that we initially requested from sponsor)

For peer review only

Appendix 11: Correlation between participant age and dropout in studies with IPD. IPD: individual patient data

	Study*	Correlation	P-Value
**CSDR**	Black 2007 (EISAI)	0.079	0.147
	Gold 2010 (GSK)	0.141	0.072
	Winblad 2007 (Novartis)	0.016	0.584
**Lundbeck**	Wilkinson 2012	0.066	0.273
	Herrmman 2013	0.124	0.017

* We were unable to assess this correlation for studies obtained through YODA and AbbVie, since at the time of this assessment we did not have access to these data

For peer review only

For peer review only - http://bmjopen.bmj.com/site/about/guidelines.xhtml

Appendix 12: Comparison Adjusted Funnel plot (all treatments vs placebo)

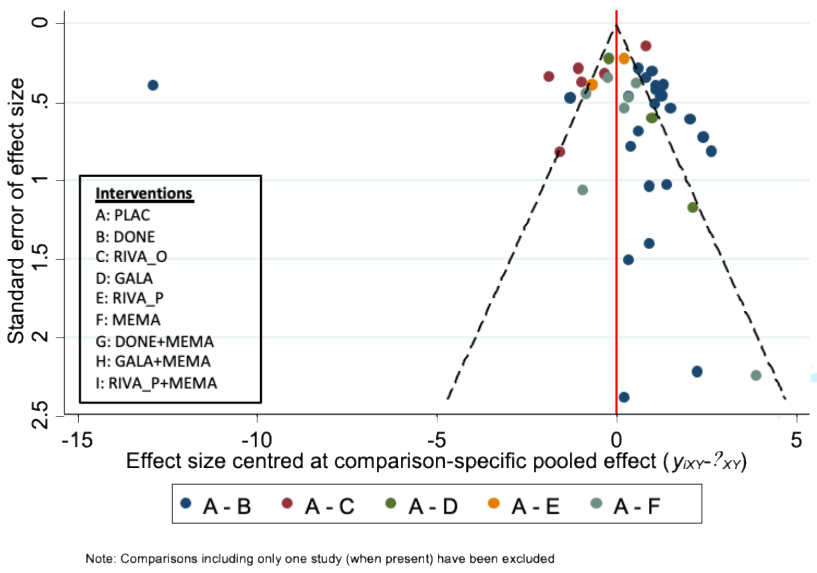

MMSE

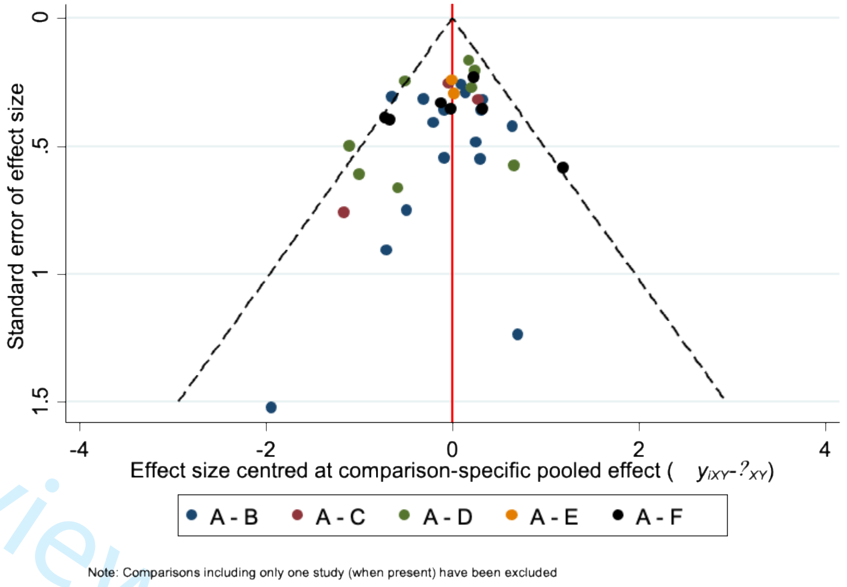

Serious Adverse Events

For peer review only - http://bmjopen.bmj.com/site/about/guidelines.xhtml

Appendix 13: Distribution of potential effect modifiers per treatment comparison and outcome

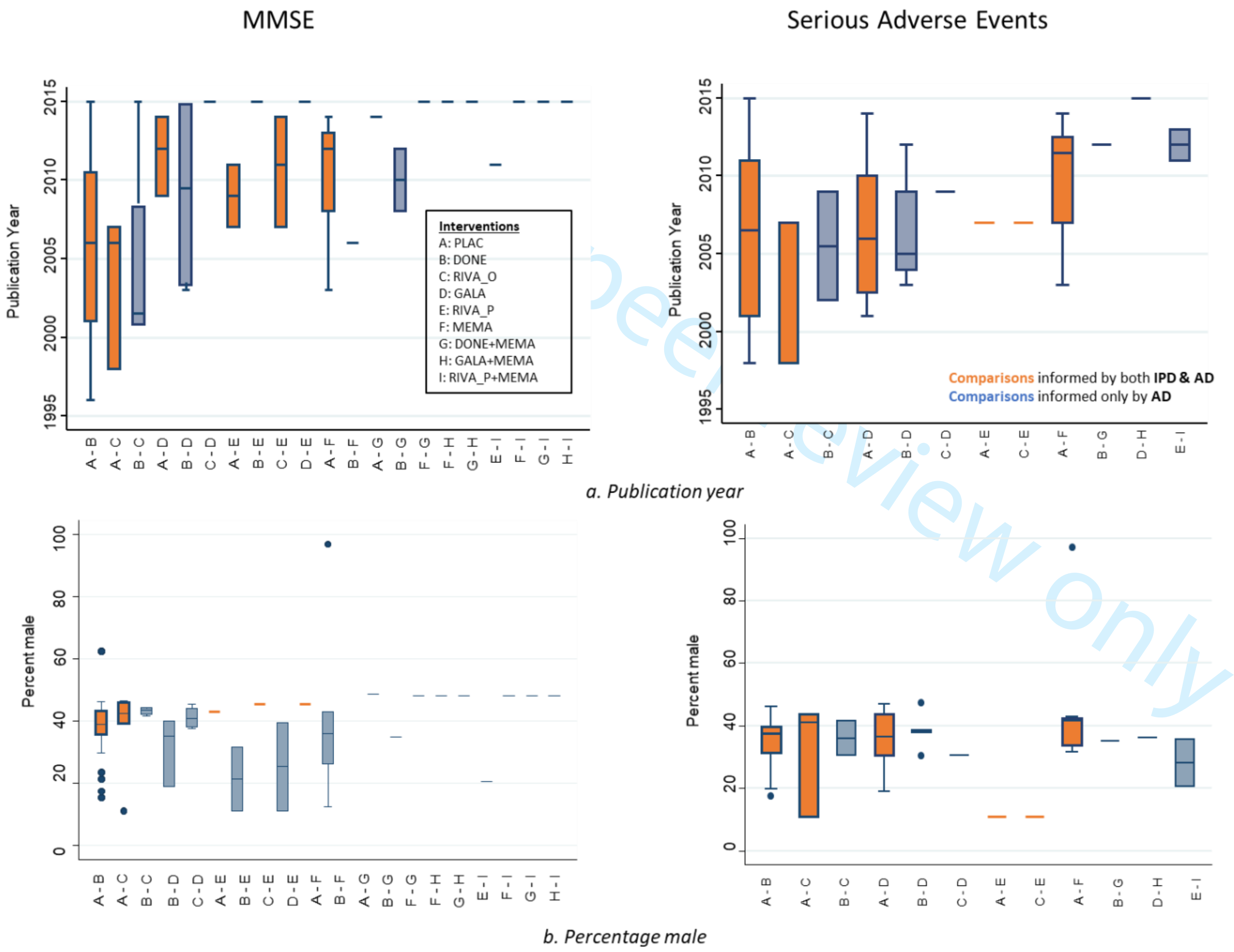

a. Publication year

b. Percentage male

For peer review only - http://bmjopen.bmj.com/site/about/guidelines.xhtml

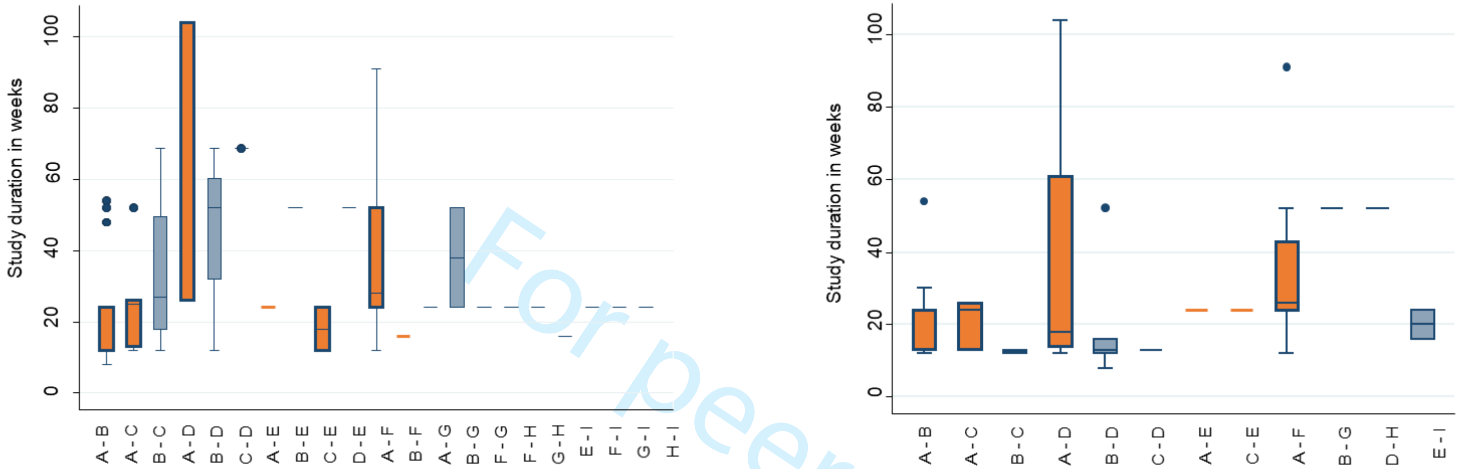

c. Study duration

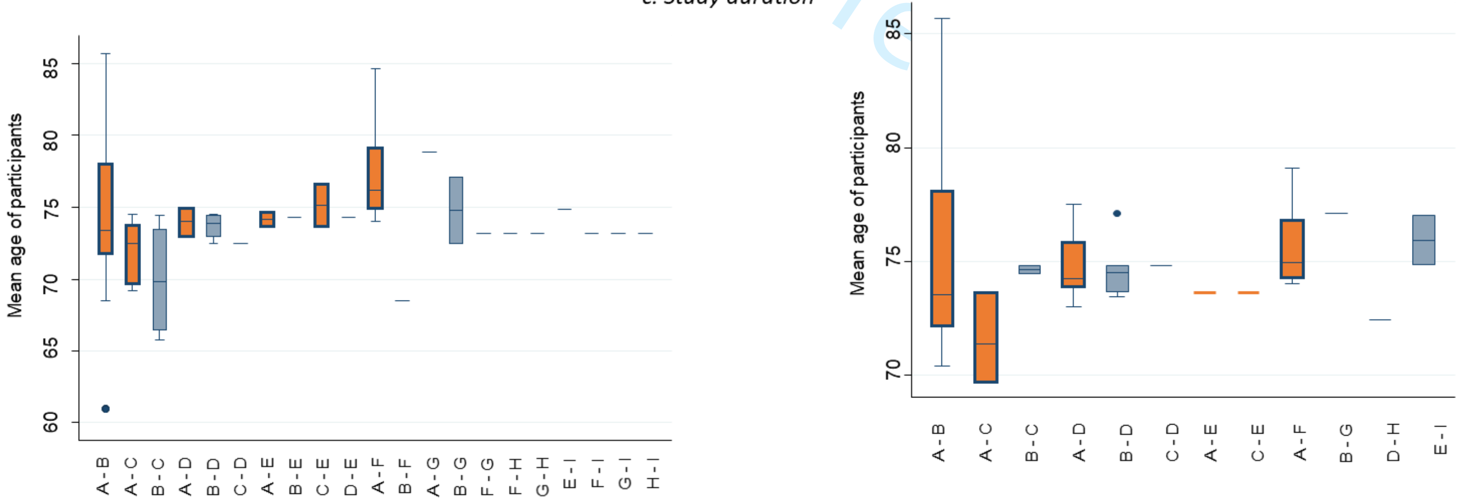

d. Mean participant age

For peer review only - http://bmjopen.bmj.com/site/about/guidelines.xhtml

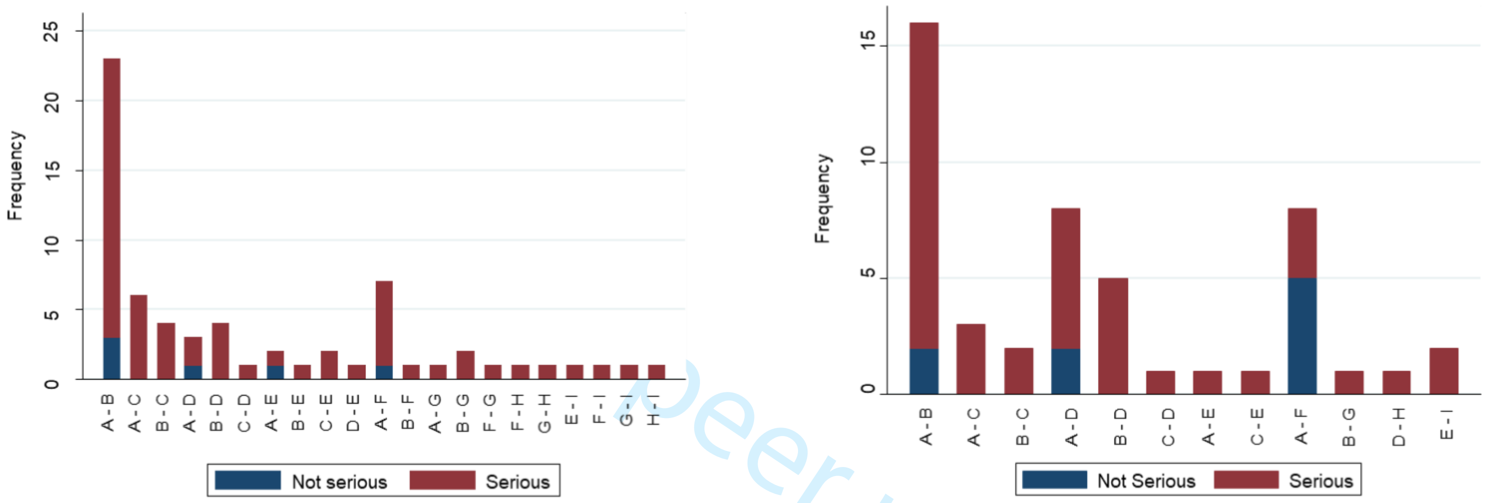

e. Overall Risk of Bias

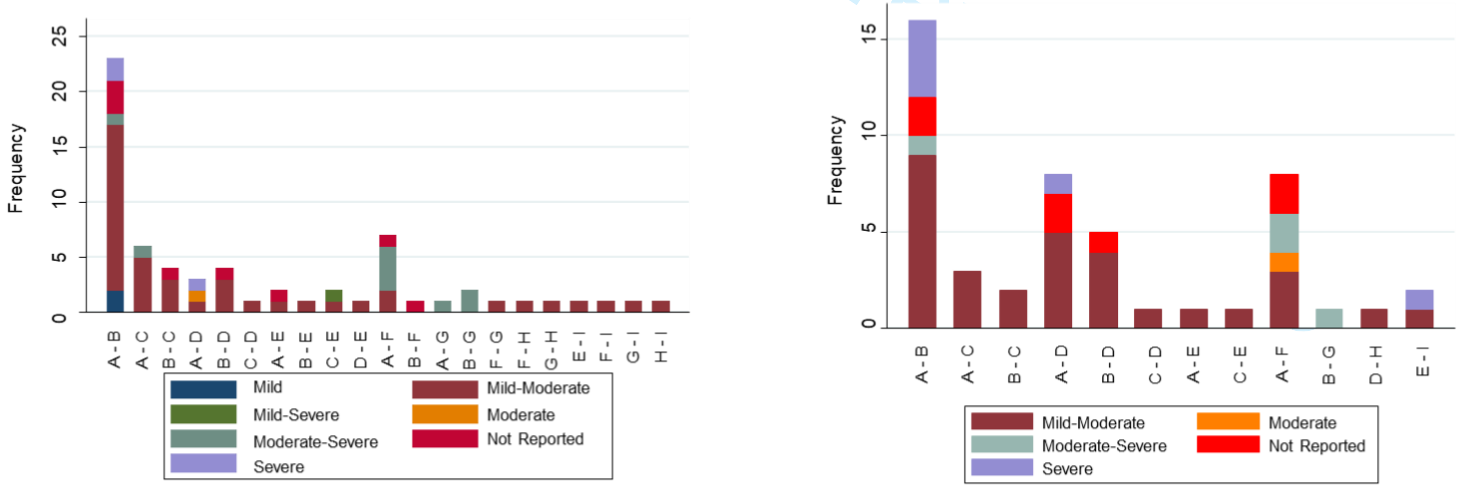

f. Alzheimer's Dementia Severity

Appendix 14: Consistency Assessment – Loop-specific approach (using adjusted treatment effects)

MMSE

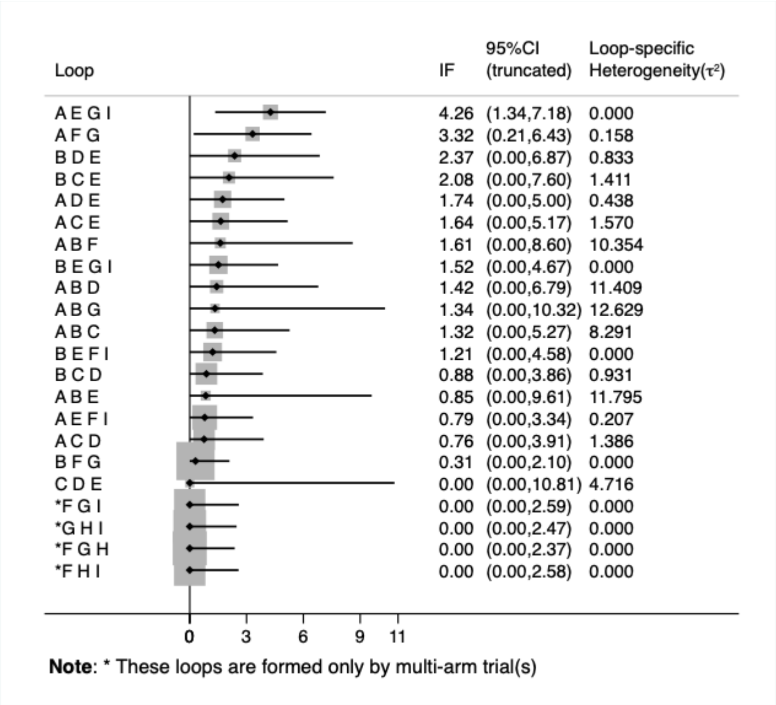

Design-by-treatment interaction model:

χ^2 statistic: 4.36, 13 degrees of freedom, P value: 0.987, between-study variance: 7.34. I^2 statistic=96%

Serious Adverse Events

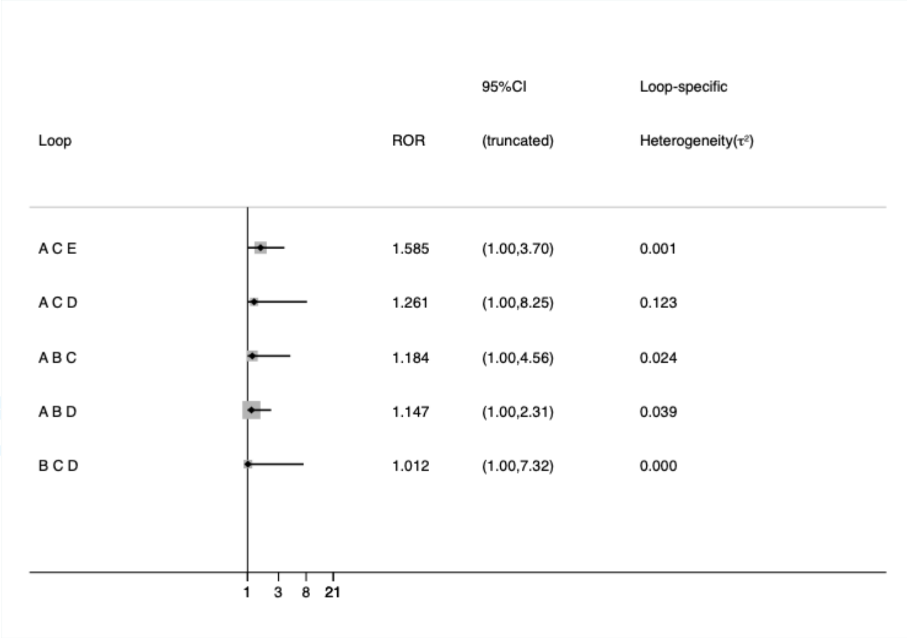

Design-by-treatment interaction model:

χ^2 statistic: 3.57, 6 degrees of freedom, P value: 0.735, between-study variance: 0.06. I^2 statistic=22%

For peer review only - http://bmjopen.bmj.com/site/about/guidelines.xhtml

Appendix 15: Network and standard meta-analysis results

Treatment Comparison	NMA estimate	95% CI	95% PI	P-score	MA estimate	95% CI	95% PI	#studies
				**Mini-Mental State Examination (MMSE)*†**				
Donepezil vs Placebo	1.41	0.51 to 2.32	-3.48 to 6.31	0.59	1.65	0.16 to 3.14	-6.02 to 9.32	24
Rivastigmine oral vs Placebo	0.69	-0.79 to 2.18	-4.35 to 5.74	0.36	0.60	-0.43 to 1.62	-3.07 to 4.26	6
Galantamine vs Placebo	0.41	-1.44 to 2.26	-4.76 to 5.58	0.28	0.04	-1.09 to 1.17	-12.39 to 12.47	3
Rivastigmine transdermal vs Placebo	2.11	-0.04 to 4.26	-3.18 to 7.40	0.72	0.56	-0.33 to 1.45	--	2
Memantine vs Placebo	0.67	-0.99 to 2.34	-4.43 to 5.78	0.35	0.52	0.03 to 1.01	-0.69 to 1.73	7
Donepezil + Memantine vs Placebo	2.57	0.07 to 5.07	-2.88 to 8.02	0.80	4.21	1.94 to 6.48	--	1
Galantamine + Memantine vs Placebo	2.24	-2.13 to 6.61	-4.33 to 8.81	0.66				
Rivastigmine transdermal + Memantine vs Placebo	1.79	-1.70 to 5.27	-4.20 to 7.78	0.60				
Placebo (reference)				0.14				
Rivastigmine transdermal vs Rivastigmine oral	1.41	-0.80 to 3.62	-3.90 to 6.73		2.26	-0.48 to 4.99	-30.56 to 35.07	3
Rivastigmine oral vs Donepezil	-0.72	-2.28 to 0.84	-5.79 to 4.35		0.16	-0.57 to 0.90	-1.45 to 1.77	4
Galantamine vs Rivastigmine oral	-0.29	-2.48 to 1.91	-5.60 to 5.02		0.06	-1.05 to 1.17		1
Rivastigmine transdermal vs Donepezil	0.69	-1.52 to 2.91	-4.62 to 6.01		-0.20	-2.78 to 2.38		1
Rivastigmine transdermal vs Galantamine	1.70	-0.93 to 4.33	-3.81 to 7.21		2.20	-0.19 to 4.59		1
Rivastigmine transdermal + Memantine vs Rivastigmine transdermal	-0.32	-3.82 to 3.18	-6.32 to 5.68		-0.40	-1.40 to 0.60		1
Memantine vs Donepezil	-0.74	-2.56 to 1.08	-5.90 to 4.42		0.20	0.88 to 1.28		1
Donepezil + Memantine vs Donepezil	1.15	-1.33 to 3.64	-4.29 to 6.59		0.88	0.64 to 1.11		2
Galantamine vs Donepezil	-1.01	-2.86 to 0.84	-6.18 to 4.16		-0.35	-1.52 to 0.83	-5.31 to 4.62	4
Donepezil + Memantine vs Memantine	1.89	-0.88 to 4.67	-3.69 to 7.48		0.37	-1.04 to 1.78		1
Galantamine + Memantine vs Memantine	1.57	-2.78 to 5.92	-4.98 to 8.12		0.82	-0.58 to 2.22		1
Rivastigmine transdermal + Memantine vs Memantine	1.12	-2.47 to 4.70	-4.93 to 7.16		0.41	-1.17 to 1.99		1

For peer review only

For peer review only - http://bmjopen.bmj.com/site/about/guidelines.xhtml

Galantamine + Memantine vs Donepezil + Memantine	-0.33	-4.72 to 4.06	-6.91 to 6.23		0.45	-0.85 to 1.75		1
Rivastigmine transdermal + Memantine vs Donepezil + Memantine	-0.78	-4.53 to 2.97	-6.93 to 5.38		0.04	-1.45 to 1.53		1
Rivastigmine transdermal + Memantine vs Galantamine + Memantine	-0.45	-5.05 to 4.14	-7.18 to 6.28		-0.41	-1.89 to 1.07		1
Common within-network between-study variance τ^2 = 5.75, I^2 = 96% (96%, 97%)								
Design-by-treatment interaction model for inconsistency χ^2 *(d.f., P-value,* τ^2*)*: 4.36 (13, 0.987, 7.35)								
**Serious Adverse Events (SAEs)***‡								
Donepezil vs Placebo	1.08	0.87 to 1.35	0.67 to 1.75	0.30	1.07	0.88 to 1.31	0.84 to 1.37	16
Rivastigmine oral vs Placebo	1.26	0.82 to 1.94	0.69 to 2.33	0.16	1.26	0.75 to 2.12	0.01 to 161.35	3
Galantamine vs Placebo	0.95	0.74 to 1.22	0.58 to 1.55	0.53	1.02	0.71 to 1.46	0.38 to 2.77	8
Rivastigmine transdermal vs Placebo	0.90	0.58 to 1.42	0.48 to 1.69	0.57	0.86	0.53 to 1.40		1
Memantine vs Placebo	0.88	0.64 to 1.20	0.52 to 1.49	0.63	0.87	0.63 to 1.20	0.38 to 1.99	8
Donepezil + Memantine vs Placebo	0.77	0.34 to 1.73	0.30 to 1.96	0.69				
Galantamine + Memantine vs Placebo	1.03	0.45 to 2.39	0.39 to 2.70	0.43				
Rivastigmine transdermal + Memantine vs Placebo	0.72	0.32 to 1.59	0.28 to 1.81	0.75				
Placebo (reference)				0.44				
Rivastigmine oral Donepezil vs	1.17	0.73 to 1.87	0.61 to 2.22		2.08	0.21 to 20.73		2
Galantamine vs Donepezil	0.88	0.64 to 1.19	0.52 to 1.49		0.79	0.46 to 1.39	0.32 to 1.96	5
Donepezil + Memantine vs Donepezil	0.71	0.33 to 1.55	0.29 to 1.76		0.71	0.37 to 1.38		1
Rivastigmine transdermal vs Rivastigmine oral	0.72	0.42 to 1.23	0.36 to 1.44		0.94	0.52 to 1.68		1
Rivastigmine transdermal + Memantine vs Rivastigmine transdermal	0.79	0.41 to 1.54	0.36 to 1.77		0.79	0.45 to 1.39		2
Galantamine vs Rivastigmine oral	0.75	0.46 to 1.22	0.39 to 1.45		0.63	0.15 to 2.64		1
Galantamine + Memantine vs Galantamine	1.09	0.49 to 2.42	0.43 to 2.75		1.09	0.55 to 2.17		1
Common within-network between-study variance τ2 = 0.04, I^2 = 22% (0%, 48%)								
Design-by-treatment interaction model for inconsistency χ^2 *(d.f., P-value,* τ^2*)*: 3.57 (6, 0.735, 0.06)								

For peer review only

For peer review only - http://bmjopen.bmj.com/site/about/guidelines.xhtml

* Aggregate data and fully adjusted results from studies with available individual patient data were used in both meta-analysis and NMA. The mean difference effect size is presented for MMSE and the odds ratio for SAE.
† MMSE: Studies with available IPD included only available participants –to assess the missing data impact on the second stage (IMDoM) a separate analysis was applied
‡ SAE: Studies with available IPD included all randomized participants

For peer review only

For peer review only - http://bmjopen.bmj.com/site/about/guidelines.xhtml

Appendix 16: Additional analyses using IPD and aggregate data

Cognition

Additional analyses using both IPD and aggregate data, were in agreement with the main analysis findings, overall (Appendix 17). Cognitive performance was better in patients with mild to moderate MMSE receiving donepezil (MD= 1·68 95% CI: 0·31 to 3·06, P-score= 69%) and most likely transdermal rivastigmine (MD= 2·74 95% CI: -0·68 to 6·16, P-score= 81%). In patients with moderate to severe MMSE the combination donepezil+memantine improved MMSE score significantly (MD= 2·49 95% CI: 1·55 to 3·44, P-score=100%), but oral rivastigmine deteriorated MMSE score significantly (MD= -1·00 95% CI: -1·87 to -0·12, P-score= 4%). Donepezil (MD= 1·31 95% CI: 0·66 to 1·96, P-score= 78%) and memantine (MD=0·69 95% CI: 0·07 to 1·31, P-score= 59%) also performed well for patients with moderate to severe cognitive impairment. However, PrIs are wide suggesting results are not conclusive.

Accounting for the impact of the outlier studies, galantamine+memantine was the second-best cognitive enhancer (MD= 1·87 95% CI: 0·08 to 3·66, P-score=82%) after donepezil+memantine (MD= 2·04 95% CI: 1·03 to 3·05, P-score= 92%). Using only IPD adjusted for comorbidities suggested that oral rivastigmine improves MMSE score, but results are inconclusive (MD= 0·88 95% CI: 0·31 to 1·45, 95% PI: -0·05 to 1·81, P-score= 75%). Similarly, using IPD adjusted for MMSE baseline suggested that oral rivastigmine (MD= 0·88 95% CI: 0·31 to 1·45, P-score= 69%) and galantamine (MD= 0·76 95% CI: 0·34 to 1·18, P-score= 62%) improve MMSE score, but in a future study, results are only stable for galantamine.

Heterogeneity in NMA was high (between-study variance = 5·75, I2= 96%) compared also to the Rhodes et al21 empirical distribution (median 0.05, 95% range: 0·00 to 7·56). However, heterogeneity decreased importantly when excluding outliers (between-study variance = 0·59, I2= 73%), when including only patients with moderate to severe AD (between-study variance = 0·18, I2= 44%), restricting to industry-sponsored trials (between-study variance = 0·16, I2= 43%), and when using IPD only (between-study variance = 0·12, I2= 29%).

Serious adverse events

Additional analyses using both IPD and aggregate data, showed that memantine was statistically significantly associated with lower odds of a SAE than placebo when using study duration as a covariate (OR= 0·61, 95% CI: 0·37 to 0·93, P-score= 88%). Restricting to low risk of bias for incomplete outcome data, galantamine was associated with significantly lower odds of a SAE (OR= 0·69, 95% CI: 0·50 to 0·97, P-score= 80%). The available case analysis for studies with available IPD showed that donepezil (OR= 1·63, 95% CI: 0·49 to 5·41) and oral rivastigmine (OR= 1·28, 95% CI: 0·08 to 19·94) were associated with higher odds of a SAE, yet these were non-statistically significant, and overall there were no major differences with the intention to treat IPD NMA results.

Heterogeneity in NMA was low (between-study variance = 0·04, I2= 22%) compared to the Turner et al20 empirical distribution (median 0·12, 95% range: 0·01 to 2·63). Heterogeneity decreased importantly when restricting to aggregate data (between-study variance = 0·00, I2= 0%), low risk of bias for incomplete outcome data (between-study variance = 0·02, I2= 10%), patients with moderate to severe cognitive impairment (between-study variance = 0·00, I2= 0%), and when adjusting for study duration (between-study variance = 0·03), year of publication (between-study variance = 0·02), mean age (between-study variance = 0.02) or sex (between-study variance = 0·03).

For peer review only - http://bmjopen.bmj.com/site/about/guidelines.xhtml

Appendix 17: Network subgroup and meta-regression analysis results

Treatment Comparison	NMA estimate	95% CI	95%PI	P-score
**Mini-Mental State Examination (MMSE)†**				
**Mean Difference: Aggregate data and crude results from studies with available individual patient data**				
Donepezil vs Placebo	1.41	0.50 to 2.33	-3.51 to 6.34	0.59
Rivastigmine oral vs Placebo	0.69	-0.80 to 2.19	-4.38 to 5.76	0.36
Galantamine vs Placebo	0.37	-1.49 to 2.23	-4.82 to 5.57	0.28
Rivastigmine transdermal vs Placebo	2.10	-0.06 to 4.26	-3.22 to 7.42	0.72
Memantine vs Placebo	0.63	-1.05 to 2.30	-4.51 to 5.76	0.34
Donepezil + Memantine vs Placebo	2.56	0.04 to 5.07	-2.92 to 8.04	0.79
Galantamine + Memantine vs Placebo	2.22	-2.18 to 6.61	-4.39 to 8.82	0.66
Rivastigmine transdermal + Memantine vs Placebo	1.77	-1.73 to 5.27	-4.25 to 7.79	0.60
Placebo (reference)				0.14
Common within-network between-study variance τ^2 = 5.81, I^2 = 96% (96%, 97%)				
Design-by-treatment interaction model for inconsistency χ^2 *(d.f., P-value,* τ^2*)*: 4.42 (13, 0.986, 7.44)				
**Mean Difference: Aggregate data results****				
Donepezil vs Placebo	1.55	0.41 to 2.68	-4.16 to 7.25	0.57
Rivastigmine oral vs Placebo	0.71	-1.10 to 2.52	-5.18 to 6.60	0.34
Galantamine vs Placebo	0.57	-1.98 to 3.12	-5.61 to 6.74	0.32
Rivastigmine transdermal vs Placebo	2.60	-0.20 to 5.40	-3.69 to 8.89	0.75
Memantine vs Placebo	0.82	-1.37 to 3.01	-5.21 to 6.84	0.37
Donepezil + Memantine vs Placebo	2.71	-0.17 to 5.60	-3.62 to 9.04	0.76
Galantamine + Memantine vs Placebo	2.44	-2.61 to 7.48	-5.19 to 10.07	0.65
Rivastigmine transdermal + Memantine vs Placebo	2.09	-1.98 to 6.15	-4.89 to 9.07	0.61
Placebo (reference)				0.15
Common within-network between-study variance τ^2 = 7.66, I2 = 97% (96%, 97%)				
Design-by-treatment interaction model for inconsistency χ^2 *(d.f., P-value,* τ^2*)*: 3.92 (11, 0.972, 8.76)				
**Mean Difference: Crude results from studies with available individual patient data**				
Donepezil vs Placebo	0.70	0.01 to 1.40	-0.67 to 2.07	0.65
Rivastigmine oral vs Placebo	0.87	-0.01 to 1.75	-0.70 to 2.44	0.73
Galantamine vs Placebo	0.45	-0.24 to 1.14	-0.91 to 1.82	0.48
Rivastigmine transdermal vs Placebo	1.06	0.04 to 2.08	-0.67 to 2.79	0.82
Memantine vs Placebo	0.05	-0.74 to 0.83	-1.42 to 1.51	0.20
Placebo (reference)				0.13
Common within-network between-study variance τ^2 = 0.12, I^2 = 29% (0%, 71%)				
Design-by-treatment interaction model for inconsistency χ^2 *(d.f., P-value,* τ^2*)*: N/A (no closed loops)				
**Mean Difference: Low Risk of Bias for Allocation Concealment***				
Donepezil vs Placebo	2.02	-0.24 to 4.28	-6.19 to 10.23	0.70
Rivastigmine oral vs Placebo	1.38	-2.27 to 5.02	-7.39 to 10.14	0.57
Galantamine vs Placebo	-0.31	-4.61 to 3.98	-9.42 to 8.79	0.31
Rivastigmine transdermal vs Placebo	0.82	-4.08 to 5.72	-8.63 to 10.27	0.48
Memantine vs Placebo	0.69	-3.01 to 4.39	-8.10 to 9.49	0.46
Donepezil + Memantine vs Placebo	2.88	-4.75 to 10.51	-8.48 to 14.23	0.69
Placebo (reference)				0.30
Common within-network between-study variance: τ^2 = 13.82, I^2 = 98% (98%, 99%)				
Design-by-treatment interaction model for inconsistency χ^2 *(d.f., P-value,* τ^2*)*: 0.13 (3, 0.99, 19.10)				
**Mean Difference: Low risk of bias for Incomplete Data***				
Donepezil vs Placebo	0.87	0.07 to 1.66	-1.67 to 3.40	0.61
Rivastigmine oral vs Placebo	-1.52	-4.41 to 1.37	-5.54 to 2.50	0.10
Galantamine vs Placebo	0.52	-0.94 to 1.99	-2.36 to 3.41	0.48
Rivastigmine transdermal vs Placebo	1.37	-0.64 to 3.38	-1.91 to 4.65	0.71
Memantine vs Placebo	0.57	-1.12 to 2.27	-2.47 to 3.62	0.48
Donepezil + Memantine vs Placebo	0.94	-2.11 to 4.00	-3.23 to 5.11	0.57
Galantamine + Memantine vs Placebo	1.39	-1.66 to 4.44	-2.77 to 5.56	0.70
Rivastigmine transdermal + Memantine vs Placebo	0.98	-2.15 to 4.12	-3.26 to 5.23	0.58
Placebo (reference)				0.27
Common within-network between-study variance: τ^2 = 1.16, I^2 = 79% (65%, 88%)				
Design-by-treatment interaction model for inconsistency χ^2 *(d.f., P-value,* τ^2*)*: 12.15 (3, 0.007, 0.863)				
**Mean Difference: Publicly-Sponsored Studies***				
Donepezil vs Placebo	6.57	-4.68 to 17.81	-129.61 to 142.74	0.71
Rivastigmine oral vs Placebo	1.40	-16.41 to 19.21	-161.58 to 164.38	0.44
Memantine vs Placebo	0.11	-17.65 to 17.87	-162.64 to 162.86	0.39
Rivastigmine transdermal + Memantine vs Placebo	5.83	-7.98 to 19.64	-139.93 to 151.59	0.65
Placebo (reference)				0.32

For peer review only - http://bmjopen.bmj.com/site/about/guidelines.xhtml

Common within-network between-study variance: τ^2 = 81.93, I^2 = 99% (99%, 100%)				
Design-by-treatment interaction model for inconsistency χ^2 (d.f., P-value, τ^2): 0.05 (1, 0.815, 116.71)				
**Mean Difference: Industry-Sponsored Studies***				
Donepezil vs Placebo	0.98	0.69 to 1.27	0.10 to 1.86	0.85
Rivastigmine oral vs Placebo	0.82	0.35 to 1.29	-0.14 to 1.78	0.69
Galantamine vs Placebo	0.41	-0.15 to 0.96	-0.60 to 1.41	0.34
Rivastigmine transdermal vs Placebo	0.80	0.18 to 1.41	-0.25 to 1.84	0.67
Memantine vs Placebo	0.60	0.06 to 1.15	-0.39 to 1.60	0.50
Rivastigmine transdermal + Memantine vs Placebo	0.40	-1.02 to 1.81	-1.29 to 2.08	0.39
Placebo (reference)				0.06
Common within-network between-study variance: τ^2 = 0.16, I^2 = 43% (15%, 62%)				
Design-by-treatment interaction model for inconsistency χ^2 (d.f., P-value, τ^2): 8.06 (7, 0.327, 0.16)				
**Mean Difference: Studies with Mild to Moderate baseline MMSE***				
Donepezil vs Placebo	1.68	0.31 to 3.06	-4.81 to 8.18	0.69
Rivastigmine oral vs Placebo	0.88	-1.29 to 3.05	-5.85 to 7.61	0.51
Galantamine vs Placebo	0.31	-2.47 to 3.09	-6.66 to 7.28	0.40
Rivastigmine transdermal vs Placebo	2.74	-0.68 to 6.16	-4.53 to 10.01	0.81
Memantine vs Placebo	-0.58	-4.84 to 3.69	-8.31 to 7.16	0.28
Donepezil + Memantine vs Placebo	0.43	-6.36 to 7.21	-9.06 to 9.91	0.45
Galantamine + Memantine vs Placebo	0.88	-5.90 to 7.66	-8.61 to 10.37	0.51
Rivastigmine transdermal + Memantine vs Placebo	1.11	-4.20 to 6.42	-7.30 to 9.52	0.55
Placebo (reference)				0.31
Common within-network between-study variance: τ^2 = 9.67, I^2 = 97% (97%, 98%)				
Design-by-treatment interaction model for inconsistency χ^2 (d.f., P-value, τ^2): 3.22 (9, 0.96, 13.28)				
**Mean Difference: Studies with Moderate to Severe baseline MMSE***				
Donepezil vs Placebo	1.31	0.66 to 1.96	-0.01 to 2.63	0.78
Rivastigmine oral vs Placebo	-1.00	-1.87 to -0.12	-2.51 to 0.51	0.04
Galantamine vs Placebo	-0.21	-1.64 to 1.21	-2.28 to 1.86	0.28
Memantine vs Placebo	0.69	0.07 to 1.31	-0.61 to 2.00	0.59
Donepezil + Memantine vs Placebo	2.49	1.55 to 3.44	0.92 to 4.07	1.00
Placebo (reference)				0.32
Common within-network between-study variance: τ^2 = 0.18, I^2 = 44% (0%, 75%)				
Design-by-treatment interaction model for inconsistency χ^2 (d.f., P-value, τ^2): 2.60 (1, 0.11, 0.11)				
**Mean Difference: Excluding outlier studies*[§]**				
Donepezil vs Placebo	0.95	0.59 to 1.32	-0.64 to 2.54	0.57
Rivastigmine oral vs Placebo	0.65	0.09 to 1.22	-1.00 to 2.30	0.37
Galantamine vs Placebo	0.36	-0.38 to 1.09	-1.36 to 2.07	0.22
Rivastigmine transdermal vs Placebo	1.03	0.15 to 1.91	-0.76 to 2.82	0.59
Memantine vs Placebo	0.67	0.02 to 1.32	-1.01 to 2.35	0.39
Donepezil + Memantine vs Placebo	2.04	1.03 to 3.05	0.18 to 3.90	0.92
Galantamine + Memantine vs Placebo	1.87	0.08 to 3.66	-0.53 to 4.26	0.82
Rivastigmine transdermal + Memantine vs Placebo	1.10	-0.33 to 2.53	-1.03 to 3.23	0.58
Placebo (reference)				0.04
Common within-network between-study variance: τ^2 = 0.59, I^2 = 73% (64%, 79%)				
Design-by-treatment interaction model for inconsistency χ^2 (d.f., P-value, τ^2): 10.60 (13, 0.64, 0.61)				
**Accounting for missing outcome data - Informative Missingness Difference of Means[¶]**				
Donepezil vs Placebo	1.42	0.51 to 2.33	0.51 to 2.33	0.59[‖]
Rivastigmine oral vs Placebo	0.45	-1.09 to 1.99	-1.09 to 1.99	0.30[‖]
Galantamine vs Placebo	0.19	-1.78 to 2.17	-1.78 to 2.17	0.25[‖]
Rivastigmine transdermal vs Placebo	2.37	-0.03 to 4.79	-0.03 to 4.79	0.76[‖]
Memantine vs Placebo	0.60	-1.09 to 2.42	-1.09 to 2.42	0.36[‖]
Donepezil + Memantine vs Placebo	2.55	0.09 to 5.01	0.09 to 5.01	0.80[‖]
Galantamine + Memantine vs Placebo	2.26	-2.03 to 6.56	-2.03 to 6.56	0.68[‖]
Rivastigmine transdermal + Memantine vs Placebo	1.81	-1.66 to 5.28	-1.66 to 5.28	0.61[‖]
Placebo (reference)				0.16[‖]
Common within-network between-study variance: τ^2 = 5.47[‖]				
Design-by-treatment interaction model for inconsistency χ^2 (d.f., P-value, τ^2): 4.45 (11, 0.955, 6.45)				
**Mean Difference: Meta-regression, Trial Mean Age[**]**				
Donepezil vs Placebo	1.53	0.52 to 2.53	-3.17 to 6.27	0.50[††]
Rivastigmine oral vs Placebo	0.80	-0.84 to 2.44	-4.15 to 5.79	0.37[††]
Galantamine vs Placebo	0.60	-1.63 to 2.83	-4.57 to 5.72	0.25[††]
Rivastigmine transdermal vs Placebo	2.53	0.06 to 4.98	-2.72 to 7.80	0.75[††]
Memantine vs Placebo	0.79	-1.18 to 2.74	-4.33 to 5.85	0.37[††]
Donepezil + Memantine vs Placebo	2.66	0.09 to 5.19	-2.70 to 7.97	0.87[††]
Galantamine + Memantine vs Placebo	2.39	-2.02 to 6.84	-4.14 to 8.83	0.75[††]
Rivastigmine transdermal + Memantine vs Placebo	2.05	-1.53 to 5.59	-3.83 to 7.94	0.75[††]
Placebo (reference)				0.12[††]
Regression coefficient	0.03	-0.14 to 0.20		
Common within-network between-study variance: τ^2 = 5.50	3.72 to 8.51			

Design-by-treatment interaction model for inconsistency χ^2 (d.f., P-value, τ^2): 3.92 (11, 0.972, 8.76)				
**Mean Difference: NMA of studies with IPD adjusted for Age**				
Donepezil vs Placebo	0.72	0.03 to 1.42	-0.66 to 2.10	0.66
Rivastigmine oral vs Placebo	0.84	-0.05 to 1.73	-0.75 to 2.43	0.70
Galantamine vs Placebo	0.46	-0.24 to 1.15	-0.92 to 1.83	0.48
Rivastigmine transdermal vs Placebo	1.05	0.04 to 2.06	-0.68 to 2.78	0.83
Memantine vs Placebo	0.06	-0.72 to 0.84	-1.40 to 1.53	0.21
Placebo (reference)				0.12
Common within-network between-study variance: τ^2 = 0.12, I^2 = 29% (0%, 71%)				
Design-by-treatment interaction model for inconsistency χ^2 (d.f., P-value, τ^2): N/A (one closed loop with a single multi-arm trial)				
**Mean Difference: Meta-regression, Percent of Male Participants**[**]				
Donepezil vs Placebo	1.62	0.58 to 2.65	-3.40 to 6.61	0.62 [††]
Rivastigmine oral vs Placebo	0.73	-0.90 to 2.35	-4.30 to 5.81	0.37 [††]
Galantamine vs Placebo	0.62	-1.65 to 2.89	-4.75 to 5.93	0.25 [††]
Rivastigmine Transdermal vs Placebo	2.51	0.01 to 5.04	-2.78 to 7.94	0.75 [††]
Memantine vs Placebo	0.66	-1.47 to 2.77	-4.54 to 5.88	0.25 [††]
Donepezil + Memantine vs Placebo	2.52	-0.40 to 5.45	-3.09 to 8.17	0.75 [††]
Galantamine + Memantine vs Placebo	2.27	-2.28 to 6.83	-4.37 to 8.90	0.75 [††]
Rivastigmine transdermal + Memantine vs Placebo	1.98	-1.67 to 5.65	-4.02 to 7.99	0.75 [††]
Placebo (reference)				0.12 [††]
Regression coefficient	0.01	-0.05 to 0.06		
Common within-network between-study variance: τ^2 = 5.73	3.83 to 8.84			
Design-by-treatment interaction model for inconsistency χ^2 (d.f., P-value, τ^2): 3.72 (10, 0.959, 8.97)				
**Mean difference: NMA of studies with IPD adjusted for Percent of Male Participants**				
Donepezil vs Placebo	0.76	0.05 to 1.47	-0.67 to 2.19	0.67
Rivastigmine oral vs Placebo	0.85	-0.07 to 1.77	-0.80 to 2.50	0.69
Galantamine vs Placebo	0.45	-0.27 to 1.16	-0.99 to 1.88	0.46
Rivastigmine transdermal vs Placebo	1.05	0.01 to 2.09	-0.74 to 2.84	0.81
Memantine vs Placebo	0.10	-0.68 to 0.89	-1.40 to 1.61	0.23
Placebo (reference)				0.11
Common within-network between-study variance: τ^2 = 0.13, I^2 = 32% (0%, 72%)				
Design-by-treatment interaction model for inconsistency χ^2 (d.f., P-value, τ^2): N/A (one closed loop with a single multi-arm trial)				
**Mean Difference: NMA of studies with IPD adjusted for MMSE baseline**				
Donepezil vs Placebo	0.79	0.26 to 1.32	-0.06 to 1.64	0.64
Rivastigmine oral vs Placebo	0.88	0.31 to 1.45	-0.05 to 1.81	0.69
Galantamine vs Placebo	0.76	0.34 to 1.18	0.08 to 1.44	0.62
Rivastigmine transdermal vs Placebo	1.02	0.27 to 1.77	-0.20 to 2.24	0.82
Memantine vs Placebo	0.07	-0.52 to 0.66	-0.89 to 1.03	0.14
Placebo (reference)				0.08
Common within-network between-study variance: τ^2 = 0.00, I^2 = 0% (0%, 79%)				
Design-by-treatment interaction model for inconsistency χ^2 (d.f., P-value, τ^2): N/A (one closed loop with a single multi-arm trial)				
**Mean Difference: NMA of studies with IPD adjusted for comorbidities**				
Donepezil vs Placebo	0.77	0.21 to 1.33	-0.15 to 1.68	0.71
Rivastigmine oral vs Placebo	0.88	0.31 to 1.45	-0.05 to 1.81	0.75
Galantamine vs Placebo	-0.29	-1.46 to 0.88	-2.19 to 1.61	0.15
Rivastigmine transdermal vs Placebo	1.05	0.30 to 1.80	-0.17 to 2.27	0.88
Memantine vs Placebo	0.05	-0.55 to 0.64	-0.92 to 1.01	0.27
Placebo (reference)				0.15
Common within-network between-study variance: τ^2 = 0.00, I^2 = 0% (0%, 67%)				
Design-by-treatment interaction model for inconsistency χ^2 (d.f., P-value, τ^2): N/A (one closed loop with a single multi-arm trial)				
**Mean Difference: NMA of studies with IPD adjusted for other medications**				
Donepezil vs Placebo	0.67	-0.34 to 1.69	-1.44 to 2.79	0.61
Rivastigmine oral vs Placebo	0.87	-0.12 to 1.86	-1.21 to 2.95	0.71
Galantamine vs Placebo	0.42	-0.35 to 1.19	-1.40 to 2.25	0.47
Rivastigmine transdermal vs Placebo	1.07	-0.04 to 2.18	-1.16 to 3.30	0.81
Memantine vs Placebo	0.11	-0.74 to 0.96	-1.80 to 2.02	0.26
Placebo (reference)				0.14
Common within-network between-study variance: τ^2 = 0.17, I^2 = 35% (0%, 76%)				
Design-by-treatment interaction model for inconsistency χ^2 (d.f., P-value, τ^2): N/A (one closed loop with a single multi-arm trial)				
**Mean Difference: Meta-regression, Study Duration**[**]				
Donepezil vs Placebo	1.66	0.67 to 2.66	-3.12 to 6.32	0.62 [††]
Rivastigmine oral vs Placebo	0.80	-0.77 to 2.37	-4.14 to 5.69	0.37 [††]
Galantamine vs Placebo	0.47	-1.75 to 2.68	-4.64 to 5.66	0.25 [††]
Rivastigmine transdermal vs Placebo	2.38	-0.04 to 4.83	-2.87 to 7.56	0.75 [††]
Memantine vs Placebo	0.67	-1.27 to 2.58	-4.35 to 5.79	0.25 [††]
Donepezil + Memantine vs Placebo	2.67	0.18 to 5.16	-2.60 to 7.97	0.88 [††]
Galantamine + Memantine vs Placebo	2.43	-1.94 to 6.79	-3.94 to 8.81	0.75 [††]
Rivastigmine transdermal + Memantine vs Placebo	2.13	-1.40 to 5.63	-3.62 to 7.87	0.75 [††]
Placebo (reference)				0.12 [††]

For peer review only - http://bmjopen.bmj.com/site/about/guidelines.xhtml

Regression coefficient	0.02	-0.01 to 0.06		
Common within-network between-study variance: τ^2 = 5.40	3.63 to 8.29			
Design-by-treatment interaction model for inconsistency χ^2 (d.f., P-value, τ^2): 4.36 (13, 0.987, 7.35)				
**Mean Difference: Meta-regression, Year of Publication**[**]				
Donepezil vs Placebo	1.53	0.51 to 2.54	-3.27 to 6.31	0.50 [††]
Rivastigmine oral vs Placebo	0.66	-1.01 to 2.32	-4.31 to 5.65	0.25 [††]
Galantamine vs Placebo	0.60	-1.65 to 2.85	-4.65 to 5.83	0.25 [††]
Rivastigmine transdermal vs Placebo	2.59	0.09 to 5.12	-2.73 to 7.95	0.75 [††]
Memantine vs Placebo	0.89	-1.05 to 2.80	-4.17 to 5.90	0.38 [††]
Donepezil + Memantine vs Placebo	2.82	0.19 to 5.44	-2.57 to 8.21	0.88 [††]
Galantamine + Memantine vs Placebo	2.59	-1.93 to 7.16	-3.98 to 9.12	0.75 [††]
Rivastigmine transdermal + Memantine vs Placebo	2.21	-1.49 to 5.95	-3.81 to 8.24	0.75 [††]
Placebo (reference)				0.12 [††]
Regression coefficient	-0.02	-0.17 to 0.14		
Common within-network between-study variance: τ^2 = 5.53	3.71 to 8.48			
Design-by-treatment interaction model for inconsistency χ^2 (d.f., P-value, τ^2): 4.36 (13, 0.987, 7.35)				
**Serious Adverse Events (SAEs)‡**				
**Odds Ratio: Aggregate data and crude results from studies with available individual patient data**				
Donepezil vs Placebo	1.07	0.86 to 1.32	0.68 to 1.67	0.31
Rivastigmine oral vs Placebo	1.26	0.83 to 1.90	0.70 to 2.24	0.16
Galantamine vs Placebo	0.95	0.75 to 1.21	0.60 to 1.51	0.52
Rivastigmine transdermal vs Placebo	0.87	0.57 to 1.35	0.48 to 1.58	0.61
Memantine vs Placebo	0.91	0.67 to 1.22	0.55 to 1.49	0.59
Donepezil + Memantine vs Placebo	0.76	0.34 to 1.68	0.31 to 1.88	0.69
Galantamine + Memantine vs Placebo	1.03	0.45 to 2.36	0.41 to 2.64	0.42
Rivastigmine transdermal + Memantine vs Placebo	0.69	0.32 to 1.51	0.28 to 1.70	0.77
Placebo (reference)				0.43
Common within-network between-study variance τ^2 = 0.04, I^2 = 20% (0%, 47%)				
Design-by-treatment interaction model for inconsistency χ^2 (d.f., P-value, τ^2): 3.58 (6, 0.733, 0.05)				
**Odds Ratio: Aggregate data results**[**]				
Donepezil vs Placebo	1.09	0.89 to 1.33	0.88 to 1.35	0.25
Rivastigmine oral vs Placebo	1.43	0.92 to 2.21	0.90 to 2.26	0.07
Galantamine vs Placebo	0.88	0.63 to 1.25	0.62 to 1.27	0.54
Rivastigmine transdermal vs Placebo	0.79	0.44 to 1.41	0.43 to 1.45	0.61
Memantine vs Placebo	0.70	0.51 to 0.97	0.50 to 0.98	0.77
Donepezil + Memantine vs Placebo	0.77	0.39 to 1.54	0.37 to 1.60	0.64
Galantamine + Memantine vs Placebo	0.96	0.45 to 2.08	0.43 to 2.16	0.44
Rivastigmine transdermal + Memantine vs Placebo	0.62	0.28 to 1.40	0.27 to 1.46	0.80
Placebo (reference)				0.38
Common within-network between-study variance τ^2 = 0.00, I^2 = 0% (0%, 42%)				
Design-by-treatment interaction model for inconsistency χ^2 (d.f., P-value, τ^2): 2.29 (4, 0.682, 0.01)				
**Odds Ratio: Crude results from studies with available individual patient data**				
Donepezil vs Placebo	0.95	0.50 to 1.78	0.33 to 2.70	0.57
Rivastigmine oral vs Placebo	0.81	0.37 to 1.75	0.25 to 2.61	0.71
Galantamine vs Placebo	1.05	0.71 to 1.56	0.44 to 2.50	0.46
Rivastigmine transdermal vs Placebo	0.92	0.38 to 2.20	0.26 to 3.31	0.57
Memantine vs Placebo	1.41	0.81 to 2.45	0.53 to 3.79	0.16
				0.53
Common within-network between-study variance τ^2 = 0.10, I^2 = 48% (0%, 76%)				
Design-by-treatment interaction model for inconsistency χ^2 (d.f., P-value, τ^2): N/A (no closed loops)				
**Odds Ratio: Low Risk of Bias for Allocation Concealment***				
Donepezil vs Placebo	0.88	0.60 to 1.29	0.42 to 1.83	0.52
Rivastigmine oral vs Placebo	1.15	0.67 to 1.98	0.50 to 2.68	0.21
Galantamine vs Placebo	0.94	0.64 to 1.38	0.45 to 1.95	0.44
Rivastigmine transdermal vs Placebo	0.88	0.52 to 1.49	0.39 to 2.02	0.51
Memantine vs Placebo	0.86	0.55 to 1.36	0.40 to 1.88	0.54
Donepezil + Memantine vs Placebo	0.63	0.24 to 1.62	0.19 to 2.05	0.75
Rivastigmine transdermal + Memantine vs Placebo	0.67	0.25 to 1.80	0.20 to 2.28	0.71
Placebo (reference)				0.33
Common within-network between-study variance: τ^2 = 0.08, I^2 = 37% (0%, 64%)				
Design-by-treatment interaction model for inconsistency χ^2 (d.f., P-value, τ^2): 2.19 (3, 0.53, 0.1)				
**Odds Ratio: Low Risk of Bias for Incomplete Data***				
Donepezil vs Placebo	0.83	0.53 to 1.29	0.45 to 1.51	0.51
Galantamine vs Placebo	0.69	0.50 to 0.97	0.42 to 1.13	0.80
Rivastigmine transdermal vs Placebo	0.79	0.42 to 1.49	0.36 to 1.76	0.56
Memantine vs Placebo	0.86	0.60 to 1.22	0.51 to 1.43	0.47
Placebo (reference)				0.16

For peer review only - http://bmjopen.bmj.com/site/about/guidelines.xhtml

Common within-network between-study variance: $\tau^2 = 0.02$, $I^2 = 10\%$ (0%, 50%)				
Design-by-treatment interaction model for inconsistency χ^2 (d.f., P-value, τ^2): 0.00 (1, 0.95, 0.04)				
**Odds Ratio: Publicly-Sponsored Studies***				
Donepezil vs Placebo	2.15	0.36 to 12.69	--	0.16
Memantine vs Placebo	0.71	0.45 to 1.12	--	0.86
Donepezil + Memantine vs Placebo	1.53	0.23 to 10.18	--	0.46
Placebo (reference)				0.51
Common within-network between-study variance: τ^2 = N/A (each comparison includes a single study)				
Design-by-treatment interaction model for inconsistency χ^2 (d.f., P-value, τ^2): N/A (no closed loops)				
**Odds Ratio: Industry-Sponsored Studies***				
Donepezil vs Placebo	1.08	0.86 to 1.35	0.64 to 1.82	0.34
Rivastigmine oral vs Placebo	1.27	0.82 to 1.98	0.66 to 2.44	0.16
Galantamine vs Placebo	0.99	0.75 to 1.31	0.57 to 1.71	0.52
Rivastigmine transdermal vs Placebo	0.91	0.57 to 1.44	0.46 to 1.77	0.62
Memantine vs Placebo	0.95	0.65 to 1.37	0.52 to 1.73	0.58
Rivastigmine transdermal + Memantine vs Placebo	0.72	0.31 to 1.64	0.27 to 1.90	0.79
Placebo (reference)				0.50
Common within-network between-study variance: $\tau^2 = 0.05$, $I^2 = 25\%$ (0%, 50%)				
Design-by-treatment interaction model for inconsistency χ^2 (d.f., P-value, τ^2): 3.68 (6, 0.72, 0.07)				
**Odds Ratio: Studies with Mild to Moderate baseline MMSE***				
Donepezil vs Placebo	1.27	0.88 to 1.83	0.61 to 2.65	0.29
Rivastigmine oral vs Placebo	1.36	0.83 to 2.24	0.60 to 3.09	0.25
Galantamine vs Placebo	1.01	0.67 to 1.55	0.47 to 2.19	0.56
Rivastigmine transdermal vs Placebo	1.02	0.50 to 2.05	0.39 to 2.69	0.55
Memantine vs Placebo	0.86	0.54 to 1.37	0.39 to 1.91	0.73
Galantamine + Memantine vs Placebo	1.10	0.40 to 3.00	0.32 to 3.78	0.48
Rivastigmine transdermal + Memantine vs Placebo	0.96	0.18 to 5.19	0.14 to 6.37	0.55
Placebo (reference)				0.59
Common within-network between-study variance: $\tau^2 = 0.09$, $I^2 = 29\%$ (0%, 57%)				
Design-by-treatment interaction model for inconsistency χ^2 (d.f., P-value, τ^2): 3.29 (5, 0.66, 0.13)				
**Odds Ratio: Studies with Moderate to Severe baseline MMSE***				
Donepezil vs Placebo	0.92	0.67 to 1.27	0.59 to 1.45	0.38
Galantamine vs Placebo	0.70	0.46 to 1.07	0.38 to 1.28	0.76
Memantine vs Placebo	0.95	0.55 to 1.62	0.44 to 2.02	0.36
Donepezil + Memantine vs Placebo	0.66	0.32 to 1.37	0.23 to 1.86	0.76
Placebo (reference)				0.23
Common within-network between-study variance: $\tau^2 = 0.00$, $I^2 = 0\%$ (0%, 72%)				
Design-by-treatment interaction model for inconsistency χ^2 (d.f., P-value, τ^2): 2.90 (1, 0.09, 0.00)				
**Odds Ratio: NMA of studies with IPD – available case analysis**				
Donepezil vs Placebo	1.63	0.49 to 5.41	0.30 to 8.73	0.33
Rivastigmine oral vs Placebo	1.28	0.08 to 19.94	0.04 to 39.11	0.46
Galantamine vs Placebo	1.05	0.67 to 1.63	0.38 to 2.85	0.58
Rivastigmine transdermal vs Placebo	0.81	0.02 to 35.04	0.01 to 82.49	0.59
Memantine vs Placebo	1.35	0.72 to 2.55	0.43 to 4.24	0.38
Placebo (reference)				0.64
Common within-network between-study variance: $\tau^2 = 0.13$, $I^2 = 50\%$ (0%, 77%)				
Design-by-treatment interaction model for inconsistency χ^2 (d.f., P-value, heterogeneity): N/A (no closed loops)				
**Odds Ratio: Meta-regression, Trial Mean Age**[**]				
Donepezil vs Placebo	1.13	0.88 to 1.43	0.68 to 1.86	0.25 [††]
Rivastigmine oral vs Placebo	1.52	0.89 to 2.53	0.77 to 3.04	0.00 [††]
Galantamine vs Placebo	0.91	0.60 to 1.30	0.52 to 1.59	0.50 [††]
Rivastigmine transdermal vs Placebo	0.84	0.39 to 1.58	0.34 to 1.80	0.75 [††]
Memantine vs Placebo	0.74	0.48 to 1.07	0.39 to 1.26	0.75 [††]
Donepezil + Memantine vs Placebo	0.92	0.38 to 1.89	0.33 to 2.15	0.62 [††]
Galantamine + Memantine vs Placebo	0.99	0.37 to 2.27	0.33 to 2.55	0.50 [††]
Rivastigmine transdermal + Memantine vs Placebo	0.73	0.24 to 1.70	0.22 to 1.87	0.87 [††]
Placebo (reference)				0.37 [††]
Regression coefficient (log-scale)	-0.03	-0.08 to 0.02		
Common within-network between-study variance: $\tau^2 = 0.02$	0.00 to 0.19			
Design-by-treatment interaction model for inconsistency χ^2 (d.f., P-value, τ^2): 3.57 (6, 0.735, 0.06)				
**Odds Ratio: NMA of studies with IPD adjusted for Age**				
Donepezil vs Placebo	0.95	0.50 to 1.78	0.33 to 2.73	0.57
Rivastigmine oral vs Placebo	0.84	0.39 to 1.81	0.26 to 2.74	0.68
Galantamine vs Placebo	1.04	0.70 to 1.55	0.43 to 2.52	0.46
Rivastigmine transdermal vs Placebo	0.91	0.38 to 2.17	0.25 to 3.28	0.58
Memantine vs Placebo	1.39	0.80 to 2.44	0.52 to 3.79	0.17
Placebo (reference)				0.53
Common within-network between-study variance: $\tau^2 = 0.10$, $I^2 = 48\%$ (0%, 76%)				
Design-by-treatment interaction model for inconsistency χ^2 (d.f., P-value, τ^2): N/A (no closed loops)				

For peer review only

**Odds Ratio: Meta-regression, Percent of Male Participants[**]**				
Donepezil vs Placebo	1.12	0.87 to 1.44	0.64 to 2.01	0.25 [††]
Rivastigmine oral vs Placebo	1.71	0.97 to 2.92	0.83 to 3.67	0.00 [††]
Galantamine vs Placebo	0.93	0.62 to 1.36	0.49 to 1.77	0.50 [††]
Rivastigmine transdermal vs Placebo	0.89	0.39 to 1.79	0.34 to 2.05	0.63 [††]
Memantine vs Placebo	0.64	0.37 to 1.00	0.29 to 1.21	0.88 [††]
Donepezil + Memantine vs Placebo	0.88	0.35 to 1.88	0.30 to 2.13	0.63 [††]
Galantamine + Memantine vs Placebo	1.13	0.39 to 2.58	0.36 to 2.95	0.38 [††]
Rivastigmine transdermal + Memantine vs Placebo	0.77	0.24 to 1.93	0.21 to 2.13	0.88 [††]
Placebo (reference)				0.38 [††]
Regression coefficient (log-scale)	0.00	0.00 to 0.02		
Common within-network between-study variance: τ^2 = 0.03	0.00 to 0.23			
Design-by-treatment interaction model for inconsistency χ^2 (d.f., P-value, τ^2): 3.57 (6, 0.735, 0.06)				
**Odds Ratio: NMA of studies with IPD adjusted for Percent of Male Participants**				
Donepezil vs Placebo	1.04	0.54 to 1.99	0.34 to 3.16	0.49
Rivastigmine oral vs Placebo	0.81	0.37 to 1.80	0.24 to 2.79	0.72
Galantamine vs Placebo	1.05	0.70 to 1.59	0.42 to 2.65	0.48
Rivastigmine transdermal vs Placebo	0.92	0.37 to 2.27	0.24 to 3.52	0.58
Memantine vs Placebo	1.40	0.80 to 2.48	0.50 to 3.98	0.19
Placebo (reference)				0.55
Common within-network between-study variance: τ^2 = 0.11, I^2 = 51% (0%, 77%)				
Design-by-treatment interaction model for inconsistency χ^2 (d.f., P-value, τ^2): N/A (no closed loops)				
**Odds Ratio: NMA of studies with IPD adjusted for MMSE baseline**				
Donepezil vs Placebo	0.97	0.46 to 2.06	0.23 to 4.03	0.56
Rivastigmine oral vs Placebo	0.81	0.33 to 2.01	0.17 to 3.91	0.70
Galantamine vs Placebo	1.29	0.74 to 2.25	0.37 to 4.55	0.28
Rivastigmine transdermal vs Placebo	0.93	0.34 to 2.53	0.18 to 4.91	0.57
Memantine vs Placebo	1.26	0.59 to 2.70	0.30 to 5.28	0.33
Placebo (reference)				0.56
Common within-network between-study variance: τ^2 = 0.16, I^2 = 52% (0%, 80%)				
Design-by-treatment interaction model for inconsistency χ^2 (d.f., P-value, τ^2): N/A (no closed loops)				
**Odds Ratio: NMA of studies with IPD adjusted for comorbidities**				
Donepezil vs Placebo	1.01	0.52 to 1.96	0.29 to 3.50	0.51
Rivastigmine oral vs Placebo	0.82	0.36 to 1.87	0.20 to 3.32	0.69
Galantamine vs Placebo	1.02	0.57 to 1.80	0.32 to 3.26	0.50
Rivastigmine transdermal vs Placebo	0.91	0.36 to 2.31	0.20 to 4.11	0.58
Memantine vs Placebo	1.42	0.79 to 2.55	0.44 to 4.59	0.18
Placebo (reference)				0.53
Common within-network between-study variance: τ^2 = 0.12, I^2 = 44% (0%, 77%)				
Design-by-treatment interaction model for inconsistency χ^2 (d.f., P-value, τ^2): N/A (no closed loops)				
**Odds Ratio: NMA of studies with IPD adjusted for other medications**				
Donepezil vs Placebo	1.17	0.49 to 3.03	0.28 to 4.88	0.41
Rivastigmine oral vs Placebo	0.82	0.37 to 1.81	0.23 to 2.91	0.72
Galantamine vs Placebo	1.03	0.69 to 1.55	0.40 to 2.65	0.51
Rivastigmine transdermal vs Placebo	0.95	0.39 to 2.34	0.24 to 2.91	0.56
Memantine vs Placebo	1.34	0.75 to 2.39	0.46 to 3.92	0.25
Placebo (reference)				0.56
Common within-network between-study variance: τ^2 = 0.11, I^2 = 51% (0%, 78%)				
Design-by-treatment interaction model for inconsistency χ^2 (d.f., P-value, τ^2): N/A (no closed loops)				
**Odds Ratio: Meta-regression, Study Duration[**]**				
Donepezil vs Placebo	1.12	0.87 to 1.43	0.63 to 1.95	0.25 [††]
Rivastigmine oral vs Placebo	1.76	1.00 to 2.99	0.88 to 3.68	0.00 [††]
Galantamine vs Placebo	0.92	0.62 to 1.36	0.50 to 1.69	0.50 [††]
Rivastigmine transdermal vs Placebo	0.87	0.39 to 1.70	0.34 to 1.96	0.63 [††]
Memantine vs Placebo	0.61	0.37 to 0.93	0.31 to 1.13	0.88 [††]
Donepezil + Memantine vs Placebo	0.76	0.29 to 1.69	0.26 to 1.90	0.75 [††]
Galantamine + Memantine vs Placebo	0.98	0.34 to 2.26	0.30 to 2.53	0.50 [††]
Rivastigmine transdermal + Memantine vs Placebo	0.75	0.25 to 1.81	0.23 to 1.97	0.75 [††]
Placebo (reference)				0.38 [††]
Regression coefficient (log-scale)	0.00	0.00 to 0.01		
Common within-network between-study variance: τ^2 = 0.03	0.00 to 0.22			
Design-by-treatment interaction model for inconsistency χ^2 (d.f., P-value, τ^2): 3.57 (6, 0.735, 0.06)				
**Odds Ratio: Meta-regression, Year of Publication[**]**				
Donepezil vs Placebo	1.05	0.79 to 1.38	0.61 to 1.77	0.38 [††]
Rivastigmine oral vs Placebo	1.68	0.98 to 2.77	0.85 to 3.37	0.00 [††]
Galantamine vs Placebo	0.91	0.61 to 1.32	0.50 to 1.64	0.63 [††]
Rivastigmine transdermal vs Placebo	0.92	0.40 to 1.84	0.36 to 2.04	0.63 [††]
Memantine vs Placebo	0.73	0.46 to 1.05	0.38 to 1.28	0.88 [††]
Donepezil + Memantine vs Placebo	0.88	0.35 to 1.83	0.31 to 2.15	0.75 [††]

For peer review only - http://bmjopen.bmj.com/site/about/guidelines.xhtml

Galantamine + Memantine vs Placebo	1.24	0.43 to 2.85	0.39 to 3.25	0.25 ††
Rivastigmine transdermal + Memantine vs Placebo	0.88	0.24 to 2.24	0.24 to 2.42	0.75 ††
Placebo (reference)				0.38 ††
Regression coefficient (log-scale)	-0.02	-0.06 to 0.03		
Common within-network between-study variance: $\tau^2 = 0.02$	0.00 to 0.21			
Design-by-treatment interaction model for inconsistency χ^2 *(d.f., P-value,* τ^2*)*: 3.57 (6, 0.735, 0.06)				

* Aggregate data and fully adjusted results from studies with available individual patient data
† MMSE: Studies with available IPD included only available participants – to assess the missing data impact on the second stage a separate analysis was applied (IMDoM)
‡ SAE: Studies with available IPD included all randomized participants
§ Outlier studies:

- Hernandez C, Unturbe F, Martinez-Lage P, Lucas A, Gregorio P, Alonso T. Effects of combined pharmacologic and cognitive treatment in the progression of moderate dementia: a two-year follow-up. REVISTA ESPANOLA DE GERIATRIA Y GERONTOLOGIA. 2007;42(1):3
- Moretti DV. Alpha rhythm oscillations and MMSE scores are differently modified by transdermal or oral rivastigmine in patients with Alzheimer's disease. American journal of neurodegenerative disease. 2014;3(2):72-83.

¶ Included studies with available raw data only, irrespective having access to individual patient data
|| Analyses were conducted in Stata using the *metamiss2* and *network* commands; I2 is not available; SUCRA values are presented instead of P-scores
** Studies with aggregate data were used (studies with available individual patient data were not included in this analysis)
†† Analyses were conducted in OpenBUGS, and SUCRA values were calculated instead of P-scores

For peer review only - http://bmjopen.bmj.com/site/about/guidelines.xhtml

Appendix 18: CINeMA results

Risk of bias contributions: The bar chart shows the contributions of each piece of study to the network estimate

Interventions
A: PLAC
B: DONE
C: RIVA_O
D: GALA
E: RIVA_P
F: MEMA
G: DONE+MEMA
H: GALA+MEMA
I: RIVA_P+MEMA

MMSE outcome

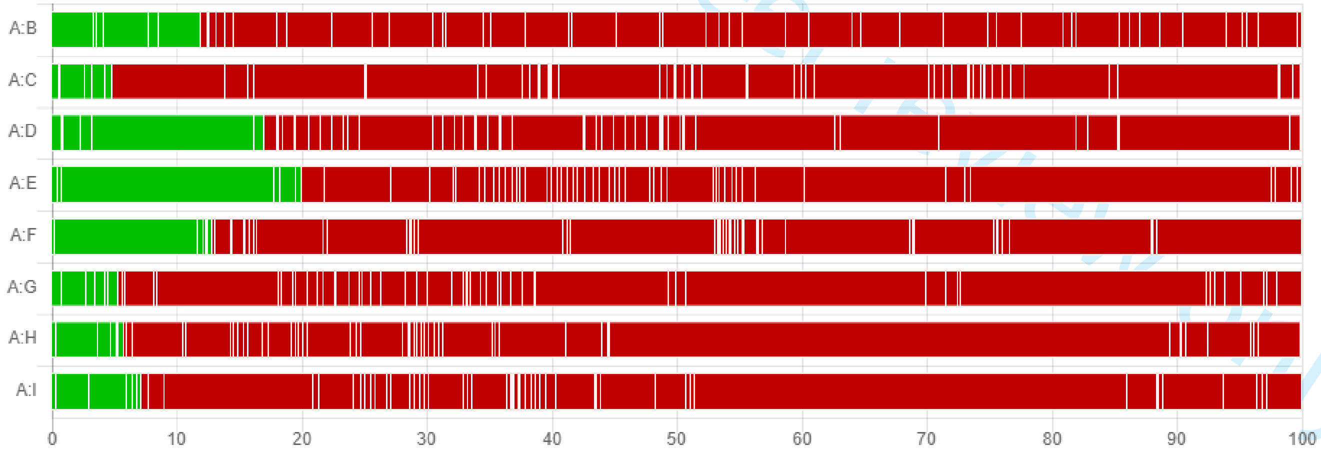

SAE outcome

For peer review only - http://bmjopen.bmj.com/site/about/guidelines.xhtml

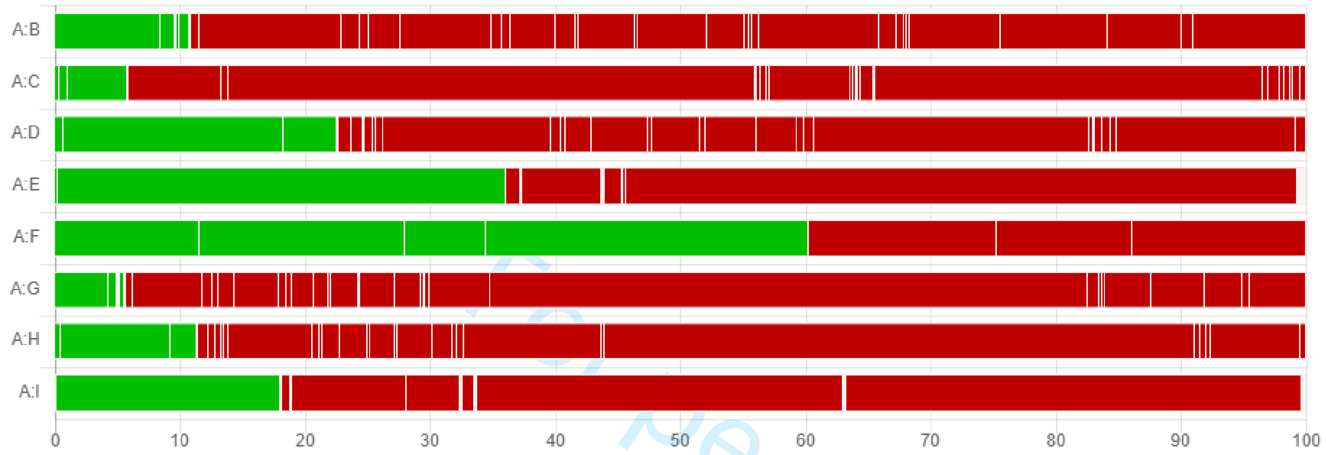

CINeMA report

MMSE outcome

Comparison	# of studies	Nature of evidence	Type of data	Within-study bias (D1)	Reporting bias (D2)	Indirectness (D3)	Imprecision (D4)	Heterogeneity (D5)	Incoherence (D6)	Confidence rating	Downgrading due to
DONE vs PLAC	24	Mixed	IPD+AD	Major concerns	Suspected	No concerns	No concerns	Major concerns	No concerns	Moderate	D5
RIVA_O vs PLAC	6	Mixed	IPD+AD	Major concerns	Suspected	No concerns	Some concerns	Some concerns	No concerns	Moderate	D4;D5
GALA vs PLAC	3	Mixed	IPD+AD	Major concerns	Suspected	No concerns	Major concerns	No concerns	No concerns	Moderate	D4
RIVA_P vs PLAC	2	Mixed	IPD+AD	Major concerns	Suspected	No concerns	Some concerns	Some concerns	No concerns	Moderate	D4;D5
MEMA vs PLAC	7	Mixed	IPD+AD	Major concerns	Suspected	No concerns	Some concerns	Some concerns	No concerns	Moderate	D4;D5
DONE+MEMA vs PLAC	1	Mixed	AD	Major concerns	Suspected	No concerns	No concerns	Major concerns	No concerns	Moderate	D5
GALA+MEMA vs PLAC	0	Indirect	-	Major concerns	Suspected	No concerns	Major concerns	No concerns	No concerns	Moderate	D4
RIVA_P+MEMA vs PLAC	0	Indirect	-	Major concerns	Suspected	No concerns	Major concerns	No concerns	No concerns	Moderate	D4

SAE outcome

Comparison	# of	Nature of	Type of	Within-study	Reporting bias	Indirectness	Imprecision	Heterogeneity	Incoherence	Confidence	Downgrading

For peer review only - http://bmjopen.bmj.com/site/about/guidelines.xhtml

	studies	evidence	data	bias (D1)	(D2)	(D3)	(D4)	(D5)	(D6)	rating	due to
DONE vs PLAC	16	Mixed	IPD+AD	Major concerns	Undetected	No concerns	Major concerns	No concerns	No concerns	Moderate	D1
RIVA_O vs PLAC	3	Mixed	IPD+AD	Major concerns	Undetected	No concerns	Major concerns	No concerns	No concerns	Moderate	D1
GALA vs PLAC	8	Mixed	IPD+AD	Major concerns	Undetected	No concerns	Major concerns	No concerns	No concerns	Moderate	D1
RIVA_P vs PLAC	2	Mixed	IPD+AD	Some concerns	Undetected	No concerns	Major concerns	No concerns	No concerns	High	
MEMA vs PLAC	7	Mixed	IPD+AD	Some concerns	Undetected	No concerns	Major concerns	No concerns	No concerns	High	
DONE+MEMA vs PLAC	2	Mixed	AD	Major concerns	Undetected	No concerns	Major concerns	No concerns	No concerns	Moderate	D1
GALA+MEMA vs PLAC	0	Indirect	-	Major concerns	Undetected	No concerns	Major concerns	No concerns	No concerns	Moderate	D1
RIVA_P+MEMA vs PLAC	0	Indirect	-	Major concerns	Undetected	No concerns	Major concerns	No concerns	No concerns	Moderate	D1

Abbreviations: DONE, donepezil; GALA, galantamine; MEMA, memantine; PLAC, placebo; RIVA_O, rivastigmine oral; RIVA_P, rivastigmine patch

For peer review only

For peer review only - http://bmjopen.bmj.com/site/about/guidelines.xhtml

Appendix 19: Rank-heat plot for serious adverse events

Circles from inside out present results for different network meta-analyses including: i) aggregate data (AD) only (studies with available IPD are not included in the analysis), ii) crude results from individual studies with individual patient data (IPD), iii) AD and crude results from studies with available IPD, and iv) AD and fully adjusted results from studies with available IPD. Numbers within each sector correspond to the P-score values as calculated in each model.

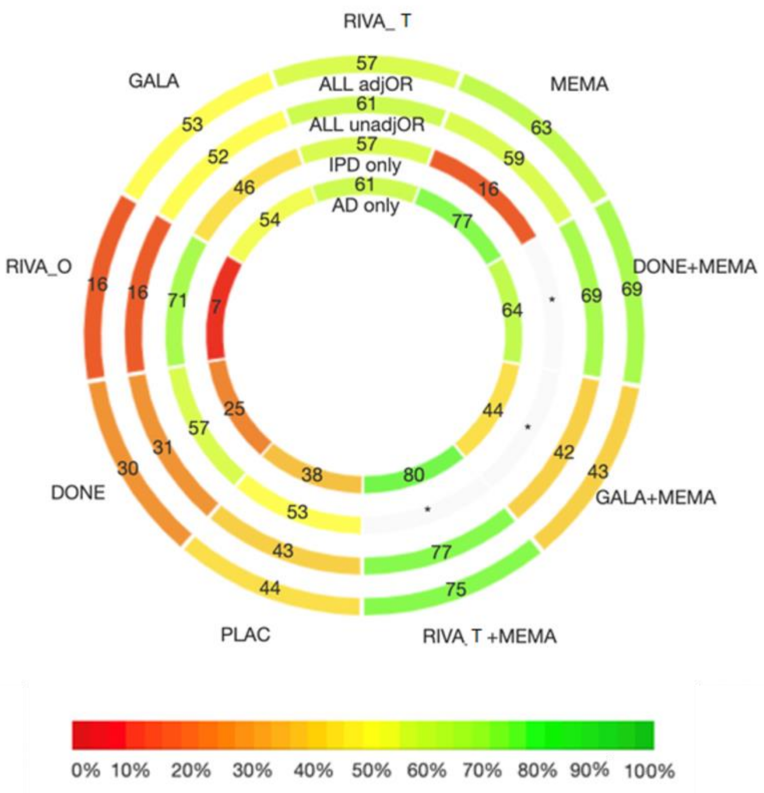

For peer review only - http://bmjopen.bmj.com/site/about/guidelines.xhtml

Appendix 20: Study definitions for serious adverse events

Author, Year	Source of Definition	Definition
Agid, 1998	Determined by Investigator	"Patients and caregivers were questioned systematically regarding the occurrence of adverse events at each clinical visit"
Ancoli-Israel, 2005	Determined by Investigator	"Only one serious AE leading to discontinuation, hepatic failure, in the donepezil-treated group was considered to be possibly due to study treatment by the investigator."
Andersen, 2012	NA	NA
Araki, 2014	NA	NA
Bakchine, 2008	Determined by Investigator	"Three patients had an SAE that was considered by the investigator to be possibly or probably related to treatment.”
Black, 2007	Determined by Investigator	"AEs were considered serious (SAEs) when death occurred, life was threatened, hospitalization or prolonged hospitalization was required, or a significant disability occurred."
Blesa González, 2011	NA	NA
Burns,1999	COSTART	"Events were coded using a modified COSTART dictionary, and the assessment of relationship to treatment for all adverse events was conducted blind to treatment assignment."
Burns, 2009	NR	NR
Burns, 2011	NR	NR
Choi, 2011	Determined by Investigator	"Investigators were asked to evaluate severity (mild, moderate, or severe), relationship to study drug (not related, probable relationship with rivastigmine patch, probable relationship with memantine, or probable relationship with an interaction of the two drugs), and seriousness of the AEs."
Corey-Bloom, 1998	NA	NA
Cretu, 2008	NA	NA
Dysken, 2014	Medical Dictionary for Regulatory Activities	"Serious AEs were coded according to the Medical Dictionary for Regulatory Activities."
Farlow, 2013	NA	NA
Feldman, 2001	Determined by Investigator	"Serious AE was defined as any AE that was life threatening or resulted in death, hospitalization, prolongation of hospitalization, or significant disability."
Feldman, 2007	World Health Organisation preferred terms	“"All adverse events were recorded using the Novartis Medical Terminology Thesaurus (a modified version of the WHO adverse reaction terminology dictionary)."
Fox, 2012	NA	NA
Frolich, 2011	NA	NA
Fuschillo, 2001	NA	NA
Gault L, 2015	Medical Dictionary for Regulatory Activities	"AEs were coded using the Medical Dictionary for Regulatory Activities"
Gold, 2010	NR	NR
Greenberg, 2000	Determined by Investigator	“Of 9 withdrawals from the study after randomization, 2 were due to serious adverse events judged to be possibly related to donepezil therapy: syncope and generalized seizure (1 patient each).”
Grossberg, 2013	Medical Dictionary for Regulatory Activities	“Adverse events were coded according to the Medical Dictionary for Regulatory Activities (version 7.0 or newer), and an assessment of the severity, chronicity, causal relationship to study medication, and seriousness of the event was provided by an investigator”
Hager, 2014	Determined by Investigator	“Safety data were monitored during the study by a company-commissioned, external, independent, blinded Data Safety Monitoring Board (DSMB). Secondary safety outcomes were the number of treatment emergent adverse events (TEAEs), including serious TEAEs.”
Haig, 2014	NR	NR
Hernández, 2007	NA	NA
Herrmann, 2013	Determined by Investigator	“The incidence of adverse events considered related to the study drug by the investigator was 30% in the placebo group and 36% in the memantine group”
Holmes, 2004		
Homma, 1998	NR	NR
Homma, 2008	Medical Dictionary for Regulatory Activities – Japanese Version	"AE terms were standardized according to the Medical Dictionary for Regulatory Activities – Japanese Version . AEs were graded on a 3-point scale (mild: discomfort noticed, but no disruption of normal daily activity; moderate: discomfort sufficient to reduce or affect normal daily activity; severe: incapacitating, with inability to work or to perform normal daily activity). "
Hong, 2006	NR	NR
Howard, 2007	NA	NA
Howard, 2012	NR	NR
Hu, 2006	NA	NA

For peer review only - http://bmjopen.bmj.com/site/about/guidelines.xhtml

Johannsen, 2006	NA	NA
Jones, 2004	Determined by Investigator	“A serious adverse event (SAE) was defined as any AE that was life threatening or resulted in death, hospitalisation, prolongation of hospitalisation, or significant disability”
Kadir, 2008	NA	NA
Kano, 2013	NA	NA
Karaman, 2005	NA	NA
Likitjaroen, 2012	NA	NA
Lorenzi, 2011	NA	NA
Maher-Edwards, 2011	Determined by Investigator	“Eight subjects experienced nonfatal serious AEs; all were considered unrelated to the study drug”
Marek, 2014	Medical Dictionary for Regulatory Activities	"Aes were coded using the Medical Dictionary for Regulatory Activities (MedDRA, version 14.0) by system organ class and preferred term"
Mazza, 2006	NA	NA
Mohs, 2001	Determined by Investigator	“In all cases, judgment of the relationship of study treatment to an adverse event and of the severity of the event was made by the investigator under double-blind conditions.”
Moretti, 2014	NA	NA
Mowla, 2007	NA	NA
Nakamura, 2011	Determined by Investigator	“Safety evaluations included recording all adverse events on Adverse Event Case Report Forms. Every serious adverse event occurring after the patient provided informed consent and until 28 days after the patient stopped the study was reported.”
Nakano, 2001	NA	NA
Nordberg, 2009	Determined by Investigator	“Safety and tolerability were monitored throughout the study by recording all adverse events (AEs).”
Pakdaman H, 2015	NA	NA
Peng, 2005	NA	NA
Peskind, 2006	NR	NR
Peters O, 2015	NR	NR
Reisberg, 2003	NR	NR
Rockwood, 2001	World Health Organisation preferred terms	"adverse events (classified according to World Health Organisation preferred terms)."
Rockwood, 2006	NR	NR
Rogers, 1996		
Rogers, 1998	COSTART	"Events, recorded using investigator terminology, were grouped and coded into common terms using a modified COSTART dictionary"
Rogers, 1998	COSTART	“Events, recorded using investigator terminology, were grouped and coded into common terms using a modified COSTART dictionary.”
Saxton, 2012	Determined by Investigator	“Treatment-emergent adverse events (TEAEs) and serious adverse events (SAEs) were recorded at all post-Screening study visits”
Scarpini, 2011	NR	NR
Schmidt, 2008	NA	NA
Seltzer, 2004	NA	NA
Shao, 2015	NA	NA
Shimizu, 2015	NA	NA
Sole-Padulles, 2013	NA	NA
Tariot, 2000	NR	NR
Tariot, 2001	COSTART	“Investigator terms describing AEs were coded to standard preferred terms using a modified Coding Symbols for Thesaurus of Adverse Reaction Terms dictionary.”
Thomas, 2001		
Wilcock, 2003	World Health Organisation preferred terms	“monitoring for adverse events (classified according to WHO preferred terms)”
Wilkinson, 2001	Determined by Investigator	“All adverse events were recorded, regardless of the considered relationship to treatment. All details of adverse events and their outcomes were recorded including severity and relationship to treatment. Serious adverse events were documented separately.”
Wilkinson, 2002	NR	NR
Wilkinson, 2012	NR	NR
Winblad, 2001	NR	NR
Winblad, 2006	COSTART	“We recorded all treatment emergent adverse events, coding them according to a modified COSTART dictionary.”
Winblad, 2007	Determined by Investigator	"Safety evaluations included recording all adverse events, which were coded using a standard glossary."
Zhang-Yi, 2005	NA	NA
Zhang, 2012	Determined by Investigator	“Serious adverse events considered to be possibly related to treatment occurred in one patient in each treatment arm”

For peer review only - http://bmjopen.bmj.com/site/about/guidelines.xhtml

Notes: [a]Unpublished data, [b]Non-English studies
Abbreviations: CR, companion report; NA, not applicable; NR, not reported.

For peer review only

For peer review only - http://bmjopen.bmj.com/site/about/guidelines.xhtml

Appendix 21: Time taken to achieve at least one serious adverse event using individual patient data

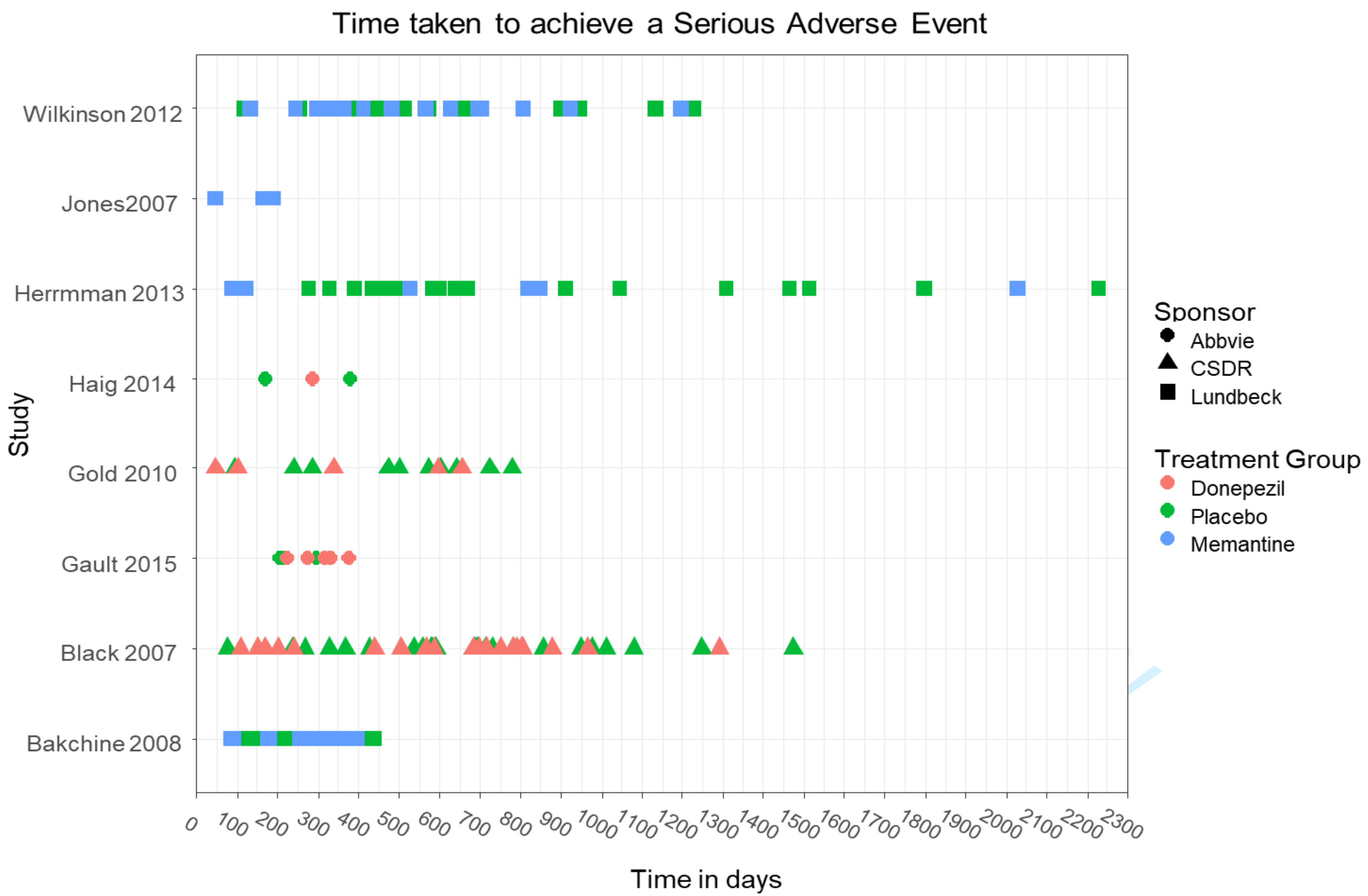

61

Appendix 22: Challenges encountered during the individual patient data request from sponsors

- The identification of the trial data set when certain details were not available (e.g. NCT number; particularly for studies published before 2005 that this was established).
- Data ownership.
- Sponsors switched platforms, while we were navigating the data.
- IPD available through proprietary sponsor-specific platforms did not allow for combination of IPD from different sponsor platforms; hence a one-stage analysis as planned in our protocol, was impossible.
- Software availability: Required R packages (e.g., mice) were not available/provided, and we were not allowed to install any new R packages; some R packages were older versions (e.g. lme4).
- Time that the platform permitted access to the IPD was often limited. This is a significant constraint given that IPD from different studies became available at different time points.
- Cost associated with obtaining access to the data for a certain amount of time. Additionally, cost associated with the WHO Drug Dictionary license to obtain access to the additional medications used for each patient; this license's approximate cost was $8,958·25 USD per sponsor.
- Available IPD did not include the full information as shown in the publication: For example, only data for placebo were available, or did not give information about a reported outcome (e.g. only baseline MMSE values were available). Also, date of follow-up was coded in some studies and it was impossible to make a judgement on first and last date.

For peer review only - http://bmjopen.bmj.com/site/about/guidelines.xhtml

PRISMA-IPD Checklist of items to include when reporting a systematic review and meta-analysis of individual participant data (IPD)

PRISMA-IPD Section/topic	Item No	Checklist item	Reported on page
**Title**			
Title	1	Identify the report as a systematic review and meta-analysis of individual participant data.	1
**Abstract**			
Structured summary	2	Provide a structured summary including as applicable:	3-4
		**Background**: state research question and main objectives, with information on participants, interventions, comparators and outcomes.	
		**Methods**: report eligibility criteria; data sources including dates of last bibliographic search or elicitation, noting that IPD were sought; methods of assessing risk of bias.	
		**Results**: provide number and type of studies and participants identified and number (%) obtained; summary effect estimates for main outcomes (benefits and harms) with confidence intervals and measures of statistical heterogeneity. Describe the direction and size of summary effects in terms meaningful to those who would put findings into practice.	
		**Discussion:** state main strengths and limitations of the evidence, general interpretation of the results and any important implications.	
		**Other:** report primary funding source, registration number and registry name for the systematic review and IPD meta-analysis.	
**Introduction**			
Rationale	3	Describe the rationale for the review in the context of what is already known.	5
Objectives	4	Provide an explicit statement of the questions being addressed with reference, as applicable, to participants, interventions, comparisons, outcomes and study design (PICOS). Include any hypotheses that relate to particular types of participant-level subgroups.	5
**Methods**			

Protocol and registration	5	Indicate if a protocol exists and where it can be accessed. If available, provide registration information including registration number and registry name. Provide publication details, if applicable.	5, Appendix 1
Eligibility criteria	6	Specify inclusion and exclusion criteria including those relating to participants, interventions, comparisons, outcomes, study design and characteristics (e.g. years when conducted, required minimum follow-up). Note whether these were applied at the study or individual level i.e. whether eligible participants were included (and ineligible participants excluded) from a study that included a wider population than specified by the review inclusion criteria. The rationale for criteria should be stated.	5-6, Appendix 1
Identifying studies - information sources	7	Describe all methods of identifying published and unpublished studies including, as applicable: which bibliographic databases were searched with dates of coverage; details of any hand searching including of conference proceedings; use of study registers and agency or company databases; contact with the original research team and experts in the field; open adverts and surveys. Give the date of last search or elicitation.	6, Appendix 1
Identifying studies - search	8	Present the full electronic search strategy for at least one database, including any limits used, such that it could be repeated.	N/A (see published protocol)
Study selection processes	9	State the process for determining which studies were eligible for inclusion.	6, Appendix 1
Data collection processes	10	Describe how IPD were requested, collected and managed, including any processes for querying and confirming data with investigators. If IPD were not sought from any eligible study, the reason for this should be stated (for each such study).<br>If applicable, describe how any studies for which IPD were not available were dealt with. This should include whether, how and what aggregate data were sought or extracted from study reports and publications (such as extracting data independently in duplicate) and any processes for obtaining and confirming these data with investigators.	6, Appendix 1
Data items	11	Describe how the information and variables to be collected were chosen. List and define all study level and participant level data that were sought, including baseline and follow-up information. If applicable, describe methods of standardising or translating variables within the IPD datasets to ensure common scales or measurements across studies.	6, Appendix 1

For peer review only

IPD integrity	A1	Describe what aspects of IPD were subject to data checking (such as sequence generation, data consistency and completeness, baseline imbalance) and how this was done.	Appendix 1
Risk of bias assessment in individual studies.	12	Describe methods used to assess risk of bias in the individual studies and whether this was applied separately for each outcome. If applicable, describe how findings of IPD checking were used to inform the assessment. Report if and how risk of bias assessment was used in any data synthesis.	6, Appendix 1
Specification of outcomes and effect measures	13	State all treatment comparisons of interests. State all outcomes addressed and define them in detail. State whether they were pre-specified for the review and, if applicable, whether they were primary/main or secondary/additional outcomes. Give the principal measures of effect (such as risk ratio, hazard ratio, difference in means) used for each outcome.	7, Appendix 1
Synthesis methods	14	Describe the meta-analysis methods used to synthesise IPD. Specify any statistical methods and models used. Issues should include (but are not restricted to):<br>• Use of a one-stage or two-stage approach.<br>• How effect estimates were generated separately within each study and combined across studies (where applicable).<br>• Specification of one-stage models (where applicable) including how clustering of patients within studies was accounted for.<br>• Use of fixed or random effects models and any other model assumptions, such as proportional hazards.<br>• How (summary) survival curves were generated (where applicable).<br>• Methods for quantifying statistical heterogeneity (such as I^2 and τ^2).<br>• How studies providing IPD and not providing IPD were analysed together (where applicable).<br>• How missing data within the IPD were dealt with (where applicable).	7, Appendix 1
Exploration of variation in effects	A2	If applicable, describe any methods used to explore variation in effects by study or participant level characteristics (such as estimation of interactions between effect and covariates). State all participant-level characteristics that were analysed as potential effect modifiers, and whether these were pre-specified.	Appendix 1
Risk of bias across studies	15	Specify any assessment of risk of bias relating to the accumulated body of evidence, including any pertaining to not obtaining IPD for particular studies, outcomes or other variables.	6, Appendix 1

For peer review only

Additional analyses	16	Describe methods of any additional analyses, including sensitivity analyses. State which of these were pre-specified.	7, Appendix 1
**Results**			
Study selection and IPD obtained	17	Give numbers of studies screened, assessed for eligibility, and included in the systematic review with reasons for exclusions at each stage. Indicate the number of studies and participants for which IPD were sought and for which IPD were obtained. For those studies where IPD were not available, give the numbers of studies and participants for which aggregate data were available. Report reasons for non-availability of IPD. Include a flow diagram.	7 – Figure 1
Study characteristics	18	For each study, present information on key study and participant characteristics (such as description of interventions, numbers of participants, demographic data, unavailability of outcomes, funding source, and if applicable duration of follow-up). Provide (main) citations for each study. Where applicable, also report similar study characteristics for any studies not providing IPD.	8 – Table 1, Appendix 5
IPD integrity	A3	Report any important issues identified in checking IPD or state that there were none.	8-9, Appendices 5 and 10
Risk of bias within studies	19	Present data on risk of bias assessments. If applicable, describe whether data checking led to the up-weighting or down-weighting of these assessments. Consider how any potential bias impacts on the robustness of meta-analysis conclusions.	8-9 – Appendix 8
Results of individual studies	20	For each comparison and for each main outcome (benefit or harm), for each individual study report the number of eligible participants for which data were obtained and show simple summary data for each intervention group (including, where applicable, the number of events), effect estimates and confidence intervals. These may be tabulated or included on a forest plot.	Appendices 6 and 10 (full data can be provided by the

For peer review only

			first author)
Results of syntheses	21	Present summary effects for each meta-analysis undertaken, including confidence intervals and measures of statistical heterogeneity. State whether the analysis was pre-specified, and report the numbers of studies and participants and, where applicable, the number of events on which it is based.	9-11 – Appendix 15
		When exploring variation in effects due to patient or study characteristics, present summary interaction estimates for each characteristic examined, including confidence intervals and measures of statistical heterogeneity. State whether the analysis was pre-specified. State whether any interaction is consistent across trials.	
		Provide a description of the direction and size of effect in terms meaningful to those who would put findings into practice.	
Risk of bias across studies	22	Present results of any assessment of risk of bias relating to the accumulated body of evidence, including any pertaining to the availability and representativeness of available studies, outcomes or other variables.	9-11 - Appendix 12
Additional analyses	23	Give results of any additional analyses (e.g. sensitivity analyses). If applicable, this should also include any analyses that incorporate aggregate data for studies that do not have IPD. If applicable, summarise the main meta-analysis results following the inclusion or exclusion of studies for which IPD were not available.	9-11 - Appendices 16 and 17
**Discussion**			
Summary of evidence	24	Summarise the main findings, including the strength of evidence for each main outcome.	11-13
Strengths and limitations	25	Discuss any important strengths and limitations of the evidence including the benefits of access to IPD and any limitations arising from IPD that were not available.	13-14
Conclusions	26	Provide a general interpretation of the findings in the context of other evidence.	13-14
Implications	A4	Consider relevance to key groups (such as policy makers, service providers and service users). Consider implications for future research.	12-13

For peer review only

Funding			
Funding	27	Describe sources of funding and other support (such as supply of IPD), and the role in the systematic review of those providing such support.	15

A1 – A3 denote new items that are additional to standard PRISMA items. A4 has been created as a result of re-arranging content of the standard PRISMA statement to suit the way that systematic review IPD meta-analyses are reported.

© Reproduced with permission of the PRISMA IPD Group, which encourages sharing and reuse for non-commercial purposes

For peer review only

PRISMA NMA Checklist of Items to Include When Reporting A Systematic Review Involving a Network Meta-analysis

Section/Topic	Item #	Checklist Item	Reported on Page #
**TITLE**			
Title	1	Identify the report as a systematic review *incorporating a network meta-analysis (or related form of meta-analysis).*	1
**ABSTRACT**			
Structured summary	2	Provide a structured summary including, as applicable:<br>**Background:** main objectives<br>**Methods:** data sources; study eligibility criteria, participants, and interventions; study appraisal; and *synthesis methods, such as network meta-analysis.*<br>**Results:** number of studies and participants identified; summary estimates with corresponding confidence/credible intervals; *treatment rankings may also be discussed. Authors may choose to summarize pairwise comparisons against a chosen treatment included in their analyses for brevity.*<br>**Discussion/Conclusions:** limitations; conclusions and implications of findings.<br>**Other:** primary source of funding; systematic review registration number with registry name.	3-4
**INTRODUCTION**			
Rationale	3	Describe the rationale for the review in the context of what is already known*, including mention of why a network meta-analysis has been conducted.*	5
Objectives	4	Provide an explicit statement of questions being addressed, with reference to participants, interventions, comparisons, outcomes, and study design (PICOS).	5
**METHODS**			
Protocol and registration	5	Indicate whether a review protocol exists and if and where it can be accessed (e.g., Web address); and, if available, provide registration information, including registration number.	5, Appendix 1
Eligibility criteria	6	Specify study characteristics (e.g., PICOS, length of follow-up) and report characteristics (e.g., years considered, language, publication status) used as criteria for eligibility, giving rationale. *Clearly describe eligible treatments included in the treatment network, and note whether any have been clustered or merged into the same node (with justification).*	6, Appendix 1
Information sources	7	Describe all information sources (e.g., databases with dates of coverage, contact with study authors to identify additional studies) in the search and date last searched.	6, Appendix 1

For peer review only

Search	8	Present full electronic search strategy for at least one database, including any limits used, such that it could be repeated.	N/A (see published protocol)
Study selection	9	State the process for selecting studies (i.e., screening, eligibility, included in systematic review, and, if applicable, included in the meta-analysis).	6, Appendix 1
Data collection process	10	Describe method of data extraction from reports (e.g., piloted forms, independently, in duplicate) and any processes for obtaining and confirming data from investigators.	6, Appendix 1
Data items	11	List and define all variables for which data were sought (e.g., PICOS, funding sources) and any assumptions and simplifications made.	6, Appendix 1
**Geometry of the network**	**S1**	Describe methods used to explore the geometry of the treatment network under study and potential biases related to it. This should include how the evidence base has been graphically summarized for presentation, and what characteristics were compiled and used to describe the evidence base to readers.	7, Appendix 1
Risk of bias within individual studies	12	Describe methods used for assessing risk of bias of individual studies (including specification of whether this was done at the study or outcome level), and how this information is to be used in any data synthesis.	6, Appendix 1
Summary measures	13	State the principal summary measures (e.g., risk ratio, difference in means). *Also describe the use of additional summary measures assessed, such as treatment rankings and surface under the cumulative ranking curve (SUCRA) values, as well as modified approaches used to present summary findings from meta-analyses.*	7, Appendix 1
Planned methods of analysis	14	Describe the methods of handling data and combining results of studies for each network meta-analysis. This should include, but not be limited to:<br>• *Handling of multi-arm trials;*<br>• *Selection of variance structure;*<br>• *Selection of prior distributions in Bayesian analyses; and*<br>• *Assessment of model fit.*	7, Appendix 1
**Assessment of Inconsistency**	**S2**	Describe the statistical methods used to evaluate the agreement of direct and indirect evidence in the treatment network(s) studied. Describe efforts taken to address its presence when found.	7, Appendix 1
Risk of bias across studies	15	Specify any assessment of risk of bias that may affect the cumulative evidence (e.g., publication bias, selective reporting within studies).	6, Appendix 1
Additional analyses	16	Describe methods of additional analyses if done, indicating which were pre-specified. This may include, but not be limited to, the following:<br>• Sensitivity or subgroup analyses;<br>• Meta-regression analyses;<br>• *Alternative formulations of the treatment network; and*<br>• *Use of alternative prior distributions for Bayesian analyses (if applicable).*	7, Appendix 1

For peer review only

RESULTS†

Study selection	17	Give numbers of studies screened, assessed for eligibility, and included in the review, with reasons for exclusions at each stage, ideally with a flow diagram.	7 – Figure 1
**Presentation of network structure**	**S3**	Provide a network graph of the included studies to enable visualization of the geometry of the treatment network.	9 – Figure 2
**Summary of network geometry**	**S4**	Provide a brief overview of characteristics of the treatment network. This may include commentary on the abundance of trials and randomized patients for the different interventions and pairwise comparisons in the network, gaps of evidence in the treatment network, and potential biases reflected by the network structure.	7-8
Study characteristics	18	For each study, present characteristics for which data were extracted (e.g., study size, PICOS, follow-up period) and provide the citations.	8 – Table 1, Appendix 5
Risk of bias within studies	19	Present data on risk of bias of each study and, if available, any outcome level assessment.	8-9 – Appendix 8
Results of individual studies	20	For all outcomes considered (benefits or harms), present, for each study: 1) simple summary data for each intervention group, and 2) effect estimates and confidence intervals. *Modified approaches may be needed to deal with information from larger networks.*	Appendices 6 and 10 (full data can be provided by the first author)
Synthesis of results	21	Present results of each meta-analysis done, including confidence/credible intervals. *In larger networks, authors may focus on comparisons versus a particular comparator (e.g. placebo or standard care), with full findings presented in an appendix. League tables and forest plots may be considered to summarize pairwise comparisons.* If additional summary measures were explored (such as treatment rankings), these should also be presented.	9-11 – Appendix 15
**Exploration for inconsistency**	**S5**	Describe results from investigations of inconsistency. This may include such information as measures of model fit to compare consistency and inconsistency models, *P* values from statistical tests, or summary of inconsistency estimates from different parts of the treatment network.	9 - Appendix 14
Risk of bias across studies	22	Present results of any assessment of risk of bias across studies for the evidence base being studied.	9-11 - Appendix 12
Results of additional analyses	23	Give results of additional analyses, if done (e.g., sensitivity or subgroup analyses, meta-regression analyses*, alternative network geometries studied, alternative choice of prior distributions for Bayesian analyses,* and so forth).	9-11 - Appendices 16 and 17

**DISCUSSION**			
Summary of evidence	24	Summarize the main findings, including the strength of evidence for each main outcome; consider their relevance to key groups (e.g., healthcare providers, users, and policy-makers).	11-13
Limitations	25	Discuss limitations at study and outcome level (e.g., risk of bias), and at review level (e.g., incomplete retrieval of identified research, reporting bias). *Comment on the validity of the assumptions, such as transitivity and consistency. Comment on any concerns regarding network geometry (e.g., avoidance of certain comparisons).*	13-14
Conclusions	26	Provide a general interpretation of the results in the context of other evidence, and implications for future research.	13-14
**FUNDING**			
Funding	27	Describe sources of funding for the systematic review and other support (e.g., supply of data); role of funders for the systematic review. This should also include information regarding whether funding has been received from manufacturers of treatments in the network and/or whether some of the authors are content experts with professional conflicts of interest that could affect use of treatments in the network.	15

PICOS = population, intervention, comparators, outcomes, study design.
* Text in italics indicateS wording specific to reporting of network meta-analyses that has been added to guidance from the PRISMA statement.
† Authors may wish to plan for use of appendices to present all relevant information in full detail for items in this section.

For peer review only

BMJ Open

Comparative safety and efficacy of cognitive enhancers for Alzheimer's dementia: A systematic review with individual patient data network meta-analysis

Journal:	*BMJ Open*
Manuscript ID	bmjopen-2021-053012.R1
Article Type:	Original research
Date Submitted by the Author:	08-Sep-2021
Complete List of Authors:	Veroniki, Areti; St. Michael's Hospital, Knowledge Translation Program; Imperial College London, Department of Surgery & Cancer, Faculty of Medicine<br>Ashoor, Huda; St Michael's Hospital, Knowledge Translation Program<br>Rios, Patricia; St Michael's Hospital, Knowledge Translation Program<br>Seitidis, Georgios; University of Ioannina, Department of Primary Education<br>Stewart, Lesley; University of York, Centre for Reviews and Dissemination<br>Clarke, Mike; Queen's University Belfast, Northern Ireland Hub for Trials Methodology Research<br>Tudur-Smith, Catrin; University of Liverpool, Department of Biostatistics<br>Mavridis, Dimitris ; University of Ioannina, Department of Primary Education<br>Hemmelgarn, Brenda; University of Alberta, Department of Medicine<br>Holroyd-Leduc, Jayna; University of Calgary, Department of Medicine<br>Straus, Sharon; St Michael's Hospital, Knowledge Translation Program; University of Toronto, Department of Geriatric Medicine<br>Tricco, Andrea; St Michael's Hospital, Knowledge Translation Program; University of Toronto, Dalla Lana School of Public Health
**Primary Subject Heading**:	Public health
Secondary Subject Heading:	Neurology
Keywords:	STATISTICS & RESEARCH METHODS, EPIDEMIOLOGY, Dementia < NEUROLOGY

SCHOLARONE™
Manuscripts

For peer review only - http://bmjopen.bmj.com/site/about/guidelines.xhtml

I, the Submitting Author has the right to grant and does grant on behalf of all authors of the Work (as defined in the below author licence), an exclusive licence and/or a non-exclusive licence for contributions from authors who are: i) UK Crown employees; ii) where BMJ has agreed a CC-BY licence shall apply, and/or iii) in accordance with the terms applicable for US Federal Government officers or employees acting as part of their official duties; on a worldwide, perpetual, irrevocable, royalty-free basis to BMJ Publishing Group Ltd ("BMJ") its licensees and where the relevant Journal is co-owned by BMJ to the co-owners of the Journal, to publish the Work in this journal and any other BMJ products and to exploit all rights, as set out in our licence.

The Submitting Author accepts and understands that any supply made under these terms is made by BMJ to the Submitting Author unless you are acting as an employee on behalf of your employer or a postgraduate student of an affiliated institution which is paying any applicable article publishing charge ("APC") for Open Access articles. Where the Submitting Author wishes to make the Work available on an Open Access basis (and intends to pay the relevant APC), the terms of reuse of such Open Access shall be governed by a Creative Commons licence – details of these licences and which Creative Commons licence will apply to this Work are set out in our licence referred to above.

Other than as permitted in any relevant BMJ Author's Self Archiving Policies, I confirm this Work has not been accepted for publication elsewhere, is not being considered for publication elsewhere and does not duplicate material already published. I confirm all authors consent to publication of this Work and authorise the granting of this licence.

For peer review only

Comparative safety and efficacy of cognitive enhancers for Alzheimer's dementia: A systematic review with individual patient data network meta-analysis

Areti Angeliki Veroniki[1,2*] PhD e-mail: averoniki@uoi.gr

Huda M. Ashoor[1] BSc e-mail: huda.ashoor@unityhealth.to

Patricia Rios[1] MSc e-mail: patricia.rios@unityhealth.to

Georgios Seitidis[3] MSc e-mail: g.seitidis@uoi.gr

Lesley A. Stewart[4] PhD e-mail: lesley.stewart@york.ac.uk

Mike Clarke[5] PhD e-mail: m.clarke@qub.ac.uk

Catrin Tudur Smith[6] PhD e-mail: cat1@liverpool.ac.uk

Dimitris Mavridis[3] PhD e-mail: dmavridi@uoi.gr

Brenda R. Hemmelgarn[7] PhD e-mail: brenda.hemmelgarn@albertahealthservices.ca

Jayna Holroyd-Leduc[8] MD e-mail: jayna.holroyd-leduc@albertahealthservices.ca

Sharon E. Straus[1,9] MD e-mail: sharon.straus@utoronto.ca

Andrea C. Tricco[1,10] PhD e-mail: andrea.tricco@unityhealth.to

[1] Knowledge Translation Program, Li Ka Shing Knowledge Institute, St. Michael's Hospital, Toronto, Ontario, Canada

[2] Institute of Reproductive and Developmental Biology, Department of Surgery & Cancer, Faculty of Medicine, Imperial College, London, United Kingdom

[3] Department of Primary Education, School of Education, University of Ioannina, Ioannina, Greece

[4] Centre for Reviews and Dissemination, University of York, York, United Kingdom

[5] Northern Ireland Hub for Trials Methodology Research, Queen's University Belfast, Belfast, United Kingdom

[6] Department of Biostatistics, University of Liverpool, Block F, Waterhouse Building, 1-5 Brownlow Hill, Liverpool, L69 3GL, UK

[7] Department of Medicine, University of Alberta, Edmonton, Alberta, Canada

[8] Department of Medicine, University of Calgary, Calgary, Alberta, Canada

[9] Department of Geriatric Medicine, University of Toronto, Toronto, Ontario, Canada

For peer review only

For peer review only - http://bmjopen.bmj.com/site/about/guidelines.xhtml

[10] Epidemiology Division & Institute of Health Policy, Management, and Evaluation, Dalla Lana School of Public Health, University of Toronto, Toronto, Ontario, Canada

***Corresponding Author**:

Dr. Areti Angeliki Veroniki, MSc, PhD

209 Victoria Street, East Building, Toronto, Ontario

M5B 1T8, Canada

Phone: 416-564-5015; Fax: 416-564-5735; Email: averoniki@uoi.gr

Word count: 4,076 (max 4000); 1 table; 4 figures; 2 additional files (21 appendices in additional file 1); 31 references

For peer review only

For peer review only - http://bmjopen.bmj.com/site/about/guidelines.xhtml

Abstract

Words: 367 (Max 300 words)

Objective: To examine the comparative efficacy and safety of cognitive enhancers by patient characteristics for managing Alzheimer's Dementia (AD).

Design: Systematic review and individual patient data (IPD) network meta-analysis (NMA)

Data Sources: MEDLINE, EMBASE, Cochrane Methodology Register, CINAHL, Ageline and Cochrane Central Register of Controlled Trials up to March 2016.

Participants: 80 randomized controlled trials (RCTs) including 21,138 adults with AD, and 12 RCTs with IPD including 6,906 patients.

Interventions: Cognitive enhancers (donepezil, rivastigmine, galantamine and memantine) alone or in any combination against other cognitive enhancers or placebo.

Data extraction and Synthesis: We requested IPD from authors, sponsors and data sharing platforms. When IPD were not available, we used aggregate data. We appraised study quality with the Cochrane risk-of-bias. We conducted a two-stage random-effects IPD-NMA, and assessed their findings using CINeMA (Confidence in Network meta-analysis).

Primary and Secondary Outcomes: We included trials assessing cognition with the Mini-Mental State Examination (MMSE), and serious adverse events (SAEs).

Results: Our IPD-NMA compared 9 treatments (including placebo). Donepezil (mean difference [MD] = 1.41, 95% confidence interval [CI]: 0.51 to 2.32) and donepezil+memantine (MD = 2.57, 95% CI: 0.07 to 5.07) improved MMSE score (56 RCTs, 11,619 participants; CINeMA score: moderate) compared with placebo. According to P-score, oral rivastigmine (odds ratio [OR] = 1.26, 95% CI: 0.82 to 1.94, P-score= 16%) and donepezil (OR = 1.08, 95% CI: 0.87 to 1.35, P-score= 30%) had the least favourable safety profile, but none of the estimated treatment effects were sufficiently precise when compared with placebo (45 RCTs, 15,649 patients; CINeMA score: moderate to high). For moderate to severe impairment, donepezil, memantine and their combination performed best, but for mild to moderate impairment donepezil and transdermal rivastigmine ranked best. Adjusting for MMSE baseline differences, oral rivastigmine and galantamine

For peer review only

For peer review only - http://bmjopen.bmj.com/site/about/guidelines.xhtml

improved MMSE score, whereas when adjusting for comorbidities only oral rivastigmine was effective.

Conclusions: The choice among the different cognitive enhancers may depend on patient's characteristics. All cognitive enhancer regiments except for single-agent oral rivastigmine, galantamine, and memantine, were clinically important for cognition (MMSE score greater than 1.4). However, two thirds of the published RCTs were associated with high risk of bias for incomplete outcome data, and IPD were only available for 15% of the included RCTs.

Registration: PROSPERO # CRD42015023507

Funding: This research was funded by the CIHR Drug Safety and Effectiveness Network (grant number 137713).

Keywords: network meta-analysis; multiple treatments meta-analysis; individual participant data; Nootropic Agents; Alzheimer Disease

Strengths and limitations of this study

- This is one of the most comprehensive systematic reviews and network meta-analysis of cognitive enhancers including individual patient data for Alzheimer's Dementia to produce treatment recommendations by patient characteristics.
- We followed the methodologically rigorous guidelines in the Cochrane Handbook for systematic reviews, and the CINeMA quality assessment guidelines.
- Access to individual patient data allowed us to 1) observe minor differences between the original published results and our re-analysis, potentially due to differences in imputation methods for missing data or because original studies have excluded some patients, and hence have used a smaller sample size, 2) overcome potential reporting bias, and 3) assess for potential effect modifiers that were not reported in the original publications (e.g., comorbidities, additional medications) and explore for treatment-by-covariate interactions on the patient-level.
- Two thirds of the included RCTs, were associated with high risk of bias for incomplete outcome data due to attrition.
- We were unable to include individual patient data for all RCTs (only 15% of the studies shared their individual patient data), highlighting potential availability bias.

For peer review only

For peer review only - http://bmjopen.bmj.com/site/about/guidelines.xhtml

Introduction

Alzheimer's dementia (AD) is the most common type of dementia.[1] Patients living with AD have a lower quality of life due to deterioration in function, cognition, behavior, and mental health over time, as well as increased mortality.[2] Pharmacological treatment for AD predominantly consists of cholinesterase inhibitors (donepezil, galantamine, rivastigmine) and the N-methyl-d-aspartate (NMDA) receptor antagonist, memantine. All three cholinesterase inhibitors and memantine are currently the only effective licensed treatments for dementia,[3] but their clinical effect can be small and there is no convincing evidence that they modify the disease process in AD.[4] Also, it is unclear whether galantamine, rivastigmine, or donepezil should be used by patients with severe AD, or whether memantine is the optimal treatment for severe AD.[5]

In AD, disease severity and sex are potential effect modifiers. However, aggregate data and covariates of interest (e.g., sex, disease severity) are not consistently reported across randomized clinical trials (RCTs).[6] The use of IPD has several advantages, such as it allows for the exploration of the relationship between treatment effects and patient-level characteristics, and it overcomes restrictions in using the information reported in the publication among others. The aim of this study was to examine the comparative efficacy and safety of cognitive enhancers for patients with different characteristics, such as severities of AD and for females versus males through a systematic review and individual patient data (IPD) NMA. NMA is an extension of standard meta-analysis synthesizing different sources of evidence from a network of RCTs comparing different treatments within a single model. NMA can provide treatment effect estimates for treatment comparisons that have not studied in a head-to-head study.

Methods

We reported our results according to the Preferred Items for Systematic Reviews and Meta-analysis (PRISMA) Statement for NMA and PRISMA-IPD.[7,8]

Protocol

For peer review only - http://bmjopen.bmj.com/site/about/guidelines.xhtml

The research question and protocol were based on our previous systematic review and NMA.[6] We registered our systematic review protocol with the prospective register of systematic reviews (PROSPERO: CRD42015023507), and published our protocol.[9] Additional information is also provided in Appendix 1 and Additional File 2. Herein, we briefly summarize our methods.

Eligibility criteria

We updated our previous systematic review,[6] using similar population, interventions, comparators, study designs and time period (PICOST) criteria. The literature search was updated from January 2015 to March 2016. We included published and English RCTs that assessed cognition via the Mini-Mental State Examination (MMSE; efficacy and primary outcome) and/or serious adverse events (SAE; safety outcome) in adults with Alzheimer's dementia.

IPD collection process

We contacted the corresponding author followed by the next-in-order author, as presented in each eligible RCT, to obtain IPD. The author contact process was part of a RCT that our team conducted to assess methods that may optimize response rates for IPD retrieval.[10] We also contacted sponsors of eligible trials, as reported in the publications. We contacted industry sponsors only, as we were not able to locate contact information for the majority of non-industry sponsors (e.g., grants and university funding). If a study had multiple sponsors, we contacted all of them. To further facilitate IPD access, we contacted the Clinical Study Data Request (CSDR)[11] and Yale University Open Data Access (YODA) data sharing platforms.[12] If a data provider was unable to provide IPD we noted the reason.

Risk of bias and quality appraisal

We appraised study quality using the Cochrane risk of bias tool.[13] To ensure data consistency[8] we compared IPD with aggregate data reported in the publication. We assessed whether

For peer review only - http://bmjopen.bmj.com/site/about/guidelines.xhtml

randomization of patients was adequate (i.e., intervention and comparison groups were balanced for important patient characteristics), by comparing numbers and types of patients in each arm.

When at least 10 studies were available for each treatment against placebo, publication bias and small-study effects were examined visually using the comparison adjusted funnel plot under the fixed-effect model.[3] When a funnel plot asymmetry was detected, we performed the Copas selection for the treatment comparisons that were informed by at least 10 studies and for which asymmetry was evident in the funnel plot. We explored the possibility that this was due to publication bias,[14] and made moderate assumptions about the probability of publication of the smaller and larger (in terms of standard error) studies. We assumed that the smallest study had a probability of publication equal to 40-50% and the largest study had a probability of 80-90%. Confidence in NMA findings was assessed for each outcome using CINeMA (Confidence in Network meta-analysis, see Appendix 1 for more details).[15]

Synthesis

We performed a descriptive analysis using frequencies and distributions of the characteristics of the included patients and treatments. For each outcome, we present the network geometry according to IPD availability. We conducted a two-stage IPD analysis, whereby data were analysed separately in each trial in the first stage and the trial parameter estimates were synthesised in a random-effects meta-analysis or NMA in the second stage.

The summary treatment effects are presented using the odds ratio (OR) or mean difference (MD) along with their corresponding CIs and predictive intervals (PIs).[16] We ranked the interventions for each outcome using the P-scores (and SUCRAs [surface under the cumulative ranking curve] in meta-regression analysis), and present them in a rank-heat plot.[17,18]

Patient and public involvement

Not applicable.

For peer review only - http://bmjopen.bmj.com/site/about/guidelines.xhtml

Results

Literature search, study selection and IPD obtained

After screening 20,410 titles and abstracts and 1,968 full-text articles, 96 studies fulfilled the eligibility criteria; 80 unique studies and 16 companion reports (Figure 1a, Appendix 2).

(Figure 1 here)

Of the 80 RCTs, 55 reported at least one industry-sponsored funder (i.e. 40 studies reported a single industry-sponsor and 15 multiple industry-sponsors). In the remaining studies, 9 were publicly-sponsored and 16 did not report any information about funding. We requested IPD by contacting the corresponding authors for 80 RCTs that included 21,138 participants. None of the original authors shared their IPD. Fifteen commercial sponsors were then contacted and 6 (40%) sponsors shared their data through proprietary sponsor-specific platforms. The 6 sponsors were contacted for 46 RCTs (14,580 participants), and we obtained IPD for 30% (14 RCTs, 8,007 participants) of these RCTs (1,058 total waiting days up to March 9, 2020). The study flow for obtaining IPD is depicted in Figure 1b.

We were able to include 12 (6,906 patients) of 14 RCTs in our NMA due to incompleteness of provided IPD (Appendix 3). The number of studies with available/non-available IPD from each data provider along with reasons for non-availability of IPD are presented in Appendix 4.

Study and patient characteristics

Most included studies (33%) were multi-national. The mean age of patients ranged from 61 to 86 years. The majority of the RCTs included patients with mild-moderate AD (56%), although the diagnostic criteria used for AD varied widely (Table 1). The most frequent longest duration of follow-up was 24 weeks (24 RCTs, 30%; Appendix 5). Important patient characteristics, such as percent of male and dropout rates, were not balanced across groups in the RCTs with provided IPD (Appendix 6). Comparing study and patient characteristics of available and non-available

For peer review only - http://bmjopen.bmj.com/site/about/guidelines.xhtml

IPD when a study was industry-sponsored, we found differences in the year of study publication, study size, and absolute mean difference (Appendix 7).

(Table 1 here)

Risk of bias and IPD integrity

Using the Cochrane risk-of-bias tool, allocation concealment was at low risk of bias for 43% and blinding of participants and personnel was low for 64% of the RCTs (Appendix 8). One third of the RCTs had low risk of incomplete outcome data bias due to attrition and almost two thirds had high potential risk of "other" bias, specifically, funding bias. The other risk of bias item was scored as unclear for 32%. Overall risk of bias was comparable in studies with available and unavailable IPD (Appendix 9).

All IPD provided were checked for consistency and results from published RCTs were reproduced and provided in Appendix 10. High dropout rates were observed in the IPD; experiencing an adverse event was the most common reason for dropout. Despite the high dropout rates observed in the individual studies, there was no indication of correlation between age and dropout (Appendix 11). Comparison-adjusted funnel plot for MMSE suggested there is indication for small-study effects (see Appendix 12). In contrast to the standard meta-analysis (MD=1·65 95% CI (0·16, 3·14)), the Copas selection model estimated a pooled treatment effect for donepezil vs. placebo MD=1·87 95% CI (1·55, 2·20) with between-study variance τ^2= 1·95, and correlation coefficient -0·45 (-0·76, -0·01) reflecting the belief that the propensity for publication was associated with the observed effect size.

Network meta-analysis

In both MMSE and SAE outcomes, on average there were no important concerns regarding the transitivity and consistency assumptions (Appendices 13 and 14; design-by-treatment interaction model MMSE: χ^2= 4·36, 13 degrees of freedom (df), P= 0·987; SAE: χ^2= 3·57, 6 df, P= 0·735).

For peer review only - http://bmjopen.bmj.com/site/about/guidelines.xhtml

Below we present the main analysis results compared to placebo. Additional analyses are presented in Appendix 15-16). The network geometry is presented in Figure 2.

(Figure 2 here)

Cognition

The NMA for MMSE included 56 RCTs, 9 treatments (including placebo), and 11,619 participants. Nine RCTs (3,625 patients) contributed IPD and 47 RCTs (7,994 patients) contributed aggregated data to the NMA. Two studies[19,20] did not report MMSE in the final publication, but in the retrieved IPD we were able to use data for this outcome.

NMA of studies with IPD and aggregate data

Studies in this NMA compared all available treatments. Donepezil (MD= 1·41, 95% CI: 0·51 to 2·32) and donepezil+memantine (MD= 2·57, 95% CI: 0·07 to 5·07) were superior to placebo in terms of MMSE score (Appendix 15). PIs suggested results are not conclusive. Transdermal rivastigmine (MD= 2·11, 95% CI: -0·04 to 4·26), and the combinations donepezil+memantine, galantamine+memantine (MD= 2·24, 95% CI: -2·13 to 6·61), and transdermal rivastigmine+memantine (MD= 1·79, 95% CI: -1·70 to 5·27) were associated with a minimal clinically important difference (MCID; above 1·40)[21] (Figure 3a). However, donepezil+memantine had the highest likelihood of being the most effective in improving MMSE score (P-score range 79-80%, Figure 4). Confidence in NMA results was moderate (Appendix 17).

(Figure 3 here)

(Figure 4 here)

NMA of studies with aggregate data

Studies in this NMA compared all available treatments. Donepezil improved MMSE score significantly (MD= 1·55 95% CI: 0·41 to 2·68). The MCID results were in agreement with the

For peer review only - http://bmjopen.bmj.com/site/about/guidelines.xhtml

NMA of IPD and aggregate data, and donepezil+memantine (MD= 2·71, 95% CI: -0·17 to 5·60) was likely the most effective in improving MMSE score (P-score= 76%).

NMA of studies with IPD

Studies in this NMA compared placebo, donepezil, oral rivastigmine, transdermal rivastigmine, galantamine, and memantine. Donepezil (MD= 0·70, 95% CI: 0·01 to 1·40) and transdermal rivastigmine (MD= 1·06, 95% CI: 0·04 to 2·08) were superior to placebo, but none was at a MCID. The most effective treatment was likely transdermal rivastigmine (P-score= 82%).

Additional analyses using IPD and aggregate data

Overall, additional analyses using both IPD and aggregate data were in agreement with the findings of the main analysis (Appendix 16). Cognitive performance was better in patients with mild to moderate MMSE receiving donepezil (MD= 1·68 95% CI: 0·31 to 3·06, P-score= 69%) and most likely when receiving transdermal rivastigmine (MD= 2·74 95% CI: -0·68 to 6·16, P-score= 81%). In patients with moderate to severe MMSE the combination donepezil+memantine improved MMSE score significantly (MD= 2·49 95% CI: 1·55 to 3·44, P-score=100%), but oral rivastigmine deteriorated MMSE score significantly (MD= -1·00 95% CI: -1·87 to -0·12, P-score= 4%). Donepezil (MD= 1·31 95% CI: 0·66 to 1·96, P-score= 78%) and memantine (MD=0·69 95% CI: 0·07 to 1·31, P-score= 59%) also performed well for patients with moderate to severe cognitive impairment. However, PIs are wide suggesting results are not conclusive.

Accounting for the impact of the outlier studies, galantamine+memantine was the second-best cognitive enhancer (MD= 1·87 95% CI: 0·08 to 3·66, P-score=82%) after donepezil+memantine (MD= 2·04 95% CI: 1·03 to 3·05, P-score= 92%). Using only IPD adjusted for comorbidities suggested that oral rivastigmine improves MMSE score, but results are inconclusive as indicated in the prediction interval (MD= 0·88 95% CI: 0·31 to 1·45, 95% PI: -0·05 to 1·81, P-score= 75%). Similarly, using IPD adjusted for cognitive impairment assessed with MMSE at baseline suggested that oral rivastigmine (MD= 0·88 95% CI: 0·31 to 1·45, P-score= 69%) and galantamine (MD= 0·76 95% CI: 0·34 to 1·18, P-score= 62%) improve MMSE score, but in a future study, results are only stable for galantamine.

For peer review only - http://bmjopen.bmj.com/site/about/guidelines.xhtml

For peer review only

Heterogeneity in NMA was high (between-study variance = 5·75, I^2= 96%) compared also to the Rhodes et *al.*[22] empirical distribution (median 0.05, 95% range: 0·00 to 7·56). However, heterogeneity decreased importantly when excluding outliers (between-study variance = 0·59, I^2= 73%), when including only patients with moderate to severe AD (between-study variance = 0·18, I2= 44%), restricting to industry-sponsored trials (between-study variance = 0·16, I^2= 43%), and when using IPD only (between-study variance = 0·12, I^2= 29%).

Serious adverse events

A NMA was conducted on serious adverse events (study definitions are provided in Appendix 19) with 45 RCTs, 9 treatments (including placebo), and 15,649 patients (Figure 2b). In particular, 12 RCTs (6420 patients) contributed to the NMA using their IPD and 33 RCTs (9229 patients) using their data on their aggregated form. The time taken to achieve at least one SAE was available in 8 studies with available IPD and ranged between 45 and 2228 days (Appendix 20). Only one study included a patient with a SAE occurring earlier than the trial opening and was excluded from the study.[23]

NMA of studies with IPD and aggregate data

Studies in this NMA compared all available treatments. According to P-score, oral rivastigmine had the least favourable safety profile regarding SAE (OR= 1·26, 95% CI: 0·82 to 1·94, P-score= 16%), followed by donepezil (OR= 1·08, 95% CI: 0·87 to 1·35, P-score= 30%) and galantamine+memantine (OR= 1·03, 95% CI: 0·45 to 2·39, P-score= 43%), yet none of these comparisons were statistically significant different from placebo (Figure 3b; Appendices 16, 18). Confidence in NMA results ranged between moderate and high (Appendix 17).

NMA of studies with aggregate data

Studies in this NMA compared all available treatments. Results were mainly consistent with NMA of IPD and aggregate data, but for memantine which was statistically significantly

For peer review only - http://bmjopen.bmj.com/site/about/guidelines.xhtml

associated with lower odds of a SAE than placebo when using aggregate data only (OR 0·70, 95% CI: 0·51 to 0·97, P-score= 77%,).

NMA of studies with IPD

Studies in this NMA compared placebo, donepezil, oral rivastigmine, transdermal rivastigmine, galantamine, and memantine. Results were on average consistent with NMA of IPD and aggregate data.

Additional analyses using IPD and aggregate data

Additional analyses using both IPD and aggregate data, showed that memantine was statistically significantly associated with lower odds of a SAE than placebo when using study duration as a covariate (OR= 0·61, 95% CI: 0·37 to 0·93, P-score= 88%). Restricting to low risk of bias for incomplete outcome data, galantamine was associated with significantly lower odds of a SAE (OR= 0·69, 95% CI: 0·50 to 0·97, P-score= 80%).

Heterogeneity in NMA was low (between-study variance = 0·04, I^2= 22%) compared to the Turner et *al.*[24] empirical distribution (median 0·12, 95% range: 0·01 to 2·63). Heterogeneity decreased importantly when restricting to aggregate data (between-study variance = 0·00, I^2= 0%), low risk of bias for incomplete outcome data (between-study variance = 0·02, I^2= 10%), patients with moderate to severe cognitive impairment (between-study variance = 0·00, I^2= 0%), and when adjusting for study duration (between-study variance = 0·03), year of publication (between-study variance = 0·02), mean age (between-study variance = 0.02) or sex (between-study variance = 0·03).

Discussion

We compared the efficacy and safety of cognitive enhancers regarding MMSE and SAE outcomes to update our previous systematic review[6] and included studies with both aggregate data and IPD. Our results are in agreement with our previous systematic review,[6] and show that donepezil+memantine, donepezil alone and transdermal rivastigmine were the most effective

For peer review only - http://bmjopen.bmj.com/site/about/guidelines.xhtml

treatments for improving MMSE score. However, heterogeneity was a major concern, and this was also captured by PIs. According to the P-score intervention ranking, both donepezil+memantine and transdermal rivastigmine had a favourable safety profile regarding SAE, whereas the therapy with the least favourable profile was oral rivastigmine followed by donepezil. However, none of the estimated treatment effects were sufficiently precise when cognitive enhancers were compared with the placebo group. CINeMA suggested that within-study bias and reporting bias were the highest concerns for the MMSE outcome, whereas within-study bias and imprecision of effect estimates were the highest concerns for the SAE outcome.

Overall, the choice among the different cognitive enhancers may depend on the patient's characteristics. In participants with moderate to severe cognitive impairment (defined by MMSE), a larger improvement in cognitive performance was observed for donepezil and memantine, and their combination (donepezil+memantine), and these efficacy-related results are expected to also be reflected when a future study becomes available. The least effective cognitive enhancer in participants with moderate to severe cognitive impairment was oral rivastigmine. For patients with mild to moderate impairments based on MMSE scores, donepezil and transdermal rivastigmine were most likely the best performing cognitive enhancers. For patients with moderate to severe cognitive impairment, cognitive enhancers were well tolerated. For patients with mild to moderate cognitive impairment, all except for memantine and its combination with transdermal rivastigmine, were associated with increased odds of a SAE, yet none of these results reached statistical significance. Overall, memantine was associated with lower odds of a SAE than placebo, yet this was statistically significant only in the subnetwork analysis including aggregate data (i.e., studies without IPD) and the meta-regression analysis using study duration as a covariate. However, acknowledging for heterogeneity in the network, prediction intervals suggested that results are inconclusive and the odds of SAE could not be differentiated between memantine and placebo. Of note, the accuracy of SAE reporting may be impacted by the degree of cognitive impairment. Using IPD only and adjusting for MMSE baseline differences, (as shown in Appendix 16, Mean Difference: NMA of studies with IPD adjusted for baseline cognitive impairment), oral rivastigmine and galantamine improved MMSE score, whereas when adjusting for comorbidities only oral rivastigmine was effective, but results can change in a future study. Considering a MCID equal to 1·4,[21] all cognitive enhancer

For peer review only - http://bmjopen.bmj.com/site/about/guidelines.xhtml

regiments except for single-agent oral rivastigmine, galantamine, and memantine, were clinically important for cognition. Our results did not differ by participant characteristics sex, age, and other medications, or by study characteristics, study duration and year of publication. However, these findings might be due to low power since meta-regression analyses depend on the number and size of studies, magnitude of the relationship between the covariate and effect size, along with its precision and heterogeneity.[25]

To the best of our knowledge, our study was the first to add IPD in a NMA of cognitive enhancers for patients with Alzheimer's Dementia to produce treatment recommendations by patient characteristics. We followed the methods guidelines in the Cochrane Handbook for systematic reviews,[26] the reporting guidelines in the PRISMA-NMA and PRISMA-IPD statements,[7,8] and the CINeMA quality assessment guidelines.[15] Compared to previous systematic reviews, we included a larger number of studies and/or studies with shared IPD, compared in a wider range of cognitive enhancers.[6,27] Our results are in agreement with previous studies overall. Access to IPD allowed us to observe minor differences between the original published results and our re-analysis. An explanation in these differences may be that many studies used the last-observation-carried-forward imputation method, whereas we used the available case analysis when assessing MMSE. Another potential explanation might be that original studies excluded some patients, and hence used a smaller sample size.

Comparing NMA, results between aggregate data and IPD were in agreement. The only difference was observed in transdermal rivastigmine that was associated with a MCID of MMSE in the aggregate data NMA compared to the IPD NMA, yet a statistically significant improvement was achieved in the IPD NMA. The inclusion of IPD in our NMA, allowed us to overcome potential reporting bias and to include IPD for 1) a study that we previously were unable to include since arm-level data were not reported in the RCT publication,[23] and 2) two studies that did not report MMSE results in their publications.[19,20] The use of IPD also allowed us to assess for potential effect modifiers that were not reported in the original publications (e.g., comorbidities, additional medications) and explore for treatment-by-covariate interactions on the patient-level. Several challenges were encountered during the IPD request from sponsors, showing that repositories are not a panacea (Appendix 21).

For peer review only - http://bmjopen.bmj.com/site/about/guidelines.xhtml

An important finding of our review is that the two thirds of the published RCTs, were associated with high risk of bias for incomplete outcome data due to attrition, and the majority of these RCTs used the last-observation-carried-forward technique for missing data. This approach may bias results favouring cognitive enhancers, since the dropout rates were greater in the treatment group compared to the placebo group in 63% of the included studies and because dementia is a progressive disease. Of the 27 studies comparing treatment against placebo and reporting the number of dropouts, 17 studies had a greater dropout rate in the treatment group (treatment group: median dropout rate= 28% IQR [17% to 39%]; placebo group: median dropout rate= 21% IQR [15% to 31%]). Last-observation-carried-forward is an inappropriate imputation method for Alzheimer's Dementia studies, since it ignores expected deterioration of the patient's condition and stabilizes the outcome at the value observed at the time of dropout (i.e., the last observation).[28] Restricting to low risk of attrition bias studies, we found that galantamine was significantly associated with decreased odds of experiencing a SAE.

Our study has limitations worth mentioning. First, we were unable to include IPD for all eligible studies (only 15% of the included RCTs shared their IPD), highlighting potential availability bias for IPD. However, recent simulations have shown that combining IPD and aggregate data in a NMA can significantly improve precision, reduce bias, and increase information compared to NMA relying on aggregated data alone.[29] Second, missing data is a big concern in the published RCTs for Alzheimer's Dementia. To assess the impact of missing data in our NMA, we applied the informative missingness of difference in means.[30] Third, the lack of studies in certain treatment comparisons may have affected the P-score calculation and treatment ranking. In particular, polytherapies were informed by maximum two studies, and ranking may have been in favour of the complex intervention group with the smaller number of studies.[31] For example, in MMSE the polytherapies including memantine in conjunction with one of the three treatments donepezil, galantamine, transdermal rivastigmine had a P-score ≥60%, but these all had wide 95% CIs for MD. As such, ranking should be interpreted with caution and along with the estimated effect sizes and their uncertainty measures. Fourth, the comparison-adjusted funnel plot for MMSE suggested there is an indication for small-study effects pointing to the treatment being better, and results should be interpreted with caution. Overall, MMSE score is only a

For peer review only - http://bmjopen.bmj.com/site/about/guidelines.xhtml

surrogate maker for determining the impact of treatments on dementia. A full assessment that considers the potential impact of treatments on cognition, function and behavioural symptoms needs to be considered within the clinical context. Fifth, differences in patient characteristics, such as sex, were observed in the RCTs with provided IPD, which increased heterogeneity across studies. To account for these differences, we used the fully adjusted treatment effect estimates in the IPD analyses and the primary NMA analysis. Also, at the NMA level, we found that on average there were no important differences across treatment comparisons to threaten the transitivity assumption. Sixth, there are clinically important limitations associated with this review, including consistent definition of outcome measures across studies, a well-established MCID for the MMSE score, lack of consideration of drug doses due to inconsistent reporting and data availability bias that we were unable to overcome (15% of the studies shared their IPD). Future studies are needed to establish ranking efficacy in drug doses and combination of interventions across different disease severity categories. Seventh, the literature searches were conducted 5 years ago and additional relevant studies may be available. However, obtaining IPD in a timely manner was very challenging and required more time than anticipated (challenges to obtain IPD are outlined in Appendix 21). Similar to all systematic reviews, the evidence should be regularly updated.

We expect that our findings will increase scientific knowledge, because people with Alzheimer's Dementia require personalized medicine to optimize their healthcare. Well-conducted meta-analyses of IPD are considered the 'gold-standard' and influence patient care since patient-level data can be provided to facilitate tailored decision making. However, results from meta-analyses of IPD are likely subject to retrieval bias and awareness of these limitations and their potential impact on findings is required.

For peer review only

For peer review only - http://bmjopen.bmj.com/site/about/guidelines.xhtml

Contributors

AAV, SES and ACT conceived and designed the study. AAV conducted the analyses, abstracted data, contacted sponsors, analysed data, interpreted results, appraised quality of results, and wrote a draft manuscript. GS conducted the analyses, appraised quality of results, and edited the manuscript. HMA coordinated the review, screened citations and full-text articles, abstracted data, appraised quality, cleaned the data, contacted sponsors, and edited the manuscript. PR helped coordinate the study, screened citations and full-text articles, extracted and categorized data, appraised quality, and edited the manuscript. SES and ACT interpreted results and edited the manuscript. ACT and HMA contacted authors. LAS, MC, CTS, DM, BRH, JHL provided input into the design, interpreted results, and edited the manuscript. All authors read and approved the final manuscript.

Declaration of interests

The authors declare that they have no competing interests.

Data sharing statement

All data relevant to the study are included in the article or uploaded as supplementary information.

Funding

This research was funded by the CIHR Drug Safety and Effectiveness Network (grant number 137713). The funder contributed to defining the scope of the systematic review but otherwise had no role in study design, data collection, analysis, and interpretation of data. Data sharing sponsors were provided the ability to review at the time of submission of the manuscript for publication.

AAV was previously funded by the Canadian Institutes of Health Research (CIHR) Banting Postdoctoral Fellowship Program [No. 139157]. GS and DM were funded from the European Union’s Horizon 2020 [No. 754936]. SES is funded by a Tier 1 Canada Research Chair in

For peer review only - http://bmjopen.bmj.com/site/about/guidelines.xhtml

Knowledge Translation [No. N/A]. ACT is funded by a Tier 2 Canada Research Chair in Knowledge Synthesis [No. N/A].

Open Access

This is an open access article distributed in accordance with the Creative Commons Attribution Non Commercial (CC BY-NC 4.0) license, which permits others to distribute, remix, adapt, build upon this work non-commercially, and license their derivative works on different terms, provided the original work is properly cited, appropriate credit is given, any changes made indicated, and the use is non-commercial. See: http:// creativecommons. org/ licenses/ by- nc/ 4. 0/.

Acknowledgements

We thank Laure Perrier for conducting the initial literature search, Alissa Epworth for updating it, and Becky Skidmore for peer reviewing the literature search. We thank Robert Peterson for his support on this study as a knowledge user. We would also like to thank Paul A. Khan, Fatemeh Yazdi, Marco Ghassemi, John D. Ivory, Charlene Soobiah, Erik Blondal, Joanne M. Ho, Shirra Berliner, and Carmen H. Ng for screening some of the citations, data abstracting included studies, or both. We thank Susan Le for helping with contacting the authors and sponsors of the included studies in this review. Finally, we thank Susan Le for formatting the manuscript and assisting with the submission.

We would also like to thank the following sponsors for sharing the data with us:

- "This publication is based on research using data from data contributors, AbbVie, Inc, that has been made available through Vivli, Inc. Vivli has not contributed to or approved, and is not in any way responsible for, the contents of this publication."
- "This study, carried out under YODA Project #2017-1671, used data obtained from the Yale University Open Data Access Project, which has an agreement with JANSSEN RESEARCH & DEVELOPMENT, L.L.C.. The interpretation and reporting of research using this data are solely

For peer review only

the responsibility of the authors and does not necessarily represent the official views of the Yale University Open Data Access Project or JANSSEN RESEARCH & DEVELOPMENT, L.L.C..”

- This publication used data obtained from Eisai, GlaxoSmithKline, and Novartis carried under www.ClinicalStudyDataRequest.com
- This publication used data obtained from Lundbeck

Ethical Approval Statement

Not applicable.

For peer review only

For peer review only - http://bmjopen.bmj.com/site/about/guidelines.xhtml

References

1. Health NCCfM. Dementia: A NICE-SCIE guideline on supporting people with dementia and their carers in health and social care. 2007: British Psychological Society; 2007.
2. Dudgeon S. Rising Tide: The Impact of Dementia on Canadian Society: a Study. 2010: Alzheimer Society of Canada; 2010.
3. O'Brien JT, Holmes C, Jones M, et al. Clinical practice with anti-dementia drugs: A revised (third) consensus statement from the British Association for Psychopharmacology. *Journal of psychopharmacology (Oxford, England)* 2017; **31**(2): 147-68.
4. National Institute for Health and Care Excellence (UK). Dementia: Assessment, management and support for people living with dementia and their carers. 2018. Available from: https://www.ncbi.nlm.nih.gov/books/NBK536484/.
5. National Institute for Health and Clinical Excellence. Donepezil, galantamine, rivastigmine and memantine for the treatment of Alzheimer's disease. London, UK, 2011.
6. Tricco AC, Ashoor HM, Soobiah C, et al. Comparative Effectiveness and Safety of Cognitive Enhancers for Treating Alzheimer's Disease: Systematic Review and Network Metaanalysis. *J Am Geriatr Soc* 2018; **66**(1): 170-8.
7. Hutton B, Salanti G, Caldwell DM, et al. The PRISMA extension statement for reporting of systematic reviews incorporating network meta-analyses of health care interventions: checklist and explanations. *Annals of internal medicine* 2015; **162**(11): 777-84.
8. Stewart LA, Clarke M, Rovers M, et al. Preferred Reporting Items for Systematic Review and Meta-Analyses of individual participant data: the PRISMA-IPD Statement. *Jama* 2015; **313**(16): 1657-65.
9. Veroniki AA, Straus SE, Ashoor HM, et al. Comparative safety and effectiveness of cognitive enhancers for Alzheimer's dementia: protocol for a systematic review and individual patient data network meta-analysis. *BMJ open* 2016; **6**(1): e010251.
10. Veroniki AA, Ashoor HM, Le SPC, et al. Retrieval of individual patient data depended on study characteristics: a randomized controlled trial. *Journal of clinical epidemiology* 2019; **113**: 176-88.
11. So D, Knoppers BM. Ethics approval in applications for open-access clinical trial data: An analysis of researcher statements to clinicalstudydatarequest.com. *PLoS One* 2017; **12**(9): e0184491.
12. Krumholz HM, Waldstreicher J. The Yale Open Data Access (YODA) Project--A Mechanism for Data Sharing. *N Engl J Med* 2016; **375**(5): 403-5.
13. Higgins JP, Altman DG, Gotzsche PC, et al. The Cochrane Collaboration's tool for assessing risk of bias in randomised trials. *Bmj* 2011; **343**: d5928.
14. Mavridis D, Sutton A, Cipriani A, Salanti G. A fully Bayesian application of the Copas selection model for publication bias extended to network meta-analysis. *Statistics in medicine* 2013; **32**(1): 51-66.
15. CINeMA: Confidence in Network Meta-Analysis. Institute of Social and Preventive Medicine, University of Bern. *Available from cinemaispmch* 2017.
16. Riley RD, Higgins JP, Deeks JJ. Interpretation of random effects meta-analyses. *Bmj* 2011; **342**: d549.
17. Veroniki AA, Straus SE, Fyraridis A, Tricco AC. The rank-heat plot is a novel way to present the results from a network meta-analysis including multiple outcomes. *Journal of clinical epidemiology* 2016; **76**: 193-9.
18. Rucker G, Schwarzer G. Ranking treatments in frequentist network meta-analysis works without resampling methods. *BMC Med Res Methodol* 2015; **15**: 58.
19. Herrmann N, Gauthier S, Boneva N, Lemming OM. A randomized, double-blind, placebo-controlled trial of memantine in a behaviorally enriched sample of patients with moderate-to-severe Alzheimer's disease. *International psychogeriatrics* 2013; **25**(6): 919-27.

For peer review only - http://bmjopen.bmj.com/site/about/guidelines.xhtml

20. Burns A, Bernabei R, Bullock R, et al. Safety and efficacy of galantamine (Reminyl) in severe Alzheimer's disease (the SERAD study): a randomised, placebo-controlled, double-blind trial. *The Lancet Neurology* 2009; **8**(1): 39-47.

21. Howard R, Phillips P, Johnson T, et al. Determining the minimum clinically important differences for outcomes in the DOMINO trial. *Int J Geriatr Psychiatry* 2011; **26**(8): 812-7.

22. Rhodes KM, Turner RM, Higgins JP. Predictive distributions were developed for the extent of heterogeneity in meta-analyses of continuous outcome data. *Journal of clinical epidemiology* 2015; **68**(1): 52-60.

23. Gold M, Alderton C, Zvartau-Hind M, et al. Rosiglitazone monotherapy in mild-to-moderate Alzheimer's disease: results from a randomized, double-blind, placebo-controlled phase III study. *Dementia and geriatric cognitive disorders* 2010; **30**(2): 131-46.

24. Turner RM, Davey J, Clarke MJ, Thompson SG, Higgins JP. Predicting the extent of heterogeneity in meta-analysis, using empirical data from the Cochrane Database of Systematic Reviews. *International journal of epidemiology* 2012; **41**(3): 818-27.

25. Hedges L, Higgins J, Rothstein H. Introduction to Meta-Analysis. John Wiley & Sons; 2009.

26. Higgins JP, Thomas J, Chandler J, et al. Cochrane handbook for systematic reviews of interventions: John Wiley & Sons; 2019.

27. Cui CC, Sun Y, Wang XY, Zhang Y, Xing Y. The effect of anti-dementia drugs on Alzheimer disease-induced cognitive impairment: A network meta-analysis. *Medicine* 2019; **98**(27): e16091.

28. Molnar FJ, Hutton B, Fergusson D. Does analysis using "last observation carried forward" introduce bias in dementia research? *CMAJ* 2008; **179**(8): 751-3.

29. Jansen JP. Network meta-analysis of individual and aggregate level data. *Res Synth Methods* 2012; **3**(2): 14.

30. Mavridis D, White IR. Dealing with missing outcome data in meta-analysis. *Res Synth Methods* 2020; **11**(1): 2-13.

31. Kibret T, Richer D, Beyene J. Bias in identification of the best treatment in a Bayesian network meta-analysis for binary outcome: a simulation study. *Clinical epidemiology* 2014; **6**: 451-60.

For peer review only

Figure Captions

Figure 1. Flow diagram for study inclusion in the review (a) and studies retrieved with individual patient data (b).

Figure 2. Network diagrams for (a) MMSE and (b) SAE outcomes. The size of each node and line indicates the number of studies included in each treatment comparison. The number of studies per treatment comparison is presented on each edge, and the number of studies with individual patient data (IPD) is depicted in a parenthesis. Orange coloured edges are informed by both IPD and aggregate data, whereas black coloured edges are informed by aggregate data only.

Figure 3. Forest plot of network meta-analysis (NMA) results for all cognitive enhancers versus placebo in (a) MMSE outcome, and (b) SAE outcome. NMA results are presented for i) aggregate data (AD) and fully adjusted results from studies with available individual patient data (IPD), ii) AD and crude results from studies with available IPD, iii) AD only (studies with available IPD are not included in the analysis), and iv) crude results from individual studies with individual patient data (IPD).

Figure 4. Rank-heat plot of P-scores for 9 treatments, including placebo, studied in randomized clinical trials with patients with Alzheimer's Dementia assessing MMSE. Circles from inside out present results for different network meta-analyses including: i) aggregate data (AD) only (studies with available IPD are not included in the analysis), ii) crude results from individual studies with individual patient data (IPD), iii) AD and crude results from studies with available IPD, and iv) AD and fully adjusted results from studies with available IPD. Numbers within each sector correspond to the P-score values as calculated in each model.

For peer review only

Tables

Table 1· Study and patient characteristics

	AD (N=80)	IPD (N=12)
**Total # participants**	21,138	5839
**Longest duration of follow-up in weeks: mean (range)**	28·28 (8 - 208)	29·33 (12 - 104)
**Mean number of patients (range)**	264·23 (14 - 2,045)	486·58 (123 - 2,045)
**Mean age in years (range)**	74·64 (61 - 85·7)	73·94 (70·4 - 78)
**Mean % Female (range)**	61·35 (3 - 89)	62·76 (53·68 - 81)
**Country of conduct: frequency (%)**		
Canada	2 (2·50)	1 (8·33)
China	6 (7·50)	-
Germany	1 (1·25)	-
Iran	2 (2·50)	-
Italy	6 (7·50)	-
Japan	7 (8·75)	1 (8·33)
Norway	1 (1·25)	-
Romania	1 (1·25)	-
South Korea	1 (1·25)	-
Spain	3 (3·75)	-
Sweden	2 (2·50)	-
Turkey	1 (1·25)	-
United Kingdom	6 (7·50)	1 (8·33)
United States	15 (18·75)	-
Multi-national	26 (32·50)	9 (75·00)
**Interventions examined: frequency***		
Placebo/no treatment	61 (76·25)	12 (100·00)
Donepezil	47 (58·75)	4 (33·33)
Galantamine	20 (25·00)	4 (33·33)
Memantine	20 (25·00)	3 (25·00)
Rivastigmine**	18 (22·50)	1 (8·33)
**Effectiveness outcomes reported: frequency***		
Mini-Mental State Examination	57 (71·25)	6 (50·00)
Serious Adverse Events	46 (57·50)	12 (100·00)
**Funding**		
Industry-sponsored	48 (60·00)	12 (100·00)
Publicly-sponsored‡	9 (11·25)	-
Mixed	7 (8·75)	-
Not Reported	16 (20·0)	-
**Severity of Alzheimer's dementia: frequency (%)**		
Mild	3 (3·75)	-
Mild-Moderate	44 (55·00)	7 (58·33)
Mild-Severe	2 (2·50)	-
Moderate	3 (3·75)	-
Moderate-Severe	11 (13·75)	1 (8·33)
Severe	6 (7·50)	2 (16·67)

For peer review only

Not Reported	11 (13·75)	2 (16·67)
**Diagnostic criteria for Alzheimer's dementia: frequency***		
Mini-Mental State Examination	70 (87·50)	12 (100·00)
National Institute of Neurological Disorders and Stroke-Alzheimer Disease and Related Disorders Association	67 (83·75)	12 (100·00)
Diagnostic and Statistical Manual of Mental Disorders	39 (48·75)	5 (41·67)
Magnetic Resonance Imaging/Computerized Tomography	9 (11·25)	2 (16·67)
Clinical Dementia Rating	6 (7·50)	-
Hachinski Ischemic Score	5 (6·25)	-
Alzheimer's Disease Assessment Scale-Cognitive Subscale	3 (3·75)	1 (8·33)
Other	20 (25·00)	1 (8·33)

Abbreviations: -, not applicable
* Multiple interventions and outcomes reported per study;
** Rivastigmine refers to either oral or transdermal administration
ǂ Including sponsors such as the National Institute of Aging, UK Medical Research Council, and Veteran Affairs

615

For peer review only

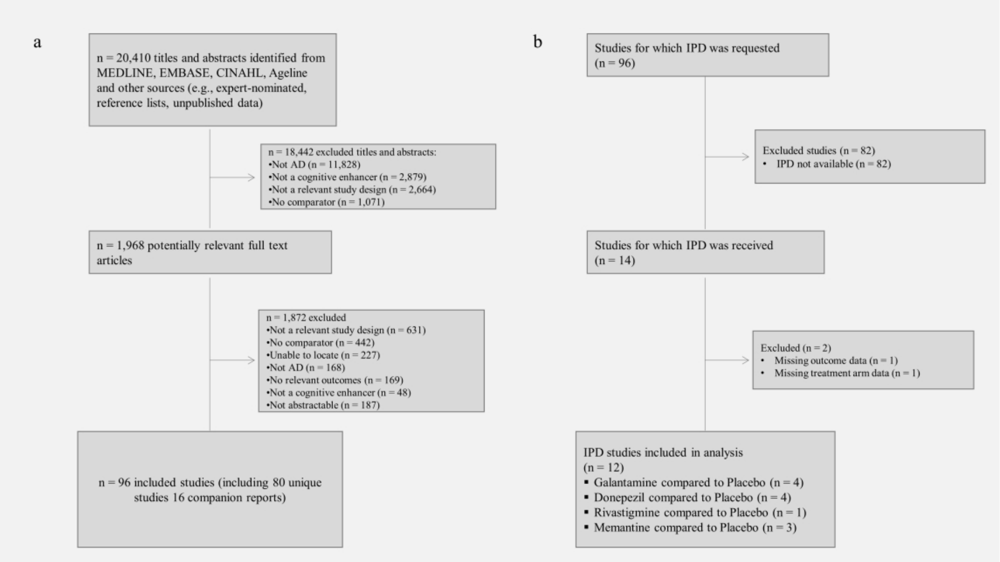

Figure 1. Flow diagram for study inclusion in the review (a) and studies retrieved with individual patient data (b).

338x190mm (300 x 300 DPI)

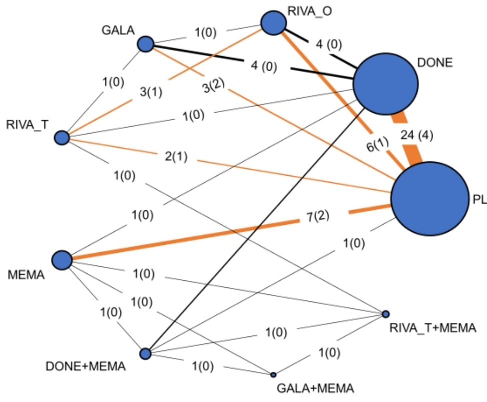

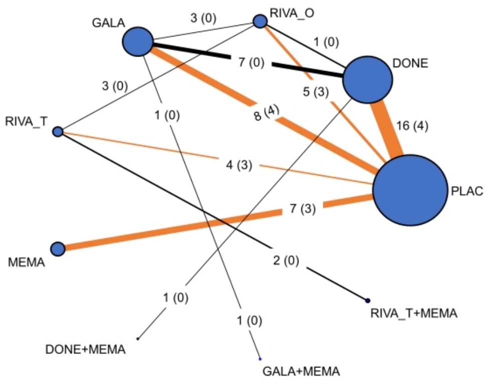

Figure 2. Network diagrams for (a) MMSE and (b) SAE outcomes. The size of each node and line indicates the number of studies included in each treatment comparison. The number of studies per treatment comparison is presented on each edge, and the number of studies with individual patient data (IPD) is depicted in a parenthesis. Orange coloured edges are informed by both IPD and aggregate data, whereas black coloured edges are informed by aggregate data only.

338x190mm (300 x 300 DPI)

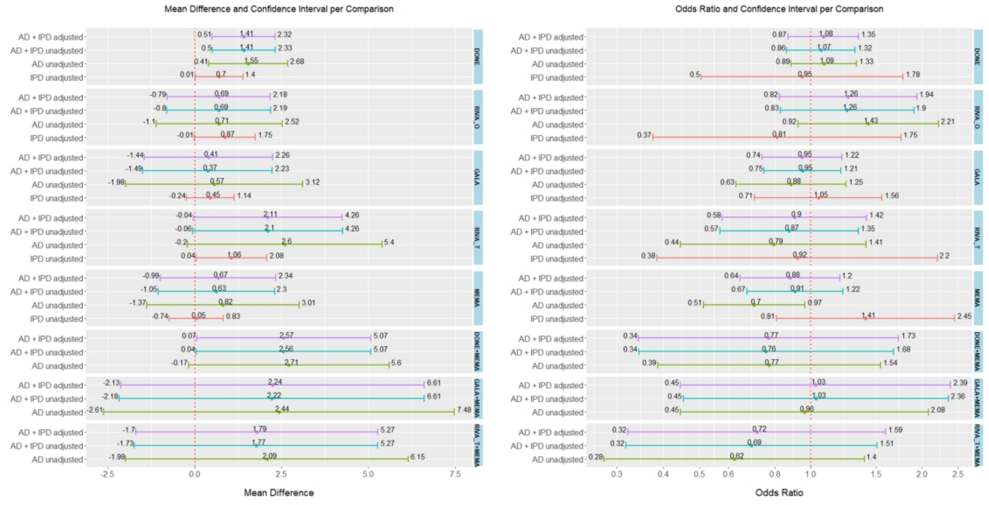

a. MMSE outcome b. SAE outcome

Figure 3. Forest plot of network meta-analysis (NMA) results for all cognitive enhancers versus placebo in (a) MMSE outcome, and (b) SAE outcome. NMA results are presented for i) aggregate data (AD) and fully adjusted results from studies with available individual patient data (IPD), ii) AD and crude results from studies with available IPD, iii) AD only (studies with available IPD are not included in the analysis), and iv) crude results from individual studies with individual patient data (IPD).

338x190mm (300 x 300 DPI)

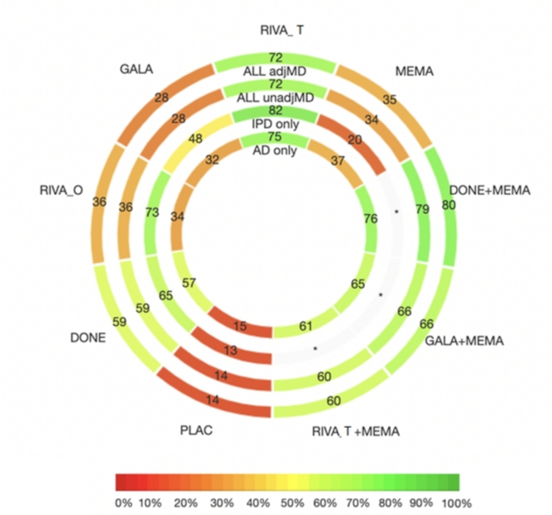

Figure 4. Rank-heat plot of P-scores for 9 treatments, including placebo, studied in randomized clinical trials with patients with Alzheimer's Dementia assessing MMSE. Circles from inside out present results for different network meta-analyses including: i) aggregate data (AD) only (studies with available IPD are not included in the analysis), ii) crude results from individual studies with individual patient data (IPD), iii) AD and crude results from studies with available IPD, and iv) AD and fully adjusted results from studies with available IPD. Numbers within each sector correspond to the P-score values as calculated in each model.

338x190mm (300 x 300 DPI)

Additional File 1: ***Comparative safety and efficacy of cognitive enhancers for Alzheimer's dementia: A systematic review with individual patient data network meta-analysis***

Appendix 1: Additional information on the methods used in the review 2
Appendix 2: Studies included in the systematic review....6
Appendix 3: Studies with available IPD but insufficient data to be included in the analysis..12
Appendix 4: List of studies requested and sponsor response....13
Appendix 5: Study characteristics of the included RCTs 16
Appendix 6. Characteristics of studies with shared IPD....23
Appendix 7: Comparison of studies with shared IPD with (a) all remaining studies and (b) studies for which sponsors claimed unavailable IPD. AD: aggregate data; IPD: individual patient data 26
Appendix 8: Cochrane Risk-of-bias appraisal results (n = 80) 28
Appendix 9: Overall risk of bias for studies with shared IPD against (a) all remaining studies and (b) studies for which sponsors claimed unavailable IPD. AD: aggregate data; IPD: individual patient data 30
Appendix 10: Study-specific effect sizes calculated from shared IPD and published data. IPD: individual patient data 31
Appendix 11: Correlation between participant age and dropout in studies with IPD. IPD: individual patient data 36
Appendix 12: Comparison Adjusted Funnel plot (all treatments vs placebo) 37
Appendix 13: Distribution of potential effect modifiers per treatment comparison and outcome 38
Appendix 14: Consistency Assessment – Loop-specific approach (using adjusted treatment effects)....41
Appendix 15: Network and standard meta-analysis results 42
Appendix 16: Network subgroup and meta-regression analysis results 45
Appendix 17: CINeMA results 52
Appendix 18: Rank-heat plot for serious adverse events....55
Appendix 19: Study definitions for serious adverse events 56
Appendix 20: Time taken to achieve at least one serious adverse event using individual patient data 59
Appendix 21: Challenges encountered during the individual patient data request from sponsors....60

For peer review only - http://bmjopen.bmj.com/site/about/guidelines.xhtml

Appendix 1: Additional information on the methods used in the review

Eligibility criteria, search strategy and study selection

We considered a SAE as defined in the individual trials, specifically, when an event led to disability or hospitalization or was life-threatening or fatal. Study definitions for a SAE were also abstracted. We included donepezil, rivastigmine, galantamine, and memantine alone or in combination with other treatment and compared with each other, supportive care or placebo. We excluded studies examining other cognitive enhancers or including individuals with mixed causes of dementia. We included published studies written in any language and of any duration.

Using terms from our previous review,4 the MEDLINE literature search was drafted by an experienced librarian (Dr. Laure Perrier) and revised after another librarian (Ms. Becky Skidmore) peer-reviewed the search terms.10 Subsequently, we searched the following databases: MEDLINE, EMBASE, Cochrane Methodology Register, CINAHL, Ageline and Cochrane Central Register of Controlled Trials. We also scanned reference lists of included studies and relevant reviews to supplement the electronic literature searches.

After pilot-testing, the results from the literature search were screened by pairs of reviewers working independently. Pairs of reviewers independently abstracted data (e.g., study characteristics, patient characteristics, outcome results) after a pilot-test. We resolved conflicts through discussion. The overall agreement among the reviewers for screening was over 70%.

IPD collection process and data abstraction

During the author contact process, two authors (a senior scientist ACT and a research assistant SL) sent a data request following several strategies as outlined in the RCT protocol:[1] a) an email requesting their IPD, b) email reminders (4 in total) at 2, 6, 10, and 14-week intervals after the initial email, c) reminders by post in week 7, and d) reminders via telephone in week 15. We also invited eligible authors to be a co-author on our updated systematic review provided that they share their anonymized IPD, and meet the International Committee of Medical Journal Editors (ICMJE) criteria for authorship.[2] Our team (AAV, SL) also contacted sponsors of the eligible trials, as reported in the publications. If a sponsor was not reported in a publication, we contacted the author (whom we emailed during the RCT) to determine who sponsored the study. To contact industry sponsors, we navigated the data sharing process from their websites or via an email, online portal, or phone inquiry. When no response was received, two follow-up reminders were sent to the sponsors.

We requested IPD on 1) patients: age, sex, severity of Alzheimer's disease (e.g. baseline MMSE level), presence of behavioral disturbance, comorbid conditions (e.g., stroke, cardiovascular conditions, Parkinson's disease), other medications used for each patient, number of drop-outs, reasons for drop-out, and number of participants, 2) medication: treatment each patient was allocated to, dosage, 3) outcomes: event, date of event, time taken to achieve the event for SAEs, MMSE values and measurement dates, and 4) date and method of randomization. We checked IPD provided for consistency with results from published RCTs., and contacted IPD providers when data inconsistencies were found.

Data extraction items included a) study characteristics: year of publication, country and continent according to the first author, journal in which the study was published, funding information; b) aggregate patient characteristics: study size and percentage of males, c) outcome data: study data (e.g., events or mean and standard deviations, and sample size per arm), and d) treatments compared. We also abstracted the corresponding authors' contact details. We categorized each study according to funding source (industry-sponsored, publicly-sponsored, mixed, and non-sponsored).

Certainty of the evidence

We used CINeMA (Confidence in Network Meta-Analysis) to assess confidence in the NMA estimates.[3] Six domains were evaluated with scores 'no concerns', 'some concerns' and 'major concerns': 1) within-study bias, 2) reporting bias, 3) indirectness, 4) imprecision, 5) heterogeneity, and 6) incoherence. We used the overall risk of bias per study, and for each treatment comparison we applied the average risk of bias. Similarly, for all treatment comparisons we used the average for indirectness. We assessed reporting bias based on the comparison-adjusted funnel plot since there are no established statistical methods to explore reporting bias. We used the *netfunnel* command in Stata to produce the comparison-adjusted funnel plot.[4] For imprecision,

For peer review only - http://bmjopen.bmj.com/site/about/guidelines.xhtml

we considered a MD=1.4 and a OR=1 as a clinically important size of effect for MMSE and SAE, respectively, and followed the CINeMA guidelines for exploring whether statistical significance and clinical importance coincide. Similarly, heterogeneity and incoherence (i.e. inconsistency) were assessed by following the standard CINEMA approach.

Statistical Analysis

We performed a descriptive analysis using frequencies and percentages of the discrete characteristics of the included patients and treatments of the eligible studies. We explored the distributions of the continuous patient characteristics per outcome and treatment group using means and standard deviations. For studies not providing outcome results for a certain outcome, we presented distributions of the available and requested patient characteristics, whenever available. Outliers for each patient characteristic were also explored in each study dataset using boxplots. We also recorded the number of missing participants per treatment group and overall. We compared the characteristics of the unavailable and the available by the sponsors' studies. In particular, we explored whether these were well-conducted according to overall risk of bias, and compared distributions of mean participant age, publication year, study duration, study size, percent male, and magnitude of treatment effect, to assess for potential bias in IPD sharing. We conducted a two-stage analysis for both standard meta-analysis and NMA. The network geometry was explored through the presentation of network plots.

First stage

All IPD from included studies were first aggregated to study-level summary statistics using each sponsor's portal. The use of different platforms and failure to obtain IPD from all studies restricted us from combining IPD in a one-stage analysis. For each separate study with IPD available, we fitted a logistic regression model for the binary outcome and a linear regression model for the continuous outcome. For MMSE, we considered the longest duration of follow-up per study (most frequently at week 24). In the shared IPD, when we were unable to make a judgement on first and last date of visit per patient, we used the older coded date and the newest coded date as baseline and final value for each patient respectively.

Initially, we did not adjust for any of the patient characteristics provided, but in a subsequent analysis we included patient-level covariates with as many interaction terms in the model as the patient characteristics were provided (considering only the ones we have asked for). For each study, we obtained the adjusted odds ratio (OR) for binary data and adjusted mean difference (MD) for continuous data, along their corresponding 95% confidence interval (CI). We adjusted for any of the following variables that were available in each study: age, sex, severity of Alzheimer's disease (e.g., baseline Mini-Mental State Examination [MMSE] level), presence of behavioural disturbance, comorbidity, and other medications. The first stage of the IPD analyses were conducted in RStudio,[5] which was available in data providers. Additional medications and comorbid conditions were grouped into broader categories according to their clinical relevance to increase power in our analysis (e.g., grouped medications as anti-psychotics, anti-depressants, and cognitive enhancers, as well as comorbid conditions as psychiatric, neurological, and cardiac disorders). Eligible studies with insufficient data to derive a pairwise estimate for NMA were summarized descriptively without performing a statistical analysis.

We applied an available case analysis for each study, since we were unable to install R packages in most sponsor-specific platforms, and hence we applied a consistent approach across all IPD datasets. We explored the impact of missing data during the second stage of analysis. Reasons for missing participants and time taken to have a serious adverse event were captured (when available).

We synthesized IPD at the first stage in four different proprietary sponsor-specific platforms. Analyses were conducted in the RStudio using different R versions[5] according to what was provided in each sponsor's platform: R version 3.4.1 for AbbVie, R version 3.4.3 for CSDR, R version 3.5.1 for YODA, R version 3.6.0 for Lundbeck.

Second stage

Since we were not successful in obtaining IPD for all eligible studies, we combined both IPD and aggregate data in a single meta-analysis or NMA model. Both IPD and aggregate data studies shared the same amount of heterogeneity. In both meta-analysis and NMA models, we combined the adjusted IPD estimates with the aggregate data (main analysis). As a secondary analysis, we combined the unadjusted estimates from retrieved

For peer review only - http://bmjopen.bmj.com/site/about/guidelines.xhtml

IPD with the evidence provided by the aggregated data studies in a joint NMA model. A common-within network between-study variance was assumed across comparisons for all NMA models.[6] We estimated the between-study variance using the DerSimonian and Laird[7] method and compared it with the relevant distributions provided by Turner et al[8] and Rhodes et al[9] to assess heterogeneity. We also calculated I^2 on the NMA level to quantify overall heterogeneity and inconsistency in each outcome.

To assess the validity of the transitivity assumption for each outcome, we assessed the distribution of potential effect modifiers (e.g., age, sex) across treatment comparisons in each network.[10-12] We visually inspected similarity and assessed whether these characteristics were likely to modify the treatment effect. We evaluated the consistency assumption using the design-by-treatment interaction model[13,14] and the loop-specific method.[15,16] In the presence of statistically significant inconsistency, we checked the data for discrepancies and if none were identified, we planned to conduct subgroup NMA or network meta-regression analysis adjusting for potential variables influencing the results.

We conducted additional NMA analyses for all potential effect modifiers requested from data providers. If relevant data were not available in the IPD, we used aggregate data of the relevant publications. Additional NMA analyses included: 1) subgroup analysis for industry vs. publicly sponsored studies, for studies with available IPD vs. studies with aggregate data (unadjusted estimates), and for AD severity, classified according to MMSE scores using the National Institute for Health and Care Excellence categories: mild (21–24), moderate (10–20), severe (<10),[17] 2) network meta-regression accounting for study duration, year of publication, mean age, and sex (% of male participants) effect modifiers separately and assuming a common regression coefficient across comparisons (studies with aggregate data were used only; studies with available IPD were pooled in a NMA separately adjusted for available covariates at first stage), 3) sensitivity analysis including studies with low risk of bias for allocation concealment and incomplete outcome data items, as these items may have an important impact on the meta-analysis results according to our previous NMA,[18] and 4) the 'informative missingness difference of means' (IMDoM) imputation method[19] for MMSE for the aggregate data studies to assess the impact of missing data in our NMA. In all additional NMA analyses, we used the adjusted effect estimates derived from the IPD within-study analysis and the aggregate data extracted from the eligible publications. Network meta-regression was performed in a Bayesian setting using OpenBUGS version 3.2.3, non-informative priors for all parameters in the model and a half-normal prior for the between standard deviation. We compared the results of the additional models by evaluating the treatment effect estimates and ranking statistics, as well as monitoring the reduction in the between-study variance.

Meta-analysis and NMA at the 2nd stage were conducted in the RStudio using R version 3.6.2 and the *meta*[20] and *netmeta*[21] packages, respectively.

Appendix 1 References:

1. Veroniki AA, Straus SE, Ashoor H, Stewart LA, Clarke M, Tricco AC. Contacting authors to retrieve individual patient data: study protocol for a randomized controlled trial. *Trials* 2016; **17**(1): 138.
2. Hager K, Baseman AS, Nye JS, et al. Effects of galantamine in a 2-year, randomized, placebo-controlled study in Alzheimer's disease. *Neuropsychiatric disease and treatment* 2014; **10**: 391-401.
3. CINeMA: Confidence in Network Meta-Analysis. Institute of Social and Preventive Medicine, University of Bern. *Available from cinemaispmch* 2017.
4. Chaimani A, Higgins JP, Mavridis D, Spyridonos P, Salanti G. Graphical tools for network meta-analysis in STATA. *PLoS One* 2013; **8**(10): e76654.
5. R Core Team. R: A Language and Environment for Statistical Computing. Vienna, Austria: R Foundation for Statistical Computing; 2019.
6. Lu G, Ades AE. Combination of direct and indirect evidence in mixed treatment comparisons. *Statistics in medicine* 2004; **23**(20): 3105-24.
7. DerSimonian R, Laird N. Meta-analysis in clinical trials. *Controlled clinical trials* 1986; **7**(3): 177-88.

For peer review only - http://bmjopen.bmj.com/site/about/guidelines.xhtml

8. Turner RM, Davey J, Clarke MJ, Thompson SG, Higgins JP. Predicting the extent of heterogeneity in meta-analysis, using empirical data from the Cochrane Database of Systematic Reviews. *International journal of epidemiology* 2012; **41**(3): 818-27.
9. Rhodes KM, Turner RM, Higgins JP. Predictive distributions were developed for the extent of heterogeneity in meta-analyses of continuous outcome data. *Journal of clinical epidemiology* 2015; **68**(1): 52-60.
10. Salanti G. Indirect and mixed-treatment comparison, network, or multiple-treatments meta-analysis: many names, many benefits, many concerns for the next generation evidence synthesis tool. *Res Synth Methods* 2012; **3**(2): 80-97.
11. Jansen JP, Naci H. Is network meta-analysis as valid as standard pairwise meta-analysis? It all depends on the distribution of effect modifiers. *BMC Med* 2013; **11**: 159.
12. Cipriani A, Higgins JP, Geddes JR, Salanti G. Conceptual and technical challenges in network meta-analysis. *Annals of internal medicine* 2013; **159**(2): 130-7.
13. Higgins JP, Jackson D, Barrett JK, Lu G, Ades AE, White IR. Consistency and inconsistency in network meta-analysis: concepts and models for multi-arm studies. *Res Synth Methods* 2012; **3**(2): 98-110.
14. White IR BJ, Jackson D, Higgins JPT. Consistency and inconsistency in network meta-analysis: model estimation using multivariate meta-regression. *Res Synth Methods* 2012; **3**(2): 15.
15. Song F, Altman DG, Glenny AM, Deeks JJ. Validity of indirect comparison for estimating efficacy of competing interventions: empirical evidence from published meta-analyses. *Bmj* 2003; **326**(7387): 472.
16. Veroniki AA, Vasiliadis HS, Higgins JP, Salanti G. Evaluation of inconsistency in networks of interventions. *International journal of epidemiology* 2013; **42**(1): 332-45.
17. National Institute for Health and Clinical Excellence. Donepezil, galantamine, rivastigmine and memantine for the treatment of Alzheimer's disease. London, UK, 2011.
18. Veroniki AA, Straus SE, Ashoor HM, et al. Comparative safety and effectiveness of cognitive enhancers for Alzheimer's dementia: protocol for a systematic review and individual patient data network meta-analysis. *BMJ open* 2016; **6**(1): e010251.
19. Mavridis D, White IR, Higgins JP, Cipriani A, Salanti G. Allowing for uncertainty due to missing continuous outcome data in pairwise and network meta-analysis. *Statistics in medicine* 2015; **34**(5): 721-41.
20. Balduzzi S, Rücker G, Schwarzer G. How to perform a meta-analysis with R: a practical tutorial. *Evidence-based mental health* 2019; **22**(4): 153-60.
21. Rücker G, Schwarzer G, Krahn U, König J. netmeta: Network Meta-Analysis using Frequentist Methods. R package version 0.9-8. 2018. https://CRAN.R-project.org/package=netmeta.

For peer review only - http://bmjopen.bmj.com/site/about/guidelines.xhtml

Appendix 2: Studies included in the systematic review

80 Main Studies:

1. Agid Y, Dubois B, Anand R, Gharabawi G, International Rivastigmine Investigators. Efficacy and tolerability of rivastigmine in patients with dementia of the Alzheimer type. *Current Therapeutic Research* 1998; **59**(12): 837-45.
2. Ancoli-Israel S, Amatniek J, Ascher S, Sadik K, Ramaswamy K. Effects of galantamine versus donepezil on sleep in patients with mild to moderate Alzheimer disease and their caregivers: a double-blind, head-to-head, randomized pilot study. *Alzheimer disease and associated disorders* 2005; **19**(4): 240-5.
3. Andersen F, Viitanen M, Halvorsen DS, Straume B, Wilsgaard T, Engstad TA. The effect of stimulation therapy and donepezil on cognitive function in Alzheimer's disease. A community based RCT with a two-by-two factorial design. *BMC neurology* 2012; **12**: 59.
4. Araki T, Wake R, Miyaoka T, et al. The effects of combine treatment of memantine and donepezil on Alzheimer's disease patients and its relationship with cerebral blood flow in the prefrontal area. *International journal of geriatric psychiatry* 2014; **29**(9): 881-9.
5. Bakchine S, Loft H. Memantine treatment in patients with mild to moderate Alzheimer's disease: results of a randomised, double-blind, placebo-controlled 6-month study. *Journal of Alzheimer's disease : JAD* 2008; **13**(1): 97-107.
6. Black SE, Doody R, Li H, et al. Donepezil preserves cognition and global function in patients with severe Alzheimer disease. *Neurology* 2007; **69**(5): 459-69.
7. Blesa González R, Boada Rovira M, Martínez Parra C, Gil-Saladié D, Almagro CA, Gobartt Vázquez AL. Evaluation of the convenience of changing the rivastigmine administration route in patients with Alzheimer disease. *Neurologia (Barcelona, Spain)* 2011; **26**(5): 262-71.
8. Burns A, Bernabei R, Bullock R, et al. Safety and efficacy of galantamine (Reminyl) in severe Alzheimer's disease (the SERAD study): a randomised, placebo-controlled, double-blind trial. *The Lancet Neurology* 2009; **8**(1): 39-47.
9. Burns A, Perry E, Holmes C, et al. A double-blind placebo-controlled randomized trial of Melissa officinalis oil and donepezil for the treatment of agitation in Alzheimer's disease. *Dementia and geriatric cognitive disorders* 2011; **31**(2): 158-64.
10. Burns A, Rossor M, Hecker J, et al. The effects of donepezil in Alzheimer's disease - results from a multinational trial. *Dementia and geriatric cognitive disorders* 1999; **10**(3): 237-44.
11. Choi SH, Park KW, Na DL, et al. Tolerability and efficacy of memantine add-on therapy to rivastigmine transdermal patches in mild to moderate Alzheimer's disease: a multicenter, randomized, open-label, parallel-group study. *Current medical research and opinion* 2011; **27**(7): 1375-83.
12. Corey-Bloom J. A randomized trial evaluating the efficacy and safety of ENA 713 (rivastigmine tartrate), a new acetylcholinesterase inhibitor, in patients with mild to moderately severe Alzheimer's disease. *Int J Geriatr Psyopharmacol* 1998; **1**: 55-65.
13. Creţu O, Szalontay AS, Chiriţă R, Chiriţă V. Effect of memantine treatment on patients with moderate-to-severe Alzheimer's disease treated with donepezil. *Revista medico-chirurgicala a Societatii de Medici si Naturalisti din Iasi* 2008; **112**(3): 641-5.
14. Dysken MW, Sano M, Asthana S, et al. Effect of vitamin E and memantine on functional decline in Alzheimer disease: the TEAM-AD VA cooperative randomized trial. *Jama* 2014; **311**(1): 33-44.
15. Farlow MR, Grossberg GT, Sadowsky CH, Meng X, Somogyi M. A 24-week, randomized, controlled trial of rivastigmine patch 13.3 mg/24 h versus 4.6 mg/24 h in severe Alzheimer's dementia. *CNS neuroscience & therapeutics* 2013; **19**(10): 745-52.
16. Feldman H, Gauthier S, Hecker J, Vellas B, Subbiah P, Whalen E. A 24-week, randomized, double-blind study of donepezil in moderate to severe Alzheimer's disease. *Neurology* 2001; **57**(4): 613-20.
17. Feldman HH, Lane R. Rivastigmine: a placebo controlled trial of twice daily and three times daily regimens in patients with Alzheimer's disease. *Journal of neurology, neurosurgery, and psychiatry* 2007; **78**(10): 1056-63.

For peer review only - http://bmjopen.bmj.com/site/about/guidelines.xhtml

18. Fox C, Crugel M, Maidment I, et al. Efficacy of memantine for agitation in Alzheimer's dementia: a randomised double-blind placebo controlled trial. *PloS one* 2012; **7**(5): e35185.
19. Frölich L, Ashwood T, Nilsson J, Eckerwall G. Effects of AZD3480 on cognition in patients with mild-to-moderate Alzheimer's disease: a phase IIb dose-finding study. *Journal of Alzheimer's disease : JAD* 2011; **24**(2): 363-74.
20. Fuschillo C, Ascoli E, Franzese G, et al. Alzheimer's disease and acetylcholinesterase inhibitor agents: a two-year longitudinal study. *Archives of gerontology and geriatrics Supplement* 2004; (9): 187-94.
21. Gault LM, Ritchie CW, Robieson WZ, Pritchett Y, Othman AA, Lenz RA. A phase 2 randomized, controlled trial of the α7 agonist ABT-126 in mild-to-moderate Alzheimer's dementia. *Alzheimer's & dementia (New York, N Y)* 2015; **1**(1): 81-90.
22. Gold M, Alderton C, Zvartau-Hind M, et al. Rosiglitazone monotherapy in mild-to-moderate Alzheimer's disease: results from a randomized, double-blind, placebo-controlled phase III study. *Dementia and geriatric cognitive disorders* 2010; **30**(2): 131-46.
23. Greenberg SM, Tennis MK, Brown LB, et al. Donepezil therapy in clinical practice: a randomized crossover study. *Archives of neurology* 2000; **57**(1): 94-9.
24. Grossberg GT, Manes F, Allegri RF, et al. The safety, tolerability, and efficacy of once-daily memantine (28 mg): a multinational, randomized, double-blind, placebo-controlled trial in patients with moderate-to-severe Alzheimer's disease taking cholinesterase inhibitors. *CNS drugs* 2013; **27**(6): 469-78.
25. Hager K, Baseman AS, Nye JS, et al. Effects of galantamine in a 2-year, randomized, placebo-controlled study in Alzheimer's disease. *Neuropsychiatric disease and treatment* 2014; **10**: 391-401.
26. Haig GM, Pritchett Y, Meier A, et al. A randomized study of H3 antagonist ABT-288 in mild-to-moderate Alzheimer's dementia. *Journal of Alzheimer's disease : JAD* 2014; **42**(3): 959-71.
27. Hernandez C, Unturbe F, Martinez-Lage P, Lucas A, Gregorio P, Alonso T. Effects of combined pharmacologic and cognitive treatment in the progression of moderate dementia: a two-year follow-up. *REVISTA ESPANOLA DE GERIATRIA Y GERONTOLOGIA* 2007; **42**(1): 3.
28. Herrmann N, Gauthier S, Boneva N, Lemming OM. A randomized, double-blind, placebo-controlled trial of memantine in a behaviorally enriched sample of patients with moderate-to-severe Alzheimer's disease. *International psychogeriatrics* 2013; **25**(6): 919-27.
29. Holmes C, Wilkinson D, Dean C, et al. The efficacy of donepezil in the treatment of neuropsychiatric symptoms in Alzheimer disease. *Neurology* 2004; **63**(2): 214-9.
30. Homma A, Imah Y, Hariguchi S. Late phase II clinical study of acetylcholinesterase inhibitor E 2020 in patients with alzheimer-type dementia-12-weeks double-blind, placebo-controlled study 3 mg/day, 5mg/day. *Clinical Evaluation* 1998; **26**: 251-84.
31. Homma A, Imai Y, Tago H, et al. Donepezil treatment of patients with severe Alzheimer's disease in a Japanese population: results from a 24-week, double-blind, placebo-controlled, randomized trial. *Dementia and geriatric cognitive disorders* 2008; **25**(5): 399-407.
32. Hong Z, Zhang Z, Wang L, et al. A randomized study comparing the effect and safety of galantamine and donepezil in patients with mild to moderate Alzheimer's disease. *Chin J Neurol* 2006; **39**(6): 379-82.
33. Howard R, McShane R, Lindesay J, et al. Donepezil and memantine for moderate-to-severe Alzheimer's disease. *New England Journal of Medicine* 2012; **366**(10): 893-903.
34. Howard RJ, Juszczak E, Ballard CG, et al. Donepezil for the treatment of agitation in Alzheimer's disease. *The New England journal of medicine* 2007; **357**(14): 1382-92.
35. Hu HT, Zhang ZX, Yao JL, et al. [Clinical efficacy and safety of akatinol memantine in treatment of mild to moderate Alzheimer disease: a donepezil-controlled, randomized trial]. *Zhonghua nei ke za zhi* 2006; **45**(4): 277-80.
36. Johannsen P, Salmon E, Hampel H, et al. Assessing therapeutic efficacy in a progressive disease: a study of donepezil in Alzheimer's disease. *CNS drugs* 2006; **20**(4): 311-25.
37. Jones RW, Soininen H, Hager K, et al. A multinational, randomised, 12-week study comparing the effects of donepezil and galantamine in patients with mild to moderate Alzheimer's disease. *International journal of geriatric psychiatry* 2004; **19**(1): 58-67.

For peer review only - http://bmjopen.bmj.com/site/about/guidelines.xhtml

38. Kadir A, Darreh-Shori T, Almkvist O, et al. PET imaging of the in vivo brain acetylcholinesterase activity and nicotine binding in galantamine-treated patients with AD. *Neurobiology of aging* 2008; **29**(8): 1204-17.

39. Kano O, Ito H, Takazawa T, et al. Clinically meaningful treatment responses after switching to galantamine and with addition of memantine in patients with Alzheimer's disease receiving donepezil. *Neuropsychiatric disease and treatment* 2013; **9**: 259-65.

40. Karaman Y, Erdoğan F, Köseoğlu E, Turan T, Ersoy AO. A 12-month study of the efficacy of rivastigmine in patients with advanced moderate Alzheimer's disease. *Dementia and geriatric cognitive disorders* 2005; **19**(1): 51-6.

41. Likitjaroen Y, Meindl T, Friese U, et al. Longitudinal changes of fractional anisotropy in Alzheimer's disease patients treated with galantamine: a 12-month randomized, placebo-controlled, double-blinded study. *European archives of psychiatry and clinical neuroscience* 2012; **262**(4): 341-50.

42. Lorenzi M, Beltramello A, Mercuri NB, et al. Effect of memantine on resting state default mode network activity in Alzheimer's disease. *Drugs & aging* 2011; **28**(3): 205-17.

43. Maher-Edwards G, Dixon R, Hunter J, et al. SB-742457 and donepezil in Alzheimer disease: a randomized, placebo-controlled study. *International journal of geriatric psychiatry* 2011; **26**(5): 536-44.

44. Marek GJ, Katz DA, Meier A, et al. Efficacy and safety evaluation of HSD-1 inhibitor ABT-384 in Alzheimer's disease. *Alzheimer's & dementia : the journal of the Alzheimer's Association* 2014; **10**(5 Suppl): S364-73.

45. Mazza M, Capuano A, Bria P, Mazza S. Ginkgo biloba and donepezil: a comparison in the treatment of Alzheimer's dementia in a randomized placebo-controlled double-blind study. *European journal of neurology* 2006; **13**(9): 981-5.

46. Mohs RC, Doody RS, Morris JC, et al. A 1-year, placebo-controlled preservation of function survival study of donepezil in AD patients. *Neurology* 2001; **57**(3): 481-8.

47. Moretti DV. Alpha rhythm oscillations and MMSE scores are differently modified by transdermal or oral rivastigmine in patients with Alzheimer's disease. *American journal of neurodegenerative disease* 2014; **3**(2): 72-83.

48. Mowla A, Mosavinasab M, Haghshenas H, Borhani Haghighi A. Does serotonin augmentation have any effect on cognition and activities of daily living in Alzheimer's dementia? A double-blind, placebo-controlled clinical trial. *Journal of clinical psychopharmacology* 2007; **27**(5): 484-7.

49. Nakamura Y, Imai Y, Shigeta M, et al. A 24-week, randomized, double-blind, placebo-controlled study to evaluate the efficacy, safety and tolerability of the rivastigmine patch in Japanese patients with Alzheimer's disease. *Dementia and geriatric cognitive disorders extra* 2011; **1**(1): 163-79.

50. Nakano S, Asada T, Matsuda H, Uno M, Takasaki M. Donepezil hydrochloride preserves regional cerebral blood flow in patients with Alzheimer's disease. *Journal of nuclear medicine : official publication, Society of Nuclear Medicine* 2001; **42**(10): 1441-5.

51. Nordberg A, Darreh-Shori T, Peskind E, et al. Different cholinesterase inhibitor effects on CSF cholinesterases in Alzheimer patients. *Current Alzheimer research* 2009; **6**(1): 4-14.

52. Pakdaman H, Harandi AA, Hatamian H, et al. Effectiveness and Safety of MLC601 in the Treatment of Mild to Moderate Alzheimer's Disease: A Multicenter, Randomized Controlled Trial. *Dementia and geriatric cognitive disorders extra* 2015; **5**(1): 96-106.

53. Peng D, Xianhao X, Wang L. Efficiency and safety assessment of donepezil for treating mild and moderate Alzheimer disease. *Chinese Journal of Tissue Engineering Research* 2005; **9**(13): 170-2.

54. Peskind ER, Potkin SG, Pomara N, et al. Memantine treatment in mild to moderate Alzheimer disease: a 24-week randomized, controlled trial. *The American journal of geriatric psychiatry : official journal of the American Association for Geriatric Psychiatry* 2006; **14**(8): 704-15.

55. Peters O, Fuentes M, Joachim LK, et al. Combined treatment with memantine and galantamine-CR compared with galantamine-CR only in antidementia drug naïve patients with mild-to-moderate Alzheimer's disease. *Alzheimer's & dementia (New York, N Y)* 2015; **1**(3): 198-204.

56. Reisberg B, Doody R, Stöffler A, Schmitt F, Ferris S, Möbius HJ. Memantine in moderate-to-severe Alzheimer's disease. *The New England journal of medicine* 2003; **348**(14): 1333-41.

For peer review only - http://bmjopen.bmj.com/site/about/guidelines.xhtml

57. Rockwood K, Fay S, Song X, MacKnight C, Gorman M. Attainment of treatment goals by people with Alzheimer's disease receiving galantamine: a randomized controlled trial. *CMAJ : Canadian Medical Association journal = journal de l'Association medicale canadienne* 2006; **174**(8): 1099-105.
58. Rockwood K, Mintzer J, Truyen L, Wessel T, Wilkinson D. Effects of a flexible galantamine dose in Alzheimer's disease: a randomised, controlled trial. *Journal of neurology, neurosurgery, and psychiatry* 2001; **71**(5): 589-95.
59. Rogers SL, Doody RS, Mohs RC, Friedhoff LT. Donepezil improves cognition and global function in Alzheimer disease: a 15-week, double-blind, placebo-controlled study. Donepezil Study Group. *Archives of internal medicine* 1998; **158**(9): 1021-31.
60. Rogers SL, Farlow MR, Doody RS, Mohs R, Friedhoff LT. A 24-week, double-blind, placebo-controlled trial of donepezil in patients with Alzheimer's disease. Donepezil Study Group. *Neurology* 1998; **50**(1): 136-45.
61. Rogers SL, Friedhoff LT. The efficacy and safety of donepezil in patients with Alzheimer's disease: results of a US Multicentre, Randomized, Double-Blind, Placebo-Controlled Trial. The Donepezil Study Group. *Dementia (Basel, Switzerland)* 1996; **7**(6): 293-303.
62. Saxton J, Hofbauer RK, Woodward M, et al. Memantine and functional communication in Alzheimer's disease: results of a 12-week, international, randomized clinical trial. *Journal of Alzheimer's disease : JAD* 2012; **28**(1): 109-18.
63. Scarpini E, Bruno G, Zappalà G, et al. Cessation versus continuation of galantamine treatment after 12 months of therapy in patients with Alzheimer's disease: a randomized, double blind, placebo controlled withdrawal trial. *Journal of Alzheimer's disease : JAD* 2011; **26**(2): 211-20.
64. Schmidt R, Ropele S, Pendl B, et al. Longitudinal multimodal imaging in mild to moderate Alzheimer disease: a pilot study with memantine. *Journal of neurology, neurosurgery, and psychiatry* 2008; **79**(12): 1312-7.
65. Seltzer B, Zolnouni P, Nunez M, et al. Efficacy of donepezil in early-stage Alzheimer disease: a randomized placebo-controlled trial. *Archives of neurology* 2004; **61**(12): 1852-6.
66. Shao ZQ. Comparison of the efficacy of four cholinesterase inhibitors in combination with memantine for the treatment of Alzheimer's disease. *International journal of clinical and experimental medicine* 2015; **8**(2): 2944-8.
67. Shimizu S, Kanetaka H, Hirose D, Sakurai H, Hanyu H. Differential effects of acetylcholinesterase inhibitors on clinical responses and cerebral blood flow changes in patients with Alzheimer's disease: a 12-month, randomized, and open-label trial. *Dementia and geriatric cognitive disorders extra* 2015; **5**(1): 135-46.
68. Solé-Padullés C, Bartrés-Faz D, Lladó A, et al. Donepezil treatment stabilizes functional connectivity during resting state and brain activity during memory encoding in Alzheimer's disease. *Journal of clinical psychopharmacology* 2013; **33**(2): 199-205.
69. Tariot PN, Cummings JL, Katz IR, et al. A randomized, double-blind, placebo-controlled study of the efficacy and safety of donepezil in patients with Alzheimer's disease in the nursing home setting. *Journal of the American Geriatrics Society* 2001; **49**(12): 1590-9.
70. Tariot PN, Solomon PR, Morris JC, Kershaw P, Lilienfeld S, Ding C. A 5-month, randomized, placebo-controlled trial of galantamine in AD. The Galantamine USA-10 Study Group. *Neurology* 2000; **54**(12): 2269-76.
71. Thomas A, Iacono D, Bonanni L, D'Andreamatteo G, Onofrj M. Donepezil, rivastigmine, and vitamin E in Alzheimer disease: a combined P300 event-related potentials/neuropsychologic evaluation over 6 months. *Clinical neuropharmacology* 2001; **24**(1): 31-42.
72. Wilcock G, Howe I, Coles H, et al. A long-term comparison of galantamine and donepezil in the treatment of Alzheimer's disease. *Drugs & aging* 2003; **20**(10): 777-89.
73. Wilkinson D. A Clinical Study Evaluating the Effects of Memantine on Brain Atrophy in Patients With Alzheimer's Disease; 2012.
74. Wilkinson D, Murray J. Galantamine: a randomized, double-blind, dose comparison in patients with Alzheimer's disease. *International journal of geriatric psychiatry* 2001; **16**(9): 852-7.

For peer review only - http://bmjopen.bmj.com/site/about/guidelines.xhtml

75. Wilkinson DG, Passmore AP, Bullock R, et al. A multinational, randomised, 12-week, comparative study of donepezil and rivastigmine in patients with mild to moderate Alzheimer's disease. *International journal of clinical practice* 2002; **56**(6): 441-6.
76. Winblad B, Cummings J, Andreasen N, et al. A six-month double-blind, randomized, placebo-controlled study of a transdermal patch in Alzheimer's disease--rivastigmine patch versus capsule. *International journal of geriatric psychiatry* 2007; **22**(5): 456-67.
77. Winblad B, Engedal K, Soininen H, et al. A 1-year, randomized, placebo-controlled study of donepezil in patients with mild to moderate AD. *Neurology* 2001; **57**(3): 489-95.
78. Winblad B, Kilander L, Eriksson S, et al. Donepezil in patients with severe Alzheimer's disease: double-blind, parallel-group, placebo-controlled study. *Lancet (London, England)* 2006; **367**(9516): 1057-65.
79. Zhang Y, Rong-jia Y, Ju-ying G, H. G, Chao-mei W. Effects of aricept on the treatment of Alzheimer disease evaluated by skull multi-slice helical CT. *Chin J Clin Rehab* 2005; **9**(25): 20-1.
80. Zhang Z, Yu L, Gaudig M, Schäuble B, Richarz U. Galantamine versus donepezil in Chinese patients with Alzheimer's disease: results from a randomized, double-blind study. *Neuropsychiatric disease and treatment* 2012; **8**: 571-7.

16 Companion Reports

1. Aronson S, Van Baelen B, Kavanagh S, Schwalen S. Optimal dosing of galantamine in patients with mild or moderate Alzheimer's disease: post Hoc analysis of a randomized, double-blind, placebo-controlled trial. *Drugs & aging* 2009; **26**(3): 231-9.
2. Cummings JL, Schneider L, Tariot PN, Kershaw PR, Yuan W. Reduction of behavioral disturbances and caregiver distress by galantamine in patients with Alzheimer's disease. *The American journal of psychiatry* 2004; **161**(3): 532-8.
3. Feldman H, Gauthier S, Hecker J, et al. Economic evaluation of donepezil in moderate to severe Alzheimer disease. *Neurology* 2004; **63**(4): 644-50.
4. Feldman H, Gauthier S, Hecker J, et al. Efficacy and safety of donepezil in patients with more severe Alzheimer's disease: a subgroup analysis from a randomized, placebo-controlled trial. *International journal of geriatric psychiatry* 2005; **20**(6): 559-69.
5. Gaudig M, Richarz U, Han J, Van Baelen B, Schäuble B. Effects of galantamine in Alzheimer's disease: double-blind withdrawal studies evaluating sustained versus interrupted treatment. *Current Alzheimer research* 2011; **8**(7): 771-80.
6. Gauthier S, Feldman H, Hecker J, Vellas B, Emir B, Subbiah P. Functional, cognitive and behavioral effects of donepezil in patients with moderate Alzheimer's disease. *Current medical research and opinion* 2002; **18**(6): 347-54.
7. Grossberg GT, Farlow MR, Meng X, Velting DM. Evaluating high-dose rivastigmine patch in severe Alzheimer's disease: analyses with concomitant memantine usage as a factor. *Current Alzheimer research* 2015; **12**(1): 53-60.
8. Han HJ, Kim BC, Lee JY, et al. Response to rivastigmine transdermal patch or memantine plus rivastigmine patch is affected by apolipoprotein E genotype in Alzheimer patients. *Dementia and geriatric cognitive disorders* 2012; **34**(3-4): 167-73.
9. Jelic V, Haglund A, Kowalski J, Langworth S, Winblad B. Donepezil treatment of severe Alzheimer's disease in nursing home settings. A responder analysis. *Dementia and geriatric cognitive disorders* 2008; **26**(5): 458-66.
10. Kumar V, Anand R, Messina J, Hartman R, Veach J. An efficacy and safety analysis of Exelon in Alzheimer's disease patients with concurrent vascular risk factors. *European journal of neurology* 2000; **7**(2): 159-69.
11. Ott BR, Blake LM, Kagan E, Resnick M. Open label, multicenter, 28-week extension study of the safety and tolerability of memantine in patients with mild to moderate Alzheimer's disease. *Journal of neurology* 2007; **254**(3): 351-8.

For peer review only - http://bmjopen.bmj.com/site/about/guidelines.xhtml

12. Reisberg B, Doody R, Stöffler A, Schmitt F, Ferris S, Möbius HJ. A 24-week open-label extension study of memantine in moderate to severe Alzheimer disease. *Archives of neurology* 2006; **63**(1): 49-54.
13. Schwam E, Xu Y. Cognition and function in Alzheimer's disease: identifying the transitions from moderate to severe disease. *Dementia and geriatric cognitive disorders* 2010; **29**(4): 309-16.
14. van Dyck CH, Tariot PN, Meyers B, Malca Resnick E. A 24-week randomized, controlled trial of memantine in patients with moderate-to-severe Alzheimer disease. *Alzheimer disease and associated disorders* 2007; **21**(2): 136-43.
15. Wimo A, Winblad B, Engedal K, et al. An economic evaluation of donepezil in mild to moderate Alzheimer's disease: results of a 1-year, double-blind, randomized trial. *Dementia and geriatric cognitive disorders* 2003; **15**(1): 44-54.
16. Winblad B, Grossberg G, Frölich L, et al. IDEAL: a 6-month, double-blind, placebo-controlled study of the first skin patch for Alzheimer disease. *Neurology* 2007; **69**(4 Suppl 1): S14-22.

For peer review only

For peer review only - http://bmjopen.bmj.com/site/about/guidelines.xhtml

Appendix 3: Studies with available IPD but insufficient data to be included in the analysis

A study[1] of 859 participants comparing transdermal rivastigmine vs. placebo included only IPD for the placebo arm. Another study[2] of 285 participants comparing 22·5 mg of galantamine vs. 30 mg of galantamine vs. 45 mg of galantamine vs. placebo did not provide information about the SAE or MMSE outcomes in the shared IPD.

CSDR: Novartis (study: NVT_SA_ENA713D1301) – Nakamura 2011

The study compares rivastigmine patch vs. placebo, but includes data only on placebo. Hence, we cannot conduct an analysis to convert data on their aggregated form so that to be included in our network meta-analysis. The IPD of this study included 288 participants in total.
According to the publication, 284 were allocated to the rivastigmine patch 5 cm2 group, 287 to the rivastigmine patch 10 cm2 group, and 288 to the placebo group.

Baseline characteristics of included patients

Characteristics	PLAC	Total	Missing Data	P-value	Outliers
Males	92 (32 %)	92 (32 %)	No	-	No
Age, mean (SD)	74.6 (7.4)	74.6 (7.4)	No	-	Yes - 1 value
SAE, events/sample size	19/288	19/288	No	-	-
Baseline MMSE, mean (SD)	16.6 (2.9)	16.6 (2.9)	Yes - 1 value	-	No
MMSE, mean (SD)	17.5 (3.4)	17.5 (3.4)	No	-	No
Change score, mean (SD)	0.9 (1.6)	0.9 (1.6)	Yes - 2 values	-	Yes - 41 values
Total number of patients	288 (100 %)	288			

YODA: JNJ-Study-GAL-93-01 –Wilkinson 2001

The study compares galantamine 22.5mg, 30mg and 45mg vs placebo. In our analysis we combined galantamine 22.5mg, 30mg and 45mg in a single group. However, we only descriptively can include this study in our paper - not in the network meta-analysis – as it does not provide any info about the SAE or MMSE outcomes (only total score for baseline). The IPD of this study included 285 participants in total.
According to the publication, 285 patients were randomized to: galantamine 18mg, 24mg, 36mg/day and placebo. Of the outcomes of interest, publication reported the SAE outcome. According to the sponsor there are no differences in the reporting of doses:

- galantamine hydrobromide 7.5 mg =6 mg galantamine base was administered tid i.e galantamine hydrobromide 22.5 mg/d = galantamine base **18mg/day**
- galantamine hydrobromide 10 mg =8 mg galantamine base was administered tid i.e galantamine hydrobromide 30mg/d= galantamine base **24mg/day** and
- galantamine hydrobromide 15 mg =12 mg galantamine base was administered tid i.e galantamine hydrobromide 45mg/d= galantamine base **36mg/day**

Baseline characteristics of included patients

Characteristics	GALA	PLAC	Total	Missing Data	P-value	Outliers
Males	85 (30%)	36 (12%)	121 (42%)	No	<0.001	No
Age, mean (SD)	73.5 (8.2)	74.2 (9.0)	73.8 (8.5)	No	0.242	Yes - 1 value
SAE, events/sample size*	-	-	-	-	-	-
Baseline MMSE, mean (SD)	18.6 (3.2)	18.8 (3.1)	18.7 (3.2)	No	0.616	No
MMSE, mean (SD)	-	-	-	-	-	-
Change score, mean (SD)	-	-	-	-	-	-
Total number of patients	198 (69%)	87 (31%)	285 (100%)			

*SAE in publication is as follows, PLAC: 3/87, GALA 18mg: 6/88, GALA 24mg: 0/56, GALA 36mg: 5/54

[1]Nakamura Y, Imai Y, Shigeta M, et al. A 24-week, randomized, double-blind, placebo-controlled study to evaluate the efficacy, safety and tolerability of the rivastigmine patch in Japanese patients with Alzheimer's disease. Dement Geriatr Cogn Dis Extra 2011; 1(1): 163-79.
[2] Wilkinson D, Murray J. Galantamine: a randomized, double-blind, dose comparison in patients with Alzheimer's disease. Int J Geriatr Psychiatry 2001; 16(9): 852-7.

For peer review only - http://bmjopen.bmj.com/site/about/guidelines.xhtml

Appendix 4: List of studies requested and sponsor response

Sponsor	Author, year	Interventions compared (dosage mg)*	Sponsor Response	IPD Received
Abbvie	Gault, 2015	Placebo/No treatment, Donepezil (10 mg)	Available	Yes
	Haig, 2014	Placebo/No treatment, Donepezil (5 – 10 mg)	Available	Yes
	Marek, 2014	Placebo/No treatment, Donepezil (5 – 10 mg)	Unavailable (Cannot share data (Potential business considerations under review))	No
AstraZeneca	Frolich, 2011	Placebo/No treatment, Donepezil (5 – 10 mg)	Available	No
Daiichi-Sankyo	Shimizu, 2015	Donepezil (5 mg), Galantamine (24 mg), Rivastigmine (18 mg)	Unavailable (Do not own data)	No
Eisai	Black, 2007	Placebo/No treatment, Donepezil (5 – 10 mg)	Available	Yes
	Burns, 1999	Placebo/No treatment, Donepezil (5 – 10 mg)	Unavailable (Cannot share data (Old study))	No
	Feldman, 2001	Placebo/No treatment, Donepezil (5 – 10 mg)	Unavailable (Do not own data)	No
	Feldman, 2004	Placebo/No treatment, Donepezil (5 – 10 mg)	Unavailable (Do not own data)	No
	Feldman, 2005	Placebo/No treatment, Donepezil (5 – 10 mg)	Unavailable (Do not own data)	No
	Gauthier, 2002	Placebo/No treatment, Donepezil (5 – 10 mg)	Unavailable (Do not own data)	No
	Holmes, 2004	Placebo/No treatment, Donepezil (10 mg)	Unavailable (Do not own data)	No
	Homma, 2008	Placebo/No treatment, Donepezil (10 mg)	Unavailable (Cannot share data (Old study))	No
	Johannsen, 2006	Placebo/No treatment, Donepezil (10 mg)	Unavailable (Do not own data)	No
	Jones, 2004	Donepezil (5 – 10 mg), Galantamine (8 – 24 mg)	Unavailable (Cannot share data (Old study))	No
	Mohs, 2001	Placebo/No treatment, Donepezil (5 – 10 mg)	Unavailable (Cannot share data (Old study))	No
	Rogers, 1996	Placebo/No treatment, Donepezil (5 mg)	Unavailable (Cannot share data (Old study))	No
	Rogers, 1998	Placebo/No treatment, Donepezil (10 mg)	Unavailable (Cannot share data (Old study))	No
	Rogers, 1998	Placebo/No treatment, Donepezil (10 mg)	Unavailable (Cannot share data (Old study))	No
	Schwam, 2010	Placebo/No treatment, Donepezil (5 – 10 mg)	Unavailable (Do not own data)	No
	Seltzer, 2004	Donepezil (5 – 10 mg), Placebo/No treatment	Unavailable (Cannot share data (Old study))	No
	Shimizu, 2015	Donepezil (5 mg), Galantamine (24 mg), Rivastigmine (18 mg)	Unavailable (Do not own data)	No
	Sole-Padulles, 2013	Placebo/No treatment, Donepezil (5 – 10 mg)	Unavailable (Do not own data)	No
	Tariot, 2001	Placebo/No treatment, Donepezil (5 – 10 mg)	Unavailable (Cannot share data (Old study))	No
	Wilkinson, 2002	Donepezil (5 – 10 mg), Rivastigmine (6 – 12 mg)	Unavailable (Do not own data)	No
Forest Laboratories/Allergen	Grossberg, 2013	Donepezil (NR) + Rivastigmine (13.3 mg) + Galantamine + Placebo, Donepezil (NR) + Rivastigmine (4.6 mg) + Galantamine (NR)+ Memantine (NR)	Unavailable (Cannot share data (No details provided))	No
	Ott, 2007	Placebo/No treatment, Memantine (5 -20 mg)	Unavailable (Cannot share data (No details provided))	No
	Peskind, 2006	Placebo/No treatment, Memantine (5 -20 mg)	Unavailable (Cannot share data (No details provided))	No
	Saxton, 2012	Placebo/No treatment, Memantine (20 mg)	Unavailable (Cannot share data (No details provided))	No
	van Dyck, 2007	Placebo/No treatment, Memantine (20 mg)	Unavailable (Cannot share data (No details provided))	No
GlaxoSmithKline	Gold, 2010	Placebo/No treatment, Donepezil (10 mg)	Available	Yes
	Maher-Edwards, 2011	Placebo/No treatment, Donepezil (5 – 10 mg)	Unavailable (Do not own data)	No
Janssen	Ancoli-Israel, 2005	Donepezil (10 mg), Galantamine (8 mg)	Unavailable (Cannot identify study)	No
	Aronson, 2009	Placebo/No treatment, Galantamine (16 – 24 mg)	Unavailable (Cannot identify study)	No
	Burns, 2009	Placebo/No treatment, Galantamine (8-24 mg)	Available	Yes
	Cummings, 2004	Placebo/No treatment, Galantamine (4, 8, 12 mg)	Available	Yes
	Gaudig, 2011	Placebo/No treatment, Galantamine (8 mg)	Unavailable (Cannot identify study)	No
	Hager K, 2014	Placebo/No treatment, Galantamine (8 – 24 mg)	Available	Yes
	Kadir, 2008	Placebo/No treatment, Galantamine (16 – 24 mg)	Unavailable (Cannot identify study)	No
	Likitjaroen, 2012	Placebo/No treatment, Galantamine (8 – 24 mg)	Unavailable(Do not own data)	No
	Rockwood, 2001	Placebo/No treatment, Galantamine (24, 32 mg)	Available	Yes
	Rockwood, 2006	Placebo/No treatment, Galantamine (16 – 24 mg)	Unavailable (IPD not available)	No
	Scarpini, 2011	Placebo/No treatment, Galantamine (16 mg)	Unavailable (IPD not available)	No

For peer review only - http://bmjopen.bmj.com/site/about/guidelines.xhtml

Sponsor	Author, year	Interventions compared (dosage mg)*	Sponsor Response	IPD Received
	Shimizu, 2015	Donepezil (5 mg), Galantamine (24 mg), Rivastigmine (18 mg)	Unavailable (Cannot identify study)	No
	Tariot, 2000	Placebo/No treatment, Galantamine (8 mg)	Unavailable (Cannot identify study)	No
	Wilcock, 2003	Donepezil (5 – 10 mg), Galantamine (16 – 24 mg)	Unavailable (Cannot identify study)	No
	Zhang, 2012	Donepezil (5 – 10 mg), Galantamine (6 – 16 mg or 6 – 24 mg)	Unavailable (IPD not available)	No
	Wilkinson, 2001	Placebo/No treatment, Galantamine (18 - 36 mg)	Available	Yes
Lundbeck	Bakchine, 2008	Placebo/No treatment, Memantine (20 mg)	Available	Yes
	Fox, 2012	Placebo/No treatment, Memantine (5 – 20 mg)	Unavailable (Do not own data)	No
	Herrmann, 2013	Placebo/No treatment, Memantine (5 – 20 mg)	Available	Yes
	Lorenzi, 2011	Placebo/No treatment, Memantine (5 – 20 mg)	Unavailable (Do not own data)	No
	Wilkinson, 2012	Placebo/No treatment, Memantine (5 – 20 mg)	Available	Yes
Merz	Reisberg, 2003	Placebo/No treatment, Memantine (20 mg)	No response from sponsor	No
	Reisberg, 2006	Placebo/No treatment, Memantine (20 mg)	No response from sponsor	No
	Schmidt, 2008	Placebo/No treatment, Memantine (5 – 20 mg)	No response from sponsor	No
	Winblad, 2007	Placebo/No treatment, Rivastigmine (3 – 12 mg)	No response from sponsor	No
Novartis	Agid, 1998	Placebo/No treatment, Rivastigmine (6 mg)	Unavailable (Cannot identify study)	No
	Blesa González, 2011	Placebo/No treatment, Rivastigmine (6 – 12 mg)	Unavailable (Cannot share data)	No
	Choi, 2011	Placebo/No treatment, Memantine (5 – 20 mg)	Unavailable (Do not own data)	No
	Corey-Bloom, 1998	Placebo/No treatment, Rivastigmine (6 – 12 mg)	Unavailable (Cannot identify study)	No
	Farlow, 2013	Rivastigmine (4.6 - 13.3 mg), Rivastigmine (4.6 mg) + Memantine (20 mg)	Unavailable (Cannot share data (Phase 4 study))	No
	Feldman, 2007	Placebo/No treatment, Rivastigmine (2 – 12 mg)	Unavailable (Cannot identify study)	No
	Grossberg, 2015	Rivastigmine (4.6 - 13.3 mg), Rivastigmine (4.6 mg) + Memantine (20 mg)	Unavailable (Cannot share data (Phase 4 study))	No
	Han, 2012	Placebo/No treatment, Memantine (5 – 20 mg)	Unavailable (Cannot identify study)	No
	Kumar, 2000	Placebo/No treatment, Rivastigmine (1 – 12 mg)	Unavailable (Cannot identify study)	No
	Nakamura, 2011	Placebo/No treatment, Rivastigmine (4.5 – 9.5 mg)	Available	Yes
	Nordberg, 2009	Donepezil (5 – 10 mg), Galantamine (8 – 24 mg), Rivastigmine (3 – 12 mg)	Unavailable (Cannot share data (Phase 4 study))	No
	Shimizu, 2015	Donepezil (5 mg), Galantamine (24 mg), Rivastigmine (18 mg)	Unavailable (Cannot identify study)	No
	Winblad, 2007	Placebo/No treatment, Rivastigmine (3 – 12 mg)	Available	Yes
ONO	Nakamura, 2011	Placebo/No treatment, Rivastigmine (4.5 – 9.5 mg)	No response from sponsor	No
Pfizer	Black, 2007	Placebo/No treatment, Donepezil (10 mg)	Unavailable (Do not own data)	No
	Feldman, 2001	Placebo/No treatment, Donepezil (5 – 10 mg)	Available	No
	Feldman, 2004	Placebo/No treatment, Donepezil (5 – 10 mg)	Unavailable (Cannot identify study)	No
	Feldman, 2005	Placebo/No treatment, Donepezil (5 – 10 mg)	Unavailable (Cannot identify study)	No
	Gauthier, 2002	Placebo/No treatment, Donepezil (5 – 10 mg)	Unavailable (Cannot identify study)	No
	Holmes, 2004	Placebo/No treatment, Donepezil (10 mg)	Unavailable (Cannot identify study)	No
	Jelic, 2008	Placebo/No treatment, Donepezil (5 – 10 mg)	Unavailable (Cannot identify study)	No
	Johannsen, 2006	Placebo/No treatment, Donepezil (10 mg)	Unavailable (Cannot identify study)	No
	Jones, 2004	Donepezil, Galantamine (8 – 24 mg)	Unavailable (Cannot identify study)	No
	Mohs, 2001	Placebo/No treatment, Donepezil (5 – 10 mg)	Unavailable (Cannot identify study)	No
	Schwam, 2010	Placebo/No treatment, Donepezil (5 – 10 mg)	Unavailable (Cannot identify study)	No
	Seltzer, 2004	Donepezil (5 – 10 mg), Placebo/No treatment	Unavailable (Cannot identify study)	No
	Sole-Padulles, 2013	Placebo/No treatment, Donepezil (5 – 10 mg)	Unavailable (Cannot identify study)	No
	Tariot, 2001	Placebo/No treatment, Donepezil (5 – 10 mg)	Unavailable (Cannot identify study)	No

For peer review only - http://bmjopen.bmj.com/site/about/guidelines.xhtml

Sponsor	Author, year	Interventions compared (dosage mg)*	Sponsor Response	IPD Received
	Wilkinson, 2002	Donepezil (5 – 10 mg), Rivastigmine (6 – 12 mg)	Unavailable (Cannot identify study)	No
	Wimo, 2003	Placebo/No treatment, Donepezil (5 – 10 mg)	Unavailable (Cannot identify study)	No
	Winblad, 2001	Placebo/No treatment, Donepezil (5 – 10 mg)	Unavailable (Cannot identify study)	No
	Winblad, 2006	Placebo/No treatment, Donepezil (5 – 10 mg)	Unavailable (Cannot identify study)	No
Roivant	Maher-Edwards, 2011	Placebo/No treatment, Donepezil (5 – 10 mg)	No response from sponsor	No
Shire Pharmaceuticals	Wilcock, 2003	Donepezil (5 – 10 mg), Galantamine (16 – 24 mg)	Unavailable (Do not own data)	No
	Wilkinson, 2001	Placebo/No treatment, Galantamine (24 mg)	Unavailable (Do not own data)	No
Takeda	Shimizu, 2015	Donepezil (5 mg), Galantamine (24 mg), Rivastigmine (18 mg)	Unavailable (Do not own data)	No
Non-Pharmaceutical	Andersen, 2012	Placebo/No treatment, Donepezil (5 – 10 mg)	NA	No
	Araki, 2014	Placebo/No treatment, Donepezil (NR) + Memantine (5 – 20 mg)	NA	No
	Burns, 2011	Placebo/No treatment, Donepezil (5 – 10 mg)	NA	No
	Dysken, 2014	Placebo/No treatment, Memantine (20 mg)	Available	No
	Greenberg, 2000	Placebo/No treatment, Donepezil (5 mg)	Unavailable (Need to contact PI)	No
	Howard, 2007	Placebo/No treatment, Donepezil (5 – 10 mg)	Unavailable (Do not own data)	No
	Howard, 2012	Donepezil (10 mg) + Memantine (5 – 20 mg), Donepezil (10 mg) + Placebo	Unavailable (Do not own data)	No
	Mowla, 2007	Placebo/No treatment, Rivastigmine (3 – 12 mg)	NA	No
	Peters, 2015	Galantamine (24 mg) + Placebo, Galantamine (24 mg) + Memantine (20 mg)	NA	No
Not reported	Cretu, 2008	Placebo/No treatment, Memantine (5 – 20 mg)	NA	No
	Fuschillo, 2001	Donepezil (5 mg), Rivastigmine (6 – 9 mg)	NA	No
	Hernández, 2007	Placebo/No treatment, Donepezil (10 mg)	NA	No
	Homma, 1998	Donepezil (3 – 5 mg), Placebo/no treatment	NA	No
	Hong, 2006	Placebo/No treatment, Galantamine (8 – 24 mg)	NA	No
	Hu, 2006	Donepezil (5 mg), Memantine (5 – 10 mg)	NA	No
	Kano, 2013	Donepezil(10 mg), Donepezil (10 mg) + Memantine (20 mg)	NA	No
	Karaman, 2005	Placebo/No treatment, Rivastigmine (3 – 12 mg)	NA	No
	Mazza, 2006	Placebo/No treatment, Donepezil (5 mg)	NA	No
	Moretti, 2014	Placebo/No treatment, Rivastigmine (3 – 12 mg)	NA	No
	Nakano, 2001	Placebo/No treatment, Donepezil (5 mg)	NA	No
	Pakdaman H, 2015	Donepezil (NR), Galantamine (NR), Rivastigmine (NR)	NA	No
	Peng, 2005	Placebo/No treatment, Donepezil (5 mg)	NA	No
	Shao, 2015	Memantine (5 – 10 mg)+ Placebo, Rivastigmine (1.5 – 3 mg) + Memantine (5 – 10 mg), Donepezil (5 – 10 mg) + Memantine (5 – 10 mg), Galantamine (2 – 6 mg) + Memantine (5 – 10 mg)	NA	No
	Thomas, 2001	Donepezil (5 – 10 mg), Rivastigmine (6 – 12 mg)	NA	No
	Zhang-Yi, 2005	Placebo/No treatment, Donepezil (5 mg)	NA	No

Abbreviations: NA, not applicable; NPH, neutral protamine Hagedorn; NR, not reported; PI, principal investigator

* In studies that examined different dosages of the same intervention, we selected the dosages that were consistent with those approved for use in Canada.

For peer review only

For peer review only - http://bmjopen.bmj.com/site/about/guidelines.xhtml

Appendix 5: Study characteristics of the included RCTs

Study	Country of conduct	Sample size; Longest duration of follow-up (weeks)	Treatments compared; Outcomes	Funding information	Date of randomization; Date trial opened; Randomization ratio	IPD available; Reasons for not providing IPD by the data providers
Agid, 1998	12 countries - Austria, Belgium, Czechoslovakia, Denmark, Finland, France, Germany, Ireland, Norway, Sweden, Switzerland, and the UK	402; 13	Rivastigmine, Placebo/No treatment; MMSE, Nausea, Vomiting, Diarrhea, SAEs, Headaches	Industry-sponsored	Not reported; Not reported; Not reported	No; Cannot identify study
Ancoli-Israel, 2005	USA	63; 8	Galantamine, Donepezil; CIBIC-plus, Mortality, Nausea, Diarrhea, SAEs, Headaches	Industry-sponsored	Not reported; Not reported; Not reported	No; Cannot identify study
Andersen, 2012	Norway	180; 52	Donepezil, Placebo; MMSE, ADAS-cog	Publicly-sponsored	Not reported; June 2003; Not reported	No; NA
Araki, 2014	Japan	37; 24	Donepezil + Memantine, Placebo; MMSE, NPI	Publicly-sponsored	Not reported; Not reported; Not reported	No; NA
Bakchine, 2008	12 countries -Austria, Belgium, Denmark, Finland, France, Greece, Lithuania, the Netherlands, Poland, Spain, Sweden and UK	470; 24	Memantine, Placebo/no treatment; ADAS-cog, ADCS-ADL, NPI, CIBIC-plus, Mortality, SAEs, Headaches, Falls	Industry-sponsored	Not reported; Not reported; Not reported	Yes; NA
Black, 2007	5 countries - USA, Canada, France, UK, Australia	343; 24	Donepezil, Placebo/No treatment; MMSE, ADCS-ADL, NPI, CIBIC-plus, Nausea, Vomiting, Diarrhea, SAEs	Industry-sponsored	Not reported; January 2001; Not reported	Yes; Do not own data
Blesa González, 2011	Spain	139; 12	Rivastigmine Patch, Rivastigmine Oral; MMSE, Nausea, Vomiting, Diarrhea	Industry-sponsored	Not reported; Not reported; Not reported	No; Cannot share data (Phase 4 study)
Burns, 1999	Australia, Belgium, Canada, France, Germany,Ireland, New Zealand, South Africa and the UK	818; 30	Donepezil, Placebo/no treatment; ADAS-cog, CIBIC-plus, Mortality, Diarrhea, Nausea, SAEs, Vomiting	Industry-sponsored	Not reported; Not reported; Not reported	No; Cannot share data (Old study)
Burns, 2009	Belgium, Finland, France, Italy, Norway, Netherlands, Spain, Sweden, Switzerland, UK	407; 26	Galantamine, Placebo/no treatment; Mortality, Nausea, Vomiting, Diarrhea, SAEs, Headaches, Falls	Industry-sponsored	Not reported; December 2003; Not reported	Yes; NA
Burns, 2011	UK	62; 12	Donepezil, Placebo/no treatment; NPI, SAEs	Publicly-sponsored	Not reported; January 2006; Not reported	No; NA
Choi, 2011	South Korea	171; 16	Memantine, Placebo/No treatment; MMSE, ADAS-cog, ADCS-ADL, NPI, SAEs, Nausea, Diarrhea, Vomiting, Headaches	Publicly-sponsored + Industry-sponsored	Not reported; December 2008; Not reported	No; Do not own data
Corey-Bloom, 1998	USA	699; 26	Rivastigmine, Placebo/No treatment; MMSE, ADAS-cog, CIBIC-plus, Mortality, Nausea, Vomiting	Industry-sponsored	Not reported; Not reported; Not reported	No; Cannot identify study

For peer review only - http://bmjopen.bmj.com/site/about/guidelines.xhtml

For peer review only

Cretu, 2008	Romania	43;<br>24	Memantine, Placebo/No treatment;<br>MMSE, ADAS-cog, NPI	NA	Not reported;<br>Not reported;<br>Not reported	No;<br>NR
Dysken, 2014	USA	307;<br>26-208	Memantine, Placebo;<br>MMSE, ADAS-cog, ADCS-ADL,<br>NPI, Mortality, SAEs	Publicly-<br>sponsored	Not reported;<br>August 2007;<br>1:1:1:1	No;<br>NA
Farlow, 2013	USA	716;<br>24	Rivastigmine + Memantine,<br>Rivastigmine;<br>NPI, Mortality, Falls, Vomiting,<br>Diarrhea, Nausea	Industry-<br>sponsored	Not reported;<br>July 2009;<br>1:1	No;<br>Cannot share data (Phase<br>4 study)
Feldman, 2001	Canada, Australia, France	290;<br>24	Donepezil, Placebo/No treatment;<br>MMSE, NPI, CIBIC-plus, Mortality,<br>Vomiting, Nausea, Diarrhea, SAEs,<br>Headaches	Industry-<br>sponsored	Not reported;<br>Not reported;<br>"50/50 split"	No;<br>NA
Feldman, 2007	Australia, Canada, Ireland, Italy,<br>South Africa, UK	450;<br>26	Rivastigmine, Placebo/No treatment;<br>MMSE, ADAS-cog, CIBIC-plus,<br>SAEs, Bradycardia, Headaches,<br>Vomiting, Diarrhea, Nausea	Industry-<br>sponsored	Not reported;<br>Not reported;<br>1:1:1	No;<br>Cannot identify study
Fox, 2012	UK	149;<br>12	Memantine, Placebo;<br>MMSE, NPI, Mortality	Industry-<br>sponsored	Not reported;<br>September 2007;<br>"assigned with equal<br>probability"	No;<br>Unavailable (Do not own<br>data)
Frolich, 2011	Austria, Belgium, Bulgaria, Czech<br>Republic, Germany, Romania,<br>Russia, Spain, UK, Canada	324;<br>12	Donepezil, Placebo/No treatment;<br>MMSE, ADAS-cog, Nausea,<br>Vomiting, Diarrhea, Headaches	Industry-<br>sponsored	Not reported;<br>July 2007;<br>Not reported	No;<br>Available
Fuschillo, 2001	Italy	27;<br>30	Donepezil, Rivastigmine;<br>MMSE, ADAS-cog, Headaches,<br>Vomiting, Diarrhea, Nausea	NA	Not reported;<br>Not reported;<br>Not reported	No;<br>NR
Gault, 2015	USA, Bulgaria, Czech Republic,<br>Slovakia, UK, South Africa	136;<br>14	Donepezil, Placebo;<br>MMSE, ADAS-cog, ADCS-ADL,<br>NPI, CIBIC-plus, Mortality, SAEs,<br>Bradycardia, Falls, Headaches,<br>Vomiting, Diarrhea, Nausea	Industry-<br>sponsored	Not reported;<br>October 2009;<br>Not reported	Yes;<br>Available
Gold, 2010	Austria, Bulgaria, Chile, China,<br>Croatia, Estonia, Germany, Greece,<br>Hungary, Mexico, New Zealand,<br>Pakistan, Peru, Republic of the<br>Philippines, Puerto Rico, Republic of<br>Korea, Russian Federation, UK and<br>USA	248;<br>24	Donepezil, Placebo/no treatment;<br>ADAS-cog, CIBIC-plus, Mortality,<br>Headaches, Nausea, Diarrhea, SAEs	Industry-<br>sponsored	Not reported;<br>February 2007;<br>2:2:2:1	Yes;<br>Available
Greenberg, 2000	USA	103;<br>24	Donepezil, Placebo/no treatment;<br>ADAS-cog, SAEs, Diarrhea, Nausea	Publicly-<br>sponsored	Not reported;<br>Not reported;<br>Not reported	No;<br>Contact PI
Grossberg, 2013	Argentina, USA, Mexico, Chile	676;<br>24	Donepezil + Rivastigmine +<br>Galantamine + Memantine, Donepezil<br>+ Rivastigmine + Galantamine +<br>Placebo;<br>NPI, CIBIC-plus, Mortality, Falls,	Industry-<br>sponsored	Not reported;<br>June 2005;<br>1:1	No;<br>Cannot share dat

For peer review only - http://bmjopen.bmj.com/site/about/guidelines.xhtml

			Headaches, Vomiting, Diarrhea, Nausea, SAEs			
Hager K, 2014	Czech Republic, Estonia, France, Germany, Greece, Italy, Latvia, Lithuania, Romania, Russia, Slovakia, Slovenia, Ukraine	2045; 104	Galantamine, Placebo; MMSE, Mortality, Headaches, Vomiting, Diarrhea, Nausea, SAEs	Industry-sponsored	Not reported; May 2008; 1:1	Yes; NA
Haig, 2014	Russia, Ukraine	123; 12	Donepezil, Placebo; MMSE, ADAS-cog, ADCS-ADL, NPI, Headaches, Nausea, SAEs	Industry-sponsored	Not reported; Not reported; 1:1:1	Yes; NA
Hernández, 2007	Spain	20; 48	Donepezil, Placebo/No treatment; MMSE, ADAS-cog	NA	Not reported; Not reported; Not reported	No; NR
Herrmann, 2013	Canada	369; 24	Memantine, Placebo; NPI, Mortality, Falls, Nausea, SAEs	Industry-sponsored	Not reported; December 2003; "equally allocated"	Yes; NA
Holmes, 2004	UK	96; 24	Donepezil, Placebo/No treatment; MMSE, NPI	Industry-sponsored	Not reported; Not reported; 3:2	No; Cannot identify study
Homma, 1998	Japan	187; 12	Donepezil, Placebo/no treatment; ADAS-cog, Mortality, SAEs, Headaches	NA	Not reported; Not reported; Not reported	No; NR
Homma, 2008	Japan	267; 24	Donepezil, Placebo/no treatment; ADCS-ADL, CIBIC-plus, Mortality, SAEs, Falls, Vomiting, Diarrhea	Industry-sponsored	Not reported; Not reported; 1:1:1	No; Cannot share data (Old study)
Hong, 2006	China	218; 16	Galantamine, Placebo/no treatment; ADAS-cog, ADCS-ADL, NPI, SAEs	NA	Not reported; Not reported; Not reported	No; NR
Howard, 2007	England	259; 12	Donepezil, Placebo/No treatment; MMSE, NPI, Mortality, Falls, Diarrhea	Publicly-sponsored	Not reported; November 2003; "probability ratios of 0.75 and 0.25 to assign treatment"	No; NA
Howard, 2012	Europe	295; 52	Donepezil + Placebo, Donepezil + Memantine; MMSE, Mortality, SAEs, Falls	Publicly-sponsored	Not reported; February 2008; Not reported	No; Do not own data
Hu, 2006	China	97; 16	Memantine, Donepezil; MMSE	NA	Not reported; Not reported; Not reported	No; NA
Johannsen, 2006	Belgium, Denmark, Germany, Greece, Hungary, Iceland, The Netherlands, Poland, USA	202; 48	Donepezil, Placebo/No treatment; MMSE, ADAS-cog, NPI, Headaches, Diarrhea, Nausea	Industry-sponsored	Not reported; February 1999; Not reported	No; Do not own data
Jones, 2004	UK, Finland, Germany and Norway	120; 12	Donepezil, Galantamine; MMSE, ADAS-cog, Headaches, Vomiting, Diarrhea, Nausea, SAEs	Industry-sponsored	Not reported; Not reported; 1:1	No; Cannot share data (Old study)
Kadir, 2008	Sweden	18; 48	Galantamine, Placebo/No treatment; MMSE, ADAS-cog	Industry-sponsored + Other	Not reported; Not reported; Not reported	No; Cannot identify study

For peer review only - http://bmjopen.bmj.com/site/about/guidelines.xhtml

Kano, 2013;	Japan	30;<br>28	Donepezil, Donepezil + Memantine ;<br>MMSE	NA	Not reported;<br>August 2011;<br>Not reported	No;<br>NR
Karaman, 2005	Turkey	44;<br>52	Rivastigmine, Placebo/No treatment;<br>MMSE, ADAS-cog, ADAS-ADL, CIBIC-plus, Headaches, Vomiting, Nausea	NA	Not reported;<br>Not reported;<br>Not reported	No;<br>NR
Likitjaroen, 2012	Germany	25;<br>26	Galantamine, Placebo;<br>MMSE	Publicly-sponsored + Industry-sponsored	Not reported;<br>September 2006;<br>Not reported	No;<br>Do not own data
Lorenzi, 2011	Italy	15;<br>24	Memantine, Placebo/No treatment;<br>MMSE	Publicly-sponsored + Industry-sponsored	Not reported;<br>Not reported;<br>Not reported	No;<br>Do not own data
Maher-Edwards, 2011	Austria, Bulgaria, Chile, Estonia, Germany, Russia, Slovakia, and UK	129;<br>24	Donepezil, Placebo/no treatment;<br>ADAS-cog, CIBIC-plus, Mortality, SAEs, Headaches, Nausea	Industry-sponsored	Not reported;<br>May 2006;<br>1:1:1	No;<br>No response from sponsor
Marek, 2014	UK, Ukraine, South Africa, Russia	132;<br>16	Donepezil, Placebo;<br>MMSE, ADAS-cog, NPI, CIBIC-plus, Mortality, Headaches, Vomiting, Diarrhea, SAEs	Industry-sponsored	Not reported;<br>May 2010;<br>"equal proportions"	No;<br>Cannot share data
Mazza, 2006	Italy	51;<br>24	Donepezil, Placebo/No treatment;<br>MMSE	NA	Not reported;<br>March 2003;<br>1:1:1	No;<br>NR
Mohs, 2001	USA	431;<br>54	Donepezil, Placebo/No treatment;<br>MMSE, Mortality, SAEs, Headaches, Diarrhea, Nausea	Industry-sponsored	Not reported;<br>Not reported;<br>Not reported	No;<br>Cannot share data
Moretti, 2014	Italy	20;<br>78	Rivastigmine Patch, Rivastigmine Oral;<br>MMSE	NA	Not reported;<br>Not reported;<br>Not reported	No;<br>NA
Mowla, 2007	Iran	81;<br>12	Rivastigmine, Placebo/No treatment;<br>MMSE	Publicly-sponsored	Not reported;<br>Not reported;<br>Not reported	No;<br>NA
Nakamura, 2011	Japan	855;<br>24	Rivastigmine, Placebo/No treatment;<br>MMSE, SAEs, Vomiting, Nausea, Diarrhea	Industry-sponsored	Not reported;<br>January 2007;<br>Not reported	Yes;<br>NA
Nakano, 2001	Japan	35;<br>48	Donepezil, Placebo/No treatment;<br>MMSE	NA	Not reported;<br>Not reported;<br>Not reported	No;<br>NR
Nordberg, 2009	USA	63;<br>13	Rivastigmine, Donepezil, Galantamine;<br>SAEs, Headaches, Vomiting, Diarrhea, Nausea	Industry-sponsored	Not reported;<br>Not reported;<br>1:1:1	No;<br>Cannot share data
Pakdaman H, 2015	Iran	198;<br>68.8	Donepezil, Galantamine, Rivastigmine;<br>MMSE, ADAS-cog, Mortality,	Industry-sponsored	Not reported;<br>Not reported;<br>Not reported	No;<br>NR

For peer review only - http://bmjopen.bmj.com/site/about/guidelines.xhtml

			Headaches, Vomiting, Diarrhea, Nausea			
Peng, 2005	China	89;<br>12	Donepezil, Placebo/No treatment;<br>MMSE	NA	Not reported;<br>1998;<br>Not reported	No;<br>NR
Peskind, 2006	USA	403;<br>24	Memantine, Placebo/no treatment;<br>ADAS-cog, ADCS-ADL, NPI, CIBIC-plus, Nausea, Vomiting, Diarrhea	Industry-sponsored	Not reported;<br>Not reported;<br>Not reported	No;<br>Cannot share data
Peters, 2015	Europe	226;<br>52	Galantamine + Memantine, Galantamine + Placebo;<br>ADAS-cog, ADCS-ADL, NPI, Mortality, SAEs, Falls	Publicly-sponsored	Not reported;<br>Not reported;<br>Not reported	No;<br>NA
Reisberg, 2003	USA	252;<br>28	Memantine, Placebo/No treatment;<br>MMSE, ADCS-ADL, NPI, CIBIC-plus, Mortality, SAEs, Diarrhea	Publicly-sponsored + Industry-sponsored	Not reported;<br>August 1998;<br>Not reported	No;<br>No response from sponsor
Rockwood, 2001	Australia, Canada, Great Britian, New Zealand, South Africa, USA	386;<br>12	Galantamine, Placebo/no treatment;<br>ADAS-cog, NPI, CIBIC-plus, Mortality, SAEs, Vomiting, Nausea	Industry-sponsored	Not reported;<br>Not reported;<br>Not reported	Yes;<br>NA
Rockwood, 2006	Canada	130;<br>16	Galantamine, Placebo/no treatment;<br>ADAS-cog, CIBIC-plus, SAEs, Vomiting, Nausea	Publicly-sponsored + Industry-sponsored	Not reported;<br>November 2001;<br>Not reported	No;<br>IPD not available
Rogers, 1996	USA	161;<br>12	Donepezil, Placebo/No treatment;<br>MMSE, ADAS-cog, Headaches, Diarrhea	Industry-sponsored	Not reported;<br>Not reported;<br>Not reported	No;<br>Cannot share data
Rogers, 1998	USA	468;<br>12	Donepezil, Placebo/No treatment;<br>MMSE, ADAS-cog, CIBIC-plus, SAEs, Headaches, Vomiting, Diarrhea, Nausea	Industry-sponsored	Not reported;<br>Not reported;<br>Not reported	No;<br>Cannot share data
Rogers, 1998	USA	473;<br>24	Donepezil, Placebo/No treatment;<br>MMSE, ADAS-cog, CIBIC-plus, Mortality, SAEs, Vomiting, Diarrhea, Nausea	Industry-sponsored	Not reported;<br>Not reported;<br>Not reported	No;<br>Cannot share data
Saxton, 2012	Australia, South Africa, New Zealand	264;<br>12	Memantine, Placebo;<br>Mortality, Falls, Headaches, Diarrhea, Nausea, SAEs	Industry-sponsored	Not reported;<br>April 2007;<br>Not reported	No;<br>Cannot share data
Scarpini, 2011	Italy	139;<br>96	Galantamine, Placebo/no treatment;<br>Mortality, SAEs	Industry-sponsored	Not reported;<br>July 2001;<br>Not reported	No;<br>IPD not available
Schmidt, 2008	Europe	36;<br>52	Memantine, Placebo/No treatment;<br>MMSE, ADAS-cog, ADCS-ADL	Industry-sponsored	Not reported;<br>Not reported;<br>Not reported	No;<br>No response from sponsor
Seltzer, 2004	USA	153;<br>24	Donepezil, Placebo/No treatment;<br>MMSE, ADAS-cog, Diarrhea, Nausea	Industry-sponsored	Not reported;<br>Not reported;<br>Not reported	No;<br>Cannot identify study

For peer review only

Shao, 2015	China	110; 24	Donepezil + Memantine, Galantamine + Memantine, Memantine + Placebo, Rivastigmine + Memantine; MMSE, ADCS-ADL	NA	Not reported; October 2009; Not reported	No; NR
Shimizu, 2015	Japan	75; 52	Donepezil, Galantamine, Rivastigmine; MMSE, ADAS-cog, NPI, Headaches, Vomiting, Nausea	Industry-sponsored	Not reported; Not reported; Not reported	No; Do not own data
Sole-Padulles, 2013	Spain	14; 13	No treatment, Donepezil; MMSE, NPI	Industry-sponsored	Not reported; Not reported; Not reported	No; Do not own data
Tariot, 2000	USA	978; 20	Galantamine, Placebo/no treatment; ADAS-cog, ADCS-ADL, NPI, Mortality, SAEs, Vomiting, Diarrhea, Nausea	Industry-sponsored	Not reported; Not reported; Not reported	No; Cannot identify study
Tariot, 2001	USA	208; 24	Donepezil, Placebo/No treatment; MMSE, Mortality, SAEs, Bradycardia, Headaches, Vomiting, Diarrhea, Nausea	Industry-sponsored	Not reported; Not reported; Not reported	No; Cannot identify study
Thomas, 2001	Italy	40; 24	Donepezil, Rivastigmine; MMSE, ADAS-cog	NA	Not reported; Not reported; Not reported	No; NR
Wilcock, 2003	UK	188; 52	Galantamine, Donepezil; MMSE, ADAS-cog, Mortality, SAEs, Falls, Headaches, Vomiting, Nausea	Industry-sponsored	Not reported; June 2000; Not reported	No; Cannot identify study
Wilkinson, 2001	UK	180; 12	Galantamine, Placebo/no treatment; ADAS-cog, SAEs, Headaches, Vomiting, Diarrhea, Nausea	Industry-sponsored	Not reported; May 1994; Not reported	Yes; NA
Wilkinson, 2002	UK, South Africa, and Switzerland	111; 12	Donepezil, Rivastigmine; MMSE, ADAS-cog, Mortality, SAEs, Bradycardia, Headaches, Vomiting, Nausea	Industry-sponsored	Not reported; Not reported; 1:1	No; Cannot identify study
Wilkinson, 2012	France, Germany, Switzerland, UK	277; 52	Memantine, Placebo/No treatment; MMSE, NPI, Mortality, SAEs, Falls	Industry-sponsored	Not reported; September 2005; 1:1	Yes; NA
Winblad, 2001	Denmark, Finland, Norway, Sweden, the Netherlands	286; 52	Donepezil, Placebo/No treatment; MMSE, SAEs, Bradycardia, Headaches, Diarrhea, Nausea	Industry-sponsored	Not reported; Not reported; Not reported	No; Cannot identify study
Winblad, 2006	Sweden	248; 24	Donepezil, Placebo/No treatment; MMSE, NPI, Mortality, SAEs, Falls, Diarrhea, Nausea	Industry-sponsored	Not reported; October 2002; Not reported	No; Cannot identify study
Winblad, 2007	Chile, Czech Republic, Denmark, Finland, Germany, Guatemala, Israel, Italy, Korea, Mexico, Norway, Peru, Poland, Portugal, Russia, Slovak Republic, Sweden, Taiwan, USA, Uruguay, Venezuela	1190; 24	Rivastigmine, Placebo/No treatment; MMSE, ADAS-cog, ADCS-ADL, NPI, Mortality, SAEs, Headaches, Vomiting, Diarrhea, Nausea	Industry-sponsored	Not reported; November 2003; Not reported	No; No response from sponsor

For peer review only

Zhang-Yi, 2005	China	120;<br>8	Donepezil, Placebo/No treatment;<br>MMSE	NA	Not reported;<br>Not reported;<br>Not reported	No;<br>NR
Zhang, 2012	China	218;<br>16	Galantamine, Donepezil;<br>MMSE, ADAS-cog, ADCS-ADL, NPI, Mortality, Vomiting, Diarrhea, Nausea, SAEs	Industry-sponsored	Not reported;<br>Not reported;<br>Not reported	No;<br>IPD not available

For peer review only

For peer review only - http://bmjopen.bmj.com/site/about/guidelines.xhtml

Appendix 6. Characteristics of studies with shared IPD

Study	Provided by	Severity of AD*	Previous response to treatment for AD	Presence of behavioural disturbance	Comorbid conditions	Other medications used	Treatment Group	Males (%)	Age, mean (SD)
Black 2007	CSDR - EISAI	Severe	NR	NR	All patients included the same exact comorbidities	NR	Donepezil	48 (27%)	78 (7.9)
							Placebo	54 (32%)	78 (8.1)
Gold 2010	CSDR - GSK	Mild-Moderate	NR	NR	Multiple reported	Multiple reported	Donepezil	16 (29%)	76.6 (8.2)
							Placebo	49 (46%)	75.5 (8.2)
Winblad 2007	CSDR - Novartis	Mild-Moderate	NR	NR	Multiple reported	Multiple reported	Rivastigmine patch	198 (33 %)	73.9 (8.0)
							Rivastigmine oral	102 (34 %)	72.9 (8.2)
							Placebo	101 (33%)	73.8 (7.5)
Hager 2014	YODA - Janssen	Mild-Moderate	NR	NR	NR	Multiple reported	Galantamine	354 (34%)	73 (8.9)
							Placebo	367 (36%)	73 (8.7)
Rockwood 2001	YODA - Janssen	Mild-Moderate	NR	NR	NR	Multiple reported	Galantamine	113 (43%)	75 (7.3)
							Placebo	58 (46%)	75 (7.6)
Cummings 2004	YODA - Janssen	NR	NR	NR	Multiple reported	Multiple reported	Galantamine	245 (35%)	76.9 (7.8)
							Placebo	108 (38%)	77.2 (7.9)
Burns 2009	YODA - Janssen	Severe	NR	NR	Multiple reported	Multiple reported	Galantamine	42 (20%)	84.0 (6.5)
							Placebo	39 (19%)	83.8 (6.7)
Gault 2015	AbbVie	Mild-Moderate	NR	NR	NR	Multiple reported	Donepezil	37 (54%)	72.4 (8.4)
							Placebo	26 (38%)	73.6 (8.2)
Haig 2014	AbbVie	Mild-Moderate	NR	NR	Multiple reported	Multiple reported	Donepezil	24 (40%)	70 (8.3)
							Placebo	24 (38%)	70 (7.8)
Bakchine 2008	Lundbeck	Mild-Moderate	NR	NR	NR	Multiple reported	Memantine	112 (35%)	74 (7.4)
							Placebo	61 (40%)	73 (6.9)
Herrman 2013	Lundbeck	69 (48%)	NR	NR	NR	Multiple reported	Memantine	77 (42%)	75 (7.9)
							Placebo	77 (41%)	75 (6.9)
Wilkinson 2012	Lundbeck	NR	NR	NR	NR	Multiple reported	Memantine	50 (38%)	74 (8.8)
							Placebo	69 (48%)	74 (7.8)

Additional characteristics of studies with shared IPD

Study	Patients experiencing at least one SAE	Missing data in SAE outcome	Baseline MMSE, mean (SD)	Final MMSE, mean (SD)	Change score, mean (SD)	Missing data in MMSE outcome	Total number of patients	Reasons for dropouts as indicated in the provided IPD	Time taken for the 1st SAE
Black 2007	21	0 (0%)	7.5 (3.3)	8.2 (5.2)	0.63 (3.1)	27 (15%)	176 (51%)	• intercurrent illness (1 [2%] – donepezil = 1; placebo = 0),<br>• request of patient or investigator (4 [7%] –	617 days (range [110, 1292])

For peer review only - http://bmjopen.bmj.com/site/about/guidelines.xhtml

	25	0 (0%)	7.4 (3.6)	7.6 (4.8)	-0.15 (3.5)	27 (16%)	167 (49%)	donepezil = 3; placebo = 1), • patient entered nursing home/facility (5 [9%] – donepezil = 1; placebo =) 4, • due to adverse experience (30 [56%] – donepezil = 15; placebo = 15), and • other (14 [26%] – donepezil = 7; placebo = 7)	691 days (range [78, 1475]).
Gold 2010	6	0 (0%)	20 (3.7)	21 (4.6)	1.11 (2.3)	18 (32%)	56 (34%)	• Adverse Event (16 [39%] – donepezil = 9; placebo = 7), • Lost to Follow-Up (4 [10%] – donepezil = 3; placebo = 1), • Non-compliance (6 [15%] – donepezil = 2; placebo = 4), • Subject decided to withdraw (11 [26%] – donepezil = 4; placebo = 7)	349 days (range [48, 656])
	10	0 (0%)	20.1 (4.2)	20.4 (5.4)	0.08 (2.7)	23 (22%)	107 (66%)		492 days (range [95, 780])
Winblad 2007	83	0 (0%)	16.6 (3.0)	17.7 (4.7)	1 (3.4)	74 (10%)	598 (50 %)	NR	NR
	37	0 (0%)	16.4 (3.1)	17.2 (4.6)	0.8 (3.2)	31 (12%)	297 (25 %)	NR	NR
	45	0 (0%)	16.4 (3.0)	16.4 (5.3)	-0.1 (3.6)	21 (7%)	302 (25 %)	NR	NR
Hager 2014	73	0 (0%)	19.0 (4.1)	17.81 (6.2)	-1.38 (4.3)	228 (22%)	1027 (50%)	NR	NR
	92	0 (0%)	19.0 (4.0)	16.99 (6.3)	-2.15 (4.4)	236 (23%)	1022 (50%)	NR	NR
Rockwood 2001	27	0 (0%)	23.2 (5.2)	NR	NR	NR	261 (68%)	NR	NR
	5	0 (0%)	22.9 (5.0)	NR	NR	NR	125 (32%)	NR	NR
Cummings 2004	23	0 (0%)	20.7 (4.9)	NR	NR	NR	692 (71%)	NR	NR
	81	0 (0%)	20.6 (4.9)	NR	NR	NR	286 (29%)	NR	NR
Burns 2009	62	0 (0%)	NR	9.2 (4.5)†	NR	NR	211 (51%)	NR	NR
	75	0 (0%)	NR	9.6 (4.9)†	NR	NR	204 (49%)	NR	NR
Gault 2015	5	0 (0%)	19.2 (4.1)	20.7 (5.1)	1.5 (2.6)	48 (71%)	68 (50%)	NR	305 days (range [224, 377])
	3	0 (0%)	18.8 (4)	18.9 (4.8)	0.1 (2.4)	45 (66%)	68 (50%)	NR	239 days (range [206, 295])
Haig 2014	2	0 (0%)	17.9 (4.2)	19.7 (3.9)	1.2 (2.8)	41 (68%)	60 (49%)	NR	286 days (range N/A – a single date was provided)
	1	0 (0%)	17.8 (3.8)	19.9 (4.2)	1.8 (1.8)	47 (75%)	63 (51%)	NR	270 days (range [161, 379]).
Bakchine 2008	33	0 (0%)	18.7 (3.3)	NR	NR	NR	318 (68%)	NR	NR
	9	0 (0%)	18.9 (3.2)	NR	NR	NR	152 (32%)	NR	NR
Herrman 2013	18	0 (0%)	11.9 (3.1)	11.3 (4.9)	-0.76 (3.4)	31 (8%)	182 (49%)	NR	NR
	11	0 (0%)	11.8 (2.9)	11.1 (4.7)	-0.68 (3.2)	32 (9%)	187 (51%)	NR	NR

For peer review only

For peer review only - http://bmjopen.bmj.com/site/about/guidelines.xhtml

Wilkinson 2012	17	0 (0%)	16.7 (2.5)	16.4 (5.2)	-0.46 (3.9)	30 (11%)	133 (48%)	NR	NR
	20	0 (0%)	17.1 (2.4)	16.4 (5.6)	-0.69 (4.0)	30 (11%)	144 (52%)	NR	NR

* According to publication
† The MMSE final value comes from visit 8 (last available visit in IPD). MMSE was not reported in study publication
Abbreviations: AD, Alzheimer's Dementia; IPD, individual patient data; MMSE, Mini-Mental State Examination; NR, not reported; N/A, not applicable; SAE, serious adverse event

For peer review only

For peer review only - http://bmjopen.bmj.com/site/about/guidelines.xhtml

Appendix 7: Comparison of studies with shared IPD with (a) all remaining studies and (b) studies for which sponsors claimed unavailable IPD. AD: aggregate data; IPD: individual patient data

a. Comparison of studies with shared IPD with all remaining studies (irrespective type of sponsor)

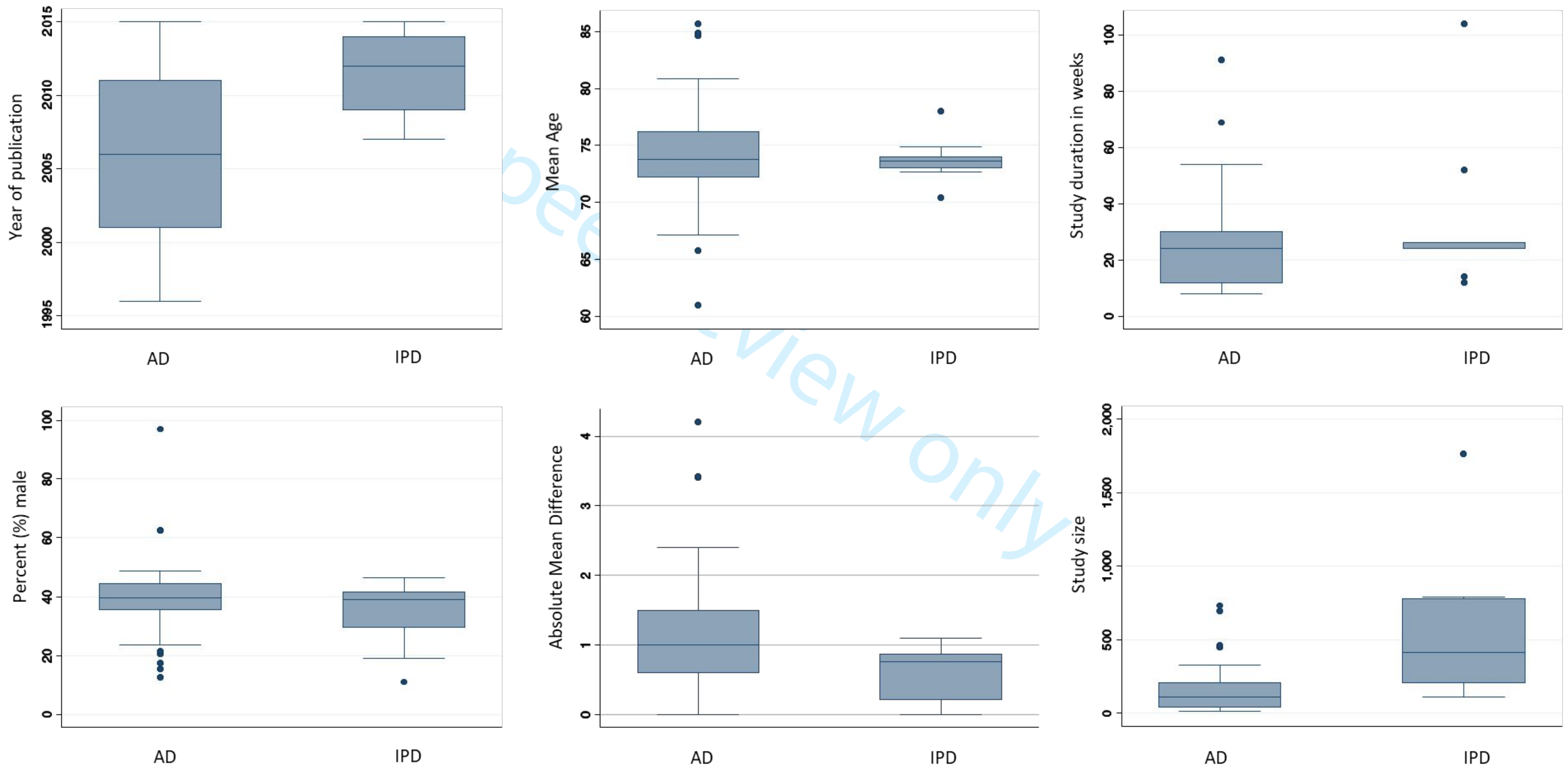

For peer review only - http://bmjopen.bmj.com/site/about/guidelines.xhtml

b. Comparison of studies with available and unavailable IPD (industry-sponsored studies only)

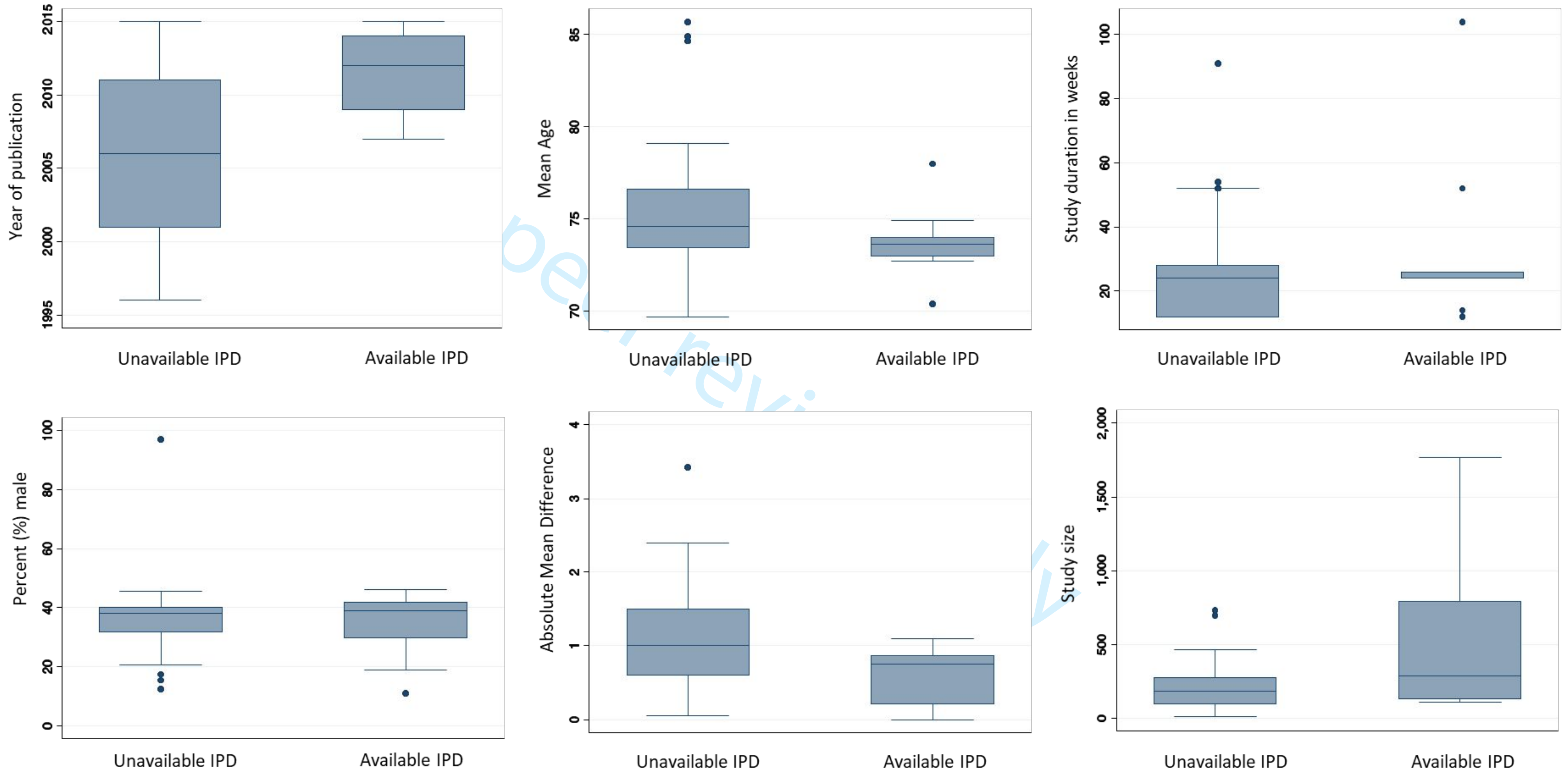

Appendix 8: Cochrane Risk-of-bias appraisal results (n = 80)

Study	1. Random sequence generation	2. Allocation concealment	3. Blinding of participants and personnel	4. Blinding of outcome assessment	5. Incomplete outcome data	6. Selective reporting	7. Other bias
Agid, 1998	Low	High	Low	Unclear	High	Unclear	High
Ancoli-Israel, 2005	Unclear	Unclear	Unclear	Unclear	High	Unclear	High
Andersen, 2012	Unclear	Low	Low	Low	High	Low	Low
Araki, 2014	Low	Unclear	Unclear	Unclear	High	Unclear	Unclear
Bakchine, 2008	Low	Low	Low	Low	Low	High	High
Black, 2007	Low	Low	Low	Low	Low	Unclear	High
Blesa Gonzalez, 2011	Unclear	Unclear	High	Unclear	High	Low	High
Burns, 1999	Unclear	Unclear	Unclear	Unclear	High	Unclear	High
Burns, 2009	Low	Low	Low	Low	Low	Unclear	High
Burns, 2011	Low	Unclear	Low	Low	High	Unclear	Unclear
Choi, 2011	Unclear	Unclear	High	High	High	Low	Low
Corey-Bloom, 1998	Low	Low	Low	Low	High	Unclear	High
Cretu, 2008	Unclear	Unclear	Unclear	Low	Unclear	Unclear	Unclear
Dysken, 2014	Low	Low	Low	Unclear	Low	Low	Low
Farlow, 2013	Low	Unclear	Low	Low	High	Unclear	High
Feldman, 2001	Low	Unclear	Low	Low	High	Unclear	High
Feldman, 2007	Low	Low	Low	Low	High	Unclear	High
Fox, 2012	Low	Low	High	Low	High	High	Unclear
Frolich, 2011	Unclear	Unclear	Low	Low	High	Low	High
Fuschillo, 2001	Unclear	Unclear	Unclear	Unclear	Low	Low	Unclear
Gault, 2015	Low	Low	Low	Unclear	Low	Low	High
Gold, 2010	Low	Unclear	Low	Low	High	Low	High
Greenberg, 2000	Low	Low	Low	Unclear	High	Low	Low
Grossberg, 2013	Low	Low	Low	Low	High	Low	High
Hager K, 2014	Low	Low	Low	Low	High	High	High
Haig, 2014	Low	Low	Low	Low	High	Low	High
Hernández, 2007	Low	Low	Low	Low	Unclear	Low	Low
Herrmann, 2013	Low	Low	Low	Low	High	Low	High
Holmes, 2004	Low	Unclear	Low	Low	High	Low	High
Homma, 1998	Low	Low	Low	Low	Low	Unclear	High
Homma, 2008	Low	Low	Low	Low	High	Unclear	Unclear
Hong, 2006	Unclear	Unclear	Unclear	Unclear	Low	Unclear	Unclear
Howard, 2007	Low	Low	Low	Low	Low	Unclear	Low
Howard, 2012	Low	Low	Low	Low	High	Low	Low
Hu, 2006	Unclear	Unclear	Unclear	Unclear	Low	Unclear	Unclear
Johannsen, 2006	Unclear	Unclear	Low	Low	Low	Unclear	High
Jones, 2004	Low	Unclear	Unclear	Low	Low	Unclear	High
Kadir, 2008	Unclear	Unclear	Unclear	Unclear	High	Unclear	High
Kano, 2013	Unclear	Unclear	Unclear	Unclear	Low	Unclear	Unclear
Karaman, 2005	Low	Unclear	Low	Low	Unclear	Unclear	Unclear
Likitjaroen, 2012	Low	Low	Low	Unclear	High	High	Unclear
Lorenzi, 2011	Unclear	Unclear	Unclear	Unclear	Unclear	Unclear	High
Maher-Edwards, 2011	Low	Unclear	Unclear	Unclear	High	Unclear	High
Marek, 2014	Low	Low	Low	Low	High	Low	High
Mazza, 2006	Low	Unclear	Low	Low	High	Unclear	Unclear
Mohs, 2001	Low	Low	Low	Low	High	Unclear	High
Moretti, 2014	Unclear	Unclear	Unclear	Unclear	Low	Unclear	Low
Mowla, 2007	Low	Unclear	Low	Unclear	High	Unclear	Unclear
Nakamura, 2011	Unclear	Low	Low	Low	Low	Low	High
Nakano, 2001	Unclear	Unclear	Unclear	Unclear	Unclear	Unclear	Unclear
Nordberg, 2009	Unclear	Unclear	High	High	Unclear	Unclear	High
Pakdaman H, 2015	Low	Unclear	High	High	High	Unclear	Unclear
Peng, 2005	Unclear	Unclear	Unclear	Unclear	Low	Unclear	Unclear
Peskind, 2006	Low	Low	Low	Unclear	Low	Unclear	High
Peters, 2015	Unclear	Unclear	Low	Low	High	Low	Low
Reisberg, 2003	Low	Unclear	Low	Unclear	High	Low	Unclear
Rockwood, 2001	Low	Low	Low	Low	Unclear	Low	High
Rockwood, 2006	Low	Low	Low	Low	Low	Unclear	Unclear
Rogers, 1996	Unclear	Unclear	Low	Unclear	Low	Unclear	Unclear
Rogers, 1998	Unclear	Unclear	Low	Low	Low	Unclear	High
Rogers, 1998	Low	Unclear	Low	Unclear	High	Unclear	High
Saxton, 2012	Low	Low	Low	Low	Low	Low	High
Scarpini, 2011	Low	Low	Low	Unclear	High	Unclear	High
Schmidt, 2008	Low	Low	Low	Low	High	Unclear	High
Seltzer, 2004	Low	Unclear	Unclear	Unclear	Unclear	Unclear	High

For peer review only - http://bmjopen.bmj.com/site/about/guidelines.xhtml

Shao, 2015	Unclear	Unclear	Unclear	Unclear	Low	Unclear	Unclear
Shimizu, 2015	Low	Unclear	High	Low	High	Unclear	Unclear
Sole-Padulles, 2013	Unclear	Unclear	Unclear	Unclear	Low	Unclear	Unclear
Tariot, 2000	Low	Unclear	Low	Low	High	Low	High
Tariot, 2001	Low	Low	Low	Low	Unclear	Unclear	High
Thomas, 2001	Low	Unclear	Unclear	Unclear	Unclear	Unclear	Unclear
Wilcock, 2003	Unclear	Unclear	Unclear	Unclear	Unclear	Unclear	Unclear
Wilkinson, 2001	Low	Low	Low	Low	High	Unclear	High
Wilkinson, 2002	Low	Low	Low	Low	High	Unclear	High
Wilkinson, 2012	Low	High	Low	Low	High	Low	High
Winblad, 2001	Low	Unclear	Unclear	Low	High	Unclear	High
Winblad, 2006	Low	Low	Low	Low	High	Low	High
Winblad, 2007	Low	Low	Low	Low	High	Unclear	High
Yi, 2005	Unclear	Unclear	Unclear	Unclear	Low	Unclear	Unclear
Zhang, 2012	Unclear	Unclear	Unclear	Unclear	High	Unclear	High

For peer review only

For peer review only - http://bmjopen.bmj.com/site/about/guidelines.xhtml

Appendix 9: Overall risk of bias for studies with shared IPD against (a) all remaining studies and (b) studies for which sponsors claimed unavailable IPD. AD: aggregate data; IPD: individual patient data

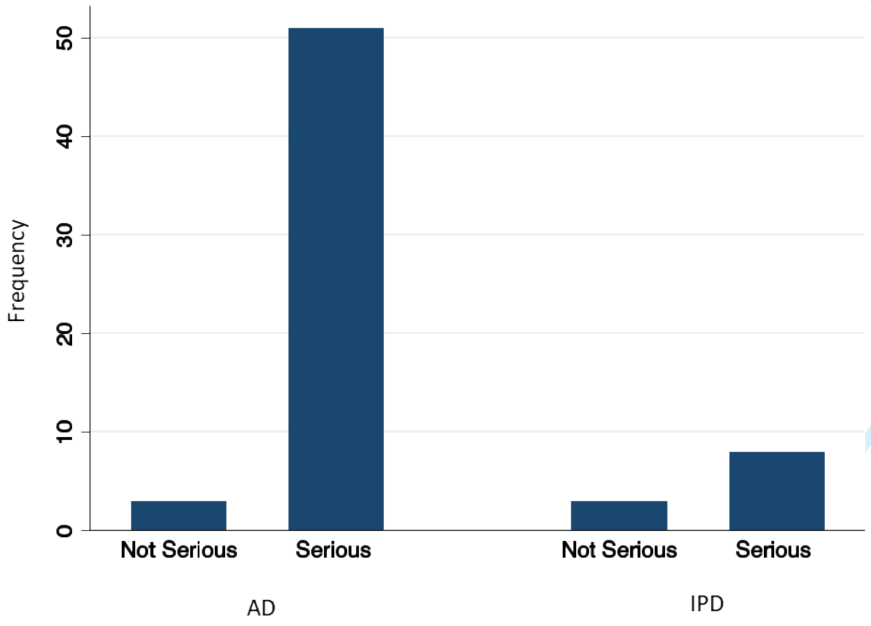

a. Comparison of studies with shared IPD with all remaining studies (irrespective type of sponsor)

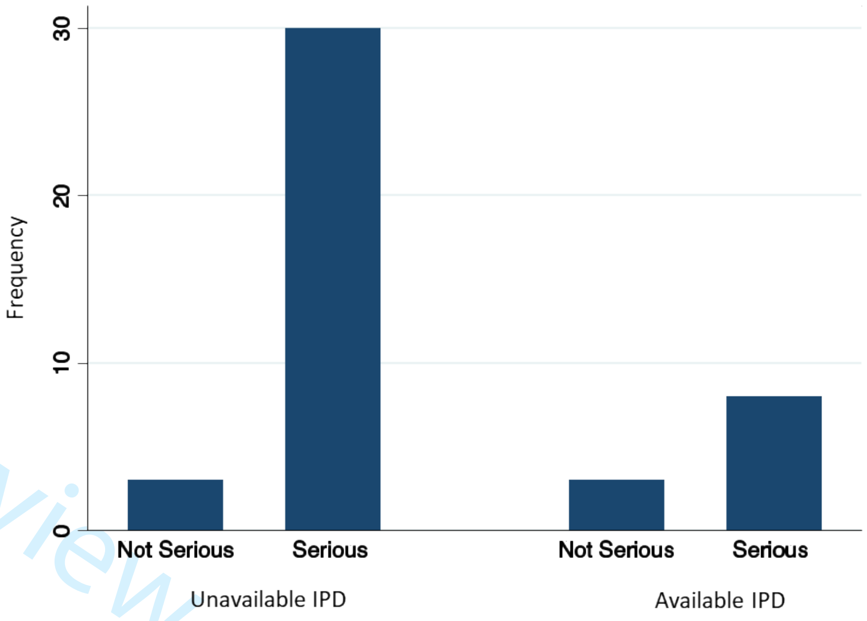

b. Comparison of studies with available and unavailable IPD (industry-sponsored studies only)

For peer review only - http://bmjopen.bmj.com/site/about/guidelines.xhtml

Appendix 10: Study-specific effect sizes calculated from shared IPD and published data. IPD: individual patient data

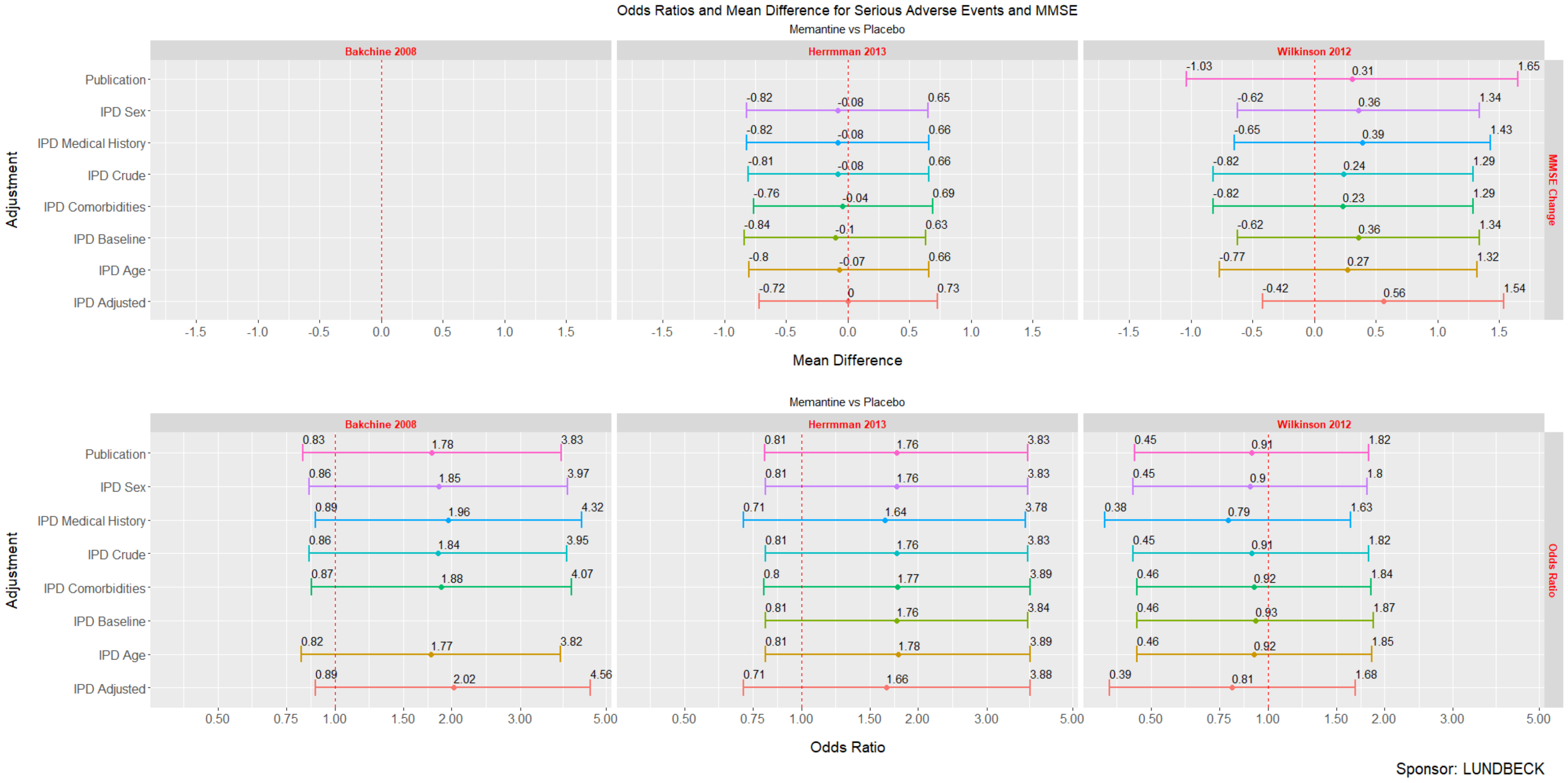

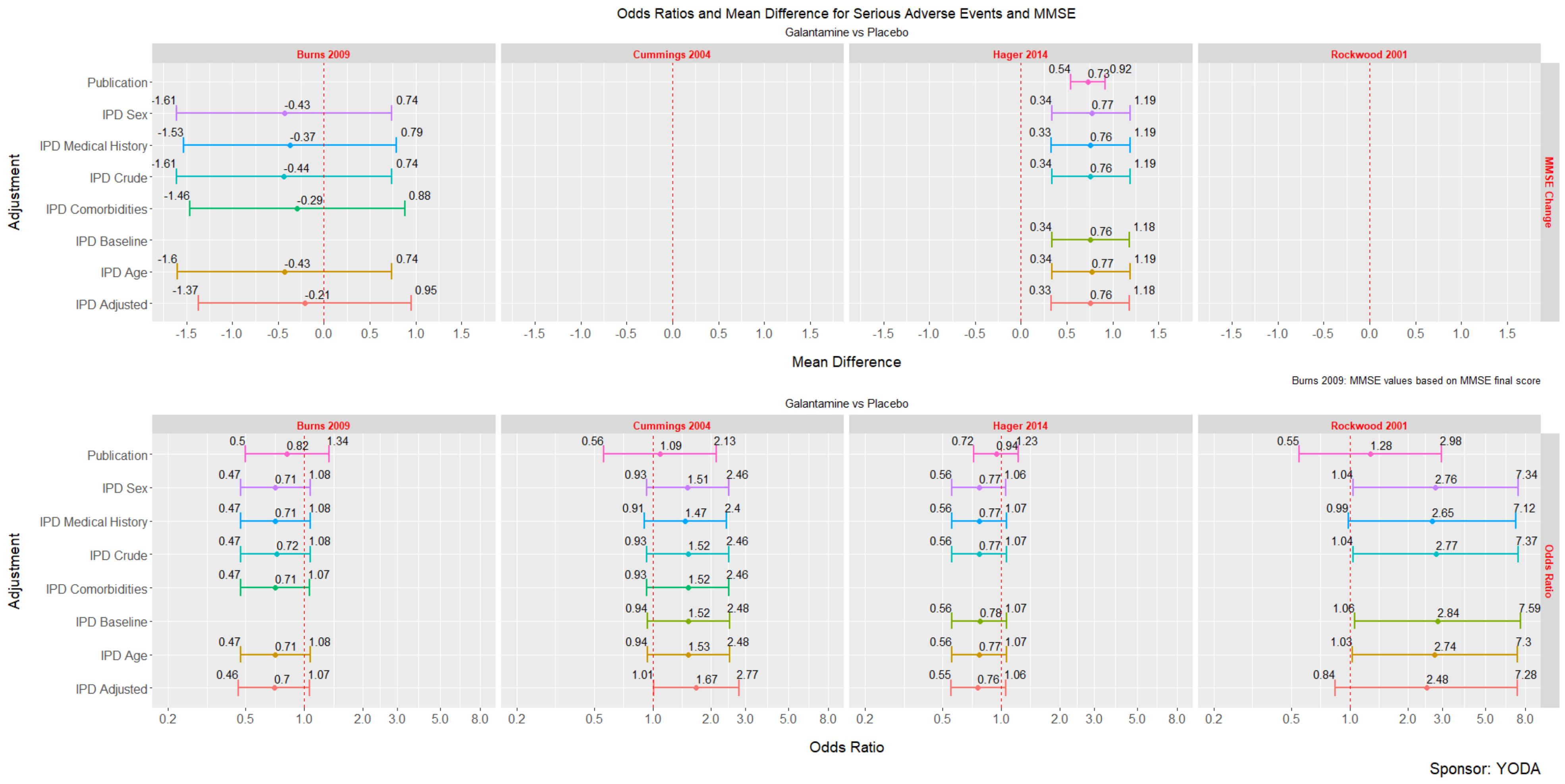
Odds Ratios and Mean Difference for Serious Adverse Events and MMSE
Galantamine vs Placebo
Burns 2009
Cummings 2004
Hager 2014
Rockwood 2001
MMSE Change
Adjustment
Publication
IPD Sex
IPD Medical History
IPD Crude
IPD Comorbidities
IPD Baseline
IPD Age
IPD Adjusted
Mean Difference
Burns 2009: MMSE values based on MMSE final score
Odds Ratio
Sponsor: YODA

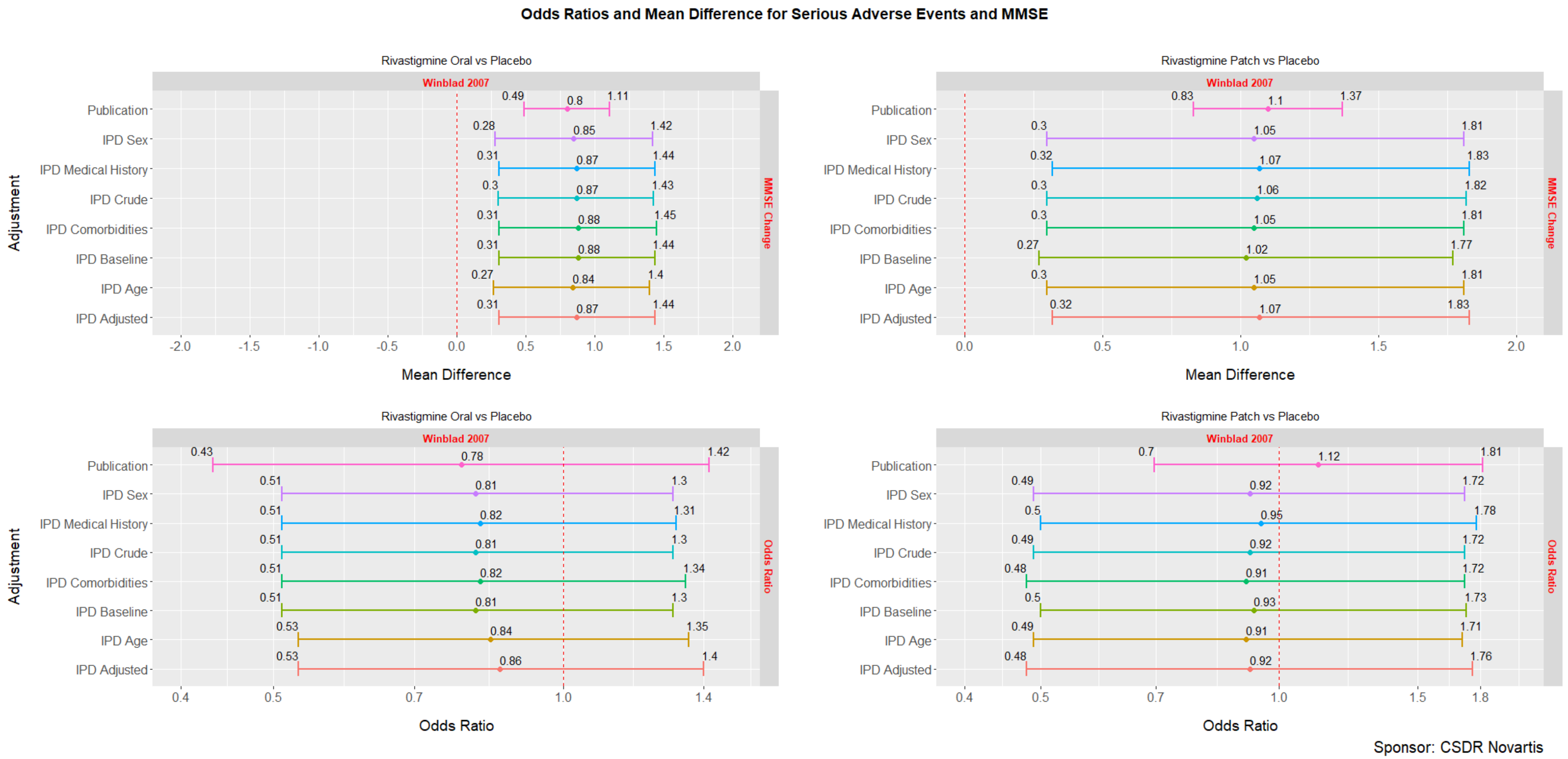
Odds Ratios and Mean Difference for Serious Adverse Events and MMSE
Rivastigmine Oral vs Placebo
Rivastigmine Patch vs Placebo
Winblad 2007
MMSE Change
Odds Ratio
Adjustment
Publication
IPD Sex
IPD Medical History
IPD Crude
IPD Comorbidities
IPD Baseline
IPD Age
IPD Adjusted
Mean Difference
Odds Ratio
Sponsor: CSDR Novartis

33

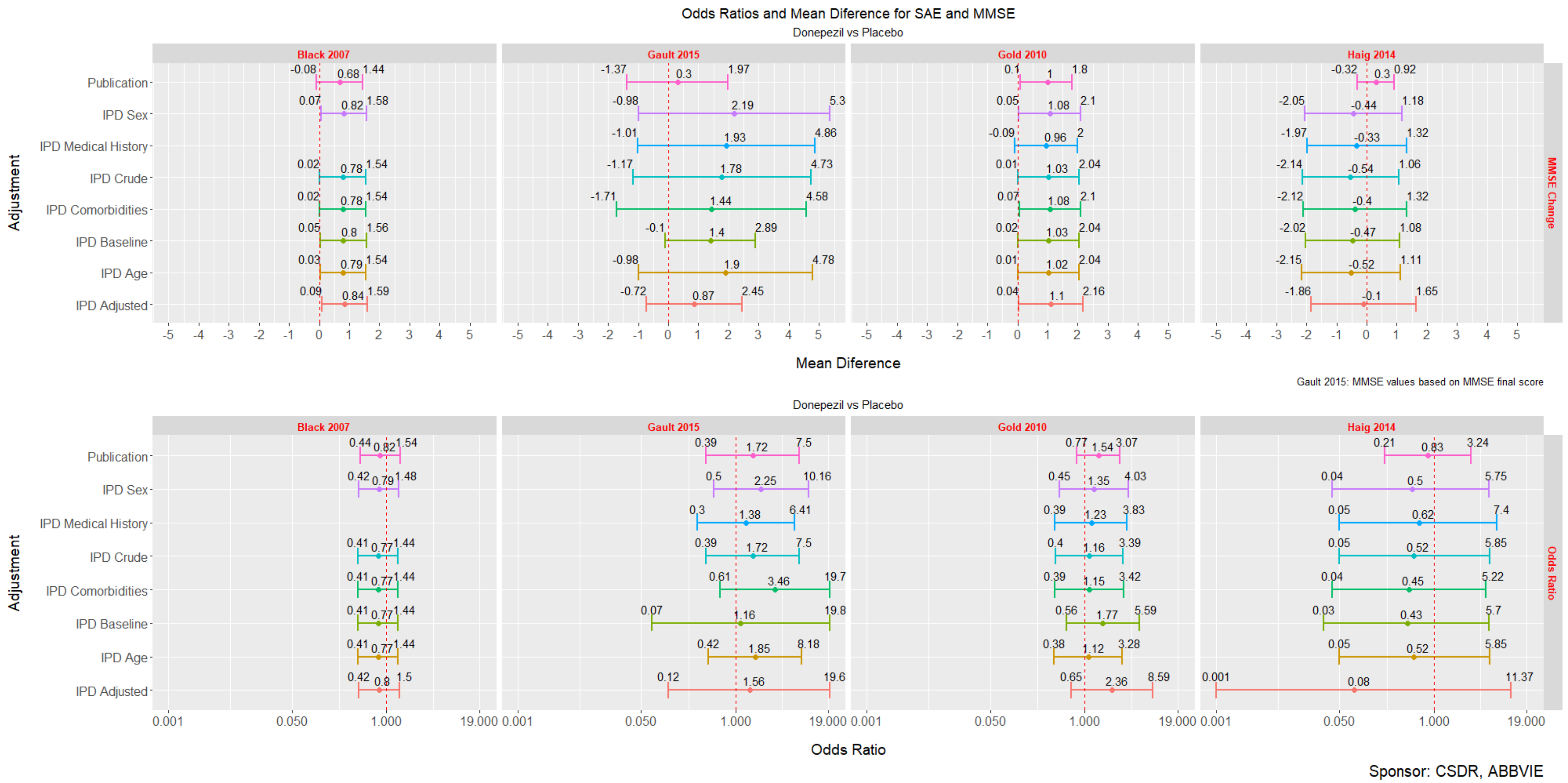

CSDR includes studies sponsored by GlaxoSmithKline, Eisai, Novartis, whereas YODA includes studies sponsored by Janssen

We also calculated the odds ratio for patients experiencing at least one SAE excluding missing participants as shown in the MMSE outcome: Gold 2010: OR 2.78, 95% CI: 0.63-12.25; Black 2007: OR 1.19, 95% CI: 0.08-17.96; Winbland 2007: rivastigmine oral, OR 1.28, 95% CI: 0.09-18.16, rivastigmine patch, OR 0.81, 95% CI: 0.02-33.59; Wilkinson 2012: OR 0.84, 95% CI: 0.38-1.86; Herrmman 2013: OR 1.70, 95% CI: 0.71-4.08; Bachine 2008: OR 1.83, 95% CI: 0.77-4.32.

We were unable to assess this for studies obtained through YODA and AbbVie, since at the time of this assessement we did not have access to these data.

Abbreviations: IPD sex, regression analysis adjusting for sex; IPD medical history, regression analysis adjusting for medical history; IPD crude, analysis with no adjustments; IPD comorbidities, regression analysis adjusting for comorbidities; IPD baseline, regression analysis adjusting for MMSE baseline; IPD age, regression analysis adjusting for age; IPD adjusted, regression analysis adjusting for all available variables (we only considered those that we initially requested from sponsor)

For peer review only

For peer review only - http://bmjopen.bmj.com/site/about/guidelines.xhtml

Appendix 11: Correlation between participant age and dropout in studies with IPD. IPD: individual patient data

	Study*	Correlation	P-Value
**CSDR**	Black 2007 (EISAI)	0.079	0.147
	Gold 2010 (GSK)	0.141	0.072
	Winblad 2007 (Novartis)	0.016	0.584
**Lundbeck**	Wilkinson 2012	0.066	0.273
	Herrmman 2013	0.124	0.017

* We were unable to assess this correlation for studies obtained through YODA and AbbVie, since at the time of this assessment we did not have access to these data

For peer review only

Appendix 12: Comparison Adjusted Funnel plot (all treatments vs placebo)

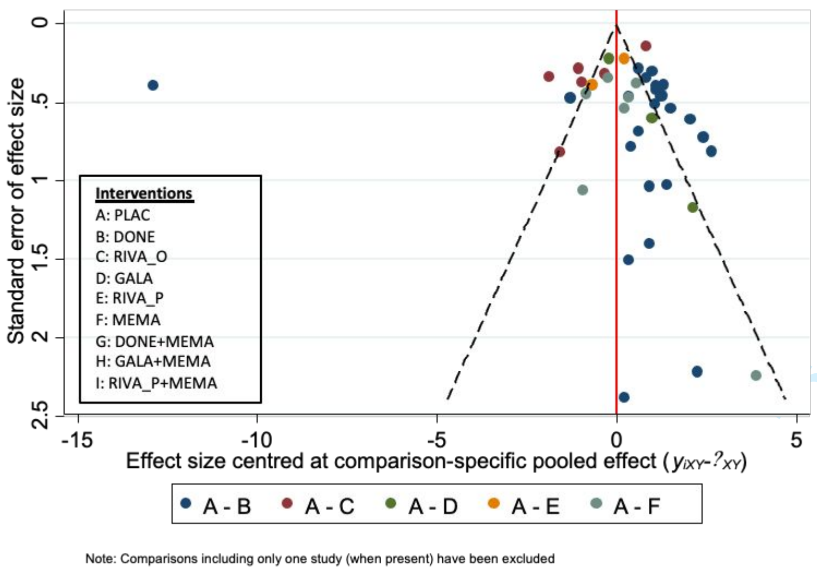

MMSE

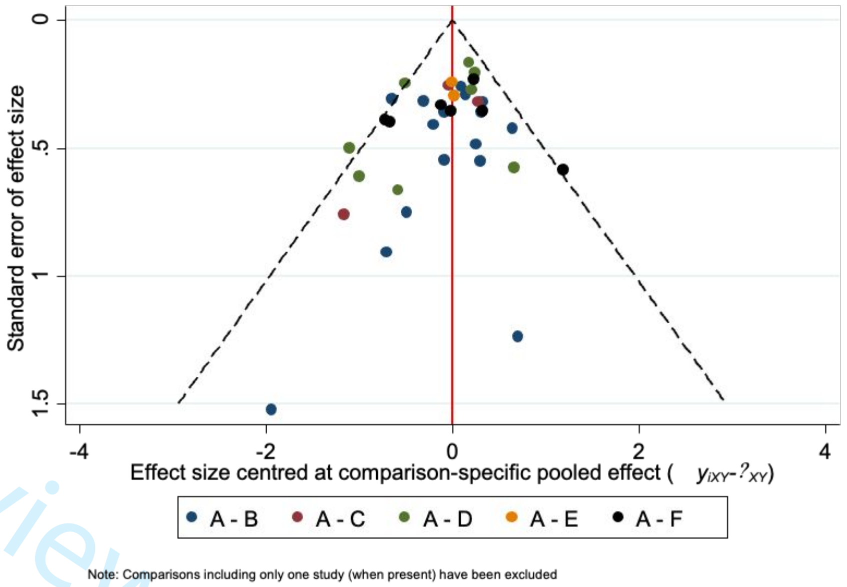

Serious Adverse Events

Appendix 13: Distribution of potential effect modifiers per treatment comparison and outcome

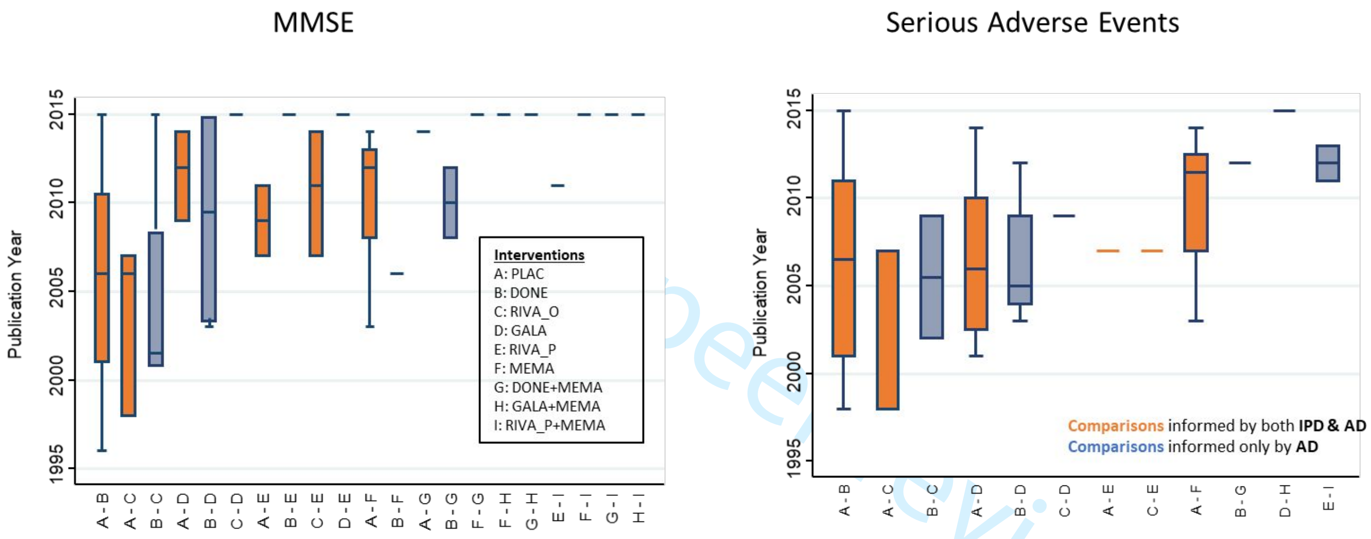

a. Publication year

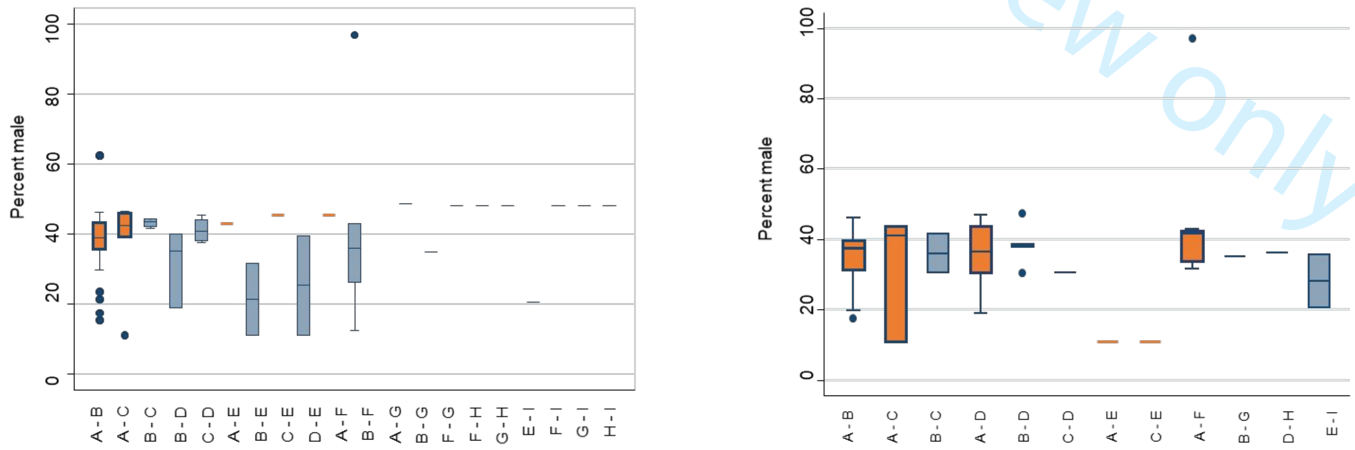

b. Percentage male

peer review only

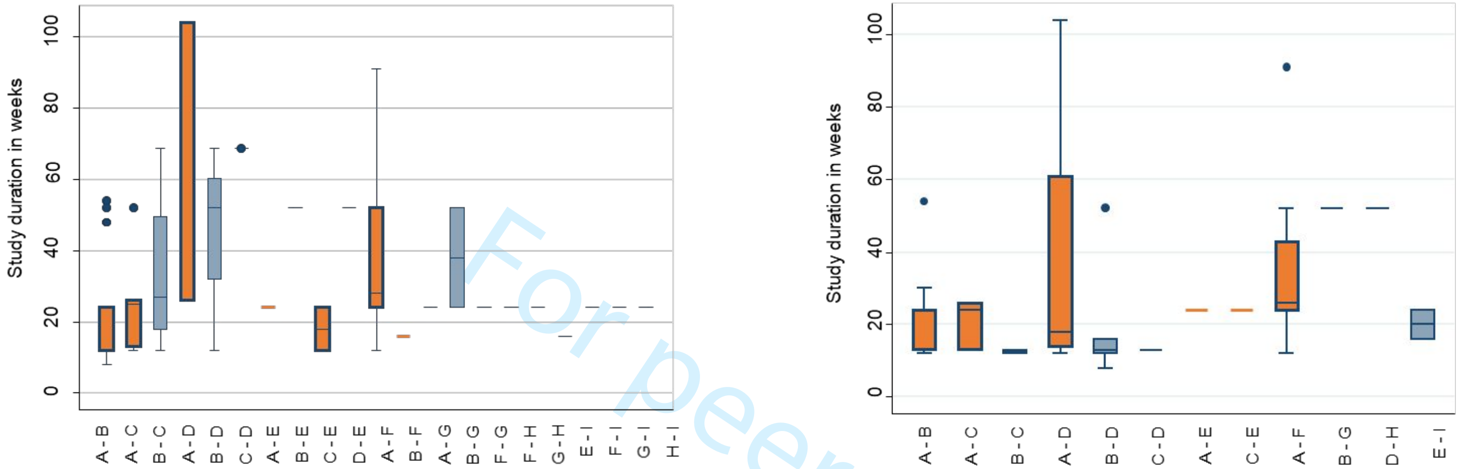

c. Study duration

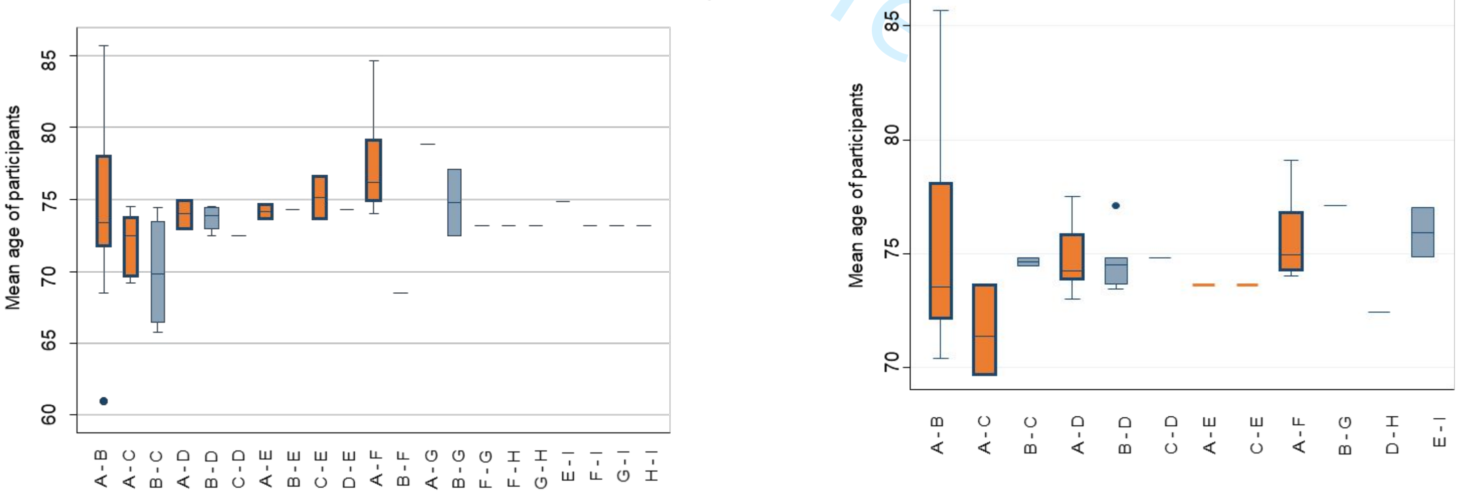

d. Mean participant age

For peer review only - http://bmjopen.bmj.com/site/about/guidelines.xhtml

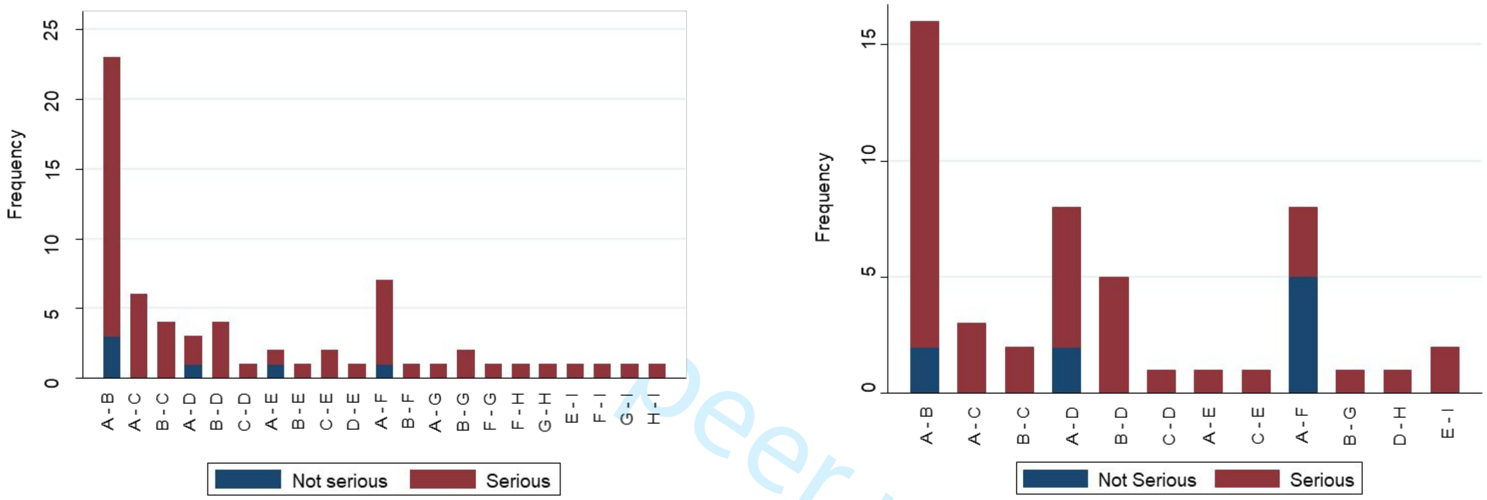

e. Overall Risk of Bias

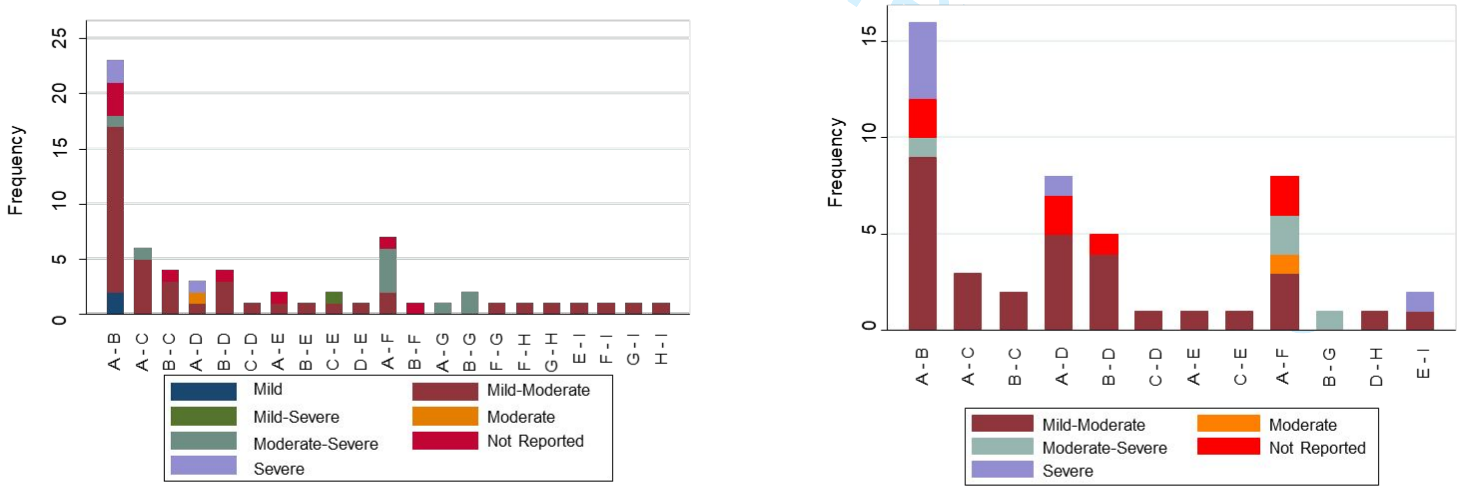

f. Alzheimer's Dementia Severity

For peer review only - http://bmjopen.bmj.com/site/about/guidelines.xhtml

Appendix 14: Consistency Assessment – Loop-specific approach (using adjusted treatment effects)

MMSE

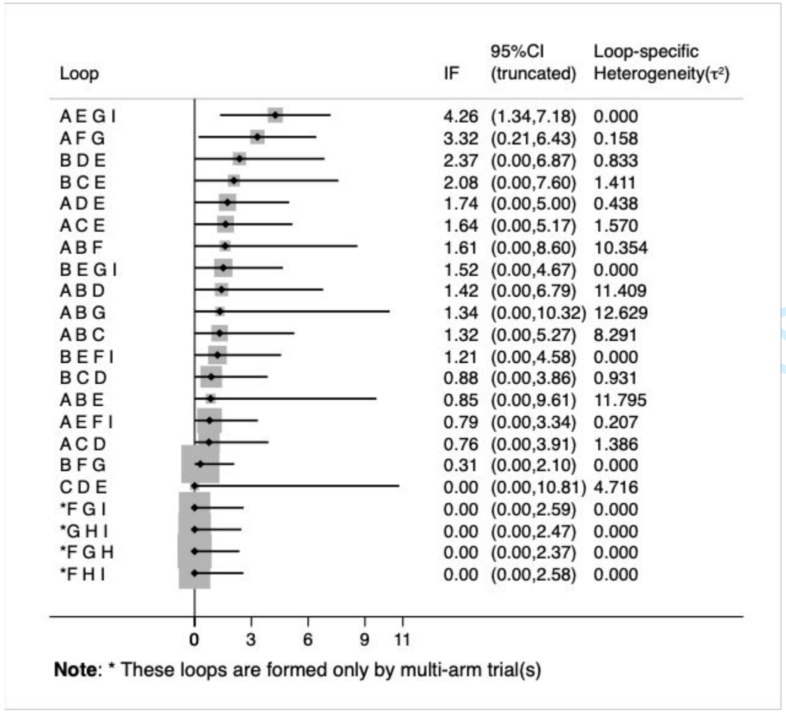

Design-by-treatment interaction model:
χ^2 statistic: 4.36, 13 degrees of freedom, P value: 0.987, between-study variance: 7.34. I^2 statistic=96%

Serious Adverse Events

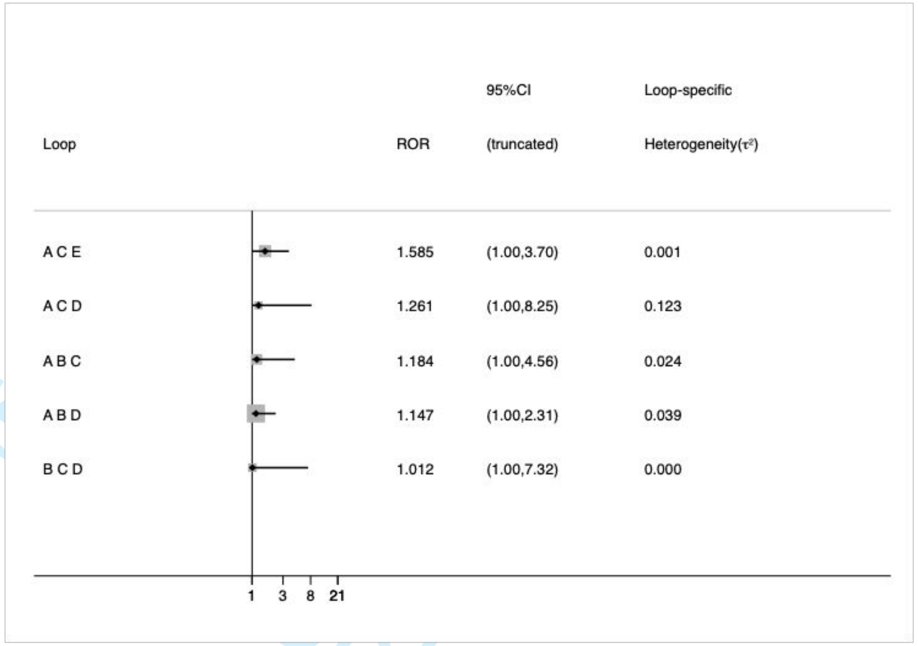

Design-by-treatment interaction model:
χ^2 statistic: 3.57, 6 degrees of freedom, P value: 0.735, between-study variance: 0.06. I^2 statistic=22%

For peer review only - http://bmjopen.bmj.com/site/about/guidelines.xhtml

Appendix 15: Network and standard meta-analysis results

Treatment Comparison	NMA estimate	95% CI	95% PI	P-score	MA estimate	95% CI	95% PI	#studies
**Mini-Mental State Examination (MMSE)*†**								
Donepezil vs Placebo	1.41	0.51 to 2.32	-3.48 to 6.31	0.59	1.65	0.16 to 3.14	-6.02 to 9.32	24
Rivastigmine oral vs Placebo	0.69	-0.79 to 2.18	-4.35 to 5.74	0.36	0.60	-0.43 to 1.62	-3.07 to 4.26	6
Galantamine vs Placebo	0.41	-1.44 to 2.26	-4.76 to 5.58	0.28	0.04	-1.09 to 1.17	-12.39 to 12.47	3
Rivastigmine transdermal vs Placebo	2.11	-0.04 to 4.26	-3.18 to 7.40	0.72	0.56	-0.33 to 1.45	--	2
Memantine vs Placebo	0.67	-0.99 to 2.34	-4.43 to 5.78	0.35	0.52	0.03 to 1.01	-0.69 to 1.73	7
Donepezil + Memantine vs Placebo	2.57	0.07 to 5.07	-2.88 to 8.02	0.80	4.21	1.94 to 6.48	--	1
Galantamine + Memantine vs Placebo	2.24	-2.13 to 6.61	-4.33 to 8.81	0.66				
Rivastigmine transdermal + Memantine vs Placebo	1.79	-1.70 to 5.27	-4.20 to 7.78	0.60				
Placebo (reference)				0.14				
Rivastigmine transdermal vs Rivastigmine oral	1.41	-0.80 to 3.62	-3.90 to 6.73		2.26	-0.48 to 4.99	-30.56 to 35.07	3
Rivastigmine oral vs Donepezil	-0.72	-2.28 to 0.84	-5.79 to 4.35		0.16	-0.57 to 0.90	-1.45 to 1.77	4
Galantamine vs Rivastigmine oral	-0.29	-2.48 to 1.91	-5.60 to 5.02		0.06	-1.05 to 1.17		1
Rivastigmine transdermal vs Donepezil	0.69	-1.52 to 2.91	-4.62 to 6.01		-0.20	-2.78 to 2.38		1
Rivastigmine transdermal vs Galantamine	1.70	-0.93 to 4.33	-3.81 to 7.21		2.20	-0.19 to 4.59		1
Rivastigmine transdermal + Memantine vs Rivastigmine transdermal	-0.32	-3.82 to 3.18	-6.32 to 5.68		-0.40	-1.40 to 0.60		1
Memantine vs Donepezil	-0.74	-2.56 to 1.08	-5.90 to 4.42		0.20	0.88 to 1.28		1
Donepezil + Memantine vs Donepezil	1.15	-1.33 to 3.64	-4.29 to 6.59		0.88	0.64 to 1.11		2
Galantamine vs Donepezil	-1.01	-2.86 to 0.84	-6.18 to 4.16		-0.35	-1.52 to 0.83	-5.31 to 4.62	4
Donepezil + Memantine vs Memantine	1.89	-0.88 to 4.67	-3.69 to 7.48		0.37	-1.04 to 1.78		1
Galantamine + Memantine vs Memantine	1.57	-2.78 to 5.92	-4.98 to 8.12		0.82	-0.58 to 2.22		1

For peer review only

For peer review only - http://bmjopen.bmj.com/site/about/guidelines.xhtml

Rivastigmine transdermal + Memantine vs Memantine	1.12	-2.47 to 4.70	-4.93 to 7.16		0.41	-1.17 to 1.99		1
Galantamine + Memantine vs Donepezil + Memantine	-0.33	-4.72 to 4.06	-6.91 to 6.23		0.45	-0.85 to 1.75		1
Rivastigmine transdermal + Memantine vs Donepezil + Memantine	-0.78	-4.53 to 2.97	-6.93 to 5.38		0.04	-1.45 to 1.53		1
Rivastigmine transdermal + Memantine vs Galantamine + Memantine	-0.45	-5.05 to 4.14	-7.18 to 6.28		-0.41	-1.89 to 1.07		1
Common within-network between-study variance τ² = 5.75, I² = 96% (96%, 97%)								
Design-by-treatment interaction model for inconsistency χ² (d.f., P-value, τ²): 4.36 (13, 0.987, 7.35)								
**Serious Adverse Events (SAEs)*‡**								
Donepezil vs Placebo	1.08	0.87 to 1.35	0.67 to 1.75	0.30	1.07	0.88 to 1.31	0.84 to 1.37	16
Rivastigmine oral vs Placebo	1.26	0.82 to 1.94	0.69 to 2.33	0.16	1.26	0.75 to 2.12	0.01 to 161.35	3
Galantamine vs Placebo	0.95	0.74 to 1.22	0.58 to 1.55	0.53	1.02	0.71 to 1.46	0.38 to 2.77	8
Rivastigmine transdermal vs Placebo	0.90	0.58 to 1.42	0.48 to 1.69	0.57	0.86	0.53 to 1.40		1
Memantine vs Placebo	0.88	0.64 to 1.20	0.52 to 1.49	0.63	0.87	0.63 to 1.20	0.38 to 1.99	8
Donepezil + Memantine vs Placebo	0.77	0.34 to 1.73	0.30 to 1.96	0.69				
Galantamine + Memantine vs Placebo	1.03	0.45 to 2.39	0.39 to 2.70	0.43				
Rivastigmine transdermal + Memantine vs Placebo	0.72	0.32 to 1.59	0.28 to 1.81	0.75				
Placebo (reference)				0.44				
Rivastigmine oral Donepezil vs	1.17	0.73 to 1.87	0.61 to 2.22		2.08	0.21 to 20.73		2
Galantamine vs Donepezil	0.88	0.64 to 1.19	0.52 to 1.49		0.79	0.46 to 1.39	0.32 to 1.96	5
Donepezil + Memantine vs Donepezil	0.71	0.33 to 1.55	0.29 to 1.76		0.71	0.37 to 1.38		1
Rivastigmine transdermal vs Rivastigmine oral	0.72	0.42 to 1.23	0.36 to 1.44		0.94	0.52 to 1.68		1
Rivastigmine transdermal + Memantine vs Rivastigmine transdermal	0.79	0.41 to 1.54	0.36 to 1.77		0.79	0.45 to 1.39		2
Galantamine vs Rivastigmine oral	0.75	0.46 to 1.22	0.39 to 1.45		0.63	0.15 to 2.64		1

For peer review only

Galantamine + Memantine vs Galantamine	1.09	0.49 to 2.42	0.43 to 2.75	1.09	0.55 to 2.17	1
Common within-network between-study variance τ2 = 0.04, I^2 = 22% (0%, 48%)						
Design-by-treatment interaction model for inconsistency χ^2 (d.f., P-value, τ^2): 3.57 (6, 0.735, 0.06)						

* Aggregate data and fully adjusted results from studies with available individual patient data were used in both meta-analysis and NMA. The mean difference effect size is presented for MMSE and the odds ratio for SAE.
† MMSE: Studies with available IPD included only available participants –to assess the missing data impact on the second stage (IMDoM) a separate analysis was applied
‡ SAE: Studies with available IPD included all randomized participants

For peer review only

Appendix 16: Network subgroup and meta-regression analysis results

Treatment Comparison	NMA estimate	95% CI	95%PI	P-score
**Mini-Mental State Examination (MMSE)†**				
**Mean Difference: Aggregate data and crude results from studies with available individual patient data**				
Donepezil vs Placebo	1.41	0.50 to 2.33	-3.51 to 6.34	0.59
Rivastigmine oral vs Placebo	0.69	-0.80 to 2.19	-4.38 to 5.76	0.36
Galantamine vs Placebo	0.37	-1.49 to 2.23	-4.82 to 5.57	0.28
Rivastigmine transdermal vs Placebo	2.10	-0.06 to 4.26	-3.22 to 7.42	0.72
Memantine vs Placebo	0.63	-1.05 to 2.30	-4.51 to 5.76	0.34
Donepezil + Memantine vs Placebo	2.56	0.04 to 5.07	-2.92 to 8.04	0.79
Galantamine + Memantine vs Placebo	2.22	-2.18 to 6.61	-4.39 to 8.82	0.66
Rivastigmine transdermal + Memantine vs Placebo	1.77	-1.73 to 5.27	-4.25 to 7.79	0.60
Placebo (reference)				0.14
Common within-network between-study variance τ^2 = 5.81, I^2 = 96% (96%, 97%)				
Design-by-treatment interaction model for inconsistency χ^2 *(d.f., P-value,* τ^2*)*: 4.42 (13, 0.986, 7.44)				
**Mean Difference: Aggregate data results**[**]				
Donepezil vs Placebo	1.55	0.41 to 2.68	-4.16 to 7.25	0.57
Rivastigmine oral vs Placebo	0.71	-1.10 to 2.52	-5.18 to 6.60	0.34
Galantamine vs Placebo	0.57	-1.98 to 3.12	-5.61 to 6.74	0.32
Rivastigmine transdermal vs Placebo	2.60	-0.20 to 5.40	-3.69 to 8.89	0.75
Memantine vs Placebo	0.82	-1.37 to 3.01	-5.21 to 6.84	0.37
Donepezil + Memantine vs Placebo	2.71	-0.17 to 5.60	-3.62 to 9.04	0.76
Galantamine + Memantine vs Placebo	2.44	-2.61 to 7.48	-5.19 to 10.07	0.65
Rivastigmine transdermal + Memantine vs Placebo	2.09	-1.98 to 6.15	-4.89 to 9.07	0.61
Placebo (reference)				0.15
Common within-network between-study variance τ^2 = 7.66, I2 = 97% (96%, 97%)				
Design-by-treatment interaction model for inconsistency χ^2 *(d.f., P-value,* τ^2*)*: 3.92 (11, 0.972, 8.76)				
**Mean Difference: Crude results from studies with available individual patient data**				
Donepezil vs Placebo	0.70	0.01 to 1.40	-0.67 to 2.07	0.65
Rivastigmine oral vs Placebo	0.87	-0.01 to 1.75	-0.70 to 2.44	0.73
Galantamine vs Placebo	0.45	-0.24 to 1.14	-0.91 to 1.82	0.48
Rivastigmine transdermal vs Placebo	1.06	0.04 to 2.08	-0.67 to 2.79	0.82
Memantine vs Placebo	0.05	-0.74 to 0.83	-1.42 to 1.51	0.20
Placebo (reference)				0.13
Common within-network between-study variance τ^2 = 0.12, I^2 = 29% (0%, 71%)				
Design-by-treatment interaction model for inconsistency χ^2 *(d.f., P-value,* τ^2*)*: N/A (no closed loops)				
**Mean Difference: Low Risk of Bias for Allocation Concealment***				
Donepezil vs Placebo	2.02	-0.24 to 4.28	-6.19 to 10.23	0.70
Rivastigmine oral vs Placebo	1.38	-2.27 to 5.02	-7.39 to 10.14	0.57
Galantamine vs Placebo	-0.31	-4.61 to 3.98	-9.42 to 8.79	0.31
Rivastigmine transdermal vs Placebo	0.82	-4.08 to 5.72	-8.63 to 10.27	0.48
Memantine vs Placebo	0.69	-3.01 to 4.39	-8.10 to 9.49	0.46
Donepezil + Memantine vs Placebo	2.88	-4.75 to 10.51	-8.48 to 14.23	0.69
Placebo (reference)				0.30
Common within-network between-study variance: τ^2 = 13.82, I^2 = 98% (98%, 99%)				
Design-by-treatment interaction model for inconsistency χ^2 *(d.f., P-value,* τ^2*)*: 0.13 (3, 0.99, 19.10)				
**Mean Difference: Low risk of bias for Incomplete Data***				
Donepezil vs Placebo	0.87	0.07 to 1.66	-1.67 to 3.40	0.61
Rivastigmine oral vs Placebo	-1.52	-4.41 to 1.37	-5.54 to 2.50	0.10
Galantamine vs Placebo	0.52	-0.94 to 1.99	-2.36 to 3.41	0.48
Rivastigmine transdermal vs Placebo	1.37	-0.64 to 3.38	-1.91 to 4.65	0.71
Memantine vs Placebo	0.57	-1.12 to 2.27	-2.47 to 3.62	0.48
Donepezil + Memantine vs Placebo	0.94	-2.11 to 4.00	-3.23 to 5.11	0.57
Galantamine + Memantine vs Placebo	1.39	-1.66 to 4.44	-2.77 to 5.56	0.70
Rivastigmine transdermal + Memantine vs Placebo	0.98	-2.15 to 4.12	-3.26 to 5.23	0.58
Placebo (reference)				0.27
Common within-network between-study variance: τ^2 = 1.16, I^2 = 79% (65%, 88%)				
Design-by-treatment interaction model for inconsistency χ^2 *(d.f., P-value,* τ^2*)*: 12.15 (3, 0.007, 0.863)				
**Mean Difference: Publicly-Sponsored Studies***				
Donepezil vs Placebo	6.57	-4.68 to 17.81	-129.61 to 142.74	0.71
Rivastigmine oral vs Placebo	1.40	-16.41 to 19.21	-161.58 to 164.38	0.44
Memantine vs Placebo	0.11	-17.65 to 17.87	-162.64 to 162.86	0.39
Rivastigmine transdermal + Memantine vs Placebo	5.83	-7.98 to 19.64	-139.93 to 151.59	0.65
Placebo (reference)				0.32

For peer review only - http://bmjopen.bmj.com/site/about/guidelines.xhtml

Common within-network between-study variance: τ^2 = 81.93, I^2 = 99% (99%, 100%)				
Design-by-treatment interaction model for inconsistency χ^2 *(d.f., P-value,* τ^2*)*: 0.05 (1, 0.815, 116.71)				
**Mean Difference: Industry-Sponsored Studies***				
Donepezil vs Placebo	0.98	0.69 to 1.27	0.10 to 1.86	0.85
Rivastigmine oral vs Placebo	0.82	0.35 to 1.29	-0.14 to 1.78	0.69
Galantamine vs Placebo	0.41	-0.15 to 0.96	-0.60 to 1.41	0.34
Rivastigmine transdermal vs Placebo	0.80	0.18 to 1.41	-0.25 to 1.84	0.67
Memantine vs Placebo	0.60	0.06 to 1.15	-0.39 to 1.60	0.50
Rivastigmine transdermal + Memantine vs Placebo	0.40	-1.02 to 1.81	-1.29 to 2.08	0.39
Placebo (reference)				0.06
Common within-network between-study variance: τ^2 = 0.16, I^2 = 43% (15%, 62%)				
Design-by-treatment interaction model for inconsistency χ^2 *(d.f., P-value,* τ^2*)*: 8.06 (7, 0.327, 0.16)				
**Mean Difference: Studies with Mild to Moderate cognitive impairment, assessed with MMSE at baseline ***				
Donepezil vs Placebo	1.68	0.31 to 3.06	-4.81 to 8.18	0.69
Rivastigmine oral vs Placebo	0.88	-1.29 to 3.05	-5.85 to 7.61	0.51
Galantamine vs Placebo	0.31	-2.47 to 3.09	-6.66 to 7.28	0.40
Rivastigmine transdermal vs Placebo	2.74	-0.68 to 6.16	-4.53 to 10.01	0.81
Memantine vs Placebo	-0.58	-4.84 to 3.69	-8.31 to 7.16	0.28
Donepezil + Memantine vs Placebo	0.43	-6.36 to 7.21	-9.06 to 9.91	0.45
Galantamine + Memantine vs Placebo	0.88	-5.90 to 7.66	-8.61 to 10.37	0.51
Rivastigmine transdermal + Memantine vs Placebo	1.11	-4.20 to 6.42	-7.30 to 9.52	0.55
Placebo (reference)				0.31
Common within-network between-study variance: τ^2 = 9.67, I^2 = 97% (97%, 98%)				
Design-by-treatment interaction model for inconsistency χ^2 *(d.f., P-value,* τ^2*)*: 3.22 (9, 0.96, 13.28)				
**Mean Difference: Studies with Moderate to Severe cognitive impairment, assessed with MMSE at baseline ***				
Donepezil vs Placebo	1.31	0.66 to 1.96	-0.01 to 2.63	0.78
Rivastigmine oral vs Placebo	-1.00	-1.87 to -0.12	-2.51 to 0.51	0.04
Galantamine vs Placebo	-0.21	-1.64 to 1.21	-2.28 to 1.86	0.28
Memantine vs Placebo	0.69	0.07 to 1.31	-0.61 to 2.00	0.59
Donepezil + Memantine vs Placebo	2.49	1.55 to 3.44	0.92 to 4.07	1.00
Placebo (reference)				0.32
Common within-network between-study variance: τ^2 = 0.18, I^2 = 44% (0%, 75%)				
Design-by-treatment interaction model for inconsistency χ^2 *(d.f., P-value,* τ^2*)*: 2.60 (1, 0.11, 0.11)				
**Mean Difference: Excluding outlier studies***[§]				
Donepezil vs Placebo	0.95	0.59 to 1.32	-0.64 to 2.54	0.57
Rivastigmine oral vs Placebo	0.65	0.09 to 1.22	-1.00 to 2.30	0.37
Galantamine vs Placebo	0.36	-0.38 to 1.09	-1.36 to 2.07	0.22
Rivastigmine transdermal vs Placebo	1.03	0.15 to 1.91	-0.76 to 2.82	0.59
Memantine vs Placebo	0.67	0.02 to 1.32	-1.01 to 2.35	0.39
Donepezil + Memantine vs Placebo	2.04	1.03 to 3.05	0.18 to 3.90	0.92
Galantamine + Memantine vs Placebo	1.87	0.08 to 3.66	-0.53 to 4.26	0.82
Rivastigmine transdermal + Memantine vs Placebo	1.10	-0.33 to 2.53	-1.03 to 3.23	0.58
Placebo (reference)				0.04
Common within-network between-study variance: τ^2 = 0.59, I^2 = 73% (64%, 79%)				
Design-by-treatment interaction model for inconsistency χ^2 *(d.f., P-value,* τ^2*)*: 10.60 (13, 0.64, 0.61)				
**Accounting for missing outcome data - Informative Missingness Difference of Means**[¶]				
Donepezil vs Placebo	1.42	0.51 to 2.33	0.51 to 2.33	0.59[‖]
Rivastigmine oral vs Placebo	0.45	-1.09 to 1.99	-1.09 to 1.99	0.30[‖]
Galantamine vs Placebo	0.19	-1.78 to 2.17	-1.78 to 2.17	0.25[‖]
Rivastigmine transdermal vs Placebo	2.37	-0.03 to 4.79	-0.03 to 4.79	0.76[‖]
Memantine vs Placebo	0.60	-1.09 to 2.42	-1.09 to 2.42	0.36[‖]
Donepezil + Memantine vs Placebo	2.55	0.09 to 5.01	0.09 to 5.01	0.80[‖]
Galantamine + Memantine vs Placebo	2.26	-2.03 to 6.56	-2.03 to 6.56	0.68[‖]
Rivastigmine transdermal + Memantine vs Placebo	1.81	-1.66 to 5.28	-1.66 to 5.28	0.61[‖]
Placebo (reference)				0.16[‖]
Common within-network between-study variance: τ^2 = 5.47[‖]				
Design-by-treatment interaction model for inconsistency χ^2 *(d.f., P-value,* τ^2*)*: 4.45 (11, 0.955, 6.45)				
**Mean Difference: Meta-regression, Trial Mean Age**[**]				
Donepezil vs Placebo	1.53	0.52 to 2.53	-3.17 to 6.27	0.50[††]
Rivastigmine oral vs Placebo	0.80	-0.84 to 2.44	-4.15 to 5.79	0.37[††]
Galantamine vs Placebo	0.60	-1.63 to 2.83	-4.57 to 5.72	0.25[††]
Rivastigmine transdermal vs Placebo	2.53	0.06 to 4.98	-2.72 to 7.80	0.75[††]
Memantine vs Placebo	0.79	-1.18 to 2.74	-4.33 to 5.85	0.37[††]
Donepezil + Memantine vs Placebo	2.66	0.09 to 5.19	-2.70 to 7.97	0.87[††]
Galantamine + Memantine vs Placebo	2.39	-2.02 to 6.84	-4.14 to 8.83	0.75[††]
Rivastigmine transdermal + Memantine vs Placebo	2.05	-1.53 to 5.59	-3.83 to 7.94	0.75[††]
Placebo (reference)				0.12[††]
Regression coefficient	0.03	-0.14 to 0.20		
Common within-network between-study variance: τ^2 = 5.50	3.72 to 8.51			

For peer review only - http://bmjopen.bmj.com/site/about/guidelines.xhtml

Design-by-treatment interaction model for inconsistency χ^2 (d.f., P-value, τ^2): 3.92 (11, 0.972, 8.76)				
**Mean Difference: NMA of studies with IPD adjusted for Age**				
Donepezil vs Placebo	0.72	0.03 to 1.42	-0.66 to 2.10	0.66
Rivastigmine oral vs Placebo	0.84	-0.05 to 1.73	-0.75 to 2.43	0.70
Galantamine vs Placebo	0.46	-0.24 to 1.15	-0.92 to 1.83	0.48
Rivastigmine transdermal vs Placebo	1.05	0.04 to 2.06	-0.68 to 2.78	0.83
Memantine vs Placebo	0.06	-0.72 to 0.84	-1.40 to 1.53	0.21
Placebo (reference)				0.12
Common within-network between-study variance: τ^2 = 0.12, I^2 = 29% (0%, 71%)				
Design-by-treatment interaction model for inconsistency χ^2 (d.f., P-value, τ^2): N/A (one closed loop with a single multi-arm trial)				
**Mean Difference: Meta-regression, Percent of Male Participants[**]**				
Donepezil vs Placebo	1.62	0.58 to 2.65	-3.40 to 6.61	0.62 [††]
Rivastigmine oral vs Placebo	0.73	-0.90 to 2.35	-4.30 to 5.81	0.37 [††]
Galantamine vs Placebo	0.62	-1.65 to 2.89	-4.75 to 5.93	0.25 [††]
Rivastigmine Transdermal vs Placebo	2.51	0.01 to 5.04	-2.78 to 7.94	0.75 [††]
Memantine vs Placebo	0.66	-1.47 to 2.77	-4.54 to 5.88	0.25 [††]
Donepezil + Memantine vs Placebo	2.52	-0.40 to 5.45	-3.09 to 8.17	0.75 [††]
Galantamine + Memantine vs Placebo	2.27	-2.28 to 6.83	-4.37 to 8.90	0.75 [††]
Rivastigmine transdermal + Memantine vs Placebo	1.98	-1.67 to 5.65	-4.02 to 7.99	0.75 [††]
Placebo (reference)				0.12 [††]
Regression coefficient	0.01	-0.05 to 0.06		
Common within-network between-study variance: τ^2 = 5.73	3.83 to 8.84			
Design-by-treatment interaction model for inconsistency χ^2 (d.f., P-value, τ^2): 3.72 (10, 0.959, 8.97)				
**Mean difference: NMA of studies with IPD adjusted for Percent of Male Participants**				
Donepezil vs Placebo	0.76	0.05 to 1.47	-0.67 to 2.19	0.67
Rivastigmine oral vs Placebo	0.85	-0.07 to 1.77	-0.80 to 2.50	0.69
Galantamine vs Placebo	0.45	-0.27 to 1.16	-0.99 to 1.88	0.46
Rivastigmine transdermal vs Placebo	1.05	0.01 to 2.09	-0.74 to 2.84	0.81
Memantine vs Placebo	0.10	-0.68 to 0.89	-1.40 to 1.61	0.23
Placebo (reference)				0.11
Common within-network between-study variance: τ^2 = 0.13, I^2 = 32% (0%, 72%)				
Design-by-treatment interaction model for inconsistency χ^2 (d.f., P-value, τ^2): N/A (one closed loop with a single multi-arm trial)				
**Mean Difference: NMA of studies with IPD adjusted for cognitive impairment, assessed with MMSE at baseline**				
Donepezil vs Placebo	0.79	0.26 to 1.32	-0.06 to 1.64	0.64
Rivastigmine oral vs Placebo	0.88	0.31 to 1.45	-0.05 to 1.81	0.69
Galantamine vs Placebo	0.76	0.34 to 1.18	0.08 to 1.44	0.62
Rivastigmine transdermal vs Placebo	1.02	0.27 to 1.77	-0.20 to 2.24	0.82
Memantine vs Placebo	0.07	-0.52 to 0.66	-0.89 to 1.03	0.14
Placebo (reference)				0.08
Common within-network between-study variance: τ^2 = 0.00, I^2 = 0% (0%, 79%)				
Design-by-treatment interaction model for inconsistency χ^2 (d.f., P-value, τ^2): N/A (one closed loop with a single multi-arm trial)				
**Mean Difference: NMA of studies with IPD adjusted for comorbidities**				
Donepezil vs Placebo	0.77	0.21 to 1.33	-0.15 to 1.68	0.71
Rivastigmine oral vs Placebo	0.88	0.31 to 1.45	-0.05 to 1.81	0.75
Galantamine vs Placebo	-0.29	-1.46 to 0.88	-2.19 to 1.61	0.15
Rivastigmine transdermal vs Placebo	1.05	0.30 to 1.80	-0.17 to 2.27	0.88
Memantine vs Placebo	0.05	-0.55 to 0.64	-0.92 to 1.01	0.27
Placebo (reference)				0.15
Common within-network between-study variance: τ^2 = 0.00, I^2 = 0% (0%, 67%)				
Design-by-treatment interaction model for inconsistency χ^2 (d.f., P-value, τ^2): N/A (one closed loop with a single multi-arm trial)				
**Mean Difference: NMA of studies with IPD adjusted for other medications**				
Donepezil vs Placebo	0.67	-0.34 to 1.69	-1.44 to 2.79	0.61
Rivastigmine oral vs Placebo	0.87	-0.12 to 1.86	-1.21 to 2.95	0.71
Galantamine vs Placebo	0.42	-0.35 to 1.19	-1.40 to 2.25	0.47
Rivastigmine transdermal vs Placebo	1.07	-0.04 to 2.18	-1.16 to 3.30	0.81
Memantine vs Placebo	0.11	-0.74 to 0.96	-1.80 to 2.02	0.26
Placebo (reference)				0.14
Common within-network between-study variance: τ^2 = 0.17, I^2 = 35% (0%, 76%)				
Design-by-treatment interaction model for inconsistency χ^2 (d.f., P-value, τ^2): N/A (one closed loop with a single multi-arm trial)				
**Mean Difference: Meta-regression, Study Duration[**]**				
Donepezil vs Placebo	1.66	0.67 to 2.66	-3.12 to 6.32	0.62 [††]
Rivastigmine oral vs Placebo	0.80	-0.77 to 2.37	-4.14 to 5.69	0.37 [††]
Galantamine vs Placebo	0.47	-1.75 to 2.68	-4.64 to 5.66	0.25 [††]
Rivastigmine transdermal vs Placebo	2.38	-0.04 to 4.83	-2.87 to 7.56	0.75 [††]
Memantine vs Placebo	0.67	-1.27 to 2.58	-4.35 to 5.79	0.25 [††]
Donepezil + Memantine vs Placebo	2.67	0.18 to 5.16	-2.60 to 7.97	0.88 [††]
Galantamine + Memantine vs Placebo	2.43	-1.94 to 6.79	-3.94 to 8.81	0.75 [††]
Rivastigmine transdermal + Memantine vs Placebo	2.13	-1.40 to 5.63	-3.62 to 7.87	0.75 [††]
Placebo (reference)				0.12 [††]

For peer review only - http://bmjopen.bmj.com/site/about/guidelines.xhtml

Regression coefficient	0.02	-0.01 to 0.06		
Common within-network between-study variance: τ^2 = 5.40	3.63 to 8.29			
Design-by-treatment interaction model for inconsistency χ^2 (d.f., P-value, τ^2): 4.36 (13, 0.987, 7.35)				
**Mean Difference: Meta-regression, Year of Publication****				
Donepezil vs Placebo	1.53	0.51 to 2.54	-3.27 to 6.31	0.50 ††
Rivastigmine oral vs Placebo	0.66	-1.01 to 2.32	-4.31 to 5.65	0.25 ††
Galantamine vs Placebo	0.60	-1.65 to 2.85	-4.65 to 5.83	0.25 ††
Rivastigmine transdermal vs Placebo	2.59	0.09 to 5.12	-2.73 to 7.95	0.75 ††
Memantine vs Placebo	0.89	-1.05 to 2.80	-4.17 to 5.90	0.38 ††
Donepezil + Memantine vs Placebo	2.82	0.19 to 5.44	-2.57 to 8.21	0.88 ††
Galantamine + Memantine vs Placebo	2.59	-1.93 to 7.16	-3.98 to 9.12	0.75 ††
Rivastigmine transdermal + Memantine vs Placebo	2.21	-1.49 to 5.95	-3.81 to 8.24	0.75 ††
Placebo (reference)				0.12 ††
Regression coefficient	-0.02	-0.17 to 0.14		
Common within-network between-study variance: τ^2 = 5.53	3.71 to 8.48			
Design-by-treatment interaction model for inconsistency χ^2 (d.f., P-value, τ^2): 4.36 (13, 0.987, 7.35)				
**Serious Adverse Events (SAEs)‡**				
**Odds Ratio: Aggregate data and crude results from studies with available individual patient data**				
Donepezil vs Placebo	1.07	0.86 to 1.32	0.68 to 1.67	0.31
Rivastigmine oral vs Placebo	1.26	0.83 to 1.90	0.70 to 2.24	0.16
Galantamine vs Placebo	0.95	0.75 to 1.21	0.60 to 1.51	0.52
Rivastigmine transdermal vs Placebo	0.87	0.57 to 1.35	0.48 to 1.58	0.61
Memantine vs Placebo	0.91	0.67 to 1.22	0.55 to 1.49	0.59
Donepezil + Memantine vs Placebo	0.76	0.34 to 1.68	0.31 to 1.88	0.69
Galantamine + Memantine vs Placebo	1.03	0.45 to 2.36	0.41 to 2.64	0.42
Rivastigmine transdermal + Memantine vs Placebo	0.69	0.32 to 1.51	0.28 to 1.70	0.77
Placebo (reference)				0.43
Common within-network between-study variance τ^2 = 0.04, I^2 = 20% (0%, 47%)				
Design-by-treatment interaction model for inconsistency χ^2 (d.f., P-value, τ^2): 3.58 (6, 0.733, 0.05)				
**Odds Ratio: Aggregate data results****				
Donepezil vs Placebo	1.09	0.89 to 1.33	0.88 to 1.35	0.25
Rivastigmine oral vs Placebo	1.43	0.92 to 2.21	0.90 to 2.26	0.07
Galantamine vs Placebo	0.88	0.63 to 1.25	0.62 to 1.27	0.54
Rivastigmine transdermal vs Placebo	0.79	0.44 to 1.41	0.43 to 1.45	0.61
Memantine vs Placebo	0.70	0.51 to 0.97	0.50 to 0.98	0.77
Donepezil + Memantine vs Placebo	0.77	0.39 to 1.54	0.37 to 1.60	0.64
Galantamine + Memantine vs Placebo	0.96	0.45 to 2.08	0.43 to 2.16	0.44
Rivastigmine transdermal + Memantine vs Placebo	0.62	0.28 to 1.40	0.27 to 1.46	0.80
Placebo (reference)				0.38
Common within-network between-study variance τ^2 = 0.00, I^2 = 0% (0%, 42%)				
Design-by-treatment interaction model for inconsistency χ^2 (d.f., P-value, τ^2): 2.29 (4, 0.682, 0.01)				
**Odds Ratio: Crude results from studies with available individual patient data**				
Donepezil vs Placebo	0.95	0.50 to 1.78	0.33 to 2.70	0.57
Rivastigmine oral vs Placebo	0.81	0.37 to 1.75	0.25 to 2.61	0.71
Galantamine vs Placebo	1.05	0.71 to 1.56	0.44 to 2.50	0.46
Rivastigmine transdermal vs Placebo	0.92	0.38 to 2.20	0.26 to 3.31	0.57
Memantine vs Placebo	1.41	0.81 to 2.45	0.53 to 3.79	0.16
				0.53
Common within-network between-study variance τ^2 = 0.10, I^2 = 48% (0%, 76%)				
Design-by-treatment interaction model for inconsistency χ^2 (d.f., P-value, τ^2): N/A (no closed loops)				
**Odds Ratio: Low Risk of Bias for Allocation Concealment***				
Donepezil vs Placebo	0.88	0.60 to 1.29	0.42 to 1.83	0.52
Rivastigmine oral vs Placebo	1.15	0.67 to 1.98	0.50 to 2.68	0.21
Galantamine vs Placebo	0.94	0.64 to 1.38	0.45 to 1.95	0.44
Rivastigmine transdermal vs Placebo	0.88	0.52 to 1.49	0.39 to 2.02	0.51
Memantine vs Placebo	0.86	0.55 to 1.36	0.40 to 1.88	0.54
Donepezil + Memantine vs Placebo	0.63	0.24 to 1.62	0.19 to 2.05	0.75
Rivastigmine transdermal + Memantine vs Placebo	0.67	0.25 to 1.80	0.20 to 2.28	0.71
Placebo (reference)				0.33
Common within-network between-study variance: τ^2 = 0.08, I^2 = 37% (0%, 64%)				
Design-by-treatment interaction model for inconsistency χ^2 (d.f., P-value, τ^2): 2.19 (3, 0.53, 0.1)				
**Odds Ratio: Low Risk of Bias for Incomplete Data***				
Donepezil vs Placebo	0.83	0.53 to 1.29	0.45 to 1.51	0.51
Galantamine vs Placebo	0.69	0.50 to 0.97	0.42 to 1.13	0.80
Rivastigmine transdermal vs Placebo	0.79	0.42 to 1.49	0.36 to 1.76	0.56
Memantine vs Placebo	0.86	0.60 to 1.22	0.51 to 1.43	0.47
Placebo (reference)				0.16

For peer review only - http://bmjopen.bmj.com/site/about/guidelines.xhtml

Common within-network between-study variance: τ^2 = 0.02, I^2 = 10% (0%, 50%)				
Design-by-treatment interaction model for inconsistency χ^2 (d.f., P-value, τ^2): 0.00 (1, 0.95, 0.04)				
**Odds Ratio: Publicly-Sponsored Studies***				
Donepezil vs Placebo	2.15	0.36 to 12.69	--	0.16
Memantine vs Placebo	0.71	0.45 to 1.12	--	0.86
Donepezil + Memantine vs Placebo	1.53	0.23 to 10.18	--	0.46
Placebo (reference)				0.51
Common within-network between-study variance: τ^2 = N/A (each comparison includes a single study)				
Design-by-treatment interaction model for inconsistency χ^2 (d.f., P-value, τ^2): N/A (no closed loops)				
**Odds Ratio: Industry-Sponsored Studies***				
Donepezil vs Placebo	1.08	0.86 to 1.35	0.64 to 1.82	0.34
Rivastigmine oral vs Placebo	1.27	0.82 to 1.98	0.66 to 2.44	0.16
Galantamine vs Placebo	0.99	0.75 to 1.31	0.57 to 1.71	0.52
Rivastigmine transdermal vs Placebo	0.91	0.57 to 1.44	0.46 to 1.77	0.62
Memantine vs Placebo	0.95	0.65 to 1.37	0.52 to 1.73	0.58
Rivastigmine transdermal + Memantine vs Placebo	0.72	0.31 to 1.64	0.27 to 1.90	0.79
Placebo (reference)				0.50
Common within-network between-study variance: τ^2 = 0.05, I^2 = 25% (0%, 50%)				
Design-by-treatment interaction model for inconsistency χ^2 (d.f., P-value, τ^2): 3.68 (6, 0.72, 0.07)				
**Odds Ratio: Studies with Mild to Moderate cognitive impairment, assessed with MMSE at baseline ***				
Donepezil vs Placebo	1.27	0.88 to 1.83	0.61 to 2.65	0.29
Rivastigmine oral vs Placebo	1.36	0.83 to 2.24	0.60 to 3.09	0.25
Galantamine vs Placebo	1.01	0.67 to 1.55	0.47 to 2.19	0.56
Rivastigmine transdermal vs Placebo	1.02	0.50 to 2.05	0.39 to 2.69	0.55
Memantine vs Placebo	0.86	0.54 to 1.37	0.39 to 1.91	0.73
Galantamine + Memantine vs Placebo	1.10	0.40 to 3.00	0.32 to 3.78	0.48
Rivastigmine transdermal + Memantine vs Placebo	0.96	0.18 to 5.19	0.14 to 6.37	0.55
Placebo (reference)				0.59
Common within-network between-study variance: τ^2 = 0.09, I^2 = 29% (0%, 57%)				
Design-by-treatment interaction model for inconsistency χ^2 (d.f., P-value, τ^2): 3.29 (5, 0.66, 0.13)				
**Odds Ratio: Studies with Moderate to Severe cognitive impairment, assessed with MMSE at baseline ***				
Donepezil vs Placebo	0.92	0.67 to 1.27	0.59 to 1.45	0.38
Galantamine vs Placebo	0.70	0.46 to 1.07	0.38 to 1.28	0.76
Memantine vs Placebo	0.95	0.55 to 1.62	0.44 to 2.02	0.36
Donepezil + Memantine vs Placebo	0.66	0.32 to 1.37	0.23 to 1.86	0.76
Placebo (reference)				0.23
Common within-network between-study variance: τ^2 = 0.00, I^2 = 0% (0%, 72%)				
Design-by-treatment interaction model for inconsistency χ^2 (d.f., P-value, τ^2): 2.90 (1, 0.09, 0.00)				
**Odds Ratio: NMA of studies with IPD – available case analysis**				
Donepezil vs Placebo	1.63	0.49 to 5.41	0.30 to 8.73	0.33
Rivastigmine oral vs Placebo	1.28	0.08 to 19.94	0.04 to 39.11	0.46
Galantamine vs Placebo	1.05	0.67 to 1.63	0.38 to 2.85	0.58
Rivastigmine transdermal vs Placebo	0.81	0.02 to 35.04	0.01 to 82.49	0.59
Memantine vs Placebo	1.35	0.72 to 2.55	0.43 to 4.24	0.38
Placebo (reference)				0.64
Common within-network between-study variance: τ^2 = 0.13, I^2 = 50% (0%, 77%)				
Design-by-treatment interaction model for inconsistency χ^2 (d.f., P-value, heterogeneity): N/A (no closed loops)				
**Odds Ratio: Meta-regression, Trial Mean Age****				
Donepezil vs Placebo	1.13	0.88 to 1.43	0.68 to 1.86	0.25 ††
Rivastigmine oral vs Placebo	1.52	0.89 to 2.53	0.77 to 3.04	0.00 ††
Galantamine vs Placebo	0.91	0.60 to 1.30	0.52 to 1.59	0.50 ††
Rivastigmine transdermal vs Placebo	0.84	0.39 to 1.58	0.34 to 1.80	0.75 ††
Memantine vs Placebo	0.74	0.48 to 1.07	0.39 to 1.26	0.75 ††
Donepezil + Memantine vs Placebo	0.92	0.38 to 1.89	0.33 to 2.15	0.62 ††
Galantamine + Memantine vs Placebo	0.99	0.37 to 2.27	0.33 to 2.55	0.50 ††
Rivastigmine transdermal + Memantine vs Placebo	0.73	0.24 to 1.70	0.22 to 1.87	0.87 ††
Placebo (reference)				0.37 ††
Regression coefficient (log-scale)	-0.03	-0.08 to 0.02		
Common within-network between-study variance: τ^2 = 0.02	0.00 to 0.19			
Design-by-treatment interaction model for inconsistency χ^2 (d.f., P-value, τ^2): 3.57 (6, 0.735, 0.06)				
**Odds Ratio: NMA of studies with IPD adjusted for Age**				
Donepezil vs Placebo	0.95	0.50 to 1.78	0.33 to 2.73	0.57
Rivastigmine oral vs Placebo	0.84	0.39 to 1.81	0.26 to 2.74	0.68
Galantamine vs Placebo	1.04	0.70 to 1.55	0.43 to 2.52	0.46
Rivastigmine transdermal vs Placebo	0.91	0.38 to 2.17	0.25 to 3.28	0.58
Memantine vs Placebo	1.39	0.80 to 2.44	0.52 to 3.79	0.17
Placebo (reference)				0.53
Common within-network between-study variance: τ^2 = 0.10, I^2 = 48% (0%, 76%)				
Design-by-treatment interaction model for inconsistency χ^2 (d.f., P-value, τ^2): N/A (no closed loops)				

For peer review only - http://bmjopen.bmj.com/site/about/guidelines.xhtml

**Odds Ratio: Meta-regression, Percent of Male Participants[**]**				
Donepezil vs Placebo	1.12	0.87 to 1.44	0.64 to 2.01	0.25 [††]
Rivastigmine oral vs Placebo	1.71	0.97 to 2.92	0.83 to 3.67	0.00 [††]
Galantamine vs Placebo	0.93	0.62 to 1.36	0.49 to 1.77	0.50 [††]
Rivastigmine transdermal vs Placebo	0.89	0.39 to 1.79	0.34 to 2.05	0.63 [††]
Memantine vs Placebo	0.64	0.37 to 1.00	0.29 to 1.21	0.88 [††]
Donepezil + Memantine vs Placebo	0.88	0.35 to 1.88	0.30 to 2.13	0.63 [††]
Galantamine + Memantine vs Placebo	1.13	0.39 to 2.58	0.36 to 2.95	0.38 [††]
Rivastigmine transdermal + Memantine vs Placebo	0.77	0.24 to 1.93	0.21 to 2.13	0.88 [††]
Placebo (reference)				0.38 [††]
Regression coefficient (log-scale)	0.00	0.00 to 0.02		
Common within-network between-study variance: $\tau^2 = 0.03$	0.00 to 0.23			
Design-by-treatment interaction model for inconsistency χ^2 (d.f., P-value, τ^2): 3.57 (6, 0.735, 0.06)				
**Odds Ratio: NMA of studies with IPD adjusted for Percent of Male Participants**				
Donepezil vs Placebo	1.04	0.54 to 1.99	0.34 to 3.16	0.49
Rivastigmine oral vs Placebo	0.81	0.37 to 1.80	0.24 to 2.79	0.72
Galantamine vs Placebo	1.05	0.70 to 1.59	0.42 to 2.65	0.48
Rivastigmine transdermal vs Placebo	0.92	0.37 to 2.27	0.24 to 3.52	0.58
Memantine vs Placebo	1.40	0.80 to 2.48	0.50 to 3.98	0.19
Placebo (reference)				0.55
Common within-network between-study variance: $\tau^2 = 0.11$, $I^2 = 51\%$ (0%, 77%)				
Design-by-treatment interaction model for inconsistency χ^2 (d.f., P-value, τ^2): N/A (no closed loops)				
**Odds Ratio: NMA of studies with IPD adjusted for cognitive impairment, assessed with MMSE at baseline**				
Donepezil vs Placebo	0.97	0.46 to 2.06	0.23 to 4.03	0.56
Rivastigmine oral vs Placebo	0.81	0.33 to 2.01	0.17 to 3.91	0.70
Galantamine vs Placebo	1.29	0.74 to 2.25	0.37 to 4.55	0.28
Rivastigmine transdermal vs Placebo	0.93	0.34 to 2.53	0.18 to 4.91	0.57
Memantine vs Placebo	1.26	0.59 to 2.70	0.30 to 5.28	0.33
Placebo (reference)				0.56
Common within-network between-study variance: $\tau^2 = 0.16$, $I^2 = 52\%$ (0%, 80%)				
Design-by-treatment interaction model for inconsistency χ^2 (d.f., P-value, τ^2): N/A (no closed loops)				
**Odds Ratio: NMA of studies with IPD adjusted for comorbidities**				
Donepezil vs Placebo	1.01	0.52 to 1.96	0.29 to 3.50	0.51
Rivastigmine oral vs Placebo	0.82	0.36 to 1.87	0.20 to 3.32	0.69
Galantamine vs Placebo	1.02	0.57 to 1.80	0.32 to 3.26	0.50
Rivastigmine transdermal vs Placebo	0.91	0.36 to 2.31	0.20 to 4.11	0.58
Memantine vs Placebo	1.42	0.79 to 2.55	0.44 to 4.59	0.18
Placebo (reference)				0.53
Common within-network between-study variance: $\tau^2 = 0.12$, $I^2 = 44\%$ (0%, 77%)				
Design-by-treatment interaction model for inconsistency χ^2 (d.f., P-value, τ^2): N/A (no closed loops)				
**Odds Ratio: NMA of studies with IPD adjusted for other medications**				
Donepezil vs Placebo	1.17	0.49 to 3.03	0.28 to 4.88	0.41
Rivastigmine oral vs Placebo	0.82	0.37 to 1.81	0.23 to 2.91	0.72
Galantamine vs Placebo	1.03	0.69 to 1.55	0.40 to 2.65	0.51
Rivastigmine transdermal vs Placebo	0.95	0.39 to 2.34	0.24 to 2.91	0.56
Memantine vs Placebo	1.34	0.75 to 2.39	0.46 to 3.92	0.25
Placebo (reference)				0.56
Common within-network between-study variance: $\tau^2 = 0.11$, $I^2 = 51\%$ (0%, 78%)				
Design-by-treatment interaction model for inconsistency χ^2 (d.f., P-value, τ^2): N/A (no closed loops)				
**Odds Ratio: Meta-regression, Study Duration[**]**				
Donepezil vs Placebo	1.12	0.87 to 1.43	0.63 to 1.95	0.25 [††]
Rivastigmine oral vs Placebo	1.76	1.00 to 2.99	0.88 to 3.68	0.00 [††]
Galantamine vs Placebo	0.92	0.62 to 1.36	0.50 to 1.69	0.50 [††]
Rivastigmine transdermal vs Placebo	0.87	0.39 to 1.70	0.34 to 1.96	0.63 [††]
Memantine vs Placebo	0.61	0.37 to 0.93	0.31 to 1.13	0.88 [††]
Donepezil + Memantine vs Placebo	0.76	0.29 to 1.69	0.26 to 1.90	0.75 [††]
Galantamine + Memantine vs Placebo	0.98	0.34 to 2.26	0.30 to 2.53	0.50 [††]
Rivastigmine transdermal + Memantine vs Placebo	0.75	0.25 to 1.81	0.23 to 1.97	0.75 [††]
Placebo (reference)				0.38 [††]
Regression coefficient (log-scale)	0.00	0.00 to 0.01		
Common within-network between-study variance: $\tau^2 = 0.03$	0.00 to 0.22			
Design-by-treatment interaction model for inconsistency χ^2 (d.f., P-value, τ^2): 3.57 (6, 0.735, 0.06)				
**Odds Ratio: Meta-regression, Year of Publication[**]**				
Donepezil vs Placebo	1.05	0.79 to 1.38	0.61 to 1.77	0.38 [††]
Rivastigmine oral vs Placebo	1.68	0.98 to 2.77	0.85 to 3.37	0.00 [††]
Galantamine vs Placebo	0.91	0.61 to 1.32	0.50 to 1.64	0.63 [††]
Rivastigmine transdermal vs Placebo	0.92	0.40 to 1.84	0.36 to 2.04	0.63 [††]
Memantine vs Placebo	0.73	0.46 to 1.05	0.38 to 1.28	0.88 [††]
Donepezil + Memantine vs Placebo	0.88	0.35 to 1.83	0.31 to 2.15	0.75 [††]

For peer review only

For peer review only - http://bmjopen.bmj.com/site/about/guidelines.xhtml

Galantamine + Memantine vs Placebo	1.24	0.43 to 2.85	0.39 to 3.25	0.25 ††
Rivastigmine transdermal + Memantine vs Placebo	0.88	0.24 to 2.24	0.24 to 2.42	0.75 ††
Placebo (reference)				0.38 ††
Regression coefficient (log-scale)	-0.02	-0.06 to 0.03		
Common within-network between-study variance: $\tau^2 = 0.02$	0.00 to 0.21			
Design-by-treatment interaction model for inconsistency χ^2 (d.f., P-value, τ^2): 3.57 (6, 0.735, 0.06)				

* Aggregate data and fully adjusted results from studies with available individual patient data
† MMSE: Studies with available IPD included only available participants – to assess the missing data impact on the second stage a separate analysis was applied (IMDoM)
‡ SAE: Studies with available IPD included all randomized participants
§ Outlier studies:

- Hernandez C, Unturbe F, Martinez-Lage P, Lucas A, Gregorio P, Alonso T. Effects of combined pharmacologic and cognitive treatment in the progression of moderate dementia: a two-year follow-up. REVISTA ESPANOLA DE GERIATRIA Y GERONTOLOGIA. 2007;42(1):3
- Moretti DV. Alpha rhythm oscillations and MMSE scores are differently modified by transdermal or oral rivastigmine in patients with Alzheimer's disease. American journal of neurodegenerative disease. 2014;3(2):72-83.

¶ Included studies with available raw data only, irrespective having access to individual patient data
|| Analyses were conducted in Stata using the *metamiss2* and *network* commands; I2 is not available; SUCRA values are presented instead of P-scores
** Studies with aggregate data were used (studies with available individual patient data were not included in this analysis)
†† Analyses were conducted in OpenBUGS, and SUCRA values were calculated instead of P-scores

For peer review only

Appendix 17: CINeMA results

Risk of bias contributions: The bar chart shows the contributions of each piece of study to the network estimate

Interventions
A: PLAC
B: DONE
C: RIVA_O
D: GALA
E: RIVA_P
F: MEMA
G: DONE+MEMA
H: GALA+MEMA
I: RIVA_P+MEMA

MMSE outcome

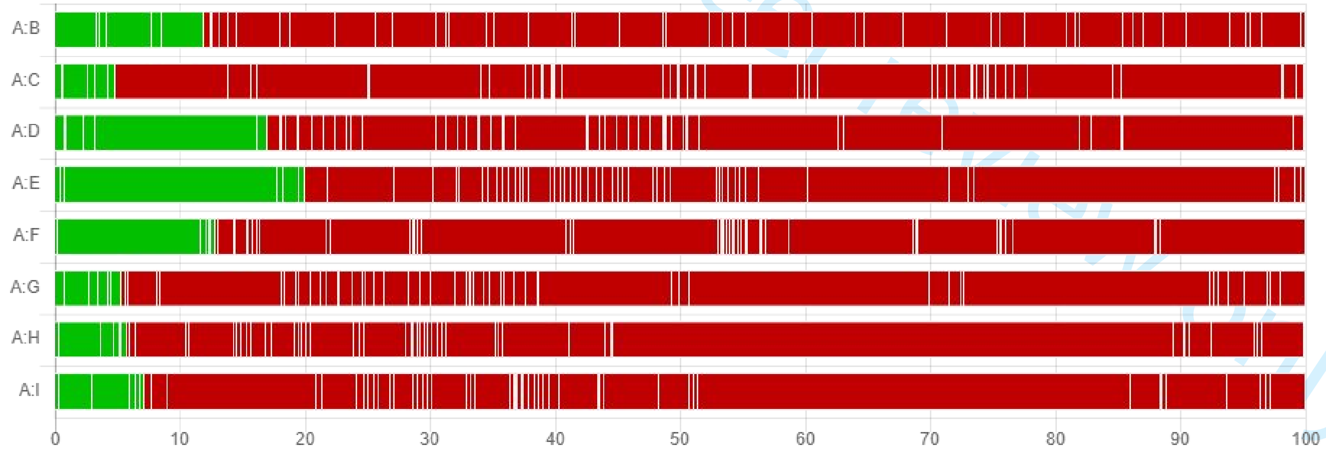

SAE outcome

For peer review only - http://bmjopen.bmj.com/site/about/guidelines.xhtml

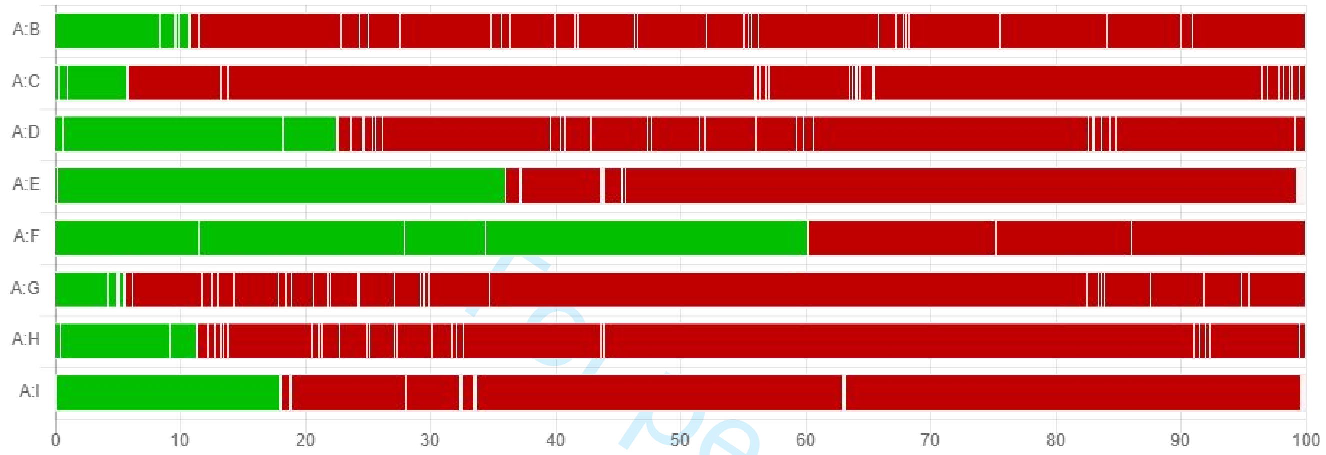

CINeMA report

MMSE outcome

Comparison	# of studies	Nature of evidence	Type of data	Within-study bias (D1)	Reporting bias (D2)	Indirectness (D3)	Imprecision (D4)	Heterogeneity (D5)	Incoherence (D6)	Confidence rating	Downgrading due to
DONE vs PLAC	24	Mixed	IPD+AD	Major concerns	Suspected	No concerns	No concerns	Major concerns	No concerns	Moderate	D5
RIVA_O vs PLAC	6	Mixed	IPD+AD	Major concerns	Suspected	No concerns	Some concerns	Some concerns	No concerns	Moderate	D4;D5
GALA vs PLAC	3	Mixed	IPD+AD	Major concerns	Suspected	No concerns	Major concerns	No concerns	No concerns	Moderate	D4
RIVA_P vs PLAC	2	Mixed	IPD+AD	Major concerns	Suspected	No concerns	Some concerns	Some concerns	No concerns	Moderate	D4;D5
MEMA vs PLAC	7	Mixed	IPD+AD	Major concerns	Suspected	No concerns	Some concerns	Some concerns	No concerns	Moderate	D4;D5
DONE+MEMA vs PLAC	1	Mixed	AD	Major concerns	Suspected	No concerns	No concerns	Major concerns	No concerns	Moderate	D5
GALA+MEMA vs PLAC	0	Indirect	-	Major concerns	Suspected	No concerns	Major concerns	No concerns	No concerns	Moderate	D4
RIVA_P+MEMA vs PLAC	0	Indirect	-	Major concerns	Suspected	No concerns	Major concerns	No concerns	No concerns	Moderate	D4

SAE outcome

For peer review only - http://bmjopen.bmj.com/site/about/guidelines.xhtml

Comparison	# of studies	Nature of evidence	Type of data	Within-study bias (D1)	Reporting bias (D2)	Indirectness (D3)	Imprecision (D4)	Heterogeneity (D5)	Incoherence (D6)	Confidence rating	Downgrading due to
DONE vs PLAC	16	Mixed	IPD+AD	Major concerns	Undetected	No concerns	Major concerns	No concerns	No concerns	Moderate	D1
RIVA_O vs PLAC	3	Mixed	IPD+AD	Major concerns	Undetected	No concerns	Major concerns	No concerns	No concerns	Moderate	D1
GALA vs PLAC	8	Mixed	IPD+AD	Major concerns	Undetected	No concerns	Major concerns	No concerns	No concerns	Moderate	D1
RIVA_P vs PLAC	2	Mixed	IPD+AD	Some concerns	Undetected	No concerns	Major concerns	No concerns	No concerns	High	
MEMA vs PLAC	7	Mixed	IPD+AD	Some concerns	Undetected	No concerns	Major concerns	No concerns	No concerns	High	
DONE+MEMA vs PLAC	2	Mixed	AD	Major concerns	Undetected	No concerns	Major concerns	No concerns	No concerns	Moderate	D1
GALA+MEMA vs PLAC	0	Indirect	-	Major concerns	Undetected	No concerns	Major concerns	No concerns	No concerns	Moderate	D1
RIVA_P+MEMA vs PLAC	0	Indirect	-	Major concerns	Undetected	No concerns	Major concerns	No concerns	No concerns	Moderate	D1

Abbreviations: DONE, donepezil; GALA, galantamine; MEMA, memantine; PLAC, placebo; RIVA_O, rivastigmine oral; RIVA_P, rivastigmine patch

For peer review only - http://bmjopen.bmj.com/site/about/guidelines.xhtml

Appendix 18: Rank-heat plot for serious adverse events

Circles from inside out present results for different network meta-analyses including: i) aggregate data (AD) only (studies with available IPD are not included in the analysis), ii) crude results from individual studies with individual patient data (IPD), iii) AD and crude results from studies with available IPD, and iv) AD and fully adjusted results from studies with available IPD. Numbers within each sector correspond to the P-score values as calculated in each model.

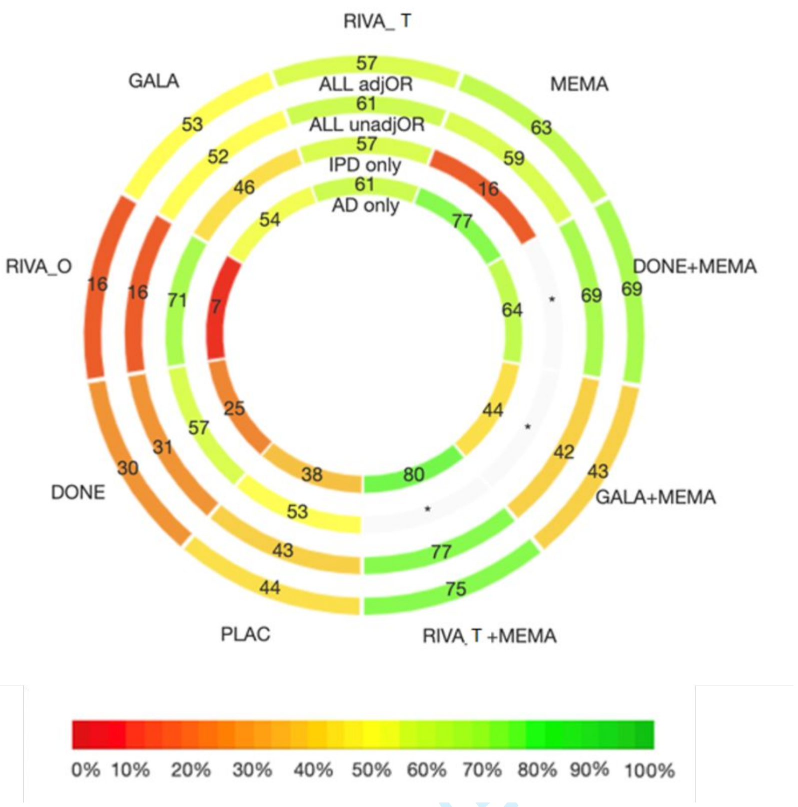

For peer review only

For peer review only - http://bmjopen.bmj.com/site/about/guidelines.xhtml

Appendix 19: Study definitions for serious adverse events

Author, Year	Source of Definition	Definition
Agid, 1998	Determined by Investigator	"Patients and caregivers were questioned systematically regarding the occurrence of adverse events at each clinical visit"
Ancoli-Israel, 2005	Determined by Investigator	"Only one serious AE leading to discontinuation, hepatic failure, in the donepezil-treated group was considered to be possibly due to study treatment by the investigator."
Andersen, 2012	NA	NA
Araki, 2014	NA	NA
Bakchine, 2008	Determined by Investigator	"Three patients had an SAE that was considered by the investigator to be possibly or probably related to treatment."
Black, 2007	Determined by Investigator	"AEs were considered serious (SAEs) when death occurred, life was threatened, hospitalization or prolonged hospitalization was required, or a significant disability occurred."
Blesa González, 2011	NA	NA
Burns,1999	COSTART	"Events were coded using a modified COSTART dictionary, and the assessment of relationship to treatment for all adverse events was conducted blind to treatment assignment."
Burns, 2009	NR	NR
Burns, 2011	NR	NR
Choi, 2011	Determined by Investigator	"Investigators were asked to evaluate severity (mild, moderate, or severe), relationship to study drug (not related, probable relationship with rivastigmine patch, probable relationship with memantine, or probable relationship with an interaction of the two drugs), and seriousness of the AEs."
Corey-Bloom, 1998	NA	NA
Cretu, 2008	NA	NA
Dysken, 2014	Medical Dictionary for Regulatory Activities	"Serious AEs were coded according to the Medical Dictionary for Regulatory Activities."
Farlow, 2013	NA	NA
Feldman, 2001	Determined by Investigator	"Serious AE was defined as any AE that was life threatening or resulted in death, hospitalization, prolongation of hospitalization, or significant disability."
Feldman, 2007	World Health Organisation preferred terms	“"All adverse events were recorded using the Novartis Medical Terminology Thesaurus (a modified version of the WHO adverse reaction terminology dictionary)."
Fox, 2012	NA	NA
Frolich, 2011	NA	NA
Fuschillo, 2001	NA	NA
Gault L, 2015	Medical Dictionary for Regulatory Activities	"AEs were coded using the Medical Dictionary for Regulatory Activities"
Gold, 2010	NR	NR
Greenberg, 2000	Determined by Investigator	“Of 9 withdrawals from the study after randomization, 2 were due to serious adverse events judged to be possibly related to donepezil therapy: syncope and generalized seizure (1 patient each).”
Grossberg, 2013	Medical Dictionary for Regulatory Activities	“Adverse events were coded according to the Medical Dictionary for Regulatory Activities (version 7.0 or newer), and an assessment of the severity, chronicity, causal relationship to study medication, and seriousness of the event was provided by an investigator”
Hager, 2014	Determined by Investigator	“Safety data were monitored during the study by a company-commissioned, external, independent, blinded Data Safety Monitoring Board (DSMB). Secondary safety outcomes were the number of treatment emergent adverse events (TEAEs), including serious TEAEs.”
Haig, 2014	NR	NR
Hernández, 2007	NA	NA
Herrmann, 2013	Determined by Investigator	“The incidence of adverse events considered related to the study drug by the investigator was 30% in the placebo group and 36% in the memantine group”
Holmes, 2004		
Homma, 1998	NR	NR
Homma, 2008	Medical Dictionary for Regulatory Activities – Japanese Version	"AE terms were standardized according to the Medical Dictionary for Regulatory Activities – Japanese Version . AEs were graded on a 3-point scale (mild: discomfort noticed, but no disruption of normal daily activity; moderate: discomfort sufficient to reduce or affect normal daily activity; severe: incapacitating, with inability to work or to perform normal daily activity). "
Hong, 2006	NR	NR
Howard, 2007	NA	NA
Howard, 2012	NR	NR
Hu, 2006	NA	NA

For peer review only - http://bmjopen.bmj.com/site/about/guidelines.xhtml

Johannsen, 2006	NA	NA
Jones, 2004	Determined by Investigator	"A serious adverse event (SAE) was defined as any AE that was life threatening or resulted in death, hospitalisation, prolongation of hospitalisation, or significant disability"
Kadir, 2008	NA	NA
Kano, 2013	NA	NA
Karaman, 2005	NA	NA
Likitjaroen, 2012	NA	NA
Lorenzi, 2011	NA	NA
Maher-Edwards, 2011	Determined by Investigator	"Eight subjects experienced nonfatal serious AEs; all were considered unrelated to the study drug"
Marek, 2014	Medical Dictionary for Regulatory Activities	"Aes were coded using the Medical Dictionary for Regulatory Activities (MedDRA, version 14.0) by system organ class and preferred term"
Mazza, 2006	NA	NA
Mohs, 2001	Determined by Investigator	"In all cases, judgment of the relationship of study treatment to an adverse event and of the severity of the event was made by the investigator under double-blind conditions."
Moretti, 2014	NA	NA
Mowla, 2007	NA	NA
Nakamura, 2011	Determined by Investigator	"Safety evaluations included recording all adverse events on Adverse Event Case Report Forms. Every serious adverse event occurring after the patient provided informed consent and until 28 days after the patient stopped the study was reported."
Nakano, 2001	NA	NA
Nordberg, 2009	Determined by Investigator	"Safety and tolerability were monitored throughout the study by recording all adverse events (AEs)."
Pakdaman H, 2015	NA	NA
Peng, 2005	NA	NA
Peskind, 2006	NR	NR
Peters O, 2015	NR	NR
Reisberg, 2003	NR	NR
Rockwood, 2001	World Health Organisation preferred terms	"adverse events (classified according to World Health Organisation preferred terms)."
Rockwood, 2006	NR	NR
Rogers, 1996		
Rogers, 1998	COSTART	"Events, recorded using investigator terminology, were grouped and coded into common terms using a modified COSTART dictionary"
Rogers, 1998	COSTART	"Events, recorded using investigator terminology, were grouped and coded into common terms using a modified COSTART dictionary."
Saxton, 2012	Determined by Investigator	"Treatment-emergent adverse events (TEAEs) and serious adverse events (SAEs) were recorded at all post-Screening study visits"
Scarpini, 2011	NR	NR
Schmidt, 2008	NA	NA
Seltzer, 2004	NA	NA
Shao, 2015	NA	NA
Shimizu, 2015	NA	NA
Sole-Padulles, 2013	NA	NA
Tariot, 2000	NR	NR
Tariot, 2001	COSTART	"Investigator terms describing AEs were coded to standard preferred terms using a modified Coding Symbols for Thesaurus of Adverse Reaction Terms dictionary."
Thomas, 2001		
Wilcock, 2003	World Health Organisation preferred terms	"monitoring for adverse events (classified according to WHO preferred terms)"
Wilkinson, 2001	Determined by Investigator	"All adverse events were recorded, regardless of the considered relationship to treatment. All details of adverse events and their outcomes were recorded including severity and relationship to treatment. Serious adverse events were documented separately."
Wilkinson, 2002	NR	NR
Wilkinson, 2012	NR	NR
Winblad, 2001	NR	NR
Winblad, 2006	COSTART	"We recorded all treatment emergent adverse events, coding them according to a modified COSTART dictionary."
Winblad, 2007	Determined by Investigator	"Safety evaluations included recording all adverse events, which were coded using a standard glossary."
Zhang-Yi, 2005	NA	NA
Zhang, 2012	Determined by Investigator	"Serious adverse events considered to be possibly related to treatment occurred in one patient in each treatment arm"

For peer review only - http://bmjopen.bmj.com/site/about/guidelines.xhtml

Notes: [a]Unpublished data, [b]Non-English studies
Abbreviations: CR, companion report; NA, not applicable; NR, not reported.

For peer review only

For peer review only - http://bmjopen.bmj.com/site/about/guidelines.xhtml

Appendix 20: Time taken to achieve at least one serious adverse event using individual patient data

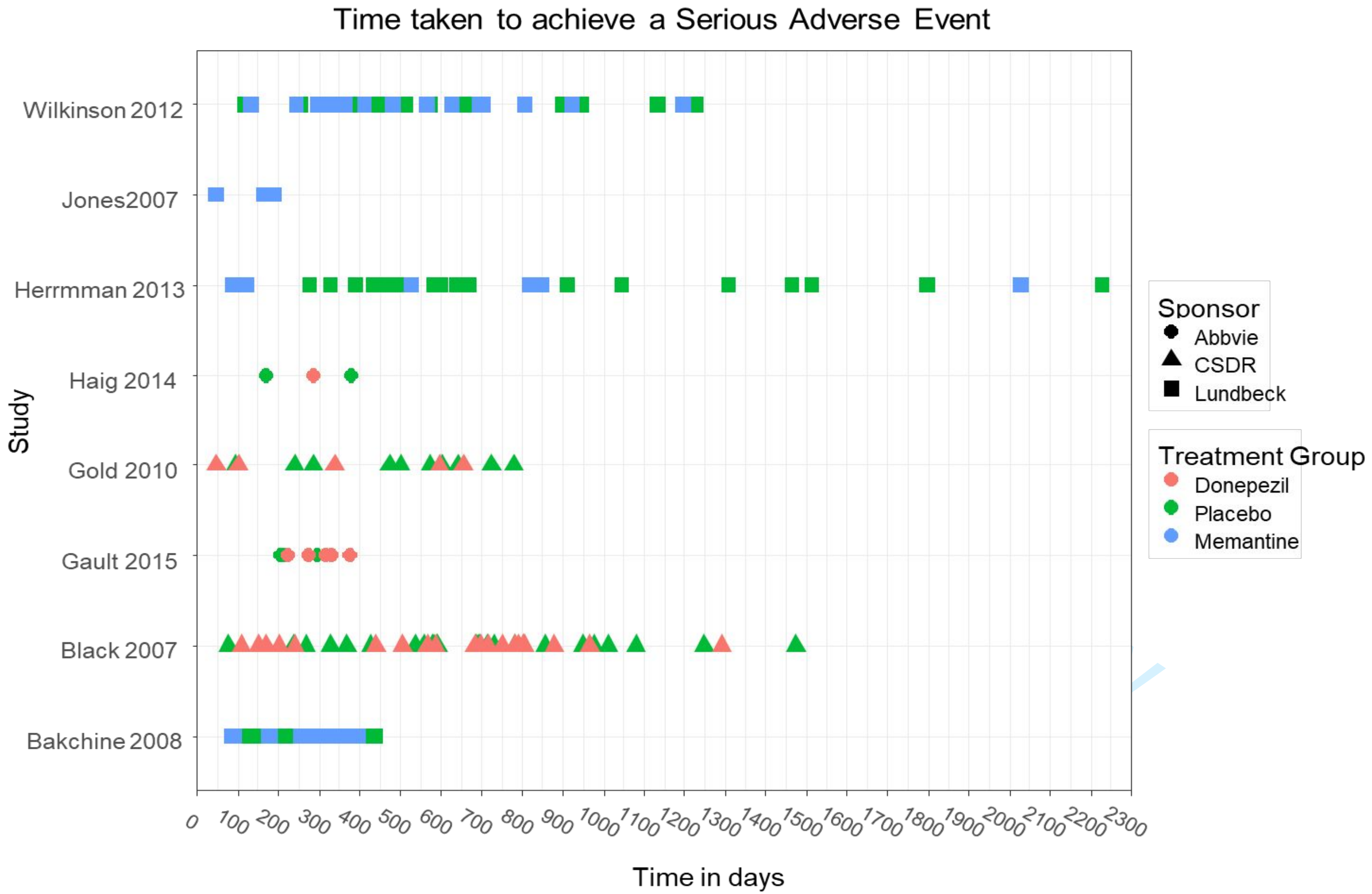

For peer review only - http://bmjopen.bmj.com/site/about/guidelines.xhtml

Appendix 21: Challenges encountered during the individual patient data request from sponsors

- The identification of the trial data set when certain details were not available (e.g. NCT number; particularly for studies published before 2005 that this was established).
- Data ownership.
- Sponsors switched platforms, while we were navigating the data.
- IPD available through proprietary sponsor-specific platforms did not allow for combination of IPD from different sponsor platforms; hence a one-stage analysis as planned in our protocol, was impossible.
- Software availability: Required R packages (e.g., mice) were not available/provided, and we were not allowed to install any new R packages; some R packages were older versions (e.g. lme4).
- Time that the platform permitted access to the IPD was often limited. This is a significant constraint given that IPD from different studies became available at different time points.
- Cost associated with obtaining access to the data for a certain amount of time. Additionally, cost associated with the WHO Drug Dictionary license to obtain access to the additional medications used for each patient; this license's approximate cost was $8,958·25 USD per sponsor.

For peer review only

- Available IPD did not include the full information as shown in the publication: For example, only data for placebo were available, or did not give information about a reported outcome (e.g. only baseline MMSE values were available). Also, date of follow-up was coded in some studies and it was impossible to make a judgement on first and last date.

For peer review only - http://bmjopen.bmj.com/site/about/guidelines.xhtml

Additional File 2: MEDLINE Search Strategy

MEDLINE Search

Database: Ovid MEDLINE(R) In-Process & Other Non-Indexed Citations and Ovid MEDLINE(R) <1946 to Present>, Embase<1980 to 2014 Week 50> Search Strategy:

----

1 alzheimer$.mp.
2 "benign senescent forgetfulness".mp.
3 (cognit$ adj2 (impair$ or declin$ or deficit$ or degenerat$ or deteriorat$ or los$ or disorder$ or complain$ or disturb$)).mp.
4 (cerebr$ adj2 (impair$ or declin$ or deficit$ or degenerat$ or deteriorat$ or los$ or disorder$ or complain$ or disturb$)).mp.
5 (mental adj2 (impair$ or declin$ or deficit$ or degenerat$ or deteriorat$ or los$ or disorder$ or complain$ or disturb$)).mp.
6 (ne?rocognit$ adj2 (impair$ or declin$ or deficit$ or degenerat$ or deteriorat$ or los$ or disorder$ or complain$ or disturb$)).mp.)
7 (ne?ro-cognit$ adj2 (impair$ or declin$ or deficit$ or degenerat$ or deteriorat$ or los$ or disorder$ or complain$ or disturb$)).mp.
8 ((cognit$ or memory or cerebral or brain) adj2 (improv$ or enhanc$ or perform$ or process$ or function$ or rehabilitation or aid$ or stimulat$)).mp.
9 cognition.ti.
10 (confusion$ or confused).tw.
11 dement$.mp.
12 ("normal pressure hydrocephalus" and shunt$).mp.
13 "organic brain disease$".mp.
14 "organic brain syndrome".mp.
15 (presenil$ or pre-senil$ or senil$).tw.
16 Alzheimer Disease/
17 Cognition/de
18 Confusion/
19 Dementia/
20 or/1-19
21 abixa.tw.
22 aricept.tw.
23 (acetylcholinesteraseadj inhibitor$).tw.
24 axura.tw.
25 akatinol.tw.
26 (anticholinesterase? or anti-cholinesterase?).tw.
27 (cognitive adjenhanc$).mp.
28 (cholinesterase adj inhibitor$).mp.
29 ChEI.tw.
30 donepezil.mp.
31 ebixa.tw.
32 eranz.tw.
33 exelon.tw.
34 galant?amin$.tw.
35 lycoremine.tw.

For peer review only
For peer review only - http://bmjopen.bmj.com/site/about/guidelines.xhtml

36 memantin$.tw.
37 memox.tw.
38 namenda.tw.
39 nimvastid.tw.
40 nivalin$.tw.
41 "N-Methyl-D-aspartic acid receptor antagonist$".tw.
42 prometax.tw.
43 razadyne.tw.
44 reminyl.tw.
45 rivastigmine.mp.
46 exp Cholinesterase Inhibitors/
47 Galantamine/
48 Memantine/
49 Galantamin.rn.
50 Memantine.rn.
51 Donepezil.rn.
52 Donepezil Hydrochloride.rn.
53 Rivastigmine.rn.
54 or/21-53
55 20 and 54
56 exp Animals/ not (exp Animals/ and Humans/)
57 55 and 56
58 (comment or editorial or interview or news).pt.
59 (letter not (letter and randomized controlled trial)).pt.
60 57 not (58 or 59)
61 (201111* or 201112* or 2012* or 2013* or 2014*).ed.
62 60 and 61
63 alzheimer$.mp.
64 "benign senescent forgetfulness".mp.
65 (cognit$ adj2 (impair$ or declin$ or deficit$ or degenerat$ or deteriorat$ or los$ or disorder$ or complain$ or disturb$)).mp.
66 (cerebr$ adj2 (impair$ or declin$ or deficit$ or degenerat$ or deteriorat$ or los$ or disorder$ or complain$ or disturb$)).mp.
67 (mental adj2 (impair$ or declin$ or deficit$ or degenerat$ or deteriorat$ or los$ or disorder$ or complain$ or disturb$)).mp.
68 (ne?rocognit$ adj2 (impair$ or declin$ or deficit$ or degenerat$ or deteriorat$ or los$ or disorder$ or complain$ or disturb$)).mp.
69 (ne?ro-cognit$ adj2 (impair$ or declin$ or deficit$ or degenerat$ or deteriorat$ or los$ or disorder$ or complain$ or disturb$)).mp.
70 ((cognit$ or memory or cerebral or brain) adj2 (improv$ or enhanc$ or perform$ or process$ or function$ or rehabilitation or aid$ or stimulat$)).mp.
71 cognition.ti.
72 (confusion$ or confused).tw.
73 dement$.mp.
74 ("normal pressure hydrocephalus" and shunt$).mp.
75 "organic brain disease$".mp.
76 "organic brain syndrome".mp.

For peer review only - http://bmjopen.bmj.com/site/about/guidelines.xhtml

77 (presenil$ or pre-senil$ or senil$).tw
78 Alzheimer disease/
79 cognitive defect/
80 confusion/
81 dementia/
82 organic brain syndrome/
83 or/63-82
84 abixa.tw.
85 aricept.tw.
86 (acetylcholinesteraseadj inhibitor$).tw.
87 axura.tw.
88 akatinol.tw.
89 (anticholinesterase? or anti-cholinesterase?).tw.
90 (cognitive adjenhanc$).mp.
91 (cholinesterase adj inhibitor$).mp.
92 ChEI.tw.
93 donepezil.mp.
94 ebixa.tw.
95 eranz.tw.
96 exelon.tw.
97 galant?amin$.tw.
98 lycoremine.tw.
99 memantin$.tw.
100 memox.tw.
101 namenda.tw.
102 nimvastid.tw.
103 nivalin$.tw.
104 "N-Methyl-D-aspartic acid receptor antagonist$".tw.
105 prometax.tw.
106 razadyne.tw.
107 reminyl.tw.
108 rivastigmine.mp.
109 exp cholinesterase inhibitor/
110 donepezil/ or donepezil plus memantine/
111 galantamine/
112 memantine/
113 rivastigmine/
114 357-70-0.rn.
115 19982-08-2.rn.
116 120011-70-3.rn.
117 120014-06-4.rn.
118 rivastigmine.rn.
119 or/84-118
120 83 and 119
121 randomized controlled trial/ or controlled clinical trial/
122 exp "clinical trial (topic)"/
123 (randomi#ed or randomly or RCT$1 or placebo*).tw.

For peer review only - http://bmjopen.bmj.com/site/about/guidelines.xhtml

124 ((singl* or doubl* or trebl* or tripl*) adj (mask* or blind* or dumm*)).tw.
125 trial.ti.
126 or/121-125
127 120 and 126
128 exp controlled clinical trial/
129 exp "controlled clinical trial (topic)"/
130 (control* adj2 trial*).tw.
131 (nonrandom* or non-random* or quasi-random* or quasi-experiment*).tw.
132 (nRCT or nRCTs or non-RCT$1).tw.
133 (control* adj3 ("before and after" or "before after")).tw.
134 time series analysis/
135 (time series adj3 interrupt*).tw.
136 pretest posttest control group design/
137 (pre- adj3 post-).tw.
138 (pretest adj3 posttest).tw.
139 controlled study/
140 (control* adj2 stud$3).tw.
141 control group/
142 (control$ adj2 group$1).tw.
143 or/128-142
144 120 and 143
145 cohort analysis/
146 cohort.tw.
147 retrospective study/
148 longitudinal study/
149 prospective study/
150 (longitudinal or prospective or retrospective).tw.
151 follow up/
152 ((followup or follow-up) adj (study or studies)).tw.
153 observational study/
154 (observation$2 adj (study or studies)).tw.
155 population research/
156 ((population or population-based) adj (study or studies or analys#s)).tw.
157 ((multidimensional or multi-dimensional) adj (study or studies)).tw.
158 exp comparative study/
159 ((comparative or comparison) adj (study or studies)).tw.
160 exp case control study/
161 ((case-control* or case-based or case-comparison) adj (study or studies)).tw.
162 or/145-161
163 120 and 162
164 127 or 144 or 163
165 exp animal experimentation/ or exp models animal/ or exp animal experiment/ or nonhuman/ or exp vertebrate/
166 exp humans/ or exp human experimentation/ or exp human experiment/
167 165 not 166
168 164 not 167
169 editorial.pt.

For peer review only - http://bmjopen.bmj.com/site/about/guidelines.xhtml

170 letter.pt.not (letter.pt. and randomized controlled trial/)
171 168 not (169 or 170)
172 (2011112* or 2011113* or 201112* or 2012* or 2013* or 2014*).dd.
173 171 and 172
174 62 use prmz
175 173 use emez
176 174 or 175
177 remove duplicates from 176
178 177 use prmz [MEDLINE UNIQUE HITS]
179 177 use emez [EMBASE UNIQUE HITS]
****

For peer review only

For peer review only - http://bmjopen.bmj.com/site/about/guidelines.xhtml

PRISMA-IPD Checklist of items to include when reporting a systematic review and meta-analysis of individual participant data (IPD)

PRISMA-IPD Section/topic	Item No	Checklist item	Reported on page
**Title**			
Title	1	Identify the report as a systematic review and meta-analysis of individual participant data.	1
**Abstract**			
Structured summary	2	Provide a structured summary including as applicable:	3-4
		**Background**: state research question and main objectives, with information on participants, interventions, comparators and outcomes.	
		**Methods**: report eligibility criteria; data sources including dates of last bibliographic search or elicitation, noting that IPD were sought; methods of assessing risk of bias.	
		**Results**: provide number and type of studies and participants identified and number (%) obtained; summary effect estimates for main outcomes (benefits and harms) with confidence intervals and measures of statistical heterogeneity. Describe the direction and size of summary effects in terms meaningful to those who would put findings into practice.	
		**Discussion:** state main strengths and limitations of the evidence, general interpretation of the results and any important implications.	
		**Other:** report primary funding source, registration number and registry name for the systematic review and IPD meta-analysis.	
**Introduction**			
Rationale	3	Describe the rationale for the review in the context of what is already known.	5
Objectives	4	Provide an explicit statement of the questions being addressed with reference, as applicable, to participants, interventions, comparisons, outcomes and study design (PICOS). Include any hypotheses that relate to particular types of participant-level subgroups.	5
**Methods**			

For peer review only

Protocol and registration	5	Indicate if a protocol exists and where it can be accessed. If available, provide registration information including registration number and registry name. Provide publication details, if applicable.	5, Appendix 1
Eligibility criteria	6	Specify inclusion and exclusion criteria including those relating to participants, interventions, comparisons, outcomes, study design and characteristics (e.g. years when conducted, required minimum follow-up). Note whether these were applied at the study or individual level i.e. whether eligible participants were included (and ineligible participants excluded) from a study that included a wider population than specified by the review inclusion criteria. The rationale for criteria should be stated.	5-6, Appendix 1
Identifying studies - information sources	7	Describe all methods of identifying published and unpublished studies including, as applicable: which bibliographic databases were searched with dates of coverage; details of any hand searching including of conference proceedings; use of study registers and agency or company databases; contact with the original research team and experts in the field; open adverts and surveys. Give the date of last search or elicitation.	6, Appendix 1
Identifying studies - search	8	Present the full electronic search strategy for at least one database, including any limits used, such that it could be repeated.	N/A (see published protocol)
Study selection processes	9	State the process for determining which studies were eligible for inclusion.	6, Appendix 1
Data collection processes	10	Describe how IPD were requested, collected and managed, including any processes for querying and confirming data with investigators. If IPD were not sought from any eligible study, the reason for this should be stated (for each such study).	6, Appendix 1
		If applicable, describe how any studies for which IPD were not available were dealt with. This should include whether, how and what aggregate data were sought or extracted from study reports and publications (such as extracting data independently in duplicate) and any processes for obtaining and confirming these data with investigators.	
Data items	11	Describe how the information and variables to be collected were chosen. List and define all study level and participant level data that were sought, including baseline and follow-up information. If applicable, describe methods of standardising or translating variables within the IPD datasets to ensure common scales or measurements across studies.	6, Appendix 1

For peer review only

IPD integrity	A1	Describe what aspects of IPD were subject to data checking (such as sequence generation, data consistency and completeness, baseline imbalance) and how this was done.	Appendix 1
Risk of bias assessment in individual studies.	12	Describe methods used to assess risk of bias in the individual studies and whether this was applied separately for each outcome. If applicable, describe how findings of IPD checking were used to inform the assessment. Report if and how risk of bias assessment was used in any data synthesis.	6, Appendix 1
Specification of outcomes and effect measures	13	State all treatment comparisons of interests. State all outcomes addressed and define them in detail. State whether they were pre-specified for the review and, if applicable, whether they were primary/main or secondary/additional outcomes. Give the principal measures of effect (such as risk ratio, hazard ratio, difference in means) used for each outcome.	7, Appendix 1
Synthesis methods	14	Describe the meta-analysis methods used to synthesise IPD. Specify any statistical methods and models used. Issues should include (but are not restricted to):<br>• Use of a one-stage or two-stage approach.<br>• How effect estimates were generated separately within each study and combined across studies (where applicable).<br>• Specification of one-stage models (where applicable) including how clustering of patients within studies was accounted for.<br>• Use of fixed or random effects models and any other model assumptions, such as proportional hazards.<br>• How (summary) survival curves were generated (where applicable).<br>• Methods for quantifying statistical heterogeneity (such as I^2 and τ^2).<br>• How studies providing IPD and not providing IPD were analysed together (where applicable).<br>• How missing data within the IPD were dealt with (where applicable).	7, Appendix 1
Exploration of variation in effects	A2	If applicable, describe any methods used to explore variation in effects by study or participant level characteristics (such as estimation of interactions between effect and covariates). State all participant-level characteristics that were analysed as potential effect modifiers, and whether these were pre-specified.	Appendix 1
Risk of bias across studies	15	Specify any assessment of risk of bias relating to the accumulated body of evidence, including any pertaining to not obtaining IPD for particular studies, outcomes or other variables.	6, Appendix 1

For peer review only

Additional analyses	16	Describe methods of any additional analyses, including sensitivity analyses. State which of these were pre-specified.	7, Appendix 1
**Results**			
Study selection and IPD obtained	17	Give numbers of studies screened, assessed for eligibility, and included in the systematic review with reasons for exclusions at each stage. Indicate the number of studies and participants for which IPD were sought and for which IPD were obtained. For those studies where IPD were not available, give the numbers of studies and participants for which aggregate data were available. Report reasons for non-availability of IPD. Include a flow diagram.	7 – Figure 1
Study characteristics	18	For each study, present information on key study and participant characteristics (such as description of interventions, numbers of participants, demographic data, unavailability of outcomes, funding source, and if applicable duration of follow-up). Provide (main) citations for each study. Where applicable, also report similar study characteristics for any studies not providing IPD.	8 – Table 1, Appendix 5
IPD integrity	A3	Report any important issues identified in checking IPD or state that there were none.	8-9, Appendices 5 and 10
Risk of bias within studies	19	Present data on risk of bias assessments. If applicable, describe whether data checking led to the up-weighting or down-weighting of these assessments. Consider how any potential bias impacts on the robustness of meta-analysis conclusions.	8-9 – Appendix 8
Results of individual studies	20	For each comparison and for each main outcome (benefit or harm), for each individual study report the number of eligible participants for which data were obtained and show simple summary data for each intervention group (including, where applicable, the number of events), effect estimates and confidence intervals. These may be tabulated or included on a forest plot.	Appendices 6 and 10 (full data can be provided by the

For peer review only

			first author)
Results of syntheses	21	Present summary effects for each meta-analysis undertaken, including confidence intervals and measures of statistical heterogeneity. State whether the analysis was pre-specified, and report the numbers of studies and participants and, where applicable, the number of events on which it is based.	9-11 – Appendix 15
		When exploring variation in effects due to patient or study characteristics, present summary interaction estimates for each characteristic examined, including confidence intervals and measures of statistical heterogeneity. State whether the analysis was pre-specified. State whether any interaction is consistent across trials.	
		Provide a description of the direction and size of effect in terms meaningful to those who would put findings into practice.	
Risk of bias across studies	22	Present results of any assessment of risk of bias relating to the accumulated body of evidence, including any pertaining to the availability and representativeness of available studies, outcomes or other variables.	9-11 - Appendix 12
Additional analyses	23	Give results of any additional analyses (e.g. sensitivity analyses). If applicable, this should also include any analyses that incorporate aggregate data for studies that do not have IPD. If applicable, summarise the main meta-analysis results following the inclusion or exclusion of studies for which IPD were not available.	9-11 - Appendices 16 and 17
**Discussion**			
Summary of evidence	24	Summarise the main findings, including the strength of evidence for each main outcome.	11-13
Strengths and limitations	25	Discuss any important strengths and limitations of the evidence including the benefits of access to IPD and any limitations arising from IPD that were not available.	13-14
Conclusions	26	Provide a general interpretation of the findings in the context of other evidence.	13-14
Implications	A4	Consider relevance to key groups (such as policy makers, service providers and service users). Consider implications for future research.	12-13

Funding			
Funding	27	Describe sources of funding and other support (such as supply of IPD), and the role in the systematic review of those providing such support.	15

A1 – A3 denote new items that are additional to standard PRISMA items. A4 has been created as a result of re-arranging content of the standard PRISMA statement to suit the way that systematic review IPD meta-analyses are reported.

© Reproduced with permission of the PRISMA IPD Group, which encourages sharing and reuse for non-commercial purposes

For peer review only

PRISMA NMA Checklist of Items to Include When Reporting A Systematic Review Involving a Network Meta-analysis

Section/Topic	Item #	Checklist Item	Reported on Page #
**TITLE**			
Title	1	Identify the report as a systematic review *incorporating a network meta-analysis (or related form of meta-analysis).*	1
**ABSTRACT**			
Structured summary	2	Provide a structured summary including, as applicable:<br>**Background:** main objectives<br>**Methods:** data sources; study eligibility criteria, participants, and interventions; study appraisal; and *synthesis methods, such as network meta-analysis.*<br>**Results:** number of studies and participants identified; summary estimates with corresponding confidence/credible intervals; *treatment rankings may also be discussed. Authors may choose to summarize pairwise comparisons against a chosen treatment included in their analyses for brevity.*<br>**Discussion/Conclusions:** limitations; conclusions and implications of findings.<br>**Other:** primary source of funding; systematic review registration number with registry name.	3-4
**INTRODUCTION**			
Rationale	3	Describe the rationale for the review in the context of what is already known*, including mention of why a network meta-analysis has been conducted.*	5
Objectives	4	Provide an explicit statement of questions being addressed, with reference to participants, interventions, comparisons, outcomes, and study design (PICOS).	5
**METHODS**			
Protocol and registration	5	Indicate whether a review protocol exists and if and where it can be accessed (e.g., Web address); and, if available, provide registration information, including registration number.	5, Appendix 1
Eligibility criteria	6	Specify study characteristics (e.g., PICOS, length of follow-up) and report characteristics (e.g., years considered, language, publication status) used as criteria for eligibility, giving rationale. *Clearly describe eligible treatments included in the treatment network, and note whether any have been clustered or merged into the same node (with justification).*	6, Appendix 1
Information sources	7	Describe all information sources (e.g., databases with dates of coverage, contact with study authors to identify additional studies) in the search and date last searched.	6, Appendix 1

For peer review only

Search	8	Present full electronic search strategy for at least one database, including any limits used, such that it could be repeated.	N/A (see published protocol)
Study selection	9	State the process for selecting studies (i.e., screening, eligibility, included in systematic review, and, if applicable, included in the meta-analysis).	6, Appendix 1
Data collection process	10	Describe method of data extraction from reports (e.g., piloted forms, independently, in duplicate) and any processes for obtaining and confirming data from investigators.	6, Appendix 1
Data items	11	List and define all variables for which data were sought (e.g., PICOS, funding sources) and any assumptions and simplifications made.	6, Appendix 1
**Geometry of the network**	**S1**	Describe methods used to explore the geometry of the treatment network under study and potential biases related to it. This should include how the evidence base has been graphically summarized for presentation, and what characteristics were compiled and used to describe the evidence base to readers.	7, Appendix 1
Risk of bias within individual studies	12	Describe methods used for assessing risk of bias of individual studies (including specification of whether this was done at the study or outcome level), and how this information is to be used in any data synthesis.	6, Appendix 1
Summary measures	13	State the principal summary measures (e.g., risk ratio, difference in means). *Also describe the use of additional summary measures assessed, such as treatment rankings and surface under the cumulative ranking curve (SUCRA) values, as well as modified approaches used to present summary findings from meta-analyses.*	7, Appendix 1
Planned methods of analysis	14	Describe the methods of handling data and combining results of studies for each network meta-analysis. This should include, but not be limited to:<br>• *Handling of multi-arm trials;*<br>• *Selection of variance structure;*<br>• *Selection of prior distributions in Bayesian analyses; and*<br>• *Assessment of model fit.*	7, Appendix 1
**Assessment of Inconsistency**	**S2**	Describe the statistical methods used to evaluate the agreement of direct and indirect evidence in the treatment network(s) studied. Describe efforts taken to address its presence when found.	7, Appendix 1
Risk of bias across studies	15	Specify any assessment of risk of bias that may affect the cumulative evidence (e.g., publication bias, selective reporting within studies).	6, Appendix 1
Additional analyses	16	Describe methods of additional analyses if done, indicating which were pre-specified. This may include, but not be limited to, the following:<br>• Sensitivity or subgroup analyses;<br>• Meta-regression analyses;<br>• *Alternative formulations of the treatment network; and*<br>• *Use of alternative prior distributions for Bayesian analyses (if applicable).*	7, Appendix 1

RESULTS†

Study selection	17	Give numbers of studies screened, assessed for eligibility, and included in the review, with reasons for exclusions at each stage, ideally with a flow diagram.	7 – Figure 1
**Presentation of network structure**	**S3**	Provide a network graph of the included studies to enable visualization of the geometry of the treatment network.	9 – Figure 2
**Summary of network geometry**	**S4**	Provide a brief overview of characteristics of the treatment network. This may include commentary on the abundance of trials and randomized patients for the different interventions and pairwise comparisons in the network, gaps of evidence in the treatment network, and potential biases reflected by the network structure.	7-8
Study characteristics	18	For each study, present characteristics for which data were extracted (e.g., study size, PICOS, follow-up period) and provide the citations.	8 – Table 1, Appendix 5
Risk of bias within studies	19	Present data on risk of bias of each study and, if available, any outcome level assessment.	8-9 – Appendix 8
Results of individual studies	20	For all outcomes considered (benefits or harms), present, for each study: 1) simple summary data for each intervention group, and 2) effect estimates and confidence intervals. *Modified approaches may be needed to deal with information from larger networks.*	Appendices 6 and 10 (full data can be provided by the first author)
Synthesis of results	21	Present results of each meta-analysis done, including confidence/credible intervals. *In larger networks, authors may focus on comparisons versus a particular comparator (e.g. placebo or standard care), with full findings presented in an appendix. League tables and forest plots may be considered to summarize pairwise comparisons.* If additional summary measures were explored (such as treatment rankings), these should also be presented.	9-11 – Appendix 15
**Exploration for inconsistency**	**S5**	Describe results from investigations of inconsistency. This may include such information as measures of model fit to compare consistency and inconsistency models, *P* values from statistical tests, or summary of inconsistency estimates from different parts of the treatment network.	9 - Appendix 14
Risk of bias across studies	22	Present results of any assessment of risk of bias across studies for the evidence base being studied.	9-11 - Appendix 12
Results of additional analyses	23	Give results of additional analyses, if done (e.g., sensitivity or subgroup analyses, meta-regression analyses*, alternative network geometries studied, alternative choice of prior distributions for Bayesian analyses,* and so forth).	9-11 - Appendices 16 and 17

**DISCUSSION**			
Summary of evidence	24	Summarize the main findings, including the strength of evidence for each main outcome; consider their relevance to key groups (e.g., healthcare providers, users, and policy-makers).	11-13
Limitations	25	Discuss limitations at study and outcome level (e.g., risk of bias), and at review level (e.g., incomplete retrieval of identified research, reporting bias). *Comment on the validity of the assumptions, such as transitivity and consistency. Comment on any concerns regarding network geometry (e.g., avoidance of certain comparisons).*	13-14
Conclusions	26	Provide a general interpretation of the results in the context of other evidence, and implications for future research.	13-14
**FUNDING**			
Funding	27	Describe sources of funding for the systematic review and other support (e.g., supply of data); role of funders for the systematic review. This should also include information regarding whether funding has been received from manufacturers of treatments in the network and/or whether some of the authors are content experts with professional conflicts of interest that could affect use of treatments in the network.	15

PICOS = population, intervention, comparators, outcomes, study design.
* Text in italics indicateS wording specific to reporting of network meta-analyses that has been added to guidance from the PRISMA statement.
† Authors may wish to plan for use of appendices to present all relevant information in full detail for items in this section.

For peer review only

PRISMA 2020 for Abstracts Checklist

Section and Topic	Item #	Checklist item	Reported (Yes/No)
**TITLE**			
Title	1	Identify the report as a systematic review.	Yes
**BACKGROUND**			
Objectives	2	Provide an explicit statement of the main objective(s) or question(s) the review addresses.	Yes
**METHODS**			
Eligibility criteria	3	Specify the inclusion and exclusion criteria for the review.	Yes
Information sources	4	Specify the information sources (e.g. databases, registers) used to identify studies and the date when each was last searched.	Yes
Risk of bias	5	Specify the methods used to assess risk of bias in the included studies.	Yes
Synthesis of results	6	Specify the methods used to present and synthesise results.	Yes
**RESULTS**			
Included studies	7	Give the total number of included studies and participants and summarise relevant characteristics of studies.	Yes
Synthesis of results	8	Present results for main outcomes, preferably indicating the number of included studies and participants for each. If meta-analysis was done, report the summary estimate and confidence/credible interval. If comparing groups, indicate the direction of the effect (i.e. which group is favoured).	Yes
**DISCUSSION**			
Limitations of evidence	9	Provide a brief summary of the limitations of the evidence included in the review (e.g. study risk of bias, inconsistency and imprecision).	Yes
Interpretation	10	Provide a general interpretation of the results and important implications.	Yes
**OTHER**			
Funding	11	Specify the primary source of funding for the review.	Yes
Registration	12	Provide the register name and registration number.	Yes

From: Page MJ, McKenzie JE, Bossuyt PM, Boutron I, Hoffmann TC, Mulrow CD, et al. The PRISMA 2020 statement: an updated guideline for reporting systematic reviews. BMJ 2021;372:n71. doi: 10.1136/bmj.n71

For more information, visit: http://www.prisma-statement.org/

For peer review only

Comparative safety and efficacy of cognitive enhancers for Alzheimer's dementia: A systematic review with individual patient data network meta-analysis

Journal:	*BMJ Open*
Manuscript ID	bmjopen-2021-053012.R2
Article Type:	Original research
Date Submitted by the Author:	17-Nov-2021
Complete List of Authors:	Veroniki, Areti; St. Michael's Hospital, Knowledge Translation Program; Imperial College London, Department of Surgery & Cancer, Faculty of Medicine<br>Ashoor, Huda; St Michael's Hospital, Knowledge Translation Program<br>Rios, Patricia; St Michael's Hospital, Knowledge Translation Program<br>Seitidis, Georgios; University of Ioannina, Department of Primary Education<br>Stewart, Lesley; University of York, Centre for Reviews and Dissemination<br>Clarke, Mike; Queen's University Belfast, Northern Ireland Hub for Trials Methodology Research<br>Tudur-Smith, Catrin; University of Liverpool, Department of Biostatistics<br>Mavridis, Dimitris ; University of Ioannina, Department of Primary Education<br>Hemmelgarn, Brenda; University of Alberta, Department of Medicine<br>Holroyd-Leduc, Jayna; University of Calgary, Department of Medicine<br>Straus, Sharon; St Michael's Hospital, Knowledge Translation Program; University of Toronto, Department of Geriatric Medicine<br>Tricco, Andrea; St Michael's Hospital, Knowledge Translation Program; University of Toronto, Dalla Lana School of Public Health
**Primary Subject Heading**:	Public health
Secondary Subject Heading:	Neurology
Keywords:	STATISTICS & RESEARCH METHODS, EPIDEMIOLOGY, Dementia < NEUROLOGY

SCHOLARONE™
Manuscripts

I, the Submitting Author has the right to grant and does grant on behalf of all authors of the Work (as defined in the below author licence), an exclusive licence and/or a non-exclusive licence for contributions from authors who are: i) UK Crown employees; ii) where BMJ has agreed a CC-BY licence shall apply, and/or iii) in accordance with the terms applicable for US Federal Government officers or employees acting as part of their official duties; on a worldwide, perpetual, irrevocable, royalty-free basis to BMJ Publishing Group Ltd ("BMJ") its licensees and where the relevant Journal is co-owned by BMJ to the co-owners of the Journal, to publish the Work in this journal and any other BMJ products and to exploit all rights, as set out in our licence.

The Submitting Author accepts and understands that any supply made under these terms is made by BMJ to the Submitting Author unless you are acting as an employee on behalf of your employer or a postgraduate student of an affiliated institution which is paying any applicable article publishing charge ("APC") for Open Access articles. Where the Submitting Author wishes to make the Work available on an Open Access basis (and intends to pay the relevant APC), the terms of reuse of such Open Access shall be governed by a Creative Commons licence – details of these licences and which Creative Commons licence will apply to this Work are set out in our licence referred to above.

Other than as permitted in any relevant BMJ Author's Self Archiving Policies, I confirm this Work has not been accepted for publication elsewhere, is not being considered for publication elsewhere and does not duplicate material already published. I confirm all authors consent to publication of this Work and authorise the granting of this licence.

For peer review only

Comparative safety and efficacy of cognitive enhancers for Alzheimer's dementia: A systematic review with individual patient data network meta-analysis

Areti Angeliki Veroniki[1,*] PhD e-mail: areti-angeliki.veroniki@unityhealth.to
Huda M. Ashoor[1] BSc e-mail: huda.ashoor@unityhealth.to
Patricia Rios[1] MSc e-mail: patricia.rios@unityhealth.to
Georgios Seitidis[2] MSc e-mail: g.seitidis@uoi.gr
Lesley A. Stewart[3] PhD e-mail: lesley.stewart@york.ac.uk
Mike Clarke[4] PhD e-mail: m.clarke@qub.ac.uk
Catrin Tudur Smith[5] PhD e-mail: cat1@liverpool.ac.uk
Dimitris Mavridis[2] PhD e-mail: dmavridi@uoi.gr
Brenda R. Hemmelgarn[6] PhD e-mail: brenda.hemmelgarn@albertahealthservices.ca
Jayna Holroyd-Leduc[7] MD e-mail: jayna.holroyd-leduc@albertahealthservices.ca
Sharon E. Straus[1,8] MD e-mail: sharon.straus@utoronto.ca
Andrea C. Tricco[1,9] PhD e-mail: andrea.tricco@unityhealth.to

[1] Knowledge Translation Program, Li Ka Shing Knowledge Institute, St. Michael's Hospital, Toronto, Ontario, Canada

[2] Department of Primary Education, School of Education, University of Ioannina, Ioannina, Greece

[3] Centre for Reviews and Dissemination, University of York, York, United Kingdom

[4] Northern Ireland Hub for Trials Methodology Research, Queen's University Belfast, Belfast, United Kingdom

[5] Department of Biostatistics, University of Liverpool, Block F, Waterhouse Building, 1-5 Brownlow Hill, Liverpool, L69 3GL, UK

[6] Department of Medicine, University of Alberta, Edmonton, Alberta, Canada

[7] Department of Medicine, University of Calgary, Calgary, Alberta, Canada

[8] Department of Geriatric Medicine, University of Toronto, Toronto, Ontario, Canada

[9] Epidemiology Division & Institute of Health Policy, Management, and Evaluation, Dalla Lana School of Public Health, University of Toronto, Toronto, Ontario, Canada

For peer review only

***Corresponding Author**:

Dr. Areti Angeliki Veroniki, MSc, PhD

209 Victoria Street, East Building, Toronto, Ontario

M5B 1T8, Canada

Phone: 416-564-5015; Fax: 416-564-5735; Email: areti-angeliki.veroniki@unityhealth.to

Word count: 4,215 (max 4000); 1 table; 4 figures; 2 additional files (21 appendices in additional file 1); 31 references

For peer review only

For peer review only - http://bmjopen.bmj.com/site/about/guidelines.xhtml

Abstract

Words: 377 (Max 300 words)

Objective: To examine the comparative efficacy and safety of cognitive enhancers by patient characteristics for managing Alzheimer's Dementia (AD).

Design: Systematic review and individual patient data (IPD) network meta-analysis (NMA)

Data Sources: MEDLINE, EMBASE, Cochrane Methodology Register, CINAHL, Ageline and Cochrane Central Register of Controlled Trials up to March 2016.

Participants: 80 randomized controlled trials (RCTs) including 21,138 adults with AD, and 12 RCTs with IPD including 6,906 patients.

Interventions: Cognitive enhancers (donepezil, rivastigmine, galantamine and memantine) alone or in any combination against other cognitive enhancers or placebo.

Data extraction and Synthesis: We requested IPD from authors, sponsors and data sharing platforms. When IPD were not available, we used aggregate data. We appraised study quality with the Cochrane risk-of-bias. We conducted a two-stage random-effects IPD-NMA, and assessed their findings using CINeMA (Confidence in Network meta-analysis).

Primary and Secondary Outcomes: We included trials assessing cognition with the Mini-Mental State Examination (MMSE), and adverse events (AEs).

Results: Our IPD-NMA compared 9 treatments (including placebo). Donepezil (mean difference [MD] = 1.41, 95% confidence interval [CI]: 0.51 to 2.32) and donepezil+memantine (MD = 2.57, 95% CI: 0.07 to 5.07) improved MMSE score (56 RCTs, 11,619 participants; CINeMA score: moderate) compared with placebo. According to P-score, oral rivastigmine (odds ratio [OR] = 1.26, 95% CI: 0.82 to 1.94, P-score= 16%) and donepezil (OR = 1.08, 95% CI: 0.87 to 1.35, P-score= 30%) had the least favourable safety profile, but none of the estimated treatment effects were sufficiently precise when compared with placebo (45 RCTs, 15,649 patients; CINeMA score: moderate to high). For moderate to severe impairment, donepezil, memantine and their combination performed best, but for mild to moderate impairment donepezil and transdermal rivastigmine ranked best. Adjusting for MMSE baseline differences, oral rivastigmine and galantamine

For peer review only - http://bmjopen.bmj.com/site/about/guidelines.xhtml

improved MMSE score, whereas when adjusting for comorbidities only oral rivastigmine was effective.

Conclusions: The choice among the different cognitive enhancers may depend on patient's characteristics. The MDs of all cognitive enhancer regimens except for single-agent oral rivastigmine, galantamine, and memantine, against placebo were clinically important for cognition (MD larger than 1.40 MMSE points), but results were quite imprecise. However, two thirds of the published RCTs were associated with high risk of bias for incomplete outcome data, and IPD were only available for 15% of the included RCTs.

Registration: PROSPERO # CRD42015023507

Funding: This research was funded by the CIHR Drug Safety and Effectiveness Network (grant number 137713).

Keywords: network meta-analysis; multiple treatments meta-analysis; individual participant data; Nootropic Agents; Alzheimer Disease

Strengths and limitations of this study

- This is one of the most comprehensive systematic reviews and network meta-analysis of cognitive enhancers including individual patient data for Alzheimer's Dementia to produce treatment recommendations by patient characteristics.
- We followed the methodologically rigorous guidelines in the Cochrane Handbook for systematic reviews, and the CINeMA quality assessment guidelines.
- Access to individual patient data allowed us to 1) observe minor differences between the original published results and our re-analysis, potentially due to differences in imputation methods for missing data or because original studies have excluded some patients, and hence have used a smaller sample size, 2) overcome potential reporting bias, and 3) assess for potential effect modifiers that were not reported in the original publications (e.g., comorbidities, additional medications) and explore for treatment-by-covariate interactions on the patient-level.
- Two thirds of the included RCTs, were associated with high risk of bias for incomplete outcome data due to attrition.
- We were unable to include individual patient data for all RCTs (only 15% of the studies shared their individual patient data), highlighting potential availability bias.

For peer review only - http://bmjopen.bmj.com/site/about/guidelines.xhtml

For peer review only

Introduction

Alzheimer's dementia (AD) is the most common type of dementia.[1] Patients living with AD have a lower quality of life due to deterioration in function, cognition, behavior, and mental health over time, as well as increased mortality.[2] Pharmacological treatment for AD predominantly consists of cholinesterase inhibitors (donepezil, galantamine, rivastigmine) and the N-methyl-d-aspartate (NMDA) receptor antagonist, memantine. All three cholinesterase inhibitors and memantine are currently the only effective licensed treatments for dementia,[3] but their clinical effect can be small and there is no convincing evidence that they modify the disease process in AD.[4] Also, it is unclear whether galantamine, rivastigmine, or donepezil should be used by patients with severe AD, or whether memantine is the optimal treatment for severe AD.[5]

In AD, disease severity and sex are potential effect modifiers. However, aggregate data and covariates of interest (e.g., sex, disease severity) are not consistently reported across randomized clinical trials (RCTs).[6] The use of IPD has several advantages, such as it allows for the exploration of the relationship between treatment effects and patient-level characteristics, and it overcomes restrictions in using the information reported in the publication among others. The aim of this study was to examine the comparative efficacy and safety of cognitive enhancers for patients with different characteristics, such as severities of AD and for females versus males through a systematic review and individual patient data (IPD) NMA. NMA is an extension of standard meta-analysis synthesizing different sources of evidence from a network of RCTs comparing different treatments within a single model. NMA can provide treatment effect estimates for treatment comparisons that have not studied in a head-to-head study.

Methods

We reported our results according to the Preferred Items for Systematic Reviews and Meta-analysis (PRISMA) Statement for NMA and PRISMA-IPD.[7,8]

Protocol

For peer review only - http://bmjopen.bmj.com/site/about/guidelines.xhtml

The research question and protocol were based on our previous systematic review and NMA.[6] We registered our systematic review protocol with the prospective register of systematic reviews (PROSPERO: CRD42015023507), and published our protocol.[9] Additional information is also provided in Appendix 1 and Additional File 2. Herein, we briefly summarize our methods.

Eligibility criteria

We updated our previous systematic review,[6] using similar population, interventions, comparators, study designs and time period (PICOST) criteria. The literature search was updated from January 2015 to March 2016. We included published and English RCTs that assessed cognition via the Mini-Mental State Examination (MMSE; efficacy and primary outcome) and/or adverse events (AE; safety outcome) in adults with Alzheimer's dementia.

IPD collection process

We contacted the corresponding author followed by the next-in-order author, as presented in each eligible RCT, to obtain IPD. The author contact process was part of a RCT that our team conducted to assess methods that may optimize response rates for IPD retrieval.[10] We also contacted sponsors of eligible trials, as reported in the publications. We contacted industry sponsors only, as we were not able to locate contact information for the majority of non-industry sponsors (e.g., grants and university funding). If a study had multiple sponsors, we contacted all of them. To further facilitate IPD access, we contacted the Clinical Study Data Request (CSDR)[11] and Yale University Open Data Access (YODA) data sharing platforms.[12] If a data provider was unable to provide IPD we noted the reason.

Risk of bias and quality appraisal

We appraised study quality using the Cochrane risk of bias tool.[13] To ensure data consistency[8] we compared IPD with aggregate data reported in the publication. We assessed whether

For peer review only - http://bmjopen.bmj.com/site/about/guidelines.xhtml

randomization of patients was adequate (i.e., intervention and comparison groups were balanced for important patient characteristics), by comparing numbers and types of patients in each arm.

When at least 10 studies were available for each treatment against placebo, publication bias and small-study effects were examined visually using the comparison adjusted funnel plot under the fixed-effect model.[3] When a funnel plot asymmetry was detected, we performed the Copas selection for the treatment comparisons that were informed by at least 10 studies and for which asymmetry was evident in the funnel plot. We explored the possibility that this was due to publication bias,[14] and made moderate assumptions about the probability of publication of the smaller and larger (in terms of standard error) studies. We assumed that the smallest study had a probability of publication equal to 40-50% and the largest study had a probability of 80-90%. Confidence in NMA findings was assessed for each outcome using CINeMA (Confidence in Network meta-analysis, see Appendix 1 for more details).[15]

Synthesis

We performed a descriptive analysis using frequencies and distributions of the characteristics of the included patients and treatments. For each outcome, we present the network geometry according to IPD availability. We conducted a two-stage IPD analysis, whereby data were analysed separately in each trial in the first stage and the trial parameter estimates were synthesised in a random-effects meta-analysis or NMA in the second stage.

The summary treatment effects are presented using the odds ratio (OR) or mean difference (MD) along with their corresponding CIs and prediction intervals (PIs).[16] We ranked the interventions for each outcome using the P-scores (and SUCRAs [surface under the cumulative ranking curve] in meta-regression analysis), and present them in a rank-heat plot.[17,18]

Patient and public involvement

Not applicable.

For peer review only - http://bmjopen.bmj.com/site/about/guidelines.xhtml

Results

Literature search, study selection and IPD obtained

After screening 20,410 titles and abstracts and 1,968 full-text articles, 96 studies fulfilled the eligibility criteria; 80 unique studies and 16 companion reports (Figure 1a, Appendix 2).

(Figure 1 here)

Of the 80 RCTs, 55 reported at least one industry-sponsored funder (i.e. 40 studies reported a single industry-sponsor and 15 multiple industry-sponsors). In the remaining studies, 9 were publicly-sponsored and 16 did not report any information about funding. We requested IPD by contacting the corresponding authors for 80 RCTs that included 21,138 participants. None of the original authors shared their IPD. Fifteen commercial sponsors were then contacted and 6 (40%) sponsors shared their data through proprietary sponsor-specific platforms. The 6 sponsors were contacted for 46 RCTs (14,580 participants), and we obtained IPD for 30% (14 RCTs, 8,007 participants) of these RCTs (1,058 total waiting days up to March 9, 2020). The study flow for obtaining IPD is depicted in Figure 1b.

We were able to include 12 (6,906 patients) of 14 RCTs in our NMA due to incompleteness of provided IPD (Appendix 3). The number of studies with available/non-available IPD from each data provider along with reasons for non-availability of IPD are presented in Appendix 4.

Study and patient characteristics

Most included studies (33%) were multi-national. The mean age of patients ranged from 61 to 86 years. The majority of the RCTs included patients with mild-moderate AD (56%), although the diagnostic criteria used for AD varied widely (Table 1). The most frequent longest duration of follow-up was 24 weeks (24 RCTs, 30%; Appendix 5). Important patient characteristics, such as percent of male and dropout rates, were not balanced across groups in the RCTs with provided IPD (Appendix 6). Comparing study and patient characteristics of available and non-available

For peer review only - http://bmjopen.bmj.com/site/about/guidelines.xhtml

IPD when a study was industry-sponsored, we found differences in the year of study publication, study size, and absolute mean difference (Appendix 7).

(Table 1 here)

Risk of bias and IPD integrity

Using the Cochrane risk-of-bias tool, allocation concealment was at low risk of bias for 43% and blinding of participants and personnel was low for 64% of the RCTs (Appendix 8). One third of the RCTs had low risk of incomplete outcome data bias due to attrition and almost two thirds had high potential risk of "other" bias, specifically, funding bias. The other risk of bias item was scored as unclear for 32%. Overall risk of bias was comparable in studies with available and unavailable IPD (Appendix 9).

All IPD provided were checked for consistency and results from published RCTs were reproduced and provided in Appendix 10. High dropout rates were observed in the IPD; experiencing an adverse event was the most common reason for dropout. Despite the high dropout rates observed in the individual studies, there was no indication of correlation between age and dropout (Appendix 11). Comparison-adjusted funnel plot for MMSE suggested there is indication for small-study effects (see Appendix 12). In contrast to the standard meta-analysis (MD=1·65 95% CI (0·16, 3·14)), the Copas selection model estimated a pooled treatment effect for donepezil vs. placebo MD=1·87 95% CI (1·55, 2·20) with between-study variance τ^2= 1·95, and correlation coefficient -0·45 (-0·76, -0·01) reflecting the belief that the propensity for publication was associated with the observed effect size.

Network meta-analysis

In both MMSE and AE outcomes, on average there were no important concerns regarding the transitivity and consistency assumptions (Appendices 13 and 14; design-by-treatment interaction model MMSE: χ^2= 4·36, 13 degrees of freedom (df), p-value= 0·987; AE: χ^2= 3·57, 6 df, p-

For peer review only - http://bmjopen.bmj.com/site/about/guidelines.xhtml

value= 0·735). Below we present the main analysis results compared to placebo. Additional analyses are presented in Appendix 15-16). The network geometry is presented in Figure 2.

(Figure 2 here)

Cognition

The NMA for MMSE included 56 RCTs, 9 treatments (including placebo), and 11,619 participants. Nine RCTs (3,625 patients) contributed IPD and 47 RCTs (7,994 patients) contributed aggregated data to the NMA. Two studies[19,20] did not report MMSE in the final publication, but in the retrieved IPD we were able to use data for this outcome.

NMA of studies with IPD and aggregate data

Studies in this NMA compared all available treatments. Donepezil (MD= 1·41, 95% CI: 0·51 to 2·32) and donepezil+memantine (MD= 2·57, 95% CI: 0·07 to 5·07) were superior to placebo in terms of MMSE score (Appendix 15). Transdermal rivastigmine (MD= 2·11, 95% CI: -0·04 to 4·26), and the combinations donepezil+memantine, galantamine+memantine (MD= 2·24, 95% CI: -2·13 to 6·61), and transdermal rivastigmine+memantine (MD= 1·79, 95% CI: -1·70 to 5·27) were associated with a MD from placebo of more than 1.40 MMSE points. A previous study suggested a MD larger than 1·40 is a minimal clinically important difference (MCID).[21] However, the associated 95% CIs were quite imprecise spanning between a mean decrease below and a mean increase above the suggested MCID value (Figure 3a). However, donepezil+memantine had the highest likelihood of being the most effective in improving MMSE score (P-score range 79-80%, Figure 4). Confidence in NMA results was moderate (Appendix 17).

(Figure 3 here)

(Figure 4 here)

NMA of studies with aggregate data

For peer review only - http://bmjopen.bmj.com/site/about/guidelines.xhtml

Studies in this NMA compared all available treatments. Donepezil improved MMSE score significantly (MD= 1·55 95% CI: 0·41 to 2·68). Assuming an MCID of 1·40, results were in agreement with the NMA of IPD and aggregate data, and donepezil+memantine (MD= 2·71, 95% CI: -0·17 to 5·60) was likely the most effective in improving MMSE score (P-score= 76%).

NMA of studies with IPD

Studies in this NMA compared placebo, donepezil, oral rivastigmine, transdermal rivastigmine, galantamine, and memantine. Donepezil (MD= 0·70, 95% CI: 0·01 to 1·40) and transdermal rivastigmine (MD= 1·06, 95% CI: 0·04 to 2·08) were superior to placebo, but none of the point estimates reached a previously suggested MCID.[21] The most effective treatment was likely transdermal rivastigmine (P-score= 82%).

Additional analyses using IPD and aggregate data

Overall, additional analyses using both IPD and aggregate data were in agreement with the findings of the main analysis (Appendix 16). Cognitive performance was better in patients with mild to moderate MMSE receiving donepezil (MD= 1·68 95% CI: 0·31 to 3·06, P-score= 69%) and most likely when receiving transdermal rivastigmine (MD= 2·74 95% CI: -0·68 to 6·16, P-score= 81%). In patients with moderate to severe MMSE the combination donepezil+memantine improved MMSE score significantly (MD= 2·49 95% CI: 1·55 to 3·44, P-score=100%), but oral rivastigmine deteriorated MMSE score significantly (MD= -1·00 95% CI: -1·87 to -0·12, P-score= 4%). Donepezil (MD= 1·31 95% CI: 0·66 to 1·96, P-score= 78%) and memantine (MD=0·69 95% CI: 0·07 to 1·31, P-score= 59%) also performed well for patients with moderate to severe cognitive impairment.

Accounting for the impact of the outlier studies, galantamine+memantine was the second-best cognitive enhancer (MD= 1·87 95% CI: 0·08 to 3·66, P-score=82%) after donepezil+memantine (MD= 2·04 95% CI: 1·03 to 3·05, P-score= 92%). Using only IPD adjusted for comorbidities suggested that oral rivastigmine improves MMSE score (MD= 0·88 95% CI: 0·31 to 1·45, P-score= 75%). Similarly, using IPD adjusted for cognitive impairment assessed with MMSE at baseline suggested that oral rivastigmine (MD= 0·88 95% CI: 0·31 to 1·45, P-score= 69%) and

For peer review only - http://bmjopen.bmj.com/site/about/guidelines.xhtml

galantamine (MD= 0·76 95% CI: 0·34 to 1·18, P-score= 62%) improve MMSE score, but in a future study, results are only stable for galantamine.

Heterogeneity in NMA was high (between-study variance = 5·75, I^2= 96%) compared also to the Rhodes et *al.*[22] empirical distribution (median 0.05, 95% range: 0·00 to 7·56). However, heterogeneity decreased importantly when excluding outliers (between-study variance = 0·59, I^2= 73%), including only patients with moderate to severe AD (between-study variance = 0·18, I2= 44%), restricting to industry-sponsored trials (between-study variance = 0·16, I^2= 43%), and using IPD only (between-study variance = 0·12, I^2= 29%).

Adverse events

A NMA was conducted on adverse events (study definitions are provided in Appendix 18) with 45 RCTs, 9 treatments (including placebo), and 15,649 patients (Figure 2b). In particular, 12 RCTs (6420 patients) contributed to the NMA using their IPD and 33 RCTs (9229 patients) using their data on their aggregated form. The time taken to achieve at least one AE was available in 8 studies with available IPD and ranged between 45 and 2228 days (Appendix 19). Only one study included a patient with a AE occurring earlier than the trial opening and was excluded from the study.[23]

NMA of studies with IPD and aggregate data

Studies in this NMA compared all available treatments. According to P-score, oral rivastigmine had the least favourable safety profile regarding AE (OR= 1·26, 95% CI: 0·82 to 1·94, P-score= 16%), followed by donepezil (OR= 1·08, 95% CI: 0·87 to 1·35, P-score= 30%) and galantamine+memantine (OR= 1·03, 95% CI: 0·45 to 2·39, P-score= 43%), yet in these comparisons the odds of experiencing an AE were imprecise and not importantly different from placebo (Figure 3b; Appendices 16, 20). Confidence in NMA results ranged between moderate and high (Appendix 17).

NMA of studies with aggregate data

For peer review only - http://bmjopen.bmj.com/site/about/guidelines.xhtml

Studies in this NMA compared all available treatments. Results were mainly consistent with NMA of IPD and aggregate data, but memantine was 0·70 times less likely to experience an AE than placebo, with an OR ranging from 0·51 to 0·97 (P-score= 77%).

NMA of studies with IPD

Studies in this NMA compared placebo, donepezil, oral rivastigmine, transdermal rivastigmine, galantamine, and memantine. Results were on average consistent with NMA of IPD and aggregate data.

Additional analyses using IPD and aggregate data

Additional analyses using both IPD and aggregate data, showed that memantine was 0·61 times less likely to experience an AE than placebo when using study duration as a covariate, with an OR ranging from 0·37 to 0·93 (P-score= 88%). Restricting to low risk of bias for incomplete outcome data, galantamine was associated with significantly lower odds of a AE (OR= 0·69, 95% CI: 0·50 to 0·97, P-score= 80%).

Heterogeneity in NMA was low (between-study variance = 0·04, I^2= 22%) compared to the Turner et *al.*[24] empirical distribution (median 0·12, 95% range: 0·01 to 2·63). Heterogeneity decreased importantly when restricting to aggregate data (between-study variance = 0·00, I^2= 0%), low risk of bias for incomplete outcome data (between-study variance = 0·02, I^2= 10%), patients with moderate to severe cognitive impairment (between-study variance = 0·00, I^2= 0%), and when adjusting for study duration (between-study variance = 0·03), year of publication (between-study variance = 0·02), mean age (between-study variance = 0.02) or sex (between-study variance = 0·03).

Discussion

We compared the efficacy and safety of cognitive enhancers regarding MMSE and AE outcomes to update our previous systematic review[6] and included studies with both aggregate data and IPD. Our results are in agreement with our previous systematic review,[6] and show that

For peer review only - http://bmjopen.bmj.com/site/about/guidelines.xhtml

donepezil+memantine, donepezil alone and transdermal rivastigmine were the most effective treatments for improving MMSE score. However, heterogeneity was a major concern, which requires careful consideration before suggesting the use of cognitive enhancers, and particularly when the efficacy is not clear on the patient's characteristics. This was also captured by PIs, but their interpretation requires caution due to evidence of funnel plot asymmetry in the MMSE outcome. Overall, PIs are expected to include the true intervention effect expected in future studies, and they incorporate an extra component of variance, specifically between-study heterogeneity. In the absence of heterogeneity, confidence intervals and PIs are equal. According to the P-score intervention ranking, both donepezil+memantine and transdermal rivastigmine had a favourable safety profile regarding AE, whereas the therapy with the least favourable profile was oral rivastigmine followed by donepezil. However, none of the estimated treatment effects were sufficiently precise when cognitive enhancers were compared with the placebo group. CINeMA suggested that within-study bias and reporting bias were the highest concerns for the MMSE outcome, whereas within-study bias and imprecision of effect estimates were the highest concerns for the AE outcome.

Overall, the choice among the different cognitive enhancers may depend on the patient's characteristics. In participants with moderate to severe cognitive impairment (defined by MMSE), a larger improvement in cognitive performance was observed for donepezil and memantine, and their combination (donepezil+memantine), and these efficacy-related results are expected to also be reflected when a future study becomes available. The least effective cognitive enhancer in participants with moderate to severe cognitive impairment was oral rivastigmine. For patients with mild to moderate impairments based on MMSE scores, donepezil and transdermal rivastigmine were most likely the best performing cognitive enhancers. For patients with moderate to severe cognitive impairment, cognitive enhancers were well tolerated. For patients with mild to moderate cognitive impairment, all except for memantine and its combination with transdermal rivastigmine, were associated with increased odds of an AE, yet none of these results reached statistical significance. Overall, memantine was associated with lower odds of an AE than placebo, yet this was statistically significant only in the subnetwork analysis including aggregate data (i.e., studies without IPD) and the meta-regression analysis using study duration as a covariate. However, acknowledging for heterogeneity in the network,

For peer review only - http://bmjopen.bmj.com/site/about/guidelines.xhtml

PIs suggested that results are inconclusive and the odds of AE could not be differentiated between memantine and placebo. Of note, the accuracy of AE reporting may be impacted by the degree of cognitive impairment. Using IPD only and adjusting for MMSE baseline differences, (as shown in Appendix 16, Mean Difference: NMA of studies with IPD adjusted for baseline cognitive impairment), oral rivastigmine and galantamine improved MMSE score, whereas when adjusting for comorbidities only oral rivastigmine was effective, but results can change in a future study. Considering a MCID equal to 1·40 points,[21] the MDs of all cognitive enhancer regimens except for single-agent oral rivastigmine, galantamine, and memantine, against placebo were clinically important for cognition, but these were associated with high uncertainty. However, the 1·40 MMSE cut-off value is not a widely adopted MCID. Our results did not differ by participant characteristics sex, age, and other medications, or by study characteristics, study duration and year of publication. However, these findings might be due to low power since meta-regression analyses depend on the number and size of studies, magnitude of the relationship between the covariate and effect size, along with its precision and heterogeneity.[25]

To the best of our knowledge, our study was the first to add IPD in a NMA of cognitive enhancers for patients with Alzheimer's Dementia to produce treatment recommendations by patient characteristics. We followed the methods guidelines in the Cochrane Handbook for systematic reviews,[26] the reporting guidelines in the PRISMA-NMA and PRISMA-IPD statements,[7,8] and the CINeMA quality assessment guidelines.[15] Compared to previous systematic reviews, we included a larger number of studies and/or studies with shared IPD, compared in a wider range of cognitive enhancers.[6,27] Our results are in agreement with previous studies overall. Access to IPD allowed us to observe minor differences between the original published results and our re-analysis. An explanation in these differences may be that many studies used the last-observation-carried-forward imputation method, whereas we used the available case analysis when assessing MMSE. Another potential explanation might be that original studies excluded some patients, and hence used a smaller sample size.

Comparing NMA, results between aggregate data and IPD were in agreement. The only difference was observed in transdermal rivastigmine that was associated with a MCID of greater than 1·40 MMSE points against placebo in the aggregate data NMA compared to the IPD NMA,

For peer review only - http://bmjopen.bmj.com/site/about/guidelines.xhtml

yet a statistically significant improvement was achieved in the IPD NMA. The inclusion of IPD in our NMA, allowed us to overcome potential reporting bias and to include IPD for 1) a study that we previously were unable to include since arm-level data were not reported in the RCT publication,[23] and 2) two studies that did not report MMSE results in their publications.[19,20] The use of IPD also allowed us to assess for potential effect modifiers that were not reported in the original publications (e.g., comorbidities, additional medications) and explore for treatment-by-covariate interactions on the patient-level. Several challenges were encountered during the IPD request from sponsors, showing that repositories are not a panacea (Appendix 21).

An important finding of our review is that the two thirds of the published RCTs, were associated with high risk of bias for incomplete outcome data due to attrition, and the majority of these RCTs used the last-observation-carried-forward technique for missing data. This approach may bias results favouring cognitive enhancers, since the dropout rates were greater in the treatment group compared to the placebo group in 63% of the included studies and because dementia is a progressive disease. Of the 27 studies comparing treatment against placebo and reporting the number of dropouts, 17 studies had a greater dropout rate in the treatment group (treatment group: median dropout rate= 28% IQR [17% to 39%]; placebo group: median dropout rate= 21% IQR [15% to 31%]). Last-observation-carried-forward is an inappropriate imputation method for Alzheimer's Dementia studies, since it ignores expected deterioration of the patient's condition and stabilizes the outcome at the value observed at the time of dropout (i.e., the last observation).[28] Restricting to low risk of attrition bias studies, we found that galantamine was significantly associated with decreased odds of experiencing an AE.

Our study has limitations worth mentioning. First, we were unable to include IPD for all eligible studies (only 15% of the included RCTs shared their IPD), highlighting potential availability bias for IPD. However, recent simulations have shown that combining IPD and aggregate data in a NMA can significantly improve precision, reduce bias, and increase information compared to NMA relying on aggregated data alone.[29] Second, missing data is a big concern in the published RCTs for AD. We found high rates of dropouts from experiencing an adverse event and the patients' characteristics that may increase the chances of such adverse reactions prior to administering these cognitive enhancers should further be explored. To assess the impact of

For peer review only - http://bmjopen.bmj.com/site/about/guidelines.xhtml

missing data in our NMA, we applied the informative missingness of difference in means.[30] However, future studies should explore the characteristics of missing participants and specific adverse events. Third, the lack of studies in certain treatment comparisons may have affected the P-score calculation and treatment ranking. In particular, polytherapies were informed by maximum two studies, and ranking may have been in favour of the complex intervention group with the smaller number of studies.[31] For example, in MMSE the polytherapies including memantine in conjunction with one of the three treatments donepezil, galantamine, transdermal rivastigmine had a P-score ≥60%, but these all had wide 95% CIs for MD. As such, ranking should be interpreted with caution and along with the estimated effect sizes and their uncertainty measures. Fourth, the comparison-adjusted funnel plot for MMSE suggested there is an indication for small-study effects pointing to the treatment being better, and results should be interpreted with caution. This may also be related to the potential risk of funding bias, since the majority of the included studies were industry-sponsored and IPD were retrieved only from industry-sponsored studies favouring cognitive enhancers over placebo. Overall, MMSE score is only a surrogate maker for determining the impact of treatments on dementia. A full assessment that considers the potential impact of treatments on cognition, function and behavioural symptoms needs to be considered within the clinical context. Fifth, differences in patient characteristics, such as sex, were observed in the RCTs with provided IPD, which increased heterogeneity across studies. To account for these differences, we used the fully adjusted treatment effect estimates in the IPD analyses and the primary NMA analysis. Also, at the NMA level, we found that on average there were no important differences across treatment comparisons to threaten the transitivity assumption. Sixth, there are clinically important limitations associated with this review, including consistent definition of outcome measures across studies, a well-established MCID for the MMSE score, lack of consideration of drug doses due to inconsistent reporting and data availability bias that we were unable to overcome (15% of the studies shared their IPD). Future studies are needed to establish ranking efficacy in drug doses and combination of interventions across different disease severity categories. Seventh, the literature searches were conducted 5 years ago and additional relevant studies may be available. However, obtaining IPD in a timely manner was very challenging and required more time than anticipated (challenges to obtain IPD are outlined in Appendix 21). Similar to all systematic reviews, the evidence should be regularly updated.

For peer review only

We expect that our findings will increase scientific knowledge, because people with Alzheimer's Dementia require personalized medicine to optimize their healthcare. Well-conducted meta-analyses of IPD are considered the 'gold-standard' and influence patient care since patient-level data can be provided to facilitate tailored decision making. However, results from meta-analyses of IPD are likely subject to retrieval bias and awareness of these limitations and their potential impact on findings is required.

For peer review only

For peer review only - http://bmjopen.bmj.com/site/about/guidelines.xhtml

Contributors

AAV, SES and ACT conceived and designed the study.

AAV conducted the analyses, abstracted data, contacted sponsors, analysed data, interpreted results, appraised quality of results, and wrote a draft manuscript.

GS conducted the analyses, appraised quality of results, and edited the manuscript.

HMA coordinated the review, screened citations and full-text articles, abstracted data, appraised quality, cleaned the data, contacted sponsors, and edited the manuscript.

PR helped coordinate the study, screened citations and full-text articles, extracted and categorized data, appraised quality, and edited the manuscript.

SES and ACT interpreted results and edited the manuscript.

ACT and HMA contacted authors. LAS, MC, CTS, DM, BRH, JHL provided input into the design, interpreted results, and edited the manuscript.

All authors read and approved the final manuscript.

Declaration of interests

The authors declare that they have no competing interests.

Data sharing statement

All data relevant to the study are included in the article or uploaded as supplementary information.

Funding

This research was funded by the CIHR Drug Safety and Effectiveness Network (grant number 137713). The funder contributed to defining the scope of the systematic review but otherwise had no role in study design, data collection, analysis, and interpretation of data. Data sharing sponsors were provided the ability to review at the time of submission of the manuscript for publication.

For peer review only

493 AAV was previously funded by the Canadian Institutes of Health Research (CIHR) Banting Postdoctoral Fellowship Program [No. 139157]. GS and DM were funded from the European Union's Horizon 2020 [No. 754936]. SES is funded by a Tier 1 Canada Research Chair in Knowledge Translation [No. N/A]. ACT is funded by a Tier 2 Canada Research Chair in Knowledge Synthesis [No. N/A].

Open Access

This is an open access article distributed in accordance with the Creative Commons Attribution Non Commercial (CC BY-NC 4.0) license, which permits others to distribute, remix, adapt, build upon this work non-commercially, and license their derivative works on different terms, provided the original work is properly cited, appropriate credit is given, any changes made indicated, and the use is non-commercial. See: http:// creativecommons. org/ licenses/ by- nc/ 4. 0/.

Acknowledgements

We thank Laure Perrier for conducting the initial literature search, Alissa Epworth for updating it, and Becky Skidmore for peer reviewing the literature search. We thank Robert Peterson for his support on this study as a knowledge user. We would also like to thank Paul A. Khan, Fatemeh Yazdi, Marco Ghassemi, John D. Ivory, Charlene Soobiah, Erik Blondal, Joanne M. Ho, Shirra Berliner, and Carmen H. Ng for screening some of the citations, data abstracting included studies, or both. We thank Susan Le for helping with contacting the authors and sponsors of the included studies in this review. Finally, we thank Susan Le and Shazia Siddiqui for formatting the manuscript and assisting with the submission.

We would also like to thank the following sponsors for sharing the data with us:

- "This publication is based on research using data from data contributors, AbbVie, Inc, that has been made available through Vivli, Inc. Vivli has not contributed to or approved, and is not in any way responsible for, the contents of this publication."

For peer review only - http://bmjopen.bmj.com/site/about/guidelines.xhtml

- "This study, carried out under YODA Project #2017-1671, used data obtained from the Yale University Open Data Access Project, which has an agreement with JANSSEN RESEARCH & DEVELOPMENT, L.L.C.. The interpretation and reporting of research using this data are solely the responsibility of the authors and does not necessarily represent the official views of the Yale University Open Data Access Project or JANSSEN RESEARCH & DEVELOPMENT, L.L.C.."
- This publication used data obtained from Eisai, GlaxoSmithKline, and Novartis carried under www.ClinicalStudyDataRequest.com
- This publication used data obtained from Lundbeck

Ethical Approval Statement

Not applicable.

For peer review only

References

1. Health NCCfM. Dementia: A NICE-SCIE guideline on supporting people with dementia and their carers in health and social care. 2007: British Psychological Society; 2007.
2. Dudgeon S. Rising Tide: The Impact of Dementia on Canadian Society: a Study. 2010: Alzheimer Society of Canada; 2010.
3. O'Brien JT, Holmes C, Jones M, et al. Clinical practice with anti-dementia drugs: A revised (third) consensus statement from the British Association for Psychopharmacology. *Journal of psychopharmacology (Oxford, England)* 2017; **31**(2): 147-68.
4. National Institute for Health and Care Excellence (UK). Dementia: Assessment, management and support for people living with dementia and their carers. 2018. Available from: https://www.ncbi.nlm.nih.gov/books/NBK536484/.
5. National Institute for Health and Clinical Excellence. Donepezil, galantamine, rivastigmine and memantine for the treatment of Alzheimer's disease. London, UK, 2011.
6. Tricco AC, Ashoor HM, Soobiah C, et al. Comparative Effectiveness and Safety of Cognitive Enhancers for Treating Alzheimer's Disease: Systematic Review and Network Metaanalysis. *J Am Geriatr Soc* 2018; **66**(1): 170-8.
7. Hutton B, Salanti G, Caldwell DM, et al. The PRISMA extension statement for reporting of systematic reviews incorporating network meta-analyses of health care interventions: checklist and explanations. *Annals of internal medicine* 2015; **162**(11): 777-84.
8. Stewart LA, Clarke M, Rovers M, et al. Preferred Reporting Items for Systematic Review and Meta-Analyses of individual participant data: the PRISMA-IPD Statement. *Jama* 2015; **313**(16): 1657-65.
9. Veroniki AA, Straus SE, Ashoor HM, et al. Comparative safety and effectiveness of cognitive enhancers for Alzheimer's dementia: protocol for a systematic review and individual patient data network meta-analysis. *BMJ open* 2016; **6**(1): e010251.
10. Veroniki AA, Ashoor HM, Le SPC, et al. Retrieval of individual patient data depended on study characteristics: a randomized controlled trial. *Journal of clinical epidemiology* 2019; **113**: 176-88.
11. So D, Knoppers BM. Ethics approval in applications for open-access clinical trial data: An analysis of researcher statements to clinicalstudydatarequest.com. *PLoS One* 2017; **12**(9): e0184491.
12. Krumholz HM, Waldstreicher J. The Yale Open Data Access (YODA) Project--A Mechanism for Data Sharing. *N Engl J Med* 2016; **375**(5): 403-5.
13. Higgins JP, Altman DG, Gotzsche PC, et al. The Cochrane Collaboration's tool for assessing risk of bias in randomised trials. *Bmj* 2011; **343**: d5928.
14. Mavridis D, Sutton A, Cipriani A, Salanti G. A fully Bayesian application of the Copas selection model for publication bias extended to network meta-analysis. *Statistics in medicine* 2013; **32**(1): 51-66.
15. CINeMA: Confidence in Network Meta-Analysis. Institute of Social and Preventive Medicine, University of Bern. *Available from cinemaispmch* 2017.
16. Riley RD, Higgins JP, Deeks JJ. Interpretation of random effects meta-analyses. *Bmj* 2011; **342**: d549.
17. Veroniki AA, Straus SE, Fyraridis A, Tricco AC. The rank-heat plot is a novel way to present the results from a network meta-analysis including multiple outcomes. *Journal of clinical epidemiology* 2016; **76**: 193-9.
18. Rucker G, Schwarzer G. Ranking treatments in frequentist network meta-analysis works without resampling methods. *BMC Med Res Methodol* 2015; **15**: 58.
19. Herrmann N, Gauthier S, Boneva N, Lemming OM. A randomized, double-blind, placebo-controlled trial of memantine in a behaviorally enriched sample of patients with moderate-to-severe Alzheimer's disease. *International psychogeriatrics* 2013; **25**(6): 919-27.

For peer review only - http://bmjopen.bmj.com/site/about/guidelines.xhtml

20. Burns A, Bernabei R, Bullock R, et al. Safety and efficacy of galantamine (Reminyl) in severe Alzheimer's disease (the SERAD study): a randomised, placebo-controlled, double-blind trial. *The Lancet Neurology* 2009; **8**(1): 39-47.
21. Howard R, Phillips P, Johnson T, et al. Determining the minimum clinically important differences for outcomes in the DOMINO trial. *Int J Geriatr Psychiatry* 2011; **26**(8): 812-7.
22. Rhodes KM, Turner RM, Higgins JP. Predictive distributions were developed for the extent of heterogeneity in meta-analyses of continuous outcome data. *Journal of clinical epidemiology* 2015; **68**(1): 52-60.
23. Gold M, Alderton C, Zvartau-Hind M, et al. Rosiglitazone monotherapy in mild-to-moderate Alzheimer's disease: results from a randomized, double-blind, placebo-controlled phase III study. *Dementia and geriatric cognitive disorders* 2010; **30**(2): 131-46.
24. Turner RM, Davey J, Clarke MJ, Thompson SG, Higgins JP. Predicting the extent of heterogeneity in meta-analysis, using empirical data from the Cochrane Database of Systematic Reviews. *International journal of epidemiology* 2012; **41**(3): 818-27.
25. Hedges L, Higgins J, Rothstein H. Introduction to Meta-Analysis. John Wiley & Sons; 2009.
26. Higgins JP, Thomas J, Chandler J, et al. Cochrane handbook for systematic reviews of interventions: John Wiley & Sons; 2019.
27. Cui CC, Sun Y, Wang XY, Zhang Y, Xing Y. The effect of anti-dementia drugs on Alzheimer disease-induced cognitive impairment: A network meta-analysis. *Medicine* 2019; **98**(27): e16091.
28. Molnar FJ, Hutton B, Fergusson D. Does analysis using "last observation carried forward" introduce bias in dementia research? *CMAJ* 2008; **179**(8): 751-3.
29. Jansen JP. Network meta-analysis of individual and aggregate level data. *Res Synth Methods* 2012; **3**(2): 14.
30. Mavridis D, White IR. Dealing with missing outcome data in meta-analysis. *Res Synth Methods* 2020; **11**(1): 2-13.
31. Kibret T, Richer D, Beyene J. Bias in identification of the best treatment in a Bayesian network meta-analysis for binary outcome: a simulation study. *Clinical epidemiology* 2014; **6**: 451-60.

For peer review only

For peer review only - http://bmjopen.bmj.com/site/about/guidelines.xhtml

Figure Captions

Figure 1. Flow diagram for study inclusion in the review (a) and studies retrieved with individual patient data (b).

Figure 2. Network diagrams for (a) MMSE and (b) AE outcomes. The size of each node and line indicates the number of studies included in each treatment comparison. The number of studies per treatment comparison is presented on each edge, and the number of studies with individual patient data (IPD) is depicted in a parenthesis. Orange coloured edges are informed by both IPD and aggregate data, whereas black coloured edges are informed by aggregate data only.

Figure 3. Forest plot of network meta-analysis (NMA) results for all cognitive enhancers versus placebo in (a) MMSE outcome, and (b) AE outcome. NMA results are presented for i) aggregate data (AD) and fully adjusted results from studies with available individual patient data (IPD), ii) AD and crude results from studies with available IPD, iii) AD only (studies with available IPD are not included in the analysis), and iv) crude results from individual studies with individual patient data (IPD).

Figure 4. Rank-heat plot of P-scores for 9 treatments, including placebo, studied in randomized clinical trials with patients with Alzheimer's Dementia assessing MMSE. Circles from inside out present results for different network meta-analyses including: i) aggregate data (AD) only (studies with available IPD are not included in the analysis), ii) crude results from individual studies with individual patient data (IPD), iii) AD and crude results from studies with available IPD, and iv) AD and fully adjusted results from studies with available IPD. Numbers within each sector correspond to the P-score values as calculated in each model.

For peer review only

Tables

Table 1· Study and patient characteristics

	AD (N=80)	IPD (N=12)
**Total # participants**	21,138	5839
**Longest duration of follow-up in weeks: mean (range)**	28·28 (8 - 208)	29·33 (12 - 104)
**Mean number of patients (range)**	264·23 (14 - 2,045)	486·58 (123 - 2,045)
**Mean age in years (range)**	74·64 (61 - 85·7)	73·94 (70·4 - 78)
**Mean % Female (range)**	61·35 (3 - 89)	62·76 (53·68 - 81)
**Country of conduct: frequency (%)**		
Canada	2 (2·50)	1 (8·33)
China	6 (7·50)	-
Germany	1 (1·25)	-
Iran	2 (2·50)	-
Italy	6 (7·50)	-
Japan	7 (8·75)	1 (8·33)
Norway	1 (1·25)	-
Romania	1 (1·25)	-
South Korea	1 (1·25)	-
Spain	3 (3·75)	-
Sweden	2 (2·50)	-
Turkey	1 (1·25)	-
United Kingdom	6 (7·50)	1 (8·33)
United States	15 (18·75)	-
Multi-national	26 (32·50)	9 (75·00)
**Interventions examined: frequency***		
Placebo/no treatment	61 (76·25)	12 (100·00)
Donepezil	47 (58·75)	4 (33·33)
Galantamine	20 (25·00)	4 (33·33)
Memantine	20 (25·00)	3 (25·00)
Rivastigmine**	18 (22·50)	1 (8·33)
**Effectiveness outcomes reported: frequency***		
Mini-Mental State Examination	57 (71·25)	6 (50·00)
Adverse Events	46 (57·50)	12 (100·00)
**Funding**		
Industry-sponsored	48 (60·00)	12 (100·00)
Publicly-sponsored‡	9 (11·25)	-
Mixed	7 (8·75)	-
Not Reported	16 (20·0)	-
**Severity of Alzheimer's dementia: frequency (%)**		
Mild	3 (3·75)	-
Mild-Moderate	44 (55·00)	7 (58·33)
Mild-Severe	2 (2·50)	-
Moderate	3 (3·75)	-
Moderate-Severe	11 (13·75)	1 (8·33)
Severe	6 (7·50)	2 (16·67)

For peer review only

For peer review only - http://bmjopen.bmj.com/site/about/guidelines.xhtml

Not Reported	11 (13·75)	2 (16·67)
**Diagnostic criteria for Alzheimer's dementia: frequency***		
Mini-Mental State Examination	70 (87·50)	12 (100·00)
National Institute of Neurological Disorders and Stroke-Alzheimer Disease and Related Disorders Association	67 (83·75)	12 (100·00)
Diagnostic and Statistical Manual of Mental Disorders	39 (48·75)	5 (41·67)
Magnetic Resonance Imaging/Computerized Tomography	9 (11·25)	2 (16·67)
Clinical Dementia Rating	6 (7·50)	-
Hachinski Ischemic Score	5 (6·25)	-
Alzheimer's Disease Assessment Scale-Cognitive Subscale	3 (3·75)	1 (8·33)
Other	20 (25·00)	1 (8·33)

Abbreviations: -, not applicable
* Multiple interventions and outcomes reported per study;
** Rivastigmine refers to either oral or transdermal administration
ǂ Including sponsors such as the National Institute of Aging, UK Medical Research Council, and Veteran Affairs

For peer review only

For peer review only - http://bmjopen.bmj.com/site/about/guidelines.xhtml

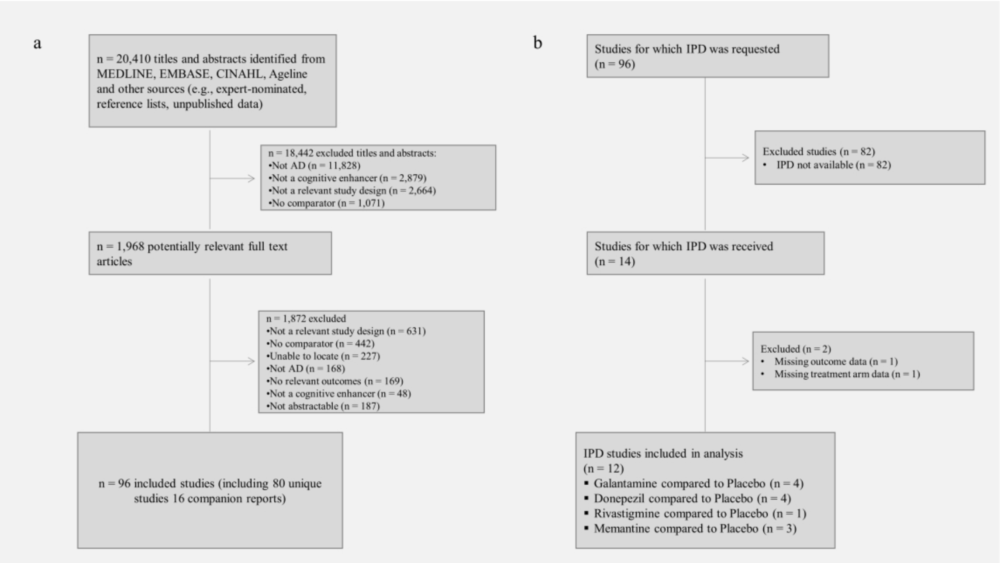

Figure 1. Flow diagram for study inclusion in the review (a) and studies retrieved with individual patient data (b).

338x190mm (300 x 300 DPI)

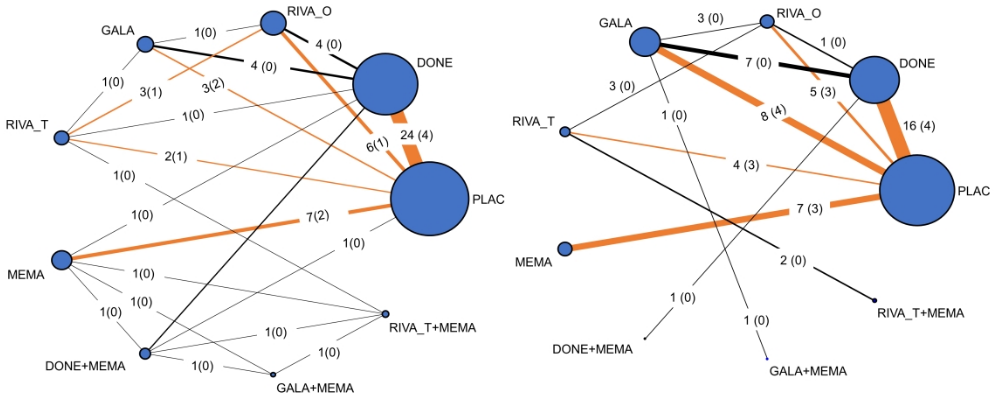

a. MMSE outcome b. AE outcome

Network diagrams for (a) MMSE and (b) AE outcomes. The size of each node and line indicates the number of studies included in each treatment comparison. The number of studies per treatment comparison is presented on each edge, and the number of studies with individual patient data (IPD) is depicted in a parenthesis. Orange coloured edges are informed by both IPD and aggregate data, whereas black coloured edges are informed by aggregate data only.

338x190mm (300 x 300 DPI)

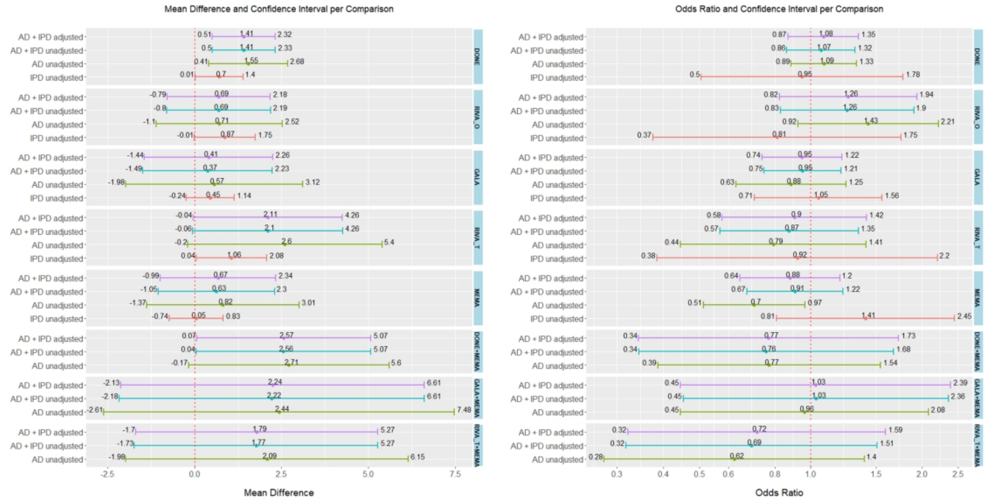

a. MMSE outcome

b. AE outcome

Forest plot of network meta-analysis (NMA) results for all cognitive enhancers versus placebo in (a) MMSE outcome, and (b) AE outcome. NMA results are presented for i) aggregate data (AD) and fully adjusted results from studies with available individual patient data (IPD), ii) AD and crude results from studies with available IPD, iii) AD only (studies with available IPD are not included in the analysis), and iv) crude results from individual studies with individual patient data (IPD).

338x190mm (300 x 300 DPI)

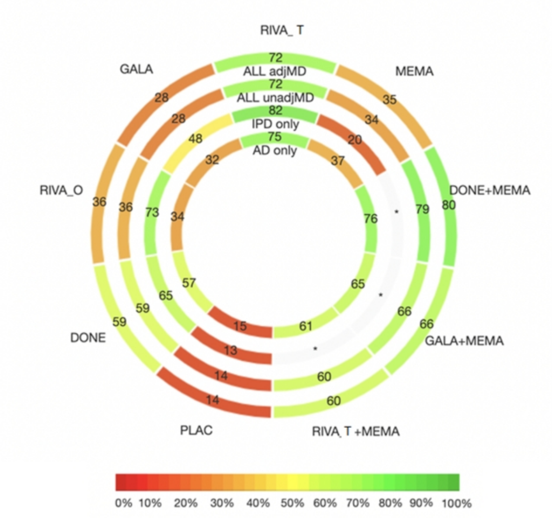

Figure 4. Rank-heat plot of P-scores for 9 treatments, including placebo, studied in randomized clinical trials with patients with Alzheimer's Dementia assessing MMSE. Circles from inside out present results for different network meta-analyses including: i) aggregate data (AD) only (studies with available IPD are not included in the analysis), ii) crude results from individual studies with individual patient data (IPD), iii) AD and crude results from studies with available IPD, and iv) AD and fully adjusted results from studies with available IPD. Numbers within each sector correspond to the P-score values as calculated in each model.

338x190mm (300 x 300 DPI)

Additional File 1: ***Comparative safety and efficacy of cognitive enhancers for Alzheimer's dementia: A systematic review with individual patient data network meta-analysis***

Appendix 1: Additional information on the methods used in the review 2
Appendix 2: Studies included in the systematic review 7
Appendix 3: Studies with available IPD but insufficient data to be included in the analysis 13
Appendix 4: List of studies requested and sponsor response 14
Appendix 5: Study characteristics of the included RCTs 17
Appendix 6. Characteristics of studies with shared IPD 24
Appendix 7: Comparison of studies with shared IPD with (a) all remaining studies and (b) studies for which sponsors claimed unavailable IPD. AD: aggregate data; IPD: individual patient data 27
Appendix 8: Cochrane Risk-of-bias appraisal results (n = 80) 29
Appendix 9: Overall risk of bias for studies with shared IPD against (a) all remaining studies and (b) studies for which sponsors claimed unavailable IPD. AD: aggregate data; IPD: individual patient data 31
Appendix 10: Study-specific effect sizes calculated from shared IPD and published data. IPD: individual patient data 32
Appendix 11: Correlation between participant age and dropout in studies with IPD. IPD: individual patient data 37
Appendix 12: Comparison Adjusted Funnel plot (all treatments vs placebo) 38
Appendix 13: Distribution of potential effect modifiers per treatment comparison and outcome 39
Appendix 14: Consistency Assessment – Loop-specific approach (using adjusted treatment effects) 42
Appendix 15: Network and standard meta-analysis results 43
Appendix 16: Network subgroup and meta-regression analysis results 46
Appendix 17: CINeMA results 53
Appendix 18: Study definitions for adverse events 56
Appendix 19: Time taken to achieve at least an adverse event using individual patient data 59
Appendix 20: Rank-heat plot for adverse events 60
Appendix 21: Challenges encountered during the individual patient data request from sponsors 61

For peer review only - http://bmjopen.bmj.com/site/about/guidelines.xhtml

Appendix 1: Additional information on the methods used in the review

Eligibility criteria, search strategy and study selection

We considered an adverse event (AE) as defined in the individual trials. Definitions were captured for each study separately. We included donepezil, rivastigmine, galantamine, and memantine alone or in combination with other treatment and compared with each other, supportive care or placebo. We excluded studies examining other cognitive enhancers or including individuals with mixed causes of dementia. We included published studies written in any language and of any duration.

Using terms from our previous review,4 the MEDLINE literature search was drafted by an experienced librarian (Dr. Laure Perrier) and revised after another librarian (Ms. Becky Skidmore) peer-reviewed the search terms.10 Subsequently, we searched the following databases: MEDLINE, EMBASE, Cochrane Methodology Register, CINAHL, Ageline and Cochrane Central Register of Controlled Trials. We also scanned reference lists of included studies and relevant reviews to supplement the electronic literature searches.

After pilot-testing, the results from the literature search were screened by pairs of reviewers working independently. Pairs of reviewers independently abstracted data (e.g., study characteristics, patient characteristics, outcome results) after a pilot-test. We resolved conflicts through discussion. The overall agreement among the reviewers for screening was over 70%.

IPD collection process and data abstraction

During the author contact process, two authors (a senior scientist ACT and a research assistant SL) sent a data request following several strategies as outlined in the RCT protocol:[1] a) an email requesting their IPD, b) email reminders (4 in total) at 2, 6, 10, and 14-week intervals after the initial email, c) reminders by post in week 7, and d) reminders via telephone in week 15. We also invited eligible authors to be a co-author on our updated systematic review provided that they share their anonymized IPD, and meet the International Committee of Medical Journal Editors (ICMJE) criteria for authorship.[2] Our team (AAV, SL) also contacted sponsors of the eligible trials, as reported in the publications. If a sponsor was not reported in a publication, we contacted the author (whom we emailed during the RCT) to determine who sponsored the study. To contact industry sponsors, we navigated the data sharing process from their websites or via an email, online portal, or phone inquiry. When no response was received, two follow-up reminders were sent to the sponsors.

We requested IPD on 1) patients: age, sex, severity of Alzheimer's disease (e.g. baseline MMSE [Mini-Mental State Examination] level), presence of behavioral disturbance, comorbid conditions (e.g., stroke, cardiovascular conditions, Parkinson's disease), other medications used for each patient, number of drop-outs, reasons for drop-out, and number of participants, 2) medication: treatment each patient was allocated to, dosage, 3) outcomes: event, date of event, time taken to achieve the event for AEs, MMSE values and measurement dates, and 4) date and method of randomization. We checked IPD provided for consistency with results from published RCTs., and contacted IPD providers when data inconsistencies were found.

Data extraction items included a) study characteristics: year of publication, country and continent according to the first author, journal in which the study was published, funding information; b) aggregate patient characteristics: study size and percentage of males, c) outcome data: study data (e.g., events or mean and standard deviations, and sample size per arm), and d) treatments compared. We also abstracted the corresponding authors' contact details. We categorized each study according to funding source (industry-sponsored, publicly-sponsored, mixed, and non-sponsored).

Certainty of the evidence

We used CINeMA (Confidence in Network Meta-Analysis) to assess confidence in the NMA estimates.[3] Six domains were evaluated with scores 'no concerns', 'some concerns' and 'major concerns': 1) within-study bias, 2) reporting bias, 3) indirectness, 4) imprecision, 5) heterogeneity, and 6) incoherence. We used the overall risk of bias per study, and for each treatment comparison we applied the average risk of bias. Similarly, for all treatment comparisons we used the average for indirectness. We assessed reporting bias based on the comparison-adjusted funnel plot since there are no established statistical methods to explore reporting bias. We used a comparison-adjusted funnel to account for the fact that each set of studies estimates a different summary effect in NMA. This is a scatterplot of the difference between the study-specific effect sizes from the

For peer review only - http://bmjopen.bmj.com/site/about/guidelines.xhtml

corresponding comparison-specific effect (obtained from standard meta-analysis) against the corresponding study-specific standard error. We used the fixed effect model for the standard meta-analysis performed for each treatment comparison, ordered treatments chronologically according to year of availability in Canada, and used only treatment comparisons versus placebo. We used the *netfunnel* command in Stata to produce the comparison-adjusted funnel plot.[4]

For imprecision, we considered a MD=1.4 and a OR=1 as a clinically important size of effect for MMSE and AE, respectively, and followed the CINeMA guidelines for exploring whether statistical significance and clinical importance coincide. Similarly, heterogeneity and incoherence (i.e. inconsistency) were assessed by following the standard CINeMA approach.

CINeMA assesses the credibility of the NMA results and heterogeneity examining the range of both confidence intervals (CIs; which do not capture heterogeneity) and prediction intervals (PIs; which capture heterogeneity) in relation to their equivalence. If a PI includes values that lead to a different conclusion than an assessment based on the corresponding CI, then this suggests that there is considerable heterogeneity. PIs are expected to include the true intervention effects in future studies with characteristics similar to the existing studies, and they incorporate the extent of between-study heterogeneity.[5 6] In the presence of considerable heterogeneity, they are wide to include intervention effects with different implications for practice. However, caution is needed in the interpretation of results in the presence of funnel plot asymmetry, since PIs are based on the assumption of a normal distribution for the study-specific effects and as such they may be problematic if the data do not follow a normal distribution.

Statistical Analysis

We performed a descriptive analysis using frequencies and percentages of the discrete characteristics of the included patients and treatments of the eligible studies. We explored the distributions of the continuous patient characteristics per outcome and treatment group using means and standard deviations. For studies not providing outcome results for a certain outcome, we presented distributions of the available and requested patient characteristics, whenever available. Outliers for each patient characteristic were also explored in each study dataset using boxplots. We also recorded the number of missing participants per treatment group and overall. We compared the characteristics of the unavailable and the available by the sponsors' studies. In particular, we explored whether these were well-conducted according to overall risk of bias, and compared distributions of mean participant age, publication year, study duration, study size, percent male, and magnitude of treatment effect, to assess for potential bias in IPD sharing. We conducted a two-stage analysis for both standard meta-analysis and NMA. The network geometry was explored through the presentation of network plots.

First stage

All IPD from included studies were first aggregated to study-level summary statistics using each sponsor's portal. The use of different platforms and failure to obtain IPD from all studies restricted us from combining IPD in a one-stage analysis. For each separate study with IPD available, we fitted a logistic regression model for the binary outcome and a linear regression model for the continuous outcome. For MMSE, we considered the longest duration of follow-up per study (most frequently at week 24). In the shared IPD, when we were unable to make a judgement on first and last date of visit per patient, we used the older coded date and the newest coded date as baseline and final value for each patient respectively.

Initially, we did not adjust for any of the patient characteristics provided, but in a subsequent analysis we included patient-level covariates with as many interaction terms in the model as the patient characteristics were provided (considering only the ones we have asked for). For each study, we obtained the adjusted odds ratio (OR) for binary data and adjusted mean difference (MD) for continuous data, along their corresponding 95% CI. We adjusted for any of the following variables that were available in each study: age, sex, severity of Alzheimer's disease (e.g., baseline Mini-Mental State Examination [MMSE] level), presence of behavioural disturbance, comorbidity, and other medications. The first stage of the IPD analyses were conducted in RStudio,[7] which was available in data providers. Additional medications and comorbid conditions were grouped into broader categories according to their clinical relevance to increase power in our analysis (e.g., grouped medications as anti-psychotics, anti-depressants, and cognitive enhancers, as well as comorbid conditions as psychiatric, neurological, and cardiac disorders). Eligible studies with insufficient data to derive a pairwise estimate for NMA were summarized descriptively without performing a statistical analysis.

For peer review only - http://bmjopen.bmj.com/site/about/guidelines.xhtml

We applied an available case analysis for each study, since we were unable to install R packages in most sponsor-specific platforms, and hence we applied a consistent approach across all IPD datasets. We explored the impact of missing data during the second stage of analysis. Reasons for missing participants and time taken to have a adverse event were captured (when available).

We synthesized IPD at the first stage in four different proprietary sponsor-specific platforms. Analyses were conducted in the RStudio using different R versions[7] according to what was provided in each sponsor's platform: R version 3.4.1 for AbbVie, R version 3.4.3 for CSDR, R version 3.5.1 for YODA, R version 3.6.0 for Lundbeck.

Second stage

Since we were not successful in obtaining IPD for all eligible studies, we combined both IPD and aggregate data in a single meta-analysis or NMA model. Both IPD and aggregate data studies shared the same amount of heterogeneity. In both meta-analysis and NMA models, we combined the adjusted IPD estimates with the aggregate data (main analysis). As a secondary analysis, we combined the unadjusted estimates from retrieved IPD with the evidence provided by the aggregated data studies in a joint NMA model. A common-within network between-study variance was assumed across comparisons for all NMA models.[8] We estimated the between-study variance using the DerSimonian and Laird[9] method and compared it with the relevant distributions provided by Turner et al[10] and Rhodes et al[11] to assess heterogeneity. We also calculated I^2 on the NMA level to quantify overall heterogeneity and inconsistency in each outcome.

To assess the validity of the transitivity assumption for each outcome, we assessed the distribution of potential effect modifiers (e.g., age, sex) across treatment comparisons in each network.[12-14] We visually inspected similarity and assessed whether these characteristics were likely to modify the treatment effect. We evaluated the consistency assumption using the design-by-treatment interaction model[15 16] and the loop-specific method.[17 18] In the presence of statistically significant inconsistency, we checked the data for discrepancies and if none were identified, we planned to conduct subgroup NMA or network meta-regression analysis adjusting for potential variables influencing the results.

We conducted additional NMA analyses for all potential effect modifiers requested from data providers. If relevant data were not available in the IPD, we used aggregate data of the relevant publications. Additional NMA analyses included: 1) subgroup analysis for industry vs. publicly sponsored studies, for studies with available IPD vs. studies with aggregate data (unadjusted estimates), and for AD severity, classified according to MMSE scores using the National Institute for Health and Care Excellence categories: mild (21–24), moderate (10–20), severe (<10),[19] 2) network meta-regression accounting for study duration, year of publication, mean age, and sex (% of male participants) effect modifiers separately and assuming a common regression coefficient across comparisons (studies with aggregate data were used only; studies with available IPD were pooled in a NMA separately adjusted for available covariates at first stage), 3) sensitivity analysis including studies with low risk of bias for allocation concealment and incomplete outcome data items, as these items may have an important impact on the meta-analysis results according to our previous NMA,[20] and 4) the 'informative missingness difference of means' (IMDoM) imputation method[21] for MMSE for the aggregate data studies to assess the impact of missing data in our NMA. In all additional NMA analyses, we used the adjusted effect estimates derived from the IPD within-study analysis and the aggregate data extracted from the eligible publications. Network meta-regression was performed in a Bayesian setting using OpenBUGS version 3.2.3, non-informative priors for all parameters in the model and a half-normal prior for the between standard deviation. We compared the results of the additional models by evaluating the treatment effect estimates and ranking statistics, as well as monitoring the reduction in the between-study variance.

We present the results using summary effect sizes, and in particular the MD for MMSE and the OR for AE, along with their corresponding CIs and PIs.[6] We ranked the interventions for each outcome according to their efficacy and safety using P-scores in frequentist analyses and SUCRAs (surface under the cumulative ranking curve) in Bayesian analyses (e.g., meta-regression analysis).[22 23] SUCRA is the numeric presentation of the intervention ranking and is based on the surface under the cumulative ranking probability function for each treatment. An equivalent frequentist statistic is the P-score measure that is based on the observed treatment effect estimates and their uncertainty. Both measures summarize the estimated probabilities for all possible ranks, account for uncertainty in relative ranking, and range between 0-100%, with 100% reflecting the best intervention with no uncertainty and 0% reflecting the worst intervention with no uncertainty. Ranking strategies are commonly encountered in NMAs,[24-26] and we present the hierarchy of cognitive enhancers in a rank-heat plot.[27]

For peer review only - http://bmjopen.bmj.com/site/about/guidelines.xhtml

Meta-analysis and NMA at the 2nd stage were conducted in the RStudio using R version 3.6.2 and the *meta*[28] and *netmeta*[29] packages, respectively.

Appendix 1 References:

1. Veroniki AA, Straus SE, Ashoor H, et al. Contacting authors to retrieve individual patient data: study protocol for a randomized controlled trial. *Trials* 2016;17(1):138. doi: 10.1186/s13063-016-1238-z [published Online First: 2016/03/16]
2. Hager K, Baseman AS, Nye JS, et al. Effects of galantamine in a 2-year, randomized, placebo-controlled study in Alzheimer's disease. *Neuropsychiatric disease and treatment* 2014;10:391-401. doi: 10.2147/ndt.s57909 [published Online First: 2014/03/05]
3. CINeMA: Confidence in Network Meta-Analysis. Institute of Social and Preventive Medicine, University of Bern. *Available from cinemaispmch* 2017
4. Chaimani A, Higgins JP, Mavridis D, et al. Graphical tools for network meta-analysis in STATA. *PLoS One* 2013;8(10):e76654. doi: 10.1371/journal.pone.0076654 [published Online First: 2013/10/08]
5. Higgins JP, Thompson SG, Spiegelhalter DJ. A re-evaluation of random-effects meta-analysis. *J R Stat Soc Ser A Stat Soc* 2009;172(1):137-59. doi: 10.1111/j.1467-985X.2008.00552.x [published Online First: 2009/04/22]
6. Riley RD, Higgins JP, Deeks JJ. Interpretation of random effects meta-analyses. *Bmj* 2011;342:d549. doi: 10.1136/bmj.d549 [published Online First: 2011/02/12]
7. R Core Team. R: A Language and Environment for Statistical Computing. Vienna, Austria: R Foundation for Statistical Computing, 2019.
8. Lu G, Ades AE. Combination of direct and indirect evidence in mixed treatment comparisons. *Statistics in medicine* 2004;23(20):3105-24. doi: 10.1002/sim.1875 [published Online First: 2004/09/28]
9. DerSimonian R, Laird N. Meta-analysis in clinical trials. *Controlled clinical trials* 1986;7(3):177-88. doi: 10.1016/0197-2456(86)90046-2
10. Turner RM, Davey J, Clarke MJ, et al. Predicting the extent of heterogeneity in meta-analysis, using empirical data from the Cochrane Database of Systematic Reviews. *International journal of epidemiology* 2012;41(3):818-27. doi: 10.1093/ije/dys041 [published Online First: 2012/03/31]
11. Rhodes KM, Turner RM, Higgins JP. Predictive distributions were developed for the extent of heterogeneity in meta-analyses of continuous outcome data. *Journal of clinical epidemiology* 2015;68(1):52-60. doi: 10.1016/j.jclinepi.2014.08.012 [published Online First: 2014/10/12]
12. Salanti G. Indirect and mixed-treatment comparison, network, or multiple-treatments meta-analysis: many names, many benefits, many concerns for the next generation evidence synthesis tool. *Res Synth Methods* 2012;3(2):80-97. doi: 10.1002/jrsm.1037 [published Online First: 2012/06/11]
13. Jansen JP, Naci H. Is network meta-analysis as valid as standard pairwise meta-analysis? It all depends on the distribution of effect modifiers. *BMC Med* 2013;11:159. doi: 10.1186/1741-7015-11-159 [published Online First: 2013/07/04]
14. Cipriani A, Higgins JP, Geddes JR, et al. Conceptual and technical challenges in network meta-analysis. *Annals of internal medicine* 2013;159(2):130-7. doi: 10.7326/0003-4819-159-2-201307160-00008
15. Higgins JP, Jackson D, Barrett JK, et al. Consistency and inconsistency in network meta-analysis: concepts and models for multi-arm studies. *Res Synth Methods* 2012;3(2):98-110. doi: 10.1002/jrsm.1044 [published Online First: 2012/06/01]
16. White IR BJ, Jackson D, Higgins JPT. Consistency and inconsistency in network meta-analysis: model estimation using multivariate meta-regression. *Res Synth Methods* 2012;3(2):15.
17. Song F, Altman DG, Glenny AM, et al. Validity of indirect comparison for estimating efficacy of competing interventions: empirical evidence from published meta-analyses. *Bmj* 2003;326(7387):472. doi: 10.1136/bmj.326.7387.472 [published Online First: 2003/03/01]
18. Veroniki AA, Vasiliadis HS, Higgins JP, et al. Evaluation of inconsistency in networks of interventions. *International journal of epidemiology* 2013;42(1):332-45. doi: 10.1093/ije/dys222
19. National Institute for Health and Clinical Excellence. Donepezil, galantamine, rivastigmine and memantine for the treatment of Alzheimer's disease. Review of NICE technology appraisal guidance 111 NICE technology appraisal guidance 217. London, UK, 2011.
20. Veroniki AA, Straus SE, Ashoor HM, et al. Comparative safety and effectiveness of cognitive enhancers for Alzheimer's dementia: protocol for a systematic review and individual patient data network meta-analysis. *BMJ open* 2016;6(1):e010251. doi: 10.1136/bmjopen-2015-010251

For peer review only - http://bmjopen.bmj.com/site/about/guidelines.xhtml

21. Mavridis D, White IR, Higgins JP, et al. Allowing for uncertainty due to missing continuous outcome data in pairwise and network meta-analysis. *Statistics in medicine* 2015;34(5):721-41. doi: 10.1002/sim.6365 [published Online First: 2014/11/14]
22. Rucker G, Schwarzer G. Ranking treatments in frequentist network meta-analysis works without resampling methods. *BMC Med Res Methodol* 2015;15:58. doi: 10.1186/s12874-015-0060-8 [published Online First: 2015/08/01]
23. Salanti G, Ades AE, Ioannidis JP. Graphical methods and numerical summaries for presenting results from multiple-treatment meta-analysis: an overview and tutorial. *J Clin Epidemiol* 2011;64(2):163-71. doi: 10.1016/j.jclinepi.2010.03.016 [published Online First: 2010/08/07]
24. Mbuagbaw L, Rochwerg B, Jaeschke R, et al. Approaches to interpreting and choosing the best treatments in network meta-analyses. *Syst Rev* 2017;6(1):79. doi: 10.1186/s13643-017-0473-z [published Online First: 2017/04/14]
25. Petropoulou M, Nikolakopoulou A, Veroniki AA, et al. Bibliographic study showed improving statistical methodology of network meta-analyses published between 1999 and 2015. *J Clin Epidemiol* 2017;82:20-28. doi: 10.1016/j.jclinepi.2016.11.002 [published Online First: 2016/11/20]
26. Veroniki AA, Straus SE, Rucker G, et al. Is providing uncertainty intervals in treatment ranking helpful in a network meta-analysis? *J Clin Epidemiol* 2018;100:122-29. doi: 10.1016/j.jclinepi.2018.02.009 [published Online First: 2018/02/13]
27. Veroniki AA, Straus SE, Fyraridis A, et al. The rank-heat plot is a novel way to present the results from a network meta-analysis including multiple outcomes. *Journal of clinical epidemiology* 2016;76:193-9. doi: 10.1016/j.jclinepi.2016.02.016 [published Online First: 2016/03/05]
28. Balduzzi S, Rücker G, Schwarzer G. How to perform a meta-analysis with R: a practical tutorial. *Evidence-based mental health* 2019;22(4):153-60. doi: 10.1136/ebmental-2019-300117 [published Online First: 2019/09/30]
29. Rücker G, Schwarzer G, Krahn U, et al. netmeta: Network Meta-Analysis using Frequentist Methods. R package version 0.9-8. 2018 [Available from: https://CRAN.R-project.org/package=netmeta.

For peer review only

For peer review only - http://bmjopen.bmj.com/site/about/guidelines.xhtml

Appendix 2: Studies included in the systematic review

80 Main Studies:

30. Agid Y, Dubois B, Anand R, Gharabawi G, International Rivastigmine Investigators. Efficacy and tolerability of rivastigmine in patients with dementia of the Alzheimer type. *Current Therapeutic Research* 1998; **59**(12): 837-45.

31. Ancoli-Israel S, Amatniek J, Ascher S, Sadik K, Ramaswamy K. Effects of galantamine versus donepezil on sleep in patients with mild to moderate Alzheimer disease and their caregivers: a double-blind, head-to-head, randomized pilot study. *Alzheimer disease and associated disorders* 2005; **19**(4): 240-5.

32. Andersen F, Viitanen M, Halvorsen DS, Straume B, Wilsgaard T, Engstad TA. The effect of stimulation therapy and donepezil on cognitive function in Alzheimer's disease. A community based RCT with a two-by-two factorial design. *BMC neurology* 2012; **12**: 59.

33. Araki T, Wake R, Miyaoka T, et al. The effects of combine treatment of memantine and donepezil on Alzheimer's disease patients and its relationship with cerebral blood flow in the prefrontal area. *International journal of geriatric psychiatry* 2014; **29**(9): 881-9.

34. Bakchine S, Loft H. Memantine treatment in patients with mild to moderate Alzheimer's disease: results of a randomised, double-blind, placebo-controlled 6-month study. *Journal of Alzheimer's disease : JAD* 2008; **13**(1): 97-107.

35. Black SE, Doody R, Li H, et al. Donepezil preserves cognition and global function in patients with severe Alzheimer disease. *Neurology* 2007; **69**(5): 459-69.

36. Blesa González R, Boada Rovira M, Martínez Parra C, Gil-Saladié D, Almagro CA, Gobartt Vázquez AL. Evaluation of the convenience of changing the rivastigmine administration route in patients with Alzheimer disease. *Neurologia (Barcelona, Spain)* 2011; **26**(5): 262-71.

37. Burns A, Bernabei R, Bullock R, et al. Safety and efficacy of galantamine (Reminyl) in severe Alzheimer's disease (the SERAD study): a randomised, placebo-controlled, double-blind trial. *The Lancet Neurology* 2009; **8**(1): 39-47.

38. Burns A, Perry E, Holmes C, et al. A double-blind placebo-controlled randomized trial of Melissa officinalis oil and donepezil for the treatment of agitation in Alzheimer's disease. *Dementia and geriatric cognitive disorders* 2011; **31**(2): 158-64.

39. Burns A, Rossor M, Hecker J, et al. The effects of donepezil in Alzheimer's disease - results from a multinational trial. *Dementia and geriatric cognitive disorders* 1999; **10**(3): 237-44.

40. Choi SH, Park KW, Na DL, et al. Tolerability and efficacy of memantine add-on therapy to rivastigmine transdermal patches in mild to moderate Alzheimer's disease: a multicenter, randomized, open-label, parallel-group study. *Current medical research and opinion* 2011; **27**(7): 1375-83.

41. Corey-Bloom J. A randomized trial evaluating the efficacy and safety of ENA 713 (rivastigmine tartrate), a new acetylcholinesterase inhibitor, in patients with mild to moderately severe Alzheimer's disease. *Int J Geriatr Psyopharmacol* 1998; **1**: 55-65.

42. Creţu O, Szalontay AS, Chiriţă R, Chiriţă V. Effect of memantine treatment on patients with moderate-to-severe Alzheimer's disease treated with donepezil. *Revista medico-chirurgicala a Societatii de Medici si Naturalisti din Iasi* 2008; **112**(3): 641-5.

43. Dysken MW, Sano M, Asthana S, et al. Effect of vitamin E and memantine on functional decline in Alzheimer disease: the TEAM-AD VA cooperative randomized trial. *Jama* 2014; **311**(1): 33-44.

44. Farlow MR, Grossberg GT, Sadowsky CH, Meng X, Somogyi M. A 24-week, randomized, controlled trial of rivastigmine patch 13.3 mg/24 h versus 4.6 mg/24 h in severe Alzheimer's dementia. *CNS neuroscience & therapeutics* 2013; **19**(10): 745-52.

45. Feldman H, Gauthier S, Hecker J, Vellas B, Subbiah P, Whalen E. A 24-week, randomized, double-blind study of donepezil in moderate to severe Alzheimer's disease. *Neurology* 2001; **57**(4): 613-20.

46. Feldman HH, Lane R. Rivastigmine: a placebo controlled trial of twice daily and three times daily regimens in patients with Alzheimer's disease. *Journal of neurology, neurosurgery, and psychiatry* 2007; **78**(10): 1056-63.

For peer review only - http://bmjopen.bmj.com/site/about/guidelines.xhtml

47. Fox C, Crugel M, Maidment I, et al. Efficacy of memantine for agitation in Alzheimer's dementia: a randomised double-blind placebo controlled trial. *PloS one* 2012; **7**(5): e35185.

48. Frölich L, Ashwood T, Nilsson J, Eckerwall G. Effects of AZD3480 on cognition in patients with mild-to-moderate Alzheimer's disease: a phase IIb dose-finding study. *Journal of Alzheimer's disease : JAD* 2011; **24**(2): 363-74.

49. Fuschillo C, Ascoli E, Franzese G, et al. Alzheimer's disease and acetylcholinesterase inhibitor agents: a two-year longitudinal study. *Archives of gerontology and geriatrics Supplement* 2004; (9): 187-94.

50. Gault LM, Ritchie CW, Robieson WZ, Pritchett Y, Othman AA, Lenz RA. A phase 2 randomized, controlled trial of the α7 agonist ABT-126 in mild-to-moderate Alzheimer's dementia. *Alzheimer's & dementia (New York, N Y)* 2015; **1**(1): 81-90.

51. Gold M, Alderton C, Zvartau-Hind M, et al. Rosiglitazone monotherapy in mild-to-moderate Alzheimer's disease: results from a randomized, double-blind, placebo-controlled phase III study. *Dementia and geriatric cognitive disorders* 2010; **30**(2): 131-46.

52. Greenberg SM, Tennis MK, Brown LB, et al. Donepezil therapy in clinical practice: a randomized crossover study. *Archives of neurology* 2000; **57**(1): 94-9.

53. Grossberg GT, Manes F, Allegri RF, et al. The safety, tolerability, and efficacy of once-daily memantine (28 mg): a multinational, randomized, double-blind, placebo-controlled trial in patients with moderate-to-severe Alzheimer's disease taking cholinesterase inhibitors. *CNS drugs* 2013; **27**(6): 469-78.

54. Hager K, Baseman AS, Nye JS, et al. Effects of galantamine in a 2-year, randomized, placebo-controlled study in Alzheimer's disease. *Neuropsychiatric disease and treatment* 2014; **10**: 391-401.

55. Haig GM, Pritchett Y, Meier A, et al. A randomized study of H3 antagonist ABT-288 in mild-to-moderate Alzheimer's dementia. *Journal of Alzheimer's disease : JAD* 2014; **42**(3): 959-71.

56. Hernandez C, Unturbe F, Martinez-Lage P, Lucas A, Gregorio P, Alonso T. Effects of combined pharmacologic and cognitive treatment in the progression of moderate dementia: a two-year follow-up. *REVISTA ESPANOLA DE GERIATRIA Y GERONTOLOGIA* 2007; **42**(1): 3.

57. Herrmann N, Gauthier S, Boneva N, Lemming OM. A randomized, double-blind, placebo-controlled trial of memantine in a behaviorally enriched sample of patients with moderate-to-severe Alzheimer's disease. *International psychogeriatrics* 2013; **25**(6): 919-27.

58. Holmes C, Wilkinson D, Dean C, et al. The efficacy of donepezil in the treatment of neuropsychiatric symptoms in Alzheimer disease. *Neurology* 2004; **63**(2): 214-9.

59. Homma A, Imah Y, Hariguchi S. Late phase II clinical study of acetylcholinesterase inhibitor E 2020 in patients with alzheimer-type dementia-12-weeks double-blind, placebo-controlled study 3 mg/day, 5mg/day. *Clinical Evaluation* 1998; **26**: 251-84.

60. Homma A, Imai Y, Tago H, et al. Donepezil treatment of patients with severe Alzheimer's disease in a Japanese population: results from a 24-week, double-blind, placebo-controlled, randomized trial. *Dementia and geriatric cognitive disorders* 2008; **25**(5): 399-407.

61. Hong Z, Zhang Z, Wang L, et al. A randomized study comparing the effect and safety of galantamine and donepezil in patients with mild to moderate Alzheimer's disease. *Chin J Neurol* 2006; **39**(6): 379-82.

62. Howard R, McShane R, Lindesay J, et al. Donepezil and memantine for moderate-to-severe Alzheimer's disease. *New England Journal of Medicine* 2012; **366**(10): 893-903.

63. Howard RJ, Juszczak E, Ballard CG, et al. Donepezil for the treatment of agitation in Alzheimer's disease. *The New England journal of medicine* 2007; **357**(14): 1382-92.

64. Hu HT, Zhang ZX, Yao JL, et al. [Clinical efficacy and safety of akatinol memantine in treatment of mild to moderate Alzheimer disease: a donepezil-controlled, randomized trial]. *Zhonghua nei ke za zhi* 2006; **45**(4): 277-80.

65. Johannsen P, Salmon E, Hampel H, et al. Assessing therapeutic efficacy in a progressive disease: a study of donepezil in Alzheimer's disease. *CNS drugs* 2006; **20**(4): 311-25.

66. Jones RW, Soininen H, Hager K, et al. A multinational, randomised, 12-week study comparing the effects of donepezil and galantamine in patients with mild to moderate Alzheimer's disease. *International journal of geriatric psychiatry* 2004; **19**(1): 58-67.

For peer review only - http://bmjopen.bmj.com/site/about/guidelines.xhtml

67. Kadir A, Darreh-Shori T, Almkvist O, et al. PET imaging of the in vivo brain acetylcholinesterase activity and nicotine binding in galantamine-treated patients with AD. *Neurobiology of aging* 2008; **29**(8): 1204-17.

68. Kano O, Ito H, Takazawa T, et al. Clinically meaningful treatment responses after switching to galantamine and with addition of memantine in patients with Alzheimer's disease receiving donepezil. *Neuropsychiatric disease and treatment* 2013; **9**: 259-65.

69. Karaman Y, Erdoğan F, Köseoğlu E, Turan T, Ersoy AO. A 12-month study of the efficacy of rivastigmine in patients with advanced moderate Alzheimer's disease. *Dementia and geriatric cognitive disorders* 2005; **19**(1): 51-6.

70. Likitjaroen Y, Meindl T, Friese U, et al. Longitudinal changes of fractional anisotropy in Alzheimer's disease patients treated with galantamine: a 12-month randomized, placebo-controlled, double-blinded study. *European archives of psychiatry and clinical neuroscience* 2012; **262**(4): 341-50.

71. Lorenzi M, Beltramello A, Mercuri NB, et al. Effect of memantine on resting state default mode network activity in Alzheimer's disease. *Drugs & aging* 2011; **28**(3): 205-17.

72. Maher-Edwards G, Dixon R, Hunter J, et al. SB-742457 and donepezil in Alzheimer disease: a randomized, placebo-controlled study. *International journal of geriatric psychiatry* 2011; **26**(5): 536-44.

73. Marek GJ, Katz DA, Meier A, et al. Efficacy and safety evaluation of HSD-1 inhibitor ABT-384 in Alzheimer's disease. *Alzheimer's & dementia : the journal of the Alzheimer's Association* 2014; **10**(5 Suppl): S364-73.

74. Mazza M, Capuano A, Bria P, Mazza S. Ginkgo biloba and donepezil: a comparison in the treatment of Alzheimer's dementia in a randomized placebo-controlled double-blind study. *European journal of neurology* 2006; **13**(9): 981-5.

75. Mohs RC, Doody RS, Morris JC, et al. A 1-year, placebo-controlled preservation of function survival study of donepezil in AD patients. *Neurology* 2001; **57**(3): 481-8.

76. Moretti DV. Alpha rhythm oscillations and MMSE scores are differently modified by transdermal or oral rivastigmine in patients with Alzheimer's disease. *American journal of neurodegenerative disease* 2014; **3**(2): 72-83.

77. Mowla A, Mosavinasab M, Haghshenas H, Borhani Haghighi A. Does serotonin augmentation have any effect on cognition and activities of daily living in Alzheimer's dementia? A double-blind, placebo-controlled clinical trial. *Journal of clinical psychopharmacology* 2007; **27**(5): 484-7.

78. Nakamura Y, Imai Y, Shigeta M, et al. A 24-week, randomized, double-blind, placebo-controlled study to evaluate the efficacy, safety and tolerability of the rivastigmine patch in Japanese patients with Alzheimer's disease. *Dementia and geriatric cognitive disorders extra* 2011; **1**(1): 163-79.

79. Nakano S, Asada T, Matsuda H, Uno M, Takasaki M. Donepezil hydrochloride preserves regional cerebral blood flow in patients with Alzheimer's disease. *Journal of nuclear medicine : official publication, Society of Nuclear Medicine* 2001; **42**(10): 1441-5.

80. Nordberg A, Darreh-Shori T, Peskind E, et al. Different cholinesterase inhibitor effects on CSF cholinesterases in Alzheimer patients. *Current Alzheimer research* 2009; **6**(1): 4-14.

81. Pakdaman H, Harandi AA, Hatamian H, et al. Effectiveness and Safety of MLC601 in the Treatment of Mild to Moderate Alzheimer's Disease: A Multicenter, Randomized Controlled Trial. *Dementia and geriatric cognitive disorders extra* 2015; **5**(1): 96-106.

82. Peng D, Xianhao X, Wang L. Efficiency and safety assessment of donepezil for treating mild and moderate Alzheimer disease. *Chinese Journal of Tissue Engineering Research* 2005; **9**(13): 170-2.

83. Peskind ER, Potkin SG, Pomara N, et al. Memantine treatment in mild to moderate Alzheimer disease: a 24-week randomized, controlled trial. *The American journal of geriatric psychiatry : official journal of the American Association for Geriatric Psychiatry* 2006; **14**(8): 704-15.

84. Peters O, Fuentes M, Joachim LK, et al. Combined treatment with memantine and galantamine-CR compared with galantamine-CR only in antidementia drug naïve patients with mild-to-moderate Alzheimer's disease. *Alzheimer's & dementia (New York, N Y)* 2015; **1**(3): 198-204.

85. Reisberg B, Doody R, Stöffler A, Schmitt F, Ferris S, Möbius HJ. Memantine in moderate-to-severe Alzheimer's disease. *The New England journal of medicine* 2003; **348**(14): 1333-41.

For peer review only - http://bmjopen.bmj.com/site/about/guidelines.xhtml

86. Rockwood K, Fay S, Song X, MacKnight C, Gorman M. Attainment of treatment goals by people with Alzheimer's disease receiving galantamine: a randomized controlled trial. *CMAJ : Canadian Medical Association journal = journal de l'Association medicale canadienne* 2006; **174**(8): 1099-105.
87. Rockwood K, Mintzer J, Truyen L, Wessel T, Wilkinson D. Effects of a flexible galantamine dose in Alzheimer's disease: a randomised, controlled trial. *Journal of neurology, neurosurgery, and psychiatry* 2001; **71**(5): 589-95.
88. Rogers SL, Doody RS, Mohs RC, Friedhoff LT. Donepezil improves cognition and global function in Alzheimer disease: a 15-week, double-blind, placebo-controlled study. Donepezil Study Group. *Archives of internal medicine* 1998; **158**(9): 1021-31.
89. Rogers SL, Farlow MR, Doody RS, Mohs R, Friedhoff LT. A 24-week, double-blind, placebo-controlled trial of donepezil in patients with Alzheimer's disease. Donepezil Study Group. *Neurology* 1998; **50**(1): 136-45.
90. Rogers SL, Friedhoff LT. The efficacy and safety of donepezil in patients with Alzheimer's disease: results of a US Multicentre, Randomized, Double-Blind, Placebo-Controlled Trial. The Donepezil Study Group. *Dementia (Basel, Switzerland)* 1996; **7**(6): 293-303.
91. Saxton J, Hofbauer RK, Woodward M, et al. Memantine and functional communication in Alzheimer's disease: results of a 12-week, international, randomized clinical trial. *Journal of Alzheimer's disease : JAD* 2012; **28**(1): 109-18.
92. Scarpini E, Bruno G, Zappalà G, et al. Cessation versus continuation of galantamine treatment after 12 months of therapy in patients with Alzheimer's disease: a randomized, double blind, placebo controlled withdrawal trial. *Journal of Alzheimer's disease : JAD* 2011; **26**(2): 211-20.
93. Schmidt R, Ropele S, Pendl B, et al. Longitudinal multimodal imaging in mild to moderate Alzheimer disease: a pilot study with memantine. *Journal of neurology, neurosurgery, and psychiatry* 2008; **79**(12): 1312-7.
94. Seltzer B, Zolnouni P, Nunez M, et al. Efficacy of donepezil in early-stage Alzheimer disease: a randomized placebo-controlled trial. *Archives of neurology* 2004; **61**(12): 1852-6.
95. Shao ZQ. Comparison of the efficacy of four cholinesterase inhibitors in combination with memantine for the treatment of Alzheimer's disease. *International journal of clinical and experimental medicine* 2015; **8**(2): 2944-8.
96. Shimizu S, Kanetaka H, Hirose D, Sakurai H, Hanyu H. Differential effects of acetylcholinesterase inhibitors on clinical responses and cerebral blood flow changes in patients with Alzheimer's disease: a 12-month, randomized, and open-label trial. *Dementia and geriatric cognitive disorders extra* 2015; **5**(1): 135-46.
97. Solé-Padullés C, Bartrés-Faz D, Lladó A, et al. Donepezil treatment stabilizes functional connectivity during resting state and brain activity during memory encoding in Alzheimer's disease. *Journal of clinical psychopharmacology* 2013; **33**(2): 199-205.
98. Tariot PN, Cummings JL, Katz IR, et al. A randomized, double-blind, placebo-controlled study of the efficacy and safety of donepezil in patients with Alzheimer's disease in the nursing home setting. *Journal of the American Geriatrics Society* 2001; **49**(12): 1590-9.
99. Tariot PN, Solomon PR, Morris JC, Kershaw P, Lilienfeld S, Ding C. A 5-month, randomized, placebo-controlled trial of galantamine in AD. The Galantamine USA-10 Study Group. *Neurology* 2000; **54**(12): 2269-76.
100. Thomas A, Iacono D, Bonanni L, D'Andreamatteo G, Onofrj M. Donepezil, rivastigmine, and vitamin E in Alzheimer disease: a combined P300 event-related potentials/neuropsychologic evaluation over 6 months. *Clinical neuropharmacology* 2001; **24**(1): 31-42.
101. Wilcock G, Howe I, Coles H, et al. A long-term comparison of galantamine and donepezil in the treatment of Alzheimer's disease. *Drugs & aging* 2003; **20**(10): 777-89.
102. Wilkinson D. A Clinical Study Evaluating the Effects of Memantine on Brain Atrophy in Patients With Alzheimer's Disease; 2012.
103. Wilkinson D, Murray J. Galantamine: a randomized, double-blind, dose comparison in patients with Alzheimer's disease. *International journal of geriatric psychiatry* 2001; **16**(9): 852-7.

For peer review only - http://bmjopen.bmj.com/site/about/guidelines.xhtml

104. Wilkinson DG, Passmore AP, Bullock R, et al. A multinational, randomised, 12-week, comparative study of donepezil and rivastigmine in patients with mild to moderate Alzheimer's disease. *International journal of clinical practice* 2002; **56**(6): 441-6.
105. Winblad B, Cummings J, Andreasen N, et al. A six-month double-blind, randomized, placebo-controlled study of a transdermal patch in Alzheimer's disease--rivastigmine patch versus capsule. *International journal of geriatric psychiatry* 2007; **22**(5): 456-67.
106. Winblad B, Engedal K, Soininen H, et al. A 1-year, randomized, placebo-controlled study of donepezil in patients with mild to moderate AD. *Neurology* 2001; **57**(3): 489-95.
107. Winblad B, Kilander L, Eriksson S, et al. Donepezil in patients with severe Alzheimer's disease: double-blind, parallel-group, placebo-controlled study. *Lancet (London, England)* 2006; **367**(9516): 1057-65.
108. Zhang Y, Rong-jia Y, Ju-ying G, H. G, Chao-mei W. Effects of aricept on the treatment of Alzheimer disease evaluated by skull multi-slice helical CT. *Chin J Clin Rehab* 2005; **9**(25): 20-1.
109. Zhang Z, Yu L, Gaudig M, Schäuble B, Richarz U. Galantamine versus donepezil in Chinese patients with Alzheimer's disease: results from a randomized, double-blind study. *Neuropsychiatric disease and treatment* 2012; **8**: 571-7.

16 Companion Reports

110. Aronson S, Van Baelen B, Kavanagh S, Schwalen S. Optimal dosing of galantamine in patients with mild or moderate Alzheimer's disease: post Hoc analysis of a randomized, double-blind, placebo-controlled trial. *Drugs & aging* 2009; 26(3): 231-9.
111. Cummings JL, Schneider L, Tariot PN, Kershaw PR, Yuan W. Reduction of behavioral disturbances and caregiver distress by galantamine in patients with Alzheimer's disease. *The American journal of psychiatry* 2004; 161(3): 532-8.
112. Feldman H, Gauthier S, Hecker J, et al. Economic evaluation of donepezil in moderate to severe Alzheimer disease. *Neurology* 2004; 63(4): 644-50.
113. Feldman H, Gauthier S, Hecker J, et al. Efficacy and safety of donepezil in patients with more severe Alzheimer's disease: a subgroup analysis from a randomized, placebo-controlled trial. *International journal of geriatric psychiatry* 2005; 20(6): 559-69.
114. Gaudig M, Richarz U, Han J, Van Baelen B, Schäuble B. Effects of galantamine in Alzheimer's disease: double-blind withdrawal studies evaluating sustained versus interrupted treatment. *Current Alzheimer research* 2011; 8(7): 771-80.
115. Gauthier S, Feldman H, Hecker J, Vellas B, Emir B, Subbiah P. Functional, cognitive and behavioral effects of donepezil in patients with moderate Alzheimer's disease. *Current medical research and opinion* 2002; 18(6): 347-54.
116. Grossberg GT, Farlow MR, Meng X, Velting DM. Evaluating high-dose rivastigmine patch in severe Alzheimer's disease: analyses with concomitant memantine usage as a factor. *Current Alzheimer research* 2015; 12(1): 53-60.
117. Han HJ, Kim BC, Lee JY, et al. Response to rivastigmine transdermal patch or memantine plus rivastigmine patch is affected by apolipoprotein E genotype in Alzheimer patients. *Dementia and geriatric cognitive disorders* 2012; 34(3-4): 167-73.
118. Jelic V, Haglund A, Kowalski J, Langworth S, Winblad B. Donepezil treatment of severe Alzheimer's disease in nursing home settings. A responder analysis. *Dementia and geriatric cognitive disorders* 2008; 26(5): 458-66.
119. Kumar V, Anand R, Messina J, Hartman R, Veach J. An efficacy and safety analysis of Exelon in Alzheimer's disease patients with concurrent vascular risk factors. *European journal of neurology* 2000; 7(2): 159-69.
120. Ott BR, Blake LM, Kagan E, Resnick M. Open label, multicenter, 28-week extension study of the safety and tolerability of memantine in patients with mild to moderate Alzheimer's disease. *Journal of neurology* 2007; 254(3): 351-8.

For peer review only - http://bmjopen.bmj.com/site/about/guidelines.xhtml

For peer review only

121. Reisberg B, Doody R, Stöffler A, Schmitt F, Ferris S, Möbius HJ. A 24-week open-label extension study of memantine in moderate to severe Alzheimer disease. *Archives of neurology* 2006; 63(1): 49-54.

122. Schwam E, Xu Y. Cognition and function in Alzheimer's disease: identifying the transitions from moderate to severe disease. *Dementia and geriatric cognitive disorders* 2010; 29(4): 309-16.

123. van Dyck CH, Tariot PN, Meyers B, Malca Resnick E. A 24-week randomized, controlled trial of memantine in patients with moderate-to-severe Alzheimer disease. *Alzheimer disease and associated disorders* 2007; 21(2): 136-43.

124. Wimo A, Winblad B, Engedal K, et al. An economic evaluation of donepezil in mild to moderate Alzheimer's disease: results of a 1-year, double-blind, randomized trial. *Dementia and geriatric cognitive disorders* 2003; 15(1): 44-54.

125. Winblad B, Grossberg G, Frölich L, et al. IDEAL: a 6-month, double-blind, placebo-controlled study of the first skin patch for Alzheimer disease. *Neurology* 2007; 69(4 Suppl 1): S14-22.

For peer review only

For peer review only - http://bmjopen.bmj.com/site/about/guidelines.xhtml

Appendix 3: Studies with available IPD but insufficient data to be included in the analysis

A study[1] of 859 participants comparing transdermal rivastigmine vs. placebo included only IPD for the placebo arm. Another study[2] of 285 participants comparing 22·5 mg of galantamine vs. 30 mg of galantamine vs. 45 mg of galantamine vs. placebo did not provide information about the AE or MMSE outcomes in the shared IPD.

CSDR: Novartis (study: NVT_SA_ENA713D1301) – Nakamura 2011

The study compares rivastigmine patch vs. placebo, but includes data only on placebo. Hence, we cannot conduct an analysis to convert data on their aggregated form so that to be included in our network meta-analysis. The IPD of this study included 288 participants in total.
According to the publication, 284 were allocated to the rivastigmine patch 5 cm2 group, 287 to the rivastigmine patch 10 cm2 group, and 288 to the placebo group.

Baseline characteristics of included patients

Characteristics	PLAC	Total	Missing Data	P-value	Outliers
Males	92 (32 %)	92 (32 %)	No	-	No
Age, mean (SD)	74.6 (7.4)	74.6 (7.4)	No	-	Yes - 1 value
AE, events/sample size	19/288	19/288	No	-	-
Baseline MMSE, mean (SD)	16.6 (2.9)	16.6 (2.9)	Yes - 1 value	-	No
MMSE, mean (SD)	17.5 (3.4)	17.5 (3.4)	No	-	No
Change score, mean (SD)	0.9 (1.6)	0.9 (1.6)	Yes - 2 values	-	Yes - 41 values
Total number of patients	288 (100 %)	288			

YODA: JNJ-Study-GAL-93-01 –Wilkinson 2001

The study compares galantamine 22.5mg, 30mg and 45mg vs placebo. In our analysis we combined galantamine 22.5mg, 30mg and 45mg in a single group. However, we only descriptively can include this study in our paper - not in the network meta-analysis – as it does not provide any info about the AE or MMSE outcomes (only total score for baseline). The IPD of this study included 285 participants in total.
According to the publication, 285 patients were randomized to: galantamine 18mg, 24mg, 36mg/day and placebo. Of the outcomes of interest, publication reported the AE outcome. According to the sponsor there are no differences in the reporting of doses:

- galantamine hydrobromide 7.5 mg =6 mg galantamine base was administered tid i.e galantamine hydrobromide 22.5 mg/d = galantamine base **18mg/day**
- galantamine hydrobromide 10 mg =8 mg galantamine base was administered tid i.e galantamine hydrobromide 30mg/d= galantamine base **24mg/day** and
- galantamine hydrobromide 15 mg =12 mg galantamine base was administered tid i.e galantamine hydrobromide 45mg/d= galantamine base **36mg/day**

Baseline characteristics of included patients

Characteristics	GALA	PLAC	Total	Missing Data	P-value	Outliers
Males	85 (30%)	36 (12%)	121 (42%)	No	<0.001	No
Age, mean (SD)	73.5 (8.2)	74.2 (9.0)	73.8 (8.5)	No	0.242	Yes - 1 value
AE, events/sample size*	-	-	-	-	-	-
Baseline MMSE, mean (SD)	18.6 (3.2)	18.8 (3.1)	18.7 (3.2)	No	0.616	No
MMSE, mean (SD)	-	-	-	-	-	-
Change score, mean (SD)	-	-	-	-	-	-
Total number of patients	198 (69%)	87 (31%)	285 (100%)			

*AE in publication is as follows, PLAC: 3/87, GALA 18mg: 6/88, GALA 24mg: 0/56, GALA 36mg: 5/54

[1]Nakamura Y, Imai Y, Shigeta M, et al. A 24-week, randomized, double-blind, placebo-controlled study to evaluate the efficacy, safety and tolerability of the rivastigmine patch in Japanese patients with Alzheimer's disease. Dement Geriatr Cogn Dis Extra 2011; 1(1): 163-79.
[2] Wilkinson D, Murray J. Galantamine: a randomized, double-blind, dose comparison in patients with Alzheimer's disease. Int J Geriatr Psychiatry 2001; 16(9): 852-7.

For peer review only - http://bmjopen.bmj.com/site/about/guidelines.xhtml

Appendix 4: List of studies requested and sponsor response

Sponsor	Author, year	Interventions compared (dosage mg)*	Sponsor Response	IPD Received
Abbvie	Gault, 2015	Placebo/No treatment, Donepezil (10 mg)	Available	Yes
	Haig, 2014	Placebo/No treatment, Donepezil (5 – 10 mg)	Available	Yes
	Marek, 2014	Placebo/No treatment, Donepezil (5 – 10 mg)	Unavailable (Cannot share data (Potential business considerations under review))	No
AstraZeneca	Frolich, 2011	Placebo/No treatment, Donepezil (5 – 10 mg)	Available	No
Daiichi-Sankyo	Shimizu, 2015	Donepezil (5 mg), Galantamine (24 mg), Rivastigmine (18 mg)	Unavailable (Do not own data)	No
Eisai	Black, 2007	Placebo/No treatment, Donepezil (5 – 10 mg)	Available	Yes
	Burns, 1999	Placebo/No treatment, Donepezil (5 – 10 mg)	Unavailable (Cannot share data (Old study))	No
	Feldman, 2001	Placebo/No treatment, Donepezil (5 – 10 mg)	Unavailable (Do not own data)	No
	Feldman, 2004	Placebo/No treatment, Donepezil (5 – 10 mg)	Unavailable (Do not own data)	No
	Feldman, 2005	Placebo/No treatment, Donepezil (5 – 10 mg)	Unavailable (Do not own data)	No
	Gauthier, 2002	Placebo/No treatment, Donepezil (5 – 10 mg)	Unavailable (Do not own data)	No
	Holmes, 2004	Placebo/No treatment, Donepezil (10 mg)	Unavailable (Do not own data)	No
	Homma, 2008	Placebo/No treatment, Donepezil (10 mg)	Unavailable (Cannot share data (Old study))	No
	Johannsen, 2006	Placebo/No treatment, Donepezil (10 mg)	Unavailable (Do not own data)	No
	Jones, 2004	Donepezil (5 – 10 mg), Galantamine (8 – 24 mg)	Unavailable (Cannot share data (Old study))	No
	Mohs, 2001	Placebo/No treatment, Donepezil (5 – 10 mg)	Unavailable (Cannot share data (Old study))	No
	Rogers, 1996	Placebo/No treatment, Donepezil (5 mg)	Unavailable (Cannot share data (Old study))	No
	Rogers, 1998	Placebo/No treatment, Donepezil (10 mg)	Unavailable (Cannot share data (Old study))	No
	Rogers, 1998	Placebo/No treatment, Donepezil (10 mg)	Unavailable (Cannot share data (Old study))	No
	Schwam, 2010	Placebo/No treatment, Donepezil (5 – 10 mg)	Unavailable (Do not own data)	No
	Seltzer, 2004	Donepezil (5 – 10 mg), Placebo/No treatment	Unavailable (Cannot share data (Old study))	No
	Shimizu, 2015	Donepezil (5 mg), Galantamine (24 mg), Rivastigmine (18 mg)	Unavailable (Do not own data)	No
	Sole-Padulles, 2013	Placebo/No treatment, Donepezil (5 – 10 mg)	Unavailable (Do not own data)	No
	Tariot, 2001	Placebo/No treatment, Donepezil (5 – 10 mg)	Unavailable (Cannot share data (Old study))	No
	Wilkinson, 2002	Donepezil (5 – 10 mg), Rivastigmine (6 – 12 mg)	Unavailable (Do not own data)	No
Forest Laboratories/Allergen	Grossberg, 2013	Donepezil (NR) + Rivastigmine (13.3 mg) + Galantamine + Placebo, Donepezil (NR) + Rivastigmine (4.6 mg) + Galantamine (NR)+ Memantine (NR)	Unavailable (Cannot share data (No details provided))	No
	Ott, 2007	Placebo/No treatment, Memantine (5 -20 mg)	Unavailable (Cannot share data (No details provided))	No
	Peskind, 2006	Placebo/No treatment, Memantine (5 -20 mg)	Unavailable (Cannot share data (No details provided))	No
	Saxton, 2012	Placebo/No treatment, Memantine (20 mg)	Unavailable (Cannot share data (No details provided))	No
	van Dyck, 2007	Placebo/No treatment, Memantine (20 mg)	Unavailable (Cannot share data (No details provided))	No
GlaxoSmithKline	Gold, 2010	Placebo/No treatment, Donepezil (10 mg)	Available	Yes
	Maher-Edwards, 2011	Placebo/No treatment, Donepezil (5 – 10 mg)	Unavailable (Do not own data)	No
Janssen	Ancoli-Israel, 2005	Donepezil (10 mg), Galantamine (8 mg)	Unavailable (Cannot identify study)	No
	Aronson, 2009	Placebo/No treatment, Galantamine (16 – 24 mg)	Unavailable (Cannot identify study)	No
	Burns, 2009	Placebo/No treatment, Galantamine (8-24 mg)	Available	Yes
	Cummings, 2004	Placebo/No treatment, Galantamine (4, 8, 12 mg)	Available	Yes
	Gaudig, 2011	Placebo/No treatment, Galantamine (8 mg)	Unavailable (Cannot identify study)	No
	Hager K, 2014	Placebo/No treatment, Galantamine (8 – 24 mg)	Available	Yes
	Kadir, 2008	Placebo/No treatment, Galantamine (16 – 24 mg)	Unavailable (Cannot identify study)	No
	Likitjaroen, 2012	Placebo/No treatment, Galantamine (8 – 24 mg)	Unavailable(Do not own data)	No
	Rockwood, 2001	Placebo/No treatment, Galantamine (24, 32 mg)	Available	Yes
	Rockwood, 2006	Placebo/No treatment, Galantamine (16 – 24 mg)	Unavailable (IPD not available)	No
	Scarpini, 2011	Placebo/No treatment, Galantamine (16 mg)	Unavailable (IPD not available)	No

For peer review only - http://bmjopen.bmj.com/site/about/guidelines.xhtml

Sponsor	Author, year	Interventions compared (dosage mg)*	Sponsor Response	IPD Received
	Shimizu, 2015	Donepezil (5 mg), Galantamine (24 mg), Rivastigmine (18 mg)	Unavailable (Cannot identify study)	No
	Tariot, 2000	Placebo/No treatment, Galantamine (8 mg)	Unavailable (Cannot identify study)	No
	Wilcock, 2003	Donepezil (5 – 10 mg), Galantamine (16 – 24 mg)	Unavailable (Cannot identify study)	No
	Zhang, 2012	Donepezil (5 – 10 mg), Galantamine (6 – 16 mg or 6 – 24 mg)	Unavailable (IPD not available)	No
	Wilkinson, 2001	Placebo/No treatment, Galantamine (18 - 36 mg)	Available	Yes
Lundbeck	Bakchine, 2008	Placebo/No treatment, Memantine (20 mg)	Available	Yes
	Fox, 2012	Placebo/No treatment, Memantine (5 – 20 mg)	Unavailable (Do not own data)	No
	Herrmann, 2013	Placebo/No treatment, Memantine (5 – 20 mg)	Available	Yes
	Lorenzi, 2011	Placebo/No treatment, Memantine (5 – 20 mg)	Unavailable (Do not own data)	No
	Wilkinson, 2012	Placebo/No treatment, Memantine (5 – 20 mg)	Available	Yes
Merz	Reisberg, 2003	Placebo/No treatment, Memantine (20 mg)	No response from sponsor	No
	Reisberg, 2006	Placebo/No treatment, Memantine (20 mg)	No response from sponsor	No
	Schmidt, 2008	Placebo/No treatment, Memantine (5 – 20 mg)	No response from sponsor	No
	Winblad, 2007	Placebo/No treatment, Rivastigmine (3 – 12 mg)	No response from sponsor	No
Novartis	Agid, 1998	Placebo/No treatment, Rivastigmine (6 mg)	Unavailable (Cannot identify study)	No
	Blesa González, 2011	Placebo/No treatment, Rivastigmine (6 – 12 mg)	Unavailable (Cannot share data)	No
	Choi, 2011	Placebo/No treatment, Memantine (5 – 20 mg)	Unavailable (Do not own data)	No
	Corey-Bloom, 1998	Placebo/No treatment, Rivastigmine (6 – 12 mg)	Unavailable (Cannot identify study)	No
	Farlow, 2013	Rivastigmine (4.6 - 13.3 mg), Rivastigmine (4.6 mg) + Memantine (20 mg)	Unavailable (Cannot share data (Phase 4 study))	No
	Feldman, 2007	Placebo/No treatment, Rivastigmine (2 – 12 mg)	Unavailable (Cannot identify study)	No
	Grossberg, 2015	Rivastigmine (4.6 - 13.3 mg), Rivastigmine (4.6 mg) + Memantine (20 mg)	Unavailable (Cannot share data (Phase 4 study))	No
	Han, 2012	Placebo/No treatment, Memantine (5 – 20 mg)	Unavailable (Cannot identify study)	No
	Kumar, 2000	Placebo/No treatment, Rivastigmine (1 – 12 mg)	Unavailable (Cannot identify study)	No
	Nakamura, 2011	Placebo/No treatment, Rivastigmine (4.5 – 9.5 mg)	Available	Yes
	Nordberg, 2009	Donepezil (5 – 10 mg), Galantamine (8 – 24 mg), Rivastigmine (3 – 12 mg)	Unavailable (Cannot share data (Phase 4 study))	No
	Shimizu, 2015	Donepezil (5 mg), Galantamine (24 mg), Rivastigmine (18 mg)	Unavailable (Cannot identify study)	No
	Winblad, 2007	Placebo/No treatment, Rivastigmine (3 – 12 mg)	Available	Yes
ONO	Nakamura, 2011	Placebo/No treatment, Rivastigmine (4.5 – 9.5 mg)	No response from sponsor	No
Pfizer	Black, 2007	Placebo/No treatment, Donepezil (10 mg)	Unavailable (Do not own data)	No
	Feldman, 2001	Placebo/No treatment, Donepezil (5 – 10 mg)	Available	No
	Feldman, 2004	Placebo/No treatment, Donepezil (5 – 10 mg)	Unavailable (Cannot identify study)	No
	Feldman, 2005	Placebo/No treatment, Donepezil (5 – 10 mg)	Unavailable (Cannot identify study)	No
	Gauthier, 2002	Placebo/No treatment, Donepezil (5 – 10 mg)	Unavailable (Cannot identify study)	No
	Holmes, 2004	Placebo/No treatment, Donepezil (10 mg)	Unavailable (Cannot identify study)	No
	Jelic, 2008	Placebo/No treatment, Donepezil (5 – 10 mg)	Unavailable (Cannot identify study)	No
	Johannsen, 2006	Placebo/No treatment, Donepezil (10 mg)	Unavailable (Cannot identify study)	No
	Jones, 2004	Donepezil, Galantamine (8 – 24 mg)	Unavailable (Cannot identify study)	No
	Mohs, 2001	Placebo/No treatment, Donepezil (5 – 10 mg)	Unavailable (Cannot identify study)	No
	Schwam, 2010	Placebo/No treatment, Donepezil (5 – 10 mg)	Unavailable (Cannot identify study)	No
	Seltzer, 2004	Donepezil (5 – 10 mg), Placebo/No treatment	Unavailable (Cannot identify study)	No
	Sole-Padulles, 2013	Placebo/No treatment, Donepezil (5 – 10 mg)	Unavailable (Cannot identify study)	No
	Tariot, 2001	Placebo/No treatment, Donepezil (5 – 10 mg)	Unavailable (Cannot identify study)	No

For peer review only - http://bmjopen.bmj.com/site/about/guidelines.xhtml

Sponsor	Author, year	Interventions compared (dosage mg)*	Sponsor Response	IPD Received
	Wilkinson, 2002	Donepezil (5 – 10 mg), Rivastigmine (6 – 12 mg)	Unavailable (Cannot identify study)	No
	Wimo, 2003	Placebo/No treatment, Donepezil (5 – 10 mg)	Unavailable (Cannot identify study)	No
	Winblad, 2001	Placebo/No treatment, Donepezil (5 – 10 mg)	Unavailable (Cannot identify study)	No
	Winblad, 2006	Placebo/No treatment, Donepezil (5 – 10 mg)	Unavailable (Cannot identify study)	No
Roivant	Maher-Edwards, 2011	Placebo/No treatment, Donepezil (5 – 10 mg)	No response from sponsor	No
Shire Pharmaceuticals	Wilcock, 2003	Donepezil (5 – 10 mg), Galantamine (16 – 24 mg)	Unavailable (Do not own data)	No
	Wilkinson, 2001	Placebo/No treatment, Galantamine (24 mg)	Unavailable (Do not own data)	No
Takeda	Shimizu, 2015	Donepezil (5 mg), Galantamine (24 mg), Rivastigmine (18 mg)	Unavailable (Do not own data)	No
Non-Pharmaceutical	Andersen, 2012	Placebo/No treatment, Donepezil (5 – 10 mg)	NA	No
	Araki, 2014	Placebo/No treatment, Donepezil (NR) + Memantine (5 – 20 mg)	NA	No
	Burns, 2011	Placebo/No treatment, Donepezil (5 – 10 mg)	NA	No
	Dysken, 2014	Placebo/No treatment, Memantine (20 mg)	Available	No
	Greenberg, 2000	Placebo/No treatment, Donepezil (5 mg)	Unavailable (Need to contact PI)	No
	Howard, 2007	Placebo/No treatment, Donepezil (5 – 10 mg)	Unavailable (Do not own data)	No
	Howard, 2012	Donepezil (10 mg) + Memantine (5 – 20 mg), Donepezil (10 mg) + Placebo	Unavailable (Do not own data)	No
	Mowla, 2007	Placebo/No treatment, Rivastigmine (3 – 12 mg)	NA	No
	Peters, 2015	Galantamine (24 mg) + Placebo, Galantamine (24 mg) + Memantine (20 mg)	NA	No
Not reported	Cretu, 2008	Placebo/No treatment, Memantine (5 – 20 mg)	NA	No
	Fuschillo, 2001	Donepezil (5 mg), Rivastigmine (6 – 9 mg)	NA	No
	Hernández, 2007	Placebo/No treatment, Donepezil (10 mg)	NA	No
	Homma, 1998	Donepezil (3 – 5 mg), Placebo/no treatment	NA	No
	Hong, 2006	Placebo/No treatment, Galantamine (8 – 24 mg)	NA	No
	Hu, 2006	Donepezil (5 mg), Memantine (5 – 10 mg)	NA	No
	Kano, 2013	Donepezil(10 mg), Donepezil (10 mg) + Memantine (20 mg)	NA	No
	Karaman, 2005	Placebo/No treatment, Rivastigmine (3 – 12 mg)	NA	No
	Mazza, 2006	Placebo/No treatment, Donepezil (5 mg)	NA	No
	Moretti, 2014	Placebo/No treatment, Rivastigmine (3 – 12 mg)	NA	No
	Nakano, 2001	Placebo/No treatment, Donepezil (5 mg)	NA	No
	Pakdaman H, 2015	Donepezil (NR), Galantamine (NR), Rivastigmine (NR)	NA	No
	Peng, 2005	Placebo/No treatment, Donepezil (5 mg)	NA	No
	Shao, 2015	Memantine (5 – 10 mg)+ Placebo, Rivastigmine (1.5 – 3 mg) + Memantine (5 – 10 mg), Donepezil (5 – 10 mg) + Memantine (5 – 10 mg), Galantamine (2 – 6 mg) + Memantine (5 – 10 mg)	NA	No
	Thomas, 2001	Donepezil (5 – 10 mg), Rivastigmine (6 – 12 mg)	NA	No
	Zhang-Yi, 2005	Placebo/No treatment, Donepezil (5 mg)	NA	No

Abbreviations: NA, not applicable; NPH, neutral protamine Hagedorn; NR, not reported; PI, principal investigator

* In studies that examined different dosages of the same intervention, we selected the dosages that were consistent with those approved for use in Canada.

For peer review only - http://bmjopen.bmj.com/site/about/guidelines.xhtml

Appendix 5: Study characteristics of the included RCTs

Study	Country of conduct	Sample size; Longest duration of follow-up (weeks)	Treatments compared; Outcomes	Funding information	Date of randomization; Date trial opened; Randomization ratio	IPD available; Reasons for not providing IPD by the data providers
Agid, 1998	12 countries - Austria, Belgium, Czechoslovakia, Denmark, Finland, France, Germany, Ireland, Norway, Sweden, Switzerland, and the UK	402; 13	Rivastigmine, Placebo/No treatment; MMSE, Nausea, Vomiting, Diarrhea, AEs, Headaches	Industry-sponsored	Not reported; Not reported; Not reported	No; Cannot identify study
Ancoli-Israel, 2005	USA	63; 8	Galantamine, Donepezil; CIBIC-plus, Mortality, Nausea, Diarrhea, AEs, Headaches	Industry-sponsored	Not reported; Not reported; Not reported	No; Cannot identify study
Andersen, 2012	Norway	180; 52	Donepezil, Placebo; MMSE, ADAS-cog	Publicly-sponsored	Not reported; June 2003; Not reported	No; NA
Araki, 2014	Japan	37; 24	Donepezil + Memantine, Placebo; MMSE, NPI	Publicly-sponsored	Not reported; Not reported; Not reported	No; NA
Bakchine, 2008	12 countries -Austria, Belgium, Denmark, Finland, France, Greece, Lithuania, the Netherlands, Poland, Spain, Sweden and UK	470; 24	Memantine, Placebo/no treatment; ADAS-cog, ADCS-ADL, NPI, CIBIC-plus, Mortality, AEs, Headaches, Falls	Industry-sponsored	Not reported; Not reported; Not reported	Yes; NA
Black, 2007	5 countries - USA, Canada, France, UK, Australia	343; 24	Donepezil, Placebo/No treatment; MMSE, ADCS-ADL, NPI, CIBIC-plus, Nausea, Vomiting, Diarrhea, AEs	Industry-sponsored	Not reported; January 2001; Not reported	Yes; Do not own data
Blesa González, 2011	Spain	139; 12	Rivastigmine Patch, Rivastigmine Oral; MMSE, Nausea, Vomiting, Diarrhea	Industry-sponsored	Not reported; Not reported; Not reported	No; Cannot share data (Phase 4 study)
Burns, 1999	Australia, Belgium, Canada, France, Germany,Ireland, New Zealand, South Africa and the UK	818; 30	Donepezil, Placebo/no treatment; ADAS-cog, CIBIC-plus, Mortality, Diarrhea, Nausea, AEs, Vomiting	Industry-sponsored	Not reported; Not reported; Not reported	No; Cannot share data (Old study)
Burns, 2009	Belgium, Finland, France, Italy, Norway, Netherlands, Spain, Sweden, Switzerland, UK	407; 26	Galantamine, Placebo/no treatment; Mortality, Nausea, Vomiting, Diarrhea, AEs, Headaches, Falls	Industry-sponsored	Not reported; December 2003; Not reported	Yes; NA
Burns, 2011	UK	62; 12	Donepezil, Placebo/no treatment; NPI, AEs	Publicly-sponsored	Not reported; January 2006; Not reported	No; NA
Choi, 2011	South Korea	171; 16	Memantine, Placebo/No treatment; MMSE, ADAS-cog, ADCS-ADL, NPI, AEs, Nausea, Diarrhea, Vomiting, Headaches	Publicly-sponsored + Industry-sponsored	Not reported; December 2008; Not reported	No; Do not own data
Corey-Bloom, 1998	USA	699; 26	Rivastigmine, Placebo/No treatment; MMSE, ADAS-cog, CIBIC-plus, Mortality, Nausea, Vomiting	Industry-sponsored	Not reported; Not reported; Not reported	No; Cannot identify study

For peer review only - http://bmjopen.bmj.com/site/about/guidelines.xhtml

Cretu, 2008	Romania	43;<br>24	Memantine, Placebo/No treatment;<br>MMSE, ADAS-cog, NPI	NA	Not reported;<br>Not reported;<br>Not reported	No;<br>NR
Dysken, 2014	USA	307;<br>26-208	Memantine, Placebo;<br>MMSE, ADAS-cog, ADCS-ADL, NPI, Mortality, AEs	Publicly-sponsored	Not reported;<br>August 2007;<br>1:1:1:1	No;<br>NA
Farlow, 2013	USA	716;<br>24	Rivastigmine + Memantine, Rivastigmine;<br>NPI, Mortality, Falls, Vomiting, Diarrhea, Nausea	Industry-sponsored	Not reported;<br>July 2009;<br>1:1	No;<br>Cannot share data (Phase 4 study)
Feldman, 2001	Canada, Australia, France	290;<br>24	Donepezil, Placebo/No treatment;<br>MMSE, NPI, CIBIC-plus, Mortality, Vomiting, Nausea, Diarrhea, AEs, Headaches	Industry-sponsored	Not reported;<br>Not reported;<br>"50/50 split"	No;<br>NA
Feldman, 2007	Australia, Canada, Ireland, Italy, South Africa, UK	450;<br>26	Rivastigmine, Placebo/No treatment;<br>MMSE, ADAS-cog, CIBIC-plus, AEs, Bradycardia, Headaches, Vomiting, Diarrhea, Nausea	Industry-sponsored	Not reported;<br>Not reported;<br>1:1:1	No;<br>Cannot identify study
Fox, 2012	UK	149;<br>12	Memantine, Placebo;<br>MMSE, NPI, Mortality	Industry-sponsored	Not reported;<br>September 2007;<br>"assigned with equal probability"	No;<br>Unavailable (Do not own data)
Frolich, 2011	Austria, Belgium, Bulgaria, Czech Republic, Germany, Romania, Russia, Spain, UK, Canada	324;<br>12	Donepezil, Placebo/No treatment;<br>MMSE, ADAS-cog, Nausea, Vomiting, Diarrhea, Headaches	Industry-sponsored	Not reported;<br>July 2007;<br>Not reported	No;<br>Available
Fuschillo, 2001	Italy	27;<br>30	Donepezil, Rivastigmine;<br>MMSE, ADAS-cog, Headaches, Vomiting, Diarrhea, Nausea	NA	Not reported;<br>Not reported;<br>Not reported	No;<br>NR
Gault, 2015	USA, Bulgaria, Czech Republic, Slovakia, UK, South Africa	136;<br>14	Donepezil, Placebo;<br>MMSE, ADAS-cog, ADCS-ADL, NPI, CIBIC-plus, Mortality, AEs, Bradycardia, Falls, Headaches, Vomiting, Diarrhea, Nausea	Industry-sponsored	Not reported;<br>October 2009;<br>Not reported	Yes;<br>Available
Gold, 2010	Austria, Bulgaria, Chile, China, Croatia, Estonia, Germany, Greece, Hungary, Mexico, New Zealand, Pakistan, Peru, Republic of the Philippines, Puerto Rico, Republic of Korea, Russian Federation, UK and USA	248;<br>24	Donepezil, Placebo/no treatment;<br>ADAS-cog, CIBIC-plus, Mortality, Headaches, Nausea, Diarrhea, AEs	Industry-sponsored	Not reported;<br>February 2007;<br>2:2:2:1	Yes;<br>Available
Greenberg, 2000	USA	103;<br>24	Donepezil, Placebo/no treatment;<br>ADAS-cog, AEs, Diarrhea, Nausea	Publicly-sponsored	Not reported;<br>Not reported;<br>Not reported	No;<br>Contact PI
Grossberg, 2013	Argentina, USA, Mexico, Chile	676;<br>24	Donepezil + Rivastigmine + Galantamine + Memantine, Donepezil + Rivastigmine + Galantamine + Placebo;<br>NPI, CIBIC-plus, Mortality, Falls,	Industry-sponsored	Not reported;<br>June 2005;<br>1:1	No;<br>Cannot share dat

For peer review only - http://bmjopen.bmj.com/site/about/guidelines.xhtml

			Headaches, Vomiting, Diarrhea, Nausea, AEs			
Hager K, 2014	Czech Republic, Estonia, France, Germany, Greece, Italy, Latvia, Lithuania, Romania, Russia, Slovakia, Slovenia, Ukraine	2045; 104	Galantamine, Placebo; MMSE, Mortality, Headaches, Vomiting, Diarrhea, Nausea, AEs	Industry-sponsored	Not reported; May 2008; 1:1	Yes; NA
Haig, 2014	Russia, Ukraine	123; 12	Donepezil, Placebo; MMSE, ADAS-cog, ADCS-ADL, NPI, Headaches, Nausea, AEs	Industry-sponsored	Not reported; Not reported; 1:1:1	Yes; NA
Hernández, 2007	Spain	20; 48	Donepezil, Placebo/No treatment; MMSE, ADAS-cog	NA	Not reported; Not reported; Not reported	No; NR
Herrmann, 2013	Canada	369; 24	Memantine, Placebo; NPI, Mortality, Falls, Nausea, AEs	Industry-sponsored	Not reported; December 2003; "equally allocated"	Yes; NA
Holmes, 2004	UK	96; 24	Donepezil, Placebo/No treatment; MMSE, NPI	Industry-sponsored	Not reported; Not reported; 3:2	No; Cannot identify study
Homma, 1998	Japan	187; 12	Donepezil, Placebo/no treatment; ADAS-cog, Mortality, AEs, Headaches	NA	Not reported; Not reported; Not reported	No; NR
Homma, 2008	Japan	267; 24	Donepezil, Placebo/no treatment; ADCS-ADL, CIBIC-plus, Mortality, AEs, Falls, Vomiting, Diarrhea	Industry-sponsored	Not reported; Not reported; 1:1:1	No; Cannot share data (Old study)
Hong, 2006	China	218; 16	Galantamine, Placebo/no treatment; ADAS-cog, ADCS-ADL, NPI, AEs	NA	Not reported; Not reported; Not reported	No; NR
Howard, 2007	England	259; 12	Donepezil, Placebo/No treatment; MMSE, NPI, Mortality, Falls, Diarrhea	Publicly-sponsored	Not reported; November 2003; "probability ratios of 0.75 and 0.25 to assign treatment"	No; NA
Howard, 2012	Europe	295; 52	Donepezil + Placebo, Donepezil + Memantine; MMSE, Mortality, AEs, Falls	Publicly-sponsored	Not reported; February 2008; Not reported	No; Do not own data
Hu, 2006	China	97; 16	Memantine, Donepezil; MMSE	NA	Not reported; Not reported; Not reported	No; NA
Johannsen, 2006	Belgium, Denmark, Germany, Greece, Hungary, Iceland, The Netherlands, Poland, USA	202; 48	Donepezil, Placebo/No treatment; MMSE, ADAS-cog, NPI, Headaches, Diarrhea, Nausea	Industry-sponsored	Not reported; February 1999; Not reported	No; Do not own data
Jones, 2004	UK, Finland, Germany and Norway	120; 12	Donepezil, Galantamine; MMSE, ADAS-cog, Headaches, Vomiting, Diarrhea, Nausea, AEs	Industry-sponsored	Not reported; Not reported; 1:1	No; Cannot share data (Old study)
Kadir, 2008	Sweden	18; 48	Galantamine, Placebo/No treatment; MMSE, ADAS-cog	Industry-sponsored + Other	Not reported; Not reported; Not reported	No; Cannot identify study

For peer review only - http://bmjopen.bmj.com/site/about/guidelines.xhtml

For peer review only

Kano, 2013;	Japan	30;<br>28	Donepezil, Donepezil + Memantine ;<br>MMSE	NA	Not reported;<br>August 2011;<br>Not reported	No;<br>NR
Karaman, 2005	Turkey	44;<br>52	Rivastigmine, Placebo/No treatment;<br>MMSE, ADAS-cog, ADAS-ADL, CIBIC-plus, Headaches, Vomiting, Nausea	NA	Not reported;<br>Not reported;<br>Not reported	No;<br>NR
Likitjaroen, 2012	Germany	25;<br>26	Galantamine, Placebo;<br>MMSE	Publicly-sponsored + Industry-sponsored	Not reported;<br>September 2006;<br>Not reported	No;<br>Do not own data
Lorenzi, 2011	Italy	15;<br>24	Memantine, Placebo/No treatment;<br>MMSE	Publicly-sponsored + Industry-sponsored	Not reported;<br>Not reported;<br>Not reported	No;<br>Do not own data
Maher-Edwards, 2011	Austria, Bulgaria, Chile, Estonia, Germany, Russia, Slovakia, and UK	129;<br>24	Donepezil, Placebo/no treatment;<br>ADAS-cog, CIBIC-plus, Mortality, AEs, Headaches, Nausea	Industry-sponsored	Not reported;<br>May 2006;<br>1:1:1	No;<br>No response from sponsor
Marek, 2014	UK, Ukraine, South Africa, Russia	132;<br>16	Donepezil, Placebo;<br>MMSE, ADAS-cog, NPI, CIBIC-plus, Mortality, Headaches, Vomiting, Diarrhea, AEs	Industry-sponsored	Not reported;<br>May 2010;<br>"equal proportions"	No;<br>Cannot share data
Mazza, 2006	Italy	51;<br>24	Donepezil, Placebo/No treatment;<br>MMSE	NA	Not reported;<br>March 2003;<br>1:1:1	No;<br>NR
Mohs, 2001	USA	431;<br>54	Donepezil, Placebo/No treatment;<br>MMSE, Mortality, AEs, Headaches, Diarrhea, Nausea	Industry-sponsored	Not reported;<br>Not reported;<br>Not reported	No;<br>Cannot share data
Moretti, 2014	Italy	20;<br>78	Rivastigmine Patch, Rivastigmine Oral;<br>MMSE	NA	Not reported;<br>Not reported;<br>Not reported	No;<br>NA
Mowla, 2007	Iran	81;<br>12	Rivastigmine, Placebo/No treatment;<br>MMSE	Publicly-sponsored	Not reported;<br>Not reported;<br>Not reported	No;<br>NA
Nakamura, 2011	Japan	855;<br>24	Rivastigmine, Placebo/No treatment;<br>MMSE, AEs, Vomiting, Nausea, Diarrhea	Industry-sponsored	Not reported;<br>January 2007;<br>Not reported	Yes;<br>NA
Nakano, 2001	Japan	35;<br>48	Donepezil, Placebo/No treatment;<br>MMSE	NA	Not reported;<br>Not reported;<br>Not reported	No;<br>NR
Nordberg, 2009	USA	63;<br>13	Rivastigmine, Donepezil, Galantamine;<br>AEs, Headaches, Vomiting, Diarrhea, Nausea	Industry-sponsored	Not reported;<br>Not reported;<br>1:1:1	No;<br>Cannot share data
Pakdaman H, 2015	Iran	198;<br>68.8	Donepezil, Galantamine, Rivastigmine;<br>MMSE, ADAS-cog, Mortality,	Industry-sponsored	Not reported;<br>Not reported;<br>Not reported	No;<br>NR

For peer review only - http://bmjopen.bmj.com/site/about/guidelines.xhtml

For peer review only

			Headaches, Vomiting, Diarrhea, Nausea			
Peng, 2005	China	89;<br>12	Donepezil, Placebo/No treatment;<br>MMSE	NA	Not reported;<br>1998;<br>Not reported	No;<br>NR
Peskind, 2006	USA	403;<br>24	Memantine, Placebo/no treatment;<br>ADAS-cog, ADCS-ADL, NPI, CIBIC-plus, Nausea, Vomiting, Diarrhea	Industry-sponsored	Not reported;<br>Not reported;<br>Not reported	No;<br>Cannot share data
Peters, 2015	Europe	226;<br>52	Galantamine + Memantine, Galantamine + Placebo;<br>ADAS-cog, ADCS-ADL, NPI, Mortality, AEs, Falls	Publicly-sponsored	Not reported;<br>Not reported;<br>Not reported	No;<br>NA
Reisberg, 2003	USA	252;<br>28	Memantine, Placebo/No treatment;<br>MMSE, ADCS-ADL, NPI, CIBIC-plus, Mortality, AEs, Diarrhea	Publicly-sponsored + Industry-sponsored	Not reported;<br>August 1998;<br>Not reported	No;<br>No response from sponsor
Rockwood, 2001	Australia, Canada, Great Britian, New Zealand, South Africa, USA	386;<br>12	Galantamine, Placebo/no treatment;<br>ADAS-cog, NPI, CIBIC-plus, Mortality, AEs, Vomiting, Nausea	Industry-sponsored	Not reported;<br>Not reported;<br>Not reported	Yes;<br>NA
Rockwood, 2006	Canada	130;<br>16	Galantamine, Placebo/no treatment;<br>ADAS-cog, CIBIC-plus, AEs, Vomiting, Nausea	Publicly-sponsored + Industry-sponsored	Not reported;<br>November 2001;<br>Not reported	No;<br>IPD not available
Rogers, 1996	USA	161;<br>12	Donepezil, Placebo/No treatment;<br>MMSE, ADAS-cog, Headaches, Diarrhea	Industry-sponsored	Not reported;<br>Not reported;<br>Not reported	No;<br>Cannot share data
Rogers, 1998	USA	468;<br>12	Donepezil, Placebo/No treatment;<br>MMSE, ADAS-cog, CIBIC-plus, AEs, Headaches, Vomiting, Diarrhea, Nausea	Industry-sponsored	Not reported;<br>Not reported;<br>Not reported	No;<br>Cannot share data
Rogers, 1998	USA	473;<br>24	Donepezil, Placebo/No treatment;<br>MMSE, ADAS-cog, CIBIC-plus, Mortality, AEs, Vomiting, Diarrhea, Nausea	Industry-sponsored	Not reported;<br>Not reported;<br>Not reported	No;<br>Cannot share data
Saxton, 2012	Australia, South Africa, New Zealand	264;<br>12	Memantine, Placebo;<br>Mortality, Falls, Headaches, Diarrhea, Nausea, AEs	Industry-sponsored	Not reported;<br>April 2007;<br>Not reported	No;<br>Cannot share data
Scarpini, 2011	Italy	139;<br>96	Galantamine, Placebo/no treatment;<br>Mortality, AEs	Industry-sponsored	Not reported;<br>July 2001;<br>Not reported	No;<br>IPD not available
Schmidt, 2008	Europe	36;<br>52	Memantine, Placebo/No treatment;<br>MMSE, ADAS-cog, ADCS-ADL	Industry-sponsored	Not reported;<br>Not reported;<br>Not reported	No;<br>No response from sponsor
Seltzer, 2004	USA	153;<br>24	Donepezil, Placebo/No treatment;<br>MMSE, ADAS-cog, Diarrhea, Nausea	Industry-sponsored	Not reported;<br>Not reported;<br>Not reported	No;<br>Cannot identify study

For peer review only - http://bmjopen.bmj.com/site/about/guidelines.xhtml

For peer review only

Shao, 2015	China	110;<br>24	Donepezil + Memantine, Galantamine + Memantine, Memantine + Placebo, Rivastigmine + Memantine;<br>MMSE, ADCS-ADL	NA	Not reported;<br>October 2009;<br>Not reported	No;<br>NR
Shimizu, 2015	Japan	75;<br>52	Donepezil, Galantamine, Rivastigmine;<br>MMSE, ADAS-cog, NPI, Headaches, Vomiting, Nausea	Industry-sponsored	Not reported;<br>Not reported;<br>Not reported	No;<br>Do not own data
Sole-Padulles, 2013	Spain	14;<br>13	No treatment, Donepezil;<br>MMSE, NPI	Industry-sponsored	Not reported;<br>Not reported;<br>Not reported	No;<br>Do not own data
Tariot, 2000	USA	978;<br>20	Galantamine, Placebo/no treatment;<br>ADAS-cog, ADCS-ADL, NPI, Mortality, AEs, Vomiting, Diarrhea, Nausea	Industry-sponsored	Not reported;<br>Not reported;<br>Not reported	No;<br>Cannot identify study
Tariot, 2001	USA	208;<br>24	Donepezil, Placebo/No treatment;<br>MMSE, Mortality, AEs, Bradycardia, Headaches, Vomiting, Diarrhea, Nausea	Industry-sponsored	Not reported;<br>Not reported;<br>Not reported	No;<br>Cannot identify study
Thomas, 2001	Italy	40;<br>24	Donepezil, Rivastigmine;<br>MMSE, ADAS-cog	NA	Not reported;<br>Not reported;<br>Not reported	No;<br>NR
Wilcock, 2003	UK	188;<br>52	Galantamine, Donepezil;<br>MMSE, ADAS-cog, Mortality, AEs, Falls, Headaches, Vomiting, Nausea	Industry-sponsored	Not reported;<br>June 2000;<br>Not reported	No;<br>Cannot identify study
Wilkinson, 2001	UK	180;<br>12	Galantamine, Placebo/no treatment;<br>ADAS-cog, AEs, Headaches, Vomiting, Diarrhea, Nausea	Industry-sponsored	Not reported;<br>May 1994;<br>Not reported	Yes;<br>NA
Wilkinson, 2002	UK, South Africa, and Switzerland	111;<br>12	Donepezil, Rivastigmine;<br>MMSE, ADAS-cog, Mortality, AEs, Bradycardia, Headaches, Vomiting, Nausea	Industry-sponsored	Not reported;<br>Not reported;<br>1:1	No;<br>Cannot identify study
Wilkinson, 2012	France, Germany, Switzerland, UK	277;<br>52	Memantine, Placebo/No treatment;<br>MMSE, NPI, Mortality, AEs, Falls	Industry-sponsored	Not reported;<br>September 2005;<br>1:1	Yes;<br>NA
Winblad, 2001	Denmark, Finland, Norway, Sweden, the Netherlands	286;<br>52	Donepezil, Placebo/No treatment;<br>MMSE, AEs, Bradycardia, Headaches, Diarrhea, Nausea	Industry-sponsored	Not reported;<br>Not reported;<br>Not reported	No;<br>Cannot identify study
Winblad, 2006	Sweden	248;<br>24	Donepezil, Placebo/No treatment;<br>MMSE, NPI, Mortality, AEs, Falls, Diarrhea, Nausea	Industry-sponsored	Not reported;<br>October 2002;<br>Not reported	No;<br>Cannot identify study
Winblad, 2007	Chile, Czech Republic, Denmark, Finland, Germany, Guatemala, Israel, Italy, Korea, Mexico, Norway, Peru, Poland, Portugal, Russia, Slovak Republic, Sweden, Taiwan, USA, Uruguay, Venezuela	1190;<br>24	Rivastigmine, Placebo/No treatment;<br>MMSE, ADAS-cog, ADCS-ADL, NPI, Mortality, AEs, Headaches, Vomiting, Diarrhea, Nausea	Industry-sponsored	Not reported;<br>November 2003;<br>Not reported	No;<br>No response from sponsor

For peer review only

Zhang-Yi, 2005	China	120;<br>8	Donepezil, Placebo/No treatment;<br>MMSE	NA	Not reported;<br>Not reported;<br>Not reported	No;<br>NR
Zhang, 2012	China	218;<br>16	Galantamine, Donepezil;<br>MMSE, ADAS-cog, ADCS-ADL, NPI, Mortality, Vomiting, Diarrhea, Nausea, AEs	Industry-sponsored	Not reported;<br>Not reported;<br>Not reported	No;<br>IPD not available

For peer review only

Appendix 6. Characteristics of studies with shared IPD

Study	Provided by	Severity of AD*	Previous response to treatment for AD	Presence of behavioural disturbance	Comorbid conditions	Other medications used	Treatment Group	Males (%)	Age, mean (SD)
Black 2007	CSDR - EISAI	Severe	NR	NR	All patients included the same exact comorbidities	NR	Donepezil	48 (27%)	78 (7.9)
							Placebo	54 (32%)	78 (8.1)
Gold 2010	CSDR - GSK	Mild-Moderate	NR	NR	Multiple reported	Multiple reported	Donepezil	16 (29%)	76.6 (8.2)
							Placebo	49 (46%)	75.5 (8.2)
Winblad 2007	CSDR - Novartis	Mild-Moderate	NR	NR	Multiple reported	Multiple reported	Rivastigmine patch	198 (33 %)	73.9 (8.0)
							Rivastigmine oral	102 (34 %)	72.9 (8.2)
							Placebo	101 (33%)	73.8 (7.5)
Hager 2014	YODA - Janssen	Mild-Moderate	NR	NR	NR	Multiple reported	Galantamine	354 (34%)	73 (8.9)
							Placebo	367 (36%)	73 (8.7)
Rockwood 2001	YODA - Janssen	Mild-Moderate	NR	NR	NR	Multiple reported	Galantamine	113 (43%)	75 (7.3)
							Placebo	58 (46%)	75 (7.6)
Cummings 2004	YODA - Janssen	NR	NR	NR	Multiple reported	Multiple reported	Galantamine	245 (35%)	76.9 (7.8)
							Placebo	108 (38%)	77.2 (7.9)
Burns 2009	YODA - Janssen	Severe	NR	NR	Multiple reported	Multiple reported	Galantamine	42 (20%)	84.0 (6.5)
							Placebo	39 (19%)	83.8 (6.7)
Gault 2015	AbbVie	Mild-Moderate	NR	NR	NR	Multiple reported	Donepezil	37 (54%)	72.4 (8.4)
							Placebo	26 (38%)	73.6 (8.2)
Haig 2014	AbbVie	Mild-Moderate	NR	NR	Multiple reported	Multiple reported	Donepezil	24 (40%)	70 (8.3)
							Placebo	24 (38%)	70 (7.8)
Bakchine 2008	Lundbeck	Mild-Moderate	NR	NR	NR	Multiple reported	Memantine	112 (35%)	74 (7.4)
							Placebo	61 (40%)	73 (6.9)
Herrman 2013	Lundbeck	69 (48%)	NR	NR	NR	Multiple reported	Memantine	77 (42%)	75 (7.9)
							Placebo	77 (41%)	75 (6.9)
Wilkinson 2012	Lundbeck	NR	NR	NR	NR	Multiple reported	Memantine	50 (38%)	74 (8.8)
							Placebo	69 (48%)	74 (7.8)

Additional characteristics of studies with shared IPD

Study	Patients experiencing at least one AE	Missing data in AE outcome	Baseline MMSE, mean (SD)	Final MMSE, mean (SD)	Change score, mean (SD)	Missing data in MMSE outcome	Total number of patients	Reasons for dropouts as indicated in the provided IPD	Time taken for the 1st AE
Black 2007	21	0 (0%)	7.5 (3.3)	8.2 (5.2)	0.63 (3.1)	27 (15%)	176 (51%)	• intercurrent illness (1 [2%] – donepezil = 1; placebo = 0),<br>• request of patient or investigator (4 [7%] –	617 days (range [110, 1292])

For peer review only - http://bmjopen.bmj.com/site/about/guidelines.xhtml

	25	0 (0%)	7.4 (3.6)	7.6 (4.8)	-0.15 (3.5)	27 (16%)	167 (49%)	donepezil = 3; placebo = 1),<br>• patient entered nursing home/facility (5 [9%] – donepezil = 1; placebo =) 4,<br>• due to adverse experience (30 [56%] – donepezil = 15; placebo = 15), and<br>• other (14 [26%] – donepezil = 7; placebo = 7)	691 days (range [78, 1475]).
Gold 2010	6	0 (0%)	20 (3.7)	21 (4.6)	1.11 (2.3)	18 (32%)	56 (34%)	• Adverse Event (16 [39%] – donepezil = 9; placebo = 7),<br>• Lost to Follow-Up (4 [10%] – donepezil = 3; placebo = 1),<br>• Non-compliance (6 [15%] – donepezil = 2; placebo = 4),<br>• Subject decided to withdraw (11 [26%] – donepezil = 4; placebo = 7)	349 days (range [48, 656])
	10	0 (0%)	20.1 (4.2)	20.4 (5.4)	0.08 (2.7)	23 (22%)	107 (66%)		492 days (range [95, 780])
Winblad 2007	83	0 (0%)	16.6 (3.0)	17.7 (4.7)	1 (3.4)	74 (10%)	598 (50 %)	NR	NR
	37	0 (0%)	16.4 (3.1)	17.2 (4.6)	0.8 (3.2)	31 (12%)	297 (25 %)	NR	NR
	45	0 (0%)	16.4 (3.0)	16.4 (5.3)	-0.1 (3.6)	21 (7%)	302 (25 %)	NR	NR
Hager 2014	73	0 (0%)	19.0 (4.1)	17.81 (6.2)	-1.38 (4.3)	228 (22%)	1027 (50%)	NR	NR
	92	0 (0%)	19.0 (4.0)	16.99 (6.3)	-2.15 (4.4)	236 (23%)	1022 (50%)	NR	NR
Rockwood 2001	27	0 (0%)	23.2 (5.2)	NR	NR	NR	261 (68%)	NR	NR
	5	0 (0%)	22.9 (5.0)	NR	NR	NR	125 (32%)	NR	NR
Cummings 2004	23	0 (0%)	20.7 (4.9)	NR	NR	NR	692 (71%)	NR	NR
	81	0 (0%)	20.6 (4.9)	NR	NR	NR	286 (29%)	NR	NR
Burns 2009	62	0 (0%)	NR	9.2 (4.5)†	NR	NR	211 (51%)	NR	NR
	75	0 (0%)	NR	9.6 (4.9)†	NR	NR	204 (49%)	NR	NR
Gault 2015	5	0 (0%)	19.2 (4.1)	20.7 (5.1)	1.5 (2.6)	48 (71%)	68 (50%)	NR	305 days (range [224, 377])
	3	0 (0%)	18.8 (4)	18.9 (4.8)	0.1 (2.4)	45 (66%)	68 (50%)	NR	239 days (range [206, 295])
Haig 2014	2	0 (0%)	17.9 (4.2)	19.7 (3.9)	1.2 (2.8)	41 (68%)	60 (49%)	NR	286 days (range N/A – a single date was provided)
	1	0 (0%)	17.8 (3.8)	19.9 (4.2)	1.8 (1.8)	47 (75%)	63 (51%)	NR	270 days (range [161, 379]).
Bakchine 2008	33	0 (0%)	18.7 (3.3)	NR	NR	NR	318 (68%)	NR	NR
	9	0 (0%)	18.9 (3.2)	NR	NR	NR	152 (32%)	NR	NR
Herrman 2013	18	0 (0%)	11.9 (3.1)	11.3 (4.9)	-0.76 (3.4)	31 (8%)	182 (49%)	NR	NR
	11	0 (0%)	11.8 (2.9)	11.1 (4.7)	-0.68 (3.2)	32 (9%)	187 (51%)	NR	NR

For peer review only

For peer review only - http://bmjopen.bmj.com/site/about/guidelines.xhtml

Wilkinson 2012	17	0 (0%)	16.7 (2.5)	16.4 (5.2)	-0.46 (3.9)	30 (11%)	133 (48%)	NR	NR
	20	0 (0%)	17.1 (2.4)	16.4 (5.6)	-0.69 (4.0)	30 (11%)	144 (52%)	NR	NR

* According to publication
† The MMSE final value comes from visit 8 (last available visit in IPD). MMSE was not reported in study publication
Abbreviations: AD, Alzheimer's Dementia; IPD, individual patient data; MMSE, Mini-Mental State Examination; NR, not reported; N/A, not applicable; AE, adverse event

For peer review only

Appendix 7: Comparison of studies with shared IPD with (a) all remaining studies and (b) studies for which sponsors claimed unavailable IPD. AD: aggregate data; IPD: individual patient data

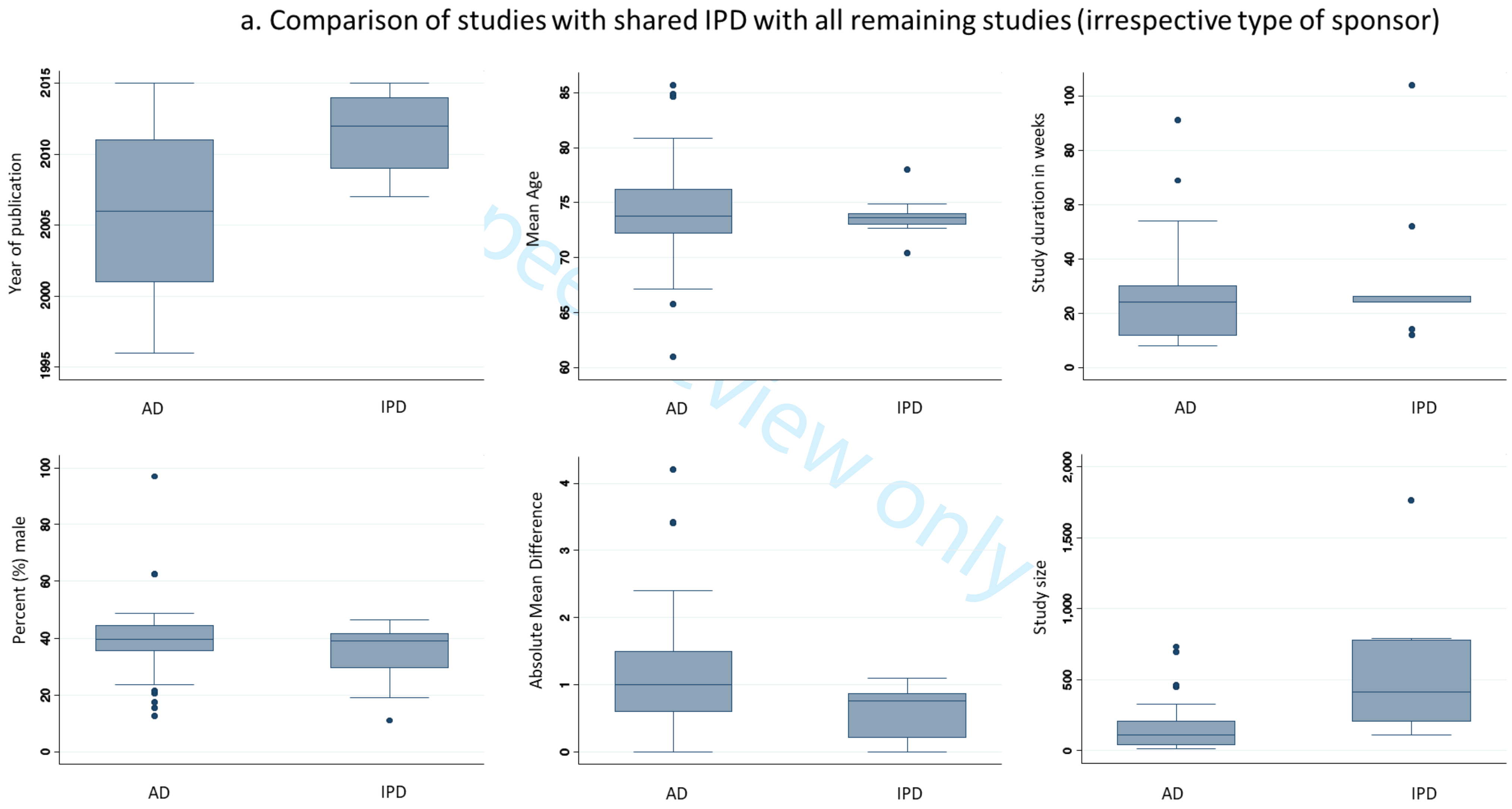

For peer review only - http://bmjopen.bmj.com/site/about/guidelines.xhtml

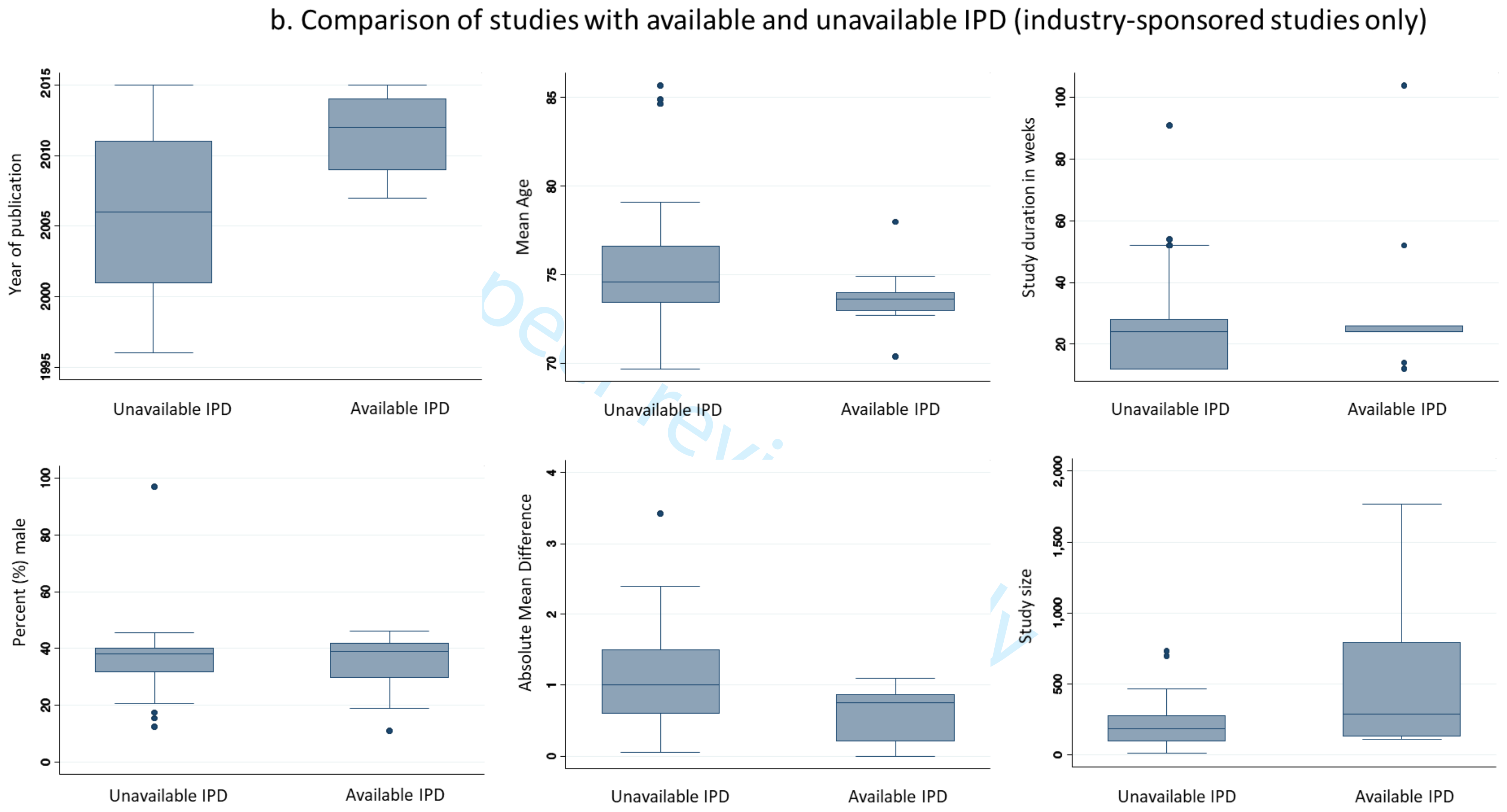
b. Comparison of studies with available and unavailable IPD (industry-sponsored studies only)
Year of publication
1995
2000
2005
2010
2015
Unavailable IPD
Available IPD
Mean Age
70
75
80
85
Unavailable IPD
Available IPD
Study duration in weeks
20
40
60
80
100
Unavailable IPD
Available IPD
Percent (%) male
0
20
40
60
80
100
Unavailable IPD
Available IPD
Absolute Mean Difference
0
1
2
3
4
Unavailable IPD
Available IPD
Study size
0
500
1,000
1,500
2,000
Unavailable IPD
Available IPD

Appendix 8: Cochrane Risk-of-bias appraisal results (n = 80)

Study	1. Random sequence generation	2. Allocation concealment	3. Blinding of participants and personnel	4. Blinding of outcome assessment	5. Incomplete outcome data	6. Selective reporting	7. Other bias*
Agid, 1998	Low	High	Low	Unclear	High	Unclear	High
Ancoli-Israel, 2005	Unclear	Unclear	Unclear	Unclear	High	Unclear	High
Andersen, 2012	Unclear	Low	Low	Low	High	Low	Low
Araki, 2014	Low	Unclear	Unclear	Unclear	High	Unclear	Unclear
Bakchine, 2008	Low	Low	Low	Low	Low	High	High
Black, 2007	Low	Low	Low	Low	Low	Unclear	High
Blesa Gonzalez, 2011	Unclear	Unclear	High	Unclear	High	Low	High
Burns, 1999	Unclear	Unclear	Unclear	Unclear	High	Unclear	High
Burns, 2009	Low	Low	Low	Low	Low	Unclear	High
Burns, 2011	Low	Unclear	Low	Low	High	Unclear	Unclear
Choi, 2011	Unclear	Unclear	High	High	High	Low	Low
Corey-Bloom, 1998	Low	Low	Low	Low	High	Unclear	High
Cretu, 2008	Unclear	Unclear	Unclear	Low	Unclear	Unclear	Unclear
Dysken, 2014	Low	Low	Low	Unclear	Low	Low	Low
Farlow, 2013	Low	Unclear	Low	Low	High	Unclear	High
Feldman, 2001	Low	Unclear	Low	Low	High	Unclear	High
Feldman, 2007	Low	Low	Low	Low	High	Unclear	High
Fox, 2012	Low	Low	High	Low	High	High	Unclear
Frolich, 2011	Unclear	Unclear	Low	Low	High	Low	High
Fuschillo, 2001	Unclear	Unclear	Unclear	Unclear	Low	Low	Unclear
Gault, 2015	Low	Low	Low	Unclear	Low	Low	High
Gold, 2010	Low	Unclear	Low	Low	High	Low	High
Greenberg, 2000	Low	Low	Low	Unclear	High	Low	Low
Grossberg, 2013	Low	Low	Low	Low	High	Low	High
Hager K, 2014	Low	Low	Low	Low	High	High	High
Haig, 2014	Low	Low	Low	Low	High	Low	High
Hernández, 2007	Low	Low	Low	Low	Unclear	Low	Low
Herrmann, 2013	Low	Low	Low	Low	High	Low	High
Holmes, 2004	Low	Unclear	Low	Low	High	Low	High
Homma, 1998	Low	Low	Low	Low	Low	Unclear	High
Homma, 2008	Low	Low	Low	Low	High	Unclear	Unclear
Hong, 2006	Unclear	Unclear	Unclear	Unclear	Low	Unclear	Unclear
Howard, 2007	Low	Low	Low	Low	Low	Unclear	Low
Howard, 2012	Low	Low	Low	Low	High	Low	Low
Hu, 2006	Unclear	Unclear	Unclear	Unclear	Low	Unclear	Unclear
Johannsen, 2006	Unclear	Unclear	Low	Low	Low	Unclear	High
Jones, 2004	Low	Unclear	Unclear	Low	Low	Unclear	High
Kadir, 2008	Unclear	Unclear	Unclear	Unclear	High	Unclear	High
Kano, 2013	Unclear	Unclear	Unclear	Unclear	Low	Unclear	Unclear
Karaman, 2005	Low	Unclear	Low	Low	Unclear	Unclear	Unclear
Likitjaroen, 2012	Low	Low	Low	Unclear	High	High	Unclear
Lorenzi, 2011	Unclear	Unclear	Unclear	Unclear	Unclear	Unclear	High
Maher-Edwards, 2011	Low	Unclear	Unclear	Unclear	High	Unclear	High
Marek, 2014	Low	Low	Low	Low	High	Low	High
Mazza, 2006	Low	Unclear	Low	Low	High	Unclear	Unclear
Mohs, 2001	Low	Low	Low	Low	High	Unclear	High
Moretti, 2014	Unclear	Unclear	Unclear	Unclear	Low	Unclear	Low
Mowla, 2007	Low	Unclear	Low	Unclear	High	Unclear	Unclear
Nakamura, 2011	Unclear	Low	Low	Low	Low	Low	High
Nakano, 2001	Unclear	Unclear	Unclear	Unclear	Unclear	Unclear	Unclear
Nordberg, 2009	Unclear	Unclear	High	High	Unclear	Unclear	High
Pakdaman H, 2015	Low	Unclear	High	High	High	Unclear	Unclear
Peng, 2005	Unclear	Unclear	Unclear	Unclear	Low	Unclear	Unclear
Peskind, 2006	Low	Low	Low	Unclear	Low	Unclear	High
Peters, 2015	Unclear	Unclear	Low	Low	High	Low	Low
Reisberg, 2003	Low	Unclear	Low	Unclear	High	Low	Unclear
Rockwood, 2001	Low	Low	Low	Low	Unclear	Low	High
Rockwood, 2006	Low	Low	Low	Low	Low	Unclear	Unclear
Rogers, 1996	Unclear	Unclear	Low	Unclear	Low	Unclear	Unclear
Rogers, 1998	Unclear	Unclear	Low	Low	Low	Unclear	High
Rogers, 1998	Low	Unclear	Low	Unclear	High	Unclear	High
Saxton, 2012	Low	Low	Low	Low	Low	Low	High
Scarpini, 2011	Low	Low	Low	Unclear	High	Unclear	High
Schmidt, 2008	Low	Low	Low	Low	High	Unclear	High
Seltzer, 2004	Low	Unclear	Unclear	Unclear	Unclear	Unclear	High

For peer review only - http://bmjopen.bmj.com/site/about/guidelines.xhtml

Shao, 2015	Unclear	Unclear	Unclear	Unclear	Low	Unclear	Unclear
Shimizu, 2015	Low	Unclear	High	Low	High	Unclear	Unclear
Sole-Padulles, 2013	Unclear	Unclear	Unclear	Unclear	Low	Unclear	Unclear
Tariot, 2000	Low	Unclear	Low	Low	High	Low	High
Tariot, 2001	Low	Low	Low	Low	Unclear	Unclear	High
Thomas, 2001	Low	Unclear	Unclear	Unclear	Unclear	Unclear	Unclear
Wilcock, 2003	Unclear	Unclear	Unclear	Unclear	Unclear	Unclear	Unclear
Wilkinson, 2001	Low	Low	Low	Low	High	Unclear	High
Wilkinson, 2002	Low	Low	Low	Low	High	Unclear	High
Wilkinson, 2012	Low	High	Low	Low	High	Low	High
Winblad, 2001	Low	Unclear	Unclear	Low	High	Unclear	High
Winblad, 2006	Low	Low	Low	Low	High	Low	High
Winblad, 2007	Low	Low	Low	Low	High	Unclear	High
Yi, 2005	Unclear	Unclear	Unclear	Unclear	Low	Unclear	Unclear
Zhang, 2012	Unclear	Unclear	Unclear	Unclear	High	Unclear	High

* Other bias was categorized as:
a) *low risk of bias* when the study appeared to be free of other sources of bias,
b) *high risk of bias* when there was at least one important risk of bias. For example, when the study had:

- A potential source of bias related to the specific study design used; or
- A conflict of interest related to funding source; or
- An author was an employee of the drug company that sponsored the study; or
- Been claimed to have been fraudulent; or
- Other potential biases.

c) *unclear risk of bias* when there was a potential for bias, but there was either:

- Insufficient information to assess whether an important risk of bias exists; or
- Insufficient rationale/evidence that an identified problem would introduce bias; or
- Funding by drug company, but conflicts were not described

For peer review only - http://bmjopen.bmj.com/site/about/guidelines.xhtml

Appendix 9: Overall risk of bias for studies with shared IPD against (a) all remaining studies and (b) studies for which sponsors claimed unavailable IPD. AD: aggregate data; IPD: individual patient data

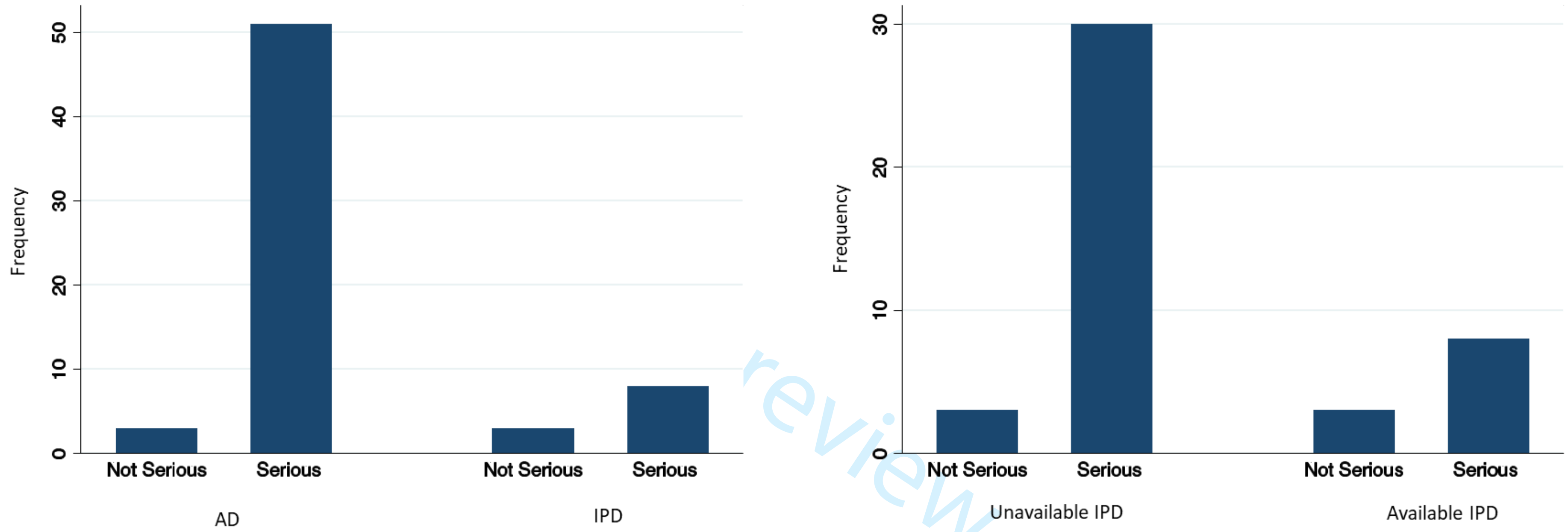

a. Comparison of studies with shared IPD with all remaining studies (irrespective type of sponsor)

b. Comparison of studies with available and unavailable IPD (industry-sponsored studies only)

review only

For peer review only - http://bmjopen.bmj.com/site/about/guidelines.xhtml

Appendix 10: Study-specific effect sizes calculated from shared IPD and published data. IPD: individual patient data

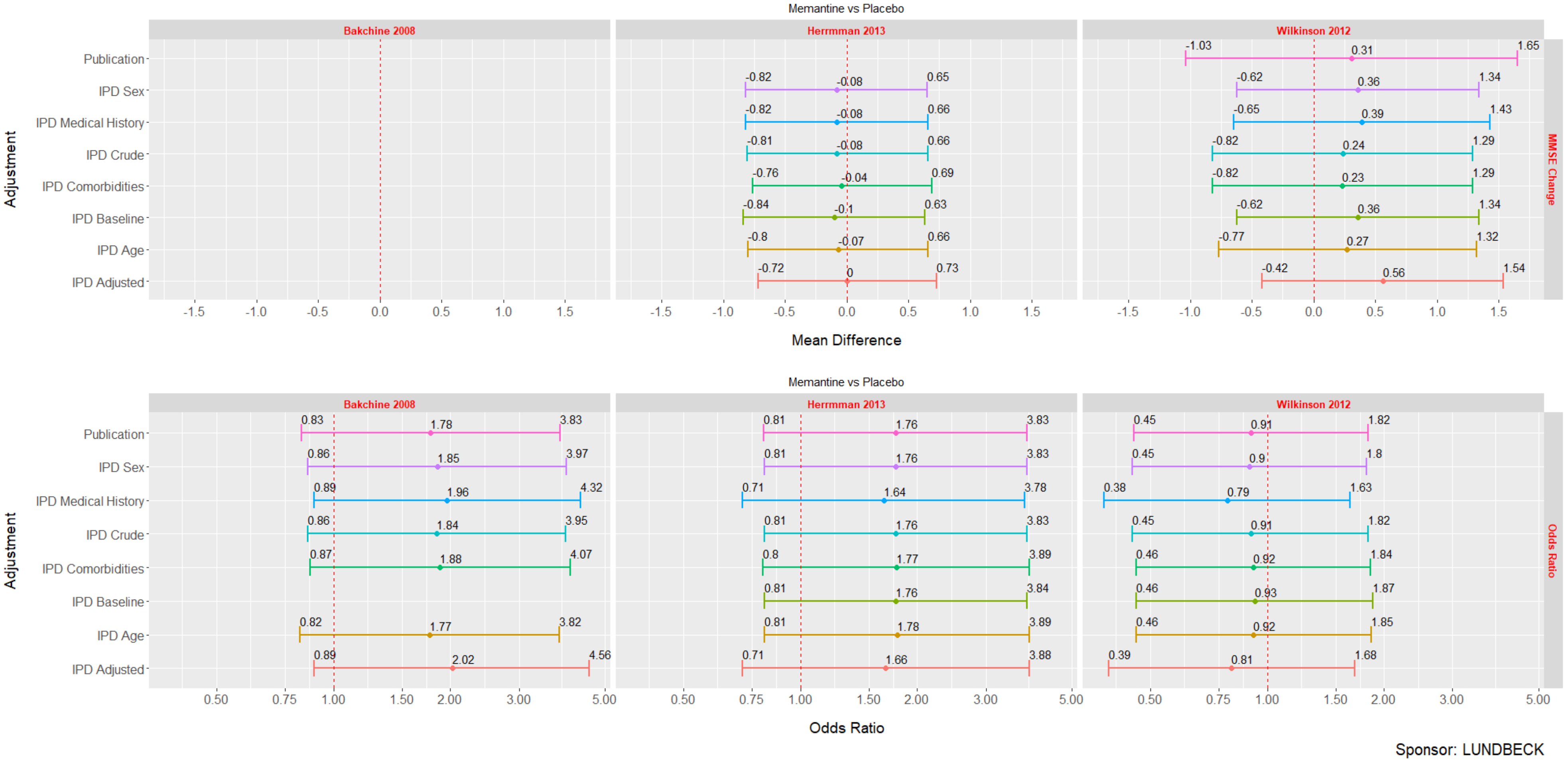

32

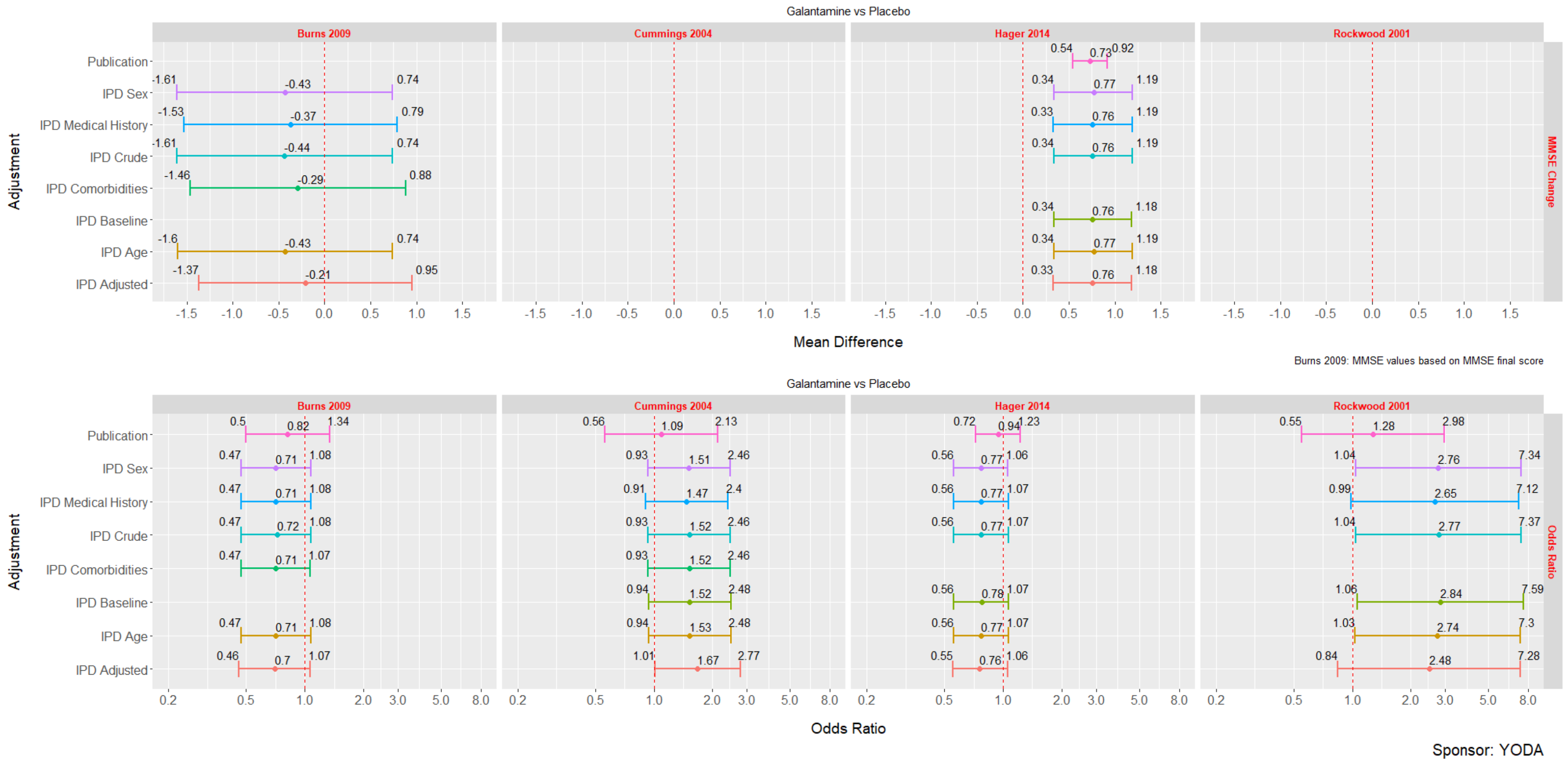
Galantamine vs Placebo
Burns 2009
Cummings 2004
Hager 2014
Rockwood 2001
Adjustment
Publication
IPD Sex
IPD Medical History
IPD Crude
IPD Comorbidities
IPD Baseline
IPD Age
IPD Adjusted
MMSE Change
Mean Difference
Burns 2009: MMSE values based on MMSE final score
Galantamine vs Placebo
Burns 2009
Cummings 2004
Hager 2014
Rockwood 2001
Adjustment
Publication
IPD Sex
IPD Medical History
IPD Crude
IPD Comorbidities
IPD Baseline
IPD Age
IPD Adjusted
Odds Ratio
Odds Ratio
Sponsor: YODA

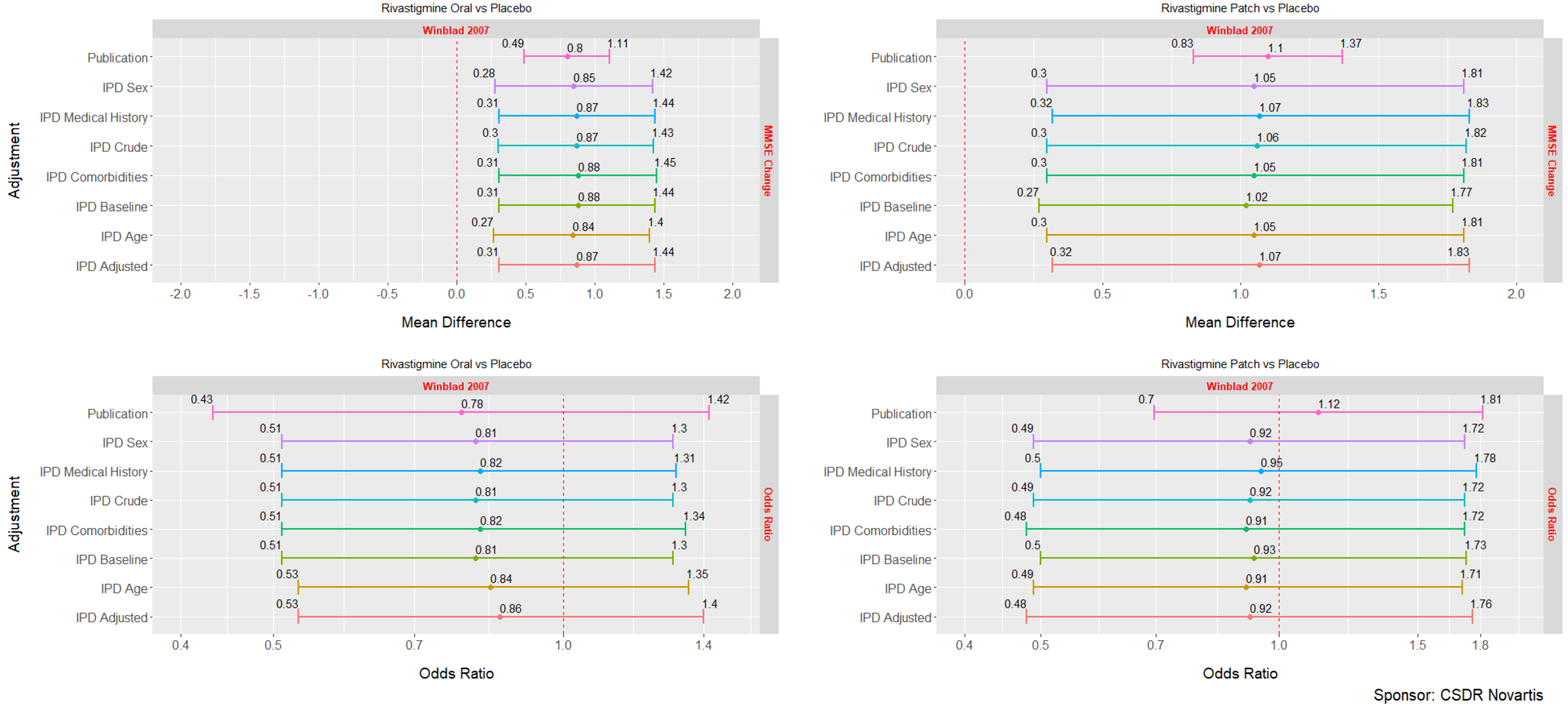
Rivastigmine Oral vs Placebo
Winblad 2007
MMSE Change
Publication 0.49 0.8 1.11
IPD Sex 0.28 0.85 1.42
IPD Medical History 0.31 0.87 1.44
IPD Crude 0.3 0.87 1.43
IPD Comorbidities 0.31 0.88 1.45
IPD Baseline 0.31 0.88 1.44
IPD Age 0.27 0.84 1.4
IPD Adjusted 0.31 0.87 1.44
Adjustment
Mean Difference
Rivastigmine Patch vs Placebo
Winblad 2007
MMSE Change
Publication 0.83 1.1 1.37
IPD Sex 0.3 1.05 1.81
IPD Medical History 0.32 1.07 1.83
IPD Crude 0.3 1.06 1.82
IPD Comorbidities 0.3 1.05 1.81
IPD Baseline 0.27 1.02 1.77
IPD Age 0.3 1.05 1.81
IPD Adjusted 0.32 1.07 1.83
Mean Difference
Rivastigmine Oral vs Placebo
Winblad 2007
Odds Ratio
Publication 0.43 0.78 1.42
IPD Sex 0.51 0.81 1.3
IPD Medical History 0.51 0.82 1.31
IPD Crude 0.51 0.81 1.3
IPD Comorbidities 0.51 0.82 1.34
IPD Baseline 0.51 0.81 1.3
IPD Age 0.53 0.84 1.35
IPD Adjusted 0.53 0.86 1.4
Adjustment
Odds Ratio
Rivastigmine Patch vs Placebo
Winblad 2007
Odds Ratio
Publication 0.7 1.12 1.81
IPD Sex 0.49 0.92 1.72
IPD Medical History 0.5 0.95 1.78
IPD Crude 0.49 0.92 1.72
IPD Comorbidities 0.48 0.91 1.72
IPD Baseline 0.5 0.93 1.73
IPD Age 0.49 0.91 1.71
IPD Adjusted 0.48 0.92 1.76
Odds Ratio
Sponsor: CSDR Novartis

34

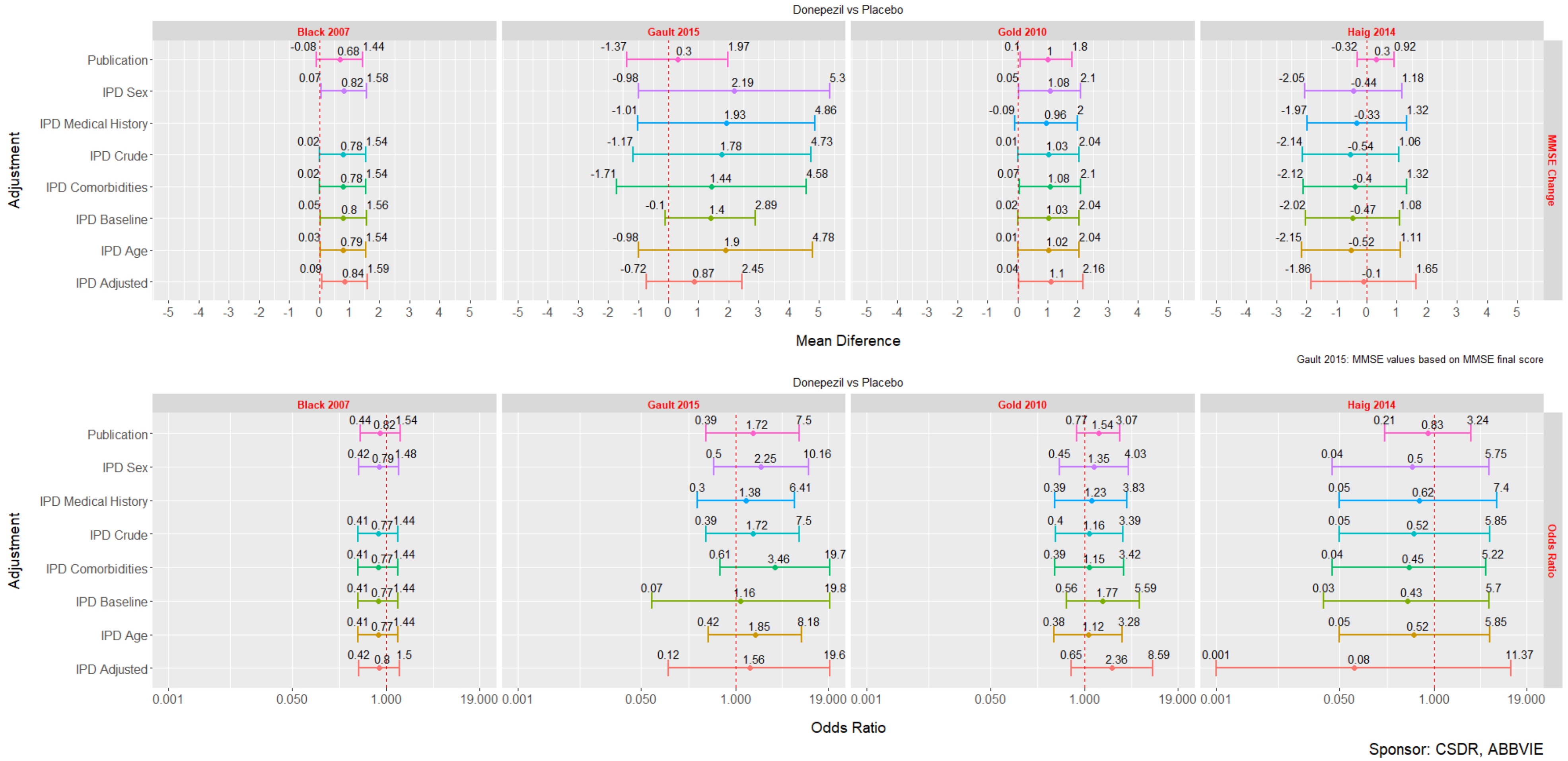

CSDR includes studies sponsored by GlaxoSmithKline, Eisai, Novartis, whereas YODA includes studies sponsored by Janssen

We also calculated the odds ratio for patients experiencing at least one AE excluding missing participants as shown in the MMSE outcome: Gold 2010: OR 2.78, 95% CI: 0.63-12.25; Black 2007: OR 1.19, 95% CI: 0.08-17.96; Winbland 2007: rivastigmine oral, OR 1.28, 95% CI: 0.09-18.16, rivastigmine patch, OR 0.81, 95% CI: 0.02-33.59; Wilkinson 2012: OR 0.84, 95% CI: 0.38-1.86; Herrmman 2013: OR 1.70, 95% CI: 0.71-4.08; Bachine 2008: OR 1.83, 95% CI: 0.77-4.32.

We were unable to assess this for studies obtained through YODA and AbbVie, since at the time of this assessement we did not have access to these data.

Abbreviations: IPD sex, regression analysis adjusting for sex; IPD medical history, regression analysis adjusting for medical history; IPD crude, analysis with no adjustments; IPD comorbidities, regression analysis adjusting for comorbidities; IPD baseline, regression analysis adjusting for MMSE baseline; IPD age, regression analysis adjusting for age; IPD adjusted, regression analysis adjusting for all available variables (we only considered those that we initially requested from sponsor)

For peer review only

For peer review only - http://bmjopen.bmj.com/site/about/guidelines.xhtml

Appendix 11: Correlation between participant age and dropout in studies with IPD. IPD: individual patient data

	Study*	Correlation	P-Value
**CSDR**	Black 2007 (EISAI)	0.079	0.147
	Gold 2010 (GSK)	0.141	0.072
	Winblad 2007 (Novartis)	0.016	0.584
**Lundbeck**	Wilkinson 2012	0.066	0.273
	Herrmman 2013	0.124	0.017

* We were unable to assess this correlation for studies obtained through YODA and AbbVie, since at the time of this assessment we did not have access to these data

For peer review only

For peer review only - http://bmjopen.bmj.com/site/about/guidelines.xhtml

Appendix 12: Comparison Adjusted Funnel plot (all treatments vs placebo)

(a) MMSE

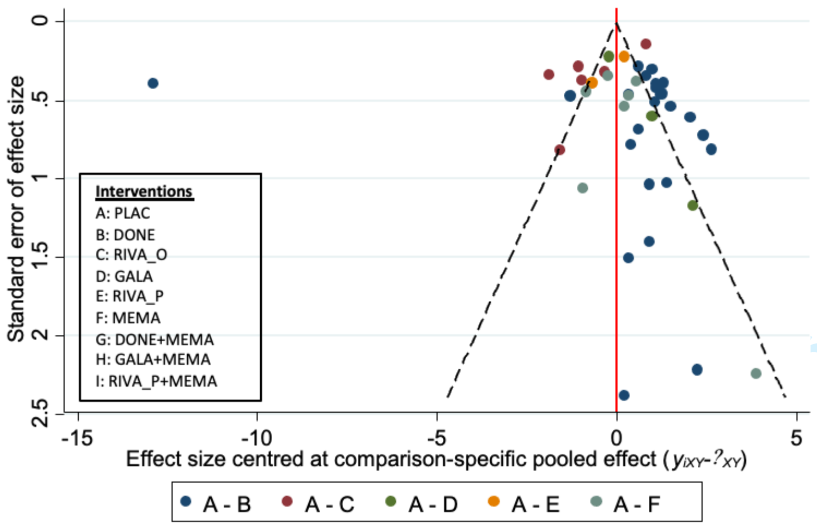

Note: Comparisons including only one study (when present) have been excluded

(b) Adverse Events

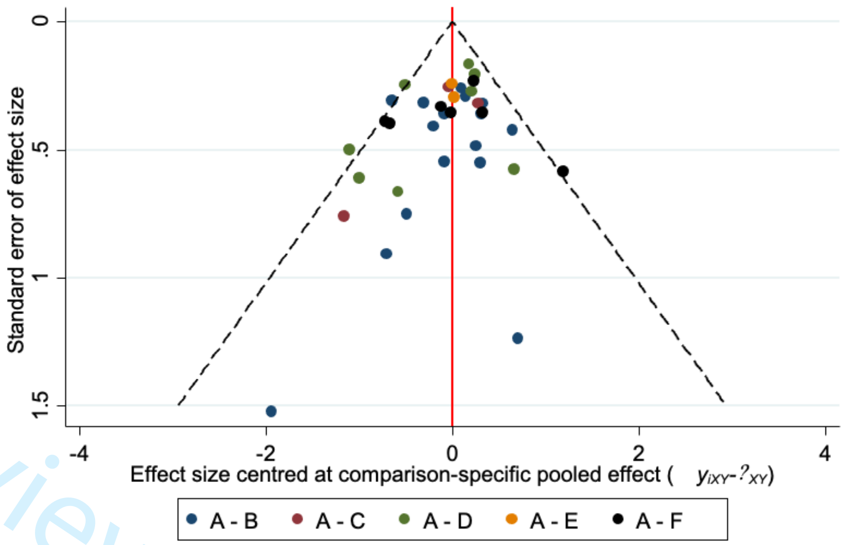

Note: Comparisons including only one study (when present) have been excluded

38

Appendix 13: Distribution of potential effect modifiers per treatment comparison and outcome

(a) MMSE

(b) Adverse Events

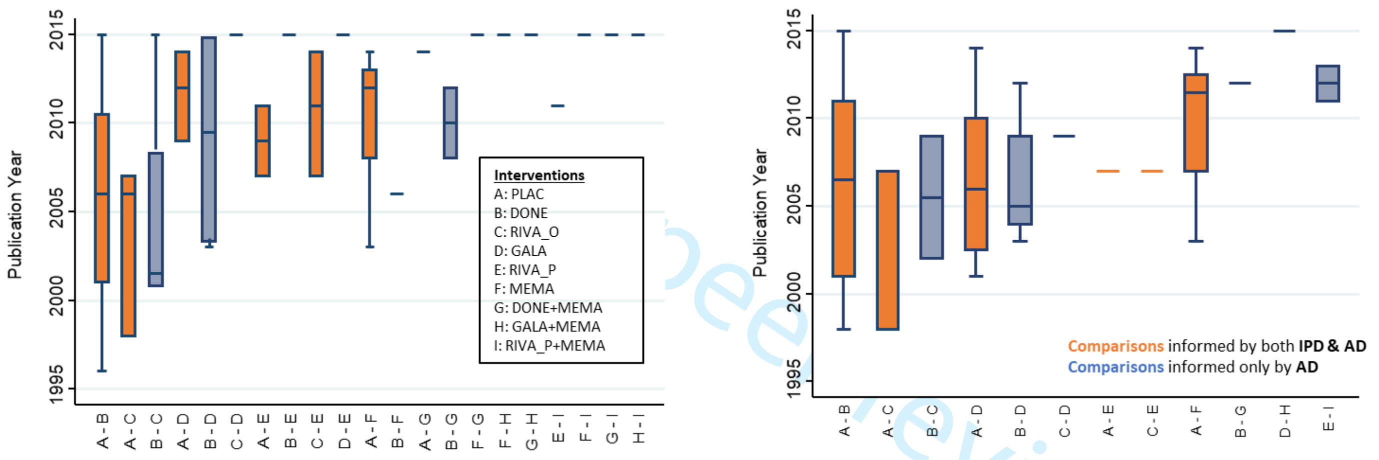

a. Publication year

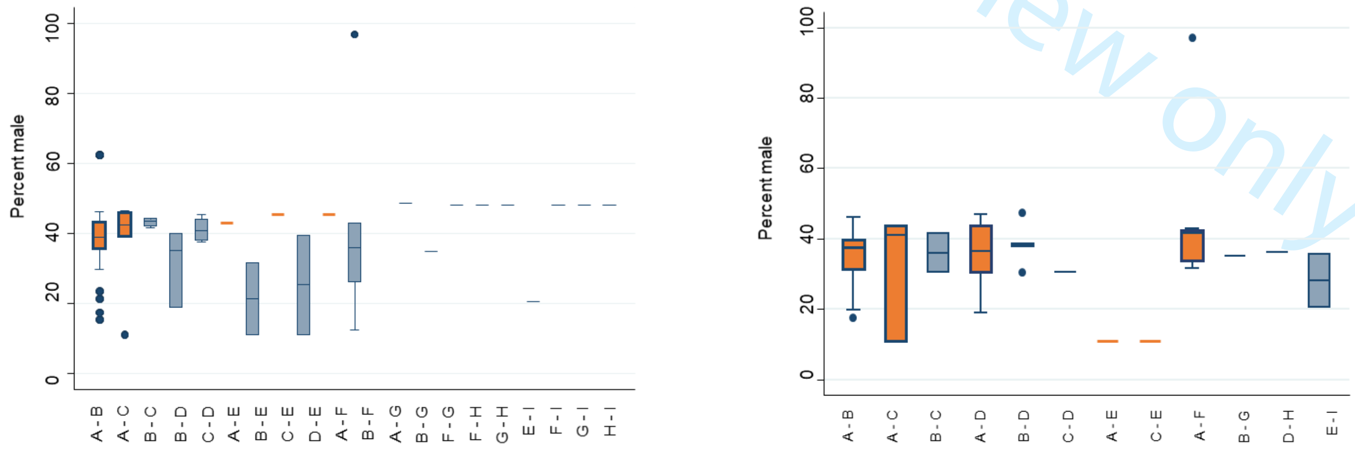

b. Percentage male

For peer review only - http://bmjopen.bmj.com/site/about/guidelines.xhtml

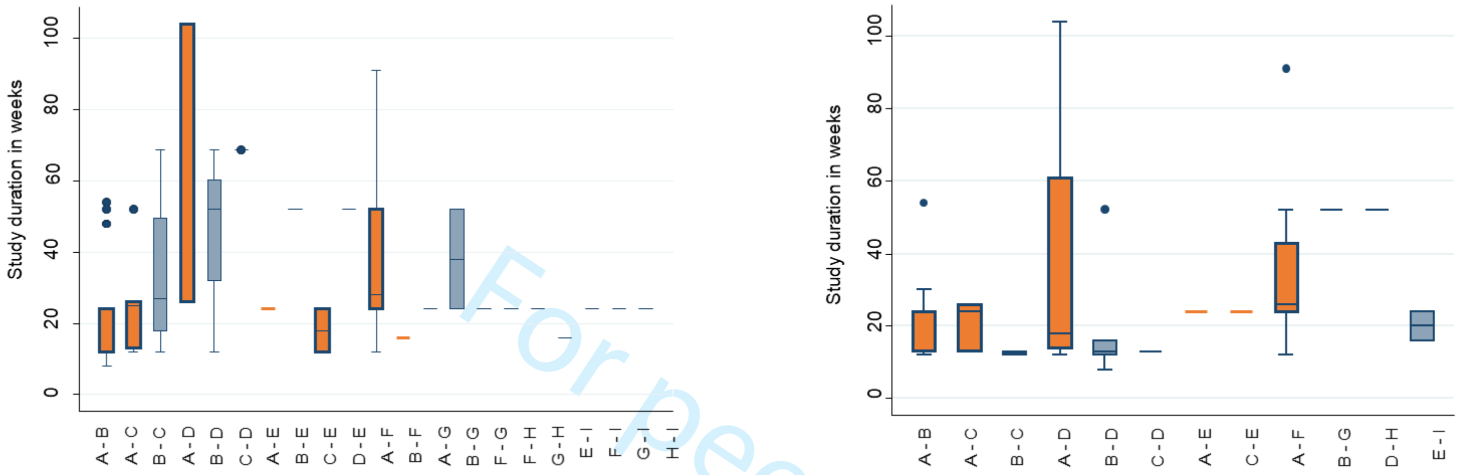

c. Study duration

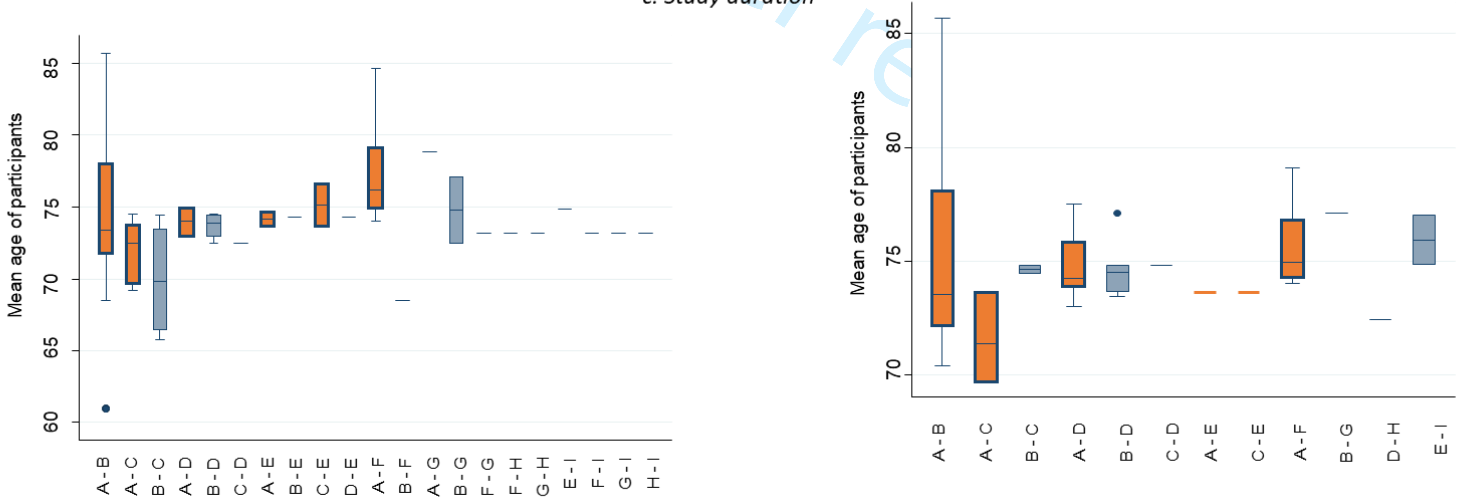

d. Mean participant age

For peer review

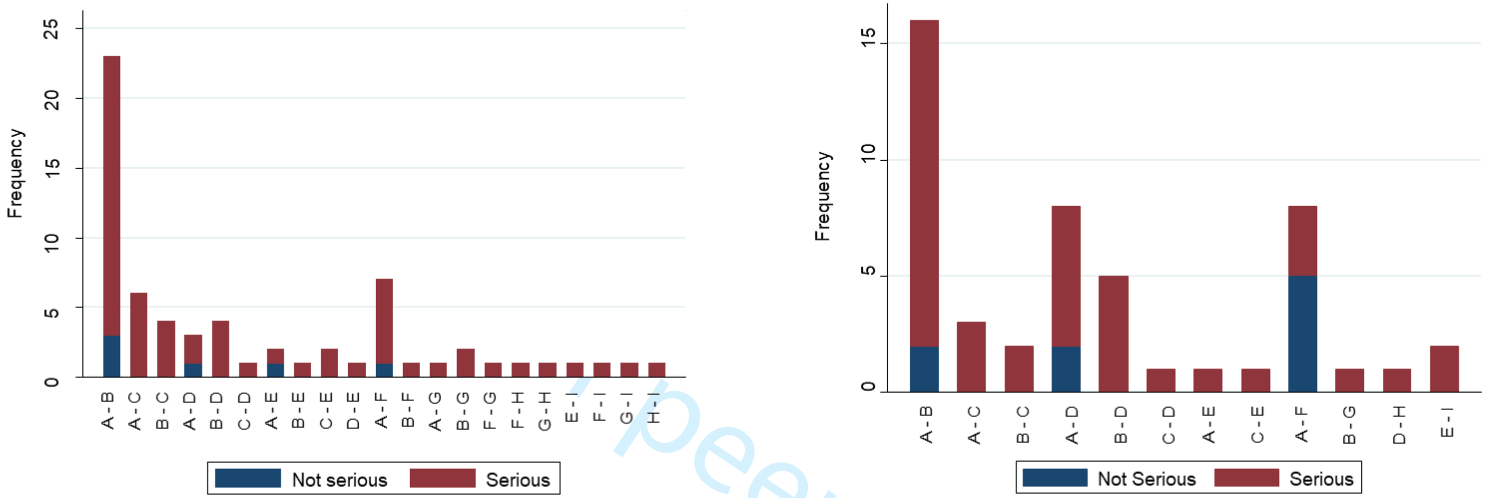

e. Overall Risk of Bias

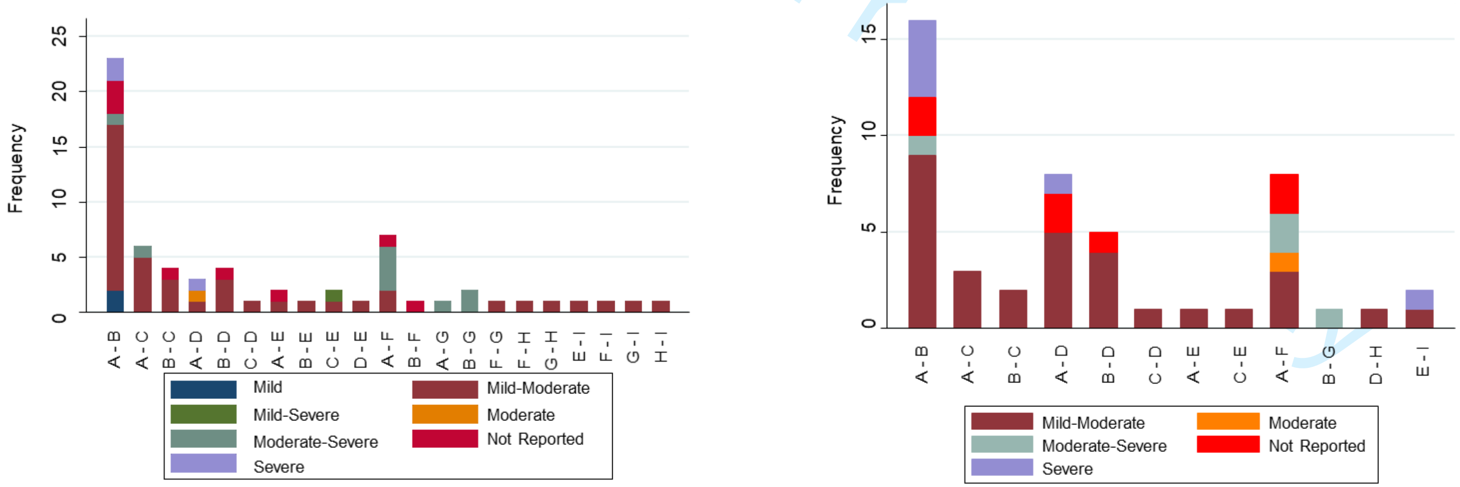

f. Alzheimer's Dementia Severity

Appendix 14: Consistency Assessment – Loop-specific approach (using adjusted treatment effects)

(a) MMSE

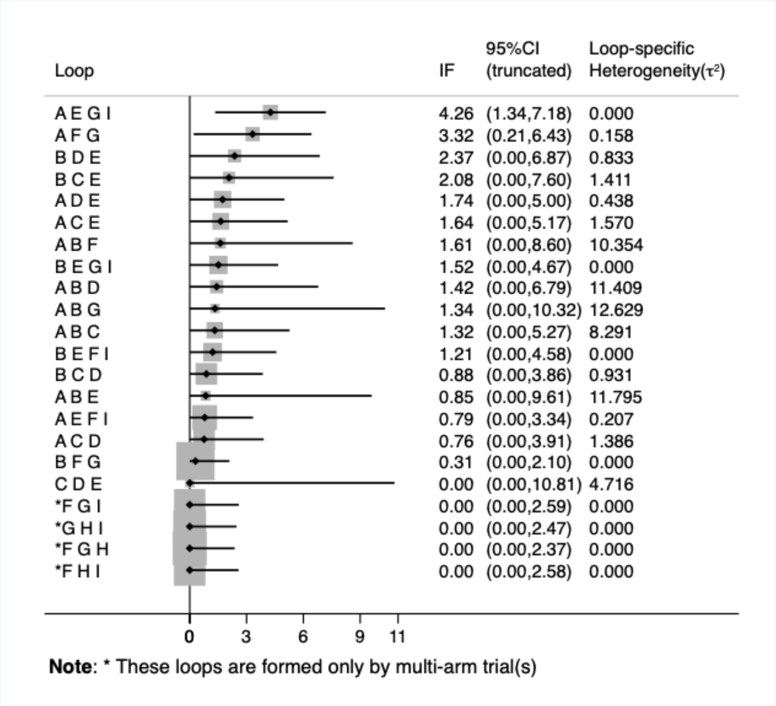

Design-by-treatment interaction model:
χ^2 statistic: 4.36, 13 degrees of freedom, P value: 0.987, between-study variance: 7.34. I^2 statistic=96%

(b) Adverse Events

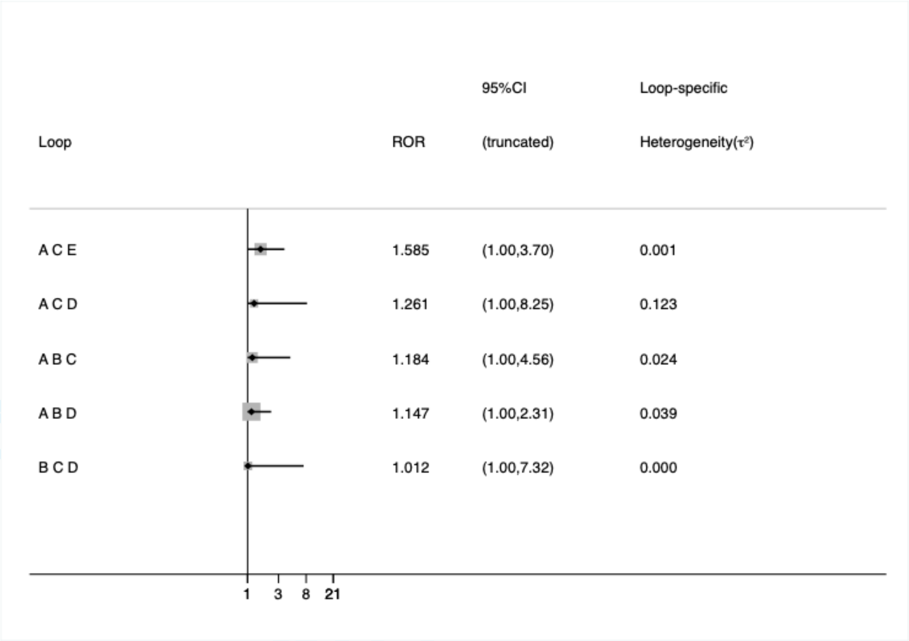

Design-by-treatment interaction model:
χ^2 statistic: 3.57, 6 degrees of freedom, P value: 0.735, between-study variance: 0.06. I^2 statistic=22%

Appendix 15: Network and standard meta-analysis results

Treatment Comparison	NMA estimate	95% CI	95% PI	P-score	MA estimate	95% CI	95% PI	#studies
**Mini-Mental State Examination (MMSE)*†**								
Donepezil vs Placebo	1.41	0.51 to 2.32	-3.48 to 6.31	0.59	1.65	0.16 to 3.14	-6.02 to 9.32	24
Rivastigmine oral vs Placebo	0.69	-0.79 to 2.18	-4.35 to 5.74	0.36	0.60	-0.43 to 1.62	-3.07 to 4.26	6
Galantamine vs Placebo	0.41	-1.44 to 2.26	-4.76 to 5.58	0.28	0.04	-1.09 to 1.17	-12.39 to 12.47	3
Rivastigmine transdermal vs Placebo	2.11	-0.04 to 4.26	-3.18 to 7.40	0.72	0.56	-0.33 to 1.45	--	2
Memantine vs Placebo	0.67	-0.99 to 2.34	-4.43 to 5.78	0.35	0.52	0.03 to 1.01	-0.69 to 1.73	7
Donepezil + Memantine vs Placebo	2.57	0.07 to 5.07	-2.88 to 8.02	0.80	4.21	1.94 to 6.48	--	1
Galantamine + Memantine vs Placebo	2.24	-2.13 to 6.61	-4.33 to 8.81	0.66				
Rivastigmine transdermal + Memantine vs Placebo	1.79	-1.70 to 5.27	-4.20 to 7.78	0.60				
Placebo (reference)				0.14				
Rivastigmine transdermal vs Rivastigmine oral	1.41	-0.80 to 3.62	-3.90 to 6.73		2.26	-0.48 to 4.99	-30.56 to 35.07	3
Rivastigmine oral vs Donepezil	-0.72	-2.28 to 0.84	-5.79 to 4.35		0.16	-0.57 to 0.90	-1.45 to 1.77	4
Galantamine vs Rivastigmine oral	-0.29	-2.48 to 1.91	-5.60 to 5.02		0.06	-1.05 to 1.17		1
Rivastigmine transdermal vs Donepezil	0.69	-1.52 to 2.91	-4.62 to 6.01		-0.20	-2.78 to 2.38		1
Rivastigmine transdermal vs Galantamine	1.70	-0.93 to 4.33	-3.81 to 7.21		2.20	-0.19 to 4.59		1
Rivastigmine transdermal + Memantine vs Rivastigmine transdermal	-0.32	-3.82 to 3.18	-6.32 to 5.68		-0.40	-1.40 to 0.60		1
Memantine vs Donepezil	-0.74	-2.56 to 1.08	-5.90 to 4.42		0.20	0.88 to 1.28		1
Donepezil + Memantine vs Donepezil	1.15	-1.33 to 3.64	-4.29 to 6.59		0.88	0.64 to 1.11		2
Galantamine vs Donepezil	-1.01	-2.86 to 0.84	-6.18 to 4.16		-0.35	-1.52 to 0.83	-5.31 to 4.62	4
Donepezil + Memantine vs Memantine	1.89	-0.88 to 4.67	-3.69 to 7.48		0.37	-1.04 to 1.78		1
Galantamine + Memantine vs Memantine	1.57	-2.78 to 5.92	-4.98 to 8.12		0.82	-0.58 to 2.22		1

For peer review only

Rivastigmine transdermal + Memantine vs Memantine	1.12	-2.47 to 4.70	-4.93 to 7.16		0.41	-1.17 to 1.99		1
Galantamine + Memantine vs Donepezil + Memantine	-0.33	-4.72 to 4.06	-6.91 to 6.23		0.45	-0.85 to 1.75		1
Rivastigmine transdermal + Memantine vs Donepezil + Memantine	-0.78	-4.53 to 2.97	-6.93 to 5.38		0.04	-1.45 to 1.53		1
Rivastigmine transdermal + Memantine vs Galantamine + Memantine	-0.45	-5.05 to 4.14	-7.18 to 6.28		-0.41	-1.89 to 1.07		1
Common within-network between-study variance τ^2 = 5.75, I^2 = 96% (96%, 97%)								
Design-by-treatment interaction model for inconsistency χ^2 *(d.f., P-value,* τ^2*)*: 4.36 (13, 0.987, 7.35)								
**Adverse Events (AEs)*‡**								
Donepezil vs Placebo	1.08	0.87 to 1.35	0.67 to 1.75	0.30	1.07	0.88 to 1.31	0.84 to 1.37	16
Rivastigmine oral vs Placebo	1.26	0.82 to 1.94	0.69 to 2.33	0.16	1.26	0.75 to 2.12	0.01 to 161.35	3
Galantamine vs Placebo	0.95	0.74 to 1.22	0.58 to 1.55	0.53	1.02	0.71 to 1.46	0.38 to 2.77	8
Rivastigmine transdermal vs Placebo	0.90	0.58 to 1.42	0.48 to 1.69	0.57	0.86	0.53 to 1.40		1
Memantine vs Placebo	0.88	0.64 to 1.20	0.52 to 1.49	0.63	0.87	0.63 to 1.20	0.38 to 1.99	8
Donepezil + Memantine vs Placebo	0.77	0.34 to 1.73	0.30 to 1.96	0.69				
Galantamine + Memantine vs Placebo	1.03	0.45 to 2.39	0.39 to 2.70	0.43				
Rivastigmine transdermal + Memantine vs Placebo	0.72	0.32 to 1.59	0.28 to 1.81	0.75				
Placebo (reference)				0.44				
Rivastigmine oral Donepezil vs	1.17	0.73 to 1.87	0.61 to 2.22		2.08	0.21 to 20.73		2
Galantamine vs Donepezil	0.88	0.64 to 1.19	0.52 to 1.49		0.79	0.46 to 1.39	0.32 to 1.96	5
Donepezil + Memantine vs Donepezil	0.71	0.33 to 1.55	0.29 to 1.76		0.71	0.37 to 1.38		1
Rivastigmine transdermal vs Rivastigmine oral	0.72	0.42 to 1.23	0.36 to 1.44		0.94	0.52 to 1.68		1
Rivastigmine transdermal + Memantine vs Rivastigmine transdermal	0.79	0.41 to 1.54	0.36 to 1.77		0.79	0.45 to 1.39		2
Galantamine vs Rivastigmine oral	0.75	0.46 to 1.22	0.39 to 1.45		0.63	0.15 to 2.64		1

For peer review only - http://bmjopen.bmj.com/site/about/guidelines.xhtml

Galantamine + Memantine vs Galantamine	1.09	0.49 to 2.42	0.43 to 2.75	1.09	0.55 to 2.17	1
Common within-network between-study variance τ2 = 0.04, I^2 = 22% (0%, 48%)						
Design-by-treatment interaction model for inconsistency χ^2 *(d.f., P-value,* τ^2*)*: 3.57 (6, 0.735, 0.06)						

* Aggregate data and fully adjusted results from studies with available individual patient data were used in both meta-analysis and NMA. The mean difference effect size is presented for MMSE and the odds ratio for AE.
† MMSE: Studies with available IPD included only available participants –to assess the missing data impact on the second stage (IMDoM) a separate analysis was applied
‡ AE: Studies with available IPD included all randomized participants

For peer review only

Appendix 16: Network subgroup and meta-regression analysis results

Treatment Comparison	NMA estimate	95% CI	95%PI	P-score
**Mini-Mental State Examination (MMSE)†**				
**Mean Difference: Aggregate data and crude results from studies with available individual patient data**				
Donepezil vs Placebo	1.41	0.50 to 2.33	-3.51 to 6.34	0.59
Rivastigmine oral vs Placebo	0.69	-0.80 to 2.19	-4.38 to 5.76	0.36
Galantamine vs Placebo	0.37	-1.49 to 2.23	-4.82 to 5.57	0.28
Rivastigmine transdermal vs Placebo	2.10	-0.06 to 4.26	-3.22 to 7.42	0.72
Memantine vs Placebo	0.63	-1.05 to 2.30	-4.51 to 5.76	0.34
Donepezil + Memantine vs Placebo	2.56	0.04 to 5.07	-2.92 to 8.04	0.79
Galantamine + Memantine vs Placebo	2.22	-2.18 to 6.61	-4.39 to 8.82	0.66
Rivastigmine transdermal + Memantine vs Placebo	1.77	-1.73 to 5.27	-4.25 to 7.79	0.60
Placebo (reference)				0.14
Common within-network between-study variance τ^2 = 5.81, I^2 = 96% (96%, 97%)				
Design-by-treatment interaction model for inconsistency χ^2 *(d.f., P-value,* τ^2*)*: 4.42 (13, 0.986, 7.44)				
**Mean Difference: Aggregate data results**[**]				
Donepezil vs Placebo	1.55	0.41 to 2.68	-4.16 to 7.25	0.57
Rivastigmine oral vs Placebo	0.71	-1.10 to 2.52	-5.18 to 6.60	0.34
Galantamine vs Placebo	0.57	-1.98 to 3.12	-5.61 to 6.74	0.32
Rivastigmine transdermal vs Placebo	2.60	-0.20 to 5.40	-3.69 to 8.89	0.75
Memantine vs Placebo	0.82	-1.37 to 3.01	-5.21 to 6.84	0.37
Donepezil + Memantine vs Placebo	2.71	-0.17 to 5.60	-3.62 to 9.04	0.76
Galantamine + Memantine vs Placebo	2.44	-2.61 to 7.48	-5.19 to 10.07	0.65
Rivastigmine transdermal + Memantine vs Placebo	2.09	-1.98 to 6.15	-4.89 to 9.07	0.61
Placebo (reference)				0.15
Common within-network between-study variance τ^2 = 7.66, I2 = 97% (96%, 97%)				
Design-by-treatment interaction model for inconsistency χ^2 *(d.f., P-value,* τ^2*)*: 3.92 (11, 0.972, 8.76)				
**Mean Difference: Crude results from studies with available individual patient data**				
Donepezil vs Placebo	0.70	0.01 to 1.40	-0.67 to 2.07	0.65
Rivastigmine oral vs Placebo	0.87	-0.01 to 1.75	-0.70 to 2.44	0.73
Galantamine vs Placebo	0.45	-0.24 to 1.14	-0.91 to 1.82	0.48
Rivastigmine transdermal vs Placebo	1.06	0.04 to 2.08	-0.67 to 2.79	0.82
Memantine vs Placebo	0.05	-0.74 to 0.83	-1.42 to 1.51	0.20
Placebo (reference)				0.13
Common within-network between-study variance τ^2 = 0.12, I^2 = 29% (0%, 71%)				
Design-by-treatment interaction model for inconsistency χ^2 *(d.f., P-value,* τ^2*)*: N/A (no closed loops)				
**Mean Difference: Low Risk of Bias for Allocation Concealment***				
Donepezil vs Placebo	2.02	-0.24 to 4.28	-6.19 to 10.23	0.70
Rivastigmine oral vs Placebo	1.38	-2.27 to 5.02	-7.39 to 10.14	0.57
Galantamine vs Placebo	-0.31	-4.61 to 3.98	-9.42 to 8.79	0.31
Rivastigmine transdermal vs Placebo	0.82	-4.08 to 5.72	-8.63 to 10.27	0.48
Memantine vs Placebo	0.69	-3.01 to 4.39	-8.10 to 9.49	0.46
Donepezil + Memantine vs Placebo	2.88	-4.75 to 10.51	-8.48 to 14.23	0.69
Placebo (reference)				0.30
Common within-network between-study variance: τ^2 = 13.82, I^2 = 98% (98%, 99%)				
Design-by-treatment interaction model for inconsistency χ^2 *(d.f., P-value,* τ^2*)*: 0.13 (3, 0.99, 19.10)				
**Mean Difference: Low risk of bias for Incomplete Data***				
Donepezil vs Placebo	0.87	0.07 to 1.66	-1.67 to 3.40	0.61
Rivastigmine oral vs Placebo	-1.52	-4.41 to 1.37	-5.54 to 2.50	0.10
Galantamine vs Placebo	0.52	-0.94 to 1.99	-2.36 to 3.41	0.48
Rivastigmine transdermal vs Placebo	1.37	-0.64 to 3.38	-1.91 to 4.65	0.71
Memantine vs Placebo	0.57	-1.12 to 2.27	-2.47 to 3.62	0.48
Donepezil + Memantine vs Placebo	0.94	-2.11 to 4.00	-3.23 to 5.11	0.57
Galantamine + Memantine vs Placebo	1.39	-1.66 to 4.44	-2.77 to 5.56	0.70
Rivastigmine transdermal + Memantine vs Placebo	0.98	-2.15 to 4.12	-3.26 to 5.23	0.58
Placebo (reference)				0.27
Common within-network between-study variance: τ^2 = 1.16, I^2 = 79% (65%, 88%)				
Design-by-treatment interaction model for inconsistency χ^2 *(d.f., P-value,* τ^2*)*: 12.15 (3, 0.007, 0.863)				
**Mean Difference: Publicly-Sponsored Studies***				
Donepezil vs Placebo	6.57	-4.68 to 17.81	-129.61 to 142.74	0.71
Rivastigmine oral vs Placebo	1.40	-16.41 to 19.21	-161.58 to 164.38	0.44
Memantine vs Placebo	0.11	-17.65 to 17.87	-162.64 to 162.86	0.39
Rivastigmine transdermal + Memantine vs Placebo	5.83	-7.98 to 19.64	-139.93 to 151.59	0.65
Placebo (reference)				0.32

For peer review only - http://bmjopen.bmj.com/site/about/guidelines.xhtml

Common within-network between-study variance: τ^2 = 81.93, I^2 = 99% (99%, 100%)				
Design-by-treatment interaction model for inconsistency χ^2 (d.f., P-value, τ^2): 0.05 (1, 0.815, 116.71)				
**Mean Difference: Industry-Sponsored Studies***				
Donepezil vs Placebo	0.98	0.69 to 1.27	0.10 to 1.86	0.85
Rivastigmine oral vs Placebo	0.82	0.35 to 1.29	-0.14 to 1.78	0.69
Galantamine vs Placebo	0.41	-0.15 to 0.96	-0.60 to 1.41	0.34
Rivastigmine transdermal vs Placebo	0.80	0.18 to 1.41	-0.25 to 1.84	0.67
Memantine vs Placebo	0.60	0.06 to 1.15	-0.39 to 1.60	0.50
Rivastigmine transdermal + Memantine vs Placebo	0.40	-1.02 to 1.81	-1.29 to 2.08	0.39
Placebo (reference)				0.06
Common within-network between-study variance: τ^2 = 0.16, I^2 = 43% (15%, 62%)				
Design-by-treatment interaction model for inconsistency χ^2 (d.f., P-value, τ^2): 8.06 (7, 0.327, 0.16)				
**Mean Difference: Studies with Mild to Moderate cognitive impairment, assessed with MMSE at baseline ***				
Donepezil vs Placebo	1.68	0.31 to 3.06	-4.81 to 8.18	0.69
Rivastigmine oral vs Placebo	0.88	-1.29 to 3.05	-5.85 to 7.61	0.51
Galantamine vs Placebo	0.31	-2.47 to 3.09	-6.66 to 7.28	0.40
Rivastigmine transdermal vs Placebo	2.74	-0.68 to 6.16	-4.53 to 10.01	0.81
Memantine vs Placebo	-0.58	-4.84 to 3.69	-8.31 to 7.16	0.28
Donepezil + Memantine vs Placebo	0.43	-6.36 to 7.21	-9.06 to 9.91	0.45
Galantamine + Memantine vs Placebo	0.88	-5.90 to 7.66	-8.61 to 10.37	0.51
Rivastigmine transdermal + Memantine vs Placebo	1.11	-4.20 to 6.42	-7.30 to 9.52	0.55
Placebo (reference)				0.31
Common within-network between-study variance: τ^2 = 9.67, I^2 = 97% (97%, 98%)				
Design-by-treatment interaction model for inconsistency χ^2 (d.f., P-value, τ^2): 3.22 (9, 0.96, 13.28)				
**Mean Difference: Studies with Moderate to Severe cognitive impairment, assessed with MMSE at baseline ***				
Donepezil vs Placebo	1.31	0.66 to 1.96	-0.01 to 2.63	0.78
Rivastigmine oral vs Placebo	-1.00	-1.87 to -0.12	-2.51 to 0.51	0.04
Galantamine vs Placebo	-0.21	-1.64 to 1.21	-2.28 to 1.86	0.28
Memantine vs Placebo	0.69	0.07 to 1.31	-0.61 to 2.00	0.59
Donepezil + Memantine vs Placebo	2.49	1.55 to 3.44	0.92 to 4.07	1.00
Placebo (reference)				0.32
Common within-network between-study variance: τ^2 = 0.18, I^2 = 44% (0%, 75%)				
Design-by-treatment interaction model for inconsistency χ^2 (d.f., P-value, τ^2): 2.60 (1, 0.11, 0.11)				
**Mean Difference: Excluding outlier studies***§				
Donepezil vs Placebo	0.95	0.59 to 1.32	-0.64 to 2.54	0.57
Rivastigmine oral vs Placebo	0.65	0.09 to 1.22	-1.00 to 2.30	0.37
Galantamine vs Placebo	0.36	-0.38 to 1.09	-1.36 to 2.07	0.22
Rivastigmine transdermal vs Placebo	1.03	0.15 to 1.91	-0.76 to 2.82	0.59
Memantine vs Placebo	0.67	0.02 to 1.32	-1.01 to 2.35	0.39
Donepezil + Memantine vs Placebo	2.04	1.03 to 3.05	0.18 to 3.90	0.92
Galantamine + Memantine vs Placebo	1.87	0.08 to 3.66	-0.53 to 4.26	0.82
Rivastigmine transdermal + Memantine vs Placebo	1.10	-0.33 to 2.53	-1.03 to 3.23	0.58
Placebo (reference)				0.04
Common within-network between-study variance: τ^2 = 0.59, I^2 = 73% (64%, 79%)				
Design-by-treatment interaction model for inconsistency χ^2 (d.f., P-value, τ^2): 10.60 (13, 0.64, 0.61)				
**Accounting for missing outcome data - Informative Missingness Difference of Means**¶				
Donepezil vs Placebo	1.42	0.51 to 2.33	0.51 to 2.33	0.59‖
Rivastigmine oral vs Placebo	0.45	-1.09 to 1.99	-1.09 to 1.99	0.30‖
Galantamine vs Placebo	0.19	-1.78 to 2.17	-1.78 to 2.17	0.25‖
Rivastigmine transdermal vs Placebo	2.37	-0.03 to 4.79	-0.03 to 4.79	0.76‖
Memantine vs Placebo	0.60	-1.09 to 2.42	-1.09 to 2.42	0.36‖
Donepezil + Memantine vs Placebo	2.55	0.09 to 5.01	0.09 to 5.01	0.80‖
Galantamine + Memantine vs Placebo	2.26	-2.03 to 6.56	-2.03 to 6.56	0.68‖
Rivastigmine transdermal + Memantine vs Placebo	1.81	-1.66 to 5.28	-1.66 to 5.28	0.61‖
Placebo (reference)				0.16‖
Common within-network between-study variance: τ^2 = 5.47‖				
Design-by-treatment interaction model for inconsistency χ^2 (d.f., P-value, τ^2): 4.45 (11, 0.955, 6.45)				
**Mean Difference: Meta-regression, Trial Mean Age****				
Donepezil vs Placebo	1.53	0.52 to 2.53	-3.17 to 6.27	0.50 ††
Rivastigmine oral vs Placebo	0.80	-0.84 to 2.44	-4.15 to 5.79	0.37 ††
Galantamine vs Placebo	0.60	-1.63 to 2.83	-4.57 to 5.72	0.25 ††
Rivastigmine transdermal vs Placebo	2.53	0.06 to 4.98	-2.72 to 7.80	0.75 ††
Memantine vs Placebo	0.79	-1.18 to 2.74	-4.33 to 5.85	0.37 ††
Donepezil + Memantine vs Placebo	2.66	0.09 to 5.19	-2.70 to 7.97	0.87 ††
Galantamine + Memantine vs Placebo	2.39	-2.02 to 6.84	-4.14 to 8.83	0.75 ††
Rivastigmine transdermal + Memantine vs Placebo	2.05	-1.53 to 5.59	-3.83 to 7.94	0.75 ††
Placebo (reference)				0.12 ††
Regression coefficient	0.03	-0.14 to 0.20		
Common within-network between-study variance: τ^2 = 5.50	3.72 to 8.51			

For peer review only

Design-by-treatment interaction model for inconsistency χ² (d.f., P-value, τ²): 3.92 (11, 0.972, 8.76)				
**Mean Difference: NMA of studies with IPD adjusted for Age**				
Donepezil vs Placebo	0.72	0.03 to 1.42	-0.66 to 2.10	0.66
Rivastigmine oral vs Placebo	0.84	-0.05 to 1.73	-0.75 to 2.43	0.70
Galantamine vs Placebo	0.46	-0.24 to 1.15	-0.92 to 1.83	0.48
Rivastigmine transdermal vs Placebo	1.05	0.04 to 2.06	-0.68 to 2.78	0.83
Memantine vs Placebo	0.06	-0.72 to 0.84	-1.40 to 1.53	0.21
Placebo (reference)				0.12
Common within-network between-study variance: τ^2 = 0.12, I^2 = 29% (0%, 71%)				
Design-by-treatment interaction model for inconsistency χ² (d.f., P-value, τ²): N/A (one closed loop with a single multi-arm trial)				
**Mean Difference: Meta-regression, Percent of Male Participants**[**]				
Donepezil vs Placebo	1.62	0.58 to 2.65	-3.40 to 6.61	0.62 [††]
Rivastigmine oral vs Placebo	0.73	-0.90 to 2.35	-4.30 to 5.81	0.37 [††]
Galantamine vs Placebo	0.62	-1.65 to 2.89	-4.75 to 5.93	0.25 [††]
Rivastigmine Transdermal vs Placebo	2.51	0.01 to 5.04	-2.78 to 7.94	0.75 [††]
Memantine vs Placebo	0.66	-1.47 to 2.77	-4.54 to 5.88	0.25 [††]
Donepezil + Memantine vs Placebo	2.52	-0.40 to 5.45	-3.09 to 8.17	0.75 [††]
Galantamine + Memantine vs Placebo	2.27	-2.28 to 6.83	-4.37 to 8.90	0.75 [††]
Rivastigmine transdermal + Memantine vs Placebo	1.98	-1.67 to 5.65	-4.02 to 7.99	0.75 [††]
Placebo (reference)				0.12 [††]
Regression coefficient	0.01	-0.05 to 0.06		
Common within-network between-study variance: τ^2 = 5.73	3.83 to 8.84			
Design-by-treatment interaction model for inconsistency χ² (d.f., P-value, τ²): 3.72 (10, 0.959, 8.97)				
**Mean difference: NMA of studies with IPD adjusted for Percent of Male Participants**				
Donepezil vs Placebo	0.76	0.05 to 1.47	-0.67 to 2.19	0.67
Rivastigmine oral vs Placebo	0.85	-0.07 to 1.77	-0.80 to 2.50	0.69
Galantamine vs Placebo	0.45	-0.27 to 1.16	-0.99 to 1.88	0.46
Rivastigmine transdermal vs Placebo	1.05	0.01 to 2.09	-0.74 to 2.84	0.81
Memantine vs Placebo	0.10	-0.68 to 0.89	-1.40 to 1.61	0.23
Placebo (reference)				0.11
Common within-network between-study variance: τ^2 = 0.13, I^2 = 32% (0%, 72%)				
Design-by-treatment interaction model for inconsistency χ² (d.f., P-value, τ²): N/A (one closed loop with a single multi-arm trial)				
**Mean Difference: NMA of studies with IPD adjusted for cognitive impairment, assessed with MMSE at baseline**				
Donepezil vs Placebo	0.79	0.26 to 1.32	-0.06 to 1.64	0.64
Rivastigmine oral vs Placebo	0.88	0.31 to 1.45	-0.05 to 1.81	0.69
Galantamine vs Placebo	0.76	0.34 to 1.18	0.08 to 1.44	0.62
Rivastigmine transdermal vs Placebo	1.02	0.27 to 1.77	-0.20 to 2.24	0.82
Memantine vs Placebo	0.07	-0.52 to 0.66	-0.89 to 1.03	0.14
Placebo (reference)				0.08
Common within-network between-study variance: τ^2 = 0.00, I^2 = 0% (0%, 79%)				
Design-by-treatment interaction model for inconsistency χ² (d.f., P-value, τ²): N/A (one closed loop with a single multi-arm trial)				
**Mean Difference: NMA of studies with IPD adjusted for comorbidities**				
Donepezil vs Placebo	0.77	0.21 to 1.33	-0.15 to 1.68	0.71
Rivastigmine oral vs Placebo	0.88	0.31 to 1.45	-0.05 to 1.81	0.75
Galantamine vs Placebo	-0.29	-1.46 to 0.88	-2.19 to 1.61	0.15
Rivastigmine transdermal vs Placebo	1.05	0.30 to 1.80	-0.17 to 2.27	0.88
Memantine vs Placebo	0.05	-0.55 to 0.64	-0.92 to 1.01	0.27
Placebo (reference)				0.15
Common within-network between-study variance: τ^2 = 0.00, I^2 = 0% (0%, 67%)				
Design-by-treatment interaction model for inconsistency χ² (d.f., P-value, τ²): N/A (one closed loop with a single multi-arm trial)				
**Mean Difference: NMA of studies with IPD adjusted for other medications**				
Donepezil vs Placebo	0.67	-0.34 to 1.69	-1.44 to 2.79	0.61
Rivastigmine oral vs Placebo	0.87	-0.12 to 1.86	-1.21 to 2.95	0.71
Galantamine vs Placebo	0.42	-0.35 to 1.19	-1.40 to 2.25	0.47
Rivastigmine transdermal vs Placebo	1.07	-0.04 to 2.18	-1.16 to 3.30	0.81
Memantine vs Placebo	0.11	-0.74 to 0.96	-1.80 to 2.02	0.26
Placebo (reference)				0.14
Common within-network between-study variance: τ^2 = 0.17, I^2 = 35% (0%, 76%)				
Design-by-treatment interaction model for inconsistency χ² (d.f., P-value, τ²): N/A (one closed loop with a single multi-arm trial)				
**Mean Difference: Meta-regression, Study Duration**[**]				
Donepezil vs Placebo	1.66	0.67 to 2.66	-3.12 to 6.32	0.62 [††]
Rivastigmine oral vs Placebo	0.80	-0.77 to 2.37	-4.14 to 5.69	0.37 [††]
Galantamine vs Placebo	0.47	-1.75 to 2.68	-4.64 to 5.66	0.25 [††]
Rivastigmine transdermal vs Placebo	2.38	-0.04 to 4.83	-2.87 to 7.56	0.75 [††]
Memantine vs Placebo	0.67	-1.27 to 2.58	-4.35 to 5.79	0.25 [††]
Donepezil + Memantine vs Placebo	2.67	0.18 to 5.16	-2.60 to 7.97	0.88 [††]
Galantamine + Memantine vs Placebo	2.43	-1.94 to 6.79	-3.94 to 8.81	0.75 [††]
Rivastigmine transdermal + Memantine vs Placebo	2.13	-1.40 to 5.63	-3.62 to 7.87	0.75 [††]
Placebo (reference)				0.12 [††]

For peer review only - http://bmjopen.bmj.com/site/about/guidelines.xhtml

Regression coefficient	0.02	-0.01 to 0.06		
Common within-network between-study variance: τ^2 = 5.40	3.63 to 8.29			
Design-by-treatment interaction model for inconsistency χ^2 (d.f., P-value, τ^2): 4.36 (13, 0.987, 7.35)				
**Mean Difference: Meta-regression, Year of Publication**[**]				
Donepezil vs Placebo	1.53	0.51 to 2.54	-3.27 to 6.31	0.50[††]
Rivastigmine oral vs Placebo	0.66	-1.01 to 2.32	-4.31 to 5.65	0.25[††]
Galantamine vs Placebo	0.60	-1.65 to 2.85	-4.65 to 5.83	0.25[††]
Rivastigmine transdermal vs Placebo	2.59	0.09 to 5.12	-2.73 to 7.95	0.75[††]
Memantine vs Placebo	0.89	-1.05 to 2.80	-4.17 to 5.90	0.38[††]
Donepezil + Memantine vs Placebo	2.82	0.19 to 5.44	-2.57 to 8.21	0.88[††]
Galantamine + Memantine vs Placebo	2.59	-1.93 to 7.16	-3.98 to 9.12	0.75[††]
Rivastigmine transdermal + Memantine vs Placebo	2.21	-1.49 to 5.95	-3.81 to 8.24	0.75[††]
Placebo (reference)				0.12[††]
Regression coefficient	-0.02	-0.17 to 0.14		
Common within-network between-study variance: τ^2 = 5.53	3.71 to 8.48			
Design-by-treatment interaction model for inconsistency χ^2 (d.f., P-value, τ^2): 4.36 (13, 0.987, 7.35)				
**Adverse Events (AEs)‡**				
**Odds Ratio: Aggregate data and crude results from studies with available individual patient data**				
Donepezil vs Placebo	1.07	0.86 to 1.32	0.68 to 1.67	0.31
Rivastigmine oral vs Placebo	1.26	0.83 to 1.90	0.70 to 2.24	0.16
Galantamine vs Placebo	0.95	0.75 to 1.21	0.60 to 1.51	0.52
Rivastigmine transdermal vs Placebo	0.87	0.57 to 1.35	0.48 to 1.58	0.61
Memantine vs Placebo	0.91	0.67 to 1.22	0.55 to 1.49	0.59
Donepezil + Memantine vs Placebo	0.76	0.34 to 1.68	0.31 to 1.88	0.69
Galantamine + Memantine vs Placebo	1.03	0.45 to 2.36	0.41 to 2.64	0.42
Rivastigmine transdermal + Memantine vs Placebo	0.69	0.32 to 1.51	0.28 to 1.70	0.77
Placebo (reference)				0.43
Common within-network between-study variance τ^2 = 0.04, I^2 = 20% (0%, 47%)				
Design-by-treatment interaction model for inconsistency χ^2 (d.f., P-value, τ^2): 3.58 (6, 0.733, 0.05)				
**Odds Ratio: Aggregate data results**[**]				
Donepezil vs Placebo	1.09	0.89 to 1.33	0.88 to 1.35	0.25
Rivastigmine oral vs Placebo	1.43	0.92 to 2.21	0.90 to 2.26	0.07
Galantamine vs Placebo	0.88	0.63 to 1.25	0.62 to 1.27	0.54
Rivastigmine transdermal vs Placebo	0.79	0.44 to 1.41	0.43 to 1.45	0.61
Memantine vs Placebo	0.70	0.51 to 0.97	0.50 to 0.98	0.77
Donepezil + Memantine vs Placebo	0.77	0.39 to 1.54	0.37 to 1.60	0.64
Galantamine + Memantine vs Placebo	0.96	0.45 to 2.08	0.43 to 2.16	0.44
Rivastigmine transdermal + Memantine vs Placebo	0.62	0.28 to 1.40	0.27 to 1.46	0.80
Placebo (reference)				0.38
Common within-network between-study variance τ^2 = 0.00, I^2 = 0% (0%, 42%)				
Design-by-treatment interaction model for inconsistency χ^2 (d.f., P-value, τ^2): 2.29 (4, 0.682, 0.01)				
**Odds Ratio: Crude results from studies with available individual patient data**				
Donepezil vs Placebo	0.95	0.50 to 1.78	0.33 to 2.70	0.57
Rivastigmine oral vs Placebo	0.81	0.37 to 1.75	0.25 to 2.61	0.71
Galantamine vs Placebo	1.05	0.71 to 1.56	0.44 to 2.50	0.46
Rivastigmine transdermal vs Placebo	0.92	0.38 to 2.20	0.26 to 3.31	0.57
Memantine vs Placebo	1.41	0.81 to 2.45	0.53 to 3.79	0.16
				0.53
Common within-network between-study variance τ^2 = 0.10, I^2 = 48% (0%, 76%)				
Design-by-treatment interaction model for inconsistency χ^2 (d.f., P-value, τ^2): N/A (no closed loops)				
**Odds Ratio: Low Risk of Bias for Allocation Concealment***				
Donepezil vs Placebo	0.88	0.60 to 1.29	0.42 to 1.83	0.52
Rivastigmine oral vs Placebo	1.15	0.67 to 1.98	0.50 to 2.68	0.21
Galantamine vs Placebo	0.94	0.64 to 1.38	0.45 to 1.95	0.44
Rivastigmine transdermal vs Placebo	0.88	0.52 to 1.49	0.39 to 2.02	0.51
Memantine vs Placebo	0.86	0.55 to 1.36	0.40 to 1.88	0.54
Donepezil + Memantine vs Placebo	0.63	0.24 to 1.62	0.19 to 2.05	0.75
Rivastigmine transdermal + Memantine vs Placebo	0.67	0.25 to 1.80	0.20 to 2.28	0.71
Placebo (reference)				0.33
Common within-network between-study variance: τ^2 = 0.08, I^2 = 37% (0%, 64%)				
Design-by-treatment interaction model for inconsistency χ^2 (d.f., P-value, τ^2): 2.19 (3, 0.53, 0.1)				
**Odds Ratio: Low Risk of Bias for Incomplete Data***				
Donepezil vs Placebo	0.83	0.53 to 1.29	0.45 to 1.51	0.51
Galantamine vs Placebo	0.69	0.50 to 0.97	0.42 to 1.13	0.80
Rivastigmine transdermal vs Placebo	0.79	0.42 to 1.49	0.36 to 1.76	0.56
Memantine vs Placebo	0.86	0.60 to 1.22	0.51 to 1.43	0.47
Placebo (reference)				0.16

For peer review only - http://bmjopen.bmj.com/site/about/guidelines.xhtml

Common within-network between-study variance: τ^2 = 0.02, I^2 = 10% (0%, 50%)				
Design-by-treatment interaction model for inconsistency χ^2 (d.f., P-value, τ^2): 0.00 (1, 0.95, 0.04)				
**Odds Ratio: Publicly-Sponsored Studies***				
Donepezil vs Placebo	2.15	0.36 to 12.69	--	0.16
Memantine vs Placebo	0.71	0.45 to 1.12	--	0.86
Donepezil + Memantine vs Placebo	1.53	0.23 to 10.18	--	0.46
Placebo (reference)				0.51
Common within-network between-study variance: τ^2 = N/A (each comparison includes a single study)				
Design-by-treatment interaction model for inconsistency χ^2 (d.f., P-value, τ^2): N/A (no closed loops)				
**Odds Ratio: Industry-Sponsored Studies***				
Donepezil vs Placebo	1.08	0.86 to 1.35	0.64 to 1.82	0.34
Rivastigmine oral vs Placebo	1.27	0.82 to 1.98	0.66 to 2.44	0.16
Galantamine vs Placebo	0.99	0.75 to 1.31	0.57 to 1.71	0.52
Rivastigmine transdermal vs Placebo	0.91	0.57 to 1.44	0.46 to 1.77	0.62
Memantine vs Placebo	0.95	0.65 to 1.37	0.52 to 1.73	0.58
Rivastigmine transdermal + Memantine vs Placebo	0.72	0.31 to 1.64	0.27 to 1.90	0.79
Placebo (reference)				0.50
Common within-network between-study variance: τ^2 = 0.05, I^2 = 25% (0%, 50%)				
Design-by-treatment interaction model for inconsistency χ^2 (d.f., P-value, τ^2): 3.68 (6, 0.72, 0.07)				
**Odds Ratio: Studies with Mild to Moderate cognitive impairment, assessed with MMSE at baseline ***				
Donepezil vs Placebo	1.27	0.88 to 1.83	0.61 to 2.65	0.29
Rivastigmine oral vs Placebo	1.36	0.83 to 2.24	0.60 to 3.09	0.25
Galantamine vs Placebo	1.01	0.67 to 1.55	0.47 to 2.19	0.56
Rivastigmine transdermal vs Placebo	1.02	0.50 to 2.05	0.39 to 2.69	0.55
Memantine vs Placebo	0.86	0.54 to 1.37	0.39 to 1.91	0.73
Galantamine + Memantine vs Placebo	1.10	0.40 to 3.00	0.32 to 3.78	0.48
Rivastigmine transdermal + Memantine vs Placebo	0.96	0.18 to 5.19	0.14 to 6.37	0.55
Placebo (reference)				0.59
Common within-network between-study variance: τ^2 = 0.09, I^2 = 29% (0%, 57%)				
Design-by-treatment interaction model for inconsistency χ^2 (d.f., P-value, τ^2): 3.29 (5, 0.66, 0.13)				
**Odds Ratio: Studies with Moderate to Severe cognitive impairment, assessed with MMSE at baseline ***				
Donepezil vs Placebo	0.92	0.67 to 1.27	0.59 to 1.45	0.38
Galantamine vs Placebo	0.70	0.46 to 1.07	0.38 to 1.28	0.76
Memantine vs Placebo	0.95	0.55 to 1.62	0.44 to 2.02	0.36
Donepezil + Memantine vs Placebo	0.66	0.32 to 1.37	0.23 to 1.86	0.76
Placebo (reference)				0.23
Common within-network between-study variance: τ^2 = 0.00, I^2 = 0% (0%, 72%)				
Design-by-treatment interaction model for inconsistency χ^2 (d.f., P-value, τ^2): 2.90 (1, 0.09, 0.00)				
**Odds Ratio: NMA of studies with IPD – available case analysis**				
Donepezil vs Placebo	1.63	0.49 to 5.41	0.30 to 8.73	0.33
Rivastigmine oral vs Placebo	1.28	0.08 to 19.94	0.04 to 39.11	0.46
Galantamine vs Placebo	1.05	0.67 to 1.63	0.38 to 2.85	0.58
Rivastigmine transdermal vs Placebo	0.81	0.02 to 35.04	0.01 to 82.49	0.59
Memantine vs Placebo	1.35	0.72 to 2.55	0.43 to 4.24	0.38
Placebo (reference)				0.64
Common within-network between-study variance: τ^2 = 0.13, I^2 = 50% (0%, 77%)				
Design-by-treatment interaction model for inconsistency χ^2 (d.f., P-value, heterogeneity): N/A (no closed loops)				
**Odds Ratio: Meta-regression, Trial Mean Age**[**]**				
Donepezil vs Placebo	1.13	0.88 to 1.43	0.68 to 1.86	0.25 [††]
Rivastigmine oral vs Placebo	1.52	0.89 to 2.53	0.77 to 3.04	0.00 [††]
Galantamine vs Placebo	0.91	0.60 to 1.30	0.52 to 1.59	0.50 [††]
Rivastigmine transdermal vs Placebo	0.84	0.39 to 1.58	0.34 to 1.80	0.75 [††]
Memantine vs Placebo	0.74	0.48 to 1.07	0.39 to 1.26	0.75 [††]
Donepezil + Memantine vs Placebo	0.92	0.38 to 1.89	0.33 to 2.15	0.62 [††]
Galantamine + Memantine vs Placebo	0.99	0.37 to 2.27	0.33 to 2.55	0.50 [††]
Rivastigmine transdermal + Memantine vs Placebo	0.73	0.24 to 1.70	0.22 to 1.87	0.87 [††]
Placebo (reference)				0.37 [††]
Regression coefficient (log-scale)	-0.03	-0.08 to 0.02		
Common within-network between-study variance: τ^2 = 0.02	0.00 to 0.19			
Design-by-treatment interaction model for inconsistency χ^2 (d.f., P-value, τ^2): 3.57 (6, 0.735, 0.06)				
**Odds Ratio: NMA of studies with IPD adjusted for Age**				
Donepezil vs Placebo	0.95	0.50 to 1.78	0.33 to 2.73	0.57
Rivastigmine oral vs Placebo	0.84	0.39 to 1.81	0.26 to 2.74	0.68
Galantamine vs Placebo	1.04	0.70 to 1.55	0.43 to 2.52	0.46
Rivastigmine transdermal vs Placebo	0.91	0.38 to 2.17	0.25 to 3.28	0.58
Memantine vs Placebo	1.39	0.80 to 2.44	0.52 to 3.79	0.17
Placebo (reference)				0.53
Common within-network between-study variance: τ^2 = 0.10, I^2 = 48% (0%, 76%)				
Design-by-treatment interaction model for inconsistency χ^2 (d.f., P-value, τ^2): N/A (no closed loops)				

For peer review only - http://bmjopen.bmj.com/site/about/guidelines.xhtml

**Odds Ratio: Meta-regression, Percent of Male Participants[**]**				
Donepezil vs Placebo	1.12	0.87 to 1.44	0.64 to 2.01	0.25[††]
Rivastigmine oral vs Placebo	1.71	0.97 to 2.92	0.83 to 3.67	0.00[††]
Galantamine vs Placebo	0.93	0.62 to 1.36	0.49 to 1.77	0.50[††]
Rivastigmine transdermal vs Placebo	0.89	0.39 to 1.79	0.34 to 2.05	0.63[††]
Memantine vs Placebo	0.64	0.37 to 1.00	0.29 to 1.21	0.88[††]
Donepezil + Memantine vs Placebo	0.88	0.35 to 1.88	0.30 to 2.13	0.63[††]
Galantamine + Memantine vs Placebo	1.13	0.39 to 2.58	0.36 to 2.95	0.38[††]
Rivastigmine transdermal + Memantine vs Placebo	0.77	0.24 to 1.93	0.21 to 2.13	0.88[††]
Placebo (reference)				0.38[††]
Regression coefficient (log-scale)	0.00	0.00 to 0.02		
Common within-network between-study variance: τ^2 = 0.03	0.00 to 0.23			
Design-by-treatment interaction model for inconsistency χ^2 *(d.f., P-value,* τ^2*)*: 3.57 (6, 0.735, 0.06)				
**Odds Ratio: NMA of studies with IPD adjusted for Percent of Male Participants**				
Donepezil vs Placebo	1.04	0.54 to 1.99	0.34 to 3.16	0.49
Rivastigmine oral vs Placebo	0.81	0.37 to 1.80	0.24 to 2.79	0.72
Galantamine vs Placebo	1.05	0.70 to 1.59	0.42 to 2.65	0.48
Rivastigmine transdermal vs Placebo	0.92	0.37 to 2.27	0.24 to 3.52	0.58
Memantine vs Placebo	1.40	0.80 to 2.48	0.50 to 3.98	0.19
Placebo (reference)				0.55
Common within-network between-study variance: τ^2 = 0.11, I^2 = 51% (0%, 77%)				
Design-by-treatment interaction model for inconsistency χ^2 *(d.f., P-value,* τ^2*)*: N/A (no closed loops)				
**Odds Ratio: NMA of studies with IPD adjusted for cognitive impairment, assessed with MMSE at baseline**				
Donepezil vs Placebo	0.97	0.46 to 2.06	0.23 to 4.03	0.56
Rivastigmine oral vs Placebo	0.81	0.33 to 2.01	0.17 to 3.91	0.70
Galantamine vs Placebo	1.29	0.74 to 2.25	0.37 to 4.55	0.28
Rivastigmine transdermal vs Placebo	0.93	0.34 to 2.53	0.18 to 4.91	0.57
Memantine vs Placebo	1.26	0.59 to 2.70	0.30 to 5.28	0.33
Placebo (reference)				0.56
Common within-network between-study variance: τ^2 = 0.16, I^2 = 52% (0%, 80%)				
Design-by-treatment interaction model for inconsistency χ^2 *(d.f., P-value,* τ^2*)*: N/A (no closed loops)				
**Odds Ratio: NMA of studies with IPD adjusted for comorbidities**				
Donepezil vs Placebo	1.01	0.52 to 1.96	0.29 to 3.50	0.51
Rivastigmine oral vs Placebo	0.82	0.36 to 1.87	0.20 to 3.32	0.69
Galantamine vs Placebo	1.02	0.57 to 1.80	0.32 to 3.26	0.50
Rivastigmine transdermal vs Placebo	0.91	0.36 to 2.31	0.20 to 4.11	0.58
Memantine vs Placebo	1.42	0.79 to 2.55	0.44 to 4.59	0.18
Placebo (reference)				0.53
Common within-network between-study variance: τ^2 = 0.12, I^2 = 44% (0%, 77%)				
Design-by-treatment interaction model for inconsistency χ^2 *(d.f., P-value,* τ^2*)*: N/A (no closed loops)				
**Odds Ratio: NMA of studies with IPD adjusted for other medications**				
Donepezil vs Placebo	1.17	0.49 to 3.03	0.28 to 4.88	0.41
Rivastigmine oral vs Placebo	0.82	0.37 to 1.81	0.23 to 2.91	0.72
Galantamine vs Placebo	1.03	0.69 to 1.55	0.40 to 2.65	0.51
Rivastigmine transdermal vs Placebo	0.95	0.39 to 2.34	0.24 to 2.91	0.56
Memantine vs Placebo	1.34	0.75 to 2.39	0.46 to 3.92	0.25
Placebo (reference)				0.56
Common within-network between-study variance: τ^2 = 0.11, I^2 = 51% (0%, 78%)				
Design-by-treatment interaction model for inconsistency χ^2 *(d.f., P-value,* τ^2*)*: N/A (no closed loops)				
**Odds Ratio: Meta-regression, Study Duration[**]**				
Donepezil vs Placebo	1.12	0.87 to 1.43	0.63 to 1.95	0.25[††]
Rivastigmine oral vs Placebo	1.76	1.00 to 2.99	0.88 to 3.68	0.00[††]
Galantamine vs Placebo	0.92	0.62 to 1.36	0.50 to 1.69	0.50[††]
Rivastigmine transdermal vs Placebo	0.87	0.39 to 1.70	0.34 to 1.96	0.63[††]
Memantine vs Placebo	0.61	0.37 to 0.93	0.31 to 1.13	0.88[††]
Donepezil + Memantine vs Placebo	0.76	0.29 to 1.69	0.26 to 1.90	0.75[††]
Galantamine + Memantine vs Placebo	0.98	0.34 to 2.26	0.30 to 2.53	0.50[††]
Rivastigmine transdermal + Memantine vs Placebo	0.75	0.25 to 1.81	0.23 to 1.97	0.75[††]
Placebo (reference)				0.38[††]
Regression coefficient (log-scale)	0.00	0.00 to 0.01		
Common within-network between-study variance: τ^2 = 0.03	0.00 to 0.22			
Design-by-treatment interaction model for inconsistency χ^2 *(d.f., P-value,* τ^2*)*: 3.57 (6, 0.735, 0.06)				
**Odds Ratio: Meta-regression, Year of Publication[**]**				
Donepezil vs Placebo	1.05	0.79 to 1.38	0.61 to 1.77	0.38[††]
Rivastigmine oral vs Placebo	1.68	0.98 to 2.77	0.85 to 3.37	0.00[††]
Galantamine vs Placebo	0.91	0.61 to 1.32	0.50 to 1.64	0.63[††]
Rivastigmine transdermal vs Placebo	0.92	0.40 to 1.84	0.36 to 2.04	0.63[††]
Memantine vs Placebo	0.73	0.46 to 1.05	0.38 to 1.28	0.88[††]
Donepezil + Memantine vs Placebo	0.88	0.35 to 1.83	0.31 to 2.15	0.75[††]

For peer review only - http://bmjopen.bmj.com/site/about/guidelines.xhtml

Galantamine + Memantine vs Placebo	1.24	0.43 to 2.85	0.39 to 3.25	0.25 ††
Rivastigmine transdermal + Memantine vs Placebo	0.88	0.24 to 2.24	0.24 to 2.42	0.75 ††
Placebo (reference)				0.38 ††
Regression coefficient (log-scale)	-0.02	-0.06 to 0.03		
Common within-network between-study variance: $\tau^2 = 0.02$	0.00 to 0.21			
Design-by-treatment interaction model for inconsistency χ^2 (d.f., P-value, τ^2): 3.57 (6, 0.735, 0.06)				

* Aggregate data and fully adjusted results from studies with available individual patient data
† MMSE: Studies with available IPD included only available participants – to assess the missing data impact on the second stage a separate analysis was applied (IMDoM)
‡ AE: Studies with available IPD included all randomized participants
§ Outlier studies:

- Hernandez C, Unturbe F, Martinez-Lage P, Lucas A, Gregorio P, Alonso T. Effects of combined pharmacologic and cognitive treatment in the progression of moderate dementia: a two-year follow-up. REVISTA ESPANOLA DE GERIATRIA Y GERONTOLOGIA. 2007;42(1):3
- Moretti DV. Alpha rhythm oscillations and MMSE scores are differently modified by transdermal or oral rivastigmine in patients with Alzheimer's disease. American journal of neurodegenerative disease. 2014;3(2):72-83.

¶ Included studies with available raw data only, irrespective having access to individual patient data
|| Analyses were conducted in Stata using the *metamiss2* and *network* commands; I2 is not available; SUCRA values are presented instead of P-scores
** Studies with aggregate data were used (studies with available individual patient data were not included in this analysis)
†† Analyses were conducted in OpenBUGS, and SUCRA values were calculated instead of P-scores

For peer review only

For peer review only - http://bmjopen.bmj.com/site/about/guidelines.xhtml

Appendix 17: CINeMA results

Risk of bias contributions: The bar chart shows the contributions of each piece of study to the network estimate

Interventions
A: PLAC
B: DONE
C: RIVA_O
D: GALA
E: RIVA_P
F: MEMA
G: DONE+MEMA
H: GALA+MEMA
I: RIVA_P+MEMA

MMSE outcome

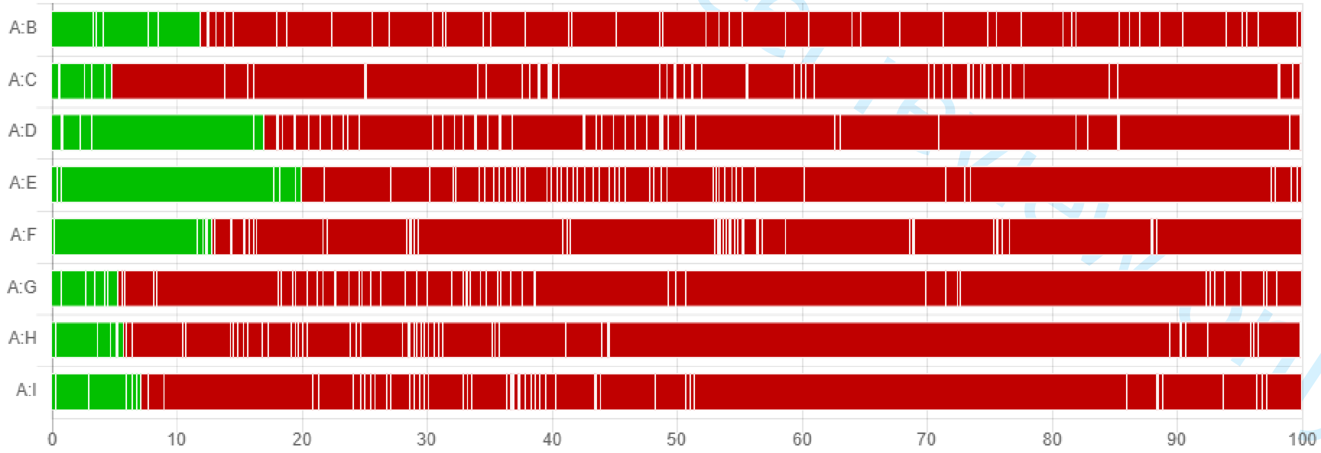

AE outcome

For peer review only - http://bmjopen.bmj.com/site/about/guidelines.xhtml

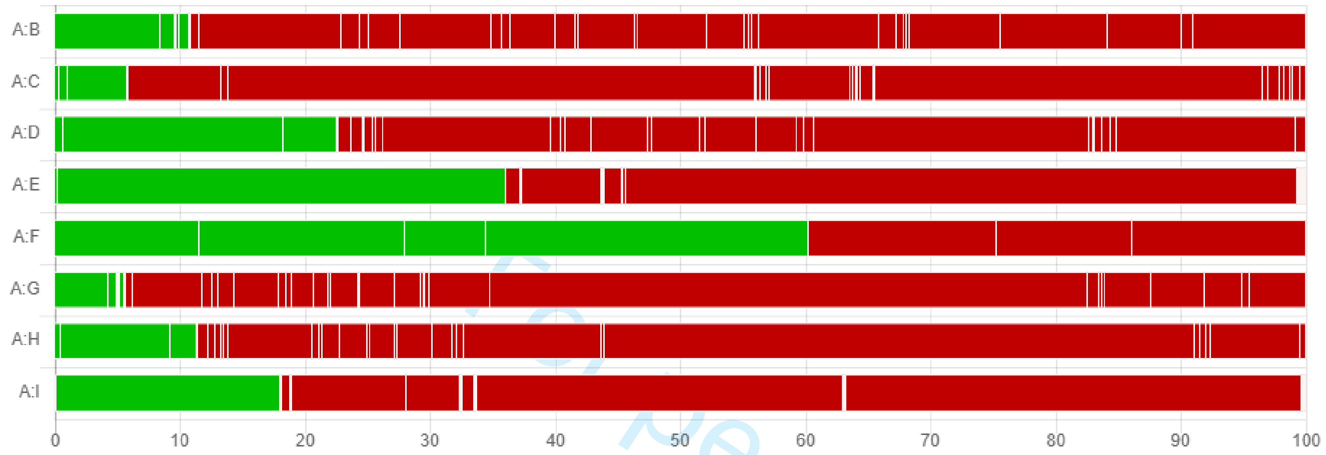

CINeMA report

MMSE outcome

Comparison	# of studies	Nature of evidence	Type of data	Within-study bias (D1)	Reporting bias (D2)	Indirectness (D3)	Imprecision (D4)	Heterogeneity (D5)	Incoherence (D6)	Confidence rating	Downgrading due to
DONE vs PLAC	24	Mixed	IPD+AD	Major concerns	Suspected	No concerns	No concerns	Major concerns	No concerns	Moderate	D5
RIVA_O vs PLAC	6	Mixed	IPD+AD	Major concerns	Suspected	No concerns	Some concerns	Some concerns	No concerns	Moderate	D4;D5
GALA vs PLAC	3	Mixed	IPD+AD	Major concerns	Suspected	No concerns	Major concerns	No concerns	No concerns	Moderate	D4
RIVA_P vs PLAC	2	Mixed	IPD+AD	Major concerns	Suspected	No concerns	Some concerns	Some concerns	No concerns	Moderate	D4;D5
MEMA vs PLAC	7	Mixed	IPD+AD	Major concerns	Suspected	No concerns	Some concerns	Some concerns	No concerns	Moderate	D4;D5
DONE+MEMA vs PLAC	1	Mixed	AD	Major concerns	Suspected	No concerns	No concerns	Major concerns	No concerns	Moderate	D5
GALA+MEMA vs PLAC	0	Indirect	-	Major concerns	Suspected	No concerns	Major concerns	No concerns	No concerns	Moderate	D4
RIVA_P+MEMA vs PLAC	0	Indirect	-	Major concerns	Suspected	No concerns	Major concerns	No concerns	No concerns	Moderate	D4

AE outcome

For peer review only - http://bmjopen.bmj.com/site/about/guidelines.xhtml

Comparison	# of studies	Nature of evidence	Type of data	Within-study bias (D1)	Reporting bias (D2)	Indirectness (D3)	Imprecision (D4)	Heterogeneity (D5)	Incoherence (D6)	Confidence rating	Downgrading due to
DONE vs PLAC	16	Mixed	IPD+AD	Major concerns	Undetected	No concerns	Major concerns	No concerns	No concerns	Moderate	D1
RIVA_O vs PLAC	3	Mixed	IPD+AD	Major concerns	Undetected	No concerns	Major concerns	No concerns	No concerns	Moderate	D1
GALA vs PLAC	8	Mixed	IPD+AD	Major concerns	Undetected	No concerns	Major concerns	No concerns	No concerns	Moderate	D1
RIVA_P vs PLAC	2	Mixed	IPD+AD	Some concerns	Undetected	No concerns	Major concerns	No concerns	No concerns	High	
MEMA vs PLAC	7	Mixed	IPD+AD	Some concerns	Undetected	No concerns	Major concerns	No concerns	No concerns	High	
DONE+MEMA vs PLAC	2	Mixed	AD	Major concerns	Undetected	No concerns	Major concerns	No concerns	No concerns	Moderate	D1
GALA+MEMA vs PLAC	0	Indirect	-	Major concerns	Undetected	No concerns	Major concerns	No concerns	No concerns	Moderate	D1
RIVA_P+MEMA vs PLAC	0	Indirect	-	Major concerns	Undetected	No concerns	Major concerns	No concerns	No concerns	Moderate	D1

Abbreviations: DONE, donepezil; GALA, galantamine; MEMA, memantine; PLAC, placebo; RIVA_O, rivastigmine oral; RIVA_P, rivastigmine patch

For peer review only - http://bmjopen.bmj.com/site/about/guidelines.xhtml

Appendix 18: Study definitions for adverse events

Author, Year	Source of Definition	Definition
Agid, 1998	Determined by Investigator	"Patients and caregivers were questioned systematically regarding the occurrence of adverse events at each clinical visit"
Ancoli-Israel, 2005	Determined by Investigator	"Only one serious AE leading to discontinuation, hepatic failure, in the donepezil-treated group was considered to be possibly due to study treatment by the investigator."
Andersen, 2012	NA	NA
Araki, 2014	NA	NA
Bakchine, 2008	Determined by Investigator	" A patient could also be withdrawn from the study if: they had a serious adverse event (SAE: death, life-threatening condition, hospitalisation) [..] Three patients had an SAE that was considered by the investigator to be possibly or probably related to treatment."
Black, 2007	Determined by Investigator	"AEs were considered serious (SAEs) when death occurred, life was threatened, hospitalization or prolonged hospitalization was required, or a significant disability occurred."
Blesa González, 2011	NA	NA
Burns,1999	COSTART	"Serious adverse events (SAE) included fatal or life-threatening situations, permanently disabling conditions or incidents that required or prolonged hospitalisation […] Events were coded using a modified COSTART dictionary, and the assessment of relationship to treatment for all adverse events was conducted blind to treatment assignment."
Burns, 2009	NR	NR
Burns, 2011	NR	NR
Choi, 2011	Determined by Investigator	"Investigators were asked to evaluate severity (mild, moderate, or severe), relationship to study drug (not related, probable relationship with rivastigmine patch, probable relationship with memantine, or probable relationship with an interaction of the two drugs), and seriousness of the AEs."
Corey-Bloom, 1998	NA	NA
Cretu, 2008	NA	NA
Dysken, 2014	Medical Dictionary for Regulatory Activities	"Serious AEs were coded according to the Medical Dictionary for Regulatory Activities."
Farlow, 2013	NA	NA
Feldman, 2001	Determined by Investigator	"Serious AE was defined as any AE that was life threatening or resulted in death, hospitalization, prolongation of hospitalization, or significant disability."
Feldman, 2007	World Health Organisation preferred terms	" A similar proportion of patients in each treatment group experienced at least one serious adverse event (any event that was fatal, considered life threatening or required hospitalisation) […] All adverse events were recorded using the Novartis Medical Terminology Thesaurus (a modified version of the WHO adverse reaction terminology dictionary)."
Fox, 2012	NA	NA
Frolich, 2011	NA	NA
Fuschillo, 2001	NA	NA
Gault L, 2015	Medical Dictionary for Regulatory Activities	"AEs were coded using the Medical Dictionary for Regulatory Activities"
Gold, 2010	NR	"SAE (fatal or nonfatal) "
Greenberg, 2000	Determined by Investigator	"Of 9 withdrawals from the study after randomization, 2 were due to serious adverse events judged to be possibly related to donepezil therapy: syncope and generalized seizure (1 patient each). "
Grossberg, 2013	Medical Dictionary for Regulatory Activities	"Adverse events were coded according to the Medical Dictionary for Regulatory Activities (version 7.0 or newer), and an assessment of the severity, chronicity, causal relationship to study medication, and seriousness of the event was provided by an investigator"
Hager, 2014	Determined by Investigator	"Safety data were monitored during the study by a company-commissioned, external, independent, blinded Data Safety Monitoring Board (DSMB). Secondary safety outcomes were the number of treatment emergent adverse events (TEAEs), including serious TEAEs."
Haig, 2014	Determined by Investigator	"The incidence of adverse events considered possibly or probably related to study drug as assessed by the investigator was generally similar across treatment groups (range 20.6% to 26.8%)." "Treatment emergent adverse events were tabulated by primary Medical Dictionary for Regulatory Activities (MedDRA) [23] version 13.1 System Organ Class and Preferred Term"
Hernández, 2007	NA	NA
Herrmann, 2013	Determined by Investigator	"The incidence of adverse events considered related to the study drug by the investigator was 30% in the placebo group and 36% in the memantine group"
Holmes, 2004	Determined by Investigator	"During these (clinic) visits, psychometric evaluations, medication compliance checks, and adverse event (AE) monitoring took place"

For peer review only - http://bmjopen.bmj.com/site/about/guidelines.xhtml

Homma, 1998	NR	NR
Homma, 2008	Medical Dictionary for Regulatory Activities – Japanese Version	"AE terms were standardized according to the Medical Dictionary for Regulatory Activities – Japanese Version . AEs were graded on a 3-point scale (mild: discomfort noticed, but no disruption of normal daily activity; moderate: discomfort sufficient to reduce or affect normal daily activity; severe: incapacitating, with inability to work or to perform normal daily activity). "
Hong, 2006	NR	NR
Howard, 2007	NA	NA
Howard, 2012	NR	NR
Hu, 2006	NA	NA
Johannsen, 2006	NA	NA
Jones, 2004	Determined by Investigator	"A serious adverse event (SAE) was defined as any AE that was life threatening or resulted in death, hospitalisation, prolongation of hospitalisation, or significant disability"
Kadir, 2008	NA	NA
Kano, 2013	NA	NA
Karaman, 2005	NA	NA
Likitjaroen, 2012	NA	NA
Lorenzi, 2011	NA	NA
Maher-Edwards, 2011	Determined by Investigator	"Eight subjects experienced nonfatal serious AEs; all were considered unrelated to the study drug"
Marek, 2014	Medical Dictionary for Regulatory Activities	"Aes were coded using the Medical Dictionary for Regulatory Activities (MedDRA, version 14.0) by system organ class and preferred term"
Mazza, 2006	NA	NA
Mohs, 2001	Determined by Investigator	"In all cases, judgment of the relationship of study treatment to an adverse event and of the severity of the event was made by the investigator under double-blind conditions. "
Moretti, 2014	NA	NA
Mowla, 2007	NA	NA
Nakamura, 2011	Determined by Investigator	"Safety evaluations included recording all adverse events on Adverse Event Case Report Forms. Every serious adverse event occurring after the patient provided informed consent and until 28 days after the patient stopped the study was reported. "
Nakano, 2001	NA	NA
Nordberg, 2009	Determined by Investigator	"Safety and tolerability were monitored throughout the study by recording all adverse events (AEs). "
Pakdaman H, 2015	NA	NA
Peng, 2005	NA	NA
Peskind, 2006	Determined by Investigator	"Overall, the type and incidence of SAEs were similar between the memantine and placebo groups. One participant death occurred in each group during the trial; neither was rated by the investigator as being treatment-related"
Peters O, 2015	NR	NR
Reisberg, 2003	NR	NR
Rockwood, 2001	World Health Organisation preferred terms	"adverse events (classified according to World Health Organisation preferred terms)."
Rockwood, 2006	NR	NR
Rogers, 1996		
Rogers, 1998	COSTART	"Events, recorded using investigator terminology, were grouped and coded into common terms using a modified COSTART dictionary"
Rogers, 1998	COSTART	"Events, recorded using investigator terminology, were grouped and coded into common terms using a modified COSTART dictionary. "
Saxton, 2012	Determined by Investigator	"Treatment-emergent adverse events (TEAEs) and serious adverse events (SAEs) were recorded at all post-Screening study visits"
Scarpini, 2011	Determined by Investigator	"Subjects with a treatment 51 (20.1) 2 (2.6) 4 (6.3) related AE, as judged by the investigator"
Schmidt, 2008	NA	NA
Seltzer, 2004	NA	NA
Shao, 2015	NA	NA
Shimizu, 2015	NA	NA
Sole-Padulles, 2013	NA	NA
Tariot, 2000	World Health Organisation preferred terms	"adverse events (classified according to World Health Organization Preferred Term). "
Tariot, 2001	COSTART	"Investigator terms describing AEs were coded to standard preferred terms using a modified Coding Symbols for Thesaurus of Adverse Reaction Terms dictionary. "
Thomas, 2001		

For peer review only - http://bmjopen.bmj.com/site/about/guidelines.xhtml

Wilcock, 2003	World Health Organisation preferred terms	"monitoring for adverse events (classified according to WHO preferred terms) "
Wilkinson, 2001	Determined by Investigator	"All adverse events were recorded, regardless of the considered relationship to treatment. All details of adverse events and their outcomes were recorded including severity and relationship to treatment. Serious adverse events were documented separately. "
Wilkinson, 2002	NR	NR
Wilkinson, 2012	Determined by Investigator	"Tolerability and safety were based on the incidence of adverse events, either reported spontaneously by the patients or in response to a non-leading question by the investigator throughout the study"
Winblad, 2001	NR	NR
Winblad, 2006	COSTART	"We recorded all treatment emergent adverse events, coding them according to a modified COSTART dictionary. "
Winblad, 2007	Determined by Investigator	"Safety evaluations included recording all adverse events, which were coded using a standard glossary."
Zhang-Yi, 2005	NA	NA
Zhang, 2012	Determined by Investigator	"Serious adverse events considered to be possibly related to treatment occurred in one patient in each treatment arm"

Notes: [a]Unpublished data, [b]Non-English studies
Abbreviations: CR, companion report; NA, not applicable; NR, not reported.

For peer review only - http://bmjopen.bmj.com/site/about/guidelines.xhtml

Appendix 19: Time taken to achieve at least an adverse event using individual patient data

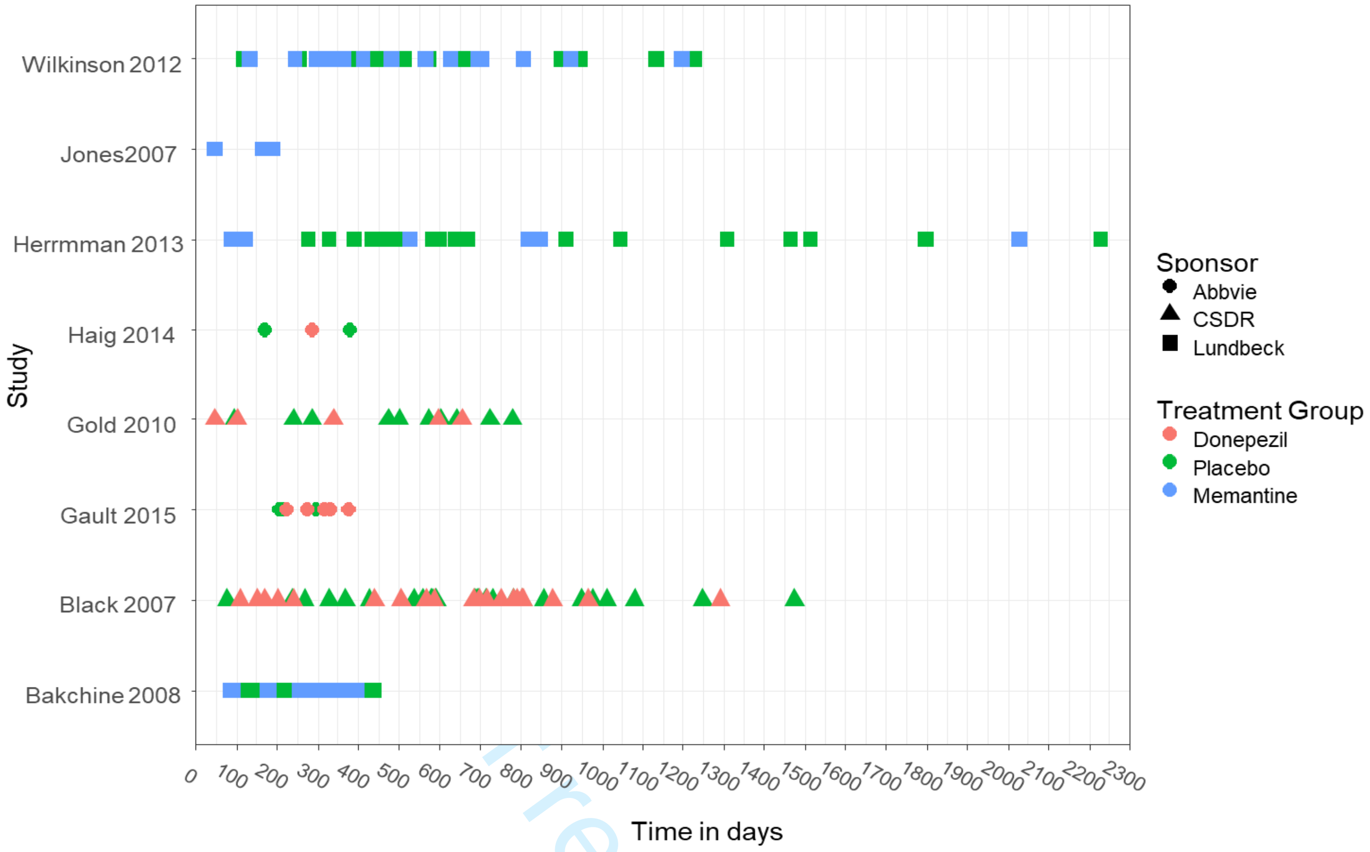

For peer review only - http://bmjopen.bmj.com/site/about/guidelines.xhtml

Appendix 20: Rank-heat plot for adverse events

Circles from inside out present results for different network meta-analyses including: i) aggregate data (AD) only (studies with available IPD are not included in the analysis), ii) crude results from individual studies with individual patient data (IPD), iii) AD and crude results from studies with available IPD, and iv) AD and fully adjusted results from studies with available IPD. Numbers within each sector correspond to the P-score values as calculated in each model.

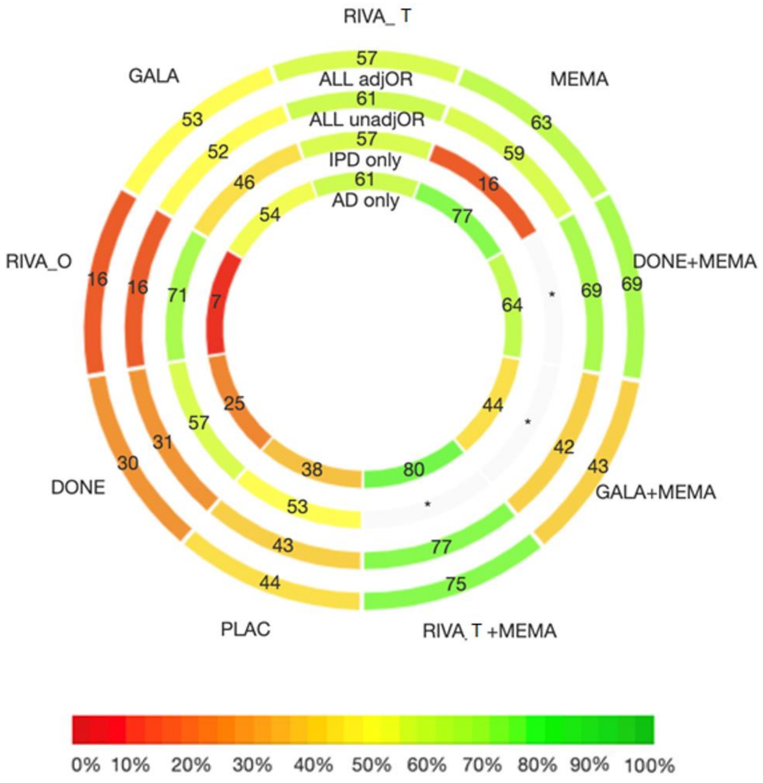

For peer review only - http://bmjopen.bmj.com/site/about/guidelines.xhtml

Appendix 21: Challenges encountered during the individual patient data request from sponsors

- The identification of the trial data set when certain details were not available (e.g. NCT number; particularly for studies published before 2005 that this was established).
- Data ownership.
- Sponsors switched platforms, while we were navigating the data.
- IPD available through proprietary sponsor-specific platforms did not allow for combination of IPD from different sponsor platforms; hence a one-stage analysis as planned in our protocol, was impossible.
- Software availability: Required R packages (e.g., mice) were not available/provided, and we were not allowed to install any new R packages; some R packages were older versions (e.g. lme4).
- Time that the platform permitted access to the IPD was often limited. This is a significant constraint given that IPD from different studies became available at different time points.
- Cost associated with obtaining access to the data for a certain amount of time. Additionally, cost associated with the WHO Drug Dictionary license to obtain access to the additional medications used for each patient; this license's approximate cost was $8,958·25 USD per sponsor.
- Available IPD did not include the full information as shown in the publication: For example, only data for placebo were available, or did not give information about a reported outcome (e.g. only baseline MMSE values were available). Also, date of follow-up was coded in some studies and it was impossible to make a judgement on first and last date.

For peer review only

Additional File 2: MEDLINE Search Strategy

MEDLINE Search

Database: Ovid MEDLINE(R) In-Process & Other Non-Indexed Citations and Ovid MEDLINE(R) <1946 to Present>, Embase<1980 to 2014 Week 50> Search Strategy:
----
1 alzheimer$.mp.
2 "benign senescent forgetfulness".mp.
3 (cognit$ adj2 (impair$ or declin$ or deficit$ or degenerat$ or deteriorat$ or los$ or disorder$ or complain$ or disturb$)).mp.
4 (cerebr$ adj2 (impair$ or declin$ or deficit$ or degenerat$ or deteriorat$ or los$ or disorder$ or complain$ or disturb$)).mp.
5 (mental adj2 (impair$ or declin$ or deficit$ or degenerat$ or deteriorat$ or los$ or disorder$ or complain$ or disturb$)).mp.
6 (ne?rocognit$ adj2 (impair$ or declin$ or deficit$ or degenerat$ or deteriorat$ or los$ or disorder$ or complain$ or disturb$)).mp.)
7 (ne?ro-cognit$ adj2 (impair$ or declin$ or deficit$ or degenerat$ or deteriorat$ or los$ or disorder$ or complain$ or disturb$)).mp.
8 ((cognit$ or memory or cerebral or brain) adj2 (improv$ or enhanc$ or perform$ or process$ or function$ or rehabilitation or aid$ or stimulat$)).mp.
9 cognition.ti.
10 (confusion$ or confused).tw.
11 dement$.mp.
12 ("normal pressure hydrocephalus" and shunt$).mp.
13 "organic brain disease$".mp.
14 "organic brain syndrome".mp.
15 (presenil$ or pre-senil$ or senil$).tw.
16 Alzheimer Disease/
17 Cognition/de
18 Confusion/
19 Dementia/
20 or/1-19
21 abixa.tw.
22 aricept.tw.
23 (acetylcholinesteraseadj inhibitor$).tw.
24 axura.tw.
25 akatinol.tw.
26 (anticholinesterase? or anti-cholinesterase?).tw.
27 (cognitive adjenhanc$).mp.
28 (cholinesterase adj inhibitor$).mp.
29 ChEI.tw.
30 donepezil.mp.
31 ebixa.tw.
32 eranz.tw.
33 exelon.tw.
34 galant?amin$.tw.
35 lycoremine.tw.

For peer review only - http://bmjopen.bmj.com/site/about/guidelines.xhtml

36 memantin$.tw.
37 memox.tw.
38 namenda.tw.
39 nimvastid.tw.
40 nivalin$.tw.
41 "N-Methyl-D-aspartic acid receptor antagonist$".tw.
42 prometax.tw.
43 razadyne.tw.
44 reminyl.tw.
45 rivastigmine.mp.
46 exp Cholinesterase Inhibitors/
47 Galantamine/
48 Memantine/
49 Galantamin.rn.
50 Memantine.rn.
51 Donepezil.rn.
52 Donepezil Hydrochloride.rn.
53 Rivastigmine.rn.
54 or/21-53
55 20 and 54
56 exp Animals/ not (exp Animals/ and Humans/)
57 55 and 56
58 (comment or editorial or interview or news).pt.
59 (letter not (letter and randomized controlled trial)).pt.
60 57 not (58 or 59)
61 (201111* or 201112* or 2012* or 2013* or 2014*).ed.
62 60 and 61
63 alzheimer$.mp.
64 "benign senescent forgetfulness".mp.
65 (cognit$ adj2 (impair$ or declin$ or deficit$ or degenerat$ or deteriorat$ or los$ or disorder$ or complain$ or disturb$)).mp.
66 (cerebr$ adj2 (impair$ or declin$ or deficit$ or degenerat$ or deteriorat$ or los$ or disorder$ or complain$ or disturb$)).mp.
67 (mental adj2 (impair$ or declin$ or deficit$ or degenerat$ or deteriorat$ or los$ or disorder$ or complain$ or disturb$)).mp.
68 (ne?rocognit$ adj2 (impair$ or declin$ or deficit$ or degenerat$ or deteriorat$ or los$ or disorder$ or complain$ or disturb$)).mp.
69 (ne?ro-cognit$ adj2 (impair$ or declin$ or deficit$ or degenerat$ or deteriorat$ or los$ or disorder$ or complain$ or disturb$)).mp.
70 ((cognit$ or memory or cerebral or brain) adj2 (improv$ or enhanc$ or perform$ or process$ or function$ or rehabilitation or aid$ or stimulat$)).mp.
71 cognition.ti.
72 (confusion$ or confused).tw.
73 dement$.mp.
74 ("normal pressure hydrocephalus" and shunt$).mp.
75 "organic brain disease$".mp.
76 "organic brain syndrome".mp.

For peer review only - http://bmjopen.bmj.com/site/about/guidelines.xhtml

77 (presenil$ or pre-senil$ or senil$).tw
78 Alzheimer disease/
79 cognitive defect/
80 confusion/
81 dementia/
82 organic brain syndrome/
83 or/63-82
84 abixa.tw.
85 aricept.tw.
86 (acetylcholinesteraseadj inhibitor$).tw.
87 axura.tw.
88 akatinol.tw.
89 (anticholinesterase? or anti-cholinesterase?).tw.
90 (cognitive adjenhanc$).mp.
91 (cholinesterase adj inhibitor$).mp.
92 ChEI.tw.
93 donepezil.mp.
94 ebixa.tw.
95 eranz.tw.
96 exelon.tw.
97 galant?amin$.tw.
98 lycoremine.tw.
99 memantin$.tw.
100 memox.tw.
101 namenda.tw.
102 nimvastid.tw.
103 nivalin$.tw.
104 "N-Methyl-D-aspartic acid receptor antagonist$".tw.
105 prometax.tw.
106 razadyne.tw.
107 reminyl.tw.
108 rivastigmine.mp.
109 exp cholinesterase inhibitor/
110 donepezil/ or donepezil plus memantine/
111 galantamine/
112 memantine/
113 rivastigmine/
114 357-70-0.rn.
115 19982-08-2.rn.
116 120011-70-3.rn.
117 120014-06-4.rn.
118 rivastigmine.rn.
119 or/84-118
120 83 and 119
121 randomized controlled trial/ or controlled clinical trial/
122 exp "clinical trial (topic)"/
123 (randomi#ed or randomly or RCT$1 or placebo*).tw.

For peer review only - http://bmjopen.bmj.com/site/about/guidelines.xhtml

124 ((singl* or doubl* or trebl* or tripl*) adj (mask* or blind* or dumm*)).tw.
125 trial.ti.
126 or/121-125
127 120 and 126
128 exp controlled clinical trial/
129 exp "controlled clinical trial (topic)"/
130 (control* adj2 trial*).tw.
131 (nonrandom* or non-random* or quasi-random* or quasi-experiment*).tw.
132 (nRCT or nRCTs or non-RCT$1).tw.
133 (control* adj3 ("before and after" or "before after")).tw.
134 time series analysis/
135 (time series adj3 interrupt*).tw.
136 pretest posttest control group design/
137 (pre- adj3 post-).tw.
138 (pretest adj3 posttest).tw.
139 controlled study/
140 (control* adj2 stud$3).tw.
141 control group/
142 (control$ adj2 group$1).tw.
143 or/128-142
144 120 and 143
145 cohort analysis/
146 cohort.tw.
147 retrospective study/
148 longitudinal study/
149 prospective study/
150 (longitudinal or prospective or retrospective).tw.
151 follow up/
152 ((followup or follow-up) adj (study or studies)).tw.
153 observational study/
154 (observation$2 adj (study or studies)).tw.
155 population research/
156 ((population or population-based) adj (study or studies or analys#s)).tw.
157 ((multidimensional or multi-dimensional) adj (study or studies)).tw.
158 exp comparative study/
159 ((comparative or comparison) adj (study or studies)).tw.
160 exp case control study/
161 ((case-control* or case-based or case-comparison) adj (study or studies)).tw.
162 or/145-161
163 120 and 162
164 127 or 144 or 163
165 exp animal experimentation/ or exp models animal/ or exp animal experiment/ or nonhuman/ or exp vertebrate/
166 exp humans/ or exp human experimentation/ or exp human experiment/
167 165 not 166
168 164 not 167
169 editorial.pt.

For peer review only - http://bmjopen.bmj.com/site/about/guidelines.xhtml
For peer review only

170 letter.pt.not (letter.pt. and randomized controlled trial/)
171 168 not (169 or 170)
172 (2011112* or 2011113* or 201112* or 2012* or 2013* or 2014*).dd.
173 171 and 172
174 62 use prmz
175 173 use emez
176 174 or 175
177 remove duplicates from 176
178 177 use prmz [MEDLINE UNIQUE HITS]
179 177 use emez [EMBASE UNIQUE HITS]
****

For peer review only

For peer review only - http://bmjopen.bmj.com/site/about/guidelines.xhtml

PRISMA 2020 for Abstracts Checklist

Section and Topic	Item #	Checklist item	Reported (Yes/No)
**TITLE**			
Title	1	Identify the report as a systematic review.	Yes
**BACKGROUND**			
Objectives	2	Provide an explicit statement of the main objective(s) or question(s) the review addresses.	Yes
**METHODS**			
Eligibility criteria	3	Specify the inclusion and exclusion criteria for the review.	Yes
Information sources	4	Specify the information sources (e.g. databases, registers) used to identify studies and the date when each was last searched.	Yes
Risk of bias	5	Specify the methods used to assess risk of bias in the included studies.	Yes
Synthesis of results	6	Specify the methods used to present and synthesise results.	Yes
**RESULTS**			
Included studies	7	Give the total number of included studies and participants and summarise relevant characteristics of studies.	Yes
Synthesis of results	8	Present results for main outcomes, preferably indicating the number of included studies and participants for each. If meta-analysis was done, report the summary estimate and confidence/credible interval. If comparing groups, indicate the direction of the effect (i.e. which group is favoured).	Yes
**DISCUSSION**			
Limitations of evidence	9	Provide a brief summary of the limitations of the evidence included in the review (e.g. study risk of bias, inconsistency and imprecision).	Yes
Interpretation	10	Provide a general interpretation of the results and important implications.	Yes
**OTHER**			
Funding	11	Specify the primary source of funding for the review.	Yes
Registration	12	Provide the register name and registration number.	Yes

From: Page MJ, McKenzie JE, Bossuyt PM, Boutron I, Hoffmann TC, Mulrow CD, et al. The PRISMA 2020 statement: an updated guideline for reporting systematic reviews. BMJ 2021;372:n71. doi: 10.1136/bmj.n71

For more information, visit: http://www.prisma-statement.org/

For peer review only

PRISMA NMA Checklist of Items to Include When Reporting A Systematic Review Involving a Network Meta-analysis

Section/Topic	Item #	Checklist Item	Reported on Page #
**TITLE**			
Title	1	Identify the report as a systematic review *incorporating a network meta-analysis (or related form of meta-analysis).*	1
**ABSTRACT**			
Structured summary	2	Provide a structured summary including, as applicable:<br>**Background:** main objectives<br>**Methods:** data sources; study eligibility criteria, participants, and interventions; study appraisal; and *synthesis methods, such as network meta-analysis.*<br>**Results:** number of studies and participants identified; summary estimates with corresponding confidence/credible intervals; *treatment rankings may also be discussed. Authors may choose to summarize pairwise comparisons against a chosen treatment included in their analyses for brevity.*<br>**Discussion/Conclusions:** limitations; conclusions and implications of findings.<br>**Other:** primary source of funding; systematic review registration number with registry name.	3-4
**INTRODUCTION**			
Rationale	3	Describe the rationale for the review in the context of what is already known*, including mention of why a network meta-analysis has been conducted.*	5
Objectives	4	Provide an explicit statement of questions being addressed, with reference to participants, interventions, comparisons, outcomes, and study design (PICOS).	5
**METHODS**			
Protocol and registration	5	Indicate whether a review protocol exists and if and where it can be accessed (e.g., Web address); and, if available, provide registration information, including registration number.	5, Appendix 1
Eligibility criteria	6	Specify study characteristics (e.g., PICOS, length of follow-up) and report characteristics (e.g., years considered, language, publication status) used as criteria for eligibility, giving rationale. *Clearly describe eligible treatments included in the treatment network, and note whether any have been clustered or merged into the same node (with justification).*	6, Appendix 1
Information sources	7	Describe all information sources (e.g., databases with dates of coverage, contact with study authors to identify additional studies) in the search and date last searched.	6, Appendix 1

For peer review only

Search	8	Present full electronic search strategy for at least one database, including any limits used, such that it could be repeated.	N/A (see published protocol)
Study selection	9	State the process for selecting studies (i.e., screening, eligibility, included in systematic review, and, if applicable, included in the meta-analysis).	6, Appendix 1
Data collection process	10	Describe method of data extraction from reports (e.g., piloted forms, independently, in duplicate) and any processes for obtaining and confirming data from investigators.	6, Appendix 1
Data items	11	List and define all variables for which data were sought (e.g., PICOS, funding sources) and any assumptions and simplifications made.	6, Appendix 1
**Geometry of the network**	**S1**	Describe methods used to explore the geometry of the treatment network under study and potential biases related to it. This should include how the evidence base has been graphically summarized for presentation, and what characteristics were compiled and used to describe the evidence base to readers.	7, Appendix 1
Risk of bias within individual studies	12	Describe methods used for assessing risk of bias of individual studies (including specification of whether this was done at the study or outcome level), and how this information is to be used in any data synthesis.	6, Appendix 1
Summary measures	13	State the principal summary measures (e.g., risk ratio, difference in means). *Also describe the use of additional summary measures assessed, such as treatment rankings and surface under the cumulative ranking curve (SUCRA) values, as well as modified approaches used to present summary findings from meta-analyses.*	7, Appendix 1
Planned methods of analysis	14	Describe the methods of handling data and combining results of studies for each network meta-analysis. This should include, but not be limited to:<br>• *Handling of multi-arm trials;*<br>• *Selection of variance structure;*<br>• *Selection of prior distributions in Bayesian analyses; and*<br>• *Assessment of model fit.*	7, Appendix 1
**Assessment of Inconsistency**	**S2**	Describe the statistical methods used to evaluate the agreement of direct and indirect evidence in the treatment network(s) studied. Describe efforts taken to address its presence when found.	7, Appendix 1
Risk of bias across studies	15	Specify any assessment of risk of bias that may affect the cumulative evidence (e.g., publication bias, selective reporting within studies).	6, Appendix 1
Additional analyses	16	Describe methods of additional analyses if done, indicating which were pre-specified. This may include, but not be limited to, the following:<br>• Sensitivity or subgroup analyses;<br>• Meta-regression analyses;<br>• *Alternative formulations of the treatment network; and*<br>• *Use of alternative prior distributions for Bayesian analyses (if applicable).*	7, Appendix 1

For peer review only

RESULTS†

Study selection	17	Give numbers of studies screened, assessed for eligibility, and included in the review, with reasons for exclusions at each stage, ideally with a flow diagram.	7 – Figure 1
**Presentation of network structure**	**S3**	Provide a network graph of the included studies to enable visualization of the geometry of the treatment network.	9 – Figure 2
**Summary of network geometry**	**S4**	Provide a brief overview of characteristics of the treatment network. This may include commentary on the abundance of trials and randomized patients for the different interventions and pairwise comparisons in the network, gaps of evidence in the treatment network, and potential biases reflected by the network structure.	7-8
Study characteristics	18	For each study, present characteristics for which data were extracted (e.g., study size, PICOS, follow-up period) and provide the citations.	8 – Table 1, Appendix 5
Risk of bias within studies	19	Present data on risk of bias of each study and, if available, any outcome level assessment.	8-9 – Appendix 8
Results of individual studies	20	For all outcomes considered (benefits or harms), present, for each study: 1) simple summary data for each intervention group, and 2) effect estimates and confidence intervals. *Modified approaches may be needed to deal with information from larger networks.*	Appendices 6 and 10 (full data can be provided by the first author)
Synthesis of results	21	Present results of each meta-analysis done, including confidence/credible intervals. *In larger networks, authors may focus on comparisons versus a particular comparator (e.g. placebo or standard care), with full findings presented in an appendix. League tables and forest plots may be considered to summarize pairwise comparisons.* If additional summary measures were explored (such as treatment rankings), these should also be presented.	9-11 – Appendix 15
**Exploration for inconsistency**	**S5**	Describe results from investigations of inconsistency. This may include such information as measures of model fit to compare consistency and inconsistency models, *P* values from statistical tests, or summary of inconsistency estimates from different parts of the treatment network.	9 - Appendix 14
Risk of bias across studies	22	Present results of any assessment of risk of bias across studies for the evidence base being studied.	9-11 - Appendix 12
Results of additional analyses	23	Give results of additional analyses, if done (e.g., sensitivity or subgroup analyses, meta-regression analyses*, alternative network geometries studied, alternative choice of prior distributions for Bayesian analyses,* and so forth).	9-11 - Appendices 16 and 17

**DISCUSSION**			
Summary of evidence	24	Summarize the main findings, including the strength of evidence for each main outcome; consider their relevance to key groups (e.g., healthcare providers, users, and policy-makers).	11-13
Limitations	25	Discuss limitations at study and outcome level (e.g., risk of bias), and at review level (e.g., incomplete retrieval of identified research, reporting bias). *Comment on the validity of the assumptions, such as transitivity and consistency. Comment on any concerns regarding network geometry (e.g., avoidance of certain comparisons).*	13-14
Conclusions	26	Provide a general interpretation of the results in the context of other evidence, and implications for future research.	13-14
**FUNDING**			
Funding	27	Describe sources of funding for the systematic review and other support (e.g., supply of data); role of funders for the systematic review. This should also include information regarding whether funding has been received from manufacturers of treatments in the network and/or whether some of the authors are content experts with professional conflicts of interest that could affect use of treatments in the network.	15

PICOS = population, intervention, comparators, outcomes, study design.
* Text in italics indicateS wording specific to reporting of network meta-analyses that has been added to guidance from the PRISMA statement.
† Authors may wish to plan for use of appendices to present all relevant information in full detail for items in this section.

For peer review only

PRISMA-IPD Checklist of items to include when reporting a systematic review and meta-analysis of individual participant data (IPD)

PRISMA-IPD Section/topic	Item No	Checklist item	Reported on page
**Title**			
Title	1	Identify the report as a systematic review and meta-analysis of individual participant data.	1
**Abstract**			
Structured summary	2	Provide a structured summary including as applicable:	3-4
		**Background**: state research question and main objectives, with information on participants, interventions, comparators and outcomes.	
		**Methods**: report eligibility criteria; data sources including dates of last bibliographic search or elicitation, noting that IPD were sought; methods of assessing risk of bias.	
		**Results**: provide number and type of studies and participants identified and number (%) obtained; summary effect estimates for main outcomes (benefits and harms) with confidence intervals and measures of statistical heterogeneity. Describe the direction and size of summary effects in terms meaningful to those who would put findings into practice.	
		**Discussion:** state main strengths and limitations of the evidence, general interpretation of the results and any important implications.	
		**Other:** report primary funding source, registration number and registry name for the systematic review and IPD meta-analysis.	
**Introduction**			
Rationale	3	Describe the rationale for the review in the context of what is already known.	5
Objectives	4	Provide an explicit statement of the questions being addressed with reference, as applicable, to participants, interventions, comparisons, outcomes and study design (PICOS). Include any hypotheses that relate to particular types of participant-level subgroups.	5
**Methods**			

For peer review only

Protocol and registration	5	Indicate if a protocol exists and where it can be accessed. If available, provide registration information including registration number and registry name. Provide publication details, if applicable.	5, Appendix 1
Eligibility criteria	6	Specify inclusion and exclusion criteria including those relating to participants, interventions, comparisons, outcomes, study design and characteristics (e.g. years when conducted, required minimum follow-up). Note whether these were applied at the study or individual level i.e. whether eligible participants were included (and ineligible participants excluded) from a study that included a wider population than specified by the review inclusion criteria. The rationale for criteria should be stated.	5-6, Appendix 1
Identifying studies - information sources	7	Describe all methods of identifying published and unpublished studies including, as applicable: which bibliographic databases were searched with dates of coverage; details of any hand searching including of conference proceedings; use of study registers and agency or company databases; contact with the original research team and experts in the field; open adverts and surveys. Give the date of last search or elicitation.	6, Appendix 1
Identifying studies - search	8	Present the full electronic search strategy for at least one database, including any limits used, such that it could be repeated.	N/A (see published protocol)
Study selection processes	9	State the process for determining which studies were eligible for inclusion.	6, Appendix 1
Data collection processes	10	Describe how IPD were requested, collected and managed, including any processes for querying and confirming data with investigators. If IPD were not sought from any eligible study, the reason for this should be stated (for each such study).<br><br>If applicable, describe how any studies for which IPD were not available were dealt with. This should include whether, how and what aggregate data were sought or extracted from study reports and publications (such as extracting data independently in duplicate) and any processes for obtaining and confirming these data with investigators.	6, Appendix 1
Data items	11	Describe how the information and variables to be collected were chosen. List and define all study level and participant level data that were sought, including baseline and follow-up information. If applicable, describe methods of standardising or translating variables within the IPD datasets to ensure common scales or measurements across studies.	6, Appendix 1

For peer review only

IPD integrity	A1	Describe what aspects of IPD were subject to data checking (such as sequence generation, data consistency and completeness, baseline imbalance) and how this was done.	Appendix 1
Risk of bias assessment in individual studies.	12	Describe methods used to assess risk of bias in the individual studies and whether this was applied separately for each outcome. If applicable, describe how findings of IPD checking were used to inform the assessment. Report if and how risk of bias assessment was used in any data synthesis.	6, Appendix 1
Specification of outcomes and effect measures	13	State all treatment comparisons of interests. State all outcomes addressed and define them in detail. State whether they were pre-specified for the review and, if applicable, whether they were primary/main or secondary/additional outcomes. Give the principal measures of effect (such as risk ratio, hazard ratio, difference in means) used for each outcome.	7, Appendix 1
Synthesis methods	14	Describe the meta-analysis methods used to synthesise IPD. Specify any statistical methods and models used. Issues should include (but are not restricted to):<br>• Use of a one-stage or two-stage approach.<br>• How effect estimates were generated separately within each study and combined across studies (where applicable).<br>• Specification of one-stage models (where applicable) including how clustering of patients within studies was accounted for.<br>• Use of fixed or random effects models and any other model assumptions, such as proportional hazards.<br>• How (summary) survival curves were generated (where applicable).<br>• Methods for quantifying statistical heterogeneity (such as I^2 and τ^2).<br>• How studies providing IPD and not providing IPD were analysed together (where applicable).<br>• How missing data within the IPD were dealt with (where applicable).	7, Appendix 1
Exploration of variation in effects	A2	If applicable, describe any methods used to explore variation in effects by study or participant level characteristics (such as estimation of interactions between effect and covariates). State all participant-level characteristics that were analysed as potential effect modifiers, and whether these were pre-specified.	Appendix 1
Risk of bias across studies	15	Specify any assessment of risk of bias relating to the accumulated body of evidence, including any pertaining to not obtaining IPD for particular studies, outcomes or other variables.	6, Appendix 1

For peer review only

Additional analyses	16	Describe methods of any additional analyses, including sensitivity analyses. State which of these were pre-specified.	7, Appendix 1
**Results**			
Study selection and IPD obtained	17	Give numbers of studies screened, assessed for eligibility, and included in the systematic review with reasons for exclusions at each stage. Indicate the number of studies and participants for which IPD were sought and for which IPD were obtained. For those studies where IPD were not available, give the numbers of studies and participants for which aggregate data were available. Report reasons for non-availability of IPD. Include a flow diagram.	7 – Figure 1
Study characteristics	18	For each study, present information on key study and participant characteristics (such as description of interventions, numbers of participants, demographic data, unavailability of outcomes, funding source, and if applicable duration of follow-up). Provide (main) citations for each study. Where applicable, also report similar study characteristics for any studies not providing IPD.	8 – Table 1, Appendix 5
IPD integrity	A3	Report any important issues identified in checking IPD or state that there were none.	8-9, Appendices 5 and 10
Risk of bias within studies	19	Present data on risk of bias assessments. If applicable, describe whether data checking led to the up-weighting or down-weighting of these assessments. Consider how any potential bias impacts on the robustness of meta-analysis conclusions.	8-9 – Appendix 8
Results of individual studies	20	For each comparison and for each main outcome (benefit or harm), for each individual study report the number of eligible participants for which data were obtained and show simple summary data for each intervention group (including, where applicable, the number of events), effect estimates and confidence intervals. These may be tabulated or included on a forest plot.	Appendices 6 and 10 (full data can be provided by the

For peer review only

			first author)
Results of syntheses	21	Present summary effects for each meta-analysis undertaken, including confidence intervals and measures of statistical heterogeneity. State whether the analysis was pre-specified, and report the numbers of studies and participants and, where applicable, the number of events on which it is based.	9-11 – Appendix 15
		When exploring variation in effects due to patient or study characteristics, present summary interaction estimates for each characteristic examined, including confidence intervals and measures of statistical heterogeneity. State whether the analysis was pre-specified. State whether any interaction is consistent across trials.	
		Provide a description of the direction and size of effect in terms meaningful to those who would put findings into practice.	
Risk of bias across studies	22	Present results of any assessment of risk of bias relating to the accumulated body of evidence, including any pertaining to the availability and representativeness of available studies, outcomes or other variables.	9-11 - Appendix 12
Additional analyses	23	Give results of any additional analyses (e.g. sensitivity analyses). If applicable, this should also include any analyses that incorporate aggregate data for studies that do not have IPD. If applicable, summarise the main meta-analysis results following the inclusion or exclusion of studies for which IPD were not available.	9-11 - Appendices 16 and 17
**Discussion**			
Summary of evidence	24	Summarise the main findings, including the strength of evidence for each main outcome.	11-13
Strengths and limitations	25	Discuss any important strengths and limitations of the evidence including the benefits of access to IPD and any limitations arising from IPD that were not available.	13-14
Conclusions	26	Provide a general interpretation of the findings in the context of other evidence.	13-14
Implications	A4	Consider relevance to key groups (such as policy makers, service providers and service users). Consider implications for future research.	12-13

For peer review only

Funding			
Funding	27	Describe sources of funding and other support (such as supply of IPD), and the role in the systematic review of those providing such support.	15

A1 – A3 denote new items that are additional to standard PRISMA items. A4 has been created as a result of re-arranging content of the standard PRISMA statement to suit the way that systematic review IPD meta-analyses are reported.

© Reproduced with permission of the PRISMA IPD Group, which encourages sharing and reuse for non-commercial purposes

For peer review only

BMJ Open

Comparative safety and efficacy of cognitive enhancers for Alzheimer's dementia: A systematic review with individual patient data network meta-analysis

Journal:	*BMJ Open*
Manuscript ID	bmjopen-2021-053012.R3
Article Type:	Original research
Date Submitted by the Author:	17-Mar-2022
Complete List of Authors:	Veroniki, Areti; St. Michael's Hospital, Knowledge Translation Program; Imperial College London, Department of Surgery & Cancer, Faculty of Medicine<br>Ashoor, Huda; St Michael's Hospital, Knowledge Translation Program<br>Rios, Patricia; St Michael's Hospital, Knowledge Translation Program<br>Seitidis, Georgios; University of Ioannina, Department of Primary Education<br>Stewart, Lesley; University of York, Centre for Reviews and Dissemination<br>Clarke, Mike; Queen's University Belfast, Northern Ireland Hub for Trials Methodology Research<br>Tudur-Smith, Catrin; University of Liverpool, Department of Biostatistics<br>Mavridis, Dimitris ; University of Ioannina, Department of Primary Education<br>Hemmelgarn, Brenda; University of Alberta, Department of Medicine<br>Holroyd-Leduc, Jayna; University of Calgary, Department of Medicine<br>Straus, Sharon; St Michael's Hospital, Knowledge Translation Program; University of Toronto, Department of Geriatric Medicine<br>Tricco, Andrea; St Michael's Hospital, Knowledge Translation Program; University of Toronto, Dalla Lana School of Public Health
**Primary Subject Heading**:	Public health
Secondary Subject Heading:	Neurology
Keywords:	STATISTICS & RESEARCH METHODS, EPIDEMIOLOGY, Dementia < NEUROLOGY

SCHOLARONE™
Manuscripts

I, the Submitting Author has the right to grant and does grant on behalf of all authors of the Work (as defined in the below author licence), an exclusive licence and/or a non-exclusive licence for contributions from authors who are: i) UK Crown employees; ii) where BMJ has agreed a CC-BY licence shall apply, and/or iii) in accordance with the terms applicable for US Federal Government officers or employees acting as part of their official duties; on a worldwide, perpetual, irrevocable, royalty-free basis to BMJ Publishing Group Ltd ("BMJ") its licensees and where the relevant Journal is co-owned by BMJ to the co-owners of the Journal, to publish the Work in this journal and any other BMJ products and to exploit all rights, as set out in our licence.

The Submitting Author accepts and understands that any supply made under these terms is made by BMJ to the Submitting Author unless you are acting as an employee on behalf of your employer or a postgraduate student of an affiliated institution which is paying any applicable article publishing charge ("APC") for Open Access articles. Where the Submitting Author wishes to make the Work available on an Open Access basis (and intends to pay the relevant APC), the terms of reuse of such Open Access shall be governed by a Creative Commons licence – details of these licences and which Creative Commons licence will apply to this Work are set out in our licence referred to above.

Other than as permitted in any relevant BMJ Author's Self Archiving Policies, I confirm this Work has not been accepted for publication elsewhere, is not being considered for publication elsewhere and does not duplicate material already published. I confirm all authors consent to publication of this Work and authorise the granting of this licence.

For peer review only

Comparative safety and efficacy of cognitive enhancers for Alzheimer's dementia: A systematic review with individual patient data network meta-analysis

Areti Angeliki Veroniki[1,2,*]	PhD	e-mail: areti-angeliki.veroniki@unityhealth.to
Huda M. Ashoor[1]	BSc	e-mail: huda.ashoor@unityhealth.to
Patricia Rios[1]	MSc	e-mail: patricia.rios@unityhealth.to
Georgios Seitidis[3]	MSc	e-mail: g.seitidis@uoi.gr
Lesley A. Stewart[4]	PhD	e-mail: lesley.stewart@york.ac.uk
Mike Clarke[5]	PhD	e-mail: m.clarke@qub.ac.uk
Catrin Tudur Smith[6]	PhD	e-mail: cat1@liverpool.ac.uk
Dimitris Mavridis[3]	PhD	e-mail: dmavridi@uoi.gr
Brenda R. Hemmelgarn[7]	PhD	e-mail: brenda.hemmelgarn@albertahealthservices.ca
Jayna Holroyd-Leduc[8]	MD	e-mail: jayna.holroyd-leduc@albertahealthservices.ca
Sharon E. Straus[1,9]	MD	e-mail: sharon.straus@utoronto.ca
Andrea C. Tricco[1,10]	PhD	e-mail: andrea.tricco@unityhealth.to

[1] Knowledge Translation Program, Li Ka Shing Knowledge Institute, St. Michael's Hospital, Toronto, Ontario, Canada

[2] Institute of Health Policy Management and Evaluation, University of Toronto, Toronto, Ontario, Canada

[3] Department of Primary Education, School of Education, University of Ioannina, Ioannina, Greece

[4] Centre for Reviews and Dissemination, University of York, York, United Kingdom

[5] Northern Ireland Hub for Trials Methodology Research, Queen's University Belfast, Belfast, United Kingdom

[6] Department of Biostatistics, University of Liverpool, Block F, Waterhouse Building, 1-5 Brownlow Hill, Liverpool, L69 3GL, UK

[7] Department of Medicine, University of Alberta, Edmonton, Alberta, Canada

[8] Department of Medicine, University of Calgary, Calgary, Alberta, Canada

[9] Department of Geriatric Medicine, University of Toronto, Toronto, Ontario, Canada

For peer review only

For peer review only - http://bmjopen.bmj.com/site/about/guidelines.xhtml

[10] Epidemiology Division & Institute of Health Policy, Management, and Evaluation, Dalla Lana School of Public Health, University of Toronto, Toronto, Ontario, Canada

***Corresponding Author**:

Dr. Areti Angeliki Veroniki, MSc, PhD

209 Victoria Street, East Building, Toronto, Ontario

M5B 1T8, Canada

Phone: 416-564-5015; Fax: 416-564-5735;

Email: areti-angeliki.veroniki@unityhealth.to

Word count: 4,415 (max 4000); 1 table; 4 figures; 2 additional files (21 appendices in additional file 1); 32 references

For peer review only

Abstract

Words: 377 (Max 300 words)

Objective: To examine the comparative efficacy and safety of cognitive enhancers by patient characteristics for managing Alzheimer's Dementia (AD).
Design: Systematic review and individual patient data (IPD) network meta-analysis (NMA) based on our previously published systematic review and aggregate data NMA.
Data Sources: MEDLINE, EMBASE, Cochrane Methodology Register, CINAHL, Ageline and Cochrane Central Register of Controlled Trials up to March 2016.
Participants: 80 randomized controlled trials (RCTs) including 21,138 adults with AD, and 12 RCTs with IPD including 6,906 patients.
Interventions: Cognitive enhancers (donepezil, rivastigmine, galantamine and memantine) alone or in any combination against other cognitive enhancers or placebo.
Data extraction and Synthesis: We requested IPD from authors, sponsors and data sharing platforms. When IPD were not available, we used aggregate data. We appraised study quality with the Cochrane risk-of-bias. We conducted a two-stage random-effects IPD-NMA, and assessed their findings using CINeMA (Confidence in Network meta-analysis).
Primary and Secondary Outcomes: We included trials assessing cognition with the Mini-Mental State Examination (MMSE), and adverse events (AEs).
Results: Our IPD-NMA compared 9 treatments (including placebo). Donepezil (mean difference [MD] = 1.41, 95% confidence interval [CI]: 0.51 to 2.32) and donepezil+memantine (MD = 2.57, 95% CI: 0.07 to 5.07) improved MMSE score (56 RCTs, 11,619 participants; CINeMA score: moderate) compared with placebo. According to P-score, oral rivastigmine (odds ratio [OR] = 1.26, 95% CI: 0.82 to 1.94, P-score= 16%) and donepezil (OR = 1.08, 95% CI: 0.87 to 1.35, P-score= 30%) had the least favourable safety profile, but none of the estimated treatment effects were sufficiently precise when compared with placebo (45 RCTs, 15,649 patients; CINeMA score: moderate to high). For moderate to severe impairment, donepezil, memantine and their combination performed best, but for mild to moderate impairment donepezil and transdermal rivastigmine ranked best. Adjusting for MMSE baseline differences, oral rivastigmine and galantamine

For peer review only - http://bmjopen.bmj.com/site/about/guidelines.xhtml

improved MMSE score, whereas when adjusting for comorbidities only oral rivastigmine was effective.

Conclusions: The choice among the different cognitive enhancers may depend on patient's characteristics. The MDs of all cognitive enhancer regimens except for single-agent oral rivastigmine, galantamine, and memantine, against placebo were clinically important for cognition (MD larger than 1.40 MMSE points), but results were quite imprecise. However, two thirds of the published RCTs were associated with high risk of bias for incomplete outcome data, and IPD were only available for 15% of the included RCTs.

Registration: PROSPERO # CRD42015023507

Funding: This research was funded by the CIHR Drug Safety and Effectiveness Network (grant number 137713).

Keywords: network meta-analysis; multiple treatments meta-analysis; individual participant data; Nootropic Agents; Alzheimer Disease

Strengths and limitations of this study

- This is one of the most comprehensive systematic reviews and network meta-analysis of cognitive enhancers including individual patient data for Alzheimer's Dementia to produce treatment recommendations by patient characteristics.
- We followed the methodologically rigorous guidelines in the Cochrane Handbook for systematic reviews, and the CINeMA quality assessment guidelines.
- Access to individual patient data allowed us to 1) observe minor differences between the original published results and our re-analysis, potentially due to differences in imputation methods for missing data or because original studies have excluded some patients, and hence have used a smaller sample size, 2) overcome potential reporting bias, and 3) assess for potential effect modifiers that were not reported in the original publications (e.g., comorbidities, additional medications) and explore for treatment-by-covariate interactions on the patient-level.
- Two thirds of the included RCTs, were associated with high risk of bias for incomplete outcome data due to attrition.
- We were unable to include individual patient data for all RCTs (only 15% of the studies shared their individual patient data), highlighting potential availability bias.

For peer review only

For peer review only - http://bmjopen.bmj.com/site/about/guidelines.xhtml

- Our literature searches were conducted 5 years ago and additional relevant studies may be available. However, obtaining IPD in a timely manner was very challenging and required more time than anticipated. Similar to all systematic reviews, the evidence should be updated regularly.

For peer review only

For peer review only - http://bmjopen.bmj.com/site/about/guidelines.xhtml

Introduction

Alzheimer's dementia (AD) is the most common type of dementia.[1] Patients living with AD have a lower quality of life due to deterioration in function, cognition, behavior, and mental health over time, as well as increased mortality.[2] Pharmacological treatment for AD predominantly consists of cholinesterase inhibitors (donepezil, galantamine, rivastigmine) and the N-methyl-d-aspartate (NMDA) receptor antagonist, memantine. All three cholinesterase inhibitors and memantine are currently the only effective licensed treatments for dementia,[3] but their clinical effect can be small and there is no convincing evidence that they modify the disease process in AD.[4] Also, it is unclear whether galantamine, rivastigmine, or donepezil should be used by patients with severe AD, or whether memantine is the optimal treatment for severe AD.[5]

In AD, disease severity and sex are potential effect modifiers. However, aggregate data and covariates of interest (e.g., sex, disease severity) are not consistently reported across randomized clinical trials (RCTs).[6] The use of IPD has several advantages, such as it allows for the exploration of the relationship between treatment effects and patient-level characteristics, and it overcomes restrictions in using the information reported in the publication among others. The aim of this study was to examine the comparative efficacy and safety of cognitive enhancers for patients with different characteristics, such as severities of AD and for females versus males through a systematic review and individual patient data (IPD) NMA. This systematic review was based on our previously published systematic review and aggregate data NMA.[6] NMA is an extension of standard meta-analysis synthesizing different sources of evidence from a network of RCTs comparing different treatments within a single model. NMA can provide treatment effect estimates for treatment comparisons that have not studied in a head-to-head study.

Methods

We reported our results according to the Preferred Items for Systematic Reviews and Meta-analysis (PRISMA) Statement for NMA and PRISMA-IPD.[7,8]

For peer review only - http://bmjopen.bmj.com/site/about/guidelines.xhtml

Protocol

The research question and protocol were based on our previous systematic review and NMA.[6] We registered our systematic review protocol with the prospective register of systematic reviews (PROSPERO: CRD42015023507), and published our protocol.[9] Additional information is also provided in Additional File 1: Appendix 1 and Additional File 2. Herein, we briefly summarize our methods.

Eligibility criteria

We updated our previous systematic review,[6] using similar population, interventions, comparators, study designs and time period (PICOST) criteria. The literature search was updated from January 2015 to March 2016. We included published and English RCTs that assessed cognition via the Mini-Mental State Examination (MMSE; efficacy and primary outcome) and/or adverse events (AE; safety outcome) in adults with Alzheimer's dementia.

IPD collection process

We contacted the corresponding author followed by the next-in-order author, as presented in each eligible RCT, to obtain IPD. The author contact process was part of a RCT that our team conducted to assess methods that may optimize response rates for IPD retrieval.[10] We also contacted sponsors of eligible trials, as reported in the publications. We contacted industry sponsors only, as we were not able to locate contact information for the majority of non-industry sponsors (e.g., grants and university funding). If a study had multiple sponsors, we contacted all of them. To further facilitate IPD access, we contacted the Clinical Study Data Request (CSDR)[11] and Yale University Open Data Access (YODA) data sharing platforms.[12] If a data provider was unable to provide IPD we noted the reason.

Risk of bias and quality appraisal

For peer review only - http://bmjopen.bmj.com/site/about/guidelines.xhtml

We appraised study quality using the Cochrane risk of bias tool.[13] To ensure data consistency[8] we compared IPD with aggregate data reported in the publication. We assessed whether randomization of patients was adequate (i.e., intervention and comparison groups were balanced for important patient characteristics), by comparing numbers and types of patients in each arm.

When at least 10 studies were available for each treatment against placebo, publication bias and small-study effects were examined visually using the comparison adjusted funnel plot under the fixed-effect model.[3] When a funnel plot asymmetry was detected, we performed the Copas selection for the treatment comparisons that were informed by at least 10 studies and for which asymmetry was evident in the funnel plot. We explored the possibility that this was due to publication bias,[14] and made moderate assumptions about the probability of publication of the smaller and larger (in terms of standard error) studies. We assumed that the smallest study had a probability of publication equal to 40-50% and the largest study had a probability of 80-90%. Confidence in NMA findings was assessed for each outcome using CINeMA (Confidence in Network meta-analysis, see Additional File 1: Appendix 1 for more details).[15]

Synthesis

We performed a descriptive analysis using frequencies and distributions of the characteristics of the included patients and treatments. For each outcome, we present the network geometry according to IPD availability. We conducted a two-stage IPD analysis, whereby data were analysed separately in each trial in the first stage and the trial parameter estimates were synthesised in a random-effects meta-analysis or NMA in the second stage.

The summary treatment effects are presented using the odds ratio (OR) or mean difference (MD) along with their corresponding CIs and prediction intervals (PIs).[16] We ranked the interventions for each outcome using the P-scores (and SUCRAs [surface under the cumulative ranking curve] in meta-regression analysis), and present them in a rank-heat plot.[17,18]

Patient and public involvement

Not applicable.

For peer review only - http://bmjopen.bmj.com/site/about/guidelines.xhtml

Results

Literature search, study selection and IPD obtained

After screening 20,410 titles and abstracts and 1,968 full-text articles, 96 studies fulfilled the eligibility criteria; 80 unique studies and 16 companion reports (Figure 1a, Additional File 1: Appendix 2).

(Figure 1 here)

Of the 80 RCTs, 55 reported at least one industry-sponsored funder (i.e. 40 studies reported a single industry-sponsor and 15 multiple industry-sponsors). In the remaining studies, 9 were publicly-sponsored and 16 did not report any information about funding. We requested IPD by contacting the corresponding authors for 80 RCTs that included 21,138 participants. None of the original authors shared their IPD. Fifteen commercial sponsors were then contacted and 6 (40%) sponsors shared their data through proprietary sponsor-specific platforms. The 6 sponsors were contacted for 46 RCTs (14,580 participants), and we obtained IPD for 30% (14 RCTs, 8,007 participants) of these RCTs (1,058 total waiting days up to March 9, 2020). The study flow for obtaining IPD is depicted in Figure 1b.

We were able to include 12 (6,906 patients) of 14 RCTs in our NMA due to incompleteness of provided IPD (Additional File 1: Appendix 3). The number of studies with available/non-available IPD from each data provider along with reasons for non-availability of IPD are presented in Additional File 1: Appendix 4.

Study and patient characteristics

Most included studies (33%) were multi-national. The mean age of patients ranged from 61 to 86 years. The majority of the RCTs included patients with mild-moderate AD (56%), although the diagnostic criteria used for AD varied widely (Table 1). The most frequent longest duration of follow-up was 24 weeks (24 RCTs, 30%; Additional File 1: Appendix 5). Important patient characteristics, such as percent of male and dropout rates, were not balanced across groups in the

For peer review only - http://bmjopen.bmj.com/site/about/guidelines.xhtml

RCTs with provided IPD (Additional File 1: Appendix 6). Comparing study and patient characteristics of available and non-available IPD when a study was industry-sponsored, we found differences in the year of study publication, study size, and absolute mean difference (Additional File 1: Appendix 7).

(Table 1 here)

Risk of bias and IPD integrity

Using the Cochrane risk-of-bias tool, allocation concealment was at low risk of bias for 43% and blinding of participants and personnel was low for 64% of the RCTs (Additional File 1: Appendix 8). One third of the RCTs had low risk of incomplete outcome data bias due to attrition and almost two thirds had high potential risk of "other" bias, specifically, funding bias. The other risk of bias item was scored as unclear for 32%. Overall risk of bias was comparable in studies with available and unavailable IPD (Additional File 1: Appendix 9).

All IPD provided were checked for consistency and results from published RCTs were reproduced and provided in Additional File 1: Appendix 10. High dropout rates were observed in the IPD; experiencing an adverse event was the most common reason for dropout. Despite the high dropout rates observed in the individual studies, there was no indication of correlation between age and dropout (Additional File 1: Appendix 11). Comparison-adjusted funnel plot for MMSE suggested there is indication for small-study effects (see Additional File 1: Appendix 12). In contrast to the standard meta-analysis (MD=1·65 95% CI (0·16, 3·14)), the Copas selection model estimated a pooled treatment effect for donepezil vs. placebo MD=1·87 95% CI (1·55, 2·20) with between-study variance τ^2= 1·95, and correlation coefficient -0·45 (-0·76, -0·01) reflecting the belief that the propensity for publication was associated with the observed effect size.

Network meta-analysis

For peer review only - http://bmjopen.bmj.com/site/about/guidelines.xhtml

In both MMSE and AE outcomes, on average there were no important concerns regarding the transitivity and consistency assumptions (Additional File 1: Appendices 13 and 14; design-by-treatment interaction model MMSE: χ^2= 4·36, 13 degrees of freedom (df), p-value= 0·987; AE: χ^2= 3·57, 6 df, p-value= 0·735). Below we present the main analysis results compared to placebo. Additional analyses are presented in Additional File 1: Appendix 15-16). The network geometry is presented in Figure 2.

(Figure 2 here)

Cognition

The NMA for MMSE included 56 RCTs, 9 treatments (including placebo), and 11,619 participants. Nine RCTs (3,625 patients) contributed IPD and 47 RCTs (7,994 patients) contributed aggregated data to the NMA. Two studies[19,20] did not report MMSE in the final publication, but in the retrieved IPD we were able to use data for this outcome.

NMA of studies with IPD and aggregate data

Studies in this NMA compared all available treatments. Donepezil (MD= 1·41, 95% CI: 0·51 to 2·32) and donepezil+memantine (MD= 2·57, 95% CI: 0·07 to 5·07) were superior to placebo in terms of MMSE score (Additional File 1: Appendix 15). Transdermal rivastigmine (MD= 2·11, 95% CI: -0·04 to 4·26), and the combinations donepezil+memantine, galantamine+memantine (MD= 2·24, 95% CI: -2·13 to 6·61), and transdermal rivastigmine+memantine (MD= 1·79, 95% CI: -1·70 to 5·27) were associated with a MD from placebo of more than 1.40 MMSE points. A previous study suggested a MD larger than 1·40 is a minimal clinically important difference (MCID).[21] However, the associated 95% CIs were quite imprecise spanning between a mean decrease below and a mean increase above the suggested MCID value (Figure 3a). However, donepezil+memantine had the highest likelihood of being the most effective in improving MMSE score (P-score range 79-80%, Figure 4). Confidence in NMA results was moderate (Additional File 1: Appendix 17).

(Figure 3 here)

(Figure 4 here)

For peer review only - http://bmjopen.bmj.com/site/about/guidelines.xhtml

NMA of studies with aggregate data

Studies in this NMA compared all available treatments. Donepezil improved MMSE score significantly (MD= 1·55 95% CI: 0·41 to 2·68). Assuming an MCID of 1·40, results were in agreement with the NMA of IPD and aggregate data, and donepezil+memantine (MD= 2·71, 95% CI: -0·17 to 5·60) was likely the most effective in improving MMSE score (P-score= 76%).

NMA of studies with IPD

Studies in this NMA compared placebo, donepezil, oral rivastigmine, transdermal rivastigmine, galantamine, and memantine. Donepezil (MD= 0·70, 95% CI: 0·01 to 1·40) and transdermal rivastigmine (MD= 1·06, 95% CI: 0·04 to 2·08) were superior to placebo, but none of the point estimates reached a previously suggested MCID.[21] The most effective treatment was likely transdermal rivastigmine (P-score= 82%).

Additional analyses using IPD and aggregate data

Overall, additional analyses using both IPD and aggregate data were in agreement with the findings of the main analysis (Additional File 1: Appendix 16). Cognitive performance was better in patients with mild to moderate MMSE receiving donepezil (MD= 1·68 95% CI: 0·31 to 3·06, P-score= 69%) and most likely when receiving transdermal rivastigmine (MD= 2·74 95% CI: -0·68 to 6·16, P-score= 81%). In patients with moderate to severe MMSE the combination donepezil+memantine improved MMSE score significantly (MD= 2·49 95% CI: 1·55 to 3·44, P-score=100%), but oral rivastigmine deteriorated MMSE score significantly (MD= -1·00 95% CI: -1·87 to -0·12, P-score= 4%). Donepezil (MD= 1·31 95% CI: 0·66 to 1·96, P-score= 78%) and memantine (MD=0·69 95% CI: 0·07 to 1·31, P-score= 59%) also performed well for patients with moderate to severe cognitive impairment.

Accounting for the impact of the outlier studies, galantamine+memantine was the second-best cognitive enhancer (MD= 1·87 95% CI: 0·08 to 3·66, P-score=82%) after donepezil+memantine (MD= 2·04 95% CI: 1·03 to 3·05, P-score= 92%). Using only IPD adjusted for comorbidities

For peer review only - http://bmjopen.bmj.com/site/about/guidelines.xhtml

suggested that oral rivastigmine improves MMSE score (MD= 0·88 95% CI: 0·31 to 1·45, P-score= 75%). Similarly, using IPD adjusted for cognitive impairment assessed with MMSE at baseline suggested that oral rivastigmine (MD= 0·88 95% CI: 0·31 to 1·45, P-score= 69%) and galantamine (MD= 0·76 95% CI: 0·34 to 1·18, P-score= 62%) improve MMSE score, but in a future study, results are only stable for galantamine.

Heterogeneity in NMA was high (between-study variance = 5·75, I^2= 96%) compared also to the Rhodes et *al.*[22] empirical distribution (median 0.05, 95% range: 0·00 to 7·56). However, heterogeneity decreased importantly when excluding outliers (between-study variance = 0·59, I^2= 73%), including only patients with moderate to severe AD (between-study variance = 0·18, I2= 44%), restricting to industry-sponsored trials (between-study variance = 0·16, I^2= 43%), and using IPD only (between-study variance = 0·12, I^2= 29%).

Adverse events

A NMA was conducted on adverse events (study definitions are provided in Additional File 1: Appendix 18) with 45 RCTs, 9 treatments (including placebo), and 15,649 patients (Figure 2b). In particular, 12 RCTs (6420 patients) contributed to the NMA using their IPD and 33 RCTs (9229 patients) using their data on their aggregated form. The time taken to achieve at least one AE was available in 8 studies with available IPD and ranged between 45 and 2228 days (Additional File 1: Appendix 19). Only one study included a patient with a AE occurring earlier than the trial opening and was excluded from the study.[23]

NMA of studies with IPD and aggregate data

Studies in this NMA compared all available treatments. According to P-score, oral rivastigmine had the least favourable safety profile regarding AE (OR= 1·26, 95% CI: 0·82 to 1·94, P-score= 16%), followed by donepezil (OR= 1·08, 95% CI: 0·87 to 1·35, P-score= 30%) and galantamine+memantine (OR= 1·03, 95% CI: 0·45 to 2·39, P-score= 43%), yet in these comparisons the odds of experiencing an AE were imprecise and not importantly different from placebo (Figure 3b; Additional File 1: Appendices 16, 20). Confidence in NMA results ranged between moderate and high (Additional File 1: Appendix 17).

For peer review only - http://bmjopen.bmj.com/site/about/guidelines.xhtml

NMA of studies with aggregate data

Studies in this NMA compared all available treatments. Results were mainly consistent with NMA of IPD and aggregate data, but memantine was 0·70 times less likely to experience an AE than placebo, with an OR ranging from 0·51 to 0·97 (P-score= 77%).

NMA of studies with IPD

Studies in this NMA compared placebo, donepezil, oral rivastigmine, transdermal rivastigmine, galantamine, and memantine. Results were on average consistent with NMA of IPD and aggregate data.

Additional analyses using IPD and aggregate data

Additional analyses using both IPD and aggregate data, showed that memantine was 0·61 times less likely to experience an AE than placebo when using study duration as a covariate, with an OR ranging from 0·37 to 0·93 (P-score= 88%). Restricting to low risk of bias for incomplete outcome data, galantamine was associated with significantly lower odds of a AE (OR= 0·69, 95% CI: 0·50 to 0·97, P-score= 80%).

Heterogeneity in NMA was low (between-study variance = 0·04, I^2= 22%) compared to the Turner et *al.*[24] empirical distribution (median 0·12, 95% range: 0·01 to 2·63). Heterogeneity decreased importantly when restricting to aggregate data (between-study variance = 0·00, I^2= 0%), low risk of bias for incomplete outcome data (between-study variance = 0·02, I^2= 10%), patients with moderate to severe cognitive impairment (between-study variance = 0·00, I^2= 0%), and when adjusting for study duration (between-study variance = 0·03), year of publication (between-study variance = 0·02), mean age (between-study variance = 0.02) or sex (between-study variance = 0·03).

Discussion

For peer review only - http://bmjopen.bmj.com/site/about/guidelines.xhtml

We compared the efficacy and safety of cognitive enhancers regarding MMSE and AE outcomes to update our previous systematic review[6] and included studies with both aggregate data and IPD. Our results are in agreement with our previous systematic review,[6] and show that donepezil+memantine, donepezil alone and transdermal rivastigmine were the most effective treatments for improving MMSE score. However, heterogeneity was a major concern, which requires careful consideration before suggesting the use of cognitive enhancers, and particularly when the efficacy is not clear on the patient's characteristics. This was also captured by PIs, but their interpretation requires caution due to evidence of funnel plot asymmetry in the MMSE outcome. Overall, PIs are expected to include the true intervention effect expected in future studies, and they incorporate an extra component of variance, specifically between-study heterogeneity. In the absence of heterogeneity, confidence intervals and PIs are equal. According to the P-score intervention ranking, both donepezil+memantine and transdermal rivastigmine had a favourable safety profile regarding AE, whereas the therapy with the least favourable profile was oral rivastigmine followed by donepezil. However, none of the estimated treatment effects were sufficiently precise when cognitive enhancers were compared with the placebo group. CINeMA suggested that within-study bias and reporting bias were the highest concerns for the MMSE outcome, whereas within-study bias and imprecision of effect estimates were the highest concerns for the AE outcome.

For peer review only

Overall, the choice among the different cognitive enhancers may depend on the patient's characteristics. In participants with moderate to severe cognitive impairment (defined by MMSE), a larger improvement in cognitive performance was observed for donepezil and memantine, and their combination (donepezil+memantine), and these efficacy-related results are expected to also be reflected when a future study becomes available. The least effective cognitive enhancer in participants with moderate to severe cognitive impairment was oral rivastigmine. For patients with mild to moderate impairments based on MMSE scores, donepezil and transdermal rivastigmine were most likely the best performing cognitive enhancers. For patients with moderate to severe cognitive impairment, cognitive enhancers were well tolerated. For patients with mild to moderate cognitive impairment, all except for memantine and its combination with transdermal rivastigmine, were associated with increased odds of an AE, yet none of these results reached statistical significance. Overall, memantine was associated with

For peer review only - http://bmjopen.bmj.com/site/about/guidelines.xhtml

lower odds of an AE than placebo, yet this was statistically significant only in the subnetwork analysis including aggregate data (i.e., studies without IPD) and the meta-regression analysis using study duration as a covariate. However, acknowledging for heterogeneity in the network, PIs suggested that results are inconclusive and the odds of AE could not be differentiated between memantine and placebo. Of note, the accuracy of AE reporting may be impacted by the degree of cognitive impairment. Using IPD only and adjusting for MMSE baseline differences, (as shown in Additional File 1: Appendix 16, Mean Difference: NMA of studies with IPD adjusted for baseline cognitive impairment), oral rivastigmine and galantamine improved MMSE score, whereas when adjusting for comorbidities only oral rivastigmine was effective, but results can change in a future study. Considering a MCID equal to 1·40 points,[21] the MDs of all cognitive enhancer regimens except for single-agent oral rivastigmine, galantamine, and memantine, against placebo were clinically important for cognition, but these were associated with high uncertainty. However, the 1·40 MMSE cut-off value is not a widely adopted MCID. Also, high variability may be related to different populations included in the studies, such as genetic profiles, race, and gender identity. Future studies should report this information to enable exploration of population characteristics that would benefit more, with a clinically important improvement, when using these treatments. Our results did not differ by participant characteristics sex, age, and other medications, or by study characteristics, study duration and year of publication. However, these findings might be due to low power since meta-regression analyses depend on the number and size of studies, magnitude of the relationship between the covariate and effect size, along with its precision and heterogeneity.[25]

To the best of our knowledge, our study was the first to add IPD in a NMA of cognitive enhancers for patients with Alzheimer's Dementia to produce treatment recommendations by patient characteristics. We followed the methods guidelines in the Cochrane Handbook for systematic reviews,[26] the reporting guidelines in the PRISMA-NMA and PRISMA-IPD statements,[7,8] and the CINeMA quality assessment guidelines.[15] Compared to previous systematic reviews, we included a larger number of studies and/or studies with shared IPD, compared in a wider range of cognitive enhancers.[6,27] Our results are in agreement with previous studies overall. Access to IPD allowed us to observe minor differences between the original published results and our re-analysis. An explanation in these differences may be that many

For peer review only - http://bmjopen.bmj.com/site/about/guidelines.xhtml

studies used the last-observation-carried-forward imputation method, whereas we used the available case analysis when assessing MMSE. Another potential explanation might be that original studies excluded some patients, and hence used a smaller sample size.

Comparing NMA, results between aggregate data and IPD were in agreement. The only difference was observed in transdermal rivastigmine that was associated with a MCID of greater than 1·40 MMSE points against placebo in the aggregate data NMA compared to the IPD NMA, yet a statistically significant improvement was achieved in the IPD NMA. The inclusion of IPD in our NMA, allowed us to overcome potential reporting bias and to include IPD for 1) a study that we previously were unable to include since arm-level data were not reported in the RCT publication,[23] and 2) two studies that did not report MMSE results in their publications.[19,20] The use of IPD also allowed us to assess for potential effect modifiers that were not reported in the original publications (e.g., comorbidities, additional medications) and explore for treatment-by-covariate interactions on the patient-level. Several challenges were encountered during the IPD request from sponsors, showing that repositories are not a panacea (Additional File 1: Appendix 21).

An important finding of our review is that the two thirds of the published RCTs, were associated with high risk of bias for incomplete outcome data due to attrition, and the majority of these RCTs used the last-observation-carried-forward technique for missing data. This approach may bias results favouring cognitive enhancers, since the dropout rates were greater in the treatment group compared to the placebo group in 63% of the included studies and because dementia is a progressive disease. Of the 27 studies comparing treatment against placebo and reporting the number of dropouts, 17 studies had a greater dropout rate in the treatment group (treatment group: median dropout rate= 28% IQR [17% to 39%]; placebo group: median dropout rate= 21% IQR [15% to 31%]). Last-observation-carried-forward is an inappropriate imputation method for Alzheimer's Dementia studies, since it ignores expected deterioration of the patient's condition and stabilizes the outcome at the value observed at the time of dropout (i.e., the last observation).[28] Restricting to low risk of attrition bias studies, we found that galantamine was significantly associated with decreased odds of experiencing an AE.

For peer review only - http://bmjopen.bmj.com/site/about/guidelines.xhtml

Our study has limitations worth mentioning. First, we were unable to include IPD for all eligible studies (only 15% of the included RCTs shared their IPD), highlighting potential availability bias for IPD. However, recent simulations have shown that combining IPD and aggregate data in a NMA can significantly improve precision, reduce bias, and increase information compared to NMA relying on aggregated data alone.[29] Second, missing data is a big concern in the published RCTs for AD. We found high rates of dropouts from experiencing an adverse event and the patients' characteristics that may increase the chances of such adverse reactions prior to administering these cognitive enhancers should further be explored. To assess the impact of missing data in our NMA, we applied the informative missingness of difference in means.[30] However, future studies should explore the characteristics of missing participants and specific adverse events. Third, the lack of studies in certain treatment comparisons may have affected the P-score calculation and treatment ranking. In particular, polytherapies were informed by maximum two studies, and ranking may have been in favour of the complex intervention group with the smaller number of studies.[31] For example, in MMSE the polytherapies including memantine in conjunction with one of the three treatments donepezil, galantamine, transdermal rivastigmine had a P-score ≥60%, but these all had wide 95% CIs for MD. As such, ranking should be interpreted with caution and along with the estimated effect sizes and their uncertainty measures. Fourth, the comparison-adjusted funnel plot for MMSE suggested there is an indication for small-study effects pointing to the treatment being better, and results should be interpreted with caution. This may also be related to the potential risk of funding bias, since the majority of the included studies were industry-sponsored and IPD were retrieved only from industry-sponsored studies favouring cognitive enhancers over placebo. Overall, MMSE score is only a surrogate maker for determining the impact of treatments on dementia. A full assessment that considers the potential impact of treatments on cognition, function and behavioural symptoms needs to be considered within the clinical context. Fifth, differences in patient characteristics, such as sex, were observed in the RCTs with provided IPD, which increased heterogeneity across studies. To account for these differences, we used the fully adjusted treatment effect estimates in the IPD analyses and the primary NMA analysis. Also, at the NMA level, we found that on average there were no important differences across treatment comparisons to threaten the transitivity assumption. Sixth, there are clinically important limitations associated with this review, including consistent definition of outcome measures

For peer review only - http://bmjopen.bmj.com/site/about/guidelines.xhtml

across studies, a well-established MCID for the MMSE score, lack of consideration of drug doses due to inconsistent reporting and data availability bias that we were unable to overcome (15% of the studies shared their IPD). Future studies are needed to establish ranking efficacy in drug doses and combination of interventions across different disease severity categories. Seventh, the literature searches were conducted 5 years ago and additional relevant studies may be available. However, obtaining IPD in a timely manner was very challenging and required more time than anticipated (challenges to obtain IPD are outlined in Additional File 1: Appendix 21). Similar to all systematic reviews, the evidence should be updated regularly.[32]

We expect that our findings will increase scientific knowledge, because people with Alzheimer's Dementia require personalized medicine to optimize their healthcare. Well-conducted meta-analyses of IPD are considered the 'gold-standard' and influence patient care since patient-level data can be provided to facilitate tailored decision making. However, results from meta-analyses of IPD are likely subject to retrieval bias and awareness of these limitations and their potential impact on findings is required.

For peer review only

Contributors

AAV, SES and ACT conceived and designed the study.
AAV conducted the analyses, abstracted data, contacted sponsors, analysed data, interpreted results, appraised quality of results, and wrote a draft manuscript.
GS conducted the analyses, appraised quality of results, and edited the manuscript.
HMA coordinated the review, screened citations and full-text articles, abstracted data, appraised quality, cleaned the data, contacted sponsors, and edited the manuscript.
PR helped coordinate the study, screened citations and full-text articles, extracted and categorized data, appraised quality, and edited the manuscript.
SES and ACT interpreted results and edited the manuscript.
ACT and HMA contacted authors. LAS, MC, CTS, DM, BRH, JHL provided input into the design, interpreted results, and edited the manuscript.
All authors read and approved the final manuscript.

Declaration of interests

The authors declare that they have no competing interests.

Data sharing statement

All data relevant to the study are included in the article or uploaded as supplementary information.

Funding

This research was funded by the CIHR Drug Safety and Effectiveness Network (grant number 137713). The funder contributed to defining the scope of the systematic review but otherwise had no role in study design, data collection, analysis, and interpretation of data. Data sharing sponsors were provided the ability to review at the time of submission of the manuscript for publication.

For peer review only

AAV was previously funded by the Canadian Institutes of Health Research (CIHR) Banting Postdoctoral Fellowship Program [No. 139157]. GS and DM were funded from the European Union's Horizon 2020 [No. 754936]. SES is funded by a Tier 1 Canada Research Chair in Knowledge Translation [No. N/A]. ACT is funded by a Tier 2 Canada Research Chair in Knowledge Synthesis [No. N/A].

Open Access

This is an open access article distributed in accordance with the Creative Commons Attribution Non Commercial (CC BY-NC 4.0) license, which permits others to distribute, remix, adapt, build upon this work non-commercially, and license their derivative works on different terms, provided the original work is properly cited, appropriate credit is given, any changes made indicated, and the use is non-commercial. See: http:// creativecommons. org/ licenses/ by- nc/ 4. 0/.

Acknowledgements

We thank Laure Perrier for conducting the initial literature search, Alissa Epworth for updating it, and Becky Skidmore for peer reviewing the literature search. We thank Robert Peterson for his support on this study as a knowledge user. We would also like to thank Paul A. Khan, Fatemeh Yazdi, Marco Ghassemi, John D. Ivory, Charlene Soobiah, Erik Blondal, Joanne M. Ho, Shirra Berliner, and Carmen H. Ng for screening some of the citations, data abstracting included studies, or both. We thank Susan Le for helping with contacting the authors and sponsors of the included studies in this review. Finally, we thank Susan Le and Shazia Siddiqui for formatting the manuscript and assisting with the submission.

We would also like to thank the following sponsors for sharing the data with us:

- "This publication is based on research using data from data contributors, AbbVie, Inc, that has been made available through Vivli, Inc. Vivli has not contributed to or approved, and is not in any way responsible for, the contents of this publication."

For peer review only - http://bmjopen.bmj.com/site/about/guidelines.xhtml

- "This study, carried out under YODA Project #2017-1671, used data obtained from the Yale University Open Data Access Project, which has an agreement with JANSSEN RESEARCH & DEVELOPMENT, L.L.C.. The interpretation and reporting of research using this data are solely the responsibility of the authors and does not necessarily represent the official views of the Yale University Open Data Access Project or JANSSEN RESEARCH & DEVELOPMENT, L.L.C.."
- This publication used data obtained from Eisai, GlaxoSmithKline, and Novartis carried under www.ClinicalStudyDataRequest.com
- This publication used data obtained from Lundbeck

Ethical Approval Statement

Not applicable.

For peer review only - http://bmjopen.bmj.com/site/about/guidelines.xhtml

References

1. Health NCCfM. Dementia: A NICE-SCIE guideline on supporting people with dementia and their carers in health and social care. 2007: British Psychological Society; 2007.
2. Dudgeon S. Rising Tide: The Impact of Dementia on Canadian Society: a Study. 2010: Alzheimer Society of Canada; 2010.
3. O'Brien JT, Holmes C, Jones M, et al. Clinical practice with anti-dementia drugs: A revised (third) consensus statement from the British Association for Psychopharmacology. *Journal of psychopharmacology (Oxford, England)* 2017; **31**(2): 147-68.
4. National Institute for Health and Care Excellence (UK). Dementia: Assessment, management and support for people living with dementia and their carers. 2018. Available from: https://www.ncbi.nlm.nih.gov/books/NBK536484/.
5. National Institute for Health and Clinical Excellence. Donepezil, galantamine, rivastigmine and memantine for the treatment of Alzheimer's disease. London, UK, 2011.
6. Tricco AC, Ashoor HM, Soobiah C, et al. Comparative Effectiveness and Safety of Cognitive Enhancers for Treating Alzheimer's Disease: Systematic Review and Network Metaanalysis. *J Am Geriatr Soc* 2018; **66**(1): 170-8.
7. Hutton B, Salanti G, Caldwell DM, et al. The PRISMA extension statement for reporting of systematic reviews incorporating network meta-analyses of health care interventions: checklist and explanations. *Annals of internal medicine* 2015; **162**(11): 777-84.
8. Stewart LA, Clarke M, Rovers M, et al. Preferred Reporting Items for Systematic Review and Meta-Analyses of individual participant data: the PRISMA-IPD Statement. *Jama* 2015; **313**(16): 1657-65.
9. Veroniki AA, Straus SE, Ashoor HM, et al. Comparative safety and effectiveness of cognitive enhancers for Alzheimer's dementia: protocol for a systematic review and individual patient data network meta-analysis. *BMJ open* 2016; **6**(1): e010251.
10. Veroniki AA, Ashoor HM, Le SPC, et al. Retrieval of individual patient data depended on study characteristics: a randomized controlled trial. *Journal of clinical epidemiology* 2019; **113**: 176-88.
11. So D, Knoppers BM. Ethics approval in applications for open-access clinical trial data: An analysis of researcher statements to clinicalstudydatarequest.com. *PLoS One* 2017; **12**(9): e0184491.
12. Krumholz HM, Waldstreicher J. The Yale Open Data Access (YODA) Project--A Mechanism for Data Sharing. *N Engl J Med* 2016; **375**(5): 403-5.
13. Higgins JP, Altman DG, Gotzsche PC, et al. The Cochrane Collaboration's tool for assessing risk of bias in randomised trials. *Bmj* 2011; **343**: d5928.
14. Mavridis D, Sutton A, Cipriani A, Salanti G. A fully Bayesian application of the Copas selection model for publication bias extended to network meta-analysis. *Statistics in medicine* 2013; **32**(1): 51-66.
15. CINeMA: Confidence in Network Meta-Analysis. Institute of Social and Preventive Medicine, University of Bern. *Available from cinemaispmch* 2017.
16. Riley RD, Higgins JP, Deeks JJ. Interpretation of random effects meta-analyses. *Bmj* 2011; **342**: d549.
17. Veroniki AA, Straus SE, Fyraridis A, Tricco AC. The rank-heat plot is a novel way to present the results from a network meta-analysis including multiple outcomes. *Journal of clinical epidemiology* 2016; **76**: 193-9.
18. Rucker G, Schwarzer G. Ranking treatments in frequentist network meta-analysis works without resampling methods. *BMC Med Res Methodol* 2015; **15**: 58.
19. Herrmann N, Gauthier S, Boneva N, Lemming OM. A randomized, double-blind, placebo-controlled trial of memantine in a behaviorally enriched sample of patients with moderate-to-severe Alzheimer's disease. *International psychogeriatrics* 2013; **25**(6): 919-27.

For peer review only - http://bmjopen.bmj.com/site/about/guidelines.xhtml

20. Burns A, Bernabei R, Bullock R, et al. Safety and efficacy of galantamine (Reminyl) in severe Alzheimer's disease (the SERAD study): a randomised, placebo-controlled, double-blind trial. *The Lancet Neurology* 2009; **8**(1): 39-47.

21. Howard R, Phillips P, Johnson T, et al. Determining the minimum clinically important differences for outcomes in the DOMINO trial. *Int J Geriatr Psychiatry* 2011; **26**(8): 812-7.

22. Rhodes KM, Turner RM, Higgins JP. Predictive distributions were developed for the extent of heterogeneity in meta-analyses of continuous outcome data. *Journal of clinical epidemiology* 2015; **68**(1): 52-60.

23. Gold M, Alderton C, Zvartau-Hind M, et al. Rosiglitazone monotherapy in mild-to-moderate Alzheimer's disease: results from a randomized, double-blind, placebo-controlled phase III study. *Dementia and geriatric cognitive disorders* 2010; **30**(2): 131-46.

24. Turner RM, Davey J, Clarke MJ, Thompson SG, Higgins JP. Predicting the extent of heterogeneity in meta-analysis, using empirical data from the Cochrane Database of Systematic Reviews. *International journal of epidemiology* 2012; **41**(3): 818-27.

25. Hedges L, Higgins J, Rothstein H. Introduction to Meta-Analysis. John Wiley & Sons; 2009.

26. Higgins JP, Thomas J, Chandler J, et al. Cochrane handbook for systematic reviews of interventions: John Wiley & Sons; 2019.

27. Cui CC, Sun Y, Wang XY, Zhang Y, Xing Y. The effect of anti-dementia drugs on Alzheimer disease-induced cognitive impairment: A network meta-analysis. *Medicine* 2019; **98**(27): e16091.

28. Molnar FJ, Hutton B, Fergusson D. Does analysis using "last observation carried forward" introduce bias in dementia research? *CMAJ* 2008; **179**(8): 751-3.

29. Jansen JP. Network meta-analysis of individual and aggregate level data. *Res Synth Methods* 2012; **3**(2): 14.

30. Mavridis D, White IR. Dealing with missing outcome data in meta-analysis. *Res Synth Methods* 2020; **11**(1): 2-13.

31. Kibret T, Richer D, Beyene J. Bias in identification of the best treatment in a Bayesian network meta-analysis for binary outcome: a simulation study. *Clinical epidemiology* 2014; **6**: 451-60.

32. Sampson M, Shojania KG, McGowan J, et al. Surveillance search techniques identified the need to update systematic reviews. *J Clin Epidemiol* 2008; **61**(8): 755-62.

For peer review only

For peer review only - http://bmjopen.bmj.com/site/about/guidelines.xhtml

Figure Captions

Figure 1. Flow diagram for study inclusion in the review (a) and studies retrieved with individual patient data (b).

Figure 2. Network diagrams for (a) MMSE and (b) AE outcomes. The size of each node and line indicates the number of studies included in each treatment comparison. The number of studies per treatment comparison is presented on each edge, and the number of studies with individual patient data (IPD) is depicted in a parenthesis. Orange coloured edges are informed by both IPD and aggregate data, whereas black coloured edges are informed by aggregate data only.

Figure 3. Forest plot of network meta-analysis (NMA) results for all cognitive enhancers versus placebo in (a) MMSE outcome, and (b) AE outcome. NMA results are presented for i) aggregate data (AD) and fully adjusted results from studies with available individual patient data (IPD), ii) AD and crude results from studies with available IPD, iii) AD only (studies with available IPD are not included in the analysis), and iv) crude results from individual studies with individual patient data (IPD).

Figure 4. Rank-heat plot of P-scores for 9 treatments, including placebo, studied in randomized clinical trials with patients with Alzheimer's Dementia assessing MMSE. Circles from inside out present results for different network meta-analyses including: i) aggregate data (AD) only (studies with available IPD are not included in the analysis), ii) crude results from individual studies with individual patient data (IPD), iii) AD and crude results from studies with available IPD, and iv) AD and fully adjusted results from studies with available IPD. Numbers within each sector correspond to the P-score values as calculated in each model.

For peer review only

650 # Tables

651

Table 1· Study and patient characteristics	AD (N=80)	IPD (N=12)
**Total # participants**	21,138	5839
**Longest duration of follow-up in weeks: mean (range)**	28·28 (8 - 208)	29·33 (12 - 104)
**Mean number of patients (range)**	264·23 (14 - 2,045)	486·58 (123 - 2,045)
**Mean age in years (range)**	74·64 (61 - 85·7)	73·94 (70·4 - 78)
**Mean % Female (range)**	61·35 (3 - 89)	62·76 (53·68 - 81)
**Country of conduct: frequency (%)**		
Canada	2 (2·50)	1 (8·33)
China	6 (7·50)	-
Germany	1 (1·25)	-
Iran	2 (2·50)	-
Italy	6 (7·50)	-
Japan	7 (8·75)	1 (8·33)
Norway	1 (1·25)	-
Romania	1 (1·25)	-
South Korea	1 (1·25)	-
Spain	3 (3·75)	-
Sweden	2 (2·50)	-
Turkey	1 (1·25)	-
United Kingdom	6 (7·50)	1 (8·33)
United States	15 (18·75)	-
Multi-national	26 (32·50)	9 (75·00)
**Interventions examined: frequency***		
Placebo/no treatment	61 (76·25)	12 (100·00)
Donepezil	47 (58·75)	4 (33·33)
Galantamine	20 (25·00)	4 (33·33)
Memantine	20 (25·00)	3 (25·00)
Rivastigmine**	18 (22·50)	1 (8·33)
**Effectiveness outcomes reported: frequency***		
Mini-Mental State Examination	57 (71·25)	6 (50·00)
Adverse Events	46 (57·50)	12 (100·00)
**Funding**		
Industry-sponsored	48 (60·00)	12 (100·00)
Publicly-sponsored‡	9 (11·25)	-
Mixed	7 (8·75)	-
Not Reported	16 (20·0)	-
**Severity of Alzheimer's dementia: frequency (%)**		
Mild	3 (3·75)	-
Mild-Moderate	44 (55·00)	7 (58·33)
Mild-Severe	2 (2·50)	-
Moderate	3 (3·75)	-
Moderate-Severe	11 (13·75)	1 (8·33)
Severe	6 (7·50)	2 (16·67)

For peer review only - http://bmjopen.bmj.com/site/about/guidelines.xhtml

Not Reported	11 (13·75)	2 (16·67)
**Diagnostic criteria for Alzheimer's dementia: frequency***		
Mini-Mental State Examination	70 (87·50)	12 (100·00)
National Institute of Neurological Disorders and Stroke-Alzheimer Disease and Related Disorders Association	67 (83·75)	12 (100·00)
Diagnostic and Statistical Manual of Mental Disorders	39 (48·75)	5 (41·67)
Magnetic Resonance Imaging/Computerized Tomography	9 (11·25)	2 (16·67)
Clinical Dementia Rating	6 (7·50)	-
Hachinski Ischemic Score	5 (6·25)	-
Alzheimer's Disease Assessment Scale-Cognitive Subscale	3 (3·75)	1 (8·33)
Other	20 (25·00)	1 (8·33)

Abbreviations: -, not applicable

[*] Multiple interventions and outcomes reported per study;

[**] Rivastigmine refers to either oral or transdermal administration

[ǂ] Including sponsors such as the National Institute of Aging, UK Medical Research Council, and Veteran Affairs

652

For peer review only

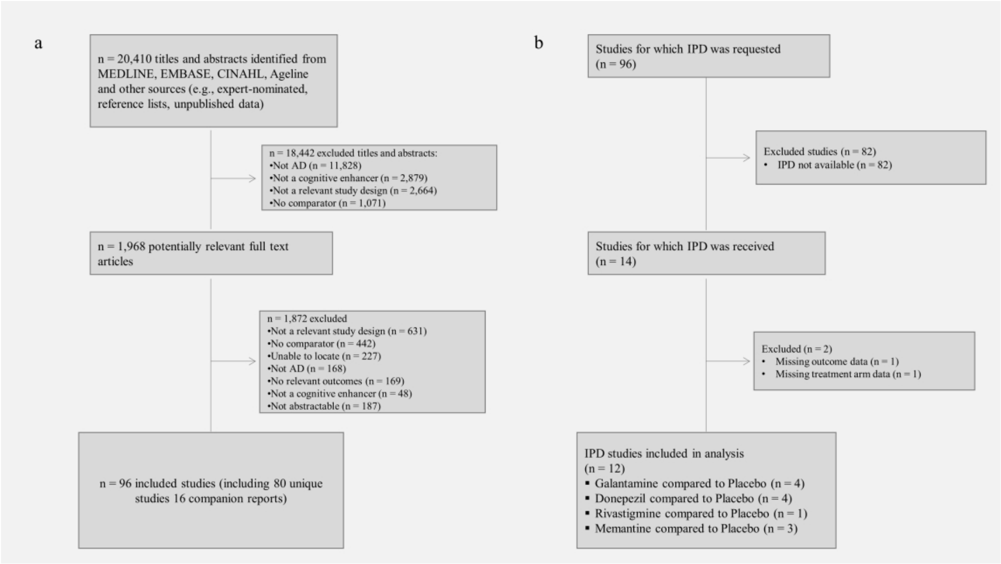

Figure 1. Flow diagram for study inclusion in the review (a) and studies retrieved with individual patient data (b).

338x190mm (300 x 300 DPI)

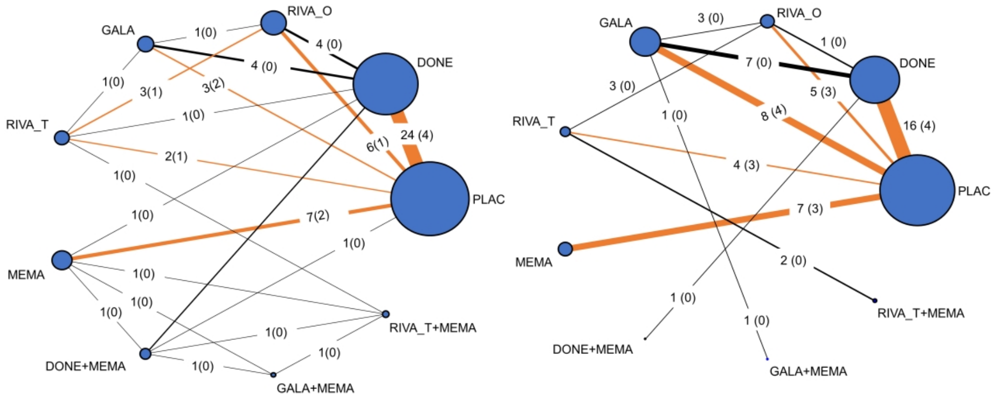

a. MMSE outcome

b. AE outcome

Network diagrams for (a) MMSE and (b) AE outcomes. The size of each node and line indicates the number of studies included in each treatment comparison. The number of studies per treatment comparison is presented on each edge, and the number of studies with individual patient data (IPD) is depicted in a parenthesis. Orange coloured edges are informed by both IPD and aggregate data, whereas black coloured edges are informed by aggregate data only.

338x190mm (300 x 300 DPI)

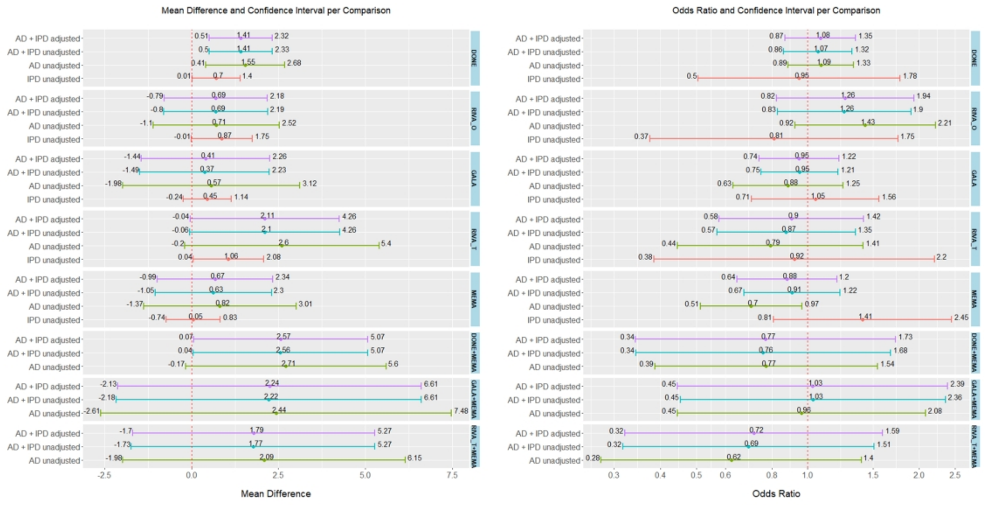

a. MMSE outcome b. AE outcome

Forest plot of network meta-analysis (NMA) results for all cognitive enhancers versus placebo in (a) MMSE outcome, and (b) AE outcome. NMA results are presented for i) aggregate data (AD) and fully adjusted results from studies with available individual patient data (IPD), ii) AD and crude results from studies with available IPD, iii) AD only (studies with available IPD are not included in the analysis), and iv) crude results from individual studies with individual patient data (IPD).

338x190mm (300 x 300 DPI)

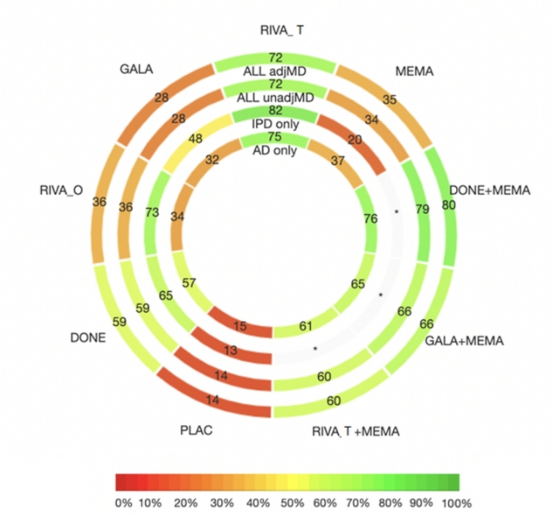

Figure 4. Rank-heat plot of P-scores for 9 treatments, including placebo, studied in randomized clinical trials with patients with Alzheimer's Dementia assessing MMSE. Circles from inside out present results for different network meta-analyses including: i) aggregate data (AD) only (studies with available IPD are not included in the analysis), ii) crude results from individual studies with individual patient data (IPD), iii) AD and crude results from studies with available IPD, and iv) AD and fully adjusted results from studies with available IPD. Numbers within each sector correspond to the P-score values as calculated in each model.

338x190mm (300 x 300 DPI)

Additional File 1: ***Comparative safety and efficacy of cognitive enhancers for Alzheimer's dementia: A systematic review with individual patient data network meta-analysis***

Appendix 1: Additional information on the methods used in the review ... 2
Appendix 2: Studies included in the systematic review ... 7
Appendix 3: Studies with available IPD but insufficient data to be included in the analysis ... 13
Appendix 4: List of studies requested and sponsor response ... 14
Appendix 5: Study characteristics of the included RCTs ... 17
Appendix 6. Characteristics of studies with shared IPD ... 24
Appendix 7: Comparison of studies with shared IPD with (a) all remaining studies and (b) studies for which sponsors claimed unavailable IPD. AD: aggregate data; IPD: individual patient data ... 27
Appendix 8: Cochrane Risk-of-bias appraisal results (n = 80) ... 29
Appendix 9: Overall risk of bias for studies with shared IPD against (a) all remaining studies and (b) studies for which sponsors claimed unavailable IPD. AD: aggregate data; IPD: individual patient data ... 31
Appendix 10: Study-specific effect sizes calculated from shared IPD and published data. IPD: individual patient data ... 32
Appendix 11: Correlation between participant age and dropout in studies with IPD. IPD: individual patient data ... 37
Appendix 12: Comparison Adjusted Funnel plot (all treatments vs placebo) ... 38
Appendix 13: Distribution of potential effect modifiers per treatment comparison and outcome ... 39
Appendix 14: Consistency Assessment – Loop-specific approach (using adjusted treatment effects) ... 42
Appendix 15: Network and standard meta-analysis results ... 43
Appendix 16: Network subgroup and meta-regression analysis results ... 46
Appendix 17: CINeMA results ... 53
Appendix 18: Study definitions for adverse events ... 56
Appendix 19: Time taken to achieve at least an adverse event using individual patient data ... 59
Appendix 20: Rank-heat plot for adverse events ... 60
Appendix 21: Challenges encountered during the individual patient data request from sponsors ... 61

For peer review only

Appendix 1: Additional information on the methods used in the review

Eligibility criteria, search strategy and study selection

We considered an adverse event (AE) as defined in the individual trials. Definitions were captured for each study separately. We included donepezil, rivastigmine, galantamine, and memantine alone or in combination with other treatment and compared with each other, supportive care or placebo. We excluded studies examining other cognitive enhancers or including individuals with mixed causes of dementia. We included published studies written in any language and of any duration.

Using terms from our previous review,4 the MEDLINE literature search was drafted by an experienced librarian (Dr. Laure Perrier) and revised after another librarian (Ms. Becky Skidmore) peer-reviewed the search terms.10 Subsequently, we searched the following databases: MEDLINE, EMBASE, Cochrane Methodology Register, CINAHL, Ageline and Cochrane Central Register of Controlled Trials. We also scanned reference lists of included studies and relevant reviews to supplement the electronic literature searches.

After pilot-testing, the results from the literature search were screened by pairs of reviewers working independently. Pairs of reviewers independently abstracted data (e.g., study characteristics, patient characteristics, outcome results) after a pilot-test. We resolved conflicts through discussion. The overall agreement among the reviewers for screening was over 70%.

IPD collection process and data abstraction

During the author contact process, two authors (a senior scientist ACT and a research assistant SL) sent a data request following several strategies as outlined in the RCT protocol:[1] a) an email requesting their IPD, b) email reminders (4 in total) at 2, 6, 10, and 14-week intervals after the initial email, c) reminders by post in week 7, and d) reminders via telephone in week 15. We also invited eligible authors to be a co-author on our updated systematic review provided that they share their anonymized IPD, and meet the International Committee of Medical Journal Editors (ICMJE) criteria for authorship.[2] Our team (AAV, SL) also contacted sponsors of the eligible trials, as reported in the publications. If a sponsor was not reported in a publication, we contacted the author (whom we emailed during the RCT) to determine who sponsored the study. To contact industry sponsors, we navigated the data sharing process from their websites or via an email, online portal, or phone inquiry. When no response was received, two follow-up reminders were sent to the sponsors.

We requested IPD on 1) patients: age, sex, severity of Alzheimer's disease (e.g. baseline MMSE [Mini-Mental State Examination] level), presence of behavioral disturbance, comorbid conditions (e.g., stroke, cardiovascular conditions, Parkinson's disease), other medications used for each patient, number of drop-outs, reasons for drop-out, and number of participants, 2) medication: treatment each patient was allocated to, dosage, 3) outcomes: event, date of event, time taken to achieve the event for AEs, MMSE values and measurement dates, and 4) date and method of randomization. We checked IPD provided for consistency with results from published RCTs., and contacted IPD providers when data inconsistencies were found.

Data extraction items included a) study characteristics: year of publication, country and continent according to the first author, journal in which the study was published, funding information; b) aggregate patient characteristics: study size and percentage of males, c) outcome data: study data (e.g., events or mean and standard deviations, and sample size per arm), and d) treatments compared. We also abstracted the corresponding authors' contact details. We categorized each study according to funding source (industry-sponsored, publicly-sponsored, mixed, and non-sponsored).

Certainty of the evidence

We used CINeMA (Confidence in Network Meta-Analysis) to assess confidence in the NMA estimates.[3] Six domains were evaluated with scores 'no concerns', 'some concerns' and 'major concerns': 1) within-study bias, 2) reporting bias, 3) indirectness, 4) imprecision, 5) heterogeneity, and 6) incoherence. We used the overall risk of bias per study, and for each treatment comparison we applied the average risk of bias. Similarly, for all treatment comparisons we used the average for indirectness. We assessed reporting bias based on the comparison-adjusted funnel plot since there are no established statistical methods to explore reporting bias. We used a comparison-adjusted funnel to account for the fact that each set of studies estimates a different summary effect in NMA. This is a scatterplot of the difference between the study-specific effect sizes from the

For peer review only - http://bmjopen.bmj.com/site/about/guidelines.xhtml

corresponding comparison-specific effect (obtained from standard meta-analysis) against the corresponding study-specific standard error. We used the fixed effect model for the standard meta-analysis performed for each treatment comparison, ordered treatments chronologically according to year of availability in Canada, and used only treatment comparisons versus placebo. We used the *netfunnel* command in Stata to produce the comparison-adjusted funnel plot.[4]

For imprecision, we considered a MD=1.4 and a OR=1 as a clinically important size of effect for MMSE and AE, respectively, and followed the CINeMA guidelines for exploring whether statistical significance and clinical importance coincide. Similarly, heterogeneity and incoherence (i.e. inconsistency) were assessed by following the standard CINeMA approach.

CINeMA assesses the credibility of the NMA results and heterogeneity examining the range of both confidence intervals (CIs; which do not capture heterogeneity) and prediction intervals (PIs; which capture heterogeneity) in relation to their equivalence. If a PI includes values that lead to a different conclusion than an assessment based on the corresponding CI, then this suggests that there is considerable heterogeneity. PIs are expected to include the true intervention effects in future studies with characteristics similar to the existing studies, and they incorporate the extent of between-study heterogeneity.[5 6] In the presence of considerable heterogeneity, they are wide to include intervention effects with different implications for practice. However, caution is needed in the interpretation of results in the presence of funnel plot asymmetry, since PIs are based on the assumption of a normal distribution for the study-specific effects and as such they may be problematic if the data do not follow a normal distribution.

Statistical Analysis

We performed a descriptive analysis using frequencies and percentages of the discrete characteristics of the included patients and treatments of the eligible studies. We explored the distributions of the continuous patient characteristics per outcome and treatment group using means and standard deviations. For studies not providing outcome results for a certain outcome, we presented distributions of the available and requested patient characteristics, whenever available. Outliers for each patient characteristic were also explored in each study dataset using boxplots. We also recorded the number of missing participants per treatment group and overall. We compared the characteristics of the unavailable and the available by the sponsors' studies. In particular, we explored whether these were well-conducted according to overall risk of bias, and compared distributions of mean participant age, publication year, study duration, study size, percent male, and magnitude of treatment effect, to assess for potential bias in IPD sharing. We conducted a two-stage analysis for both standard meta-analysis and NMA. The network geometry was explored through the presentation of network plots.

First stage

All IPD from included studies were first aggregated to study-level summary statistics using each sponsor's portal. The use of different platforms and failure to obtain IPD from all studies restricted us from combining IPD in a one-stage analysis. For each separate study with IPD available, we fitted a logistic regression model for the binary outcome and a linear regression model for the continuous outcome. For MMSE, we considered the longest duration of follow-up per study (most frequently at week 24). In the shared IPD, when we were unable to make a judgement on first and last date of visit per patient, we used the older coded date and the newest coded date as baseline and final value for each patient respectively.

Initially, we did not adjust for any of the patient characteristics provided, but in a subsequent analysis we included patient-level covariates with as many interaction terms in the model as the patient characteristics were provided (considering only the ones we have asked for). For each study, we obtained the adjusted odds ratio (OR) for binary data and adjusted mean difference (MD) for continuous data, along their corresponding 95% CI. We adjusted for any of the following variables that were available in each study: age, sex, severity of Alzheimer's disease (e.g., baseline Mini-Mental State Examination [MMSE] level), presence of behavioural disturbance, comorbidity, and other medications. The first stage of the IPD analyses were conducted in RStudio,[7] which was available in data providers. Additional medications and comorbid conditions were grouped into broader categories according to their clinical relevance to increase power in our analysis (e.g., grouped medications as anti-psychotics, anti-depressants, and cognitive enhancers, as well as comorbid conditions as psychiatric, neurological, and cardiac disorders). Eligible studies with insufficient data to derive a pairwise estimate for NMA were summarized descriptively without performing a statistical analysis.

For peer review only - http://bmjopen.bmj.com/site/about/guidelines.xhtml

We applied an available case analysis for each study, since we were unable to install R packages in most sponsor-specific platforms, and hence we applied a consistent approach across all IPD datasets. We explored the impact of missing data during the second stage of analysis. Reasons for missing participants and time taken to have a adverse event were captured (when available).

We synthesized IPD at the first stage in four different proprietary sponsor-specific platforms. Analyses were conducted in the RStudio using different R versions[7] according to what was provided in each sponsor's platform: R version 3.4.1 for AbbVie, R version 3.4.3 for CSDR, R version 3.5.1 for YODA, R version 3.6.0 for Lundbeck.

Second stage

Since we were not successful in obtaining IPD for all eligible studies, we combined both IPD and aggregate data in a single meta-analysis or NMA model. Both IPD and aggregate data studies shared the same amount of heterogeneity. In both meta-analysis and NMA models, we combined the adjusted IPD estimates with the aggregate data (main analysis). As a secondary analysis, we combined the unadjusted estimates from retrieved IPD with the evidence provided by the aggregated data studies in a joint NMA model. A common-within network between-study variance was assumed across comparisons for all NMA models.[8] We estimated the between-study variance using the DerSimonian and Laird[9] method and compared it with the relevant distributions provided by Turner et al[10] and Rhodes et al[11] to assess heterogeneity. We also calculated I^2 on the NMA level to quantify overall heterogeneity and inconsistency in each outcome.

To assess the validity of the transitivity assumption for each outcome, we assessed the distribution of potential effect modifiers (e.g., age, sex) across treatment comparisons in each network.[12-14] We visually inspected similarity and assessed whether these characteristics were likely to modify the treatment effect. We evaluated the consistency assumption using the design-by-treatment interaction model[15,16] and the loop-specific method.[17,18] In the presence of statistically significant inconsistency, we checked the data for discrepancies and if none were identified, we planned to conduct subgroup NMA or network meta-regression analysis adjusting for potential variables influencing the results.

We conducted additional NMA analyses for all potential effect modifiers requested from data providers. If relevant data were not available in the IPD, we used aggregate data of the relevant publications. Additional NMA analyses included: 1) subgroup analysis for industry vs. publicly sponsored studies, for studies with available IPD vs. studies with aggregate data (unadjusted estimates), and for AD severity, classified according to MMSE scores using the National Institute for Health and Care Excellence categories: mild (21–24), moderate (10–20), severe (<10),[19] 2) network meta-regression accounting for study duration, year of publication, mean age, and sex (% of male participants) effect modifiers separately and assuming a common regression coefficient across comparisons (studies with aggregate data were used only; studies with available IPD were pooled in a NMA separately adjusted for available covariates at first stage), 3) sensitivity analysis including studies with low risk of bias for allocation concealment and incomplete outcome data items, as these items may have an important impact on the meta-analysis results according to our previous NMA,[20] and 4) the 'informative missingness difference of means' (IMDoM) imputation method[21] for MMSE for the aggregate data studies to assess the impact of missing data in our NMA. In all additional NMA analyses, we used the adjusted effect estimates derived from the IPD within-study analysis and the aggregate data extracted from the eligible publications. Network meta-regression was performed in a Bayesian setting using OpenBUGS version 3.2.3, non-informative priors for all parameters in the model and a half-normal prior for the between standard deviation. We compared the results of the additional models by evaluating the treatment effect estimates and ranking statistics, as well as monitoring the reduction in the between-study variance.

We present the results using summary effect sizes, and in particular the MD for MMSE and the OR for AE, along with their corresponding CIs and PIs.[6] We ranked the interventions for each outcome according to their efficacy and safety using P-scores in frequentist analyses and SUCRAs (surface under the cumulative ranking curve) in Bayesian analyses (e.g., meta-regression analysis).[22,23] SUCRA is the numeric presentation of the intervention ranking and is based on the surface under the cumulative ranking probability function for each treatment. An equivalent frequentist statistic is the P-score measure that is based on the observed treatment effect estimates and their uncertainty. Both measures summarize the estimated probabilities for all possible ranks, account for uncertainty in relative ranking, and range between 0-100%, with 100% reflecting the best intervention with no uncertainty and 0% reflecting the worst intervention with no uncertainty. Ranking strategies are commonly encountered in NMAs,[24-26] and we present the hierarchy of cognitive enhancers in a rank-heat plot.[27]

For peer review only - http://bmjopen.bmj.com/site/about/guidelines.xhtml

Meta-analysis and NMA at the 2nd stage were conducted in the RStudio using R version 3.6.2 and the *meta*[28] and *netmeta*[29] packages, respectively.

Appendix 1 References:

1. Veroniki AA, Straus SE, Ashoor H, et al. Contacting authors to retrieve individual patient data: study protocol for a randomized controlled trial. *Trials* 2016;17(1):138. doi: 10.1186/s13063-016-1238-z [published Online First: 2016/03/16]
2. Hager K, Baseman AS, Nye JS, et al. Effects of galantamine in a 2-year, randomized, placebo-controlled study in Alzheimer's disease. *Neuropsychiatric disease and treatment* 2014;10:391-401. doi: 10.2147/ndt.s57909 [published Online First: 2014/03/05]
3. CINeMA: Confidence in Network Meta-Analysis. Institute of Social and Preventive Medicine, University of Bern. *Available from cinemaispmch* 2017
4. Chaimani A, Higgins JP, Mavridis D, et al. Graphical tools for network meta-analysis in STATA. *PLoS One* 2013;8(10):e76654. doi: 10.1371/journal.pone.0076654 [published Online First: 2013/10/08]
5. Higgins JP, Thompson SG, Spiegelhalter DJ. A re-evaluation of random-effects meta-analysis. *J R Stat Soc Ser A Stat Soc* 2009;172(1):137-59. doi: 10.1111/j.1467-985X.2008.00552.x [published Online First: 2009/04/22]
6. Riley RD, Higgins JP, Deeks JJ. Interpretation of random effects meta-analyses. *Bmj* 2011;342:d549. doi: 10.1136/bmj.d549 [published Online First: 2011/02/12]
7. R Core Team. R: A Language and Environment for Statistical Computing. Vienna, Austria: R Foundation for Statistical Computing, 2019.
8. Lu G, Ades AE. Combination of direct and indirect evidence in mixed treatment comparisons. *Statistics in medicine* 2004;23(20):3105-24. doi: 10.1002/sim.1875 [published Online First: 2004/09/28]
9. DerSimonian R, Laird N. Meta-analysis in clinical trials. *Controlled clinical trials* 1986;7(3):177-88. doi: 10.1016/0197-2456(86)90046-2
10. Turner RM, Davey J, Clarke MJ, et al. Predicting the extent of heterogeneity in meta-analysis, using empirical data from the Cochrane Database of Systematic Reviews. *International journal of epidemiology* 2012;41(3):818-27. doi: 10.1093/ije/dys041 [published Online First: 2012/03/31]
11. Rhodes KM, Turner RM, Higgins JP. Predictive distributions were developed for the extent of heterogeneity in meta-analyses of continuous outcome data. *Journal of clinical epidemiology* 2015;68(1):52-60. doi: 10.1016/j.jclinepi.2014.08.012 [published Online First: 2014/10/12]
12. Salanti G. Indirect and mixed-treatment comparison, network, or multiple-treatments meta-analysis: many names, many benefits, many concerns for the next generation evidence synthesis tool. *Res Synth Methods* 2012;3(2):80-97. doi: 10.1002/jrsm.1037 [published Online First: 2012/06/11]
13. Jansen JP, Naci H. Is network meta-analysis as valid as standard pairwise meta-analysis? It all depends on the distribution of effect modifiers. *BMC Med* 2013;11:159. doi: 10.1186/1741-7015-11-159 [published Online First: 2013/07/04]
14. Cipriani A, Higgins JP, Geddes JR, et al. Conceptual and technical challenges in network meta-analysis. *Annals of internal medicine* 2013;159(2):130-7. doi: 10.7326/0003-4819-159-2-201307160-00008
15. Higgins JP, Jackson D, Barrett JK, et al. Consistency and inconsistency in network meta-analysis: concepts and models for multi-arm studies. *Res Synth Methods* 2012;3(2):98-110. doi: 10.1002/jrsm.1044 [published Online First: 2012/06/01]
16. White IR BJ, Jackson D, Higgins JPT. Consistency and inconsistency in network meta-analysis: model estimation using multivariate meta-regression. *Res Synth Methods* 2012;3(2):15.
17. Song F, Altman DG, Glenny AM, et al. Validity of indirect comparison for estimating efficacy of competing interventions: empirical evidence from published meta-analyses. *Bmj* 2003;326(7387):472. doi: 10.1136/bmj.326.7387.472 [published Online First: 2003/03/01]
18. Veroniki AA, Vasiliadis HS, Higgins JP, et al. Evaluation of inconsistency in networks of interventions. *International journal of epidemiology* 2013;42(1):332-45. doi: 10.1093/ije/dys222
19. National Institute for Health and Clinical Excellence. Donepezil, galantamine, rivastigmine and memantine for the treatment of Alzheimer's disease. Review of NICE technology appraisal guidance 111 NICE technology appraisal guidance 217. London, UK, 2011.
20. Veroniki AA, Straus SE, Ashoor HM, et al. Comparative safety and effectiveness of cognitive enhancers for Alzheimer's dementia: protocol for a systematic review and individual patient data network meta-analysis. *BMJ open* 2016;6(1):e010251. doi: 10.1136/bmjopen-2015-010251

For peer review only - http://bmjopen.bmj.com/site/about/guidelines.xhtml

21. Mavridis D, White IR, Higgins JP, et al. Allowing for uncertainty due to missing continuous outcome data in pairwise and network meta-analysis. *Statistics in medicine* 2015;34(5):721-41. doi: 10.1002/sim.6365 [published Online First: 2014/11/14]
22. Rucker G, Schwarzer G. Ranking treatments in frequentist network meta-analysis works without resampling methods. *BMC Med Res Methodol* 2015;15:58. doi: 10.1186/s12874-015-0060-8 [published Online First: 2015/08/01]
23. Salanti G, Ades AE, Ioannidis JP. Graphical methods and numerical summaries for presenting results from multiple-treatment meta-analysis: an overview and tutorial. *J Clin Epidemiol* 2011;64(2):163-71. doi: 10.1016/j.jclinepi.2010.03.016 [published Online First: 2010/08/07]
24. Mbuagbaw L, Rochwerg B, Jaeschke R, et al. Approaches to interpreting and choosing the best treatments in network meta-analyses. *Syst Rev* 2017;6(1):79. doi: 10.1186/s13643-017-0473-z [published Online First: 2017/04/14]
25. Petropoulou M, Nikolakopoulou A, Veroniki AA, et al. Bibliographic study showed improving statistical methodology of network meta-analyses published between 1999 and 2015. *J Clin Epidemiol* 2017;82:20-28. doi: 10.1016/j.jclinepi.2016.11.002 [published Online First: 2016/11/20]
26. Veroniki AA, Straus SE, Rucker G, et al. Is providing uncertainty intervals in treatment ranking helpful in a network meta-analysis? *J Clin Epidemiol* 2018;100:122-29. doi: 10.1016/j.jclinepi.2018.02.009 [published Online First: 2018/02/13]
27. Veroniki AA, Straus SE, Fyraridis A, et al. The rank-heat plot is a novel way to present the results from a network meta-analysis including multiple outcomes. *Journal of clinical epidemiology* 2016;76:193-9. doi: 10.1016/j.jclinepi.2016.02.016 [published Online First: 2016/03/05]
28. Balduzzi S, Rücker G, Schwarzer G. How to perform a meta-analysis with R: a practical tutorial. *Evidence-based mental health* 2019;22(4):153-60. doi: 10.1136/ebmental-2019-300117 [published Online First: 2019/09/30]
29. Rücker G, Schwarzer G, Krahn U, et al. netmeta: Network Meta-Analysis using Frequentist Methods. R package version 0.9-8. 2018 [Available from: https://CRAN.R-project.org/package=netmeta.

For peer review only

For peer review only - http://bmjopen.bmj.com/site/about/guidelines.xhtml

Appendix 2: Studies included in the systematic review

80 Main Studies:

30. Agid Y, Dubois B, Anand R, Gharabawi G, International Rivastigmine Investigators. Efficacy and tolerability of rivastigmine in patients with dementia of the Alzheimer type. *Current Therapeutic Research* 1998; **59**(12): 837-45.
31. Ancoli-Israel S, Amatniek J, Ascher S, Sadik K, Ramaswamy K. Effects of galantamine versus donepezil on sleep in patients with mild to moderate Alzheimer disease and their caregivers: a double-blind, head-to-head, randomized pilot study. *Alzheimer disease and associated disorders* 2005; **19**(4): 240-5.
32. Andersen F, Viitanen M, Halvorsen DS, Straume B, Wilsgaard T, Engstad TA. The effect of stimulation therapy and donepezil on cognitive function in Alzheimer's disease. A community based RCT with a two-by-two factorial design. *BMC neurology* 2012; **12**: 59.
33. Araki T, Wake R, Miyaoka T, et al. The effects of combine treatment of memantine and donepezil on Alzheimer's disease patients and its relationship with cerebral blood flow in the prefrontal area. *International journal of geriatric psychiatry* 2014; **29**(9): 881-9.
34. Bakchine S, Loft H. Memantine treatment in patients with mild to moderate Alzheimer's disease: results of a randomised, double-blind, placebo-controlled 6-month study. *Journal of Alzheimer's disease : JAD* 2008; **13**(1): 97-107.
35. Black SE, Doody R, Li H, et al. Donepezil preserves cognition and global function in patients with severe Alzheimer disease. *Neurology* 2007; **69**(5): 459-69.
36. Blesa González R, Boada Rovira M, Martínez Parra C, Gil-Saladié D, Almagro CA, Gobartt Vázquez AL. Evaluation of the convenience of changing the rivastigmine administration route in patients with Alzheimer disease. *Neurologia (Barcelona, Spain)* 2011; **26**(5): 262-71.
37. Burns A, Bernabei R, Bullock R, et al. Safety and efficacy of galantamine (Reminyl) in severe Alzheimer's disease (the SERAD study): a randomised, placebo-controlled, double-blind trial. *The Lancet Neurology* 2009; **8**(1): 39-47.
38. Burns A, Perry E, Holmes C, et al. A double-blind placebo-controlled randomized trial of Melissa officinalis oil and donepezil for the treatment of agitation in Alzheimer's disease. *Dementia and geriatric cognitive disorders* 2011; **31**(2): 158-64.
39. Burns A, Rossor M, Hecker J, et al. The effects of donepezil in Alzheimer's disease - results from a multinational trial. *Dementia and geriatric cognitive disorders* 1999; **10**(3): 237-44.
40. Choi SH, Park KW, Na DL, et al. Tolerability and efficacy of memantine add-on therapy to rivastigmine transdermal patches in mild to moderate Alzheimer's disease: a multicenter, randomized, open-label, parallel-group study. *Current medical research and opinion* 2011; **27**(7): 1375-83.
41. Corey-Bloom J. A randomized trial evaluating the efficacy and safety of ENA 713 (rivastigmine tartrate), a new acetylcholinesterase inhibitor, in patients with mild to moderately severe Alzheimer's disease. *Int J Geriatr Psyopharmacol* 1998; **1**: 55-65.
42. Creţu O, Szalontay AS, Chiriţă R, Chiriţă V. Effect of memantine treatment on patients with moderate-to-severe Alzheimer's disease treated with donepezil. *Revista medico-chirurgicala a Societatii de Medici si Naturalisti din Iasi* 2008; **112**(3): 641-5.
43. Dysken MW, Sano M, Asthana S, et al. Effect of vitamin E and memantine on functional decline in Alzheimer disease: the TEAM-AD VA cooperative randomized trial. *Jama* 2014; **311**(1): 33-44.
44. Farlow MR, Grossberg GT, Sadowsky CH, Meng X, Somogyi M. A 24-week, randomized, controlled trial of rivastigmine patch 13.3 mg/24 h versus 4.6 mg/24 h in severe Alzheimer's dementia. *CNS neuroscience & therapeutics* 2013; **19**(10): 745-52.
45. Feldman H, Gauthier S, Hecker J, Vellas B, Subbiah P, Whalen E. A 24-week, randomized, double-blind study of donepezil in moderate to severe Alzheimer's disease. *Neurology* 2001; **57**(4): 613-20.
46. Feldman HH, Lane R. Rivastigmine: a placebo controlled trial of twice daily and three times daily regimens in patients with Alzheimer's disease. *Journal of neurology, neurosurgery, and psychiatry* 2007; **78**(10): 1056-63.

For peer review only - http://bmjopen.bmj.com/site/about/guidelines.xhtml

47. Fox C, Crugel M, Maidment I, et al. Efficacy of memantine for agitation in Alzheimer's dementia: a randomised double-blind placebo controlled trial. *PloS one* 2012; **7**(5): e35185.
48. Frölich L, Ashwood T, Nilsson J, Eckerwall G. Effects of AZD3480 on cognition in patients with mild-to-moderate Alzheimer's disease: a phase IIb dose-finding study. *Journal of Alzheimer's disease : JAD* 2011; **24**(2): 363-74.
49. Fuschillo C, Ascoli E, Franzese G, et al. Alzheimer's disease and acetylcholinesterase inhibitor agents: a two-year longitudinal study. *Archives of gerontology and geriatrics Supplement* 2004; (9): 187-94.
50. Gault LM, Ritchie CW, Robieson WZ, Pritchett Y, Othman AA, Lenz RA. A phase 2 randomized, controlled trial of the α7 agonist ABT-126 in mild-to-moderate Alzheimer's dementia. *Alzheimer's & dementia (New York, N Y)* 2015; **1**(1): 81-90.
51. Gold M, Alderton C, Zvartau-Hind M, et al. Rosiglitazone monotherapy in mild-to-moderate Alzheimer's disease: results from a randomized, double-blind, placebo-controlled phase III study. *Dementia and geriatric cognitive disorders* 2010; **30**(2): 131-46.
52. Greenberg SM, Tennis MK, Brown LB, et al. Donepezil therapy in clinical practice: a randomized crossover study. *Archives of neurology* 2000; **57**(1): 94-9.
53. Grossberg GT, Manes F, Allegri RF, et al. The safety, tolerability, and efficacy of once-daily memantine (28 mg): a multinational, randomized, double-blind, placebo-controlled trial in patients with moderate-to-severe Alzheimer's disease taking cholinesterase inhibitors. *CNS drugs* 2013; **27**(6): 469-78.
54. Hager K, Baseman AS, Nye JS, et al. Effects of galantamine in a 2-year, randomized, placebo-controlled study in Alzheimer's disease. *Neuropsychiatric disease and treatment* 2014; **10**: 391-401.
55. Haig GM, Pritchett Y, Meier A, et al. A randomized study of H3 antagonist ABT-288 in mild-to-moderate Alzheimer's dementia. *Journal of Alzheimer's disease : JAD* 2014; **42**(3): 959-71.
56. Hernandez C, Unturbe F, Martinez-Lage P, Lucas A, Gregorio P, Alonso T. Effects of combined pharmacologic and cognitive treatment in the progression of moderate dementia: a two-year follow-up. *REVISTA ESPANOLA DE GERIATRIA Y GERONTOLOGIA* 2007; **42**(1): 3.
57. Herrmann N, Gauthier S, Boneva N, Lemming OM. A randomized, double-blind, placebo-controlled trial of memantine in a behaviorally enriched sample of patients with moderate-to-severe Alzheimer's disease. *International psychogeriatrics* 2013; **25**(6): 919-27.
58. Holmes C, Wilkinson D, Dean C, et al. The efficacy of donepezil in the treatment of neuropsychiatric symptoms in Alzheimer disease. *Neurology* 2004; **63**(2): 214-9.
59. Homma A, Imah Y, Hariguchi S. Late phase II clinical study of acetylcholinesterase inhibitor E 2020 in patients with alzheimer-type dementia-12-weeks double-blind, placebo-controlled study 3 mg/day, 5mg/day. *Clinical Evaluation* 1998; **26**: 251-84.
60. Homma A, Imai Y, Tago H, et al. Donepezil treatment of patients with severe Alzheimer's disease in a Japanese population: results from a 24-week, double-blind, placebo-controlled, randomized trial. *Dementia and geriatric cognitive disorders* 2008; **25**(5): 399-407.
61. Hong Z, Zhang Z, Wang L, et al. A randomized study comparing the effect and safety of galantamine and donepezil in patients with mild to moderate Alzheimer's disease. *Chin J Neurol* 2006; **39**(6): 379-82.
62. Howard R, McShane R, Lindesay J, et al. Donepezil and memantine for moderate-to-severe Alzheimer's disease. *New England Journal of Medicine* 2012; **366**(10): 893-903.
63. Howard RJ, Juszczak E, Ballard CG, et al. Donepezil for the treatment of agitation in Alzheimer's disease. *The New England journal of medicine* 2007; **357**(14): 1382-92.
64. Hu HT, Zhang ZX, Yao JL, et al. [Clinical efficacy and safety of akatinol memantine in treatment of mild to moderate Alzheimer disease: a donepezil-controlled, randomized trial]. *Zhonghua nei ke za zhi* 2006; **45**(4): 277-80.
65. Johannsen P, Salmon E, Hampel H, et al. Assessing therapeutic efficacy in a progressive disease: a study of donepezil in Alzheimer's disease. *CNS drugs* 2006; **20**(4): 311-25.
66. Jones RW, Soininen H, Hager K, et al. A multinational, randomised, 12-week study comparing the effects of donepezil and galantamine in patients with mild to moderate Alzheimer's disease. *International journal of geriatric psychiatry* 2004; **19**(1): 58-67.

For peer review only - http://bmjopen.bmj.com/site/about/guidelines.xhtml

67. Kadir A, Darreh-Shori T, Almkvist O, et al. PET imaging of the in vivo brain acetylcholinesterase activity and nicotine binding in galantamine-treated patients with AD. *Neurobiology of aging* 2008; **29**(8): 1204-17.
68. Kano O, Ito H, Takazawa T, et al. Clinically meaningful treatment responses after switching to galantamine and with addition of memantine in patients with Alzheimer's disease receiving donepezil. *Neuropsychiatric disease and treatment* 2013; **9**: 259-65.
69. Karaman Y, Erdoğan F, Köseoğlu E, Turan T, Ersoy AO. A 12-month study of the efficacy of rivastigmine in patients with advanced moderate Alzheimer's disease. *Dementia and geriatric cognitive disorders* 2005; **19**(1): 51-6.
70. Likitjaroen Y, Meindl T, Friese U, et al. Longitudinal changes of fractional anisotropy in Alzheimer's disease patients treated with galantamine: a 12-month randomized, placebo-controlled, double-blinded study. *European archives of psychiatry and clinical neuroscience* 2012; **262**(4): 341-50.
71. Lorenzi M, Beltramello A, Mercuri NB, et al. Effect of memantine on resting state default mode network activity in Alzheimer's disease. *Drugs & aging* 2011; **28**(3): 205-17.
72. Maher-Edwards G, Dixon R, Hunter J, et al. SB-742457 and donepezil in Alzheimer disease: a randomized, placebo-controlled study. *International journal of geriatric psychiatry* 2011; **26**(5): 536-44.
73. Marek GJ, Katz DA, Meier A, et al. Efficacy and safety evaluation of HSD-1 inhibitor ABT-384 in Alzheimer's disease. *Alzheimer's & dementia : the journal of the Alzheimer's Association* 2014; **10**(5 Suppl): S364-73.
74. Mazza M, Capuano A, Bria P, Mazza S. Ginkgo biloba and donepezil: a comparison in the treatment of Alzheimer's dementia in a randomized placebo-controlled double-blind study. *European journal of neurology* 2006; **13**(9): 981-5.
75. Mohs RC, Doody RS, Morris JC, et al. A 1-year, placebo-controlled preservation of function survival study of donepezil in AD patients. *Neurology* 2001; **57**(3): 481-8.
76. Moretti DV. Alpha rhythm oscillations and MMSE scores are differently modified by transdermal or oral rivastigmine in patients with Alzheimer's disease. *American journal of neurodegenerative disease* 2014; **3**(2): 72-83.
77. Mowla A, Mosavinasab M, Haghshenas H, Borhani Haghighi A. Does serotonin augmentation have any effect on cognition and activities of daily living in Alzheimer's dementia? A double-blind, placebo-controlled clinical trial. *Journal of clinical psychopharmacology* 2007; **27**(5): 484-7.
78. Nakamura Y, Imai Y, Shigeta M, et al. A 24-week, randomized, double-blind, placebo-controlled study to evaluate the efficacy, safety and tolerability of the rivastigmine patch in Japanese patients with Alzheimer's disease. *Dementia and geriatric cognitive disorders extra* 2011; **1**(1): 163-79.
79. Nakano S, Asada T, Matsuda H, Uno M, Takasaki M. Donepezil hydrochloride preserves regional cerebral blood flow in patients with Alzheimer's disease. *Journal of nuclear medicine : official publication, Society of Nuclear Medicine* 2001; **42**(10): 1441-5.
80. Nordberg A, Darreh-Shori T, Peskind E, et al. Different cholinesterase inhibitor effects on CSF cholinesterases in Alzheimer patients. *Current Alzheimer research* 2009; **6**(1): 4-14.
81. Pakdaman H, Harandi AA, Hatamian H, et al. Effectiveness and Safety of MLC601 in the Treatment of Mild to Moderate Alzheimer's Disease: A Multicenter, Randomized Controlled Trial. *Dementia and geriatric cognitive disorders extra* 2015; **5**(1): 96-106.
82. Peng D, Xianhao X, Wang L. Efficiency and safety assessment of donepezil for treating mild and moderate Alzheimer disease. *Chinese Journal of Tissue Engineering Research* 2005; **9**(13): 170-2.
83. Peskind ER, Potkin SG, Pomara N, et al. Memantine treatment in mild to moderate Alzheimer disease: a 24-week randomized, controlled trial. *The American journal of geriatric psychiatry : official journal of the American Association for Geriatric Psychiatry* 2006; **14**(8): 704-15.
84. Peters O, Fuentes M, Joachim LK, et al. Combined treatment with memantine and galantamine-CR compared with galantamine-CR only in antidementia drug naïve patients with mild-to-moderate Alzheimer's disease. *Alzheimer's & dementia (New York, N Y)* 2015; **1**(3): 198-204.
85. Reisberg B, Doody R, Stöffler A, Schmitt F, Ferris S, Möbius HJ. Memantine in moderate-to-severe Alzheimer's disease. *The New England journal of medicine* 2003; **348**(14): 1333-41.

For peer review only - http://bmjopen.bmj.com/site/about/guidelines.xhtml

86. Rockwood K, Fay S, Song X, MacKnight C, Gorman M. Attainment of treatment goals by people with Alzheimer's disease receiving galantamine: a randomized controlled trial. *CMAJ : Canadian Medical Association journal = journal de l'Association medicale canadienne* 2006; **174**(8): 1099-105.
87. Rockwood K, Mintzer J, Truyen L, Wessel T, Wilkinson D. Effects of a flexible galantamine dose in Alzheimer's disease: a randomised, controlled trial. *Journal of neurology, neurosurgery, and psychiatry* 2001; **71**(5): 589-95.
88. Rogers SL, Doody RS, Mohs RC, Friedhoff LT. Donepezil improves cognition and global function in Alzheimer disease: a 15-week, double-blind, placebo-controlled study. Donepezil Study Group. *Archives of internal medicine* 1998; **158**(9): 1021-31.
89. Rogers SL, Farlow MR, Doody RS, Mohs R, Friedhoff LT. A 24-week, double-blind, placebo-controlled trial of donepezil in patients with Alzheimer's disease. Donepezil Study Group. *Neurology* 1998; **50**(1): 136-45.
90. Rogers SL, Friedhoff LT. The efficacy and safety of donepezil in patients with Alzheimer's disease: results of a US Multicentre, Randomized, Double-Blind, Placebo-Controlled Trial. The Donepezil Study Group. *Dementia (Basel, Switzerland)* 1996; **7**(6): 293-303.
91. Saxton J, Hofbauer RK, Woodward M, et al. Memantine and functional communication in Alzheimer's disease: results of a 12-week, international, randomized clinical trial. *Journal of Alzheimer's disease : JAD* 2012; **28**(1): 109-18.
92. Scarpini E, Bruno G, Zappalà G, et al. Cessation versus continuation of galantamine treatment after 12 months of therapy in patients with Alzheimer's disease: a randomized, double blind, placebo controlled withdrawal trial. *Journal of Alzheimer's disease : JAD* 2011; **26**(2): 211-20.
93. Schmidt R, Ropele S, Pendl B, et al. Longitudinal multimodal imaging in mild to moderate Alzheimer disease: a pilot study with memantine. *Journal of neurology, neurosurgery, and psychiatry* 2008; **79**(12): 1312-7.
94. Seltzer B, Zolnouni P, Nunez M, et al. Efficacy of donepezil in early-stage Alzheimer disease: a randomized placebo-controlled trial. *Archives of neurology* 2004; **61**(12): 1852-6.
95. Shao ZQ. Comparison of the efficacy of four cholinesterase inhibitors in combination with memantine for the treatment of Alzheimer's disease. *International journal of clinical and experimental medicine* 2015; **8**(2): 2944-8.
96. Shimizu S, Kanetaka H, Hirose D, Sakurai H, Hanyu H. Differential effects of acetylcholinesterase inhibitors on clinical responses and cerebral blood flow changes in patients with Alzheimer's disease: a 12-month, randomized, and open-label trial. *Dementia and geriatric cognitive disorders extra* 2015; **5**(1): 135-46.
97. Solé-Padullés C, Bartrés-Faz D, Lladó A, et al. Donepezil treatment stabilizes functional connectivity during resting state and brain activity during memory encoding in Alzheimer's disease. *Journal of clinical psychopharmacology* 2013; **33**(2): 199-205.
98. Tariot PN, Cummings JL, Katz IR, et al. A randomized, double-blind, placebo-controlled study of the efficacy and safety of donepezil in patients with Alzheimer's disease in the nursing home setting. *Journal of the American Geriatrics Society* 2001; **49**(12): 1590-9.
99. Tariot PN, Solomon PR, Morris JC, Kershaw P, Lilienfeld S, Ding C. A 5-month, randomized, placebo-controlled trial of galantamine in AD. The Galantamine USA-10 Study Group. *Neurology* 2000; **54**(12): 2269-76.
100. Thomas A, Iacono D, Bonanni L, D'Andreamatteo G, Onofrj M. Donepezil, rivastigmine, and vitamin E in Alzheimer disease: a combined P300 event-related potentials/neuropsychologic evaluation over 6 months. *Clinical neuropharmacology* 2001; **24**(1): 31-42.
101. Wilcock G, Howe I, Coles H, et al. A long-term comparison of galantamine and donepezil in the treatment of Alzheimer's disease. *Drugs & aging* 2003; **20**(10): 777-89.
102. Wilkinson D. A Clinical Study Evaluating the Effects of Memantine on Brain Atrophy in Patients With Alzheimer's Disease; 2012.
103. Wilkinson D, Murray J. Galantamine: a randomized, double-blind, dose comparison in patients with Alzheimer's disease. *International journal of geriatric psychiatry* 2001; **16**(9): 852-7.

For peer review only - http://bmjopen.bmj.com/site/about/guidelines.xhtml

104. Wilkinson DG, Passmore AP, Bullock R, et al. A multinational, randomised, 12-week, comparative study of donepezil and rivastigmine in patients with mild to moderate Alzheimer's disease. *International journal of clinical practice* 2002; **56**(6): 441-6.
105. Winblad B, Cummings J, Andreasen N, et al. A six-month double-blind, randomized, placebo-controlled study of a transdermal patch in Alzheimer's disease--rivastigmine patch versus capsule. *International journal of geriatric psychiatry* 2007; **22**(5): 456-67.
106. Winblad B, Engedal K, Soininen H, et al. A 1-year, randomized, placebo-controlled study of donepezil in patients with mild to moderate AD. *Neurology* 2001; **57**(3): 489-95.
107. Winblad B, Kilander L, Eriksson S, et al. Donepezil in patients with severe Alzheimer's disease: double-blind, parallel-group, placebo-controlled study. *Lancet (London, England)* 2006; **367**(9516): 1057-65.
108. Zhang Y, Rong-jia Y, Ju-ying G, H. G, Chao-mei W. Effects of aricept on the treatment of Alzheimer disease evaluated by skull multi-slice helical CT. *Chin J Clin Rehab* 2005; **9**(25): 20-1.
109. Zhang Z, Yu L, Gaudig M, Schäuble B, Richarz U. Galantamine versus donepezil in Chinese patients with Alzheimer's disease: results from a randomized, double-blind study. *Neuropsychiatric disease and treatment* 2012; **8**: 571-7.

16 Companion Reports

110. Aronson S, Van Baelen B, Kavanagh S, Schwalen S. Optimal dosing of galantamine in patients with mild or moderate Alzheimer's disease: post Hoc analysis of a randomized, double-blind, placebo-controlled trial. *Drugs & aging* 2009; 26(3): 231-9.
111. Cummings JL, Schneider L, Tariot PN, Kershaw PR, Yuan W. Reduction of behavioral disturbances and caregiver distress by galantamine in patients with Alzheimer's disease. *The American journal of psychiatry* 2004; 161(3): 532-8.
112. Feldman H, Gauthier S, Hecker J, et al. Economic evaluation of donepezil in moderate to severe Alzheimer disease. *Neurology* 2004; 63(4): 644-50.
113. Feldman H, Gauthier S, Hecker J, et al. Efficacy and safety of donepezil in patients with more severe Alzheimer's disease: a subgroup analysis from a randomized, placebo-controlled trial. *International journal of geriatric psychiatry* 2005; 20(6): 559-69.
114. Gaudig M, Richarz U, Han J, Van Baelen B, Schäuble B. Effects of galantamine in Alzheimer's disease: double-blind withdrawal studies evaluating sustained versus interrupted treatment. *Current Alzheimer research* 2011; 8(7): 771-80.
115. Gauthier S, Feldman H, Hecker J, Vellas B, Emir B, Subbiah P. Functional, cognitive and behavioral effects of donepezil in patients with moderate Alzheimer's disease. *Current medical research and opinion* 2002; 18(6): 347-54.
116. Grossberg GT, Farlow MR, Meng X, Velting DM. Evaluating high-dose rivastigmine patch in severe Alzheimer's disease: analyses with concomitant memantine usage as a factor. *Current Alzheimer research* 2015; 12(1): 53-60.
117. Han HJ, Kim BC, Lee JY, et al. Response to rivastigmine transdermal patch or memantine plus rivastigmine patch is affected by apolipoprotein E genotype in Alzheimer patients. *Dementia and geriatric cognitive disorders* 2012; 34(3-4): 167-73.
118. Jelic V, Haglund A, Kowalski J, Langworth S, Winblad B. Donepezil treatment of severe Alzheimer's disease in nursing home settings. A responder analysis. *Dementia and geriatric cognitive disorders* 2008; 26(5): 458-66.
119. Kumar V, Anand R, Messina J, Hartman R, Veach J. An efficacy and safety analysis of Exelon in Alzheimer's disease patients with concurrent vascular risk factors. *European journal of neurology* 2000; 7(2): 159-69.
120. Ott BR, Blake LM, Kagan E, Resnick M. Open label, multicenter, 28-week extension study of the safety and tolerability of memantine in patients with mild to moderate Alzheimer's disease. *Journal of neurology* 2007; 254(3): 351-8.

For peer review only - http://bmjopen.bmj.com/site/about/guidelines.xhtml

121. Reisberg B, Doody R, Stöffler A, Schmitt F, Ferris S, Möbius HJ. A 24-week open-label extension study of memantine in moderate to severe Alzheimer disease. *Archives of neurology* 2006; 63(1): 49-54.

122. Schwam E, Xu Y. Cognition and function in Alzheimer's disease: identifying the transitions from moderate to severe disease. *Dementia and geriatric cognitive disorders* 2010; 29(4): 309-16.

123. van Dyck CH, Tariot PN, Meyers B, Malca Resnick E. A 24-week randomized, controlled trial of memantine in patients with moderate-to-severe Alzheimer disease. *Alzheimer disease and associated disorders* 2007; 21(2): 136-43.

124. Wimo A, Winblad B, Engedal K, et al. An economic evaluation of donepezil in mild to moderate Alzheimer's disease: results of a 1-year, double-blind, randomized trial. *Dementia and geriatric cognitive disorders* 2003; 15(1): 44-54.

125. Winblad B, Grossberg G, Frölich L, et al. IDEAL: a 6-month, double-blind, placebo-controlled study of the first skin patch for Alzheimer disease. *Neurology* 2007; 69(4 Suppl 1): S14-22.

For peer review only

Appendix 3: Studies with available IPD but insufficient data to be included in the analysis

A study[1] of 859 participants comparing transdermal rivastigmine vs. placebo included only IPD for the placebo arm. Another study[2] of 285 participants comparing 22·5 mg of galantamine vs. 30 mg of galantamine vs. 45 mg of galantamine vs. placebo did not provide information about the AE or MMSE outcomes in the shared IPD.

CSDR: Novartis (study: NVT_SA_ENA713D1301) – Nakamura 2011

The study compares rivastigmine patch vs. placebo, but includes data only on placebo. Hence, we cannot conduct an analysis to convert data on their aggregated form so that to be included in our network meta-analysis. The IPD of this study included 288 participants in total.
According to the publication, 284 were allocated to the rivastigmine patch 5 cm2 group, 287 to the rivastigmine patch 10 cm2 group, and 288 to the placebo group.

Baseline characteristics of included patients

Characteristics	PLAC	Total	Missing Data	P-value	Outliers
Males	92 (32 %)	92 (32 %)	No	-	No
Age, mean (SD)	74.6 (7.4)	74.6 (7.4)	No	-	Yes - 1 value
AE, events/sample size	19/288	19/288	No	-	-
Baseline MMSE, mean (SD)	16.6 (2.9)	16.6 (2.9)	Yes - 1 value	-	No
MMSE, mean (SD)	17.5 (3.4)	17.5 (3.4)	No	-	No
Change score, mean (SD)	0.9 (1.6)	0.9 (1.6)	Yes - 2 values	-	Yes - 41 values
Total number of patients	288 (100 %)	288			

YODA: JNJ-Study-GAL-93-01 –Wilkinson 2001

The study compares galantamine 22.5mg, 30mg and 45mg vs placebo. In our analysis we combined galantamine 22.5mg, 30mg and 45mg in a single group. However, we only descriptively can include this study in our paper - not in the network meta-analysis – as it does not provide any info about the AE or MMSE outcomes (only total score for baseline). The IPD of this study included 285 participants in total.
According to the publication, 285 patients were randomized to: galantamine 18mg, 24mg, 36mg/day and placebo. Of the outcomes of interest, publication reported the AE outcome. According to the sponsor there are no differences in the reporting of doses:

- galantamine hydrobromide 7.5 mg =6 mg galantamine base was administered tid i.e galantamine hydrobromide 22.5 mg/d = galantamine base **18mg/day**
- galantamine hydrobromide 10 mg =8 mg galantamine base was administered tid i.e galantamine hydrobromide 30mg/d= galantamine base **24mg/day** and
- galantamine hydrobromide 15 mg =12 mg galantamine base was administered tid i.e galantamine hydrobromide 45mg/d= galantamine base **36mg/day**

Baseline characteristics of included patients

Characteristics	GALA	PLAC	Total	Missing Data	P-value	Outliers
Males	85 (30%)	36 (12%)	121 (42%)	No	<0.001	No
Age, mean (SD)	73.5 (8.2)	74.2 (9.0)	73.8 (8.5)	No	0.242	Yes - 1 value
AE, events/sample size*	-	-	-	-	-	-
Baseline MMSE, mean (SD)	18.6 (3.2)	18.8 (3.1)	18.7 (3.2)	No	0.616	No
MMSE, mean (SD)	-	-	-	-	-	-
Change score, mean (SD)	-	-	-	-	-	-
Total number of patients	198 (69%)	87 (31%)	285 (100%)			

*AE in publication is as follows, PLAC: 3/87, GALA 18mg: 6/88, GALA 24mg: 0/56, GALA 36mg: 5/54

[1]Nakamura Y, Imai Y, Shigeta M, et al. A 24-week, randomized, double-blind, placebo-controlled study to evaluate the efficacy, safety and tolerability of the rivastigmine patch in Japanese patients with Alzheimer's disease. Dement Geriatr Cogn Dis Extra 2011; 1(1): 163-79.
[2] Wilkinson D, Murray J. Galantamine: a randomized, double-blind, dose comparison in patients with Alzheimer's disease. Int J Geriatr Psychiatry 2001; 16(9): 852-7.

For peer review only - http://bmjopen.bmj.com/site/about/guidelines.xhtml

Appendix 4: List of studies requested and sponsor response

Sponsor	Author, year	Interventions compared (dosage mg)*	Sponsor Response	IPD Received
Abbvie	Gault, 2015	Placebo/No treatment, Donepezil (10 mg)	Available	Yes
	Haig, 2014	Placebo/No treatment, Donepezil (5 – 10 mg)	Available	Yes
	Marek, 2014	Placebo/No treatment, Donepezil (5 – 10 mg)	Unavailable (Cannot share data (Potential business considerations under review))	No
AstraZeneca	Frolich, 2011	Placebo/No treatment, Donepezil (5 – 10 mg)	Available	No
Daiichi-Sankyo	Shimizu, 2015	Donepezil (5 mg), Galantamine (24 mg), Rivastigmine (18 mg)	Unavailable (Do not own data)	No
Eisai	Black, 2007	Placebo/No treatment, Donepezil (5 – 10 mg)	Available	Yes
	Burns, 1999	Placebo/No treatment, Donepezil (5 – 10 mg)	Unavailable (Cannot share data (Old study))	No
	Feldman, 2001	Placebo/No treatment, Donepezil (5 – 10 mg)	Unavailable (Do not own data)	No
	Feldman, 2004	Placebo/No treatment, Donepezil (5 – 10 mg)	Unavailable (Do not own data)	No
	Feldman, 2005	Placebo/No treatment, Donepezil (5 – 10 mg)	Unavailable (Do not own data)	No
	Gauthier, 2002	Placebo/No treatment, Donepezil (5 – 10 mg)	Unavailable (Do not own data)	No
	Holmes, 2004	Placebo/No treatment, Donepezil (10 mg)	Unavailable (Do not own data)	No
	Homma, 2008	Placebo/No treatment, Donepezil (10 mg)	Unavailable (Cannot share data (Old study))	No
	Johannsen, 2006	Placebo/No treatment, Donepezil (10 mg)	Unavailable (Do not own data)	No
	Jones, 2004	Donepezil (5 – 10 mg), Galantamine (8 – 24 mg)	Unavailable (Cannot share data (Old study))	No
	Mohs, 2001	Placebo/No treatment, Donepezil (5 – 10 mg)	Unavailable (Cannot share data (Old study))	No
	Rogers, 1996	Placebo/No treatment, Donepezil (5 mg)	Unavailable (Cannot share data (Old study))	No
	Rogers, 1998	Placebo/No treatment, Donepezil (10 mg)	Unavailable (Cannot share data (Old study))	No
	Rogers, 1998	Placebo/No treatment, Donepezil (10 mg)	Unavailable (Cannot share data (Old study))	No
	Schwam, 2010	Placebo/No treatment, Donepezil (5 – 10 mg)	Unavailable (Do not own data)	No
	Seltzer, 2004	Donepezil (5 – 10 mg), Placebo/No treatment	Unavailable (Cannot share data (Old study))	No
	Shimizu, 2015	Donepezil (5 mg), Galantamine (24 mg), Rivastigmine (18 mg)	Unavailable (Do not own data)	No
	Sole-Padulles, 2013	Placebo/No treatment, Donepezil (5 – 10 mg)	Unavailable (Do not own data)	No
	Tariot, 2001	Placebo/No treatment, Donepezil (5 – 10 mg)	Unavailable (Cannot share data (Old study))	No
	Wilkinson, 2002	Donepezil (5 – 10 mg), Rivastigmine (6 – 12 mg)	Unavailable (Do not own data)	No
Forest Laboratories/Allergen	Grossberg, 2013	Donepezil (NR) + Rivastigmine (13.3 mg) + Galantamine + Placebo, Donepezil (NR) + Rivastigmine (4.6 mg) + Galantamine (NR)+ Memantine (NR)	Unavailable (Cannot share data (No details provided))	No
	Ott, 2007	Placebo/No treatment, Memantine (5 -20 mg)	Unavailable (Cannot share data (No details provided))	No
	Peskind, 2006	Placebo/No treatment, Memantine (5 -20 mg)	Unavailable (Cannot share data (No details provided))	No
	Saxton, 2012	Placebo/No treatment, Memantine (20 mg)	Unavailable (Cannot share data (No details provided))	No
	van Dyck, 2007	Placebo/No treatment, Memantine (20 mg)	Unavailable (Cannot share data (No details provided))	No
GlaxoSmithKline	Gold, 2010	Placebo/No treatment, Donepezil (10 mg)	Available	Yes
	Maher-Edwards, 2011	Placebo/No treatment, Donepezil (5 – 10 mg)	Unavailable (Do not own data)	No
Janssen	Ancoli-Israel, 2005	Donepezil (10 mg), Galantamine (8 mg)	Unavailable (Cannot identify study)	No
	Aronson, 2009	Placebo/No treatment, Galantamine (16 – 24 mg)	Unavailable (Cannot identify study)	No
	Burns, 2009	Placebo/No treatment, Galantamine (8-24 mg)	Available	Yes
	Cummings, 2004	Placebo/No treatment, Galantamine (4, 8, 12 mg)	Available	Yes
	Gaudig, 2011	Placebo/No treatment, Galantamine (8 mg)	Unavailable (Cannot identify study)	No
	Hager K, 2014	Placebo/No treatment, Galantamine (8 – 24 mg)	Available	Yes
	Kadir, 2008	Placebo/No treatment, Galantamine (16 – 24 mg)	Unavailable (Cannot identify study)	No
	Likitjaroen, 2012	Placebo/No treatment, Galantamine (8 – 24 mg)	Unavailable(Do not own data)	No
	Rockwood, 2001	Placebo/No treatment, Galantamine (24, 32 mg)	Available	Yes
	Rockwood, 2006	Placebo/No treatment, Galantamine (16 – 24 mg)	Unavailable (IPD not available)	No
	Scarpini, 2011	Placebo/No treatment, Galantamine (16 mg)	Unavailable (IPD not available)	No

For peer review only - http://bmjopen.bmj.com/site/about/guidelines.xhtml

Sponsor	Author, year	Interventions compared (dosage mg)*	Sponsor Response	IPD Received
	Shimizu, 2015	Donepezil (5 mg), Galantamine (24 mg), Rivastigmine (18 mg)	Unavailable (Cannot identify study)	No
	Tariot, 2000	Placebo/No treatment, Galantamine (8 mg)	Unavailable (Cannot identify study)	No
	Wilcock, 2003	Donepezil (5 – 10 mg), Galantamine (16 – 24 mg)	Unavailable (Cannot identify study)	No
	Zhang, 2012	Donepezil (5 – 10 mg), Galantamine (6 – 16 mg or 6 – 24 mg)	Unavailable (IPD not available)	No
	Wilkinson, 2001	Placebo/No treatment, Galantamine (18 - 36 mg)	Available	Yes
Lundbeck	Bakchine, 2008	Placebo/No treatment, Memantine (20 mg)	Available	Yes
	Fox, 2012	Placebo/No treatment, Memantine (5 – 20 mg)	Unavailable (Do not own data)	No
	Herrmann, 2013	Placebo/No treatment, Memantine (5 – 20 mg)	Available	Yes
	Lorenzi, 2011	Placebo/No treatment, Memantine (5 – 20 mg)	Unavailable (Do not own data)	No
	Wilkinson, 2012	Placebo/No treatment, Memantine (5 – 20 mg)	Available	Yes
Merz	Reisberg, 2003	Placebo/No treatment, Memantine (20 mg)	No response from sponsor	No
	Reisberg, 2006	Placebo/No treatment, Memantine (20 mg)	No response from sponsor	No
	Schmidt, 2008	Placebo/No treatment, Memantine (5 – 20 mg)	No response from sponsor	No
	Winblad, 2007	Placebo/No treatment, Rivastigmine (3 – 12 mg)	No response from sponsor	No
Novartis	Agid, 1998	Placebo/No treatment, Rivastigmine (6 mg)	Unavailable (Cannot identify study)	No
	Blesa González, 2011	Placebo/No treatment, Rivastigmine (6 – 12 mg)	Unavailable (Cannot share data)	No
	Choi, 2011	Placebo/No treatment, Memantine (5 – 20 mg)	Unavailable (Do not own data)	No
	Corey-Bloom, 1998	Placebo/No treatment, Rivastigmine (6 – 12 mg)	Unavailable (Cannot identify study)	No
	Farlow, 2013	Rivastigmine (4.6 - 13.3 mg), Rivastigmine (4.6 mg) + Memantine (20 mg)	Unavailable (Cannot share data (Phase 4 study))	No
	Feldman, 2007	Placebo/No treatment, Rivastigmine (2 – 12 mg)	Unavailable (Cannot identify study)	No
	Grossberg, 2015	Rivastigmine (4.6 - 13.3 mg), Rivastigmine (4.6 mg) + Memantine (20 mg)	Unavailable (Cannot share data (Phase 4 study))	No
	Han, 2012	Placebo/No treatment, Memantine (5 – 20 mg)	Unavailable (Cannot identify study)	No
	Kumar, 2000	Placebo/No treatment, Rivastigmine (1 – 12 mg)	Unavailable (Cannot identify study)	No
	Nakamura, 2011	Placebo/No treatment, Rivastigmine (4.5 – 9.5 mg)	Available	Yes
	Nordberg, 2009	Donepezil (5 – 10 mg), Galantamine (8 – 24 mg), Rivastigmine (3 – 12 mg)	Unavailable (Cannot share data (Phase 4 study))	No
	Shimizu, 2015	Donepezil (5 mg), Galantamine (24 mg), Rivastigmine (18 mg)	Unavailable (Cannot identify study)	No
	Winblad, 2007	Placebo/No treatment, Rivastigmine (3 – 12 mg)	Available	Yes
ONO	Nakamura, 2011	Placebo/No treatment, Rivastigmine (4.5 – 9.5 mg)	No response from sponsor	No
Pfizer	Black, 2007	Placebo/No treatment, Donepezil (10 mg)	Unavailable (Do not own data)	No
	Feldman, 2001	Placebo/No treatment, Donepezil (5 – 10 mg)	Available	No
	Feldman, 2004	Placebo/No treatment, Donepezil (5 – 10 mg)	Unavailable (Cannot identify study)	No
	Feldman, 2005	Placebo/No treatment, Donepezil (5 – 10 mg)	Unavailable (Cannot identify study)	No
	Gauthier, 2002	Placebo/No treatment, Donepezil (5 – 10 mg)	Unavailable (Cannot identify study)	No
	Holmes, 2004	Placebo/No treatment, Donepezil (10 mg)	Unavailable (Cannot identify study)	No
	Jelic, 2008	Placebo/No treatment, Donepezil (5 – 10 mg)	Unavailable (Cannot identify study)	No
	Johannsen, 2006	Placebo/No treatment, Donepezil (10 mg)	Unavailable (Cannot identify study)	No
	Jones, 2004	Donepezil, Galantamine (8 – 24 mg)	Unavailable (Cannot identify study)	No
	Mohs, 2001	Placebo/No treatment, Donepezil (5 – 10 mg)	Unavailable (Cannot identify study)	No
	Schwam, 2010	Placebo/No treatment, Donepezil (5 – 10 mg)	Unavailable (Cannot identify study)	No
	Seltzer, 2004	Donepezil (5 – 10 mg), Placebo/No treatment	Unavailable (Cannot identify study)	No
	Sole-Padulles, 2013	Placebo/No treatment, Donepezil (5 – 10 mg)	Unavailable (Cannot identify study)	No
	Tariot, 2001	Placebo/No treatment, Donepezil (5 – 10 mg)	Unavailable (Cannot identify study)	No

For peer review only - http://bmjopen.bmj.com/site/about/guidelines.xhtml
For peer review only

Sponsor	Author, year	Interventions compared (dosage mg)*	Sponsor Response	IPD Received
	Wilkinson, 2002	Donepezil (5 – 10 mg), Rivastigmine (6 – 12 mg)	Unavailable (Cannot identify study)	No
	Wimo, 2003	Placebo/No treatment, Donepezil (5 – 10 mg)	Unavailable (Cannot identify study)	No
	Winblad, 2001	Placebo/No treatment, Donepezil (5 – 10 mg)	Unavailable (Cannot identify study)	No
	Winblad, 2006	Placebo/No treatment, Donepezil (5 – 10 mg)	Unavailable (Cannot identify study)	No
Roivant	Maher-Edwards, 2011	Placebo/No treatment, Donepezil (5 – 10 mg)	No response from sponsor	No
Shire Pharmaceuticals	Wilcock, 2003	Donepezil (5 – 10 mg), Galantamine (16 – 24 mg)	Unavailable (Do not own data)	No
	Wilkinson, 2001	Placebo/No treatment, Galantamine (24 mg)	Unavailable (Do not own data)	No
Takeda	Shimizu, 2015	Donepezil (5 mg), Galantamine (24 mg), Rivastigmine (18 mg)	Unavailable (Do not own data)	No
Non-Pharmaceutical	Andersen, 2012	Placebo/No treatment, Donepezil (5 – 10 mg)	NA	No
	Araki, 2014	Placebo/No treatment, Donepezil (NR) + Memantine (5 – 20 mg)	NA	No
	Burns, 2011	Placebo/No treatment, Donepezil (5 – 10 mg)	NA	No
	Dysken, 2014	Placebo/No treatment, Memantine (20 mg)	Available	No
	Greenberg, 2000	Placebo/No treatment, Donepezil (5 mg)	Unavailable (Need to contact PI)	No
	Howard, 2007	Placebo/No treatment, Donepezil (5 – 10 mg)	Unavailable (Do not own data)	No
	Howard, 2012	Donepezil (10 mg) + Memantine (5 – 20 mg), Donepezil (10 mg) + Placebo	Unavailable (Do not own data)	No
	Mowla, 2007	Placebo/No treatment, Rivastigmine (3 – 12 mg)	NA	No
	Peters, 2015	Galantamine (24 mg) + Placebo, Galantamine (24 mg) + Memantine (20 mg)	NA	No
Not reported	Cretu, 2008	Placebo/No treatment, Memantine (5 – 20 mg)	NA	No
	Fuschillo, 2001	Donepezil (5 mg), Rivastigmine (6 – 9 mg)	NA	No
	Hernández, 2007	Placebo/No treatment, Donepezil (10 mg)	NA	No
	Homma, 1998	Donepezil (3 – 5 mg), Placebo/no treatment	NA	No
	Hong, 2006	Placebo/No treatment, Galantamine (8 – 24 mg)	NA	No
	Hu, 2006	Donepezil (5 mg), Memantine (5 – 10 mg)	NA	No
	Kano, 2013	Donepezil(10 mg), Donepezil (10 mg) + Memantine (20 mg)	NA	No
	Karaman, 2005	Placebo/No treatment, Rivastigmine (3 – 12 mg)	NA	No
	Mazza, 2006	Placebo/No treatment, Donepezil (5 mg)	NA	No
	Moretti, 2014	Placebo/No treatment, Rivastigmine (3 – 12 mg)	NA	No
	Nakano, 2001	Placebo/No treatment, Donepezil (5 mg)	NA	No
	Pakdaman H, 2015	Donepezil (NR), Galantamine (NR), Rivastigmine (NR)	NA	No
	Peng, 2005	Placebo/No treatment, Donepezil (5 mg)	NA	No
	Shao, 2015	Memantine (5 – 10 mg)+ Placebo, Rivastigmine (1.5 – 3 mg) + Memantine (5 – 10 mg), Donepezil (5 – 10 mg) + Memantine (5 – 10 mg), Galantamine (2 – 6 mg) + Memantine (5 – 10 mg)	NA	No
	Thomas, 2001	Donepezil (5 – 10 mg), Rivastigmine (6 – 12 mg)	NA	No
	Zhang-Yi, 2005	Placebo/No treatment, Donepezil (5 mg)	NA	No

Abbreviations: NA, not applicable; NPH, neutral protamine Hagedorn; NR, not reported; PI, principal investigator

* In studies that examined different dosages of the same intervention, we selected the dosages that were consistent with those approved for use in Canada.

For peer review only

Appendix 5: Study characteristics of the included RCTs

Study	Country of conduct	Sample size; Longest duration of follow-up (weeks)	Treatments compared; Outcomes	Funding information	Date of randomization; Date trial opened; Randomization ratio	IPD available; Reasons for not providing IPD by the data providers
Agid, 1998	12 countries - Austria, Belgium, Czechoslovakia, Denmark, Finland, France, Germany, Ireland, Norway, Sweden, Switzerland, and the UK	402; 13	Rivastigmine, Placebo/No treatment; MMSE, Nausea, Vomiting, Diarrhea, AEs, Headaches	Industry-sponsored	Not reported; Not reported; Not reported	No; Cannot identify study
Ancoli-Israel, 2005	USA	63; 8	Galantamine, Donepezil; CIBIC-plus, Mortality, Nausea, Diarrhea, AEs, Headaches	Industry-sponsored	Not reported; Not reported; Not reported	No; Cannot identify study
Andersen, 2012	Norway	180; 52	Donepezil, Placebo; MMSE, ADAS-cog	Publicly-sponsored	Not reported; June 2003; Not reported	No; NA
Araki, 2014	Japan	37; 24	Donepezil + Memantine, Placebo; MMSE, NPI	Publicly-sponsored	Not reported; Not reported; Not reported	No; NA
Bakchine, 2008	12 countries -Austria, Belgium, Denmark, Finland, France, Greece, Lithuania, the Netherlands, Poland, Spain, Sweden and UK	470; 24	Memantine, Placebo/no treatment; ADAS-cog, ADCS-ADL, NPI, CIBIC-plus, Mortality, AEs, Headaches, Falls	Industry-sponsored	Not reported; Not reported; Not reported	Yes; NA
Black, 2007	5 countries - USA, Canada, France, UK, Australia	343; 24	Donepezil, Placebo/No treatment; MMSE, ADCS-ADL, NPI, CIBIC-plus, Nausea, Vomiting, Diarrhea, AEs	Industry-sponsored	Not reported; January 2001; Not reported	Yes; Do not own data
Blesa González, 2011	Spain	139; 12	Rivastigmine Patch, Rivastigmine Oral; MMSE, Nausea, Vomiting, Diarrhea	Industry-sponsored	Not reported; Not reported; Not reported	No; Cannot share data (Phase 4 study)
Burns, 1999	Australia, Belgium, Canada, France, Germany,Ireland, New Zealand, South Africa and the UK	818; 30	Donepezil, Placebo/no treatment; ADAS-cog, CIBIC-plus, Mortality, Diarrhea, Nausea, AEs, Vomiting	Industry-sponsored	Not reported; Not reported; Not reported	No; Cannot share data (Old study)
Burns, 2009	Belgium, Finland, France, Italy, Norway, Netherlands, Spain, Sweden, Switzerland, UK	407; 26	Galantamine, Placebo/no treatment; Mortality, Nausea, Vomiting, Diarrhea, AEs, Headaches, Falls	Industry-sponsored	Not reported; December 2003; Not reported	Yes; NA
Burns, 2011	UK	62; 12	Donepezil, Placebo/no treatment; NPI, AEs	Publicly-sponsored	Not reported; January 2006; Not reported	No; NA
Choi, 2011	South Korea	171; 16	Memantine, Placebo/No treatment; MMSE, ADAS-cog, ADCS-ADL, NPI, AEs, Nausea, Diarrhea, Vomiting, Headaches	Publicly-sponsored + Industry-sponsored	Not reported; December 2008; Not reported	No; Do not own data
Corey-Bloom, 1998	USA	699; 26	Rivastigmine, Placebo/No treatment; MMSE, ADAS-cog, CIBIC-plus, Mortality, Nausea, Vomiting	Industry-sponsored	Not reported; Not reported; Not reported	No; Cannot identify study

For peer review only - http://bmjopen.bmj.com/site/about/guidelines.xhtml

Cretu, 2008	Romania	43;<br>24	Memantine, Placebo/No treatment;<br>MMSE, ADAS-cog, NPI	NA	Not reported;<br>Not reported;<br>Not reported	No;<br>NR
Dysken, 2014	USA	307;<br>26-208	Memantine, Placebo;<br>MMSE, ADAS-cog, ADCS-ADL, NPI, Mortality, AEs	Publicly-sponsored	Not reported;<br>August 2007;<br>1:1:1:1	No;<br>NA
Farlow, 2013	USA	716;<br>24	Rivastigmine + Memantine, Rivastigmine;<br>NPI, Mortality, Falls, Vomiting, Diarrhea, Nausea	Industry-sponsored	Not reported;<br>July 2009;<br>1:1	No;<br>Cannot share data (Phase 4 study)
Feldman, 2001	Canada, Australia, France	290;<br>24	Donepezil, Placebo/No treatment;<br>MMSE, NPI, CIBIC-plus, Mortality, Vomiting, Nausea, Diarrhea, AEs, Headaches	Industry-sponsored	Not reported;<br>Not reported;<br>"50/50 split"	No;<br>NA
Feldman, 2007	Australia, Canada, Ireland, Italy, South Africa, UK	450;<br>26	Rivastigmine, Placebo/No treatment;<br>MMSE, ADAS-cog, CIBIC-plus, AEs, Bradycardia, Headaches, Vomiting, Diarrhea, Nausea	Industry-sponsored	Not reported;<br>Not reported;<br>1:1:1	No;<br>Cannot identify study
Fox, 2012	UK	149;<br>12	Memantine, Placebo;<br>MMSE, NPI, Mortality	Industry-sponsored	Not reported;<br>September 2007;<br>"assigned with equal probability"	No;<br>Unavailable (Do not own data)
Frolich, 2011	Austria, Belgium, Bulgaria, Czech Republic, Germany, Romania, Russia, Spain, UK, Canada	324;<br>12	Donepezil, Placebo/No treatment;<br>MMSE, ADAS-cog, Nausea, Vomiting, Diarrhea, Headaches	Industry-sponsored	Not reported;<br>July 2007;<br>Not reported	No;<br>Available
Fuschillo, 2001	Italy	27;<br>30	Donepezil, Rivastigmine;<br>MMSE, ADAS-cog, Headaches, Vomiting, Diarrhea, Nausea	NA	Not reported;<br>Not reported;<br>Not reported	No;<br>NR
Gault, 2015	USA, Bulgaria, Czech Republic, Slovakia, UK, South Africa	136;<br>14	Donepezil, Placebo;<br>MMSE, ADAS-cog, ADCS-ADL, NPI, CIBIC-plus, Mortality, AEs, Bradycardia, Falls, Headaches, Vomiting, Diarrhea, Nausea	Industry-sponsored	Not reported;<br>October 2009;<br>Not reported	Yes;<br>Available
Gold, 2010	Austria, Bulgaria, Chile, China, Croatia, Estonia, Germany, Greece, Hungary, Mexico, New Zealand, Pakistan, Peru, Republic of the Philippines, Puerto Rico, Republic of Korea, Russian Federation, UK and USA	248;<br>24	Donepezil, Placebo/no treatment;<br>ADAS-cog, CIBIC-plus, Mortality, Headaches, Nausea, Diarrhea, AEs	Industry-sponsored	Not reported;<br>February 2007;<br>2:2:2:1	Yes;<br>Available
Greenberg, 2000	USA	103;<br>24	Donepezil, Placebo/no treatment;<br>ADAS-cog, AEs, Diarrhea, Nausea	Publicly-sponsored	Not reported;<br>Not reported;<br>Not reported	No;<br>Contact PI
Grossberg, 2013	Argentina, USA, Mexico, Chile	676;<br>24	Donepezil + Rivastigmine + Galantamine + Memantine, Donepezil + Rivastigmine + Galantamine + Placebo;<br>NPI, CIBIC-plus, Mortality, Falls,	Industry-sponsored	Not reported;<br>June 2005;<br>1:1	No;<br>Cannot share dat

For peer review only

			Headaches, Vomiting, Diarrhea, Nausea, AEs			
Hager K, 2014	Czech Republic, Estonia, France, Germany, Greece, Italy, Latvia, Lithuania, Romania, Russia, Slovakia, Slovenia, Ukraine	2045; 104	Galantamine, Placebo; MMSE, Mortality, Headaches, Vomiting, Diarrhea, Nausea, AEs	Industry-sponsored	Not reported; May 2008; 1:1	Yes; NA
Haig, 2014	Russia, Ukraine	123; 12	Donepezil, Placebo; MMSE, ADAS-cog, ADCS-ADL, NPI, Headaches, Nausea, AEs	Industry-sponsored	Not reported; Not reported; 1:1:1	Yes; NA
Hernández, 2007	Spain	20; 48	Donepezil, Placebo/No treatment; MMSE, ADAS-cog	NA	Not reported; Not reported; Not reported	No; NR
Herrmann, 2013	Canada	369; 24	Memantine, Placebo; NPI, Mortality, Falls, Nausea, AEs	Industry-sponsored	Not reported; December 2003; "equally allocated"	Yes; NA
Holmes, 2004	UK	96; 24	Donepezil, Placebo/No treatment; MMSE, NPI	Industry-sponsored	Not reported; Not reported; 3:2	No; Cannot identify study
Homma, 1998	Japan	187; 12	Donepezil, Placebo/no treatment; ADAS-cog, Mortality, AEs, Headaches	NA	Not reported; Not reported; Not reported	No; NR
Homma, 2008	Japan	267; 24	Donepezil, Placebo/no treatment; ADCS-ADL, CIBIC-plus, Mortality, AEs, Falls, Vomiting, Diarrhea	Industry-sponsored	Not reported; Not reported; 1:1:1	No; Cannot share data (Old study)
Hong, 2006	China	218; 16	Galantamine, Placebo/no treatment; ADAS-cog, ADCS-ADL, NPI, AEs	NA	Not reported; Not reported; Not reported	No; NR
Howard, 2007	England	259; 12	Donepezil, Placebo/No treatment; MMSE, NPI, Mortality, Falls, Diarrhea	Publicly-sponsored	Not reported; November 2003; "probability ratios of 0.75 and 0.25 to assign treatment"	No; NA
Howard, 2012	Europe	295; 52	Donepezil + Placebo, Donepezil + Memantine; MMSE, Mortality, AEs, Falls	Publicly-sponsored	Not reported; February 2008; Not reported	No; Do not own data
Hu, 2006	China	97; 16	Memantine, Donepezil; MMSE	NA	Not reported; Not reported; Not reported	No; NA
Johannsen, 2006	Belgium, Denmark, Germany, Greece, Hungary, Iceland, The Netherlands, Poland, USA	202; 48	Donepezil, Placebo/No treatment; MMSE, ADAS-cog, NPI, Headaches, Diarrhea, Nausea	Industry-sponsored	Not reported; February 1999; Not reported	No; Do not own data
Jones, 2004	UK, Finland, Germany and Norway	120; 12	Donepezil, Galantamine; MMSE, ADAS-cog, Headaches, Vomiting, Diarrhea, Nausea, AEs	Industry-sponsored	Not reported; Not reported; 1:1	No; Cannot share data (Old study)
Kadir, 2008	Sweden	18; 48	Galantamine, Placebo/No treatment; MMSE, ADAS-cog	Industry-sponsored + Other	Not reported; Not reported; Not reported	No; Cannot identify study

For peer review only - http://bmjopen.bmj.com/site/about/guidelines.xhtml

Kano, 2013;	Japan	30;<br>28	Donepezil, Donepezil + Memantine ;<br>MMSE	NA	Not reported;<br>August 2011;<br>Not reported	No;<br>NR
Karaman, 2005	Turkey	44;<br>52	Rivastigmine, Placebo/No treatment;<br>MMSE, ADAS-cog, ADAS-ADL, CIBIC-plus, Headaches, Vomiting, Nausea	NA	Not reported;<br>Not reported;<br>Not reported	No;<br>NR
Likitjaroen, 2012	Germany	25;<br>26	Galantamine, Placebo;<br>MMSE	Publicly-sponsored + Industry-sponsored	Not reported;<br>September 2006;<br>Not reported	No;<br>Do not own data
Lorenzi, 2011	Italy	15;<br>24	Memantine, Placebo/No treatment;<br>MMSE	Publicly-sponsored + Industry-sponsored	Not reported;<br>Not reported;<br>Not reported	No;<br>Do not own data
Maher-Edwards, 2011	Austria, Bulgaria, Chile, Estonia, Germany, Russia, Slovakia, and UK	129;<br>24	Donepezil, Placebo/no treatment;<br>ADAS-cog, CIBIC-plus, Mortality, AEs, Headaches, Nausea	Industry-sponsored	Not reported;<br>May 2006;<br>1:1:1	No;<br>No response from sponsor
Marek, 2014	UK, Ukraine, South Africa, Russia	132;<br>16	Donepezil, Placebo;<br>MMSE, ADAS-cog, NPI, CIBIC-plus, Mortality, Headaches, Vomiting, Diarrhea, AEs	Industry-sponsored	Not reported;<br>May 2010;<br>"equal proportions"	No;<br>Cannot share data
Mazza, 2006	Italy	51;<br>24	Donepezil, Placebo/No treatment;<br>MMSE	NA	Not reported;<br>March 2003;<br>1:1:1	No;<br>NR
Mohs, 2001	USA	431;<br>54	Donepezil, Placebo/No treatment;<br>MMSE, Mortality, AEs, Headaches, Diarrhea, Nausea	Industry-sponsored	Not reported;<br>Not reported;<br>Not reported	No;<br>Cannot share data
Moretti, 2014	Italy	20;<br>78	Rivastigmine Patch, Rivastigmine Oral;<br>MMSE	NA	Not reported;<br>Not reported;<br>Not reported	No;<br>NA
Mowla, 2007	Iran	81;<br>12	Rivastigmine, Placebo/No treatment;<br>MMSE	Publicly-sponsored	Not reported;<br>Not reported;<br>Not reported	No;<br>NA
Nakamura, 2011	Japan	855;<br>24	Rivastigmine, Placebo/No treatment;<br>MMSE, AEs, Vomiting, Nausea, Diarrhea	Industry-sponsored	Not reported;<br>January 2007;<br>Not reported	Yes;<br>NA
Nakano, 2001	Japan	35;<br>48	Donepezil, Placebo/No treatment;<br>MMSE	NA	Not reported;<br>Not reported;<br>Not reported	No;<br>NR
Nordberg, 2009	USA	63;<br>13	Rivastigmine, Donepezil, Galantamine;<br>AEs, Headaches, Vomiting, Diarrhea, Nausea	Industry-sponsored	Not reported;<br>Not reported;<br>1:1:1	No;<br>Cannot share data
Pakdaman H, 2015	Iran	198;<br>68.8	Donepezil, Galantamine, Rivastigmine;<br>MMSE, ADAS-cog, Mortality,	Industry-sponsored	Not reported;<br>Not reported;<br>Not reported	No;<br>NR

For peer review only

			Headaches, Vomiting, Diarrhea, Nausea			
Peng, 2005	China	89;<br>12	Donepezil, Placebo/No treatment;<br>MMSE	NA	Not reported;<br>1998;<br>Not reported	No;<br>NR
Peskind, 2006	USA	403;<br>24	Memantine, Placebo/no treatment;<br>ADAS-cog, ADCS-ADL, NPI, CIBIC-plus, Nausea, Vomiting, Diarrhea	Industry-sponsored	Not reported;<br>Not reported;<br>Not reported	No;<br>Cannot share data
Peters, 2015	Europe	226;<br>52	Galantamine + Memantine, Galantamine + Placebo;<br>ADAS-cog, ADCS-ADL, NPI, Mortality, AEs, Falls	Publicly-sponsored	Not reported;<br>Not reported;<br>Not reported	No;<br>NA
Reisberg, 2003	USA	252;<br>28	Memantine, Placebo/No treatment;<br>MMSE, ADCS-ADL, NPI, CIBIC-plus, Mortality, AEs, Diarrhea	Publicly-sponsored + Industry-sponsored	Not reported;<br>August 1998;<br>Not reported	No;<br>No response from sponsor
Rockwood, 2001	Australia, Canada, Great Britian, New Zealand, South Africa, USA	386;<br>12	Galantamine, Placebo/no treatment;<br>ADAS-cog, NPI, CIBIC-plus, Mortality, AEs, Vomiting, Nausea	Industry-sponsored	Not reported;<br>Not reported;<br>Not reported	Yes;<br>NA
Rockwood, 2006	Canada	130;<br>16	Galantamine, Placebo/no treatment;<br>ADAS-cog, CIBIC-plus, AEs, Vomiting, Nausea	Publicly-sponsored + Industry-sponsored	Not reported;<br>November 2001;<br>Not reported	No;<br>IPD not available
Rogers, 1996	USA	161;<br>12	Donepezil, Placebo/No treatment;<br>MMSE, ADAS-cog, Headaches, Diarrhea	Industry-sponsored	Not reported;<br>Not reported;<br>Not reported	No;<br>Cannot share data
Rogers, 1998	USA	468;<br>12	Donepezil, Placebo/No treatment;<br>MMSE, ADAS-cog, CIBIC-plus, AEs, Headaches, Vomiting, Diarrhea, Nausea	Industry-sponsored	Not reported;<br>Not reported;<br>Not reported	No;<br>Cannot share data
Rogers, 1998	USA	473;<br>24	Donepezil, Placebo/No treatment;<br>MMSE, ADAS-cog, CIBIC-plus, Mortality, AEs, Vomiting, Diarrhea, Nausea	Industry-sponsored	Not reported;<br>Not reported;<br>Not reported	No;<br>Cannot share data
Saxton, 2012	Australia, South Africa, New Zealand	264;<br>12	Memantine, Placebo;<br>Mortality, Falls, Headaches, Diarrhea, Nausea, AEs	Industry-sponsored	Not reported;<br>April 2007;<br>Not reported	No;<br>Cannot share data
Scarpini, 2011	Italy	139;<br>96	Galantamine, Placebo/no treatment;<br>Mortality, AEs	Industry-sponsored	Not reported;<br>July 2001;<br>Not reported	No;<br>IPD not available
Schmidt, 2008	Europe	36;<br>52	Memantine, Placebo/No treatment;<br>MMSE, ADAS-cog, ADCS-ADL	Industry-sponsored	Not reported;<br>Not reported;<br>Not reported	No;<br>No response from sponsor
Seltzer, 2004	USA	153;<br>24	Donepezil, Placebo/No treatment;<br>MMSE, ADAS-cog, Diarrhea, Nausea	Industry-sponsored	Not reported;<br>Not reported;<br>Not reported	No;<br>Cannot identify study

For peer review only - http://bmjopen.bmj.com/site/about/guidelines.xhtml

For peer review only

Shao, 2015	China	110;<br>24	Donepezil + Memantine, Galantamine + Memantine, Memantine + Placebo, Rivastigmine + Memantine;<br>MMSE, ADCS-ADL	NA	Not reported;<br>October 2009;<br>Not reported	No;<br>NR
Shimizu, 2015	Japan	75;<br>52	Donepezil, Galantamine, Rivastigmine;<br>MMSE, ADAS-cog, NPI, Headaches, Vomiting, Nausea	Industry-sponsored	Not reported;<br>Not reported;<br>Not reported	No;<br>Do not own data
Sole-Padulles, 2013	Spain	14;<br>13	No treatment, Donepezil;<br>MMSE, NPI	Industry-sponsored	Not reported;<br>Not reported;<br>Not reported	No;<br>Do not own data
Tariot, 2000	USA	978;<br>20	Galantamine, Placebo/no treatment;<br>ADAS-cog, ADCS-ADL, NPI, Mortality, AEs, Vomiting, Diarrhea, Nausea	Industry-sponsored	Not reported;<br>Not reported;<br>Not reported	No;<br>Cannot identify study
Tariot, 2001	USA	208;<br>24	Donepezil, Placebo/No treatment;<br>MMSE, Mortality, AEs, Bradycardia, Headaches, Vomiting, Diarrhea, Nausea	Industry-sponsored	Not reported;<br>Not reported;<br>Not reported	No;<br>Cannot identify study
Thomas, 2001	Italy	40;<br>24	Donepezil, Rivastigmine;<br>MMSE, ADAS-cog	NA	Not reported;<br>Not reported;<br>Not reported	No;<br>NR
Wilcock, 2003	UK	188;<br>52	Galantamine, Donepezil;<br>MMSE, ADAS-cog, Mortality, AEs, Falls, Headaches, Vomiting, Nausea	Industry-sponsored	Not reported;<br>June 2000;<br>Not reported	No;<br>Cannot identify study
Wilkinson, 2001	UK	180;<br>12	Galantamine, Placebo/no treatment;<br>ADAS-cog, AEs, Headaches, Vomiting, Diarrhea, Nausea	Industry-sponsored	Not reported;<br>May 1994;<br>Not reported	Yes;<br>NA
Wilkinson, 2002	UK, South Africa, and Switzerland	111;<br>12	Donepezil, Rivastigmine;<br>MMSE, ADAS-cog, Mortality, AEs, Bradycardia, Headaches, Vomiting, Nausea	Industry-sponsored	Not reported;<br>Not reported;<br>1:1	No;<br>Cannot identify study
Wilkinson, 2012	France, Germany, Switzerland, UK	277;<br>52	Memantine, Placebo/No treatment;<br>MMSE, NPI, Mortality, AEs, Falls	Industry-sponsored	Not reported;<br>September 2005;<br>1:1	Yes;<br>NA
Winblad, 2001	Denmark, Finland, Norway, Sweden, the Netherlands	286;<br>52	Donepezil, Placebo/No treatment;<br>MMSE, AEs, Bradycardia, Headaches, Diarrhea, Nausea	Industry-sponsored	Not reported;<br>Not reported;<br>Not reported	No;<br>Cannot identify study
Winblad, 2006	Sweden	248;<br>24	Donepezil, Placebo/No treatment;<br>MMSE, NPI, Mortality, AEs, Falls, Diarrhea, Nausea	Industry-sponsored	Not reported;<br>October 2002;<br>Not reported	No;<br>Cannot identify study
Winblad, 2007	Chile, Czech Republic, Denmark, Finland, Germany, Guatemala, Israel, Italy, Korea, Mexico, Norway, Peru, Poland, Portugal, Russia, Slovak Republic, Sweden, Taiwan, USA, Uruguay, Venezuela	1190;<br>24	Rivastigmine, Placebo/No treatment;<br>MMSE, ADAS-cog, ADCS-ADL, NPI, Mortality, AEs, Headaches, Vomiting, Diarrhea, Nausea	Industry-sponsored	Not reported;<br>November 2003;<br>Not reported	No;<br>No response from sponsor

For peer review only

For peer review only - http://bmjopen.bmj.com/site/about/guidelines.xhtml

Zhang-Yi, 2005	China	120;<br>8	Donepezil, Placebo/No treatment;<br>MMSE	NA	Not reported;<br>Not reported;<br>Not reported	No;<br>NR
Zhang, 2012	China	218;<br>16	Galantamine, Donepezil;<br>MMSE, ADAS-cog, ADCS-ADL, NPI, Mortality, Vomiting, Diarrhea, Nausea, AEs	Industry-sponsored	Not reported;<br>Not reported;<br>Not reported	No;<br>IPD not available

For peer review only

Appendix 6. Characteristics of studies with shared IPD

Study	Provided by	Severity of AD*	Previous response to treatment for AD	Presence of behavioural disturbance	Comorbid conditions	Other medications used	Treatment Group	Males (%)	Age, mean (SD)
Black 2007	CSDR - EISAI	Severe	NR	NR	All patients included the same exact comorbidities	NR	Donepezil	48 (27%)	78 (7.9)
							Placebo	54 (32%)	78 (8.1)
Gold 2010	CSDR - GSK	Mild-Moderate	NR	NR	Multiple reported	Multiple reported	Donepezil	16 (29%)	76.6 (8.2)
							Placebo	49 (46%)	75.5 (8.2)
Winblad 2007	CSDR - Novartis	Mild-Moderate	NR	NR	Multiple reported	Multiple reported	Rivastigmine patch	198 (33 %)	73.9 (8.0)
							Rivastigmine oral	102 (34 %)	72.9 (8.2)
							Placebo	101 (33%)	73.8 (7.5)
Hager 2014	YODA - Janssen	Mild-Moderate	NR	NR	NR	Multiple reported	Galantamine	354 (34%)	73 (8.9)
							Placebo	367 (36%)	73 (8.7)
Rockwood 2001	YODA - Janssen	Mild-Moderate	NR	NR	NR	Multiple reported	Galantamine	113 (43%)	75 (7.3)
							Placebo	58 (46%)	75 (7.6)
Cummings 2004	YODA - Janssen	NR	NR	NR	Multiple reported	Multiple reported	Galantamine	245 (35%)	76.9 (7.8)
							Placebo	108 (38%)	77.2 (7.9)
Burns 2009	YODA - Janssen	Severe	NR	NR	Multiple reported	Multiple reported	Galantamine	42 (20%)	84.0 (6.5)
							Placebo	39 (19%)	83.8 (6.7)
Gault 2015	AbbVie	Mild-Moderate	NR	NR	NR	Multiple reported	Donepezil	37 (54%)	72.4 (8.4)
							Placebo	26 (38%)	73.6 (8.2)
Haig 2014	AbbVie	Mild-Moderate	NR	NR	Multiple reported	Multiple reported	Donepezil	24 (40%)	70 (8.3)
							Placebo	24 (38%)	70 (7.8)
Bakchine 2008	Lundbeck	Mild-Moderate	NR	NR	NR	Multiple reported	Memantine	112 (35%)	74 (7.4)
							Placebo	61 (40%)	73 (6.9)
Herrman 2013	Lundbeck	69 (48%)	NR	NR	NR	Multiple reported	Memantine	77 (42%)	75 (7.9)
							Placebo	77 (41%)	75 (6.9)
Wilkinson 2012	Lundbeck	NR	NR	NR	NR	Multiple reported	Memantine	50 (38%)	74 (8.8)
							Placebo	69 (48%)	74 (7.8)

Additional characteristics of studies with shared IPD

Study	Patients experiencing at least one AE	Missing data in AE outcome	Baseline MMSE, mean (SD)	Final MMSE, mean (SD)	Change score, mean (SD)	Missing data in MMSE outcome	Total number of patients	Reasons for dropouts as indicated in the provided IPD	Time taken for the 1st AE
Black 2007	21	0 (0%)	7.5 (3.3)	8.2 (5.2)	0.63 (3.1)	27 (15%)	176 (51%)	• intercurrent illness (1 [2%] – donepezil = 1; placebo = 0),<br>• request of patient or investigator (4 [7%] –	617 days (range [110, 1292])

For peer review only - http://bmjopen.bmj.com/site/about/guidelines.xhtml

	25	0 (0%)	7.4 (3.6)	7.6 (4.8)	-0.15 (3.5)	27 (16%)	167 (49%)	donepezil = 3; placebo = 1),<br>• patient entered nursing home/facility (5 [9%] – donepezil = 1; placebo =) 4,<br>• due to adverse experience (30 [56%] – donepezil = 15; placebo = 15), and<br>• other (14 [26%] – donepezil = 7; placebo = 7)	691 days (range [78, 1475]).
Gold 2010	6	0 (0%)	20 (3.7)	21 (4.6)	1.11 (2.3)	18 (32%)	56 (34%)	• Adverse Event (16 [39%] – donepezil = 9; placebo = 7),<br>• Lost to Follow-Up (4 [10%] – donepezil = 3; placebo = 1),<br>• Non-compliance (6 [15%] – donepezil = 2; placebo = 4),<br>• Subject decided to withdraw (11 [26%] – donepezil = 4; placebo = 7)	349 days (range [48, 656])
	10	0 (0%)	20.1 (4.2)	20.4 (5.4)	0.08 (2.7)	23 (22%)	107 (66%)		492 days (range [95, 780])
Winblad 2007	83	0 (0%)	16.6 (3.0)	17.7 (4.7)	1 (3.4)	74 (10%)	598 (50 %)	NR	NR
	37	0 (0%)	16.4 (3.1)	17.2 (4.6)	0.8 (3.2)	31 (12%)	297 (25 %)	NR	NR
	45	0 (0%)	16.4 (3.0)	16.4 (5.3)	-0.1 (3.6)	21 (7%)	302 (25 %)	NR	NR
Hager 2014	73	0 (0%)	19.0 (4.1)	17.81 (6.2)	-1.38 (4.3)	228 (22%)	1027 (50%)	NR	NR
	92	0 (0%)	19.0 (4.0)	16.99 (6.3)	-2.15 (4.4)	236 (23%)	1022 (50%)	NR	NR
Rockwood 2001	27	0 (0%)	23.2 (5.2)	NR	NR	NR	261 (68%)	NR	NR
	5	0 (0%)	22.9 (5.0)	NR	NR	NR	125 (32%)	NR	NR
Cummings 2004	23	0 (0%)	20.7 (4.9)	NR	NR	NR	692 (71%)	NR	NR
	81	0 (0%)	20.6 (4.9)	NR	NR	NR	286 (29%)	NR	NR
Burns 2009	62	0 (0%)	NR	9.2 (4.5)†	NR	NR	211 (51%)	NR	NR
	75	0 (0%)	NR	9.6 (4.9)†	NR	NR	204 (49%)	NR	NR
Gault 2015	5	0 (0%)	19.2 (4.1)	20.7 (5.1)	1.5 (2.6)	48 (71%)	68 (50%)	NR	305 days (range [224, 377])
	3	0 (0%)	18.8 (4)	18.9 (4.8)	0.1 (2.4)	45 (66%)	68 (50%)	NR	239 days (range [206, 295])
Haig 2014	2	0 (0%)	17.9 (4.2)	19.7 (3.9)	1.2 (2.8)	41 (68%)	60 (49%)	NR	286 days (range N/A – a single date was provided)
	1	0 (0%)	17.8 (3.8)	19.9 (4.2)	1.8 (1.8)	47 (75%)	63 (51%)	NR	270 days (range [161, 379]).
Bakchine 2008	33	0 (0%)	18.7 (3.3)	NR	NR	NR	318 (68%)	NR	NR
	9	0 (0%)	18.9 (3.2)	NR	NR	NR	152 (32%)	NR	NR
Herrman 2013	18	0 (0%)	11.9 (3.1)	11.3 (4.9)	-0.76 (3.4)	31 (8%)	182 (49%)	NR	NR
	11	0 (0%)	11.8 (2.9)	11.1 (4.7)	-0.68 (3.2)	32 (9%)	187 (51%)	NR	NR

For peer review only

Wilkinson 2012	17	0 (0%)	16.7 (2.5)	16.4 (5.2)	-0.46 (3.9)	30 (11%)	133 (48%)	NR	NR
	20	0 (0%)	17.1 (2.4)	16.4 (5.6)	-0.69 (4.0)	30 (11%)	144 (52%)	NR	NR

* According to publication
† The MMSE final value comes from visit 8 (last available visit in IPD). MMSE was not reported in study publication
Abbreviations: AD, Alzheimer's Dementia; IPD, individual patient data; MMSE, Mini-Mental State Examination; NR, not reported; N/A, not applicable; AE, adverse event

For peer review only

Appendix 7: Comparison of studies with shared IPD with (a) all remaining studies and (b) studies for which sponsors claimed unavailable IPD. AD: aggregate data; IPD: individual patient data

a. Comparison of studies with shared IPD with all remaining studies (irrespective type of sponsor)

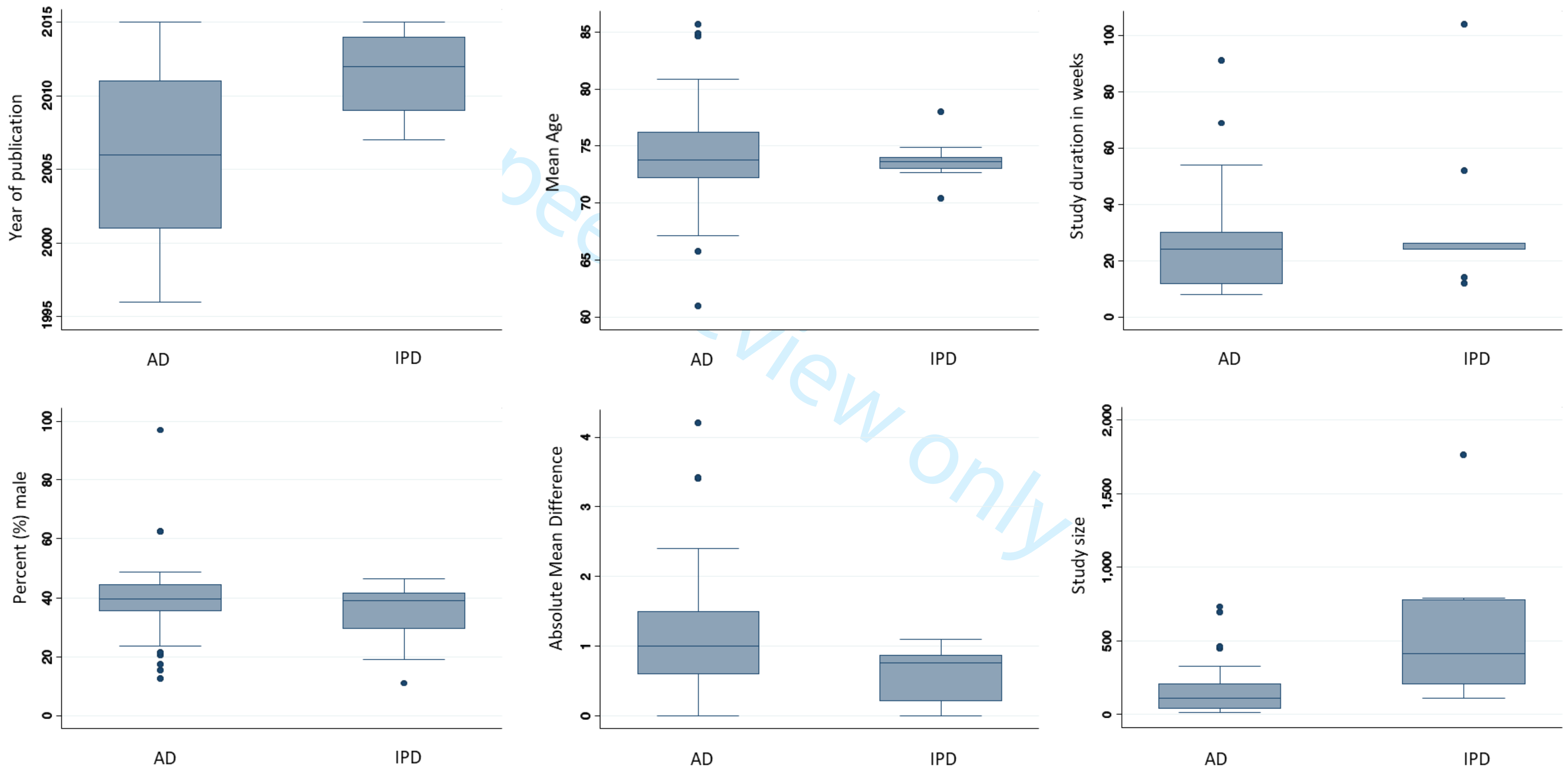

b. Comparison of studies with available and unavailable IPD (industry-sponsored studies only)

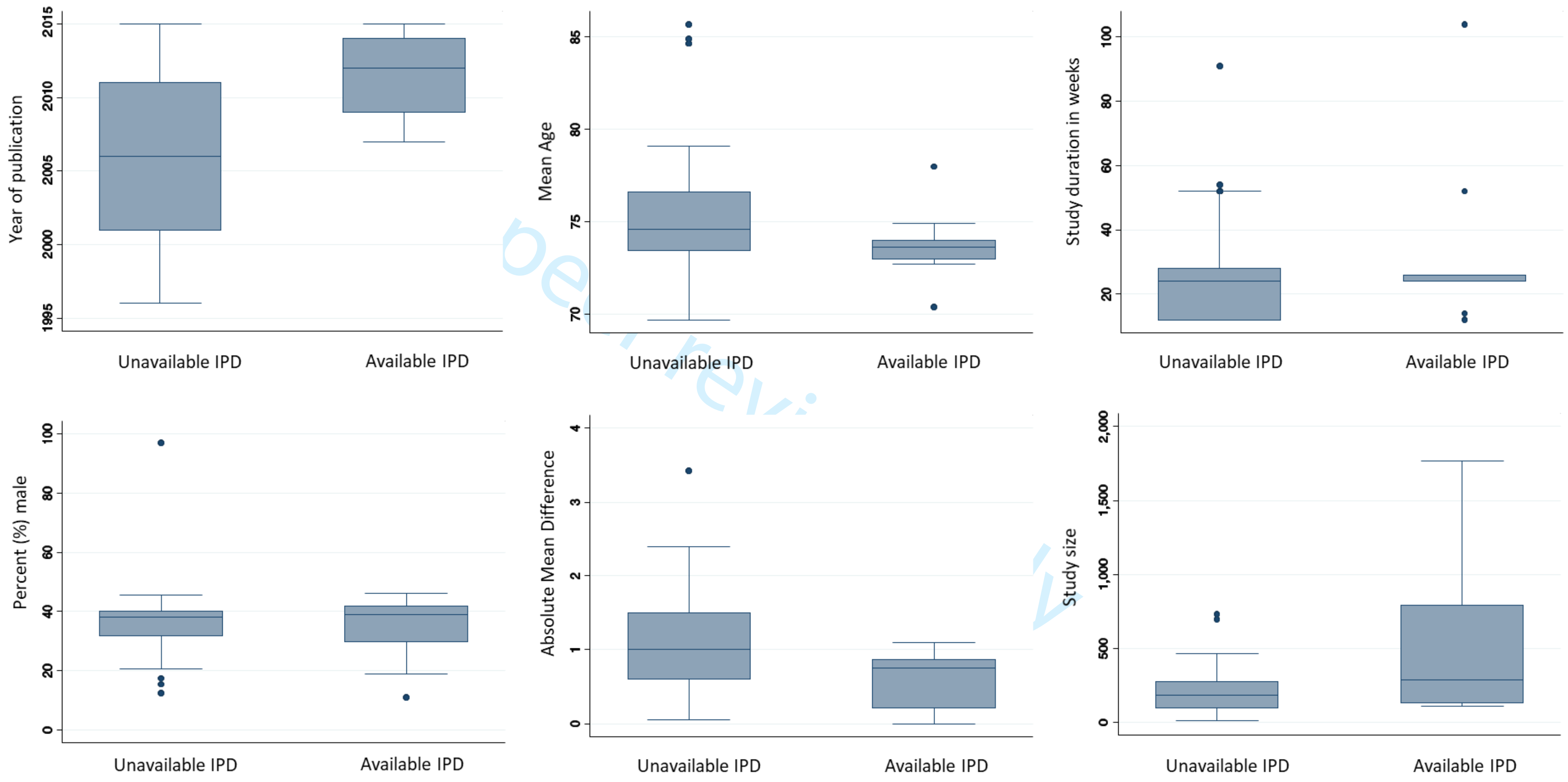

For peer review only - http://bmjopen.bmj.com/site/about/guidelines.xhtml

Appendix 8: Cochrane Risk-of-bias appraisal results (n = 80)

Study	1. Random sequence generation	2. Allocation concealment	3. Blinding of participants and personnel	4. Blinding of outcome assessment	5. Incomplete outcome data	6. Selective reporting	7. Other bias*
Agid, 1998	Low	High	Low	Unclear	High	Unclear	High
Ancoli-Israel, 2005	Unclear	Unclear	Unclear	Unclear	High	Unclear	High
Andersen, 2012	Unclear	Low	Low	Low	High	Low	Low
Araki, 2014	Low	Unclear	Unclear	Unclear	High	Unclear	Unclear
Bakchine, 2008	Low	Low	Low	Low	Low	High	High
Black, 2007	Low	Low	Low	Low	Low	Unclear	High
Blesa Gonzalez, 2011	Unclear	Unclear	High	Unclear	High	Low	High
Burns, 1999	Unclear	Unclear	Unclear	Unclear	High	Unclear	High
Burns, 2009	Low	Low	Low	Low	Low	Unclear	High
Burns, 2011	Low	Unclear	Low	Low	High	Unclear	Unclear
Choi, 2011	Unclear	Unclear	High	High	High	Low	Low
Corey-Bloom, 1998	Low	Low	Low	Low	High	Unclear	High
Cretu, 2008	Unclear	Unclear	Unclear	Low	Unclear	Unclear	Unclear
Dysken, 2014	Low	Low	Low	Unclear	Low	Low	Low
Farlow, 2013	Low	Unclear	Low	Low	High	Unclear	High
Feldman, 2001	Low	Unclear	Low	Low	High	Unclear	High
Feldman, 2007	Low	Low	Low	Low	High	Unclear	High
Fox, 2012	Low	Low	High	Low	High	High	Unclear
Frolich, 2011	Unclear	Unclear	Low	Low	High	Low	High
Fuschillo, 2001	Unclear	Unclear	Unclear	Unclear	Low	Low	Unclear
Gault, 2015	Low	Low	Low	Unclear	Low	Low	High
Gold, 2010	Low	Unclear	Low	Low	High	Low	High
Greenberg, 2000	Low	Low	Low	Unclear	High	Low	Low
Grossberg, 2013	Low	Low	Low	Low	High	Low	High
Hager K, 2014	Low	Low	Low	Low	High	High	High
Haig, 2014	Low	Low	Low	Low	High	Low	High
Hernández, 2007	Low	Low	Low	Low	Unclear	Low	Low
Herrmann, 2013	Low	Low	Low	Low	High	Low	High
Holmes, 2004	Low	Unclear	Low	Low	High	Low	High
Homma, 1998	Low	Low	Low	Low	Low	Unclear	High
Homma, 2008	Low	Low	Low	Low	High	Unclear	Unclear
Hong, 2006	Unclear	Unclear	Unclear	Unclear	Low	Unclear	Unclear
Howard, 2007	Low	Low	Low	Low	Low	Unclear	Low
Howard, 2012	Low	Low	Low	Low	High	Low	Low
Hu, 2006	Unclear	Unclear	Unclear	Unclear	Low	Unclear	Unclear
Johannsen, 2006	Unclear	Unclear	Low	Low	Low	Unclear	High
Jones, 2004	Low	Unclear	Unclear	Low	Low	Unclear	High
Kadir, 2008	Unclear	Unclear	Unclear	Unclear	High	Unclear	High
Kano, 2013	Unclear	Unclear	Unclear	Unclear	Low	Unclear	Unclear
Karaman, 2005	Low	Unclear	Low	Low	Unclear	Unclear	Unclear
Likitjaroen, 2012	Low	Low	Low	Unclear	High	High	Unclear
Lorenzi, 2011	Unclear	Unclear	Unclear	Unclear	Unclear	Unclear	High
Maher-Edwards, 2011	Low	Unclear	Unclear	Unclear	High	Unclear	High
Marek, 2014	Low	Low	Low	Low	High	Low	High
Mazza, 2006	Low	Unclear	Low	Low	High	Unclear	Unclear
Mohs, 2001	Low	Low	Low	Low	High	Unclear	High
Moretti, 2014	Unclear	Unclear	Unclear	Unclear	Low	Unclear	Low
Mowla, 2007	Low	Unclear	Low	Unclear	High	Unclear	Unclear
Nakamura, 2011	Unclear	Low	Low	Low	Low	Low	High
Nakano, 2001	Unclear	Unclear	Unclear	Unclear	Unclear	Unclear	Unclear
Nordberg, 2009	Unclear	Unclear	High	High	Unclear	Unclear	High
Pakdaman H, 2015	Low	Unclear	High	High	High	Unclear	Unclear
Peng, 2005	Unclear	Unclear	Unclear	Unclear	Low	Unclear	Unclear
Peskind, 2006	Low	Low	Low	Unclear	Low	Unclear	High
Peters, 2015	Unclear	Unclear	Low	Low	High	Low	Low
Reisberg, 2003	Low	Unclear	Low	Unclear	High	Low	Unclear
Rockwood, 2001	Low	Low	Low	Low	Unclear	Low	High
Rockwood, 2006	Low	Low	Low	Low	Low	Unclear	Unclear
Rogers, 1996	Unclear	Unclear	Low	Unclear	Low	Unclear	Unclear
Rogers, 1998	Unclear	Unclear	Low	Low	Low	Unclear	High
Rogers, 1998	Low	Unclear	Low	Unclear	High	Unclear	High
Saxton, 2012	Low	Low	Low	Low	Low	Low	High
Scarpini, 2011	Low	Low	Low	Unclear	High	Unclear	High
Schmidt, 2008	Low	Low	Low	Low	High	Unclear	High
Seltzer, 2004	Low	Unclear	Unclear	Unclear	Unclear	Unclear	High

For peer review only - http://bmjopen.bmj.com/site/about/guidelines.xhtml

Shao, 2015	Unclear	Unclear	Unclear	Unclear	Low	Unclear	Unclear
Shimizu, 2015	Low	Unclear	High	Low	High	Unclear	Unclear
Sole-Padulles, 2013	Unclear	Unclear	Unclear	Unclear	Low	Unclear	Unclear
Tariot, 2000	Low	Unclear	Low	Low	High	Low	High
Tariot, 2001	Low	Low	Low	Low	Unclear	Unclear	High
Thomas, 2001	Low	Unclear	Unclear	Unclear	Unclear	Unclear	Unclear
Wilcock, 2003	Unclear	Unclear	Unclear	Unclear	Unclear	Unclear	Unclear
Wilkinson, 2001	Low	Low	Low	Low	High	Unclear	High
Wilkinson, 2002	Low	Low	Low	Low	High	Unclear	High
Wilkinson, 2012	Low	High	Low	Low	High	Low	High
Winblad, 2001	Low	Unclear	Unclear	Low	High	Unclear	High
Winblad, 2006	Low	Low	Low	Low	High	Low	High
Winblad, 2007	Low	Low	Low	Low	High	Unclear	High
Yi, 2005	Unclear	Unclear	Unclear	Unclear	Low	Unclear	Unclear
Zhang, 2012	Unclear	Unclear	Unclear	Unclear	High	Unclear	High

* Other bias was categorized as:
a) *low risk of bias* when the study appeared to be free of other sources of bias,
b) *high risk of bias* when there was at least one important risk of bias. For example, when the study had:

- A potential source of bias related to the specific study design used; or
- A conflict of interest related to funding source; or
- An author was an employee of the drug company that sponsored the study; or
- Been claimed to have been fraudulent; or
- Other potential biases.

c) *unclear risk of bias* when there was a potential for bias, but there was either:

- Insufficient information to assess whether an important risk of bias exists; or
- Insufficient rationale/evidence that an identified problem would introduce bias; or
- Funding by drug company, but conflicts were not described

For peer review only - http://bmjopen.bmj.com/site/about/guidelines.xhtml

Appendix 9: Overall risk of bias for studies with shared IPD against (a) all remaining studies and (b) studies for which sponsors claimed unavailable IPD. AD: aggregate data; IPD: individual patient data

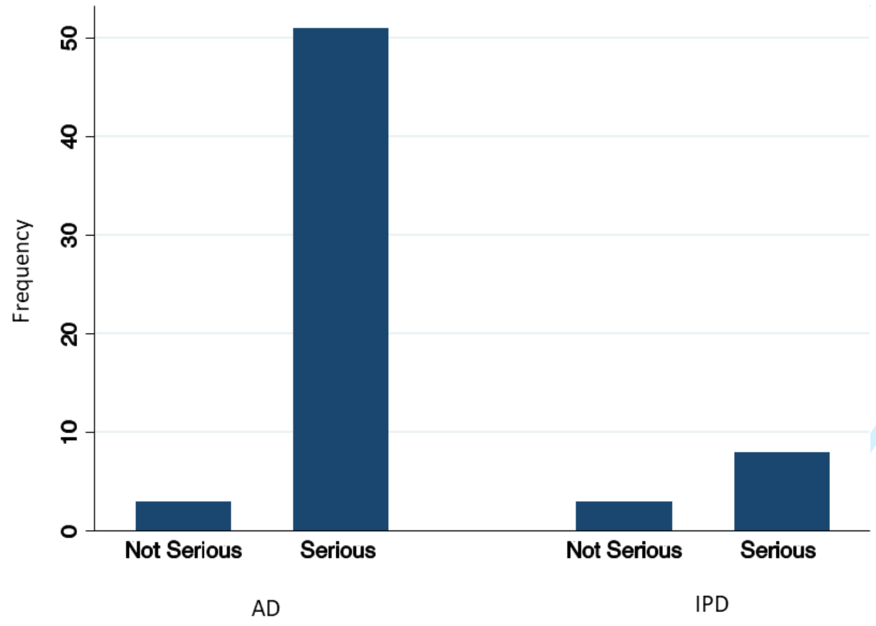

a. Comparison of studies with shared IPD with all remaining studies (irrespective type of sponsor)

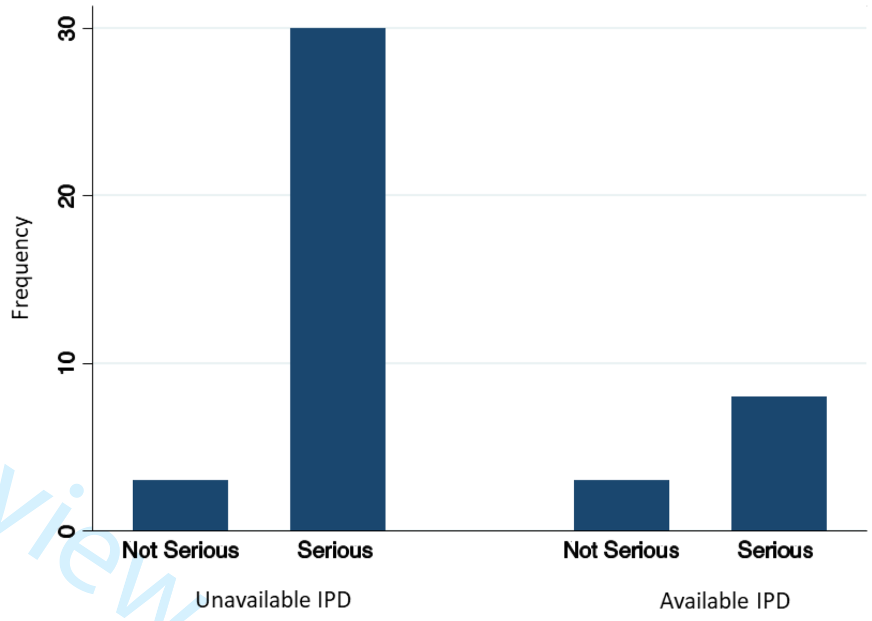

b. Comparison of studies with available and unavailable IPD (industry-sponsored studies only)

For peer review only - http://bmjopen.bmj.com/site/about/guidelines.xhtml

Appendix 10: Study-specific effect sizes calculated from shared IPD and published data. IPD: individual patient data

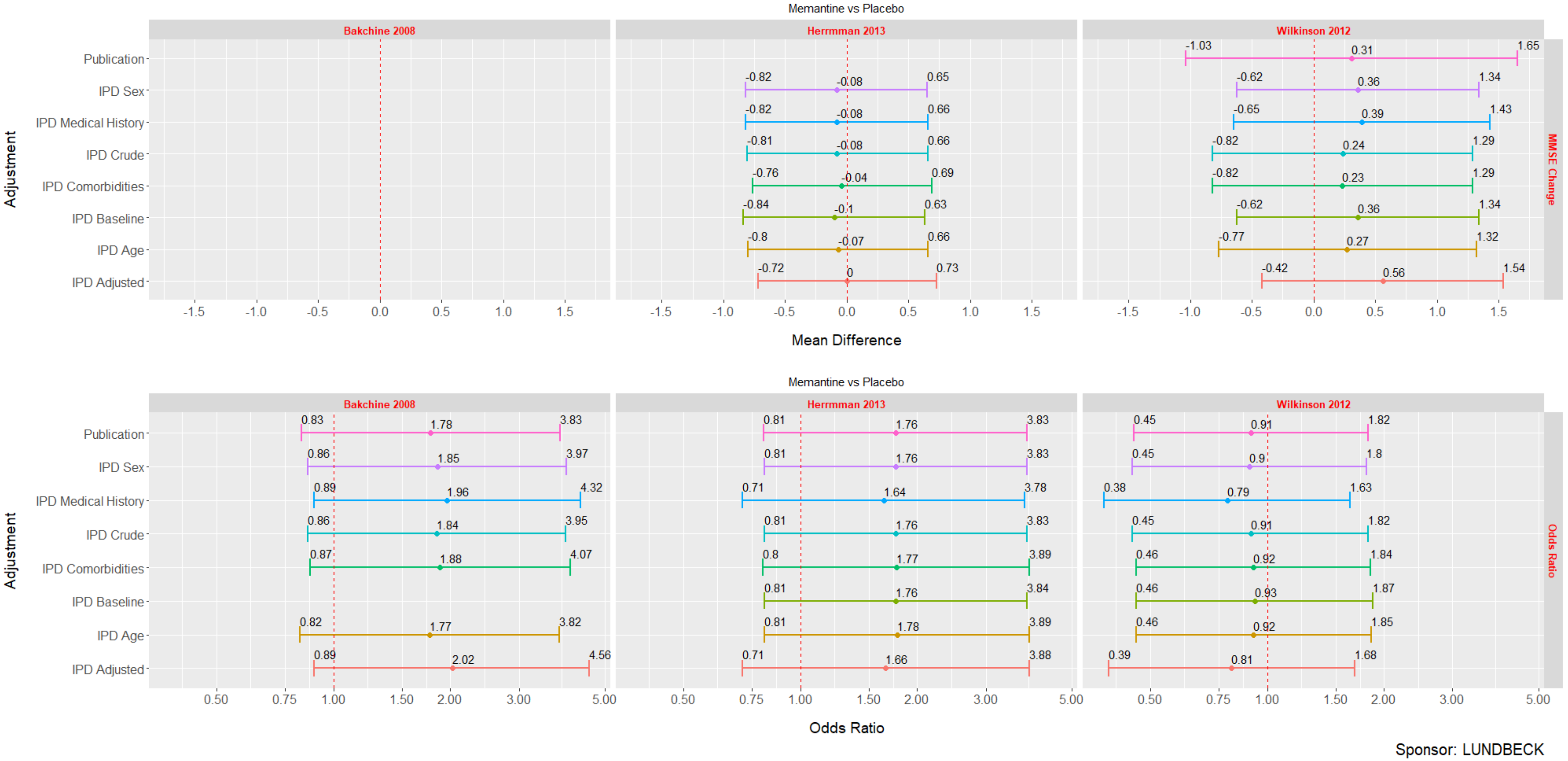

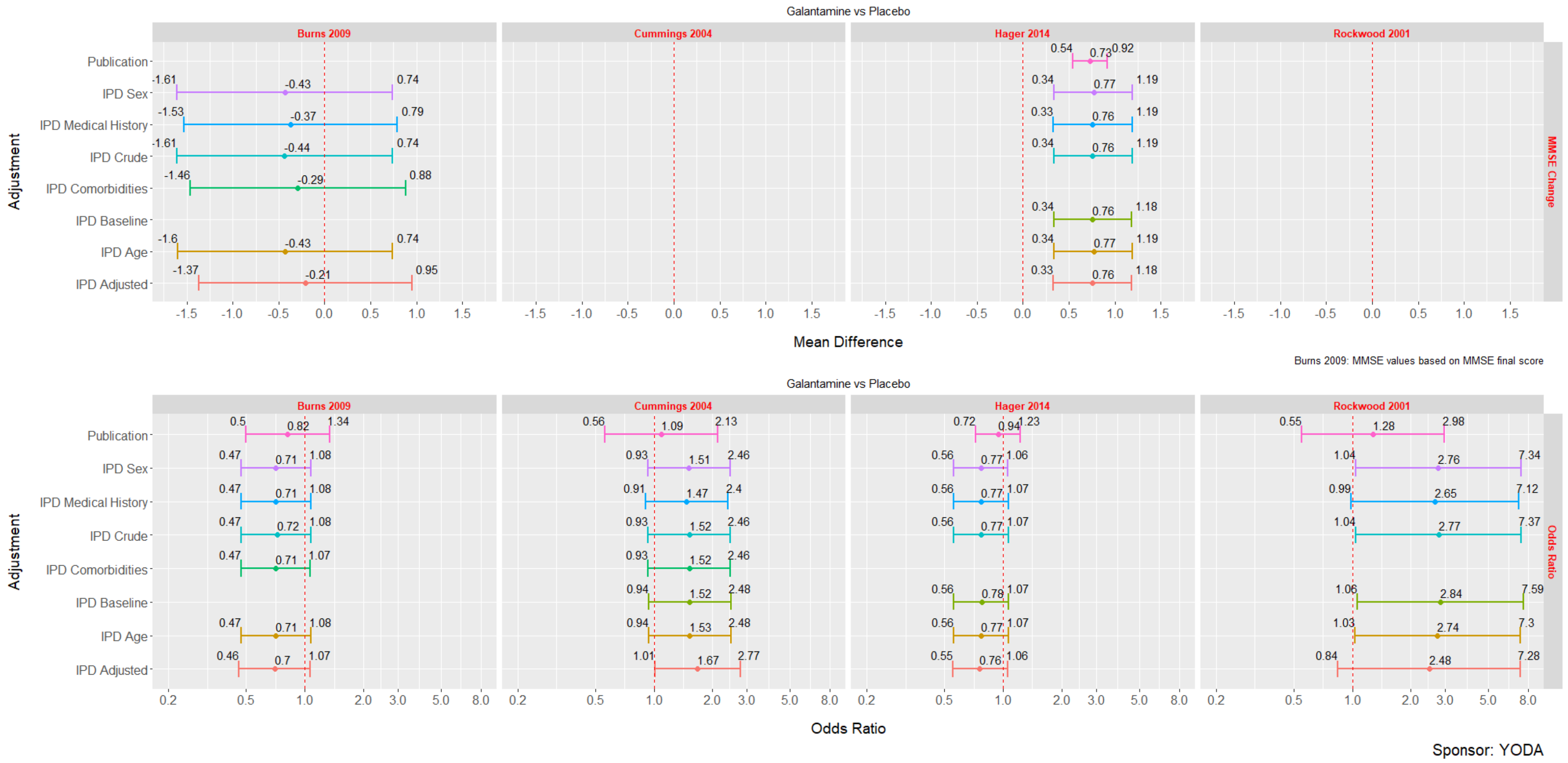
Galantamine vs Placebo
Burns 2009
Cummings 2004
Hager 2014
Rockwood 2001
Adjustment
Publication
IPD Sex
IPD Medical History
IPD Crude
IPD Comorbidities
IPD Baseline
IPD Age
IPD Adjusted
MMSE Change
Mean Difference
Burns 2009: MMSE values based on MMSE final score
Galantamine vs Placebo
Odds Ratio
Sponsor: YODA

33

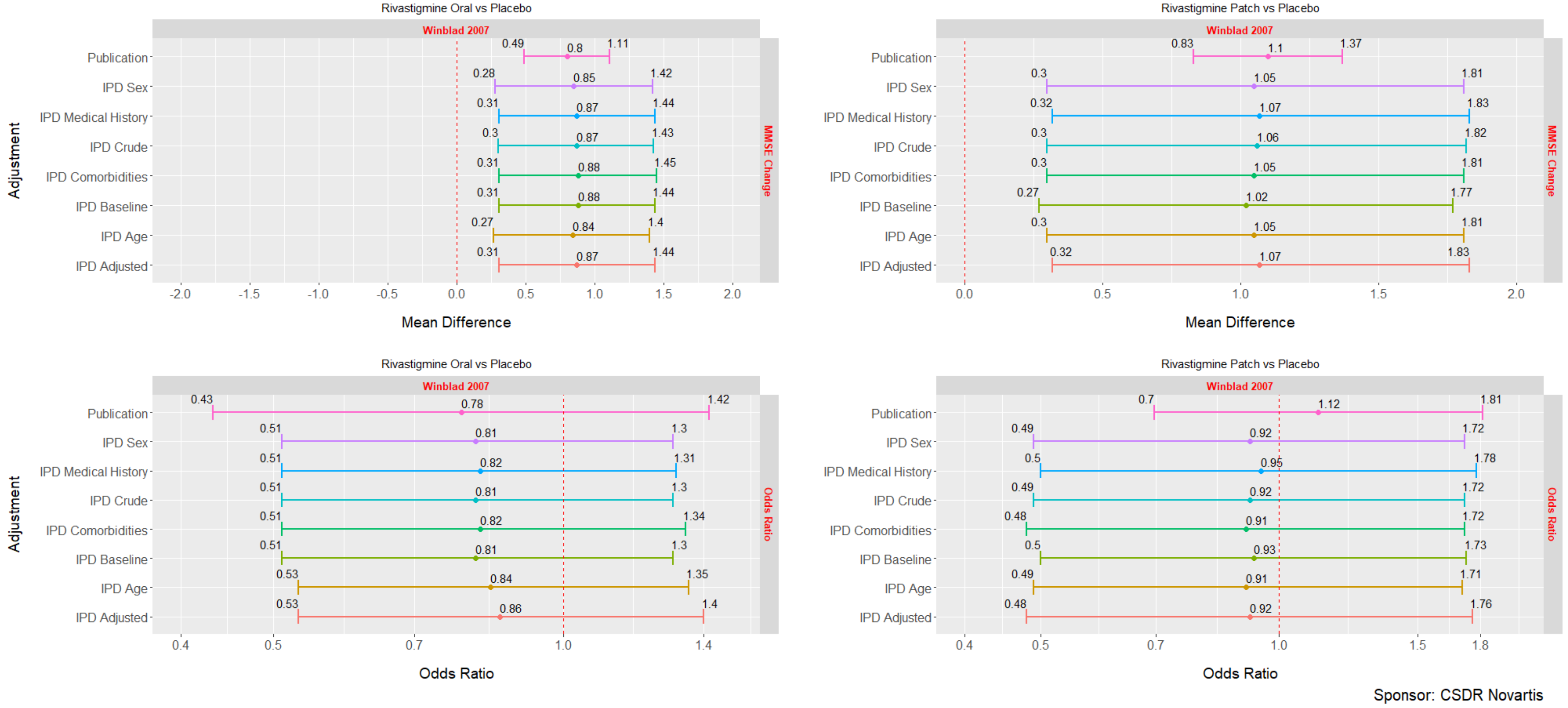
Rivastigmine Oral vs Placebo
Winblad 2007
MMSE Change
Adjustment
Publication
IPD Sex
IPD Medical History
IPD Crude
IPD Comorbidities
IPD Baseline
IPD Age
IPD Adjusted
0.49 0.8 1.11
0.28 0.85 1.42
0.31 0.87 1.44
0.3 0.87 1.43
0.31 0.88 1.45
0.31 0.88 1.44
0.27 0.84 1.4
0.31 0.87 1.44
-2.0 -1.5 -1.0 -0.5 0.0 0.5 1.0 1.5 2.0
Mean Difference
Rivastigmine Patch vs Placebo
Winblad 2007
MMSE Change
0.83 1.1 1.37
0.3 1.05 1.81
0.32 1.07 1.83
0.3 1.06 1.82
0.3 1.05 1.81
0.27 1.02 1.77
0.3 1.05 1.81
0.32 1.07 1.83
0.0 0.5 1.0 1.5 2.0
Mean Difference
Rivastigmine Oral vs Placebo
Winblad 2007
Odds Ratio
0.43 0.78 1.42
0.51 0.81 1.3
0.51 0.82 1.31
0.51 0.81 1.3
0.51 0.82 1.34
0.51 0.81 1.3
0.53 0.84 1.35
0.53 0.86 1.4
0.4 0.5 0.7 1.0 1.4
Odds Ratio
Rivastigmine Patch vs Placebo
Winblad 2007
Odds Ratio
0.7 1.12 1.81
0.49 0.92 1.72
0.5 0.95 1.78
0.49 0.92 1.72
0.48 0.91 1.72
0.5 0.93 1.73
0.49 0.91 1.71
0.48 0.92 1.76
0.4 0.5 0.7 1.0 1.5 1.8
Odds Ratio
Sponsor: CSDR Novartis

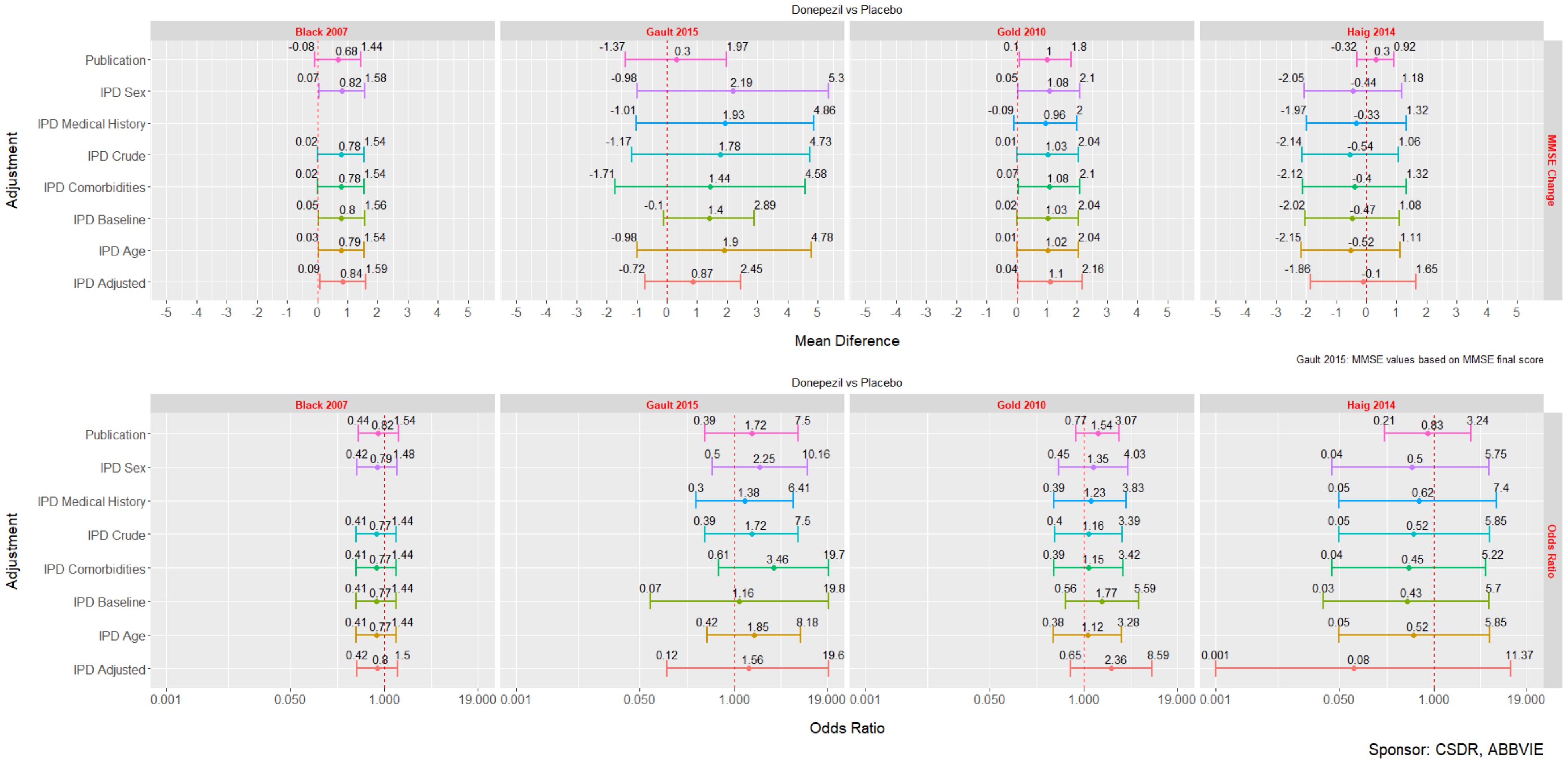

CSDR includes studies sponsored by GlaxoSmithKline, Eisai, Novartis, whereas YODA includes studies sponsored by Janssen

We also calculated the odds ratio for patients experiencing at least one AE excluding missing participants as shown in the MMSE outcome: Gold 2010: OR 2.78, 95% CI: 0.63-12.25; Black 2007: OR 1.19, 95% CI: 0.08-17.96; Winbland 2007: rivastigmine oral, OR 1.28, 95% CI: 0.09-18.16, rivastigmine patch, OR 0.81, 95% CI: 0.02-33.59; Wilkinson 2012: OR 0.84, 95% CI: 0.38-1.86; Herrmman 2013: OR 1.70, 95% CI: 0.71-4.08; Bachine 2008: OR 1.83, 95% CI: 0.77-4.32.

We were unable to assess this for studies obtained through YODA and AbbVie, since at the time of this assessement we did not have access to these data.

Abbreviations: IPD sex, regression analysis adjusting for sex; IPD medical history, regression analysis adjusting for medical history; IPD crude, analysis with no adjustments; IPD comorbidities, regression analysis adjusting for comorbidities; IPD baseline, regression analysis adjusting for MMSE baseline; IPD age, regression analysis adjusting for age; IPD adjusted, regression analysis adjusting for all available variables (we only considered those that we initially requested from sponsor)

For peer review only

For peer review only - http://bmjopen.bmj.com/site/about/guidelines.xhtml

Appendix 11: Correlation between participant age and dropout in studies with IPD. IPD: individual patient data

	Study*	Correlation	P-Value
**CSDR**	Black 2007 (EISAI)	0.079	0.147
	Gold 2010 (GSK)	0.141	0.072
	Winblad 2007 (Novartis)	0.016	0.584
**Lundbeck**	Wilkinson 2012	0.066	0.273
	Herrmman 2013	0.124	0.017

* We were unable to assess this correlation for studies obtained through YODA and AbbVie, since at the time of this assessment we did not have access to these data

For peer review only

For peer review only - http://bmjopen.bmj.com/site/about/guidelines.xhtml

Appendix 12: Comparison Adjusted Funnel plot (all treatments vs placebo)

(a) MMSE

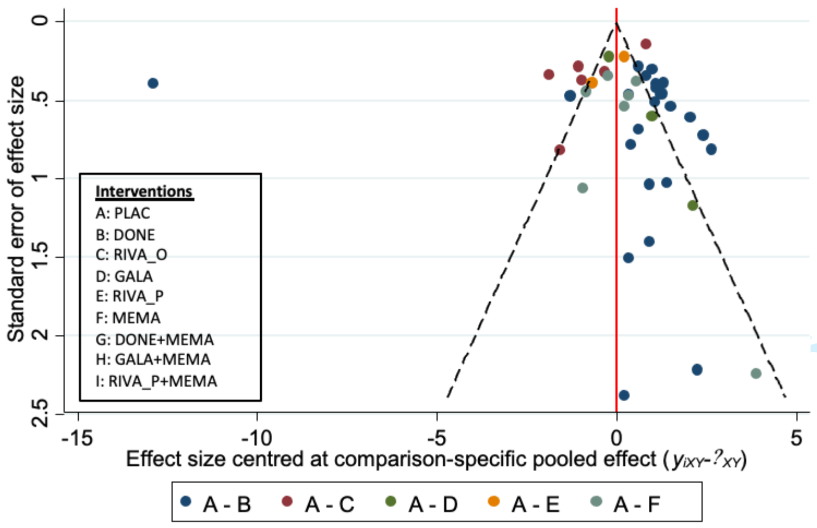

Note: Comparisons including only one study (when present) have been excluded

(b) Adverse Events

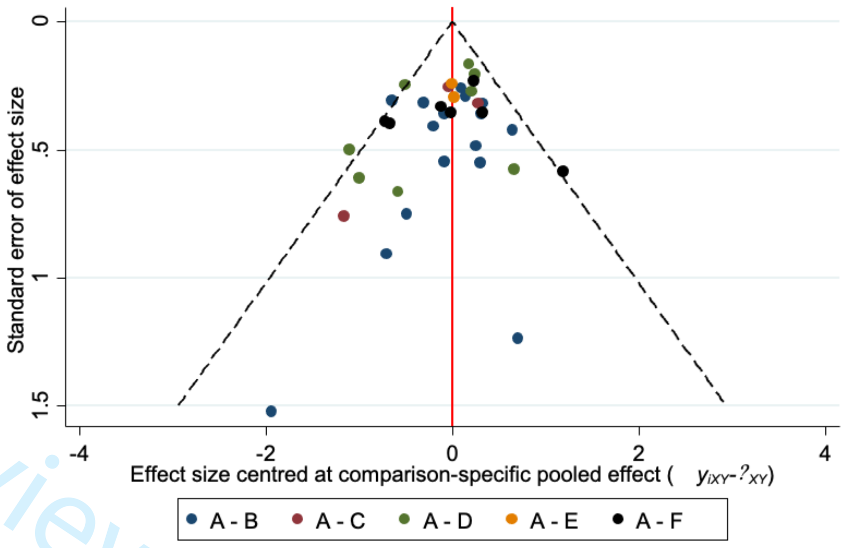

Note: Comparisons including only one study (when present) have been excluded

For peer review only - http://bmjopen.bmj.com/site/about/guidelines.xhtml

Appendix 13: Distribution of potential effect modifiers per treatment comparison and outcome

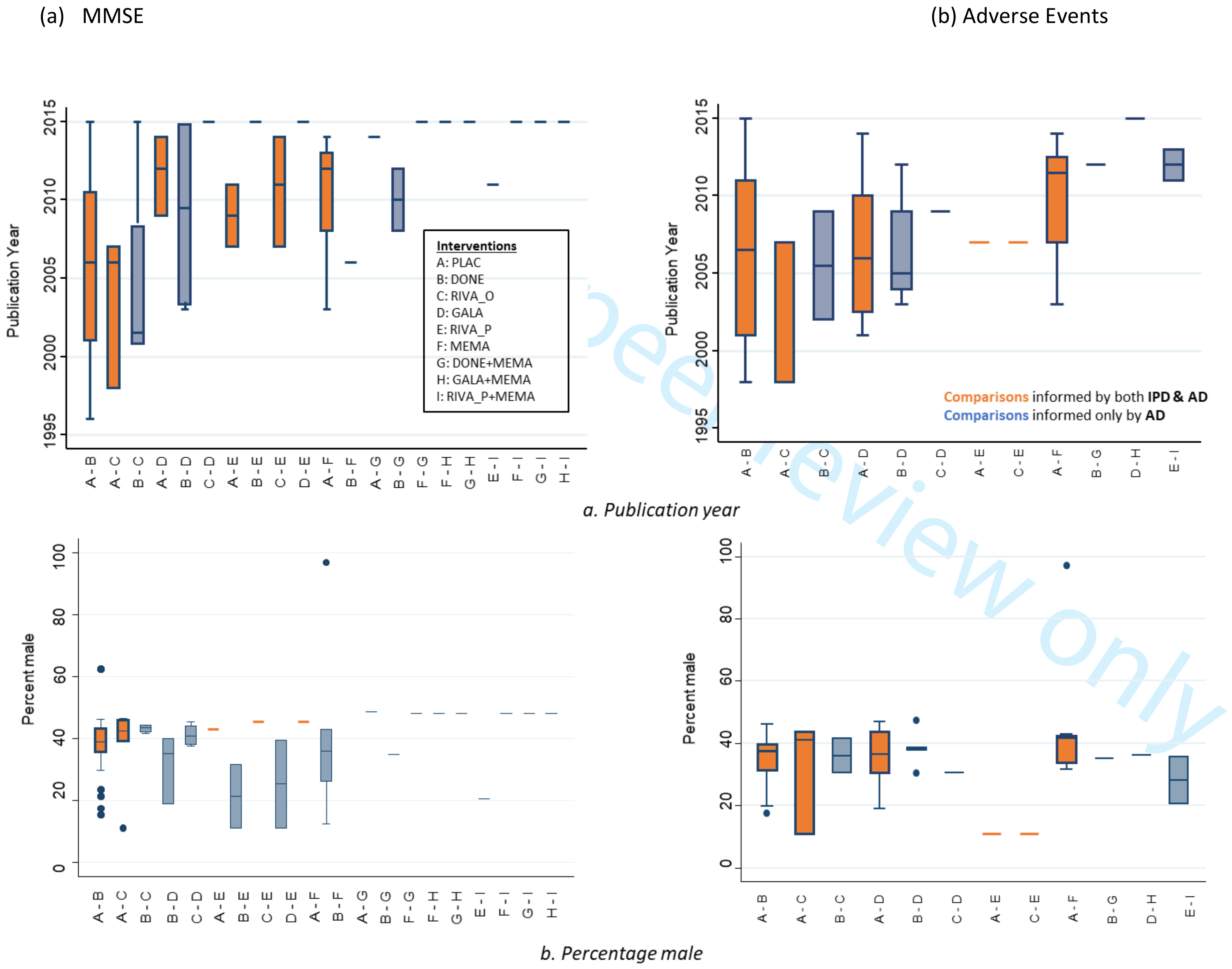

39

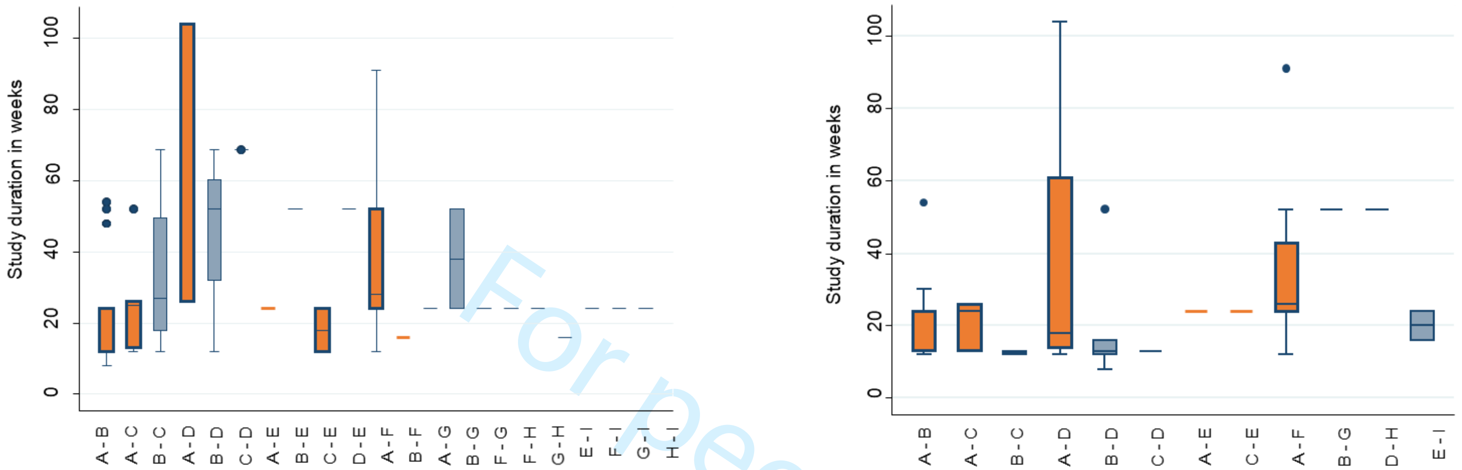

c. Study duration

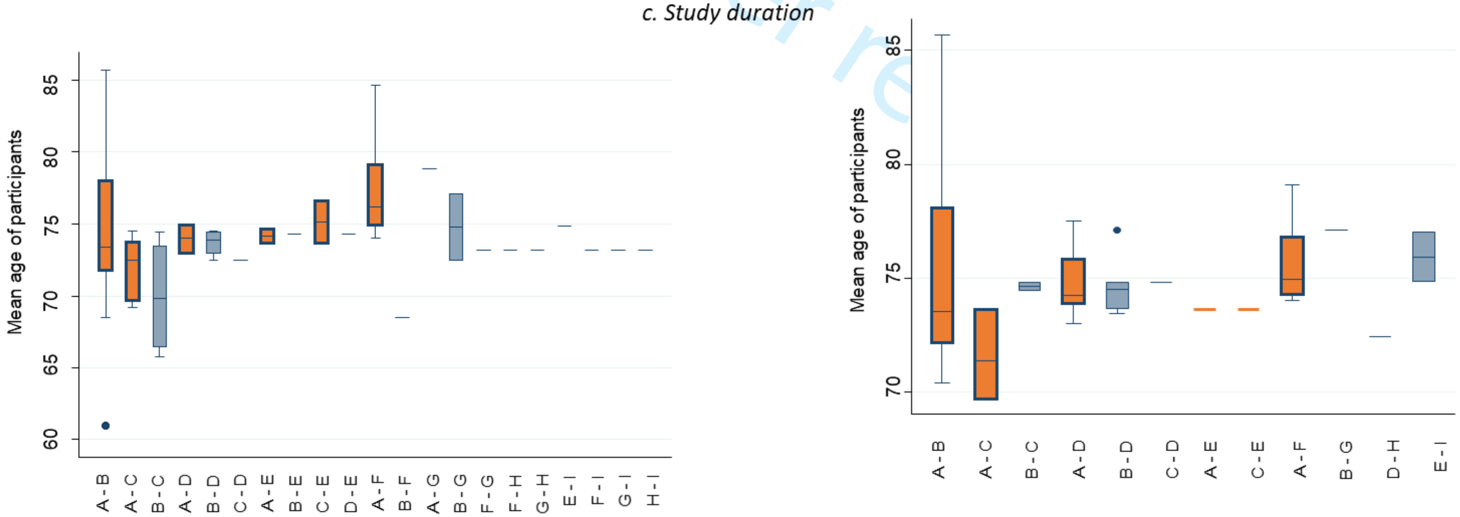

d. Mean participant age

For peer re

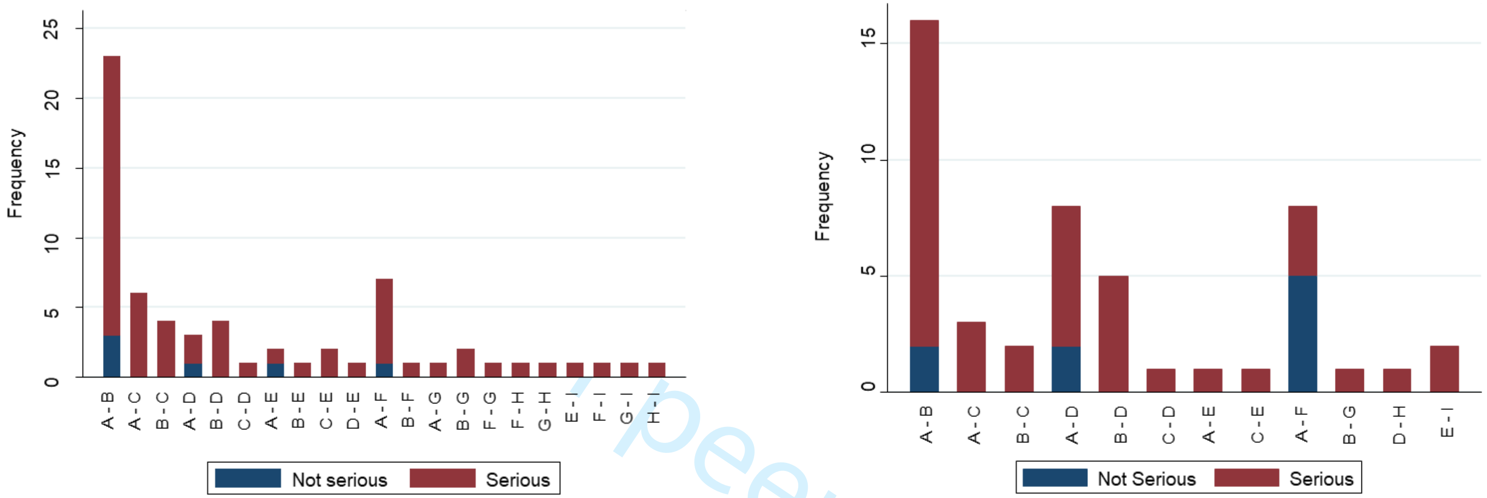

e. Overall Risk of Bias

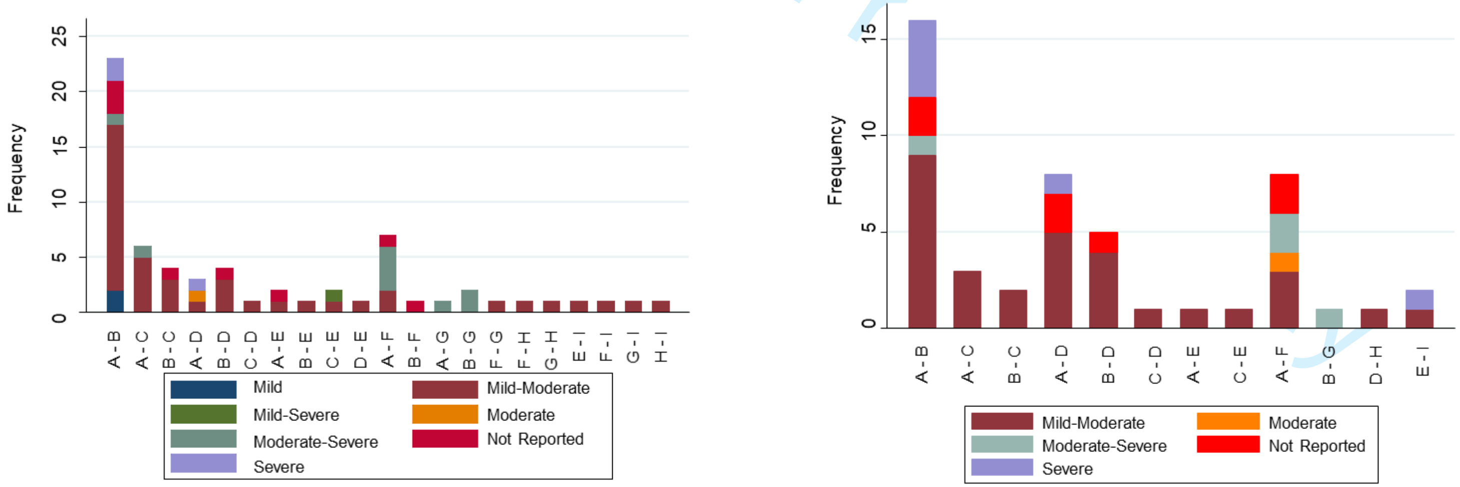

f. Alzheimer's Dementia Severity

For peer review only - http://bmjopen.bmj.com/site/about/guidelines.xhtml

Appendix 14: Consistency Assessment – Loop-specific approach (using adjusted treatment effects)

(a) MMSE

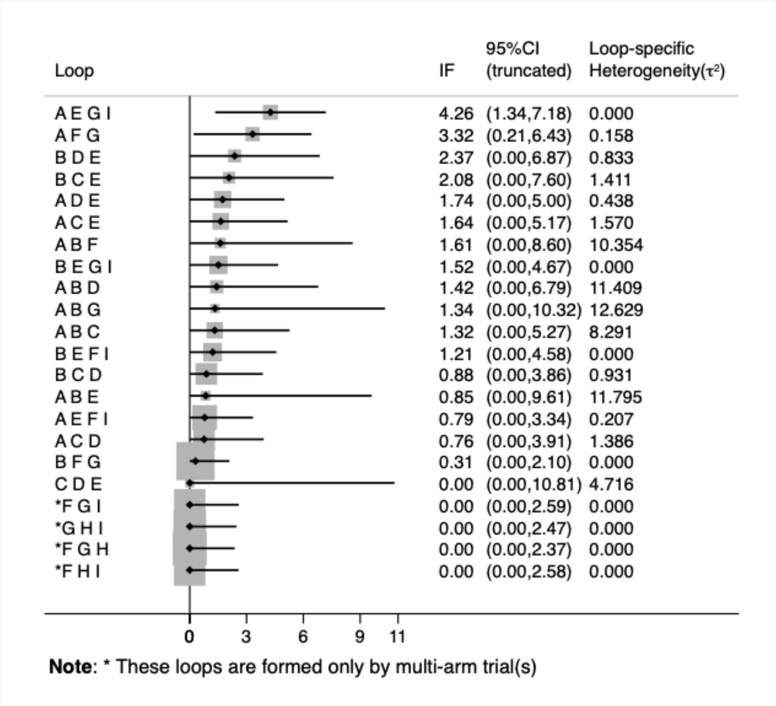

Design-by-treatment interaction model:
χ^2 statistic: 4.36, 13 degrees of freedom, P value: 0.987, between-study variance: 7.34. I^2 statistic=96%

(b) Adverse Events

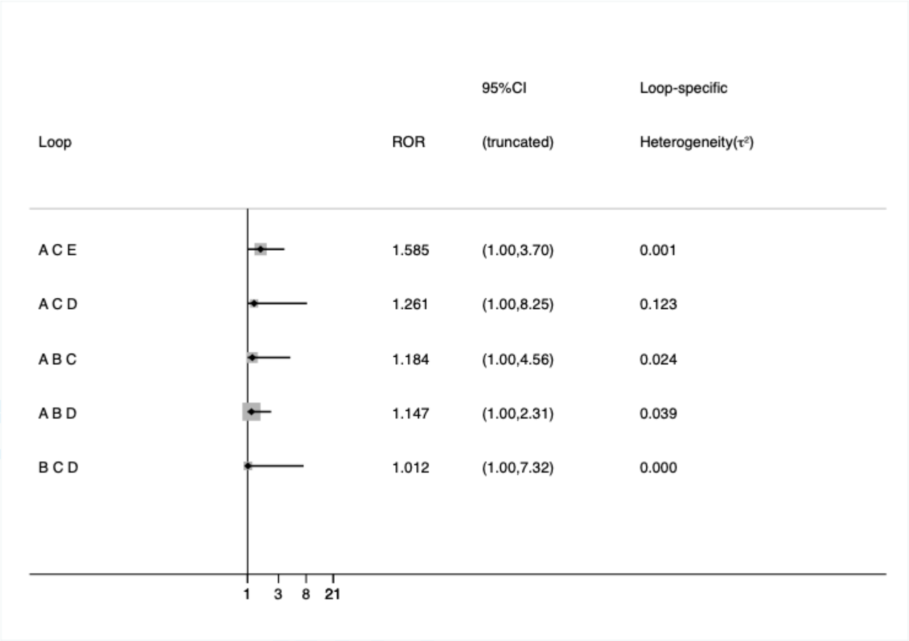

Design-by-treatment interaction model:
χ^2 statistic: 3.57, 6 degrees of freedom, P value: 0.735, between-study variance: 0.06. I^2 statistic=22%

For peer review only - http://bmjopen.bmj.com/site/about/guidelines.xhtml

Appendix 15: Network and standard meta-analysis results

Treatment Comparison	NMA estimate	95% CI	95% PI	P-score	MA estimate	95% CI	95% PI	#studies
**Mini-Mental State Examination (MMSE)*†**								
Donepezil vs Placebo	1.41	0.51 to 2.32	-3.48 to 6.31	0.59	1.65	0.16 to 3.14	-6.02 to 9.32	24
Rivastigmine oral vs Placebo	0.69	-0.79 to 2.18	-4.35 to 5.74	0.36	0.60	-0.43 to 1.62	-3.07 to 4.26	6
Galantamine vs Placebo	0.41	-1.44 to 2.26	-4.76 to 5.58	0.28	0.04	-1.09 to 1.17	-12.39 to 12.47	3
Rivastigmine transdermal vs Placebo	2.11	-0.04 to 4.26	-3.18 to 7.40	0.72	0.56	-0.33 to 1.45	--	2
Memantine vs Placebo	0.67	-0.99 to 2.34	-4.43 to 5.78	0.35	0.52	0.03 to 1.01	-0.69 to 1.73	7
Donepezil + Memantine vs Placebo	2.57	0.07 to 5.07	-2.88 to 8.02	0.80	4.21	1.94 to 6.48	--	1
Galantamine + Memantine vs Placebo	2.24	-2.13 to 6.61	-4.33 to 8.81	0.66				
Rivastigmine transdermal + Memantine vs Placebo	1.79	-1.70 to 5.27	-4.20 to 7.78	0.60				
Placebo (reference)				0.14				
Rivastigmine transdermal vs Rivastigmine oral	1.41	-0.80 to 3.62	-3.90 to 6.73		2.26	-0.48 to 4.99	-30.56 to 35.07	3
Rivastigmine oral vs Donepezil	-0.72	-2.28 to 0.84	-5.79 to 4.35		0.16	-0.57 to 0.90	-1.45 to 1.77	4
Galantamine vs Rivastigmine oral	-0.29	-2.48 to 1.91	-5.60 to 5.02		0.06	-1.05 to 1.17		1
Rivastigmine transdermal vs Donepezil	0.69	-1.52 to 2.91	-4.62 to 6.01		-0.20	-2.78 to 2.38		1
Rivastigmine transdermal vs Galantamine	1.70	-0.93 to 4.33	-3.81 to 7.21		2.20	-0.19 to 4.59		1
Rivastigmine transdermal + Memantine vs Rivastigmine transdermal	-0.32	-3.82 to 3.18	-6.32 to 5.68		-0.40	-1.40 to 0.60		1
Memantine vs Donepezil	-0.74	-2.56 to 1.08	-5.90 to 4.42		0.20	0.88 to 1.28		1
Donepezil + Memantine vs Donepezil	1.15	-1.33 to 3.64	-4.29 to 6.59		0.88	0.64 to 1.11		2
Galantamine vs Donepezil	-1.01	-2.86 to 0.84	-6.18 to 4.16		-0.35	-1.52 to 0.83	-5.31 to 4.62	4
Donepezil + Memantine vs Memantine	1.89	-0.88 to 4.67	-3.69 to 7.48		0.37	-1.04 to 1.78		1
Galantamine + Memantine vs Memantine	1.57	-2.78 to 5.92	-4.98 to 8.12		0.82	-0.58 to 2.22		1

For peer review only

For peer review only - http://bmjopen.bmj.com/site/about/guidelines.xhtml

Rivastigmine transdermal + Memantine vs Memantine	1.12	-2.47 to 4.70	-4.93 to 7.16		0.41	-1.17 to 1.99		1
Galantamine + Memantine vs Donepezil + Memantine	-0.33	-4.72 to 4.06	-6.91 to 6.23		0.45	-0.85 to 1.75		1
Rivastigmine transdermal + Memantine vs Donepezil + Memantine	-0.78	-4.53 to 2.97	-6.93 to 5.38		0.04	-1.45 to 1.53		1
Rivastigmine transdermal + Memantine vs Galantamine + Memantine	-0.45	-5.05 to 4.14	-7.18 to 6.28		-0.41	-1.89 to 1.07		1
Common within-network between-study variance τ^2 = 5.75, I^2 = 96% (96%, 97%)								
Design-by-treatment interaction model for inconsistency χ^2 *(d.f., P-value,* τ^2*)*: 4.36 (13, 0.987, 7.35)								
**Adverse Events (AEs)*‡**								
Donepezil vs Placebo	1.08	0.87 to 1.35	0.67 to 1.75	0.30	1.07	0.88 to 1.31	0.84 to 1.37	16
Rivastigmine oral vs Placebo	1.26	0.82 to 1.94	0.69 to 2.33	0.16	1.26	0.75 to 2.12	0.01 to 161.35	3
Galantamine vs Placebo	0.95	0.74 to 1.22	0.58 to 1.55	0.53	1.02	0.71 to 1.46	0.38 to 2.77	8
Rivastigmine transdermal vs Placebo	0.90	0.58 to 1.42	0.48 to 1.69	0.57	0.86	0.53 to 1.40		1
Memantine vs Placebo	0.88	0.64 to 1.20	0.52 to 1.49	0.63	0.87	0.63 to 1.20	0.38 to 1.99	8
Donepezil + Memantine vs Placebo	0.77	0.34 to 1.73	0.30 to 1.96	0.69				
Galantamine + Memantine vs Placebo	1.03	0.45 to 2.39	0.39 to 2.70	0.43				
Rivastigmine transdermal + Memantine vs Placebo	0.72	0.32 to 1.59	0.28 to 1.81	0.75				
Placebo (reference)				0.44				
Rivastigmine oral Donepezil vs	1.17	0.73 to 1.87	0.61 to 2.22		2.08	0.21 to 20.73		2
Galantamine vs Donepezil	0.88	0.64 to 1.19	0.52 to 1.49		0.79	0.46 to 1.39	0.32 to 1.96	5
Donepezil + Memantine vs Donepezil	0.71	0.33 to 1.55	0.29 to 1.76		0.71	0.37 to 1.38		1
Rivastigmine transdermal vs Rivastigmine oral	0.72	0.42 to 1.23	0.36 to 1.44		0.94	0.52 to 1.68		1
Rivastigmine transdermal + Memantine vs Rivastigmine transdermal	0.79	0.41 to 1.54	0.36 to 1.77		0.79	0.45 to 1.39		2
Galantamine vs Rivastigmine oral	0.75	0.46 to 1.22	0.39 to 1.45		0.63	0.15 to 2.64		1

For peer review only

For peer review only - http://bmjopen.bmj.com/site/about/guidelines.xhtml

Galantamine + Memantine vs Galantamine	1.09	0.49 to 2.42	0.43 to 2.75	1.09	0.55 to 2.17	1
Common within-network between-study variance τ2 = 0.04, I^2 = 22% (0%, 48%)						
Design-by-treatment interaction model for inconsistency χ^2 *(d.f., P-value,* τ^2*)*: 3.57 (6, 0.735, 0.06)						

* Aggregate data and fully adjusted results from studies with available individual patient data were used in both meta-analysis and NMA. The mean difference effect size is presented for MMSE and the odds ratio for AE.
† MMSE: Studies with available IPD included only available participants –to assess the missing data impact on the second stage (IMDoM) a separate analysis was applied
‡ AE: Studies with available IPD included all randomized participants

For peer review only

Appendix 16: Network subgroup and meta-regression analysis results

Treatment Comparison	NMA estimate	95% CI	95%PI	P-score
**Mini-Mental State Examination (MMSE)†**				
**Mean Difference: Aggregate data and crude results from studies with available individual patient data**				
Donepezil vs Placebo	1.41	0.50 to 2.33	-3.51 to 6.34	0.59
Rivastigmine oral vs Placebo	0.69	-0.80 to 2.19	-4.38 to 5.76	0.36
Galantamine vs Placebo	0.37	-1.49 to 2.23	-4.82 to 5.57	0.28
Rivastigmine transdermal vs Placebo	2.10	-0.06 to 4.26	-3.22 to 7.42	0.72
Memantine vs Placebo	0.63	-1.05 to 2.30	-4.51 to 5.76	0.34
Donepezil + Memantine vs Placebo	2.56	0.04 to 5.07	-2.92 to 8.04	0.79
Galantamine + Memantine vs Placebo	2.22	-2.18 to 6.61	-4.39 to 8.82	0.66
Rivastigmine transdermal + Memantine vs Placebo	1.77	-1.73 to 5.27	-4.25 to 7.79	0.60
Placebo (reference)				0.14
Common within-network between-study variance τ^2 = 5.81, I^2 = 96% (96%, 97%)				
Design-by-treatment interaction model for inconsistency χ^2 *(d.f., P-value,* τ^2*)*: 4.42 (13, 0.986, 7.44)				
**Mean Difference: Aggregate data results****				
Donepezil vs Placebo	1.55	0.41 to 2.68	-4.16 to 7.25	0.57
Rivastigmine oral vs Placebo	0.71	-1.10 to 2.52	-5.18 to 6.60	0.34
Galantamine vs Placebo	0.57	-1.98 to 3.12	-5.61 to 6.74	0.32
Rivastigmine transdermal vs Placebo	2.60	-0.20 to 5.40	-3.69 to 8.89	0.75
Memantine vs Placebo	0.82	-1.37 to 3.01	-5.21 to 6.84	0.37
Donepezil + Memantine vs Placebo	2.71	-0.17 to 5.60	-3.62 to 9.04	0.76
Galantamine + Memantine vs Placebo	2.44	-2.61 to 7.48	-5.19 to 10.07	0.65
Rivastigmine transdermal + Memantine vs Placebo	2.09	-1.98 to 6.15	-4.89 to 9.07	0.61
Placebo (reference)				0.15
Common within-network between-study variance τ^2 = 7.66, I2 = 97% (96%, 97%)				
Design-by-treatment interaction model for inconsistency χ^2 *(d.f., P-value,* τ^2*)*: 3.92 (11, 0.972, 8.76)				
**Mean Difference: Crude results from studies with available individual patient data**				
Donepezil vs Placebo	0.70	0.01 to 1.40	-0.67 to 2.07	0.65
Rivastigmine oral vs Placebo	0.87	-0.01 to 1.75	-0.70 to 2.44	0.73
Galantamine vs Placebo	0.45	-0.24 to 1.14	-0.91 to 1.82	0.48
Rivastigmine transdermal vs Placebo	1.06	0.04 to 2.08	-0.67 to 2.79	0.82
Memantine vs Placebo	0.05	-0.74 to 0.83	-1.42 to 1.51	0.20
Placebo (reference)				0.13
Common within-network between-study variance τ^2 = 0.12, I^2 = 29% (0%, 71%)				
Design-by-treatment interaction model for inconsistency χ^2 *(d.f., P-value,* τ^2*)*: N/A (no closed loops)				
**Mean Difference: Low Risk of Bias for Allocation Concealment***				
Donepezil vs Placebo	2.02	-0.24 to 4.28	-6.19 to 10.23	0.70
Rivastigmine oral vs Placebo	1.38	-2.27 to 5.02	-7.39 to 10.14	0.57
Galantamine vs Placebo	-0.31	-4.61 to 3.98	-9.42 to 8.79	0.31
Rivastigmine transdermal vs Placebo	0.82	-4.08 to 5.72	-8.63 to 10.27	0.48
Memantine vs Placebo	0.69	-3.01 to 4.39	-8.10 to 9.49	0.46
Donepezil + Memantine vs Placebo	2.88	-4.75 to 10.51	-8.48 to 14.23	0.69
Placebo (reference)				0.30
Common within-network between-study variance: τ^2 = 13.82, I^2 = 98% (98%, 99%)				
Design-by-treatment interaction model for inconsistency χ^2 *(d.f., P-value,* τ^2*)*: 0.13 (3, 0.99, 19.10)				
**Mean Difference: Low risk of bias for Incomplete Data***				
Donepezil vs Placebo	0.87	0.07 to 1.66	-1.67 to 3.40	0.61
Rivastigmine oral vs Placebo	-1.52	-4.41 to 1.37	-5.54 to 2.50	0.10
Galantamine vs Placebo	0.52	-0.94 to 1.99	-2.36 to 3.41	0.48
Rivastigmine transdermal vs Placebo	1.37	-0.64 to 3.38	-1.91 to 4.65	0.71
Memantine vs Placebo	0.57	-1.12 to 2.27	-2.47 to 3.62	0.48
Donepezil + Memantine vs Placebo	0.94	-2.11 to 4.00	-3.23 to 5.11	0.57
Galantamine + Memantine vs Placebo	1.39	-1.66 to 4.44	-2.77 to 5.56	0.70
Rivastigmine transdermal + Memantine vs Placebo	0.98	-2.15 to 4.12	-3.26 to 5.23	0.58
Placebo (reference)				0.27
Common within-network between-study variance: τ^2 = 1.16, I^2 = 79% (65%, 88%)				
Design-by-treatment interaction model for inconsistency χ^2 *(d.f., P-value,* τ^2*)*: 12.15 (3, 0.007, 0.863)				
**Mean Difference: Publicly-Sponsored Studies***				
Donepezil vs Placebo	6.57	-4.68 to 17.81	-129.61 to 142.74	0.71
Rivastigmine oral vs Placebo	1.40	-16.41 to 19.21	-161.58 to 164.38	0.44
Memantine vs Placebo	0.11	-17.65 to 17.87	-162.64 to 162.86	0.39
Rivastigmine transdermal + Memantine vs Placebo	5.83	-7.98 to 19.64	-139.93 to 151.59	0.65
Placebo (reference)				0.32

For peer review only - http://bmjopen.bmj.com/site/about/guidelines.xhtml

Common within-network between-study variance: τ^2 = 81.93, I^2 = 99% (99%, 100%)				
Design-by-treatment interaction model for inconsistency χ^2 (d.f., P-value, τ^2): 0.05 (1, 0.815, 116.71)				
**Mean Difference: Industry-Sponsored Studies***				
Donepezil vs Placebo	0.98	0.69 to 1.27	0.10 to 1.86	0.85
Rivastigmine oral vs Placebo	0.82	0.35 to 1.29	-0.14 to 1.78	0.69
Galantamine vs Placebo	0.41	-0.15 to 0.96	-0.60 to 1.41	0.34
Rivastigmine transdermal vs Placebo	0.80	0.18 to 1.41	-0.25 to 1.84	0.67
Memantine vs Placebo	0.60	0.06 to 1.15	-0.39 to 1.60	0.50
Rivastigmine transdermal + Memantine vs Placebo	0.40	-1.02 to 1.81	-1.29 to 2.08	0.39
Placebo (reference)				0.06
Common within-network between-study variance: τ^2 = 0.16, I^2 = 43% (15%, 62%)				
Design-by-treatment interaction model for inconsistency χ^2 (d.f., P-value, τ^2): 8.06 (7, 0.327, 0.16)				
**Mean Difference: Studies with Mild to Moderate cognitive impairment, assessed with MMSE at baseline ***				
Donepezil vs Placebo	1.68	0.31 to 3.06	-4.81 to 8.18	0.69
Rivastigmine oral vs Placebo	0.88	-1.29 to 3.05	-5.85 to 7.61	0.51
Galantamine vs Placebo	0.31	-2.47 to 3.09	-6.66 to 7.28	0.40
Rivastigmine transdermal vs Placebo	2.74	-0.68 to 6.16	-4.53 to 10.01	0.81
Memantine vs Placebo	-0.58	-4.84 to 3.69	-8.31 to 7.16	0.28
Donepezil + Memantine vs Placebo	0.43	-6.36 to 7.21	-9.06 to 9.91	0.45
Galantamine + Memantine vs Placebo	0.88	-5.90 to 7.66	-8.61 to 10.37	0.51
Rivastigmine transdermal + Memantine vs Placebo	1.11	-4.20 to 6.42	-7.30 to 9.52	0.55
Placebo (reference)				0.31
Common within-network between-study variance: τ^2 = 9.67, I^2 = 97% (97%, 98%)				
Design-by-treatment interaction model for inconsistency χ^2 (d.f., P-value, τ^2): 3.22 (9, 0.96, 13.28)				
**Mean Difference: Studies with Moderate to Severe cognitive impairment, assessed with MMSE at baseline ***				
Donepezil vs Placebo	1.31	0.66 to 1.96	-0.01 to 2.63	0.78
Rivastigmine oral vs Placebo	-1.00	-1.87 to -0.12	-2.51 to 0.51	0.04
Galantamine vs Placebo	-0.21	-1.64 to 1.21	-2.28 to 1.86	0.28
Memantine vs Placebo	0.69	0.07 to 1.31	-0.61 to 2.00	0.59
Donepezil + Memantine vs Placebo	2.49	1.55 to 3.44	0.92 to 4.07	1.00
Placebo (reference)				0.32
Common within-network between-study variance: τ^2 = 0.18, I^2 = 44% (0%, 75%)				
Design-by-treatment interaction model for inconsistency χ^2 (d.f., P-value, τ^2): 2.60 (1, 0.11, 0.11)				
**Mean Difference: Excluding outlier studies***[§]				
Donepezil vs Placebo	0.95	0.59 to 1.32	-0.64 to 2.54	0.57
Rivastigmine oral vs Placebo	0.65	0.09 to 1.22	-1.00 to 2.30	0.37
Galantamine vs Placebo	0.36	-0.38 to 1.09	-1.36 to 2.07	0.22
Rivastigmine transdermal vs Placebo	1.03	0.15 to 1.91	-0.76 to 2.82	0.59
Memantine vs Placebo	0.67	0.02 to 1.32	-1.01 to 2.35	0.39
Donepezil + Memantine vs Placebo	2.04	1.03 to 3.05	0.18 to 3.90	0.92
Galantamine + Memantine vs Placebo	1.87	0.08 to 3.66	-0.53 to 4.26	0.82
Rivastigmine transdermal + Memantine vs Placebo	1.10	-0.33 to 2.53	-1.03 to 3.23	0.58
Placebo (reference)				0.04
Common within-network between-study variance: τ^2 = 0.59, I^2 = 73% (64%, 79%)				
Design-by-treatment interaction model for inconsistency χ^2 (d.f., P-value, τ^2): 10.60 (13, 0.64, 0.61)				
**Accounting for missing outcome data - Informative Missingness Difference of Means**[¶]				
Donepezil vs Placebo	1.42	0.51 to 2.33	0.51 to 2.33	0.59[‖]
Rivastigmine oral vs Placebo	0.45	-1.09 to 1.99	-1.09 to 1.99	0.30[‖]
Galantamine vs Placebo	0.19	-1.78 to 2.17	-1.78 to 2.17	0.25[‖]
Rivastigmine transdermal vs Placebo	2.37	-0.03 to 4.79	-0.03 to 4.79	0.76[‖]
Memantine vs Placebo	0.60	-1.09 to 2.42	-1.09 to 2.42	0.36[‖]
Donepezil + Memantine vs Placebo	2.55	0.09 to 5.01	0.09 to 5.01	0.80[‖]
Galantamine + Memantine vs Placebo	2.26	-2.03 to 6.56	-2.03 to 6.56	0.68[‖]
Rivastigmine transdermal + Memantine vs Placebo	1.81	-1.66 to 5.28	-1.66 to 5.28	0.61[‖]
Placebo (reference)				0.16[‖]
Common within-network between-study variance: τ^2 = 5.47[‖]				
Design-by-treatment interaction model for inconsistency χ^2 (d.f., P-value, τ^2): 4.45 (11, 0.955, 6.45)				
**Mean Difference: Meta-regression, Trial Mean Age**[**]				
Donepezil vs Placebo	1.53	0.52 to 2.53	-3.17 to 6.27	0.50 [††]
Rivastigmine oral vs Placebo	0.80	-0.84 to 2.44	-4.15 to 5.79	0.37 [††]
Galantamine vs Placebo	0.60	-1.63 to 2.83	-4.57 to 5.72	0.25 [††]
Rivastigmine transdermal vs Placebo	2.53	0.06 to 4.98	-2.72 to 7.80	0.75 [††]
Memantine vs Placebo	0.79	-1.18 to 2.74	-4.33 to 5.85	0.37 [††]
Donepezil + Memantine vs Placebo	2.66	0.09 to 5.19	-2.70 to 7.97	0.87 [††]
Galantamine + Memantine vs Placebo	2.39	-2.02 to 6.84	-4.14 to 8.83	0.75 [††]
Rivastigmine transdermal + Memantine vs Placebo	2.05	-1.53 to 5.59	-3.83 to 7.94	0.75 [††]
Placebo (reference)				0.12 [††]
Regression coefficient	0.03	-0.14 to 0.20		
Common within-network between-study variance: τ^2 = 5.50	3.72 to 8.51			

For peer review only - http://bmjopen.bmj.com/site/about/guidelines.xhtml

Design-by-treatment interaction model for inconsistency χ^2 (d.f., P-value, τ^2): 3.92 (11, 0.972, 8.76)				
**Mean Difference: NMA of studies with IPD adjusted for Age**				
Donepezil vs Placebo	0.72	0.03 to 1.42	-0.66 to 2.10	0.66
Rivastigmine oral vs Placebo	0.84	-0.05 to 1.73	-0.75 to 2.43	0.70
Galantamine vs Placebo	0.46	-0.24 to 1.15	-0.92 to 1.83	0.48
Rivastigmine transdermal vs Placebo	1.05	0.04 to 2.06	-0.68 to 2.78	0.83
Memantine vs Placebo	0.06	-0.72 to 0.84	-1.40 to 1.53	0.21
Placebo (reference)				0.12
Common within-network between-study variance: τ^2 = 0.12, I^2 = 29% (0%, 71%)				
Design-by-treatment interaction model for inconsistency χ^2 (d.f., P-value, τ^2): N/A (one closed loop with a single multi-arm trial)				
**Mean Difference: Meta-regression, Percent of Male Participants**[**]				
Donepezil vs Placebo	1.62	0.58 to 2.65	-3.40 to 6.61	0.62 [††]
Rivastigmine oral vs Placebo	0.73	-0.90 to 2.35	-4.30 to 5.81	0.37 [††]
Galantamine vs Placebo	0.62	-1.65 to 2.89	-4.75 to 5.93	0.25 [††]
Rivastigmine Transdermal vs Placebo	2.51	0.01 to 5.04	-2.78 to 7.94	0.75 [††]
Memantine vs Placebo	0.66	-1.47 to 2.77	-4.54 to 5.88	0.25 [††]
Donepezil + Memantine vs Placebo	2.52	-0.40 to 5.45	-3.09 to 8.17	0.75 [††]
Galantamine + Memantine vs Placebo	2.27	-2.28 to 6.83	-4.37 to 8.90	0.75 [††]
Rivastigmine transdermal + Memantine vs Placebo	1.98	-1.67 to 5.65	-4.02 to 7.99	0.75 [††]
Placebo (reference)				0.12 [††]
Regression coefficient	0.01	-0.05 to 0.06		
Common within-network between-study variance: τ^2 = 5.73	3.83 to 8.84			
Design-by-treatment interaction model for inconsistency χ^2 (d.f., P-value, τ^2): 3.72 (10, 0.959, 8.97)				
**Mean difference: NMA of studies with IPD adjusted for Percent of Male Participants**				
Donepezil vs Placebo	0.76	0.05 to 1.47	-0.67 to 2.19	0.67
Rivastigmine oral vs Placebo	0.85	-0.07 to 1.77	-0.80 to 2.50	0.69
Galantamine vs Placebo	0.45	-0.27 to 1.16	-0.99 to 1.88	0.46
Rivastigmine transdermal vs Placebo	1.05	0.01 to 2.09	-0.74 to 2.84	0.81
Memantine vs Placebo	0.10	-0.68 to 0.89	-1.40 to 1.61	0.23
Placebo (reference)				0.11
Common within-network between-study variance: τ^2 = 0.13, I^2 = 32% (0%, 72%)				
Design-by-treatment interaction model for inconsistency χ^2 (d.f., P-value, τ^2): N/A (one closed loop with a single multi-arm trial)				
**Mean Difference: NMA of studies with IPD adjusted for cognitive impairment, assessed with MMSE at baseline**				
Donepezil vs Placebo	0.79	0.26 to 1.32	-0.06 to 1.64	0.64
Rivastigmine oral vs Placebo	0.88	0.31 to 1.45	-0.05 to 1.81	0.69
Galantamine vs Placebo	0.76	0.34 to 1.18	0.08 to 1.44	0.62
Rivastigmine transdermal vs Placebo	1.02	0.27 to 1.77	-0.20 to 2.24	0.82
Memantine vs Placebo	0.07	-0.52 to 0.66	-0.89 to 1.03	0.14
Placebo (reference)				0.08
Common within-network between-study variance: τ^2 = 0.00, I^2 = 0% (0%, 79%)				
Design-by-treatment interaction model for inconsistency χ^2 (d.f., P-value, τ^2): N/A (one closed loop with a single multi-arm trial)				
**Mean Difference: NMA of studies with IPD adjusted for comorbidities**				
Donepezil vs Placebo	0.77	0.21 to 1.33	-0.15 to 1.68	0.71
Rivastigmine oral vs Placebo	0.88	0.31 to 1.45	-0.05 to 1.81	0.75
Galantamine vs Placebo	-0.29	-1.46 to 0.88	-2.19 to 1.61	0.15
Rivastigmine transdermal vs Placebo	1.05	0.30 to 1.80	-0.17 to 2.27	0.88
Memantine vs Placebo	0.05	-0.55 to 0.64	-0.92 to 1.01	0.27
Placebo (reference)				0.15
Common within-network between-study variance: τ^2 = 0.00, I^2 = 0% (0%, 67%)				
Design-by-treatment interaction model for inconsistency χ^2 (d.f., P-value, τ^2): N/A (one closed loop with a single multi-arm trial)				
**Mean Difference: NMA of studies with IPD adjusted for other medications**				
Donepezil vs Placebo	0.67	-0.34 to 1.69	-1.44 to 2.79	0.61
Rivastigmine oral vs Placebo	0.87	-0.12 to 1.86	-1.21 to 2.95	0.71
Galantamine vs Placebo	0.42	-0.35 to 1.19	-1.40 to 2.25	0.47
Rivastigmine transdermal vs Placebo	1.07	-0.04 to 2.18	-1.16 to 3.30	0.81
Memantine vs Placebo	0.11	-0.74 to 0.96	-1.80 to 2.02	0.26
Placebo (reference)				0.14
Common within-network between-study variance: τ^2 = 0.17, I^2 = 35% (0%, 76%)				
Design-by-treatment interaction model for inconsistency χ^2 (d.f., P-value, τ^2): N/A (one closed loop with a single multi-arm trial)				
**Mean Difference: Meta-regression, Study Duration**[**]				
Donepezil vs Placebo	1.66	0.67 to 2.66	-3.12 to 6.32	0.62 [††]
Rivastigmine oral vs Placebo	0.80	-0.77 to 2.37	-4.14 to 5.69	0.37 [††]
Galantamine vs Placebo	0.47	-1.75 to 2.68	-4.64 to 5.66	0.25 [††]
Rivastigmine transdermal vs Placebo	2.38	-0.04 to 4.83	-2.87 to 7.56	0.75 [††]
Memantine vs Placebo	0.67	-1.27 to 2.58	-4.35 to 5.79	0.25 [††]
Donepezil + Memantine vs Placebo	2.67	0.18 to 5.16	-2.60 to 7.97	0.88 [††]
Galantamine + Memantine vs Placebo	2.43	-1.94 to 6.79	-3.94 to 8.81	0.75 [††]
Rivastigmine transdermal + Memantine vs Placebo	2.13	-1.40 to 5.63	-3.62 to 7.87	0.75 [††]
Placebo (reference)				0.12 [††]

For peer review only - http://bmjopen.bmj.com/site/about/guidelines.xhtml

Regression coefficient	0.02	-0.01 to 0.06		
Common within-network between-study variance: τ^2 = 5.40	3.63 to 8.29			
Design-by-treatment interaction model for inconsistency χ^2 (d.f., P-value, τ^2): 4.36 (13, 0.987, 7.35)				
**Mean Difference: Meta-regression, Year of Publication[**]**				
Donepezil vs Placebo	1.53	0.51 to 2.54	-3.27 to 6.31	0.50[††]
Rivastigmine oral vs Placebo	0.66	-1.01 to 2.32	-4.31 to 5.65	0.25[††]
Galantamine vs Placebo	0.60	-1.65 to 2.85	-4.65 to 5.83	0.25[††]
Rivastigmine transdermal vs Placebo	2.59	0.09 to 5.12	-2.73 to 7.95	0.75[††]
Memantine vs Placebo	0.89	-1.05 to 2.80	-4.17 to 5.90	0.38[††]
Donepezil + Memantine vs Placebo	2.82	0.19 to 5.44	-2.57 to 8.21	0.88[††]
Galantamine + Memantine vs Placebo	2.59	-1.93 to 7.16	-3.98 to 9.12	0.75[††]
Rivastigmine transdermal + Memantine vs Placebo	2.21	-1.49 to 5.95	-3.81 to 8.24	0.75[††]
Placebo (reference)				0.12[††]
Regression coefficient	-0.02	-0.17 to 0.14		
Common within-network between-study variance: τ^2 = 5.53	3.71 to 8.48			
Design-by-treatment interaction model for inconsistency χ^2 (d.f., P-value, τ^2): 4.36 (13, 0.987, 7.35)				
**Adverse Events (AEs)‡**				
**Odds Ratio: Aggregate data and crude results from studies with available individual patient data**				
Donepezil vs Placebo	1.07	0.86 to 1.32	0.68 to 1.67	0.31
Rivastigmine oral vs Placebo	1.26	0.83 to 1.90	0.70 to 2.24	0.16
Galantamine vs Placebo	0.95	0.75 to 1.21	0.60 to 1.51	0.52
Rivastigmine transdermal vs Placebo	0.87	0.57 to 1.35	0.48 to 1.58	0.61
Memantine vs Placebo	0.91	0.67 to 1.22	0.55 to 1.49	0.59
Donepezil + Memantine vs Placebo	0.76	0.34 to 1.68	0.31 to 1.88	0.69
Galantamine + Memantine vs Placebo	1.03	0.45 to 2.36	0.41 to 2.64	0.42
Rivastigmine transdermal + Memantine vs Placebo	0.69	0.32 to 1.51	0.28 to 1.70	0.77
Placebo (reference)				0.43
Common within-network between-study variance τ^2 = 0.04, I^2 = 20% (0%, 47%)				
Design-by-treatment interaction model for inconsistency χ^2 (d.f., P-value, τ^2): 3.58 (6, 0.733, 0.05)				
**Odds Ratio: Aggregate data results[**]**				
Donepezil vs Placebo	1.09	0.89 to 1.33	0.88 to 1.35	0.25
Rivastigmine oral vs Placebo	1.43	0.92 to 2.21	0.90 to 2.26	0.07
Galantamine vs Placebo	0.88	0.63 to 1.25	0.62 to 1.27	0.54
Rivastigmine transdermal vs Placebo	0.79	0.44 to 1.41	0.43 to 1.45	0.61
Memantine vs Placebo	0.70	0.51 to 0.97	0.50 to 0.98	0.77
Donepezil + Memantine vs Placebo	0.77	0.39 to 1.54	0.37 to 1.60	0.64
Galantamine + Memantine vs Placebo	0.96	0.45 to 2.08	0.43 to 2.16	0.44
Rivastigmine transdermal + Memantine vs Placebo	0.62	0.28 to 1.40	0.27 to 1.46	0.80
Placebo (reference)				0.38
Common within-network between-study variance τ^2 = 0.00, I^2 = 0% (0%, 42%)				
Design-by-treatment interaction model for inconsistency χ^2 (d.f., P-value, τ^2): 2.29 (4, 0.682, 0.01)				
**Odds Ratio: Crude results from studies with available individual patient data**				
Donepezil vs Placebo	0.95	0.50 to 1.78	0.33 to 2.70	0.57
Rivastigmine oral vs Placebo	0.81	0.37 to 1.75	0.25 to 2.61	0.71
Galantamine vs Placebo	1.05	0.71 to 1.56	0.44 to 2.50	0.46
Rivastigmine transdermal vs Placebo	0.92	0.38 to 2.20	0.26 to 3.31	0.57
Memantine vs Placebo	1.41	0.81 to 2.45	0.53 to 3.79	0.16
				0.53
Common within-network between-study variance τ^2 = 0.10, I^2 = 48% (0%, 76%)				
Design-by-treatment interaction model for inconsistency χ^2 (d.f., P-value, τ^2): N/A (no closed loops)				
**Odds Ratio: Low Risk of Bias for Allocation Concealment***				
Donepezil vs Placebo	0.88	0.60 to 1.29	0.42 to 1.83	0.52
Rivastigmine oral vs Placebo	1.15	0.67 to 1.98	0.50 to 2.68	0.21
Galantamine vs Placebo	0.94	0.64 to 1.38	0.45 to 1.95	0.44
Rivastigmine transdermal vs Placebo	0.88	0.52 to 1.49	0.39 to 2.02	0.51
Memantine vs Placebo	0.86	0.55 to 1.36	0.40 to 1.88	0.54
Donepezil + Memantine vs Placebo	0.63	0.24 to 1.62	0.19 to 2.05	0.75
Rivastigmine transdermal + Memantine vs Placebo	0.67	0.25 to 1.80	0.20 to 2.28	0.71
Placebo (reference)				0.33
Common within-network between-study variance: τ^2 = 0.08, I^2 = 37% (0%, 64%)				
Design-by-treatment interaction model for inconsistency χ^2 (d.f., P-value, τ^2): 2.19 (3, 0.53, 0.1)				
**Odds Ratio: Low Risk of Bias for Incomplete Data***				
Donepezil vs Placebo	0.83	0.53 to 1.29	0.45 to 1.51	0.51
Galantamine vs Placebo	0.69	0.50 to 0.97	0.42 to 1.13	0.80
Rivastigmine transdermal vs Placebo	0.79	0.42 to 1.49	0.36 to 1.76	0.56
Memantine vs Placebo	0.86	0.60 to 1.22	0.51 to 1.43	0.47
Placebo (reference)				0.16

For peer review only - http://bmjopen.bmj.com/site/about/guidelines.xhtml

Common within-network between-study variance: τ^2 = 0.02, I^2 = 10% (0%, 50%)				
Design-by-treatment interaction model for inconsistency χ^2 (d.f., P-value, τ^2): 0.00 (1, 0.95, 0.04)				
**Odds Ratio: Publicly-Sponsored Studies***				
Donepezil vs Placebo	2.15	0.36 to 12.69	--	0.16
Memantine vs Placebo	0.71	0.45 to 1.12	--	0.86
Donepezil + Memantine vs Placebo	1.53	0.23 to 10.18	--	0.46
Placebo (reference)				0.51
Common within-network between-study variance: τ^2 = N/A (each comparison includes a single study)				
Design-by-treatment interaction model for inconsistency χ^2 (d.f., P-value, τ^2): N/A (no closed loops)				
**Odds Ratio: Industry-Sponsored Studies***				
Donepezil vs Placebo	1.08	0.86 to 1.35	0.64 to 1.82	0.34
Rivastigmine oral vs Placebo	1.27	0.82 to 1.98	0.66 to 2.44	0.16
Galantamine vs Placebo	0.99	0.75 to 1.31	0.57 to 1.71	0.52
Rivastigmine transdermal vs Placebo	0.91	0.57 to 1.44	0.46 to 1.77	0.62
Memantine vs Placebo	0.95	0.65 to 1.37	0.52 to 1.73	0.58
Rivastigmine transdermal + Memantine vs Placebo	0.72	0.31 to 1.64	0.27 to 1.90	0.79
Placebo (reference)				0.50
Common within-network between-study variance: τ^2 = 0.05, I^2 = 25% (0%, 50%)				
Design-by-treatment interaction model for inconsistency χ^2 (d.f., P-value, τ^2): 3.68 (6, 0.72, 0.07)				
**Odds Ratio: Studies with Mild to Moderate cognitive impairment, assessed with MMSE at baseline ***				
Donepezil vs Placebo	1.27	0.88 to 1.83	0.61 to 2.65	0.29
Rivastigmine oral vs Placebo	1.36	0.83 to 2.24	0.60 to 3.09	0.25
Galantamine vs Placebo	1.01	0.67 to 1.55	0.47 to 2.19	0.56
Rivastigmine transdermal vs Placebo	1.02	0.50 to 2.05	0.39 to 2.69	0.55
Memantine vs Placebo	0.86	0.54 to 1.37	0.39 to 1.91	0.73
Galantamine + Memantine vs Placebo	1.10	0.40 to 3.00	0.32 to 3.78	0.48
Rivastigmine transdermal + Memantine vs Placebo	0.96	0.18 to 5.19	0.14 to 6.37	0.55
Placebo (reference)				0.59
Common within-network between-study variance: τ^2 = 0.09, I^2 = 29% (0%, 57%)				
Design-by-treatment interaction model for inconsistency χ^2 (d.f., P-value, τ^2): 3.29 (5, 0.66, 0.13)				
**Odds Ratio: Studies with Moderate to Severe cognitive impairment, assessed with MMSE at baseline ***				
Donepezil vs Placebo	0.92	0.67 to 1.27	0.59 to 1.45	0.38
Galantamine vs Placebo	0.70	0.46 to 1.07	0.38 to 1.28	0.76
Memantine vs Placebo	0.95	0.55 to 1.62	0.44 to 2.02	0.36
Donepezil + Memantine vs Placebo	0.66	0.32 to 1.37	0.23 to 1.86	0.76
Placebo (reference)				0.23
Common within-network between-study variance: τ^2 = 0.00, I^2 = 0% (0%, 72%)				
Design-by-treatment interaction model for inconsistency χ^2 (d.f., P-value, τ^2): 2.90 (1, 0.09, 0.00)				
**Odds Ratio: NMA of studies with IPD – available case analysis**				
Donepezil vs Placebo	1.63	0.49 to 5.41	0.30 to 8.73	0.33
Rivastigmine oral vs Placebo	1.28	0.08 to 19.94	0.04 to 39.11	0.46
Galantamine vs Placebo	1.05	0.67 to 1.63	0.38 to 2.85	0.58
Rivastigmine transdermal vs Placebo	0.81	0.02 to 35.04	0.01 to 82.49	0.59
Memantine vs Placebo	1.35	0.72 to 2.55	0.43 to 4.24	0.38
Placebo (reference)				0.64
Common within-network between-study variance: τ^2 = 0.13, I^2 = 50% (0%, 77%)				
Design-by-treatment interaction model for inconsistency χ^2 (d.f., P-value, heterogeneity): N/A (no closed loops)				
**Odds Ratio: Meta-regression, Trial Mean Age**[**]**				
Donepezil vs Placebo	1.13	0.88 to 1.43	0.68 to 1.86	0.25 [††]
Rivastigmine oral vs Placebo	1.52	0.89 to 2.53	0.77 to 3.04	0.00 [††]
Galantamine vs Placebo	0.91	0.60 to 1.30	0.52 to 1.59	0.50 [††]
Rivastigmine transdermal vs Placebo	0.84	0.39 to 1.58	0.34 to 1.80	0.75 [††]
Memantine vs Placebo	0.74	0.48 to 1.07	0.39 to 1.26	0.75 [††]
Donepezil + Memantine vs Placebo	0.92	0.38 to 1.89	0.33 to 2.15	0.62 [††]
Galantamine + Memantine vs Placebo	0.99	0.37 to 2.27	0.33 to 2.55	0.50 [††]
Rivastigmine transdermal + Memantine vs Placebo	0.73	0.24 to 1.70	0.22 to 1.87	0.87 [††]
Placebo (reference)				0.37 [††]
Regression coefficient (log-scale)	-0.03	-0.08 to 0.02		
Common within-network between-study variance: τ^2 = 0.02	0.00 to 0.19			
Design-by-treatment interaction model for inconsistency χ^2 (d.f., P-value, τ^2): 3.57 (6, 0.735, 0.06)				
**Odds Ratio: NMA of studies with IPD adjusted for Age**				
Donepezil vs Placebo	0.95	0.50 to 1.78	0.33 to 2.73	0.57
Rivastigmine oral vs Placebo	0.84	0.39 to 1.81	0.26 to 2.74	0.68
Galantamine vs Placebo	1.04	0.70 to 1.55	0.43 to 2.52	0.46
Rivastigmine transdermal vs Placebo	0.91	0.38 to 2.17	0.25 to 3.28	0.58
Memantine vs Placebo	1.39	0.80 to 2.44	0.52 to 3.79	0.17
Placebo (reference)				0.53
Common within-network between-study variance: τ^2 = 0.10, I^2 = 48% (0%, 76%)				
Design-by-treatment interaction model for inconsistency χ^2 (d.f., P-value, τ^2): N/A (no closed loops)				

For peer review only - http://bmjopen.bmj.com/site/about/guidelines.xhtml

**Odds Ratio: Meta-regression, Percent of Male Participants[**]**				
Donepezil vs Placebo	1.12	0.87 to 1.44	0.64 to 2.01	0.25 [††]
Rivastigmine oral vs Placebo	1.71	0.97 to 2.92	0.83 to 3.67	0.00 [††]
Galantamine vs Placebo	0.93	0.62 to 1.36	0.49 to 1.77	0.50 [††]
Rivastigmine transdermal vs Placebo	0.89	0.39 to 1.79	0.34 to 2.05	0.63 [††]
Memantine vs Placebo	0.64	0.37 to 1.00	0.29 to 1.21	0.88 [††]
Donepezil + Memantine vs Placebo	0.88	0.35 to 1.88	0.30 to 2.13	0.63 [††]
Galantamine + Memantine vs Placebo	1.13	0.39 to 2.58	0.36 to 2.95	0.38 [††]
Rivastigmine transdermal + Memantine vs Placebo	0.77	0.24 to 1.93	0.21 to 2.13	0.88 [††]
Placebo (reference)				0.38 [††]
Regression coefficient (log-scale)	0.00	0.00 to 0.02		
Common within-network between-study variance: $\tau^2 = 0.03$	0.00 to 0.23			
Design-by-treatment interaction model for inconsistency χ^2 (d.f., P-value, τ^2): 3.57 (6, 0.735, 0.06)				
**Odds Ratio: NMA of studies with IPD adjusted for Percent of Male Participants**				
Donepezil vs Placebo	1.04	0.54 to 1.99	0.34 to 3.16	0.49
Rivastigmine oral vs Placebo	0.81	0.37 to 1.80	0.24 to 2.79	0.72
Galantamine vs Placebo	1.05	0.70 to 1.59	0.42 to 2.65	0.48
Rivastigmine transdermal vs Placebo	0.92	0.37 to 2.27	0.24 to 3.52	0.58
Memantine vs Placebo	1.40	0.80 to 2.48	0.50 to 3.98	0.19
Placebo (reference)				0.55
Common within-network between-study variance: $\tau^2 = 0.11$, $I^2 = 51\%$ (0%, 77%)				
Design-by-treatment interaction model for inconsistency χ^2 (d.f., P-value, τ^2): N/A (no closed loops)				
**Odds Ratio: NMA of studies with IPD adjusted for cognitive impairment, assessed with MMSE at baseline**				
Donepezil vs Placebo	0.97	0.46 to 2.06	0.23 to 4.03	0.56
Rivastigmine oral vs Placebo	0.81	0.33 to 2.01	0.17 to 3.91	0.70
Galantamine vs Placebo	1.29	0.74 to 2.25	0.37 to 4.55	0.28
Rivastigmine transdermal vs Placebo	0.93	0.34 to 2.53	0.18 to 4.91	0.57
Memantine vs Placebo	1.26	0.59 to 2.70	0.30 to 5.28	0.33
Placebo (reference)				0.56
Common within-network between-study variance: $\tau^2 = 0.16$, $I^2 = 52\%$ (0%, 80%)				
Design-by-treatment interaction model for inconsistency χ^2 (d.f., P-value, τ^2): N/A (no closed loops)				
**Odds Ratio: NMA of studies with IPD adjusted for comorbidities**				
Donepezil vs Placebo	1.01	0.52 to 1.96	0.29 to 3.50	0.51
Rivastigmine oral vs Placebo	0.82	0.36 to 1.87	0.20 to 3.32	0.69
Galantamine vs Placebo	1.02	0.57 to 1.80	0.32 to 3.26	0.50
Rivastigmine transdermal vs Placebo	0.91	0.36 to 2.31	0.20 to 4.11	0.58
Memantine vs Placebo	1.42	0.79 to 2.55	0.44 to 4.59	0.18
Placebo (reference)				0.53
Common within-network between-study variance: $\tau^2 = 0.12$, $I^2 = 44\%$ (0%, 77%)				
Design-by-treatment interaction model for inconsistency χ^2 (d.f., P-value, τ^2): N/A (no closed loops)				
**Odds Ratio: NMA of studies with IPD adjusted for other medications**				
Donepezil vs Placebo	1.17	0.49 to 3.03	0.28 to 4.88	0.41
Rivastigmine oral vs Placebo	0.82	0.37 to 1.81	0.23 to 2.91	0.72
Galantamine vs Placebo	1.03	0.69 to 1.55	0.40 to 2.65	0.51
Rivastigmine transdermal vs Placebo	0.95	0.39 to 2.34	0.24 to 2.91	0.56
Memantine vs Placebo	1.34	0.75 to 2.39	0.46 to 3.92	0.25
Placebo (reference)				0.56
Common within-network between-study variance: $\tau^2 = 0.11$, $I^2 = 51\%$ (0%, 78%)				
Design-by-treatment interaction model for inconsistency χ^2 (d.f., P-value, τ^2): N/A (no closed loops)				
**Odds Ratio: Meta-regression, Study Duration[**]**				
Donepezil vs Placebo	1.12	0.87 to 1.43	0.63 to 1.95	0.25 [††]
Rivastigmine oral vs Placebo	1.76	1.00 to 2.99	0.88 to 3.68	0.00 [††]
Galantamine vs Placebo	0.92	0.62 to 1.36	0.50 to 1.69	0.50 [††]
Rivastigmine transdermal vs Placebo	0.87	0.39 to 1.70	0.34 to 1.96	0.63 [††]
Memantine vs Placebo	0.61	0.37 to 0.93	0.31 to 1.13	0.88 [††]
Donepezil + Memantine vs Placebo	0.76	0.29 to 1.69	0.26 to 1.90	0.75 [††]
Galantamine + Memantine vs Placebo	0.98	0.34 to 2.26	0.30 to 2.53	0.50 [††]
Rivastigmine transdermal + Memantine vs Placebo	0.75	0.25 to 1.81	0.23 to 1.97	0.75 [††]
Placebo (reference)				0.38 [††]
Regression coefficient (log-scale)	0.00	0.00 to 0.01		
Common within-network between-study variance: $\tau^2 = 0.03$	0.00 to 0.22			
Design-by-treatment interaction model for inconsistency χ^2 (d.f., P-value, τ^2): 3.57 (6, 0.735, 0.06)				
**Odds Ratio: Meta-regression, Year of Publication[**]**				
Donepezil vs Placebo	1.05	0.79 to 1.38	0.61 to 1.77	0.38[††]
Rivastigmine oral vs Placebo	1.68	0.98 to 2.77	0.85 to 3.37	0.00 [††]
Galantamine vs Placebo	0.91	0.61 to 1.32	0.50 to 1.64	0.63 [††]
Rivastigmine transdermal vs Placebo	0.92	0.40 to 1.84	0.36 to 2.04	0.63 [††]
Memantine vs Placebo	0.73	0.46 to 1.05	0.38 to 1.28	0.88 [††]
Donepezil + Memantine vs Placebo	0.88	0.35 to 1.83	0.31 to 2.15	0.75 [††]

For peer review only - http://bmjopen.bmj.com/site/about/guidelines.xhtml

Galantamine + Memantine vs Placebo	1.24	0.43 to 2.85	0.39 to 3.25	0.25 ††
Rivastigmine transdermal + Memantine vs Placebo	0.88	0.24 to 2.24	0.24 to 2.42	0.75 ††
Placebo (reference)				0.38 ††
Regression coefficient (log-scale)	-0.02	-0.06 to 0.03		
Common within-network between-study variance: $\tau^2 = 0.02$	0.00 to 0.21			
Design-by-treatment interaction model for inconsistency χ^2 *(d.f., P-value,* τ^2*)*: 3.57 (6, 0.735, 0.06)				

* Aggregate data and fully adjusted results from studies with available individual patient data
† MMSE: Studies with available IPD included only available participants – to assess the missing data impact on the second stage a separate analysis was applied (IMDoM)
‡ AE: Studies with available IPD included all randomized participants
§ Outlier studies:

- Hernandez C, Unturbe F, Martinez-Lage P, Lucas A, Gregorio P, Alonso T. Effects of combined pharmacologic and cognitive treatment in the progression of moderate dementia: a two-year follow-up. REVISTA ESPANOLA DE GERIATRIA Y GERONTOLOGIA. 2007;42(1):3
- Moretti DV. Alpha rhythm oscillations and MMSE scores are differently modified by transdermal or oral rivastigmine in patients with Alzheimer's disease. American journal of neurodegenerative disease. 2014;3(2):72-83.

¶ Included studies with available raw data only, irrespective having access to individual patient data
|| Analyses were conducted in Stata using the *metamiss2* and *network* commands; I2 is not available; SUCRA values are presented instead of P-scores
** Studies with aggregate data were used (studies with available individual patient data were not included in this analysis)
†† Analyses were conducted in OpenBUGS, and SUCRA values were calculated instead of P-scores

For peer review only

For peer review only - http://bmjopen.bmj.com/site/about/guidelines.xhtml

Appendix 17: CINeMA results

Risk of bias contributions: The bar chart shows the contributions of each piece of study to the network estimate

Interventions
A: PLAC
B: DONE
C: RIVA_O
D: GALA
E: RIVA_P
F: MEMA
G: DONE+MEMA
H: GALA+MEMA
I: RIVA_P+MEMA

MMSE outcome

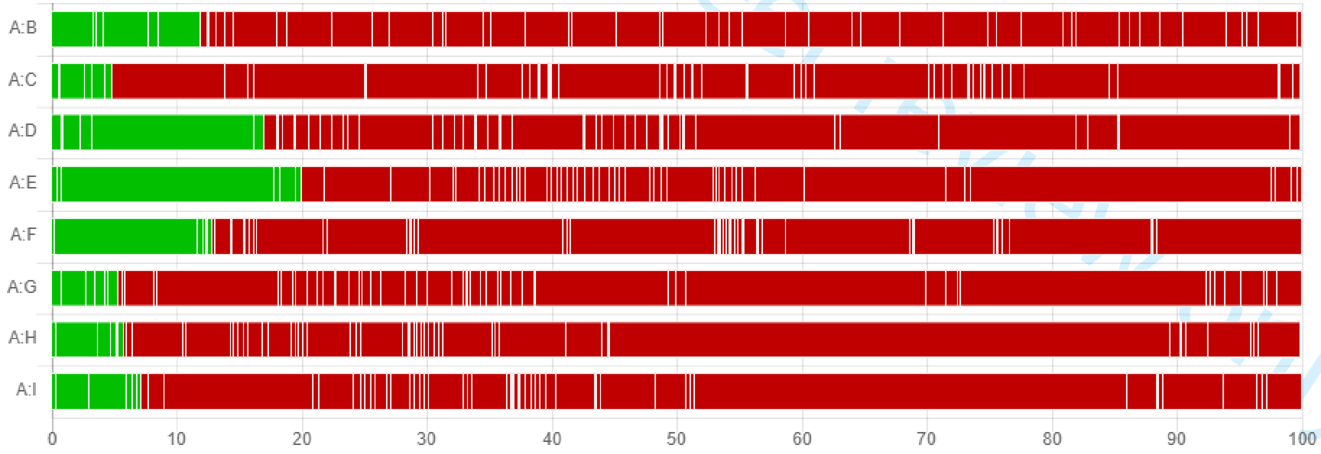

AE outcome

For peer review only - http://bmjopen.bmj.com/site/about/guidelines.xhtml

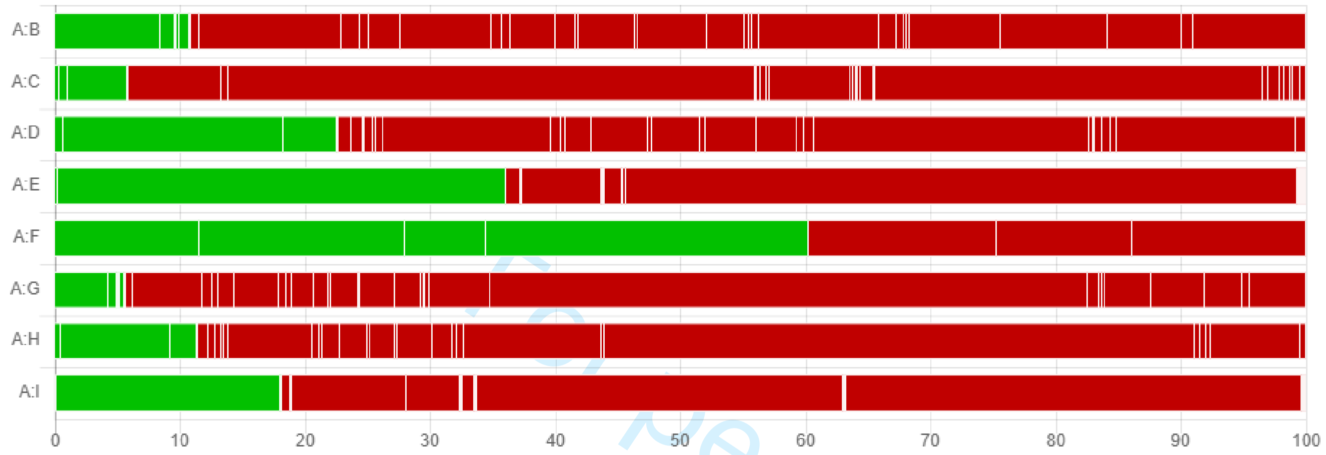

CINeMA report

MMSE outcome

Comparison	# of studies	Nature of evidence	Type of data	Within-study bias (D1)	Reporting bias (D2)	Indirectness (D3)	Imprecision (D4)	Heterogeneity (D5)	Incoherence (D6)	Confidence rating	Downgrading due to
DONE vs PLAC	24	Mixed	IPD+AD	Major concerns	Suspected	No concerns	No concerns	Major concerns	No concerns	Moderate	D5
RIVA_O vs PLAC	6	Mixed	IPD+AD	Major concerns	Suspected	No concerns	Some concerns	Some concerns	No concerns	Moderate	D4;D5
GALA vs PLAC	3	Mixed	IPD+AD	Major concerns	Suspected	No concerns	Major concerns	No concerns	No concerns	Moderate	D4
RIVA_P vs PLAC	2	Mixed	IPD+AD	Major concerns	Suspected	No concerns	Some concerns	Some concerns	No concerns	Moderate	D4;D5
MEMA vs PLAC	7	Mixed	IPD+AD	Major concerns	Suspected	No concerns	Some concerns	Some concerns	No concerns	Moderate	D4;D5
DONE+MEMA vs PLAC	1	Mixed	AD	Major concerns	Suspected	No concerns	No concerns	Major concerns	No concerns	Moderate	D5
GALA+MEMA vs PLAC	0	Indirect	-	Major concerns	Suspected	No concerns	Major concerns	No concerns	No concerns	Moderate	D4
RIVA_P+MEMA vs PLAC	0	Indirect	-	Major concerns	Suspected	No concerns	Major concerns	No concerns	No concerns	Moderate	D4

AE outcome

Comparison	# of studies	Nature of evidence	Type of data	Within-study bias (D1)	Reporting bias (D2)	Indirectness (D3)	Imprecision (D4)	Heterogeneity (D5)	Incoherence (D6)	Confidence rating	Downgrading due to
DONE vs PLAC	16	Mixed	IPD+AD	Major concerns	Undetected	No concerns	Major concerns	No concerns	No concerns	Moderate	D1
RIVA_O vs PLAC	3	Mixed	IPD+AD	Major concerns	Undetected	No concerns	Major concerns	No concerns	No concerns	Moderate	D1
GALA vs PLAC	8	Mixed	IPD+AD	Major concerns	Undetected	No concerns	Major concerns	No concerns	No concerns	Moderate	D1
RIVA_P vs PLAC	2	Mixed	IPD+AD	Some concerns	Undetected	No concerns	Major concerns	No concerns	No concerns	High	
MEMA vs PLAC	7	Mixed	IPD+AD	Some concerns	Undetected	No concerns	Major concerns	No concerns	No concerns	High	
DONE+MEMA vs PLAC	2	Mixed	AD	Major concerns	Undetected	No concerns	Major concerns	No concerns	No concerns	Moderate	D1
GALA+MEMA vs PLAC	0	Indirect	-	Major concerns	Undetected	No concerns	Major concerns	No concerns	No concerns	Moderate	D1
RIVA_P+MEMA vs PLAC	0	Indirect	-	Major concerns	Undetected	No concerns	Major concerns	No concerns	No concerns	Moderate	D1

Abbreviations: DONE, donepezil; GALA, galantamine; MEMA, memantine; PLAC, placebo; RIVA_O, rivastigmine oral; RIVA_P, rivastigmine patch

For peer review only - http://bmjopen.bmj.com/site/about/guidelines.xhtml

Appendix 18: Study definitions for adverse events

Author, Year	Source of Definition	Definition
Agid, 1998	Determined by Investigator	"Patients and caregivers were questioned systematically regarding the occurrence of adverse events at each clinical visit"
Ancoli-Israel, 2005	Determined by Investigator	"Only one serious AE leading to discontinuation, hepatic failure, in the donepezil-treated group was considered to be possibly due to study treatment by the investigator."
Andersen, 2012	NA	NA
Araki, 2014	NA	NA
Bakchine, 2008	Determined by Investigator	" A patient could also be withdrawn from the study if: they had a serious adverse event (SAE: death, life-threatening condition, hospitalisation) [..] Three patients had an SAE that was considered by the investigator to be possibly or probably related to treatment.”
Black, 2007	Determined by Investigator	"AEs were considered serious (SAEs) when death occurred, life was threatened, hospitalization or prolonged hospitalization was required, or a significant disability occurred."
Blesa González, 2011	NA	NA
Burns,1999	COSTART	"Serious adverse events (SAE) included fatal or life-threatening situations, permanently disabling conditions or incidents that required or prolonged hospitalisation […] Events were coded using a modified COSTART dictionary, and the assessment of relationship to treatment for all adverse events was conducted blind to treatment assignment."
Burns, 2009	NR	NR
Burns, 2011	NR	NR
Choi, 2011	Determined by Investigator	"Investigators were asked to evaluate severity (mild, moderate, or severe), relationship to study drug (not related, probable relationship with rivastigmine patch, probable relationship with memantine, or probable relationship with an interaction of the two drugs), and seriousness of the AEs."
Corey-Bloom, 1998	NA	NA
Cretu, 2008	NA	NA
Dysken, 2014	Medical Dictionary for Regulatory Activities	"Serious AEs were coded according to the Medical Dictionary for Regulatory Activities."
Farlow, 2013	NA	NA
Feldman, 2001	Determined by Investigator	"Serious AE was defined as any AE that was life threatening or resulted in death, hospitalization, prolongation of hospitalization, or significant disability."
Feldman, 2007	World Health Organisation preferred terms	" A similar proportion of patients in each treatment group experienced at least one serious adverse event (any event that was fatal, considered life threatening or required hospitalisation) […] All adverse events were recorded using the Novartis Medical Terminology Thesaurus (a modified version of the WHO adverse reaction terminology dictionary)."
Fox, 2012	NA	NA
Frolich, 2011	NA	NA
Fuschillo, 2001	NA	NA
Gault L, 2015	Medical Dictionary for Regulatory Activities	"AEs were coded using the Medical Dictionary for Regulatory Activities"
Gold, 2010	NR	"SAE (fatal or nonfatal) "
Greenberg, 2000	Determined by Investigator	"Of 9 withdrawals from the study after randomization, 2 were due to serious adverse events judged to be possibly related to donepezil therapy: syncope and generalized seizure (1 patient each). "
Grossberg, 2013	Medical Dictionary for Regulatory Activities	"Adverse events were coded according to the Medical Dictionary for Regulatory Activities (version 7.0 or newer), and an assessment of the severity, chronicity, causal relationship to study medication, and seriousness of the event was provided by an investigator"
Hager, 2014	Determined by Investigator	"Safety data were monitored during the study by a company-commissioned, external, independent, blinded Data Safety Monitoring Board (DSMB). Secondary safety outcomes were the number of treatment emergent adverse events (TEAEs), including serious TEAEs."
Haig, 2014	Determined by Investigator	"The incidence of adverse events considered possibly or probably related to study drug as assessed by the investigator was generally similar across treatment groups (range 20.6% to 26.8%).” “Treatment emergent adverse events were tabulated by primary Medical Dictionary for Regulatory Activities (MedDRA) [23] version 13.1 System Organ Class and Preferred Term"
Hernández, 2007	NA	NA
Herrmann, 2013	Determined by Investigator	"The incidence of adverse events considered related to the study drug by the investigator was 30% in the placebo group and 36% in the memantine group"
Holmes, 2004	Determined by Investigator	"During these (clinic) visits, psychometric evaluations, medication compliance checks, and adverse event (AE) monitoring took place"

For peer review only - http://bmjopen.bmj.com/site/about/guidelines.xhtml

Homma, 1998	NR	NR
Homma, 2008	Medical Dictionary for Regulatory Activities – Japanese Version	"AE terms were standardized according to the Medical Dictionary for Regulatory Activities – Japanese Version . AEs were graded on a 3-point scale (mild: discomfort noticed, but no disruption of normal daily activity; moderate: discomfort sufficient to reduce or affect normal daily activity; severe: incapacitating, with inability to work or to perform normal daily activity). "
Hong, 2006	NR	NR
Howard, 2007	NA	NA
Howard, 2012	NR	NR
Hu, 2006	NA	NA
Johannsen, 2006	NA	NA
Jones, 2004	Determined by Investigator	"A serious adverse event (SAE) was defined as any AE that was life threatening or resulted in death, hospitalisation, prolongation of hospitalisation, or significant disability"
Kadir, 2008	NA	NA
Kano, 2013	NA	NA
Karaman, 2005	NA	NA
Likitjaroen, 2012	NA	NA
Lorenzi, 2011	NA	NA
Maher-Edwards, 2011	Determined by Investigator	"Eight subjects experienced nonfatal serious AEs; all were considered unrelated to the study drug"
Marek, 2014	Medical Dictionary for Regulatory Activities	"Aes were coded using the Medical Dictionary for Regulatory Activities (MedDRA, version 14.0) by system organ class and preferred term"
Mazza, 2006	NA	NA
Mohs, 2001	Determined by Investigator	"In all cases, judgment of the relationship of study treatment to an adverse event and of the severity of the event was made by the investigator under double-blind conditions. "
Moretti, 2014	NA	NA
Mowla, 2007	NA	NA
Nakamura, 2011	Determined by Investigator	"Safety evaluations included recording all adverse events on Adverse Event Case Report Forms. Every serious adverse event occurring after the patient provided informed consent and until 28 days after the patient stopped the study was reported. "
Nakano, 2001	NA	NA
Nordberg, 2009	Determined by Investigator	"Safety and tolerability were monitored throughout the study by recording all adverse events (AEs). "
Pakdaman H, 2015	NA	NA
Peng, 2005	NA	NA
Peskind, 2006	Determined by Investigator	"Overall, the type and incidence of SAEs were similar between the memantine and placebo groups. One participant death occurred in each group during the trial; neither was rated by the investigator as being treatment-related"
Peters O, 2015	NR	NR
Reisberg, 2003	NR	NR
Rockwood, 2001	World Health Organisation preferred terms	"adverse events (classified according to World Health Organisation preferred terms)."
Rockwood, 2006	NR	NR
Rogers, 1996		
Rogers, 1998	COSTART	"Events, recorded using investigator terminology, were grouped and coded into common terms using a modified COSTART dictionary"
Rogers, 1998	COSTART	"Events, recorded using investigator terminology, were grouped and coded into common terms using a modified COSTART dictionary. "
Saxton, 2012	Determined by Investigator	"Treatment-emergent adverse events (TEAEs) and serious adverse events (SAEs) were recorded at all post-Screening study visits"
Scarpini, 2011	Determined by Investigator	"Subjects with a treatment 51 (20.1) 2 (2.6) 4 (6.3) related AE, as judged by the investigator"
Schmidt, 2008	NA	NA
Seltzer, 2004	NA	NA
Shao, 2015	NA	NA
Shimizu, 2015	NA	NA
Sole-Padulles, 2013	NA	NA
Tariot, 2000	World Health Organisation preferred terms	"adverse events (classified according to World Health Organization Preferred Term). "
Tariot, 2001	COSTART	"Investigator terms describing AEs were coded to standard preferred terms using a modified Coding Symbols for Thesaurus of Adverse Reaction Terms dictionary. "
Thomas, 2001		

For peer review only - http://bmjopen.bmj.com/site/about/guidelines.xhtml

Wilcock, 2003	World Health Organisation preferred terms	"monitoring for adverse events (classified according to WHO preferred terms) "
Wilkinson, 2001	Determined by Investigator	"All adverse events were recorded, regardless of the considered relationship to treatment. All details of adverse events and their outcomes were recorded including severity and relationship to treatment. Serious adverse events were documented separately. "
Wilkinson, 2002	NR	NR
Wilkinson, 2012	Determined by Investigator	"Tolerability and safety were based on the incidence of adverse events, either reported spontaneously by the patients or in response to a non-leading question by the investigator throughout the study"
Winblad, 2001	NR	NR
Winblad, 2006	COSTART	"We recorded all treatment emergent adverse events, coding them according to a modified COSTART dictionary. "
Winblad, 2007	Determined by Investigator	"Safety evaluations included recording all adverse events, which were coded using a standard glossary."
Zhang-Yi, 2005	NA	NA
Zhang, 2012	Determined by Investigator	"Serious adverse events considered to be possibly related to treatment occurred in one patient in each treatment arm"

Notes: [a]Unpublished data, [b]Non-English studies
Abbreviations: CR, companion report; NA, not applicable; NR, not reported.

For peer review only

Appendix 19: Time taken to achieve at least an adverse event using individual patient data

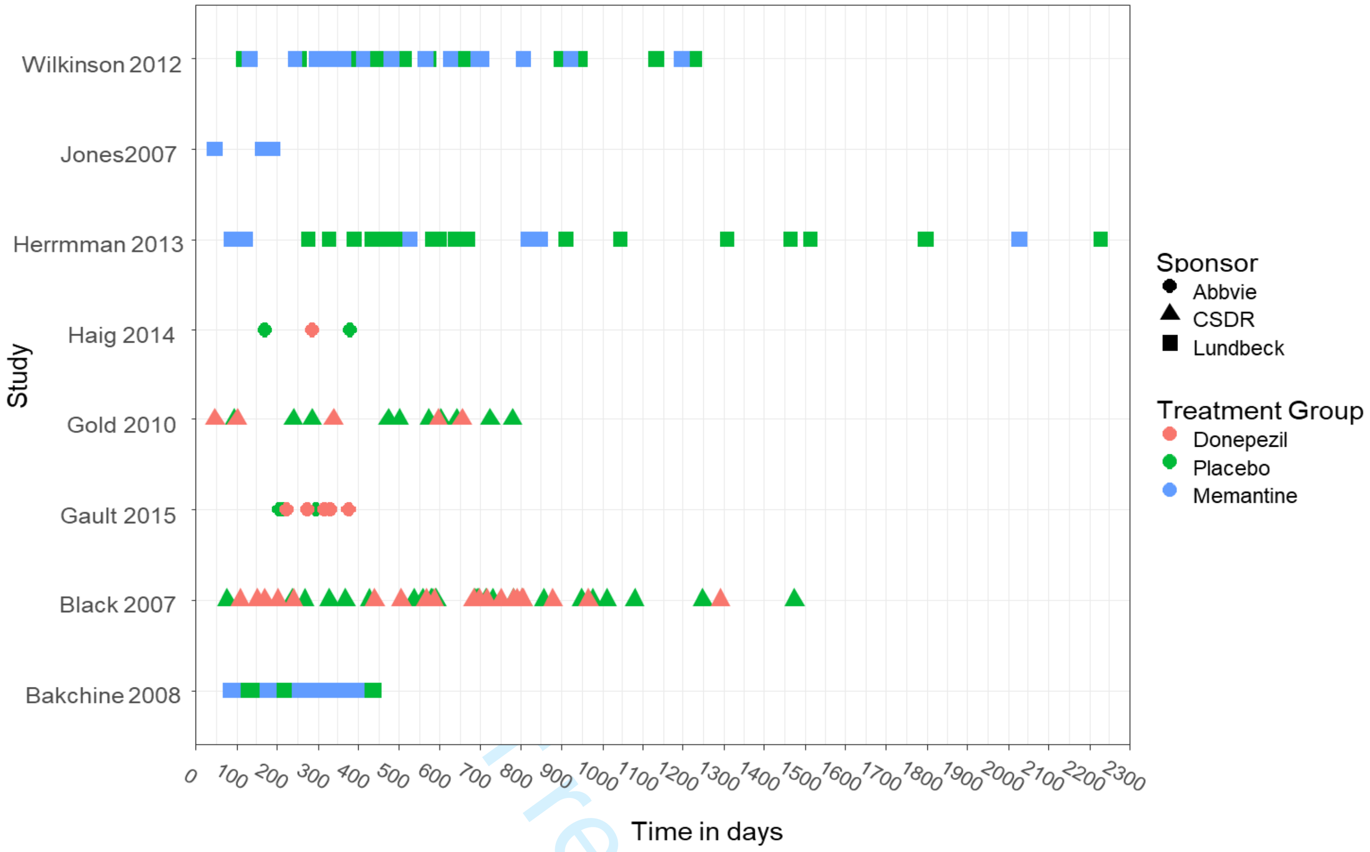

Appendix 20: Rank-heat plot for adverse events

Circles from inside out present results for different network meta-analyses including: i) aggregate data (AD) only (studies with available IPD are not included in the analysis), ii) crude results from individual studies with individual patient data (IPD), iii) AD and crude results from studies with available IPD, and iv) AD and fully adjusted results from studies with available IPD. Numbers within each sector correspond to the P-score values as calculated in each model.

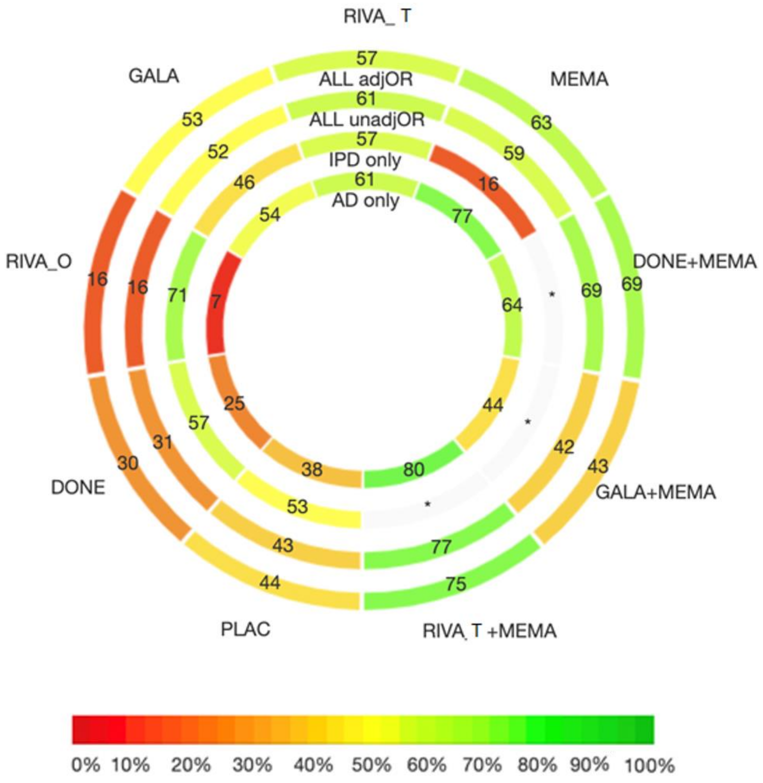

For peer review only - http://bmjopen.bmj.com/site/about/guidelines.xhtml

Appendix 21: Challenges encountered during the individual patient data request from sponsors

- The identification of the trial data set when certain details were not available (e.g. NCT number; particularly for studies published before 2005 that this was established).
- Data ownership.
- Sponsors switched platforms, while we were navigating the data.
- IPD available through proprietary sponsor-specific platforms did not allow for combination of IPD from different sponsor platforms; hence a one-stage analysis as planned in our protocol, was impossible.
- Software availability: Required R packages (e.g., mice) were not available/provided, and we were not allowed to install any new R packages; some R packages were older versions (e.g. lme4).
- Time that the platform permitted access to the IPD was often limited. This is a significant constraint given that IPD from different studies became available at different time points.
- Cost associated with obtaining access to the data for a certain amount of time. Additionally, cost associated with the WHO Drug Dictionary license to obtain access to the additional medications used for each patient; this license's approximate cost was $8,958·25 USD per sponsor.
- Available IPD did not include the full information as shown in the publication: For example, only data for placebo were available, or did not give information about a reported outcome (e.g. only baseline MMSE values were available). Also, date of follow-up was coded in some studies and it was impossible to make a judgement on first and last date.

For peer review only - http://bmjopen.bmj.com/site/about/guidelines.xhtml

Additional File 2: MEDLINE Search Strategy

MEDLINE Search

Database: Ovid MEDLINE(R) In-Process & Other Non-Indexed Citations and Ovid MEDLINE(R) <1946 to Present>, Embase<1980 to 2014 Week 50> Search Strategy:

----

1 alzheimer$.mp.
2 "benign senescent forgetfulness".mp.
3 (cognit$ adj2 (impair$ or declin$ or deficit$ or degenerat$ or deteriorat$ or los$ or disorder$ or complain$ or disturb$)).mp.
4 (cerebr$ adj2 (impair$ or declin$ or deficit$ or degenerat$ or deteriorat$ or los$ or disorder$ or complain$ or disturb$)).mp.
5 (mental adj2 (impair$ or declin$ or deficit$ or degenerat$ or deteriorat$ or los$ or disorder$ or complain$ or disturb$)).mp.
6 (ne?rocognit$ adj2 (impair$ or declin$ or deficit$ or degenerat$ or deteriorat$ or los$ or disorder$ or complain$ or disturb$)).mp.)
7 (ne?ro-cognit$ adj2 (impair$ or declin$ or deficit$ or degenerat$ or deteriorat$ or los$ or disorder$ or complain$ or disturb$)).mp.
8 ((cognit$ or memory or cerebral or brain) adj2 (improv$ or enhanc$ or perform$ or process$ or function$ or rehabilitation or aid$ or stimulat$)).mp.
9 cognition.ti.
10 (confusion$ or confused).tw.
11 dement$.mp.
12 ("normal pressure hydrocephalus" and shunt$).mp.
13 "organic brain disease$".mp.
14 "organic brain syndrome".mp.
15 (presenil$ or pre-senil$ or senil$).tw.
16 Alzheimer Disease/
17 Cognition/de
18 Confusion/
19 Dementia/
20 or/1-19
21 abixa.tw.
22 aricept.tw.
23 (acetylcholinesteraseadj inhibitor$).tw.
24 axura.tw.
25 akatinol.tw.
26 (anticholinesterase? or anti-cholinesterase?).tw.
27 (cognitive adjenhanc$).mp.
28 (cholinesterase adj inhibitor$).mp.
29 ChEI.tw.
30 donepezil.mp.
31 ebixa.tw.
32 eranz.tw.
33 exelon.tw.
34 galant?amin$.tw.
35 lycoremine.tw.

For peer review only - http://bmjopen.bmj.com/site/about/guidelines.xhtml

36 memantin$.tw.
37 memox.tw.
38 namenda.tw.
39 nimvastid.tw.
40 nivalin$.tw.
41 "N-Methyl-D-aspartic acid receptor antagonist$".tw.
42 prometax.tw.
43 razadyne.tw.
44 reminyl.tw.
45 rivastigmine.mp.
46 exp Cholinesterase Inhibitors/
47 Galantamine/
48 Memantine/
49 Galantamin.rn.
50 Memantine.rn.
51 Donepezil.rn.
52 Donepezil Hydrochloride.rn.
53 Rivastigmine.rn.
54 or/21-53
55 20 and 54
56 exp Animals/ not (exp Animals/ and Humans/)
57 55 and 56
58 (comment or editorial or interview or news).pt.
59 (letter not (letter and randomized controlled trial)).pt.
60 57 not (58 or 59)
61 (201111* or 201112* or 2012* or 2013* or 2014*).ed.
62 60 and 61
63 alzheimer$.mp.
64 "benign senescent forgetfulness".mp.
65 (cognit$ adj2 (impair$ or declin$ or deficit$ or degenerat$ or deteriorat$ or los$ or disorder$ or complain$ or disturb$)).mp.
66 (cerebr$ adj2 (impair$ or declin$ or deficit$ or degenerat$ or deteriorat$ or los$ or disorder$ or complain$ or disturb$)).mp.
67 (mental adj2 (impair$ or declin$ or deficit$ or degenerat$ or deteriorat$ or los$ or disorder$ or complain$ or disturb$)).mp.
68 (ne?rocognit$ adj2 (impair$ or declin$ or deficit$ or degenerat$ or deteriorat$ or los$ or disorder$ or complain$ or disturb$)).mp.
69 (ne?ro-cognit$ adj2 (impair$ or declin$ or deficit$ or degenerat$ or deteriorat$ or los$ or disorder$ or complain$ or disturb$)).mp.
70 ((cognit$ or memory or cerebral or brain) adj2 (improv$ or enhanc$ or perform$ or process$ or function$ or rehabilitation or aid$ or stimulat$)).mp.
71 cognition.ti.
72 (confusion$ or confused).tw.
73 dement$.mp.
74 ("normal pressure hydrocephalus" and shunt$).mp.
75 "organic brain disease$".mp.
76 "organic brain syndrome".mp.

For peer review only - http://bmjopen.bmj.com/site/about/guidelines.xhtml

77 (presenil$ or pre-senil$ or senil$).tw
78 Alzheimer disease/
79 cognitive defect/
80 confusion/
81 dementia/
82 organic brain syndrome/
83 or/63-82
84 abixa.tw.
85 aricept.tw.
86 (acetylcholinesteraseadj inhibitor$).tw.
87 axura.tw.
88 akatinol.tw.
89 (anticholinesterase? or anti-cholinesterase?).tw.
90 (cognitive adjenhanc$).mp.
91 (cholinesterase adj inhibitor$).mp.
92 ChEI.tw.
93 donepezil.mp.
94 ebixa.tw.
95 eranz.tw.
96 exelon.tw.
97 galant?amin$.tw.
98 lycoremine.tw.
99 memantin$.tw.
100 memox.tw.
101 namenda.tw.
102 nimvastid.tw.
103 nivalin$.tw.
104 "N-Methyl-D-aspartic acid receptor antagonist$".tw.
105 prometax.tw.
106 razadyne.tw.
107 reminyl.tw.
108 rivastigmine.mp.
109 exp cholinesterase inhibitor/
110 donepezil/ or donepezil plus memantine/
111 galantamine/
112 memantine/
113 rivastigmine/
114 357-70-0.rn.
115 19982-08-2.rn.
116 120011-70-3.rn.
117 120014-06-4.rn.
118 rivastigmine.rn.
119 or/84-118
120 83 and 119
121 randomized controlled trial/ or controlled clinical trial/
122 exp "clinical trial (topic)"/
123 (randomi#ed or randomly or RCT$1 or placebo*).tw.

For peer review only - http://bmjopen.bmj.com/site/about/guidelines.xhtml

124 ((singl* or doubl* or trebl* or tripl*) adj (mask* or blind* or dumm*)).tw.
125 trial.ti.
126 or/121-125
127 120 and 126
128 exp controlled clinical trial/
129 exp "controlled clinical trial (topic)"/
130 (control* adj2 trial*).tw.
131 (nonrandom* or non-random* or quasi-random* or quasi-experiment*).tw.
132 (nRCT or nRCTs or non-RCT$1).tw.
133 (control* adj3 ("before and after" or "before after")).tw.
134 time series analysis/
135 (time series adj3 interrupt*).tw.
136 pretest posttest control group design/
137 (pre- adj3 post-).tw.
138 (pretest adj3 posttest).tw.
139 controlled study/
140 (control* adj2 stud$3).tw.
141 control group/
142 (control$ adj2 group$1).tw.
143 or/128-142
144 120 and 143
145 cohort analysis/
146 cohort.tw.
147 retrospective study/
148 longitudinal study/
149 prospective study/
150 (longitudinal or prospective or retrospective).tw.
151 follow up/
152 ((followup or follow-up) adj (study or studies)).tw.
153 observational study/
154 (observation$2 adj (study or studies)).tw.
155 population research/
156 ((population or population-based) adj (study or studies or analys#s)).tw.
157 ((multidimensional or multi-dimensional) adj (study or studies)).tw.
158 exp comparative study/
159 ((comparative or comparison) adj (study or studies)).tw.
160 exp case control study/
161 ((case-control* or case-based or case-comparison) adj (study or studies)).tw.
162 or/145-161
163 120 and 162
164 127 or 144 or 163
165 exp animal experimentation/ or exp models animal/ or exp animal experiment/ or nonhuman/ or exp vertebrate/
166 exp humans/ or exp human experimentation/ or exp human experiment/
167 165 not 166
168 164 not 167
169 editorial.pt.

For peer review only - http://bmjopen.bmj.com/site/about/guidelines.xhtml

For peer review only

170 letter.pt.not (letter.pt. and randomized controlled trial/)
171 168 not (169 or 170)
172 (2011112* or 2011113* or 201112* or 2012* or 2013* or 2014*).dd.
173 171 and 172
174 62 use prmz
175 173 use emez
176 174 or 175
177 remove duplicates from 176
178 177 use prmz [MEDLINE UNIQUE HITS]
179 177 use emez [EMBASE UNIQUE HITS]
****

For peer review only

For peer review only - http://bmjopen.bmj.com/site/about/guidelines.xhtml

PRISMA 2020 for Abstracts Checklist

Section and Topic	Item #	Checklist item	Reported (Yes/No)
**TITLE**			
Title	1	Identify the report as a systematic review.	Yes
**BACKGROUND**			
Objectives	2	Provide an explicit statement of the main objective(s) or question(s) the review addresses.	Yes
**METHODS**			
Eligibility criteria	3	Specify the inclusion and exclusion criteria for the review.	Yes
Information sources	4	Specify the information sources (e.g. databases, registers) used to identify studies and the date when each was last searched.	Yes
Risk of bias	5	Specify the methods used to assess risk of bias in the included studies.	Yes
Synthesis of results	6	Specify the methods used to present and synthesise results.	Yes
**RESULTS**			
Included studies	7	Give the total number of included studies and participants and summarise relevant characteristics of studies.	Yes
Synthesis of results	8	Present results for main outcomes, preferably indicating the number of included studies and participants for each. If meta-analysis was done, report the summary estimate and confidence/credible interval. If comparing groups, indicate the direction of the effect (i.e. which group is favoured).	Yes
**DISCUSSION**			
Limitations of evidence	9	Provide a brief summary of the limitations of the evidence included in the review (e.g. study risk of bias, inconsistency and imprecision).	Yes
Interpretation	10	Provide a general interpretation of the results and important implications.	Yes
**OTHER**			
Funding	11	Specify the primary source of funding for the review.	Yes
Registration	12	Provide the register name and registration number.	Yes

From: Page MJ, McKenzie JE, Bossuyt PM, Boutron I, Hoffmann TC, Mulrow CD, et al. The PRISMA 2020 statement: an updated guideline for reporting systematic reviews. BMJ 2021;372:n71. doi: 10.1136/bmj.n71

For more information, visit: http://www.prisma-statement.org/

For peer review only

PRISMA NMA Checklist of Items to Include When Reporting A Systematic Review Involving a Network Meta-analysis

Section/Topic	Item #	Checklist Item	Reported on Page #
**TITLE**			
Title	1	Identify the report as a systematic review *incorporating a network meta-analysis (or related form of meta-analysis).*	1
**ABSTRACT**			
Structured summary	2	Provide a structured summary including, as applicable:<br>**Background:** main objectives<br>**Methods:** data sources; study eligibility criteria, participants, and interventions; study appraisal; and *synthesis methods, such as network meta-analysis.*<br>**Results:** number of studies and participants identified; summary estimates with corresponding confidence/credible intervals; *treatment rankings may also be discussed. Authors may choose to summarize pairwise comparisons against a chosen treatment included in their analyses for brevity.*<br>**Discussion/Conclusions:** limitations; conclusions and implications of findings.<br>**Other:** primary source of funding; systematic review registration number with registry name.	3-4
**INTRODUCTION**			
Rationale	3	Describe the rationale for the review in the context of what is already known*, including mention of why a network meta-analysis has been conducted.*	5
Objectives	4	Provide an explicit statement of questions being addressed, with reference to participants, interventions, comparisons, outcomes, and study design (PICOS).	5
**METHODS**			
Protocol and registration	5	Indicate whether a review protocol exists and if and where it can be accessed (e.g., Web address); and, if available, provide registration information, including registration number.	5, Appendix 1
Eligibility criteria	6	Specify study characteristics (e.g., PICOS, length of follow-up) and report characteristics (e.g., years considered, language, publication status) used as criteria for eligibility, giving rationale. *Clearly describe eligible treatments included in the treatment network, and note whether any have been clustered or merged into the same node (with justification).*	6, Appendix 1
Information sources	7	Describe all information sources (e.g., databases with dates of coverage, contact with study authors to identify additional studies) in the search and date last searched.	6, Appendix 1

For peer review only

Search	8	Present full electronic search strategy for at least one database, including any limits used, such that it could be repeated.	N/A (see published protocol)
Study selection	9	State the process for selecting studies (i.e., screening, eligibility, included in systematic review, and, if applicable, included in the meta-analysis).	6, Appendix 1
Data collection process	10	Describe method of data extraction from reports (e.g., piloted forms, independently, in duplicate) and any processes for obtaining and confirming data from investigators.	6, Appendix 1
Data items	11	List and define all variables for which data were sought (e.g., PICOS, funding sources) and any assumptions and simplifications made.	6, Appendix 1
**Geometry of the network**	**S1**	Describe methods used to explore the geometry of the treatment network under study and potential biases related to it. This should include how the evidence base has been graphically summarized for presentation, and what characteristics were compiled and used to describe the evidence base to readers.	7, Appendix 1
Risk of bias within individual studies	12	Describe methods used for assessing risk of bias of individual studies (including specification of whether this was done at the study or outcome level), and how this information is to be used in any data synthesis.	6, Appendix 1
Summary measures	13	State the principal summary measures (e.g., risk ratio, difference in means). *Also describe the use of additional summary measures assessed, such as treatment rankings and surface under the cumulative ranking curve (SUCRA) values, as well as modified approaches used to present summary findings from meta-analyses.*	7, Appendix 1
Planned methods of analysis	14	Describe the methods of handling data and combining results of studies for each network meta-analysis. This should include, but not be limited to:<br>• *Handling of multi-arm trials;*<br>• *Selection of variance structure;*<br>• *Selection of prior distributions in Bayesian analyses; and*<br>• *Assessment of model fit.*	7, Appendix 1
**Assessment of Inconsistency**	**S2**	Describe the statistical methods used to evaluate the agreement of direct and indirect evidence in the treatment network(s) studied. Describe efforts taken to address its presence when found.	7, Appendix 1
Risk of bias across studies	15	Specify any assessment of risk of bias that may affect the cumulative evidence (e.g., publication bias, selective reporting within studies).	6, Appendix 1
Additional analyses	16	Describe methods of additional analyses if done, indicating which were pre-specified. This may include, but not be limited to, the following:<br>• Sensitivity or subgroup analyses;<br>• Meta-regression analyses;<br>• *Alternative formulations of the treatment network; and*<br>• *Use of alternative prior distributions for Bayesian analyses (if applicable).*	7, Appendix 1

For peer review only

RESULTS†

Study selection	17	Give numbers of studies screened, assessed for eligibility, and included in the review, with reasons for exclusions at each stage, ideally with a flow diagram.	7 – Figure 1
**Presentation of network structure**	**S3**	Provide a network graph of the included studies to enable visualization of the geometry of the treatment network.	9 – Figure 2
**Summary of network geometry**	**S4**	Provide a brief overview of characteristics of the treatment network. This may include commentary on the abundance of trials and randomized patients for the different interventions and pairwise comparisons in the network, gaps of evidence in the treatment network, and potential biases reflected by the network structure.	7-8
Study characteristics	18	For each study, present characteristics for which data were extracted (e.g., study size, PICOS, follow-up period) and provide the citations.	8 – Table 1, Appendix 5
Risk of bias within studies	19	Present data on risk of bias of each study and, if available, any outcome level assessment.	8-9 – Appendix 8
Results of individual studies	20	For all outcomes considered (benefits or harms), present, for each study: 1) simple summary data for each intervention group, and 2) effect estimates and confidence intervals. *Modified approaches may be needed to deal with information from larger networks.*	Appendices 6 and 10 (full data can be provided by the first author)
Synthesis of results	21	Present results of each meta-analysis done, including confidence/credible intervals. *In larger networks, authors may focus on comparisons versus a particular comparator (e.g. placebo or standard care), with full findings presented in an appendix. League tables and forest plots may be considered to summarize pairwise comparisons.* If additional summary measures were explored (such as treatment rankings), these should also be presented.	9-11 – Appendix 15
**Exploration for inconsistency**	**S5**	Describe results from investigations of inconsistency. This may include such information as measures of model fit to compare consistency and inconsistency models, *P* values from statistical tests, or summary of inconsistency estimates from different parts of the treatment network.	9 - Appendix 14
Risk of bias across studies	22	Present results of any assessment of risk of bias across studies for the evidence base being studied.	9-11 - Appendix 12
Results of additional analyses	23	Give results of additional analyses, if done (e.g., sensitivity or subgroup analyses, meta-regression analyses*, alternative network geometries studied, alternative choice of prior distributions for Bayesian analyses,* and so forth).	9-11 - Appendices 16 and 17

**DISCUSSION**			
Summary of evidence	24	Summarize the main findings, including the strength of evidence for each main outcome; consider their relevance to key groups (e.g., healthcare providers, users, and policy-makers).	11-13
Limitations	25	Discuss limitations at study and outcome level (e.g., risk of bias), and at review level (e.g., incomplete retrieval of identified research, reporting bias). *Comment on the validity of the assumptions, such as transitivity and consistency. Comment on any concerns regarding network geometry (e.g., avoidance of certain comparisons).*	13-14
Conclusions	26	Provide a general interpretation of the results in the context of other evidence, and implications for future research.	13-14
**FUNDING**			
Funding	27	Describe sources of funding for the systematic review and other support (e.g., supply of data); role of funders for the systematic review. This should also include information regarding whether funding has been received from manufacturers of treatments in the network and/or whether some of the authors are content experts with professional conflicts of interest that could affect use of treatments in the network.	15

PICOS = population, intervention, comparators, outcomes, study design.
* Text in italics indicateS wording specific to reporting of network meta-analyses that has been added to guidance from the PRISMA statement.
† Authors may wish to plan for use of appendices to present all relevant information in full detail for items in this section.

For peer review only

PRISMA-IPD Checklist of items to include when reporting a systematic review and meta-analysis of individual participant data (IPD)

PRISMA-IPD Section/topic	Item No	Checklist item	Reported on page
**Title**			
Title	1	Identify the report as a systematic review and meta-analysis of individual participant data.	1
**Abstract**			
Structured summary	2	Provide a structured summary including as applicable:	3-4
		**Background**: state research question and main objectives, with information on participants, interventions, comparators and outcomes.	
		**Methods**: report eligibility criteria; data sources including dates of last bibliographic search or elicitation, noting that IPD were sought; methods of assessing risk of bias.	
		**Results**: provide number and type of studies and participants identified and number (%) obtained; summary effect estimates for main outcomes (benefits and harms) with confidence intervals and measures of statistical heterogeneity. Describe the direction and size of summary effects in terms meaningful to those who would put findings into practice.	
		**Discussion:** state main strengths and limitations of the evidence, general interpretation of the results and any important implications.	
		**Other:** report primary funding source, registration number and registry name for the systematic review and IPD meta-analysis.	
**Introduction**			
Rationale	3	Describe the rationale for the review in the context of what is already known.	5
Objectives	4	Provide an explicit statement of the questions being addressed with reference, as applicable, to participants, interventions, comparisons, outcomes and study design (PICOS). Include any hypotheses that relate to particular types of participant-level subgroups.	5
**Methods**			

For peer review only

Protocol and registration	5	Indicate if a protocol exists and where it can be accessed. If available, provide registration information including registration number and registry name. Provide publication details, if applicable.	5, Appendix 1
Eligibility criteria	6	Specify inclusion and exclusion criteria including those relating to participants, interventions, comparisons, outcomes, study design and characteristics (e.g. years when conducted, required minimum follow-up). Note whether these were applied at the study or individual level i.e. whether eligible participants were included (and ineligible participants excluded) from a study that included a wider population than specified by the review inclusion criteria. The rationale for criteria should be stated.	5-6, Appendix 1
Identifying studies - information sources	7	Describe all methods of identifying published and unpublished studies including, as applicable: which bibliographic databases were searched with dates of coverage; details of any hand searching including of conference proceedings; use of study registers and agency or company databases; contact with the original research team and experts in the field; open adverts and surveys. Give the date of last search or elicitation.	6, Appendix 1
Identifying studies - search	8	Present the full electronic search strategy for at least one database, including any limits used, such that it could be repeated.	N/A (see published protocol)
Study selection processes	9	State the process for determining which studies were eligible for inclusion.	6, Appendix 1
Data collection processes	10	Describe how IPD were requested, collected and managed, including any processes for querying and confirming data with investigators. If IPD were not sought from any eligible study, the reason for this should be stated (for each such study).	6, Appendix 1
		If applicable, describe how any studies for which IPD were not available were dealt with. This should include whether, how and what aggregate data were sought or extracted from study reports and publications (such as extracting data independently in duplicate) and any processes for obtaining and confirming these data with investigators.	
Data items	11	Describe how the information and variables to be collected were chosen. List and define all study level and participant level data that were sought, including baseline and follow-up information. If applicable, describe methods of standardising or translating variables within the IPD datasets to ensure common scales or measurements across studies.	6, Appendix 1

For peer review only

IPD integrity	A1	Describe what aspects of IPD were subject to data checking (such as sequence generation, data consistency and completeness, baseline imbalance) and how this was done.	Appendix 1
Risk of bias assessment in individual studies.	12	Describe methods used to assess risk of bias in the individual studies and whether this was applied separately for each outcome. If applicable, describe how findings of IPD checking were used to inform the assessment. Report if and how risk of bias assessment was used in any data synthesis.	6, Appendix 1
Specification of outcomes and effect measures	13	State all treatment comparisons of interests. State all outcomes addressed and define them in detail. State whether they were pre-specified for the review and, if applicable, whether they were primary/main or secondary/additional outcomes. Give the principal measures of effect (such as risk ratio, hazard ratio, difference in means) used for each outcome.	7, Appendix 1
Synthesis methods	14	Describe the meta-analysis methods used to synthesise IPD. Specify any statistical methods and models used. Issues should include (but are not restricted to):<br>• Use of a one-stage or two-stage approach.<br>• How effect estimates were generated separately within each study and combined across studies (where applicable).<br>• Specification of one-stage models (where applicable) including how clustering of patients within studies was accounted for.<br>• Use of fixed or random effects models and any other model assumptions, such as proportional hazards.<br>• How (summary) survival curves were generated (where applicable).<br>• Methods for quantifying statistical heterogeneity (such as I^2 and τ^2).<br>• How studies providing IPD and not providing IPD were analysed together (where applicable).<br>• How missing data within the IPD were dealt with (where applicable).	7, Appendix 1
Exploration of variation in effects	A2	If applicable, describe any methods used to explore variation in effects by study or participant level characteristics (such as estimation of interactions between effect and covariates). State all participant-level characteristics that were analysed as potential effect modifiers, and whether these were pre-specified.	Appendix 1
Risk of bias across studies	15	Specify any assessment of risk of bias relating to the accumulated body of evidence, including any pertaining to not obtaining IPD for particular studies, outcomes or other variables.	6, Appendix 1

For peer review only

Additional analyses	16	Describe methods of any additional analyses, including sensitivity analyses. State which of these were pre-specified.	7, Appendix 1
**Results**			
Study selection and IPD obtained	17	Give numbers of studies screened, assessed for eligibility, and included in the systematic review with reasons for exclusions at each stage. Indicate the number of studies and participants for which IPD were sought and for which IPD were obtained. For those studies where IPD were not available, give the numbers of studies and participants for which aggregate data were available. Report reasons for non-availability of IPD. Include a flow diagram.	7 – Figure 1
Study characteristics	18	For each study, present information on key study and participant characteristics (such as description of interventions, numbers of participants, demographic data, unavailability of outcomes, funding source, and if applicable duration of follow-up). Provide (main) citations for each study. Where applicable, also report similar study characteristics for any studies not providing IPD.	8 – Table 1, Appendix 5
IPD integrity	A3	Report any important issues identified in checking IPD or state that there were none.	8-9, Appendices 5 and 10
Risk of bias within studies	19	Present data on risk of bias assessments. If applicable, describe whether data checking led to the up-weighting or down-weighting of these assessments. Consider how any potential bias impacts on the robustness of meta-analysis conclusions.	8-9 – Appendix 8
Results of individual studies	20	For each comparison and for each main outcome (benefit or harm), for each individual study report the number of eligible participants for which data were obtained and show simple summary data for each intervention group (including, where applicable, the number of events), effect estimates and confidence intervals. These may be tabulated or included on a forest plot.	Appendices 6 and 10 (full data can be provided by the

For peer review only

			first author)
Results of syntheses	21	Present summary effects for each meta-analysis undertaken, including confidence intervals and measures of statistical heterogeneity. State whether the analysis was pre-specified, and report the numbers of studies and participants and, where applicable, the number of events on which it is based.	9-11 – Appendix 15
		When exploring variation in effects due to patient or study characteristics, present summary interaction estimates for each characteristic examined, including confidence intervals and measures of statistical heterogeneity. State whether the analysis was pre-specified. State whether any interaction is consistent across trials.	
		Provide a description of the direction and size of effect in terms meaningful to those who would put findings into practice.	
Risk of bias across studies	22	Present results of any assessment of risk of bias relating to the accumulated body of evidence, including any pertaining to the availability and representativeness of available studies, outcomes or other variables.	9-11 - Appendix 12
Additional analyses	23	Give results of any additional analyses (e.g. sensitivity analyses). If applicable, this should also include any analyses that incorporate aggregate data for studies that do not have IPD. If applicable, summarise the main meta-analysis results following the inclusion or exclusion of studies for which IPD were not available.	9-11 - Appendices 16 and 17
**Discussion**			
Summary of evidence	24	Summarise the main findings, including the strength of evidence for each main outcome.	11-13
Strengths and limitations	25	Discuss any important strengths and limitations of the evidence including the benefits of access to IPD and any limitations arising from IPD that were not available.	13-14
Conclusions	26	Provide a general interpretation of the findings in the context of other evidence.	13-14
Implications	A4	Consider relevance to key groups (such as policy makers, service providers and service users). Consider implications for future research.	12-13

For peer review only

Funding			
Funding	27	Describe sources of funding and other support (such as supply of IPD), and the role in the systematic review of those providing such support.	15

A1 – A3 denote new items that are additional to standard PRISMA items. A4 has been created as a result of re-arranging content of the standard PRISMA statement to suit the way that systematic review IPD meta-analyses are reported.

© Reproduced with permission of the PRISMA IPD Group, which encourages sharing and reuse for non-commercial purposes

For peer review only
